# Pattern Analysis of Organellar Maps for Interpretation of Proteomic Data

**DOI:** 10.3390/proteomes10020018

**Published:** 2022-05-23

**Authors:** Jordan B. Burton, Nicholas J. Carruthers, Zhanjun Hou, Larry H. Matherly, Paul M. Stemmer

**Affiliations:** 1 Institute of Environmental Health Sciences, Wayne State University, Detroit, MI 48202, USA; jordan.burton@wayne.edu; 2 Bioinformatics Core, University of Michigan, Ann Arbor, MI 48109, USA; ncarruth@med.umich.edu; 3 Department of Oncology, Karmanos Cancer Institute, Wayne State University, Detroit, MI 48202, USA; houz@karmanos.org (Z.H.); matherly@karmanos.org (L.H.M.)

**Keywords:** proteomics, pattern analysis, bioinformatics, proximity labeling, exosomes

## Abstract

Localization of organelle proteins by isotope tagging (LOPIT) maps are a coordinate-directed representation of proteome data that can aid in biological interpretation. Analysis of organellar association for proteins as displayed using LOPIT is evaluated and interpreted for two types of proteomic data sets. First, test and control group protein abundances and fold change data obtained in a proximity labeling experiment are plotted on a LOPIT map to evaluate the likelihood of true protein interactions. Selection of true positives based on co-localization of proteins in the organellar space is shown to be consistent with carboxylase enrichment which serves as a positive control for biotinylation in streptavidin affinity selected proteome data sets. The mapping in organellar space facilitates discrimination between the test and control groups and aids in identification of proteins of interest. The same representation of proteins in organellar space is used in the analysis of extracellular vesicle proteomes for which protein abundance and fold change data are evaluated. Vesicular protein organellar localization patterns provide information about the subcellular origin of the proteins in the samples which are isolates from the extracellular milieu. The organellar localization patterns are indicative of the provenance of the vesicular proteome origin and allow discrimination between proteomes prepared using different enrichment methods. The patterns in LOPIT displays are easy to understand and compare which aids in the biological interpretation of proteome data.

## 1. Introduction

Using pattern recognition to aid in the understanding of protein function is an underutilized approach for biological interpretation of proteomic data. Patterns arise in proteome data sets because the functions of proteins in the samples are related to specific chemical environments and interaction partners present in subcellular locations and organelles where those protein reside [1]. Localization of organelle proteins by isotope tagging (LOPIT) was introduced in 2004 by Dunkley and coworkers to establish the subcellular localization of proteins by using differential centrifugation to fractionate organellar proteomes based on density and then mass spectrometry to identify proteins that localize in organellar fractions [2]. The LOPIT data, annotated with subcellular assignments made via machine learning, were input into a t-distributed stochastic neighbor embedding (t-SNE) algorithm to reduce the dimensionality of the data and to map the subcellular structures, protein complexes, and signaling pathway localizations of isoform-specific proteins with high accuracy [3,4,5]. Using the coordinates from the published LOPIT distributions and mapping proteome data from unrelated experiments to the organellar space that has previously been reported facilitates the identification and analysis of patterns that are useful in biological interpretation of proteome data from non-related experiments.

LOPIT data and distribution maps are publicly available through the pRoloc R-package (http://bioconductor.org/packages/pRoloc (accessed on 5 August 2021)), which contains 35 datasets [6,7]. The pRoloc R-package is well documented, easy to use, and contains functions for the visualization of LOPIT data. LOPIT maps provide a coordinate-directed way to identify proteins of biological interest [8]. The distribution of proteins in organellar assignment space for proteomic data sets allows insight into the underlying biology that is not provided by other representations of the data. LOPIT map protein distributions are included in the Human Protein Atlas and the subcellular and organellar locations for many proteins are confirmed by immunofluorescent imaging [9]. Proteins identified as highly abundant that do not reside in a clearly assigned organellar space can be searched in the Human Protein Atlas to determine the localization assignment based on immunohistochemical imaging. Addition of quantitative values for proteins in the data sets plotted on a LOPIT map adds a dimension in addition to the localization that highlights the importance of a subset of the proteins that differ in abundance from the reference proteome.

**Figure 1:**
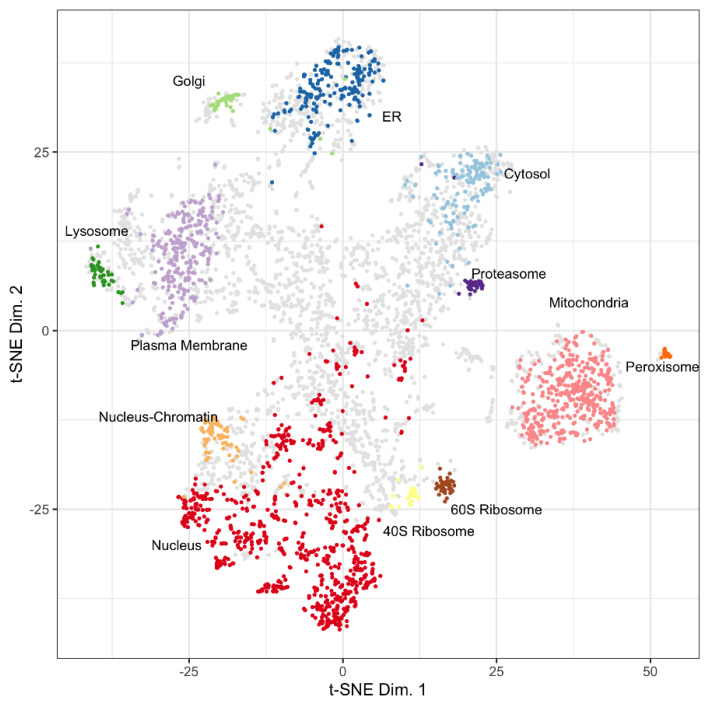
Distribution of LOPIT localized proteins for u2os cells from Thul, Akeson et al., 2017. Assigned coordinates for proteins in the cytosol (light blue), endoplasmic reticulum (ER, dark blue), golgi (light green), lysosome (dark green), mitochondria (pink), nucleus (red), nucleus-chromatin (light orange), peroxisome (dark orange), plasma membrane (light purple), proteasome (dark purple), ribosome 40S (yellow), ribosome 60S (brown), and unknown (grey) are shown. Reproduced with permission from Science.

A t-SNE plot of 5020 proteins from u2os cell lysates plotted using t-SNE coordinate assignments to 13 subcellular locations using Localization of Organelle Proteins by Isotope Tagging (LOPIT) is shown in Figure 1 (Thul, Akeson et al. 2017) [4]. Several proteins in the t-SNE plot have an undefined origin, occupying the central space between the designated organellar locations. Proteins have an organellar assignment if they were co-isolated experimentally with proteins that define a certain organelle or if they have similar characteristics to organelle protein markers. Proteins have an unknown assignment in the hyperLOPIT data if they are present in multiple fractions during the density gradient centrifugation and do not have similar characteristics to established organelle protein markers.

Pattern recognition has value in interpretation of proximity labeling experiments as demonstrated by the publicly available resource for mapping proteins in a streptavidin affinity selection experiment at www.humancellmap.org (accessed on 15 August 2021) [10]. BioID proximity labeling experiments use a promiscuous BirA biotin ligase expressed as a fusion with a protein of interest in order to enzymatically attach biotin to interacting proteins [11]. Proteins that are biotinylated by the BirA are isolated using streptavidin columns or beads. A major obstacle to interpretation of proximity labeling data is the large number of background proteins detected [12]. For these data sets, a close association of an identified protein with the target protein on the LOPIT map increases the confidence that it is a true positive. Proteins that are assigned to an organellar space far from the bait protein have an increased likelihood of being false positives and, minimally, should be evaluated with the understanding that the connection between the bait protein and the protein that is potentially of interest is unexpected.

Extracellular vesicles are important vehicles of intercellular communication that can potentially be used for disease identification, assessment of response to therapy, and for drug delivery [13,14]. Identifying low abundance proteins in the vesicle preparations via mass spectrometry has emerged as a powerful tool in biomarker discovery and in epidemiological studies [15]. It remains a challenge to enrich vesicles from plasma, urine, and other biological matrixes. The consistency of the vesicle enrichment and the degree to which the vesicles are enriched have major influence on the interpretation of data subsequently obtained with the preparations [16]. The provenance of the vesicles being examined is critical to identifying the role of the identified proteins in the biology or disease process being studied. However, determining the provenance of isolated vesicles is challenging because exosomes, micro vesicles, and apoptotic bodies carry mixtures of proteins that are not specific to the biogenesis pathway of any one vesicle type [17]. Localization patterns of proteomes from extracellular vesicle preparations on LOPIT maps aids in establishing the provenance of proteomes from isolated vesicles and establishing the effectiveness of vesicle enrichment.

This work emphasizes the utility of LOPIT maps for the identification of patterns in a proximity labeling experiment and for the assessment of extracellular vesicle preparations. The LOPIT maps allow unambiguous distinction between the test and control groups in a proximity labeling experiment. In addition, the identification of proteins of interest in a BioID experiment is refined using LOPIT-based patterns. Finally, the use of LOPIT plots to validate the enrichment of vesicular proteins by mapping the vesicular proteins to the LOPIT coordinates from the t-SNE plot is demonstrated.

## 2. Materials and Methods

### 2.1. Biotin Affinity Selection

#### 2.1.1. Biotin Proximity Labeling Workflow

The human proton-coupled folate transporter (PCFT, SLC46A1) [18,19] was stably expressed as a fusion protein with a BioID2-HA [20] in the C-terminus at residue 459 in HeLa cells (PCFT-BioID2). The uncomplexed biotin transferase was used as a control to identify proteins that are biotinylated randomly due to sheer abundance and/or an unnatural affinity to the BioID2 ligase. HeLa PCFT-BioID2 cells expressed the PCFT fusion protein which localized to the plasma membrane and exhibited robust transport typical of PCFT. HeLa PCFT-BioID2 and BioID2 cells were cultured in complete DMEM (biotin-free), with and without 50 µM biotin (Sigma-Aldrich Inc., St. Louis, MO, USA) for the final 16 h in culture, then biotinylated proteins were selected using streptavidin beads.

**Figure 2:**
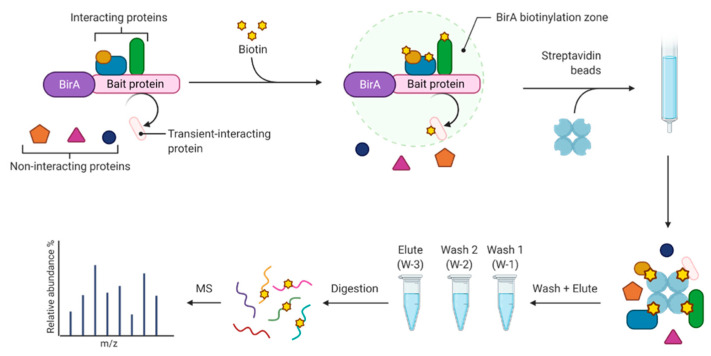
Biotin proximity labeling workflow.

Figure 2 shows the biotin proximity labeling workflow that was adapted [20]. In the proton-coupled folate transporter group (PCFT), the bait protein, PCFT (Q96NT5), was expressed as a fusion with BirA in order to label interaction partners with biotin. In the ground reference group (GR) BirA was expressed independently and was not localized to any subcellular region. Cells were incubated with 50 µM biotin for 16 h, then washed 3 times with 1X phosphate-buffered saline (PBS) (Sigma-Aldrich Inc., St. Louis, MO, USA) and lysed in 1.5 mL of buffer containing 50 mM Tris (Sigma-Aldrich Inc., St. Louis, MO, USA), pH 7.4; 500 mM NaCl (Sigma-Aldrich Inc., St. Louis, MO, USA); 0.4% (*w*/*v*) sodium dodecyl sulfate (Sigma-Aldrich Inc., St. Louis, MO, USA), 1 mM dithiothreitol (Sigma-Aldrich Inc., St. Louis, MO, USA), and 1X complete protease inhibitor (Roche Diagnostics Deutschland GmbH, Mannheim, Germany). After collecting the cells, Triton X-100 (Sigma-Aldrich Inc., St. Louis, MO, USA) was added to a 2% (*w*/*v*) final concentration. Cells were sonicated twice for 1 min at a 30% duty cycle with an output level of 4 (Sonifer-250; Branson Ultrasonics Corporation, Danbury, CT, USA) and an equal volume of 50 mM Tris, pH 7.4 was added before centrifugation at 16,500× *g* for 10 min. The supernatant was collected in a 15 mL conical tube and incubated with 200 µL pre-washed streptavidin-coated Dynabeads (Invitrogen, Carlsbad, CA, USA) overnight with rotation at 4 °C.

The Dynabeads were washed to remove background proteins by mixing 100 µL of 1% (*w*/*v*) lithium dodecyl sulfate (Sigma-Aldrich Inc., St. Louis, MO, USA), 2X PBS with the 100 µL bed volume of beads at room temperature. Beads were collected using a magnetic stand and the supernatant was collected as Wash 1. The beads were washed a second time to remove background proteins. The second wash was 200 µL 1% (*w*/*v*) unbuffered lithium dodecyl sulfate at room temperature [21]. The supernatant from the low ionic strength wash was collected as Wash 2. The beads were washed further with 50 µL of 2X PBS and the supernatant was combined with Wash 2. Biotinylated proteins were eluted from the Dynabeads with 50 μL of water followed by incubating the beads 3 times in 50 µL 1% (*w*/*v*) lithium dodecyl sulfate, 25 µM biotin at 65 °C for 5 min before collecting the supernatant as the Eluate.

Wash 1, Wash 2, and Elute samples were reduced and alkylated in 1% (*w*/*v*) lithium docecyl sulfate buffered with 20 mM TEAB (Sigma-Aldrich Inc., St. Louis, MO, USA), by incubation with 5 mM dithiothreitol (Sigma-Aldrich Inc., St. Louis, MO, USA), for 30 min at 56 °C followed by incubation with 15 mM iodoacetamide (Sigma-Aldrich Inc., St. Louis, MO, USA), for 30 min at room temperature. Samples were acidified by adding 10% of the original volume of 12% phosphoric acid (Sigma-Aldrich Inc., St. Louis, MO, USA), and precipitated by adding 7 volumes of 90% methanol (Sigma-Aldrich Inc., St. Louis, MO, USA), 10% TEAB. Precipitated proteins were collected by centrifugation and washed twice with 0.5 mL 90% methano1, 1% TEAB to remove residual detergent. The precipitate was resuspended in 40 mM HEPES, pH 7.9 before adding 0.1 µg Promega Trypsin and incubating for 1 h at 47 °C then 6 h at 37 °C.

#### 2.1.2. Liquid Chromatography Mass Spectrometry

Peptides from sample digests were loaded onto an Acclaim PepMap 75 µm × 2 cm C-18 trap (Thermo Fisher Scientific, Waltham, MA, USA and separated using an Acclaim PepMap RSLC 75 µm × 25 cm C-18 column with gradients generated by an Easy-1000 nanoLC (Thermo Fisher Scientific, Waltham, MA, USA coupled to a Fusion Tribrid Mass Spectrometer (Thermo Fisher Scientific, Waltham, MA, USA. Peptides were separated on a 50 min gradient with a flow rate of 250 nL/min. The gradient started at 94% Solvent A (0.1% formic acid) (Thermo Fisher Scientific, Waltham, MA, USA and was ramped to 12% Solvent B (99.9% Acetonitrile (Sigma-Aldrich Inc., St. Louis, MO, USA), 0.1% Formic Acid) (Thermo Fisher Scientific, Waltham, MA, USA for 12 min, then ramped to 20% Solvent B for 12 min, then to 28% Solvent B for 8 min, 40% Solvent B for 5 min, 95% Solvent B for 1 min, and held at 95% Solvent B for 12 min. The mass spectrometer was operated in positive ion mode and data dependent acquisition. Precursor ions were detected at 120 K resolution in the orbitrap with a scan range of 350–1600 *m*/*z*, maximum injection time of 100 ms, 1e6 AGC target, and 250% normalized AGC target. Precursor ions were excluded for 15 s after the first acquisition. Precursor ions above a minimum intensity of 3000 and charge states from 2–7 were selected for isolation and fragmentation using CID set at 29, 10 ms activation time, and 0.25 activation Q. Fragment ions were detected in the ion trap with a rapid scan rate and 100 ms maximum injection time. The AGC target was set to 1e5 with a 100% normalized AGC target.

#### 2.1.3. Database Searching

Data were searched with Proteome Discoverer (v. 2.4) (Thermo Fisher Scientific, Waltham, MA, USA) against a human reference database (UniProt, accessed on 21 December 2018) using Sequest HT. The enzyme was set to trypsin allowing up to 2 missed cleavages. The precursor mass tolerance was set to 20 ppm and the fragment mass tolerance was set to 0.5 Da. Dynamic modifications were oxidation of methionine as well as deamidation of asparagine and glutamine. Carbamidomethylation of cysteine was set as a static modification. The false discovery rate was calculated using the target decoy PSM validator node with concatenated scores. Match between runs was enabled for each sample type and quantitation was made using precursor ion intensities.

### 2.2. Publicly Available Data

#### 2.2.1. LOPIT Data

The LOPIT maps used in this work were generated using the pRoloc R package after downloading the hyperLOPIT2017 u2os dataset using the pRolocData R package (Bioconductor) [4,6,7,9]. As described in those papers, to create the LOPIT organellar protein distribution map a crude membrane preparation from a u2os cell lysate was fractionated by density gradient ultracentrifugation to separate and enrich organelles based on density. After quantitative mass spectrometry proteins were assigned to organelles based on similarities in distribution in the density gradient to well-annotated organelle protein markers. The t-SNE machine learning algorithm was used to reduce the number of dimensions in the LOPIT proteomic data to a 2D map where proteins cluster by similarity from multiple experimental factors [3]. All LOPIT protein coordinates were used as originally determined and without additional refinement [4].

#### 2.2.2. Exosome Enrichment by Density Gradient Ultracentrifugation

The dataset S1 from the supplementary material published by Kowal et al. was selected as a benchmark for a highly enriched exosomal vesicle proteome [13]. Briefly, Kowal et al. prepared extracellular vesicles from human primary monocyte-derived dendritic cells by centrifugation at 10,000× *g* (10 K) or 100,000× *g* (100 K). Fraction 3 (F3) and Fraction 5 (F5) from the density gradient contained most of the extracellular vesicles and exosomes as determined by Western blot analysis of exosome marker proteins including the tetraspanins CD9, CD63, and CD81. F3 had an average density of 1.115 g/mL and contained vesicles of 50–150 nm in diameter. F5 had an average density of 1.145 g/mL and contained vesicles over 150 nm in diameter.

#### 2.2.3. Exosome Enrichment by Size Exclusion Chromatography, Density Gradient Ultracentrifugation, and Ultracentrifugation

Supplementary Tables S1–S6 from Kugeratski et al. were downloaded for use in this analysis. The files contain results from mass spectrometry analysis that was intended to establish a proposed set of core exosome proteins. Proteins are attributed to vesicular origin for preparations from 14 cell lines enriched by size exclusion chromatography and for 3 cell lines after enrichment by ultracentrifugation, size exclusion chromatography, or density gradient ultracentrifugation [14].

### 2.3. Data Processing, Visualization, and Availability

All data were imported into R Studio (version 4.0.5, https://www.rstudio.com/products/rstudio/download/#download, accessed on 31 March 2021) for processing and visualization. The R scripts for data processing and visualization have been made publicly available at https://zenodo.org/record/5851697 (accessed on 31 March 2021). Those R scripts take group protein abundance data then bin them before overlaying the abundance information using the protein specific coordinates for the LOPIT map from Thul et al. The mass spectrometry proteomics data have been deposited to the ProteomeXchange Consortium via the PRIDE partner repository with the dataset identifier PXD032297 and 10.6019/PXD032297.

## 3. Results

### 3.1. Mapping Proteins from a Proximity Labeling Experiment to the LOPIT Plot

Streptavidin affinity selection of biotinylated proteins following expression of a promiscuous BirA fusion was designed to facilitate discovery of transient and low affinity protein interactions. In practice, the number of proteins identified in these experiments range into the low thousands, a high background that makes interpretation of the data problematic. The experimental design incorporates stringent washes of the sample-loaded streptavidin beads with detergents as the streptavidin-biotin interaction is considered to be very stable. However, biotin binding to streptavidin is reversible and the affinity is highly dependent on the ionic strength and temperature [21]. Biotinylated proteins are expected to have a slow off rate for release from streptavidin under wash conditions with high ionic strength and low temperature. The off rate for the biotinylated proteins increases with increased temperature and in low ionic strength buffer. Efficient recovery of biotinylated proteins is promoted by including free biotin in the elution buffer to prevent protein rebinding.

The degree to which biotinylated proteins are lost in the washes and obtained in the elution steps is monitored by measuring carboxylase enzymes. Carboxylases serve as an endogenous positive control for the affinity selection on streptavidin as they are the normal substrate for biotin transferase enzymes in mammalian cells [22]. Carboxylases are typically detected in the wash solutions demonstrating that the wash is sufficiently stringent to disrupt the biotin streptavidin interaction to some degree. By monitoring the abundance of the carboxylases in both the final wash and the elution fractions, a wash to elute ratio for the abundance of known biotinylated proteins can be calculated and used as a benchmark in evaluating candidate proteins. The four carboxylases quantified as positive control for streptavidin selection are pyruvate carboxylase, mitochondrial (P11498); propionyl-CoA carboxylase alpha chain, mitochondrial (P05165); methylcrotonoyl-CoA carboxylase subunit alpha, mitochondrial (Q96RQ3); and acetyl-CoA carboxylase 1 (Q13085).

#### 3.1.1. Protein Abundances

**Figure 3:**
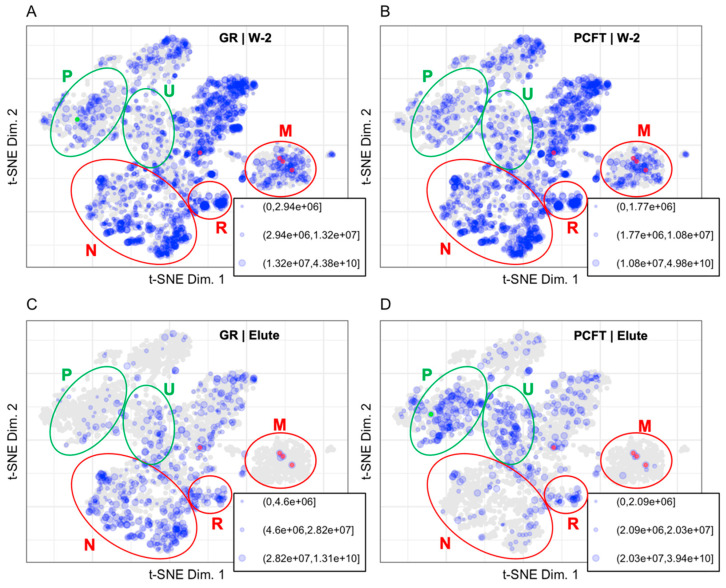
Binned protein abundances are mapped to the LOPIT coordinates for (**A**) W-2 GR (1896 proteins), (**B**) W-2 PCFT (1766 proteins), (**C**) Elute GR (593 proteins), and (**D**) Elute PCFT (500 proteins). Size indicates relative protein abundance. PCFT is highlighted in green. Carboxylases were identified in all fractions and are highlighted in red. Regions for plasma membrane (P) and undefined (U) are shown in green ellipses to represent enrichment in the PCFT elute. Regions for nuclear (N), ribosomal (R), and mitochondrial (M) are shown in red ellipses to represent background in the PCFT elute.

Abundance of proteins identified and quantified in the Wash 1 (W-1), Wash 2 (W-2), and Elute (E) fractions of control and PCFT-BirA groups (Figure 1) were binned. Each group and each fraction are mapped to the protein coordinates determined in the t-SNE plot from Thul et al., 2017, presented in Figure 3 [4]. Table 1 displays the number of proteins identified in each fraction, the number of proteins contained in the LOPIT dataset for each fraction, the number of proteins identified for each group, and the percentage of identified proteins in each group that is present in the LOPIT data. The full data set is available as Supplementary Table S1.

**Figure 4:**
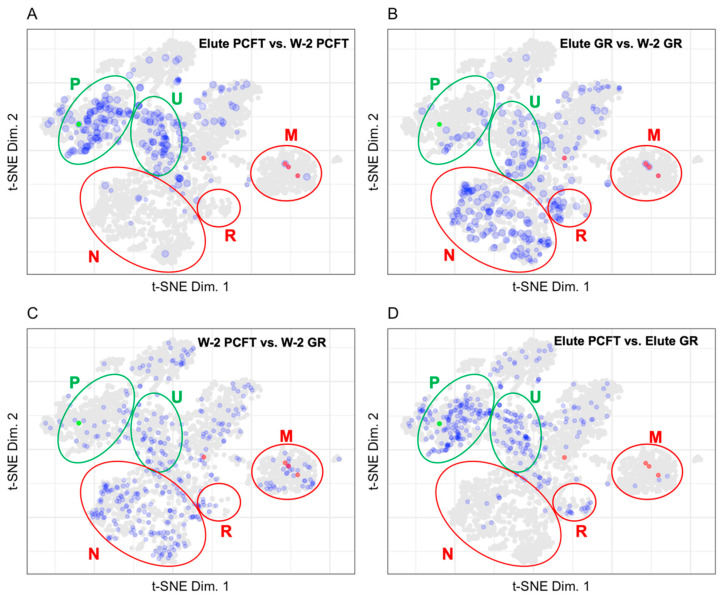
Plots displaying the fold change with a 3X cut-off for (**A**) Elute PCFT vs. W-2 PCFT (238 proteins), (**B**) Elute GR vs. W-2 GR (264 proteins), (**C**) W-2 PCFT vs. W-2 GR (350 proteins), and (**D**) Elute PCFT vs. Elute GR (310 proteins). PCFT is highlighted in green. Carboxylases are highlighted in red. Regions for plasma membrane (P) and undefined (U) are shown in green ellipses to represent enrichment in the PCFT elute. Regions for nuclear (N), ribosomal (R), and mitochondrial (M) are shown in red ellipses to represent background in the PCFT elute.

The PCFT bait protein is expected to localize as an integral plasma membrane protein with a cytosolic C-terminus [23,24]. Inspection of the protein distribution patterns for the Elute fractions in Figure 3 confirms the presence of folate transporter in the PCFT group and the absence of PCFT in the GR group. The decrease in the number of proteins identified in the Elute fraction compared to the W-2 fraction demonstrates the effectiveness of the wash for removing background proteins. The carboxylases are localized to the mitochondrial coordinates and are highlighted by being colored red in Figure 3 and Figure 4. The carboxylase proteins are detected at a lower abundance in the W-2 fraction than in the Elute fraction, demonstrating that biotinylated proteins are largely retained through the wash process and released primarily under elution conditions. The representation of data in Figure 3 allows us to eliminate, or at least reduce interest in, proteins that are identified but that map to organellar space far away from the PCFT.

The high abundance of proteins that map to the cytosol, mitochondria, and nuclear space in the LOPIT maps for the wash indicate that those regions are likely to contain primarily or exclusively background proteins. It is notable that the distribution patterns of the wash fractions for both the PCFT and GR groups are indistinguishable. The carboxylases, which are mitochondrial proteins, are essentially the only mitochondrial proteins that are retained in the elute samples.

#### 3.1.2. Mapping BioID Wash to Elute Ratio Data

The proximity labeling design discussed here uses the ratio of individual protein abundance in the Elute vs. W-2 fractions to identify true interacting proteins. The presence of known biotinylated carboxylase proteins establishes a benchmark ratio for biotinylated proteins. The ratio of the Elute vs. W-2 fraction displayed in Figure 4 was calculated as the Elute fraction protein abundance over the W-2 fraction protein abundance.

As endogenously biotinylated proteins [22], carboxylases should always be detected in the Elute fraction. In practice, carboxylases are also detected in the wash fractions where their abundance gives an indication of the loss of specific binding, which is loss of signal from true positives. Although we assume that the biotin-streptavidin affinity and off rate are constant and independent of the protein that the biotin is coupled to, this has never been evaluated. The carboxylases have a single biotin modification as the endogenous biotin transferase that catalyzes modification of those proteins is specific for a single site on each carboxylase. The promiscuous BirA, however, can and is likely to biotinylate at random and at multiple sites on proteins in closest proximity to the bait. The presence of multiple biotins on any single protein would dramatically slow the loss of those proteins from the streptavidin during washes as the dissociated biotin would have a chance to rebind. Another caveat to using carboxylases as a positive control is that the context of where the biotin is on the protein could change the affinity of the biotin for streptavidin. The ratio of total carboxylase abundance between fractions establishes a minimum ratio necessary to be considered as specific binding during streptavidin selection. The ratio of carboxylase abundance in Elute to W-2 fractions range from 2.1 to 68.8 for the PCFT group and from 6.7 to 83.9 for the GR group. The number of analytes mapped to the LOPIT plot in Figure 4 for the PCFT group is decreased from 1220 to 238. The resultant set of proteins for the PCFT group are localized in the plasma membrane and undefined organellar space whereas the proteins for the GR group, a non-specific control, localize mainly in the nuclear and undefined space. Comparisons of the wash fractions between the PCFT and GR groups in Figure 3A vs. Figure 3B and Figure 4C show no clear differences between the two groups in the wash fractions. This lack of difference in conjunction with the clear differences in organellar localization of proteins for the Elute fractions as seen when comparing Figure 3C,D or in Figure 4D confirms that the criteria established for proteins of interest in the PCFT Elute group are likely to be true positives for PCFT interacting proteins.

### 3.2. Mapping Proteins from Extracellular Vesicle Preparations to the LOPIT Plot

**Figure 5:**
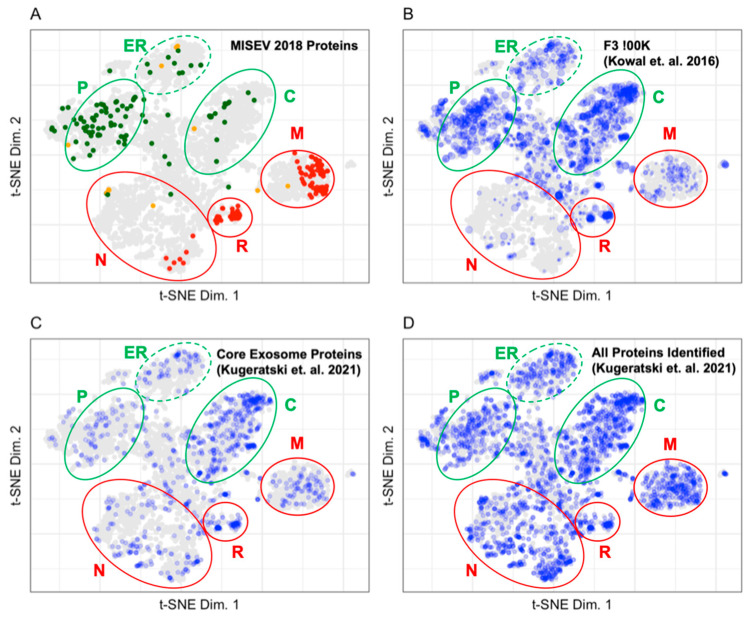
(**A**) Distribution of proteins from the MISEV2018 recommendation. Proteins are colored according to their ISEV interpretation. (**B**) Distribution of proteins from a benchmark exosome preparation enriched in vesicles by density gradient centrifugation (Kowal et. al., F3-100K). (**C**) Distribution of core exosome proteins proposed by Kugeratski et al. (**D**) Distribution of all exosome proteins identified by Kugeratski et al. Blue points indicate that the protein is in the experimental data set. Size indicates protein abundance. The plasma membrane (P), cytosol (C), and endoplasmic reticulum (ER) are circled in green, indicating that these regions contain markers of extracellular vesicles. The nucleus (N), ribosomes (R), and mitochondria (M) are circled in red, indicating that these regions contain markers of contaminant proteins in extracellular vesicle preparations.

Establishing a reliable benchmark for the proteomes of the different extracellular vesicle (EV) types is an ongoing process. The International Society for Extracellular Vesicles (ISEV) publishes recommendations for benchmarking extracellular vesicle preparations in the Minimal Information for Studies of Extracellular Vesicles (MISEV) [17]. In addition, a benchmark proteome for EVs with exosomal characteristics was selected due to the high information content in enrichment process used to obtain that EV population and evaluation data for those vesicles [13]. Figure 5 displays the LOPIT distribution of exosomal marker proteins published in the MISEV2018 recommendation [17], the proteins identified in the high-quality benchmark exosome preparation of vesicles enriched by density gradient centrifugation [13], proposed core exosome proteins identified by, and all proteins identified in, exosome preparations in Kugeratski et al. [14]. Of the 561 proteins proposed as markers for the evaluation of extracellular vesicle sample preparations in the MISEV2018 (Table 2), 48% have LOPIT coordinates [4]. There were 2979 proteins identified in the benchmark exosome preparation by Kowal et al., of which 68% have LOPIT coordinates [13]. There are 1243 proteins proposed as core exosome proteins by Kugeratski et al. after comparing proteomes for vesicles enriched from 14 cell lines. Of those proteins, 73% have LOPIT coordinates as do 39% of the 6491 full set of identified proteins in the exosome preparations from all cells evaluated [14]. The MISEV2018 recommended proteins shown in Figure 5A establish the organellar space for EV assigned and contaminant proteins in the extracellular vesicle preparations [17]. Proteins recommended for use as extracellular vesicle markers by MISEV2018 are shown in green and map to the plasma membrane, lysosome, ER, and cytosol. Proteins recommended for use as contaminant markers by MISEV2018 are shown in red and map to the mitochondria, 60S ribosome, 40S ribosome, and the nucleus.

Proteins in the Kowal et al. and Kugeratski et al. data sets were binned and mapped to the LOPIT plot in Figure 5B–D [13,14]. The F3-100K (Figure 5B) is a high-quality benchmark exosomal preparation. It contains 49 (44.5%), 29 (52.7%), 73 (37.4%), 10 (24.4%), and 8 (5.0%) of the category 1, 2, 3, 4, and 5 proteins as recommended by MISEV2018, respectively. Table 3 shows that highly abundant proteins in the Fraction 3 100K localize predominantly in the cytosol and plasma membrane which corresponds to the subcellular origin of proteins recommended for use as extracellular vesicle markers by MISEV2018. All proteins recommended in the MISEV2018 categories for the evaluation of extracellular vesicle preparations were included in the core exosome proteins (Figure 5C). Table 3 shows that most proteins identified as belonging in the core exosome proteins category map to unknown, nucleus, cytosol, and mitochondria. A minimal number of proteins in the core exosome proteins mapped to the endoplasmic reticulum, 60S ribosome, plasma membrane, proteasome, and 40S ribosome. The core exosome proteins proposed by Kugeratski et al. do not have the same density of proteins identified in the plasma membrane or the endoplasmic reticulum that are associated with exosome production from the endosomal release pathway. The distribution of all proteins identified in exosome preparations from 14 cell lines in Kugeratski et al. is shown in Figure 5D [14]. The distribution of proteins in the nucleus and mitochondria is much denser in preparations from the 14 cell lines described by Kugeratski et al. as compared to the Fraction 3 100K exosome preparation in the Kowal et al. dataset.

**Figure 6:**
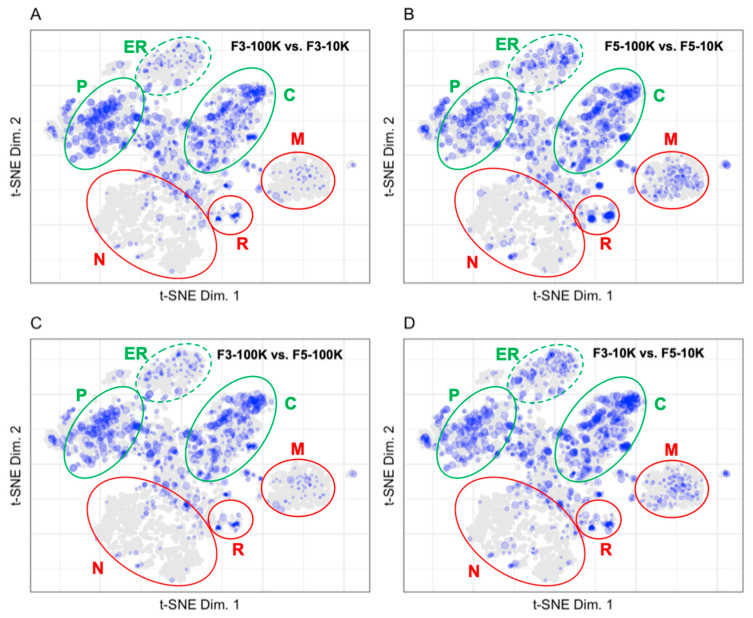
Fold changes between fractions for (**A**) F3-100K vs. F3-10K (951 proteins), (**B**) F5-100K vs. F5-10K (1052 proteins), (**C**) F3-100K vs. F5-100K (893 proteins), and (**D**) F3-10K vs. F5-10K (1120 proteins). The plasma membrane (P), cytosol (C), and endoplasmic reticulum (ER) are circled in green, indicating that these regions contain markers of extracellular vesicles. The nucleus (N), ribosomes (R), and mitochondria (M) are circled in red, indicating that these regions contain markers of contaminant proteins in extracellular vesicle preparations.

The fold change in protein abundances for the different fractions prepared by Kowal et al. were calculated and binned before plotting to LOPIT coordinates in Figure 6 [13]. The size of each point corresponds to the binned protein abundance ratio where larger sized points indicate higher fold changes between fractions. Mapping the fold change in protein abundances between fractions allows us to identify proteins that are enriched to a fraction and proteins that are equally distributed in several fractions. Mapping the fold change in protein abundances between the F3 and F5 fractions after a 10K or 100K ultracentrifugation allows us to demonstrate the difference in patterns of protein abundance between the vesicles of 50–150 nm in diameter found in fraction F3-100K and the larger vesicles that predominate in the F3-10K, F5-10K, and F3-100K fractions [13]. Figure 6A shows the low abundance of endoplasmic reticulum, ribosome, proteasome, and mitochondrial proteins in the F3-100K vesicle preparation as compared to the F3-10K vesicle preparation. This is consistent with the MISEV recommendations shown in Figure 5A that those organelles do not contribute many proteins to exosomal-type vesicles. The F3-100K preparation is enriched in endosomal, plasma membrane, and cytosolic components as compared to the F3-10K preparation. The greater abundance of cytosolic proteins in the F3-100 fraction as compared to the MISEV recommendations suggests that the MISEV set of recommended proteins does not capture the full exosomal proteome or that the abundance of those cytosolic proteins in the F3-100 fraction is characteristic of that particular cell type. Figure 6B demonstrates the enrichment of plasma membrane, endoplasmic reticulum, mitochondrial, and cytosolic components in the F5-100K vesicle preparation as compared to the F5-10K vesicle preparation. Figure 6C demonstrates the enrichment of plasma membrane and cytosolic proteins, as well as the decreased abundance of endoplasmic reticulum and mitochondrial components in the F3-100K vesicle preparation compared to the F5-100K vesicle preparation. A similar pattern to Figure 6C is shown in Figure 6D in the comparison between the F3-10K and F5-100K vesicle preparations where the plasma membrane and cytosolic proteins are enriched in the F3-10K preparation while the mitochondrial and endoplasmic reticulum components are enriched in the F5-10K preparation.

**Figure 7:**
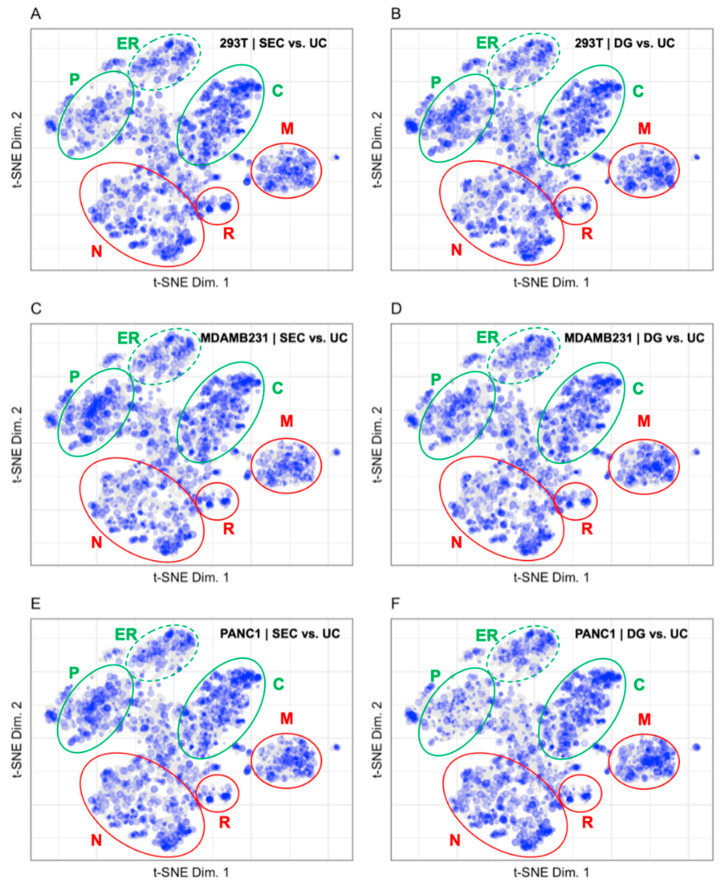
Fold changes for (**A**) SEC vs. UC 293T (1994 proteins), (**B**) DG vs. UC 293T (2174 proteins), (**C**) SEC vs. UC MDAMB231 (2088 proteins), (**D**) DG vs. UC MDAMB231 (2116 proteins), (**E**) SEC vs. UC PANC1 (2047 proteins), and (**F**) DG vs. UC PANC1 (2106 proteins). The plasma membrane (P), cytosol (C), and endoplasmic reticulum (ER) are circled in green, indicating that these regions contain markers of extracellular vesicles. The nucleus (N), ribosomes (R), and mitochondria (M) are circled in red, indicating that these regions contain markers of contaminant proteins in extracellular vesicle preparations.

The fold change in protein abundance for comparisons between exosome preparations by Kugeratski et al. via ultracentrifugation (UC), size exclusion chromatography (SEC), and density gradient ultracentrifugation (DG) were calculated for 3 cell lines with UC as the reference and binned before plotting to the LOPIT coordinates in Figure 7 [14]. The size of each point in Figure 7 corresponds to the binned protein abundance ratio where larger sized points indicate higher fold changes between fractions. Mapping the fold change in protein abundances between preparation methods allows us to visually compare distribution patterns from the different vesicle enrichment methods. The fold change patterns for vesicle preparations from the cell lines 293T, MDAMB231, and PANC1 in Figure 7 demonstrate the differences between SEC and DG using UC as a reference [14]. The difference between SEC and UC is shown in Figure 7 for the cell lines 293T (A), MDAMB231 (C), and PANC1 (E). In all three cell lines, SEC is enriched in all organelles compared to UC. There are 27 and 8 fewer proteins identified in the plasma membrane compartment for the SEC enrichment of MDAMB231 and PANC1 compared to the 293T preparation. The difference between DG and UC is shown in Figure 7 for the cell lines 293T (B), MDAMB231 (C), and PANC1 (F). In all three cell lines, DG is enriched in all organelles compared to UC with the exception of the plasma membrane in the PANC1 enrichment (F). There are 11 and 9 fewer proteins identified in the plasma membrane compartment for the DG enrichment of PANC1 and 293T compared to the MDAMB231 preparation. In the 293T cell line (A and B) there are 103 fewer proteins identified in the unknown compartment, 15 fewer proteins identified in the plasma membrane, 33 fewer proteins identified in the nucleus, and 19 fewer proteins identified in the mitochondria for the SEC enrichment compared to the DG enrichment. In the MDAMB231 cell line (C and D) there is a higher fold change in protein abundance in the plasma membrane and mitochondrial compartments for the SEC enrichment compared to the DG enrichment. In the PANC1 cell line (E and F), there is a higher fold change in abundance of proteins in the plasma membrane and a lower fold change in abundance of proteins in the mitochondrial compartment for the SEC enrichment compared to the DG enrichment.

## 4. Discussion

### 4.1. LOPIT Plots for the Interpretation of Data from a BioID Proximity Labeling Experiment

The patterns that emerge when mapping protein abundance or abundance ratios on LOPIT plots allow us to define proteins of interest. Armed with the knowledge that the primary protein of interest was the plasma membrane protein PCFT and that highly abundant proteins in the W-2 fractions of the GR and PCFT groups are predominantly background proteins, clear differences between the control and test groups could be identified. The pattern of protein abundance in the GR group Elute fraction is similar to the pattern of protein abundance in the GR group W-2 fractions which correspond to background proteins in the experiment.

The patterns that emerge on LOPIT plots of the fold change between the Elute and W-2 fractions allow us to differentiate between background proteins and proteins of interest for each group. Proteins with larger fold changes between the Elute and the W-2 fractions are likely to have true interactions with the bait protein. The W-2 fractions contain background proteins that are likely to be false positives in the Elute fraction. Proteins with a high fold change between the PCFT Elute and W-2 fractions and are localized to the plasma membrane, are likely to be true positive interactors with the folate transporter protein.

Carboxylases are endogenously biotinylated proteins that we use as a positive control when assessing the efficiency of the streptavidin pulldown. The carboxylases are between 2.1 and 83.9 times more abundant in the Elute than the W-2 fractions. The carboxylase ratio between the Elute and W-2 fractions establishes a cut off to increase confidence that the identifying proteins are true positives and are biotinylated.

The utility of LOPIT maps for interpretation of data from proximity labeling experiments is based on the coordinates of proteins determined in cells at rest. Interventions that promote intracellular movement of proteins by diffusion or cyclosis are not accounted for. For the PCFT protein, which is localized to the plasma membrane, such processes might be less important that for the GR protein which is not expected to be localized within the cell [18,22].

### 4.2. Pattern Recognition Is Useful for the Interpretation of Data from Extracellular Vesicle Preparations

Displaying vesicular protein abundance on the LOPIT plot allows patterns from disparate isolation techniques to be recognized [15]. The vesicular proteins from ultracentrifugation, size exclusion chromatography, and polymer-capillary channel hydrophilic interaction chromatography isolation from blood serum have a similar profile when displayed on a LOPIT plot. Most proteins originating from serum-derived exosomes are localized in the plasma membrane and cytosol along with some proteins that reside in the proteasome and lysosome. Serum-derived exosomes display few proteins from the mitochondria or nucleus and the presence of proteins that reside primarily in those organelles could indicate a low degree of enrichment for the vesicles.

The patterns of protein abundance shown in Figure 5 allow us to evaluate the degree of vesicle enrichment in the various preparations. Mapping the MISEV2018 recommended proteins on the LOPIT plot indicates that protein markers of extracellular vesicles are primarily located in coordinates from the plasma membrane, lysosome, ER, and cytosol while contaminant proteins are present in the mitochondria, ribosome, and nucleus [17]. The F3-100K data from Kowal et al. serve as a high-quality benchmark for evaluating exosome preparations [13]. The most abundant proteins in the F3-100K data are present in the plasma membrane, endoplasmic reticulum, and cytosol while lower abundance proteins are present in the mitochondria, nucleus, ribosomes, and proteasome. The highly abundant proteins in F3-100K correlate well with the location of marker proteins from the MISEV2018 recommendation and the lower abundance proteins correlate well with the location of the contaminant proteins from the MISEV2018 recommendation.

The patterns of protein abundance of the proposed core exosome proteins from Kugeratski et al. on the LOPIT plot in Figure 5 are very different from those for the benchmark F3-100K sample from Kowal et al. [13,14]. Many of the proposed core exosome proteins are located in the cytosol, endoplasmic reticulum, and plasma membrane which is in line with the exosome marker proteins in the MISEV2018 recommendation [17]. However, the high densities of proteins located in the mitochondria, ribosomes, and nucleus that are considered to be contaminant proteins in the MISEV2018 recommendation as well as the absence of those same proteins in the F3-100K map suggest that the data are from poorly enriched vesicle preparations.

The patterns of fold change in Figure 6 allow us to differentiate between four vesicular preparation fractions from Kowal et al. [13]. The fractions differed in the first ultracentrifugation cycle where vesicles in a cell culture media were pelleted at 10,000× *g* or 100,000× *g*. The 10,000× *g* pellet contained vesicles larger than 200 nm and the 100,000× *g* pellet contained vesicles between 50 and 150 nm. Vesicle pellets were fractionated by floating in a density gradient where fraction 3 contained exosomes and fraction 5 contained larger vesicles. The fold change plots demonstrate that exosomes have higher protein abundances in the plasma membrane and cytosol with lower protein abundances in the mitochondria and endoplasmic reticulum when compared to larger vesicles.

The patterns of fold change in Figure 7 do not differentiate very well between the UC, SEC, and DG vesicular preparation methods in Kugeratski et al. [14]. UC is used as the benchmark in the comparison of preparation methods for evaluating these data because UC is the most commonly used method to prepare extracellular vesicles [19]. While there is some increase in signal density in the plasma membrane space for the SEC preparations, in general there is no marked difference for either SEC or DG relative to proteins prepared by UC. The uniformity of the protein distribution across all organellar locations may indicate that soluble protein carryover is present in all three preparation methods and is consistent across each of the cell lines evaluated by Kugeratski et al.

## 5. Conclusions

Interpretations of proteome data can be enhanced by comparing patterns of protein localization on the LOPIT map in proximity labeling experiments. Proteins localized in close proximity to the PCFT protein in the plasma membrane region of the map for the Elute fraction are likely to be true positives. Proteins that are localized in compartments different from the plasma membrane and that are enriched to as great an extent as carboxylase proteins are also of interest. Plotting only proteins that are enriched at least 3-fold in the Eluate as compared to the final wash, a ratio established from the carboxylase recoveries, highlights 238 proteins of interest in the PCFT Elute fraction.

The LOPIT maps help in assessing the degree to which exosome preparations are enriched in vesicles. The pattern of protein localization from core exosome proteins proposed in the MISEV2018 are similar to the benchmark vesicle preparation in Kowal et al. because most proteins are localized to the plasma membrane and cytosol in both data sets. Contaminant proteins defined by the MISEV2018 are shown in the Kowal et al. preparation and are expected to be present based on the MISEV2018 recommendation. The patterns presented in the LOPIT map from the core exosome proteins proposed in the MISEV2018 and the benchmark vesicle preparation from Kowal et al. differs from the proteins identified in Kugeratski et al. There is an abundance of nuclear proteins in the Kugeratski et al. preparations which are missing in the LOPIT maps from the MISEV2018 and Kowal et al. data. Further validation of nuclear proteins as exosome-associated proteins would be required in order to incorporate nuclear proteins as exosome marker proteins. The use of LOPIT plots to reevaluate data from public repositories is a feature of the analysis presented. The representations of those data in LOPIT plots changes the interpretation of the previously published work.

LOPIT maps are powerful tools that can be leveraged to aid in the interpretation of proteomics data. They provide a coordinate-directed representation of proteomics data that is freely and easily available to all investigators and, therefore, provides a shared frame of reference. The visual patterns that arise on LOPIT maps are easy to understand and compare. It is not practical, or a good use of resources, for every investigator to generate LOPIT type plots from their own cell culture or tissue samples. The exception to this is any condition, such as a disease state, that causes an abnormal organellar distribution of proteins. In such instances, using coordinates from normal cells would not address questions of disruptions in protein distribution. Future work to increase the depth of coverage from the current 5020 proteins to include more of the 10,000 or so proteins expected to be in any cell and to increase resolution of organellar boundaries in LOPIT maps will increase the utility of the map for the interpretation of proteomic data.

## Figures and Tables

**Table 1:** The total number of proteins identified for and contained in the LOPIT plot is shown for each fraction and group.

Fraction	Total Protein IDs	Total LOPIT Proteins	GR Protein IDs	GR LOPIT Proteins (%)	PCFT Protein IDs	PCFT LOPIT Proteins (%)
**W-1**	2330	1986	2297	85.42%	2284	85.42%
**W-2**	3249	2403	2375	79.83%	2222	79.48%
**Elute**	1220	887	781	75.93%	687	72.78%

**Table 2:** Proteins recommended for the evaluation of extracellular vesicle sample preparation.

Category	Proteins	Mapped Proteins	Proteins Included	LOPIT Color
1	110	38	Proteins associated to the plasma membrane and/or endosomes	Green
2	55	35	Proteins recovered in the cytosol of extracellular vesicles	Green
3	195	157	Non-extracellular vesicle co-isolated structures	Red
4	41	14	Markers for extracellular vesicle subtypes	Orange
5	160	28	Functional component of EVs	Green

**Table 3:** The number of proteins mapped to each LOPIT assignment is shown for the MISEV, Kowal et al., and Kurgeratski et al. data.

Assignment	MISEV 2018 Mapped Proteins	Kowal et. al. F3-100K Mapped Proteins	Kugeratski et al. Core Exosome Mapped Proteins	Kugeratski et al. Cell Exosome Mapped Proteins
Cytosol	2	94	82	109
Endoplasmic reticulum	6	79	42	108
Golgi	0	4	2	15
Lysosome	2	20	2	27
Mitochondria	45	90	78	240
Nucleus	9	68	112	429
Nucleus-chromatin	8	3	10	35
Peroxisome	0	6	3	9
Plasma membrane	29	127	34	128
Proteasome	0	32	27	29
Ribosome 40S	30	29	23	31
Ribosome 60S	47	39	39	44
Unknown	94	995	454	1349

## Data Availability

The mass spectrometry proteomics data have been deposited to the ProteomeXchange Consortium via the PRIDE partner repository with the dataset identifier PXD032297 and 10.6019/PXD032297.

## Supplementary Materials

The following are available online at https://www.mdpi.com/article/10.3390/proteomes10020018/s1, Table S1: Quantitative data for all proteins identified in the proximity labeling experiment.
