# Supplementary material for: Pattern Analysis of Organellar Maps for Interpretation of Proteomic Data
Source: Proteomes. 2022 May 23;10(2):18. doi: 10.3390/proteomes10020018 (PMC9149908; doi:10.3390/proteomes10020018)
Supplement: Supplementary file 1 [file proteomes-10-00018-s001.zip › proteomes-1672250-supplementary.pdf]

## LOPIT Assignments

| **Row Number** | Accession | Description | assignment | tSNE Dimension.1 | tSNE Dimension.2 | inElute | inWash01 | inWash02 |
|---|---|---|---|---|---|---|---|---|
| **1** | P10412 | Histone H1.4 | NUCLEUS-CHROMATIN | -22.23 | -12.53 | TRUE | TRUE | TRUE |
| **2** | P16403 | Histone H1.2 | NUCLEUS-CHROMATIN | -22.26 | -12.63 | FALSE | TRUE | TRUE |
| **3** | P62861 | 40S ribosomal protein S30 | RIBOSOME 40S | 12.28 | -23.48 | TRUE | TRUE | TRUE |
| **4** | P08238 | Heat shock protein HSP 90-beta | CYTOSOL | 20.67 | 22.31 | TRUE | TRUE | TRUE |
| **5** | Q9NX58 | Cell growth-regulating nucleolar pro | NUCLEUS | 2.41 | -28.07 | TRUE | TRUE | TRUE |
| **6** | Q13428 | Treacle protein | NUCLEUS | -21.21 | -30.08 | TRUE | TRUE | TRUE |
| **7** | P68104 | Elongation factor 1-alpha 1 | unknown | -4.40 | -10.29 | TRUE | TRUE | TRUE |
| **8** | P62979 | Ubiquitin-40S ribosomal protein S27 | unknown | 17.51 | -18.28 | TRUE | TRUE | TRUE |
| **9** | P07305 | Histone H1.0 | NUCLEUS-CHROMATIN | -22.39 | -12.90 | TRUE | TRUE | TRUE |
| **10** | P13639 | Elongation factor 2 | unknown | 14.67 | -21.12 | TRUE | TRUE | TRUE |
| **11** | P14618 | Pyruvate kinase PKM | unknown | 10.11 | -2.34 | TRUE | TRUE | TRUE |
| **12** | P62750 | 60S ribosomal protein L23a | RIBOSOME 60S | 16.20 | -22.26 | TRUE | TRUE | TRUE |
| **13** | P07437 | Tubulin beta chain | unknown | 17.51 | 12.63 | TRUE | TRUE | TRUE |
| **14** | P00338 | L-lactate dehydrogenase A chain | unknown | 17.59 | 7.15 | TRUE | TRUE | TRUE |
| **15** | P07900 | Heat shock protein HSP 90-alpha | unknown | 23.54 | 23.08 | TRUE | TRUE | TRUE |
| **16** | P04406 | Glyceraldehyde-3-phosphate dehy | unknown | 10.91 | -2.32 | FALSE | TRUE | TRUE |
| **17** | P11142 | Heat shock cognate 71 kDa protein | unknown | 13.41 | 11.74 | TRUE | TRUE | TRUE |
| **18** | P62805 | Histone H4 | NUCLEUS-CHROMATIN | -22.92 | -13.63 | TRUE | TRUE | TRUE |
| **19** | P68371 | Tubulin beta-4B chain | CYTOSOL | 20.99 | 18.38 | TRUE | TRUE | TRUE |
| **20** | O00567 | Nucleolar protein 56 | NUCLEUS | -3.62 | -40.72 | TRUE | TRUE | TRUE |
| **21** | P49327 | Fatty acid synthase | unknown | 19.38 | 9.92 | TRUE | TRUE | TRUE |
| **22** | P08670 | Vimentin | NUCLEUS | -26.11 | -26.79 | TRUE | TRUE | TRUE |
| **23** | P31327 | Carbamoyl-phosphate synthase [ar | MITOCHONDRIA | 36.41 | -8.41 | FALSE | TRUE | TRUE |
| **24** | P05141 | ADP/ATP translocase 2 | MITOCHONDRIA | 32.33 | -13.30 | TRUE | TRUE | TRUE |
| **25** | Q02878 | 60S ribosomal protein L6 | RIBOSOME 60S | 16.06 | -21.88 | TRUE | TRUE | TRUE |
| **26** | Q562R1 | Beta-actin-like protein 2 | unknown | 3.89 | 8.32 | FALSE | TRUE | TRUE |
| **27** | Q1ED39 | Lysine-rich nucleolar protein 1 | NUCLEUS | 0.46 | -28.34 | TRUE | TRUE | TRUE |
| **28** | P07195 | L-lactate dehydrogenase B chain | CYTOSOL | 22.23 | 21.65 | FALSE | TRUE | TRUE |
| **29** | P07737 | Profilin-1 | CYTOSOL | 22.56 | 22.08 | FALSE | TRUE | TRUE |
| **30** | P62937 | Peptidyl-prolyl cis-trans isomerase | unknown | 15.45 | 10.96 | TRUE | TRUE | TRUE |
| **31** | P60842 | Eukaryotic initiation factor 4A-I | unknown | 13.49 | -1.19 | TRUE | TRUE | TRUE |
| **32** | P18583 | Protein SON | NUCLEUS | -3.66 | -34.30 | TRUE | TRUE | TRUE |
| **33** | P06733 | Alpha-enolase | CYTOSOL | 22.75 | 19.08 | FALSE | TRUE | TRUE |
| **34** | P47914 | 60S ribosomal protein L29 | RIBOSOME 60S | 16.99 | -20.62 | TRUE | TRUE | TRUE |

| | | | | | | | | |
|---|---|---|---|---|---|---|---|---|
| **35** | P62241 | 40S ribosomal protein S8 | RIBOSOME 40S | 11.84 | -22.95 | TRUE | TRUE | TRUE |
| **36** | P11021 | Endoplasmic reticulum chaperone E | ER | -1.49 | 38.57 | TRUE | TRUE | TRUE |
| **37** | P22626 | Heterogeneous nuclear ribonucleop | NUCLEUS | -13.47 | -36.47 | TRUE | TRUE | TRUE |
| **38** | P39023 | 60S ribosomal protein L3 | RIBOSOME 60S | 16.15 | -21.46 | TRUE | TRUE | TRUE |
| **39** | P62424 | 60S ribosomal protein L7a | RIBOSOME 60S | 17.04 | -21.59 | TRUE | TRUE | TRUE |
| **40** | Q06830 | Peroxiredoxin-1 | NUCLEUS | -2.16 | -12.94 | TRUE | TRUE | TRUE |
| **41** | P11387 | DNA topoisomerase 1 | NUCLEUS-CHROMATIN | -22.28 | -15.61 | FALSE | TRUE | TRUE |
| **42** | Q08211 | ATP-dependent RNA helicase A | NUCLEUS | -6.14 | -31.92 | TRUE | TRUE | TRUE |
| **43** | P12235 | ADP/ATP translocase 1 | unknown | 29.48 | -15.75 | FALSE | TRUE | TRUE |
| **44** | P63244 | Receptor of activated protein C kina | RIBOSOME 40S | 10.96 | -22.66 | FALSE | TRUE | TRUE |
| **45** | Q969Q0 | 60S ribosomal protein L36a-like | RIBOSOME 60S | 17.26 | -22.14 | TRUE | TRUE | TRUE |
| **46** | P62826 | GTP-binding nuclear protein Ran | unknown | 23.38 | 12.48 | TRUE | TRUE | TRUE |
| **47** | Q6P2Q9 | Pre-mRNA-processing-splicing facto | NUCLEUS | 1.57 | -37.78 | TRUE | TRUE | TRUE |
| **48** | O60841 | Eukaryotic translation initiation fact | NUCLEUS | -8.21 | -15.56 | TRUE | TRUE | TRUE |
| **49** | Q9NR30 | Nucleolar RNA helicase 2 | NUCLEUS | -1.24 | -35.52 | TRUE | TRUE | TRUE |
| **50** | Q9BRT6 | Protein LLP homolog | NUCLEUS | 5.86 | -26.53 | TRUE | TRUE | TRUE |
| **51** | Q9BUF5 | Tubulin beta-6 chain | unknown | 15.81 | 9.71 | FALSE | TRUE | TRUE |
| **52** | P10809 | 60 kDa heat shock protein, mitocho | MITOCHONDRIA | 36.01 | -6.60 | TRUE | TRUE | TRUE |
| **53** | P23396 | 40S ribosomal protein S3 | RIBOSOME 40S | 11.14 | -22.60 | FALSE | TRUE | TRUE |
| **54** | P26641 | Elongation factor 1-gamma | NUCLEUS | -8.90 | -14.58 | FALSE | TRUE | TRUE |
| **55** | O14617 | AP-3 complex subunit delta-1 | unknown | -13.11 | 3.47 | FALSE | TRUE | TRUE |
| **56** | P78527 | DNA-dependent protein kinase cata | unknown | -14.76 | -16.04 | TRUE | TRUE | TRUE |
| **57** | Q00839 | Heterogeneous nuclear ribonucleop | NUCLEUS | -6.74 | -31.74 | TRUE | TRUE | TRUE |
| **58** | Q00839 | Heterogeneous nuclear ribonucleop | NUCLEUS | -6.81 | -31.81 | TRUE | TRUE | TRUE |
| **59** | P62753 | 40S ribosomal protein S6 | RIBOSOME 40S | 11.39 | -22.69 | FALSE | TRUE | TRUE |
| **60** | P21333 | Filamin-A | unknown | -8.02 | -4.54 | TRUE | TRUE | TRUE |
| **61** | Q14240 | Eukaryotic initiation factor 4A-II | unknown | 4.06 | 6.23 | FALSE | TRUE | TRUE |
| **62** | P62829 | 60S ribosomal protein L23 | RIBOSOME 60S | 15.98 | -22.41 | TRUE | TRUE | TRUE |
| **63** | Q07020 | 60S ribosomal protein L18 | RIBOSOME 60S | 17.48 | -21.04 | FALSE | TRUE | TRUE |
| **64** | Q12931 | Heat shock protein 75 kDa, mitocho | MITOCHONDRIA | 38.00 | -8.37 | FALSE | TRUE | TRUE |
| **65** | P07355 | Annexin A2 | unknown | -17.10 | 13.74 | TRUE | TRUE | TRUE |
| **66** | Q09666 | Neuroblast differentiation-associate | unknown | -10.35 | 23.31 | TRUE | TRUE | TRUE |
| **67** | P23526 | Adenosylhomocysteinase | CYTOSOL | 22.32 | 22.66 | TRUE | TRUE | TRUE |
| **68** | P30041 | Peroxiredoxin-6 | unknown | 24.55 | 24.23 | TRUE | TRUE | TRUE |
| **69** | O60832 | H/ACA ribonucleoprotein complex s | NUCLEUS | -4.14 | -39.00 | TRUE | TRUE | TRUE |
| **70** | P06748 | Nucleophosmin | unknown | 6.89 | -13.95 | TRUE | TRUE | TRUE |
| **71** | P23284 | Peptidyl-prolyl cis-trans isomerase I | ER | 1.79 | 36.67 | TRUE | TRUE | TRUE |
| **72** | P62917 | 60S ribosomal protein L8 | RIBOSOME 60S | 17.02 | -21.27 | TRUE | TRUE | TRUE |

| | | | | | | | | | |
|---|---|---|---|---|---|---|---|---|---|
| **73** | P62280 | 40S ribosomal protein S11 | RIBOSOME 40S | 11.41 | -22.61 | FALSE | TRUE | TRUE |
| **74** | Q92522 | Histone H1x | NUCLEUS-CHROMATIN | -14.88 | -19.84 | TRUE | TRUE | TRUE |
| **75** | P83731 | 60S ribosomal protein L24 | RIBOSOME 60S | 17.38 | -21.67 | TRUE | TRUE | TRUE |
| **76** | P62701 | 40S ribosomal protein S4, X isoform | RIBOSOME 40S | 11.23 | -22.87 | FALSE | TRUE | TRUE |
| **77** | P18124 | 60S ribosomal protein L7 | RIBOSOME 60S | 16.01 | -21.95 | FALSE | TRUE | TRUE |
| **78** | Q8NE71 | ATP-binding cassette sub-family F r | unknown | -3.94 | -10.81 | TRUE | TRUE | TRUE |
| **79** | P15880 | 40S ribosomal protein S2 | RIBOSOME 40S | 11.08 | -22.58 | TRUE | TRUE | TRUE |
| **80** | O76021 | Ribosomal L1 domain-containing pr | NUCLEUS | 0.69 | -38.72 | TRUE | TRUE | TRUE |
| **81** | P11498 | Pyruvate carboxylase, mitochondria | MITOCHONDRIA | 36.81 | -6.11 | TRUE | TRUE | TRUE |
| **82** | O00571 | ATP-dependent RNA helicase DDX3 | unknown | 3.93 | -26.10 | TRUE | TRUE | TRUE |
| **83** | P62263 | 40S ribosomal protein S14 | RIBOSOME 40S | 11.79 | -22.91 | TRUE | TRUE | TRUE |
| **84** | P36578 | 60S ribosomal protein L4 | RIBOSOME 60S | 15.64 | -21.82 | TRUE | TRUE | TRUE |
| **85** | P61313 | 60S ribosomal protein L15 | RIBOSOME 60S | 16.41 | -21.24 | FALSE | TRUE | TRUE |
| **86** | Q02543 | 60S ribosomal protein L18a | RIBOSOME 60S | 16.65 | -21.31 | FALSE | TRUE | TRUE |
| **87** | P46777 | 60S ribosomal protein L5 | RIBOSOME 60S | 16.70 | -21.41 | FALSE | TRUE | TRUE |
| **88** | P17844 | Probable ATP-dependent RNA helic | NUCLEUS | 1.86 | -32.46 | TRUE | TRUE | TRUE |
| **89** | P11388 | DNA topoisomerase 2-alpha | NUCLEUS-CHROMATIN | -21.59 | -13.79 | TRUE | TRUE | TRUE |
| **90** | P46776 | 60S ribosomal protein L27a | RIBOSOME 60S | 15.37 | -21.93 | TRUE | TRUE | TRUE |
| **91** | Q00610 | Clathrin heavy chain 1 | unknown | -10.03 | 13.72 | FALSE | TRUE | TRUE |
| **92** | P06576 | ATP synthase subunit beta, mitocho | MITOCHONDRIA | 35.78 | -7.03 | FALSE | TRUE | TRUE |
| **93** | P14625 | Endoplasmin | ER | -1.05 | 39.01 | FALSE | TRUE | TRUE |
| **94** | Q9BVP2 | Guanine nucleotide-binding protein | NUCLEUS | 2.10 | -35.18 | TRUE | TRUE | TRUE |
| **95** | Q14204 | Cytoplasmic dynein 1 heavy chain 1 | unknown | -11.35 | -7.41 | FALSE | TRUE | TRUE |
| **96** | Q7KZF4 | Staphylococcal nuclease domain-co | RIBOSOME 60S | 16.90 | -23.47 | FALSE | TRUE | TRUE |
| **97** | P61353 | 60S ribosomal protein L27 | RIBOSOME 60S | 17.62 | -22.17 | FALSE | TRUE | TRUE |
| **98** | P38646 | Stress-70 protein, mitochondrial | MITOCHONDRIA | 37.28 | -4.73 | FALSE | TRUE | TRUE |
| **99** | Q9UQ80 | Proliferation-associated protein 2G4 | unknown | 11.54 | -20.80 | TRUE | TRUE | TRUE |
| **100** | P17987 | T-complex protein 1 subunit alpha | unknown | 15.73 | 9.83 | FALSE | TRUE | TRUE |
| **101** | P00558 | Phosphoglycerate kinase 1 | CYTOSOL | 22.88 | 22.95 | TRUE | TRUE | TRUE |
| **102** | P35579 | Myosin-9 | unknown | 24.35 | -5.17 | TRUE | TRUE | TRUE |
| **103** | P60174 | Triosephosphate isomerase | CYTOSOL | 21.56 | 22.84 | FALSE | TRUE | TRUE |
| **104** | Q14697 | Neutral alpha-glucosidase AB | unknown | 0.93 | 40.29 | FALSE | TRUE | TRUE |
| **105** | P25705 | ATP synthase subunit alpha, mitoch | MITOCHONDRIA | 35.86 | -8.72 | FALSE | TRUE | TRUE |
| **106** | P19338 | Nucleolin | unknown | 23.34 | 12.20 | TRUE | TRUE | TRUE |
| **107** | P55060 | Exportin-2 | unknown | 27.66 | 22.33 | TRUE | TRUE | TRUE |
| **108** | P14866 | Heterogeneous nuclear ribonucleop | NUCLEUS | -13.51 | -36.53 | TRUE | TRUE | TRUE |
| **109** | P62847 | 40S ribosomal protein S24 | RIBOSOME 40S | 11.04 | -22.97 | FALSE | TRUE | TRUE |
| **110** | P11940 | Polyadenylate-binding protein 1 | unknown | 3.93 | -22.17 | FALSE | TRUE | TRUE |

| | | | | | | | | |
|---|---|---|---|---|---|---|---|---|
| **111** | P22392 | Nucleoside diphosphate kinase B | unknown | 19.69 | 9.81 | FALSE | TRUE | TRUE |
| **112** | P63241 | Eukaryotic translation initiation fact | unknown | 2.34 | -13.49 | TRUE | TRUE | TRUE |
| **113** | P30050 | 60S ribosomal protein L12 | RIBOSOME 60S | 16.23 | -21.51 | FALSE | TRUE | TRUE |
| **114** | P38919 | Eukaryotic initiation factor 4A-III | NUCLEUS | -3.27 | -40.69 | TRUE | TRUE | TRUE |
| **115** | P12956 | X-ray repair cross-complementing p | NUCLEUS | -4.81 | -16.05 | FALSE | TRUE | TRUE |
| **116** | P49411 | Elongation factor Tu, mitochondrial | MITOCHONDRIA | 39.58 | -7.73 | FALSE | TRUE | TRUE |
| **117** | Q9H0A0 | RNA cytidine acetyltransferase | NUCLEUS | -1.77 | -36.99 | TRUE | TRUE | TRUE |
| **118** | Q15365 | Poly(rC)-binding protein 1 | unknown | 13.27 | 1.63 | TRUE | TRUE | TRUE |
| **119** | P26639 | Threonine--tRNA ligase, cytoplasmi | unknown | 16.42 | 5.10 | TRUE | TRUE | TRUE |
| **120** | P31943 | Heterogeneous nuclear ribonucleop | NUCLEUS | -19.22 | -32.48 | TRUE | TRUE | TRUE |
| **121** | P63104 | 14-3-3 protein zeta/delta | unknown | 12.40 | 16.27 | FALSE | TRUE | TRUE |
| **122** | P42677 | 40S ribosomal protein S27 | RIBOSOME 40S | 10.56 | -23.47 | TRUE | TRUE | TRUE |
| **123** | P46781 | 40S ribosomal protein S9 | RIBOSOME 40S | 11.82 | -22.76 | FALSE | TRUE | TRUE |
| **124** | P41091 | Eukaryotic translation initiation fact | NUCLEUS | -3.17 | -13.89 | TRUE | TRUE | TRUE |
| **125** | P02786 | Transferrin receptor protein 1 | unknown | -36.28 | 15.05 | TRUE | TRUE | TRUE |
| **126** | P29401 | Transketolase | unknown | 8.23 | -4.49 | TRUE | TRUE | TRUE |
| **127** | P51991 | Heterogeneous nuclear ribonucleop | NUCLEUS | -13.12 | -36.41 | TRUE | TRUE | TRUE |
| **128** | Q15149 | Plectin | NUCLEUS | -11.97 | -18.97 | TRUE | TRUE | TRUE |
| **129** | Q15149 | Plectin | unknown | 2.19 | -26.46 | TRUE | TRUE | TRUE |
| **130** | Q9Y2X3 | Nucleolar protein 58 | NUCLEUS | -3.37 | -40.99 | FALSE | TRUE | TRUE |
| **131** | P15121 | Aldose reductase | CYTOSOL | 20.89 | 20.79 | FALSE | TRUE | TRUE |
| **132** | Q00325 | Phosphate carrier protein, mitochor | MITOCHONDRIA | 36.91 | -12.21 | FALSE | TRUE | TRUE |
| **133** | P62888 | 60S ribosomal protein L30 | RIBOSOME 60S | 16.79 | -21.50 | FALSE | TRUE | TRUE |
| **134** | Q99623 | Prohibitin-2 | MITOCHONDRIA | 30.33 | -6.19 | FALSE | TRUE | TRUE |
| **135** | P09874 | Poly [ADP-ribose] polymerase 1 | NUCLEUS-CHROMATIN | -22.18 | -15.66 | FALSE | TRUE | TRUE |
| **136** | P50991 | T-complex protein 1 subunit delta | unknown | 10.92 | -1.40 | FALSE | TRUE | TRUE |
| **137** | P46778 | 60S ribosomal protein L21 | RIBOSOME 60S | 16.47 | -22.22 | TRUE | TRUE | TRUE |
| **138** | P31946 | 14-3-3 protein beta/alpha | unknown | 12.67 | 16.10 | FALSE | TRUE | TRUE |
| **139** | P84103 | Serine/arginine-rich splicing factor 3 | NUCLEUS | -5.54 | -31.21 | FALSE | TRUE | TRUE |
| **140** | P61254 | 60S ribosomal protein L26 | RIBOSOME 60S | 16.60 | -22.74 | TRUE | TRUE | TRUE |
| **141** | P50395 | Rab GDP dissociation inhibitor beta | unknown | 14.71 | 23.14 | FALSE | TRUE | TRUE |
| **142** | P26373 | 60S ribosomal protein L13 | RIBOSOME 60S | 17.39 | -21.61 | TRUE | TRUE | TRUE |
| **143** | Q15029 | 116 kDa U5 small nuclear ribonucle | NUCLEUS | 1.76 | -37.42 | FALSE | TRUE | TRUE |
| **144** | Q99832 | T-complex protein 1 subunit eta | unknown | 10.62 | -1.78 | FALSE | TRUE | TRUE |
| **145** | P62258 | 14-3-3 protein epsilon | unknown | 11.54 | 14.69 | FALSE | TRUE | TRUE |
| **146** | Q01813 | ATP-dependent 6-phosphofructokir | unknown | 8.51 | -3.07 | FALSE | TRUE | TRUE |
| **147** | P26038 | Moesin | unknown | -13.75 | 13.59 | TRUE | TRUE | TRUE |
| **148** | O75643 | U5 small nuclear ribonucleoprotein | NUCLEUS | 1.39 | -37.63 | TRUE | TRUE | TRUE |

| | | | | | | | | |
|---|---|---|---|---|---|---|---|---|
| **149** | P50990 | T-complex protein 1 subunit theta | CYTOSOL | 16.88 | 17.72 | TRUE | TRUE | TRUE |
| **150** | P02545 | Prelamin-A/C | unknown | 23.57 | -12.75 | TRUE | TRUE | TRUE |
| **151** | P48643 | T-complex protein 1 subunit epsilon | unknown | 16.64 | 17.32 | TRUE | TRUE | TRUE |
| **152** | P04075 | Fructose-bisphosphate aldolase A | unknown | 8.94 | 4.39 | FALSE | TRUE | TRUE |
| **153** | P61247 | 40S ribosomal protein S3a | RIBOSOME 40S | 11.05 | -22.91 | TRUE | TRUE | TRUE |
| **154** | P07814 | Bifunctional glutamate/proline--tRN | unknown | 5.75 | -3.24 | TRUE | TRUE | TRUE |
| **155** | P49368 | T-complex protein 1 subunit gamma | unknown | 10.47 | 1.37 | FALSE | TRUE | TRUE |
| **156** | Q15233 | Non-POU domain-containing octam | NUCLEUS | 2.04 | -33.31 | TRUE | TRUE | TRUE |
| **157** | P15311 | Ezrin | unknown | -13.75 | 13.55 | TRUE | TRUE | TRUE |
| **158** | P62854 | 40S ribosomal protein S26 | RIBOSOME 40S | 12.72 | -19.11 | TRUE | TRUE | TRUE |
| **159** | P46087 | Probable 28S rRNA (cytosine(4447) | NUCLEUS | -0.11 | -35.62 | TRUE | TRUE | TRUE |
| **160** | P27348 | 14-3-3 protein theta | unknown | 13.05 | 15.97 | TRUE | TRUE | TRUE |
| **161** | P22087 | rRNA 2'-O-methyltransferase fibrilla | NUCLEUS | -0.97 | -38.80 | FALSE | TRUE | TRUE |
| **162** | P62081 | 40S ribosomal protein S7 | RIBOSOME 40S | 11.33 | -23.11 | FALSE | TRUE | TRUE |
| **163** | P62249 | 40S ribosomal protein S16 | RIBOSOME 40S | 11.30 | -22.62 | FALSE | TRUE | TRUE |
| **164** | Q99714 | 3-hydroxyacyl-CoA dehydrogenase | MITOCHONDRIA | 37.05 | -4.17 | FALSE | TRUE | TRUE |
| **165** | P33993 | DNA replication licensing factor MC | unknown | -15.45 | -31.51 | FALSE | TRUE | TRUE |
| **166** | P18669 | Phosphoglycerate mutase 1 | CYTOSOL | 23.14 | 19.88 | FALSE | TRUE | TRUE |
| **167** | Q86UE4 | Protein LYRIC | unknown | 5.07 | 38.63 | TRUE | TRUE | TRUE |
| **168** | P20908 | Collagen alpha-1(V) chain | unknown | -14.54 | 20.25 | FALSE | TRUE | TRUE |
| **169** | P35268 | 60S ribosomal protein L22 | RIBOSOME 60S | 17.38 | -20.02 | TRUE | TRUE | TRUE |
| **170** | P07910 | Heterogeneous nuclear ribonucleop | NUCLEUS | -13.13 | -36.32 | TRUE | TRUE | TRUE |
| **171** | Q9NZM1 | Myoferlin | unknown | -33.67 | 10.04 | TRUE | TRUE | TRUE |
| **172** | Q14684 | Ribosomal RNA processing protein 1 | NUCLEUS | -0.75 | -35.25 | TRUE | TRUE | TRUE |
| **173** | P21796 | Voltage-dependent anion-selective | unknown | 28.29 | -6.28 | FALSE | TRUE | TRUE |
| **174** | P60866 | 40S ribosomal protein S20 | RIBOSOME 40S | 11.44 | -22.84 | FALSE | TRUE | TRUE |
| **175** | P34932 | Heat shock 70 kDa protein 4 | CYTOSOL | 23.36 | 23.40 | FALSE | TRUE | TRUE |
| **176** | P05387 | 60S acidic ribosomal protein P2 | RIBOSOME 60S | 15.95 | -21.03 | FALSE | TRUE | TRUE |
| **177** | P23528 | Cofilin-1 | unknown | 3.47 | 9.47 | TRUE | TRUE | TRUE |
| **178** | P62913 | 60S ribosomal protein L11 | RIBOSOME 60S | 16.02 | -21.63 | FALSE | TRUE | TRUE |
| **179** | P61604 | 10 kDa heat shock protein, mitocho | MITOCHONDRIA | 32.44 | -6.91 | FALSE | TRUE | TRUE |
| **180** | Q9UQ35 | Serine/arginine repetitive matrix pr | NUCLEUS | -10.41 | -29.80 | FALSE | TRUE | TRUE |
| **181** | Q7L014 | Probable ATP-dependent RNA helic | NUCLEUS | 4.29 | -31.23 | TRUE | TRUE | TRUE |
| **182** | P32119 | Peroxiredoxin-2 | unknown | 10.36 | 1.08 | TRUE | TRUE | TRUE |
| **183** | Q99873 | Protein arginine N-methyltransfera | unknown | 26.35 | 21.97 | FALSE | TRUE | TRUE |
| **184** | P40429 | 60S ribosomal protein L13a | RIBOSOME 60S | 16.81 | -21.55 | FALSE | TRUE | TRUE |
| **185** | Q9HB71 | Calcyclin-binding protein | unknown | 9.40 | -0.92 | FALSE | TRUE | TRUE |
| **186** | P62244 | 40S ribosomal protein S15a | RIBOSOME 40S | 11.50 | -22.87 | FALSE | TRUE | TRUE |

| | | | | | | | | |
|---|---|---|---|---|---|---|---|---|
| **187** | P33992 | DNA replication licensing factor MC | NUCLEUS | -3.74 | -16.51 | FALSE | TRUE | TRUE |
| **188** | P62906 | 60S ribosomal protein L10a | RIBOSOME 60S | 16.14 | -21.65 | FALSE | TRUE | TRUE |
| **189** | P32969 | 60S ribosomal protein L9 | RIBOSOME 60S | 16.79 | -21.46 | FALSE | TRUE | TRUE |
| **190** | P40227 | T-complex protein 1 subunit zeta | unknown | 9.47 | -0.06 | TRUE | TRUE | TRUE |
| **191** | Q16629 | Serine/arginine-rich splicing factor 7 | NUCLEUS | 3.97 | -26.74 | FALSE | TRUE | TRUE |
| **192** | P38159 | RNA-binding motif protein, X chrom | NUCLEUS | 1.59 | -35.43 | FALSE | TRUE | TRUE |
| **193** | P55072 | Transitional endoplasmic reticulum | unknown | 2.96 | 11.63 | TRUE | TRUE | TRUE |
| **194** | P61981 | 14-3-3 protein gamma | unknown | 10.14 | 13.95 | FALSE | TRUE | TRUE |
| **195** | P24534 | Elongation factor 1-beta | NUCLEUS | 4.01 | -6.42 | TRUE | TRUE | TRUE |
| **196** | O43707 | Alpha-actinin-4 | unknown | 4.03 | 9.41 | FALSE | TRUE | TRUE |
| **197** | P55795 | Heterogeneous nuclear ribonucleop | NUCLEUS | -19.30 | -32.48 | FALSE | TRUE | TRUE |
| **198** | P04843 | Dolichyl-diphosphooligosaccharide- | ER | 2.60 | 34.84 | FALSE | TRUE | TRUE |
| **199** | P37802 | Transgelin-2 | unknown | 11.19 | 13.24 | TRUE | TRUE | TRUE |
| **200** | Q07021 | Complement component 1 Q subco | MITOCHONDRIA | 35.06 | -7.62 | FALSE | TRUE | TRUE |
| **201** | P62266 | 40S ribosomal protein S23 | RIBOSOME 40S | 11.46 | -22.92 | FALSE | TRUE | TRUE |
| **202** | P27797 | Calreticulin | ER | -0.75 | 39.15 | FALSE | TRUE | TRUE |
| **203** | P62820 | Ras-related protein Rab-1A | PM | -28.73 | 13.58 | FALSE | TRUE | TRUE |
| **204** | O75400 | Pre-mRNA-processing factor 40 hor | NUCLEUS | 3.71 | -35.99 | TRUE | TRUE | TRUE |
| **205** | Q07955 | Serine/arginine-rich splicing factor 1 | unknown | -16.09 | -31.46 | FALSE | TRUE | TRUE |
| **206** | Q9Y383 | Putative RNA-binding protein Luc7-l | unknown | 7.13 | -24.56 | TRUE | TRUE | TRUE |
| **207** | Q13310 | Polyadenylate-binding protein 4 | unknown | 3.86 | -22.06 | FALSE | TRUE | TRUE |
| **208** | P05023 | Sodium/potassium-transporting AT | PM | -22.77 | 16.68 | TRUE | TRUE | TRUE |
| **209** | Q13838 | Spliceosome RNA helicase DDX39B | NUCLEUS | -2.89 | -41.61 | FALSE | TRUE | TRUE |
| **210** | P08865 | 40S ribosomal protein SA | RIBOSOME 40S | 11.69 | -22.19 | FALSE | TRUE | TRUE |
| **211** | P49207 | 60S ribosomal protein L34 | RIBOSOME 60S | 17.93 | -21.15 | FALSE | TRUE | TRUE |
| **212** | P25398 | 40S ribosomal protein S12 | RIBOSOME 40S | 11.45 | -22.22 | FALSE | TRUE | TRUE |
| **213** | Q9H0U4 | Ras-related protein Rab-1B | unknown | -18.21 | 20.49 | FALSE | TRUE | FALSE |
| **214** | P62899 | 60S ribosomal protein L31 | RIBOSOME 60S | 17.48 | -22.29 | FALSE | TRUE | TRUE |
| **215** | O00148 | ATP-dependent RNA helicase DDX3 | NUCLEUS | -11.40 | -25.94 | FALSE | TRUE | TRUE |
| **216** | P50914 | 60S ribosomal protein L14 | RIBOSOME 60S | 16.60 | -21.49 | TRUE | TRUE | TRUE |
| **217** | P25205 | DNA replication licensing factor MC | NUCLEUS | -14.93 | -28.96 | FALSE | TRUE | TRUE |
| **218** | Q9BU76 | Multiple myeloma tumor-associate | unknown | 2.23 | -26.61 | TRUE | TRUE | TRUE |
| **219** | P05388 | 60S acidic ribosomal protein P0 | RIBOSOME 60S | 17.12 | -21.36 | FALSE | TRUE | TRUE |
| **220** | P13010 | X-ray repair cross-complementing p | NUCLEUS | -4.85 | -16.06 | TRUE | TRUE | TRUE |
| **221** | P27635 | 60S ribosomal protein L10 | RIBOSOME 60S | 16.94 | -21.08 | FALSE | TRUE | TRUE |
| **222** | P27824 | Calnexin | ER | -6.05 | 31.91 | TRUE | TRUE | TRUE |
| **223** | Q9UG63 | ATP-binding cassette sub-family F r | unknown | 10.10 | -20.95 | FALSE | TRUE | TRUE |
| **224** | P16615 | Sarcoplasmic/endoplasmic reticulu | ER | -6.24 | 33.40 | FALSE | TRUE | TRUE |

| | | | | | | | | |
|---|---|---|---|---|---|---|---|---|
| **225** | P78371 | T-complex protein 1 subunit beta | unknown | 16.02 | 10.50 | FALSE | TRUE | TRUE |
| **226** | P40939 | Trifunctional enzyme subunit alpha | MITOCHONDRIA | 41.70 | -6.16 | FALSE | TRUE | TRUE |
| **227** | P35241 | Radixin | unknown | -13.18 | 13.59 | TRUE | TRUE | TRUE |
| **228** | Q14103 | Heterogeneous nuclear ribonucleop | NUCLEUS | -19.81 | -32.67 | TRUE | TRUE | TRUE |
| **229** | P51149 | Ras-related protein Rab-7a | unknown | -25.19 | 18.08 | FALSE | TRUE | TRUE |
| **230** | Q02539 | Histone H1.1 | NUCLEUS-CHROMATIN | -22.35 | -12.47 | FALSE | FALSE | TRUE |
| **231** | P13797 | Plastin-3 | unknown | 9.51 | 13.21 | TRUE | TRUE | TRUE |
| **232** | P53621 | Coatomer subunit alpha | unknown | 6.45 | 3.59 | FALSE | TRUE | TRUE |
| **233** | P06744 | Glucose-6-phosphate isomerase | unknown | 13.72 | -2.01 | FALSE | TRUE | TRUE |
| **234** | P27708 | CAD protein | unknown | 9.07 | -13.07 | FALSE | TRUE | TRUE |
| **235** | P61026 | Ras-related protein Rab-10 | unknown | -11.92 | 20.30 | FALSE | TRUE | TRUE |
| **236** | P31947 | 14-3-3 protein sigma | unknown | 21.94 | 25.58 | FALSE | TRUE | TRUE |
| **237** | P61106 | Ras-related protein Rab-14 | unknown | -33.55 | 13.60 | FALSE | TRUE | TRUE |
| **238** | O14979 | Heterogeneous nuclear ribonucleop | NUCLEUS | -20.50 | -33.22 | FALSE | TRUE | TRUE |
| **239** | P62910 | 60S ribosomal protein L32 | RIBOSOME 60S | 15.15 | -22.12 | FALSE | TRUE | TRUE |
| **240** | P18621 | 60S ribosomal protein L17 | RIBOSOME 60S | 16.16 | -21.89 | TRUE | TRUE | TRUE |
| **241** | Q04917 | 14-3-3 protein eta | unknown | 11.55 | 14.00 | FALSE | TRUE | TRUE |
| **242** | P11413 | Glucose-6-phosphate 1-dehydroger | unknown | 15.89 | 11.14 | TRUE | TRUE | TRUE |
| **243** | Q15286 | Ras-related protein Rab-35 | PM | -25.26 | 16.30 | FALSE | TRUE | TRUE |
| **244** | P53999 | Activated RNA polymerase II transc | unknown | 17.13 | 3.05 | TRUE | TRUE | TRUE |
| **245** | O43175 | D-3-phosphoglycerate dehydrogen | unknown | 12.51 | 10.15 | FALSE | TRUE | TRUE |
| **246** | P31948 | Stress-induced-phosphoprotein 1 | CYTOSOL | 22.26 | 22.77 | TRUE | TRUE | TRUE |
| **247** | P23246 | Splicing factor, proline- and glutami | NUCLEUS | 2.00 | -33.44 | TRUE | TRUE | TRUE |
| **248** | P49756 | RNA-binding protein 25 | NUCLEUS | -9.16 | -35.92 | TRUE | TRUE | TRUE |
| **249** | P62269 | 40S ribosomal protein S18 | RIBOSOME 40S | 10.97 | -23.12 | FALSE | TRUE | TRUE |
| **250** | O43143 | Pre-mRNA-splicing factor ATP-depe | NUCLEUS | -12.60 | -29.38 | TRUE | TRUE | TRUE |
| **251** | Q7Z6E9 | E3 ubiquitin-protein ligase RBBP6 | NUCLEUS | -10.08 | -35.24 | TRUE | TRUE | TRUE |
| **252** | Q9P258 | Protein RCC2 | unknown | -15.60 | -17.90 | FALSE | TRUE | TRUE |
| **253** | P00367 | Glutamate dehydrogenase 1, mitoc | MITOCHONDRIA | 33.27 | -14.00 | FALSE | TRUE | TRUE |
| **254** | P00966 | Argininosuccinate synthase | unknown | 10.05 | -2.65 | FALSE | TRUE | TRUE |
| **255** | P29692 | Elongation factor 1-delta | NUCLEUS | -8.70 | -13.58 | FALSE | TRUE | TRUE |
| **256** | P29692 | Elongation factor 1-delta | NUCLEUS | 2.09 | 6.56 | FALSE | TRUE | TRUE |
| **257** | P61006 | Ras-related protein Rab-8A | PM | -28.69 | 12.05 | TRUE | TRUE | TRUE |
| **258** | P62277 | 40S ribosomal protein S13 | RIBOSOME 40S | 11.88 | -23.11 | FALSE | TRUE | TRUE |
| **259** | Q9Y230 | RuvB-like 2 | NUCLEUS | -13.57 | -27.19 | TRUE | TRUE | TRUE |
| **260** | P84085 | ADP-ribosylation factor 5 | unknown | 14.60 | 6.48 | TRUE | TRUE | TRUE |
| **261** | P22234 | Multifunctional protein ADE2 | unknown | 13.08 | -2.06 | TRUE | TRUE | TRUE |
| **262** | O95433 | Activator of 90 kDa heat shock prot | unknown | 20.55 | 18.96 | FALSE | TRUE | TRUE |

| | | | | | | | | |
|---|---|---|---|---|---|---|---|---|
| **263** | P46940 | Ras GTPase-activating-like protein I | unknown | 3.25 | 9.11 | TRUE | TRUE | TRUE |
| **264** | P40926 | Malate dehydrogenase, mitochond | MITOCHONDRIA | 40.13 | -3.97 | FALSE | TRUE | TRUE |
| **265** | Q9Y3F4 | Serine-threonine kinase receptor-as | unknown | 18.04 | 10.51 | FALSE | TRUE | TRUE |
| **266** | Q14839 | Chromodomain-helicase-DNA-bindi | NUCLEUS | -25.65 | -25.98 | TRUE | TRUE | TRUE |
| **267** | P09382 | Galectin-1 | CYTOSOL | 15.26 | 18.52 | FALSE | TRUE | TRUE |
| **268** | P05198 | Eukaryotic translation initiation fact | unknown | 9.82 | -1.65 | FALSE | TRUE | TRUE |
| **269** | P52272 | Heterogeneous nuclear ribonucleop | NUCLEUS | -12.12 | -35.94 | TRUE | TRUE | TRUE |
| **270** | P55265 | Double-stranded RNA-specific aden | NUCLEUS | -10.23 | -35.84 | FALSE | TRUE | TRUE |
| **271** | P46779 | 60S ribosomal protein L28 | RIBOSOME 60S | 17.65 | -21.02 | TRUE | TRUE | TRUE |
| **272** | P07237 | Protein disulfide-isomerase | ER | -0.80 | 39.13 | FALSE | TRUE | TRUE |
| **273** | P04792 | Heat shock protein beta-1 | unknown | 9.33 | 18.75 | TRUE | TRUE | TRUE |
| **274** | Q13162 | Peroxiredoxin-4 | unknown | 1.19 | 39.97 | TRUE | TRUE | TRUE |
| **275** | O15371 | Eukaryotic translation initiation fact | unknown | 13.73 | -10.93 | FALSE | TRUE | TRUE |
| **276** | Q9BQ39 | ATP-dependent RNA helicase DDX5 | NUCLEUS | -1.22 | -35.11 | FALSE | TRUE | TRUE |
| **277** | P51659 | Peroxisomal multifunctional enzym | PEROXISOME | 52.72 | -3.48 | FALSE | TRUE | TRUE |
| **278** | Q13200 | 26S proteasome non-ATPase regula | PROTEASOME | 21.54 | 6.80 | FALSE | TRUE | TRUE |
| **279** | Q14690 | Protein RRP5 homolog | NUCLEUS | -1.15 | -41.12 | FALSE | TRUE | TRUE |
| **280** | Q8TDN6 | Ribosome biogenesis protein BRX1 I | NUCLEUS | 0.36 | -37.46 | FALSE | TRUE | TRUE |
| **281** | P26368 | Splicing factor U2AF 65 kDa subunit | NUCLEUS | -6.29 | -31.56 | TRUE | TRUE | TRUE |
| **282** | P62879 | Guanine nucleotide-binding protein | PM | -27.45 | 7.10 | FALSE | TRUE | TRUE |
| **283** | P12004 | Proliferating cell nuclear antigen | unknown | 26.44 | 21.57 | FALSE | TRUE | TRUE |
| **284** | P49915 | GMP synthase [glutamine-hydrolyz | CYTOSOL | 17.49 | 6.28 | FALSE | TRUE | TRUE |
| **285** | O00299 | Chloride intracellular channel protei | unknown | 12.99 | 15.68 | FALSE | TRUE | TRUE |
| **286** | P41440 | Folate transporter 1 | PM | -29.95 | 9.67 | TRUE | TRUE | TRUE |
| **287** | Q13347 | Eukaryotic translation initiation fact | unknown | 13.53 | -10.84 | FALSE | TRUE | TRUE |
| **288** | P31939 | Bifunctional purine biosynthesis pro | CYTOSOL | 20.41 | 20.74 | FALSE | TRUE | TRUE |
| **289** | Q96E39 | RNA binding motif protein, X-linked- | NUCLEUS | 1.56 | -35.55 | TRUE | TRUE | TRUE |
| **290** | P30101 | Protein disulfide-isomerase A3 | ER | -6.18 | 35.78 | FALSE | TRUE | TRUE |
| **291** | Q99848 | Probable rRNA-processing protein E | NUCLEUS | 0.90 | -35.44 | TRUE | TRUE | TRUE |
| **292** | Q9BZE4 | Nucleolar GTP-binding protein 1 | NUCLEUS | 1.93 | -37.90 | FALSE | TRUE | TRUE |
| **293** | P22102 | Trifunctional purine biosynthetic pro | CYTOSOL | 15.66 | 5.16 | FALSE | TRUE | TRUE |
| **294** | Q99575 | Ribonucleases P/MRP protein subur | NUCLEUS | -0.71 | -25.16 | FALSE | TRUE | TRUE |
| **295** | Q96RQ3 | Methylcrotonoyl-CoA carboxylase s | MITOCHONDRIA | 39.74 | -9.35 | TRUE | TRUE | TRUE |
| **296** | P30086 | Phosphatidylethanolamine-binding | CYTOSOL | 23.35 | 19.57 | TRUE | TRUE | TRUE |
| **297** | P17812 | CTP synthase 1 | unknown | 15.48 | 5.83 | FALSE | TRUE | TRUE |
| **298** | Q14974 | Importin subunit beta-1 | unknown | 21.41 | 12.01 | FALSE | TRUE | TRUE |
| **299** | P67809 | Nuclease-sensitive element-binding | NUCLEUS | 6.95 | -12.17 | FALSE | TRUE | TRUE |
| **300** | P62851 | 40S ribosomal protein S25 | unknown | 12.86 | -19.15 | FALSE | TRUE | TRUE |

| | | | | | | | | | |
|---|---|---|---|---|---|---|---|---|---|
| **301** | O15067 | Phosphoribosylformylglycinamidine | CYTOSOL | 21.21 | 22.84 | FALSE | TRUE | TRUE |
| **302** | P11586 | C-1-tetrahydrofolate synthase, cyto | RIBOSOME 60S | 15.25 | -21.44 | FALSE | TRUE | TRUE |
| **303** | P18085 | ADP-ribosylation factor 4 | unknown | 10.72 | 8.24 | FALSE | TRUE | TRUE |
| **304** | Q08J23 | tRNA (cytosine(34)-C(5))-methyltra | unknown | 16.69 | 6.37 | TRUE | TRUE | TRUE |
| **305** | P04083 | Annexin A1 | unknown | 14.47 | 18.56 | TRUE | TRUE | TRUE |
| **306** | P09960 | Leukotriene A-4 hydrolase | unknown | 24.39 | 23.73 | FALSE | TRUE | TRUE |
| **307** | P09429 | High mobility group protein B1 | unknown | -12.63 | -8.51 | FALSE | TRUE | TRUE |
| **308** | Q96PK6 | RNA-binding protein 14 | NUCLEUS | -9.34 | -34.88 | FALSE | TRUE | TRUE |
| **309** | P35232 | Prohibitin | MITOCHONDRIA | 33.99 | -8.06 | FALSE | TRUE | TRUE |
| **310** | Q9BQG0 | Myb-binding protein 1A | NUCLEUS | 0.42 | -39.38 | TRUE | TRUE | TRUE |
| **311** | O43776 | Asparagine--tRNA ligase, cytoplasm | unknown | 19.95 | 9.94 | FALSE | TRUE | TRUE |
| **312** | Q15181 | Inorganic pyrophosphatase | unknown | 18.03 | 24.51 | FALSE | TRUE | TRUE |
| **313** | Q15393 | Splicing factor 3B subunit 3 | NUCLEUS | 4.04 | -35.57 | FALSE | TRUE | TRUE |
| **314** | P51148 | Ras-related protein Rab-5C | unknown | -23.68 | 1.41 | FALSE | TRUE | TRUE |
| **315** | P12814 | Alpha-actinin-1 | unknown | 4.03 | 9.41 | FALSE | TRUE | TRUE |
| **316** | Q12907 | Vesicular integral-membrane prote | unknown | -9.77 | 32.76 | FALSE | TRUE | TRUE |
| **317** | Q16658 | Fascin | unknown | 10.61 | 14.51 | FALSE | TRUE | TRUE |
| **318** | P41252 | Isoleucine--tRNA ligase, cytoplasmi | unknown | 5.80 | -3.22 | FALSE | TRUE | TRUE |
| **319** | Q92598 | Heat shock protein 105 kDa | CYTOSOL | 21.46 | 18.70 | FALSE | TRUE | TRUE |
| **320** | P08237 | ATP-dependent 6-phosphofructoki | unknown | 2.56 | -15.37 | FALSE | TRUE | TRUE |
| **321** | P31153 | S-adenosylmethionine synthase iso | CYTOSOL | 23.63 | 20.15 | FALSE | TRUE | TRUE |
| **322** | P00491 | Purine nucleoside phosphorylase | unknown | 24.09 | 22.25 | FALSE | TRUE | TRUE |
| **323** | Q9Y277 | Voltage-dependent anion-selective | unknown | 28.31 | -6.39 | FALSE | TRUE | TRUE |
| **324** | P20700 | Lamin-B1 | unknown | 23.61 | -12.61 | TRUE | TRUE | TRUE |
| **325** | Q14498 | RNA-binding protein 39 | NUCLEUS | 2.99 | -31.24 | TRUE | TRUE | TRUE |
| **326** | Q99497 | Protein/nucleic acid deglycase DJ-1 | unknown | 9.67 | 9.18 | FALSE | TRUE | TRUE |
| **327** | P18206 | Vinculin | unknown | 12.84 | 15.48 | TRUE | TRUE | TRUE |
| **328** | P55209 | Nucleosome assembly protein 1-lik | CYTOSOL | 16.48 | 17.25 | TRUE | TRUE | TRUE |
| **329** | P46782 | 40S ribosomal protein S5 | RIBOSOME 40S | 11.18 | -22.06 | FALSE | TRUE | TRUE |
| **330** | Q13247 | Serine/arginine-rich splicing factor 6 | unknown | -10.85 | -30.75 | FALSE | TRUE | TRUE |
| **331** | P41250 | Glycine--tRNA ligase | unknown | 9.68 | 9.03 | FALSE | TRUE | TRUE |
| **332** | P55884 | Eukaryotic translation initiation fact | unknown | 13.29 | -10.80 | TRUE | TRUE | TRUE |
| **333** | P39019 | 40S ribosomal protein S19 | RIBOSOME 40S | 11.85 | -23.20 | TRUE | TRUE | TRUE |
| **334** | P13489 | Ribonuclease inhibitor | CYTOSOL | 21.23 | 20.68 | FALSE | TRUE | TRUE |
| **335** | O75534 | Cold shock domain-containing prote | unknown | 14.51 | 4.74 | TRUE | TRUE | TRUE |
| **336** | Q9HAV0 | Guanine nucleotide-binding protein | PM | -29.58 | 2.14 | FALSE | TRUE | TRUE |
| **337** | P49736 | DNA replication licensing factor MC | unknown | 24.19 | 14.14 | FALSE | TRUE | TRUE |
| **338** | Q01650 | Large neutral amino acids transport | PM | -26.50 | 6.16 | TRUE | TRUE | TRUE |

| | | | | | | | | |
|---|---|---|---|---|---|---|---|---|
| **339** | Q99729 | Heterogeneous nuclear ribonucleop | NUCLEUS | -20.35 | -33.06 | FALSE | TRUE | TRUE |
| **340** | O75694 | Nuclear pore complex protein Nup1 | NUCLEUS | -22.01 | -29.58 | FALSE | TRUE | TRUE |
| **341** | O43242 | 26S proteasome non-ATPase regul | PROTEASOME | 20.67 | 6.00 | FALSE | TRUE | TRUE |
| **342** | Q04637 | Eukaryotic translation initiation fact | NUCLEUS | -1.55 | -15.34 | TRUE | TRUE | TRUE |
| **343** | P56192 | Methionine--tRNA ligase, cytoplasm | unknown | 5.87 | -3.15 | FALSE | TRUE | TRUE |
| **344** | P52597 | Heterogeneous nuclear ribonucleop | unknown | -15.72 | -31.72 | TRUE | TRUE | TRUE |
| **345** | Q13151 | Heterogeneous nuclear ribonucleop | NUCLEUS | -12.62 | -35.62 | FALSE | TRUE | TRUE |
| **346** | P51153 | Ras-related protein Rab-13 | PM | -25.24 | 12.88 | FALSE | TRUE | TRUE |
| **347** | P26640 | Valine--tRNA ligase | unknown | 14.69 | 4.63 | TRUE | TRUE | TRUE |
| **348** | P33991 | DNA replication licensing factor MC | unknown | -15.52 | -31.48 | FALSE | TRUE | TRUE |
| **349** | P18754 | Regulator of chromosome condens | NUCLEUS-CHROMATIN | -22.49 | -16.08 | FALSE | TRUE | TRUE |
| **350** | P55786 | Puromycin-sensitive aminopeptida | CYTOSOL | 17.97 | 15.39 | FALSE | TRUE | TRUE |
| **351** | Q15717 | ELAV-like protein 1 | NUCLEUS | -13.78 | -36.52 | FALSE | TRUE | TRUE |
| **352** | Q92621 | Nuclear pore complex protein Nup2 | NUCLEUS | -21.26 | -29.54 | FALSE | TRUE | TRUE |
| **353** | Q9P2J5 | Leucine--tRNA ligase, cytoplasmic | unknown | 21.01 | 12.04 | FALSE | TRUE | TRUE |
| **354** | Q15050 | Ribosome biogenesis regulatory pr | NUCLEUS | 1.82 | -37.86 | TRUE | TRUE | TRUE |
| **355** | P17858 | ATP-dependent 6-phosphofructoki | unknown | 8.38 | -4.44 | FALSE | TRUE | TRUE |
| **356** | P67936 | Tropomyosin alpha-4 chain | unknown | 10.25 | 18.74 | FALSE | TRUE | TRUE |
| **357** | Q10471 | Polypeptide N-acetylgalactosaminy | GOLGI | -19.34 | 32.43 | FALSE | TRUE | TRUE |
| **358** | P62857 | 40S ribosomal protein S28 | RIBOSOME 40S | 11.19 | -23.02 | FALSE | TRUE | TRUE |
| **359** | Q02880 | DNA topoisomerase 2-beta | NUCLEUS-CHROMATIN | -21.42 | -13.54 | TRUE | TRUE | TRUE |
| **360** | Q15084 | Protein disulfide-isomerase A6 | ER | 1.46 | 39.28 | FALSE | TRUE | TRUE |
| **361** | P62873 | Guanine nucleotide-binding protein | PM | -27.75 | 6.84 | FALSE | TRUE | TRUE |
| **362** | P08621 | U1 small nuclear ribonucleoprotein | NUCLEUS | 6.10 | -34.33 | FALSE | TRUE | TRUE |
| **363** | Q13813 | Spectrin alpha chain, non-erythrocy | unknown | 1.88 | 26.12 | TRUE | TRUE | TRUE |
| **364** | Q13813 | Spectrin alpha chain, non-erythrocy | unknown | 1.91 | 26.07 | TRUE | TRUE | TRUE |
| **365** | P60953 | Cell division control protein 42 hom | PM | -23.38 | 13.73 | TRUE | TRUE | TRUE |
| **366** | P06753 | Tropomyosin alpha-3 chain | unknown | 10.28 | 13.07 | FALSE | TRUE | TRUE |
| **367** | O75390 | Citrate synthase, mitochondrial | MITOCHONDRIA | 40.98 | -8.15 | FALSE | TRUE | TRUE |
| **368** | Q96GQ7 | Probable ATP-dependent RNA helic | NUCLEUS | 0.91 | -36.59 | FALSE | TRUE | TRUE |
| **369** | Q2NL82 | Pre-rRNA-processing protein TSR1 h | NUCLEUS | 3.86 | -31.08 | FALSE | TRUE | TRUE |
| **370** | Q14566 | DNA replication licensing factor MC | unknown | -15.94 | -31.97 | FALSE | TRUE | TRUE |
| **371** | Q9Y617 | Phosphoserine aminotransferase | CYTOSOL | 20.97 | 22.88 | FALSE | TRUE | TRUE |
| **372** | Q8NI36 | WD repeat-containing protein 36 | NUCLEUS | -1.78 | -40.80 | FALSE | TRUE | TRUE |
| **373** | Q05519 | Serine/arginine-rich splicing factor 1 | NUCLEUS | 3.95 | -31.25 | FALSE | TRUE | TRUE |
| **374** | P08243 | Asparagine synthetase [glutamine- | CYTOSOL | 20.45 | 14.42 | FALSE | TRUE | TRUE |
| **375** | P53985 | Monocarboxylate transporter 1 | PM | -24.11 | 18.08 | TRUE | TRUE | TRUE |
| **376** | P60891 | Ribose-phosphate pyrophosphokin | unknown | 8.14 | -4.66 | FALSE | TRUE | TRUE |

| | | | | | | | | | |
|---|---|---|---|---|---|---|---|---|---|
| **377** | P62495 | Eukaryotic peptide chain release fa | unknown | -7.99 | -0.37 | FALSE | TRUE | TRUE |
| **378** | O43684 | Mitotic checkpoint protein BUB3 | NUCLEUS | 1.53 | -30.44 | FALSE | TRUE | TRUE |
| **379** | Q8IYB3 | Serine/arginine repetitive matrix pr | NUCLEUS | 0.99 | -19.22 | TRUE | TRUE | TRUE |
| **380** | Q8NBJ5 | Procollagen galactosyltransferase 1 | ER | 4.56 | 39.09 | FALSE | TRUE | TRUE |
| **381** | P42704 | Leucine-rich PPR motif-containing p | MITOCHONDRIA | 42.73 | -11.81 | FALSE | TRUE | TRUE |
| **382** | Q9UMS4 | Pre-mRNA-processing factor 19 | NUCLEUS | -3.09 | -32.14 | FALSE | TRUE | TRUE |
| **383** | Q9NQ29 | Putative RNA-binding protein Luc7-l | unknown | 7.40 | -24.29 | FALSE | TRUE | TRUE |
| **384** | Q9NVP1 | ATP-dependent RNA helicase DDX1 | NUCLEUS | 0.41 | -36.38 | FALSE | TRUE | TRUE |
| **385** | Q13283 | Ras GTPase-activating protein-bind | unknown | 9.04 | -20.96 | TRUE | TRUE | TRUE |
| **386** | Q9Y265 | RuvB-like 1 | NUCLEUS | -13.39 | -26.92 | TRUE | TRUE | TRUE |
| **387** | P61513 | 60S ribosomal protein L37a | RIBOSOME 60S | 17.49 | -21.08 | FALSE | TRUE | TRUE |
| **388** | Q7L2E3 | ATP-dependent RNA helicase DHX3 | unknown | -7.93 | -3.13 | FALSE | TRUE | TRUE |
| **389** | Q15185 | Prostaglandin E synthase 3 | CYTOSOL | 17.67 | 14.28 | FALSE | TRUE | TRUE |
| **390** | Q9NZ01 | Very-long-chain enoyl-CoA reducta | ER | 3.21 | 32.92 | FALSE | TRUE | TRUE |
| **391** | P62136 | Serine/threonine-protein phosphata | unknown | 1.53 | -7.79 | FALSE | TRUE | FALSE |
| **392** | P08754 | Guanine nucleotide-binding protein | PM | -24.52 | 15.65 | FALSE | TRUE | TRUE |
| **393** | P62140 | Serine/threonine-protein phosphata | NUCLEUS | 1.48 | -7.78 | FALSE | TRUE | TRUE |
| **394** | P05386 | 60S acidic ribosomal protein P1 | RIBOSOME 60S | 15.84 | -20.59 | FALSE | TRUE | TRUE |
| **395** | P13667 | Protein disulfide-isomerase A4 | ER | -0.70 | 39.68 | FALSE | TRUE | TRUE |
| **396** | P52701 | DNA mismatch repair protein Msh6 | NUCLEUS | -10.44 | -14.61 | FALSE | TRUE | TRUE |
| **397** | P38606 | V-type proton ATPase catalytic sub | unknown | -17.50 | 3.82 | FALSE | TRUE | TRUE |
| **398** | Q9H583 | HEAT repeat-containing protein 1 | NUCLEUS | -2.49 | -41.32 | FALSE | TRUE | TRUE |
| **399** | Q96AG4 | Leucine-rich repeat-containing prot | unknown | 4.54 | 37.62 | TRUE | TRUE | TRUE |
| **400** | P18077 | 60S ribosomal protein L35a | RIBOSOME 60S | 16.81 | -21.37 | FALSE | TRUE | TRUE |
| **401** | Q14152 | Eukaryotic translation initiation fact | unknown | 13.70 | -10.98 | FALSE | TRUE | TRUE |
| **402** | P06493 | Cyclin-dependent kinase 1 | unknown | -4.54 | -8.01 | FALSE | TRUE | TRUE |
| **403** | Q9Y3I0 | tRNA-splicing ligase RtcB homolog | unknown | 5.31 | -24.86 | FALSE | TRUE | TRUE |
| **404** | Q7Z2W4 | Zinc finger CCCH-type antiviral prot | unknown | -7.95 | -0.26 | TRUE | TRUE | TRUE |
| **405** | P84098 | 60S ribosomal protein L19 | RIBOSOME 60S | 16.25 | -21.71 | TRUE | TRUE | TRUE |
| **406** | P63000 | Ras-related C3 botulinum toxin subs | PM | -25.72 | 11.25 | FALSE | TRUE | FALSE |
| **407** | Q13263 | Transcription intermediary factor 1- | NUCLEUS | -13.80 | -29.50 | TRUE | TRUE | TRUE |
| **408** | P11908 | Ribose-phosphate pyrophosphokin | unknown | 8.23 | -1.76 | FALSE | TRUE | TRUE |
| **409** | P35613 | Basigin | PM | -26.78 | 12.42 | TRUE | TRUE | TRUE |
| **410** | Q9Y5B9 | FACT complex subunit SPT16 | unknown | -12.26 | -8.93 | FALSE | TRUE | TRUE |
| **411** | P42766 | 60S ribosomal protein L35 | RIBOSOME 60S | 17.83 | -22.46 | TRUE | TRUE | TRUE |
| **412** | P14550 | Alcohol dehydrogenase [NADP(+)] | CYTOSOL | 22.81 | 21.63 | FALSE | TRUE | TRUE |
| **413** | Q92769 | Histone deacetylase 2 | NUCLEUS | -25.22 | -27.51 | FALSE | TRUE | TRUE |
| **414** | Q01081 | Splicing factor U2AF 35 kDa subuni | NUCLEUS | 1.82 | -27.85 | TRUE | TRUE | TRUE |

| | | | | | | | | | |
|---|---|---|---|---|---|---|---|---|---|
| **415** | O00159 | Unconventional myosin-Ic | unknown | -9.77 | 25.21 | FALSE | TRUE | TRUE |
| **416** | Q12905 | Interleukin enhancer-binding factor | NUCLEUS | -12.06 | -35.66 | FALSE | TRUE | TRUE |
| **417** | Q16576 | Histone-binding protein RBBP7 | unknown | -16.24 | -31.30 | FALSE | TRUE | TRUE |
| **418** | Q9ULV4 | Coronin-1C | unknown | -3.49 | 1.95 | TRUE | TRUE | TRUE |
| **419** | P31150 | Rab GDP dissociation inhibitor alpha | unknown | 16.16 | 23.29 | FALSE | TRUE | TRUE |
| **420** | Q13523 | Serine/threonine-protein kinase PR | NUCLEUS | 2.88 | -33.34 | TRUE | TRUE | TRUE |
| **421** | P30044 | Peroxiredoxin-5, mitochondrial | unknown | 22.23 | -3.90 | FALSE | TRUE | TRUE |
| **422** | O00410 | Importin-5 | unknown | 9.80 | 8.78 | FALSE | TRUE | TRUE |
| **423** | P61586 | Transforming protein RhoA | PM | -29.44 | 8.67 | FALSE | TRUE | TRUE |
| **424** | Q9Y2L1 | Exosome complex exonuclease RRP | NUCLEUS | -13.37 | -27.07 | FALSE | TRUE | TRUE |
| **425** | O76094 | Signal recognition particle subunit S | unknown | 5.33 | -4.05 | FALSE | TRUE | TRUE |
| **426** | P16989 | Y-box-binding protein 3 | NUCLEUS | 9.49 | -6.90 | FALSE | TRUE | TRUE |
| **427** | P43243 | Matrin-3 | NUCLEUS | -11.37 | -36.46 | TRUE | TRUE | TRUE |
| **428** | P62995 | Transformer-2 protein homolog bet | NUCLEUS | -6.17 | -39.08 | FALSE | TRUE | TRUE |
| **429** | P63220 | 40S ribosomal protein S21 | RIBOSOME 40S | 11.73 | -22.25 | FALSE | TRUE | TRUE |
| **430** | Q8NC51 | Plasminogen activator inhibitor 1 RI | unknown | 10.25 | -21.18 | TRUE | TRUE | TRUE |
| **431** | Q92688 | Acidic leucine-rich nuclear phospho | unknown | 9.77 | 8.11 | FALSE | TRUE | TRUE |
| **432** | P46783 | 40S ribosomal protein S10 | RIBOSOME 40S | 10.99 | -22.86 | FALSE | TRUE | TRUE |
| **433** | Q9BUQ8 | Probable ATP-dependent RNA helic | NUCLEUS | 3.50 | -34.35 | FALSE | TRUE | TRUE |
| **434** | Q13085 | Acetyl-CoA carboxylase 1 | unknown | 9.85 | -2.81 | TRUE | TRUE | TRUE |
| **435** | P31949 | Protein S100-A11 | unknown | 15.12 | 18.68 | TRUE | TRUE | TRUE |
| **436** | O43865 | S-adenosylhomocysteine hydrolase | unknown | 7.02 | 7.85 | TRUE | TRUE | TRUE |
| **437** | P51858 | Hepatoma-derived growth factor | unknown | 26.95 | 21.86 | FALSE | TRUE | TRUE |
| **438** | P52565 | Rho GDP-dissociation inhibitor 1 | unknown | 18.03 | 24.49 | FALSE | TRUE | TRUE |
| **439** | P14314 | Glucosidase 2 subunit beta | ER | -2.43 | 37.47 | FALSE | TRUE | TRUE |
| **440** | O15144 | Actin-related protein 2/3 complex s | unknown | 1.84 | -15.37 | FALSE | TRUE | TRUE |
| **441** | P20339 | Ras-related protein Rab-5A | unknown | -19.62 | 13.04 | FALSE | TRUE | TRUE |
| **442** | Q96AE4 | Far upstream element-binding prot | unknown | -13.90 | -31.77 | TRUE | TRUE | TRUE |
| **443** | Q96AE4 | Far upstream element-binding prot | unknown | -13.73 | -31.85 | TRUE | TRUE | TRUE |
| **444** | P19623 | Spermidine synthase | CYTOSOL | 21.18 | 18.62 | FALSE | TRUE | TRUE |
| **445** | O14818 | Proteasome subunit alpha type-7 | PROTEASOME | 20.89 | 5.75 | FALSE | TRUE | TRUE |
| **446** | Q8IVT2 | Mitotic interactor and substrate of P | unknown | -6.07 | 10.31 | TRUE | TRUE | TRUE |
| **447** | P17931 | Galectin-3 | unknown | -24.29 | 16.59 | FALSE | TRUE | TRUE |
| **448** | P20042 | Eukaryotic translation initiation fact | NUCLEUS | -3.30 | -13.86 | TRUE | TRUE | TRUE |
| **449** | P13693 | Translationally-controlled tumor pro | unknown | 14.70 | -10.35 | FALSE | TRUE | TRUE |
| **450** | Q13185 | Chromobox protein homolog 3 | NUCLEUS | -15.71 | -29.35 | TRUE | TRUE | TRUE |
| **451** | Q16181 | Septin-7 | unknown | -2.47 | 4.98 | TRUE | TRUE | TRUE |
| **452** | Q16181 | Septin-7 | unknown | -2.33 | 4.94 | TRUE | TRUE | TRUE |

| | | | | | | | | |
|---|---|---|---|---|---|---|---|---|
| **453** | P12268 | Inosine-5'-monophosphate dehydr | PROTEASOME | 20.62 | 6.46 | FALSE | TRUE | TRUE |
| **454** | Q9UNX4 | WD repeat-containing protein 3 | NUCLEUS | -1.39 | -40.57 | FALSE | TRUE | TRUE |
| **455** | P55084 | Trifunctional enzyme subunit beta, | MITOCHONDRIA | 41.47 | -4.66 | FALSE | TRUE | TRUE |
| **456** | O75153 | Clustered mitochondria protein hon | unknown | 8.73 | -13.63 | FALSE | TRUE | TRUE |
| **457** | P04899 | Guanine nucleotide-binding protein | PM | -26.14 | 14.20 | FALSE | TRUE | TRUE |
| **458** | Q96T88 | E3 ubiquitin-protein ligase UHRF1 | NUCLEUS | -25.79 | -23.64 | FALSE | TRUE | TRUE |
| **459** | P15170 | Eukaryotic peptide chain release fa | CYTOSOL | 19.00 | 11.51 | TRUE | TRUE | TRUE |
| **460** | P21291 | Cysteine and glycine-rich protein 1 | unknown | -5.94 | -2.37 | TRUE | TRUE | TRUE |
| **461** | O94776 | Metastasis-associated protein MTA | NUCLEUS | -20.38 | -32.17 | TRUE | TRUE | TRUE |
| **462** | P00505 | Aspartate aminotransferase, mitocl | MITOCHONDRIA | 35.56 | -4.98 | FALSE | TRUE | TRUE |
| **463** | P98179 | RNA-binding protein 3 | NUCLEUS | -19.17 | -32.68 | FALSE | TRUE | TRUE |
| **464** | Q01082 | Spectrin beta chain, non-erythrocyt | unknown | 1.29 | 25.34 | TRUE | TRUE | TRUE |
| **465** | Q01082 | Spectrin beta chain, non-erythrocyt | unknown | 1.65 | 26.00 | TRUE | TRUE | TRUE |
| **466** | P28066 | Proteasome subunit alpha type-5 | PROTEASOME | 22.19 | 6.71 | FALSE | TRUE | TRUE |
| **467** | P31930 | Cytochrome b-c1 complex subunit 1 | MITOCHONDRIA | 31.94 | -13.45 | FALSE | TRUE | TRUE |
| **468** | Q15019 | Septin-2 | unknown | -2.39 | 4.73 | TRUE | TRUE | TRUE |
| **469** | O95232 | Luc7-like protein 3 | unknown | 5.93 | -26.30 | TRUE | TRUE | TRUE |
| **470** | P61619 | Protein transport protein Sec61 sub | ER | 5.76 | 37.40 | FALSE | TRUE | TRUE |
| **471** | P46063 | ATP-dependent DNA helicase Q1 | unknown | -21.41 | -21.39 | FALSE | TRUE | TRUE |
| **472** | Q08945 | FACT complex subunit SSRP1 | unknown | -12.27 | -8.99 | FALSE | TRUE | TRUE |
| **473** | O95831 | Apoptosis-inducing factor 1, mitoch | MITOCHONDRIA | 34.34 | -11.85 | FALSE | TRUE | TRUE |
| **474** | P30740 | Leukocyte elastase inhibitor | CYTOSOL | 11.76 | 20.41 | FALSE | TRUE | TRUE |
| **475** | O75821 | Eukaryotic translation initiation fact | unknown | 13.16 | -10.81 | TRUE | TRUE | TRUE |
| **476** | P51572 | B-cell receptor-associated protein 3 | ER | -11.18 | 30.26 | FALSE | TRUE | TRUE |
| **477** | Q16881 | Thioredoxin reductase 1, cytoplasm | unknown | 13.39 | 16.86 | TRUE | TRUE | TRUE |
| **478** | P61019 | Ras-related protein Rab-2A | unknown | -18.45 | 20.95 | FALSE | TRUE | TRUE |
| **479** | O14744 | Protein arginine N-methyltransferas | unknown | 18.36 | 16.85 | FALSE | TRUE | TRUE |
| **480** | O95373 | Importin-7 | unknown | 19.36 | 15.98 | FALSE | TRUE | TRUE |
| **481** | Q15369 | Elongin-C | unknown | 19.47 | 11.97 | FALSE | TRUE | TRUE |
| **482** | P51571 | Translocon-associated protein subu | ER | 2.48 | 35.39 | FALSE | TRUE | TRUE |
| **483** | O96019 | Actin-like protein 6A | NUCLEUS | -17.07 | -28.30 | FALSE | TRUE | TRUE |
| **484** | Q02040 | A-kinase anchor protein 17A | NUCLEUS | 1.12 | -28.98 | TRUE | TRUE | TRUE |
| **485** | P08559 | Pyruvate dehydrogenase E1 compo | MITOCHONDRIA | 44.03 | -12.40 | FALSE | TRUE | TRUE |
| **486** | P13987 | CD59 glycoprotein | unknown | -21.58 | 10.55 | FALSE | TRUE | TRUE |
| **487** | P30520 | Adenylosuccinate synthetase isozy | CYTOSOL | 22.83 | 19.22 | FALSE | TRUE | TRUE |
| **488** | P49755 | Transmembrane emp24 domain-cc | unknown | -12.95 | 30.72 | FALSE | TRUE | TRUE |
| **489** | Q9Y3U8 | 60S ribosomal protein L36 | RIBOSOME 60S | 18.10 | -21.69 | TRUE | TRUE | TRUE |
| **490** | Q92499 | ATP-dependent RNA helicase DDX1 | unknown | 5.23 | -25.00 | FALSE | TRUE | TRUE |

| | | | | | | | | |
|---|---|---|---|---|---|---|---|---|
| **491** | P57088 | Transmembrane protein 33 | unknown | 6.12 | 35.21 | FALSE | TRUE | TRUE |
| **492** | P20618 | Proteasome subunit beta type-1 | PROTEASOME | 21.83 | 6.01 | FALSE | TRUE | TRUE |
| **493** | O75131 | Copine-3 | unknown | 13.56 | 17.44 | FALSE | TRUE | TRUE |
| **494** | P35998 | 26S proteasome regulatory subunit | PROTEASOME | 20.48 | 6.72 | TRUE | TRUE | TRUE |
| **495** | Q9UHD8 | Septin-9 | unknown | -1.97 | 4.39 | TRUE | TRUE | TRUE |
| **496** | Q96RP9 | Elongation factor G, mitochondrial | MITOCHONDRIA | 38.19 | -8.51 | FALSE | TRUE | TRUE |
| **497** | Q01518 | Adenylyl cyclase-associated protei | CYTOSOL | 13.92 | 15.43 | FALSE | TRUE | TRUE |
| **498** | P27694 | Replication protein A 70 kDa DNA-b | unknown | 23.45 | 12.66 | TRUE | TRUE | TRUE |
| **499** | P35637 | RNA-binding protein FUS | NUCLEUS | -6.64 | -30.32 | FALSE | TRUE | TRUE |
| **500** | O00622 | Protein CYR61 | unknown | -32.86 | 6.50 | FALSE | TRUE | TRUE |
| **501** | P59998 | Actin-related protein 2/3 complex s | unknown | 1.54 | -14.55 | FALSE | TRUE | TRUE |
| **502** | Q9BY44 | Eukaryotic translation initiation fact | RIBOSOME 40S | 11.01 | -22.74 | FALSE | TRUE | TRUE |
| **503** | P22695 | Cytochrome b-c1 complex subunit 2 | MITOCHONDRIA | 31.83 | -13.23 | FALSE | TRUE | TRUE |
| **504** | P61020 | Ras-related protein Rab-5B | unknown | -21.94 | 4.60 | FALSE | TRUE | TRUE |
| **505** | Q14739 | Lamin-B receptor | unknown | 23.82 | -12.10 | FALSE | TRUE | TRUE |
| **506** | O96008 | Mitochondrial import receptor subu | MITOCHONDRIA | 30.08 | -6.89 | FALSE | TRUE | TRUE |
| **507** | Q9Y281 | Cofilin-2 | unknown | 3.76 | 9.55 | FALSE | TRUE | TRUE |
| **508** | P27695 | DNA-(apurinic or apyrimidinic site) l | unknown | -12.09 | -14.54 | FALSE | TRUE | TRUE |
| **509** | Q15287 | RNA-binding protein with serine-rich | NUCLEUS | -3.86 | -41.13 | TRUE | TRUE | TRUE |
| **510** | Q9BUJ2 | Heterogeneous nuclear ribonucleop | NUCLEUS | -9.16 | -29.10 | FALSE | TRUE | TRUE |
| **511** | P47897 | Glutamine--tRNA ligase | unknown | 5.67 | -3.29 | FALSE | TRUE | TRUE |
| **512** | P49458 | Signal recognition particle 9 kDa pro | NUCLEUS | 9.17 | -6.73 | FALSE | TRUE | TRUE |
| **513** | O60488 | Long-chain-fatty-acid--CoA ligase 4 | unknown | -1.55 | 26.17 | FALSE | TRUE | TRUE |
| **514** | P20290 | Transcription factor BTF3 | unknown | 7.92 | -18.18 | FALSE | TRUE | TRUE |
| **515** | Q14692 | Ribosome biogenesis protein BMS1 | NUCLEUS | -1.55 | -39.15 | TRUE | TRUE | TRUE |
| **516** | P40925 | Malate dehydrogenase, cytoplasm | CYTOSOL | 21.43 | 21.21 | FALSE | TRUE | TRUE |
| **517** | Q9UBX3 | Mitochondrial dicarboxylate carrier | MITOCHONDRIA | 38.69 | -10.83 | FALSE | TRUE | TRUE |
| **518** | P52209 | 6-phosphogluconate dehydrogenas | unknown | 24.80 | 20.23 | FALSE | TRUE | TRUE |
| **519** | P26196 | Probable ATP-dependent RNA helic | unknown | 5.86 | -20.98 | FALSE | TRUE | TRUE |
| **520** | O15143 | Actin-related protein 2/3 complex s | unknown | 1.40 | -14.47 | FALSE | TRUE | TRUE |
| **521** | O60264 | SWI/SNF-related matrix-associated | NUCLEUS-CHROMATIN | -22.24 | -15.69 | FALSE | TRUE | TRUE |
| **522** | P52292 | Importin subunit alpha-1 | unknown | 11.81 | 1.13 | FALSE | TRUE | TRUE |
| **523** | P49588 | Alanine--tRNA ligase, cytoplasmic | CYTOSOL | 21.76 | 22.70 | TRUE | TRUE | TRUE |
| **524** | Q7L7X3 | Serine/threonine-protein kinase TA | unknown | -14.61 | 13.97 | FALSE | TRUE | TRUE |
| **525** | P16435 | NADPH--cytochrome P450 reductas | ER | -10.54 | 30.39 | FALSE | TRUE | TRUE |
| **526** | Q9Y266 | Nuclear migration protein nudC | unknown | 23.90 | 23.57 | TRUE | TRUE | TRUE |
| **527** | P50454 | Serpin H1 | ER | 3.04 | 38.39 | FALSE | TRUE | TRUE |
| **528** | P60900 | Proteasome subunit alpha type-6 | PROTEASOME | 22.29 | 6.44 | FALSE | TRUE | TRUE |

| | | | | | | | | |
|---|---|---|---|---|---|---|---|---|
| **529** | O75683 | Surfeit locus protein 6 | NUCLEUS | 2.00 | -37.95 | TRUE | TRUE | TRUE |
| **530** | P62314 | Small nuclear ribonucleoprotein Sm | NUCLEUS | 5.10 | -35.02 | FALSE | TRUE | TRUE |
| **531** | P61224 | Ras-related protein Rap-1b | PM | -25.86 | 15.15 | FALSE | TRUE | TRUE |
| **532** | Q15645 | Pachytene checkpoint protein 2 hor | unknown | 15.12 | 7.55 | FALSE | TRUE | TRUE |
| **533** | P47756 | F-actin-capping protein subunit beta | unknown | 5.69 | 4.29 | FALSE | TRUE | TRUE |
| **534** | Q13057 | Bifunctional coenzyme A synthase | unknown | 9.21 | 7.87 | FALSE | TRUE | TRUE |
| **535** | P46977 | Dolichyl-diphosphooligosaccharide- | unknown | 5.53 | 37.01 | TRUE | TRUE | TRUE |
| **536** | P50502 | Hsc70-interacting protein | CYTOSOL | 22.86 | 22.10 | FALSE | TRUE | TRUE |
| **537** | Q12788 | Transducin beta-like protein 3 | NUCLEUS | -2.45 | -40.40 | FALSE | TRUE | TRUE |
| **538** | P34897 | Serine hydroxymethyltransferase, r | MITOCHONDRIA | 39.28 | -6.92 | FALSE | TRUE | TRUE |
| **539** | O00303 | Eukaryotic translation initiation fact | unknown | 13.70 | -10.87 | FALSE | TRUE | TRUE |
| **540** | P53618 | Coatomer subunit beta | unknown | 10.16 | 3.67 | FALSE | TRUE | TRUE |
| **541** | P35610 | Sterol O-acyltransferase 1 | unknown | 3.40 | 31.67 | FALSE | TRUE | TRUE |
| **542** | P23921 | Ribonucleoside-diphosphate reduct | unknown | 11.34 | -1.77 | FALSE | TRUE | TRUE |
| **543** | Q13823 | Nucleolar GTP-binding protein 2 | NUCLEUS | 2.34 | -33.52 | TRUE | TRUE | TRUE |
| **544** | Q8IY81 | pre-rRNA processing protein FTSJ3 | NUCLEUS | 2.90 | -34.80 | TRUE | TRUE | TRUE |
| **545** | P62841 | 40S ribosomal protein S15 | RIBOSOME 40S | 11.69 | -22.83 | FALSE | TRUE | FALSE |
| **546** | P31040 | Succinate dehydrogenase [ubiquino | MITOCHONDRIA | 39.31 | -5.20 | FALSE | TRUE | TRUE |
| **547** | P14868 | Aspartate--tRNA ligase, cytoplasmi | unknown | 5.99 | -3.04 | FALSE | TRUE | TRUE |
| **548** | P52907 | F-actin-capping protein subunit alph | unknown | 5.61 | 4.04 | FALSE | TRUE | TRUE |
| **549** | P00403 | Cytochrome c oxidase subunit 2 | MITOCHONDRIA | 30.48 | -14.79 | FALSE | TRUE | TRUE |
| **550** | Q5SRE5 | Nucleoporin NUP188 homolog | unknown | -6.73 | -22.42 | FALSE | TRUE | TRUE |
| **551** | Q8N163 | Cell cycle and apoptosis regulator p | unknown | 24.01 | 13.55 | TRUE | TRUE | TRUE |
| **552** | P39656 | Dolichyl-diphosphooligosaccharide- | ER | 2.32 | 29.42 | FALSE | TRUE | TRUE |
| **553** | Q6PCB5 | Round spermatid basic protein 1-lik | NUCLEUS | 1.80 | -32.45 | TRUE | TRUE | TRUE |
| **554** | P30048 | Thioredoxin-dependent peroxide re | MITOCHONDRIA | 43.82 | -11.08 | FALSE | TRUE | TRUE |
| **555** | P11177 | Pyruvate dehydrogenase E1 compo | MITOCHONDRIA | 39.09 | -9.76 | FALSE | TRUE | TRUE |
| **556** | P61160 | Actin-related protein 2 | unknown | 1.53 | -14.36 | FALSE | TRUE | TRUE |
| **557** | Q92616 | eIF-2-alpha kinase activator GCN1 | unknown | -3.39 | -11.69 | FALSE | TRUE | TRUE |
| **558** | O95573 | Long-chain-fatty-acid--CoA ligase 3 | ER | -0.30 | 33.65 | FALSE | TRUE | TRUE |
| **559** | O00154 | Cytosolic acyl coenzyme A thioeste | CYTOSOL | 17.59 | 14.77 | FALSE | TRUE | TRUE |
| **560** | P12429 | Annexin A3 | unknown | 14.81 | 18.94 | FALSE | TRUE | TRUE |
| **561** | P00492 | Hypoxanthine-guanine phosphoribo | CYTOSOL | 21.41 | 23.77 | FALSE | TRUE | TRUE |
| **562** | P08758 | Annexin A5 | unknown | 13.52 | 18.40 | FALSE | TRUE | TRUE |
| **563** | Q14527 | Helicase-like transcription factor | unknown | -21.29 | -20.60 | FALSE | TRUE | TRUE |
| **564** | O14980 | Exportin-1 | unknown | 21.18 | 11.69 | FALSE | TRUE | TRUE |
| **565** | Q13155 | Aminoacyl tRNA synthase complex- | unknown | 5.70 | -3.26 | FALSE | TRUE | TRUE |
| **566** | Q8TCS8 | Polyribonucleotide nucleotidyltrans | MITOCHONDRIA | 34.82 | -11.15 | FALSE | TRUE | TRUE |

| | | | | | | | | |
|---|---|---|---|---|---|---|---|---|
| **567** | P62316 | Small nuclear ribonucleoprotein Sm | NUCLEUS | 5.09 | -34.95 | FALSE | TRUE | TRUE |
| **568** | P43487 | Ran-specific GTPase-activating prot | unknown | 13.87 | 23.69 | FALSE | TRUE | TRUE |
| **569** | Q8N1F7 | Nuclear pore complex protein Nup9 | NUCLEUS | -21.16 | -29.53 | FALSE | TRUE | TRUE |
| **570** | P62318 | Small nuclear ribonucleoprotein Sm | NUCLEUS | 5.14 | -34.70 | TRUE | TRUE | TRUE |
| **571** | Q96DI7 | U5 small nuclear ribonucleoprotein | NUCLEUS | 1.29 | -37.95 | FALSE | TRUE | TRUE |
| **572** | P00441 | Superoxide dismutase [Cu-Zn] | unknown | 16.00 | 19.22 | FALSE | TRUE | TRUE |
| **573** | Q9P035 | Very-long-chain (3R)-3-hydroxyacy | ER | 1.20 | 33.84 | FALSE | TRUE | TRUE |
| **574** | P37837 | Transaldolase | CYTOSOL | 16.76 | 21.49 | FALSE | TRUE | TRUE |
| **575** | P25789 | Proteasome subunit alpha type-4 | PROTEASOME | 21.61 | 6.43 | FALSE | TRUE | TRUE |
| **576** | P16152 | Carbonyl reductase [NADPH] 1 | CYTOSOL | 15.17 | 12.16 | FALSE | TRUE | TRUE |
| **577** | Q86VP6 | Cullin-associated NEDD8-dissociate | unknown | 9.97 | 1.63 | FALSE | TRUE | TRUE |
| **578** | O75436 | Vacuolar protein sorting-associated | unknown | -14.61 | 8.82 | FALSE | TRUE | TRUE |
| **579** | Q9NTJ3 | Structural maintenance of chromos | unknown | -7.69 | -9.02 | FALSE | TRUE | TRUE |
| **580** | Q13242 | Serine/arginine-rich splicing factor | NUCLEUS | -12.40 | -36.51 | FALSE | TRUE | TRUE |
| **581** | Q9Y3A5 | Ribosome maturation protein SBDS | unknown | -8.46 | -0.49 | FALSE | TRUE | TRUE |
| **582** | P17655 | Calpain-2 catalytic subunit | CYTOSOL | 19.21 | 21.49 | FALSE | TRUE | TRUE |
| **583** | Q14978 | Nucleolar and coiled-body phospho | NUCLEUS | -17.29 | -28.99 | TRUE | TRUE | TRUE |
| **584** | O00231 | 26S proteasome non-ATPase regul | PROTEASOME | 21.24 | 6.48 | FALSE | TRUE | TRUE |
| **585** | Q9Y399 | 28S ribosomal protein S2, mitochon | MITOCHONDRIA | 43.24 | -13.03 | FALSE | TRUE | TRUE |
| **586** | P51114 | Fragile X mental retardation syndro | unknown | 8.09 | -25.07 | FALSE | TRUE | TRUE |
| **587** | P61221 | ATP-binding cassette sub-family E r | unknown | 4.84 | -18.75 | FALSE | TRUE | TRUE |
| **588** | Q14141 | Septin-6 | unknown | -2.05 | 4.45 | FALSE | TRUE | TRUE |
| **589** | Q86V81 | THO complex subunit 4 | NUCLEUS | -3.40 | -40.14 | FALSE | TRUE | TRUE |
| **590** | P07384 | Calpain-1 catalytic subunit | CYTOSOL | 17.07 | 18.01 | FALSE | TRUE | TRUE |
| **591** | P36871 | Phosphoglucomutase-1 | CYTOSOL | 25.18 | 23.25 | FALSE | TRUE | TRUE |
| **592** | Q6UB35 | Monofunctional C1-tetrahydrofolat | MITOCHONDRIA | 35.53 | -14.19 | FALSE | TRUE | TRUE |
| **593** | Q02241 | Kinesin-like protein KIF23 | unknown | -13.68 | -11.12 | FALSE | TRUE | TRUE |
| **594** | Q06210 | Glutamine--fructose-6-phosphate a | unknown | 8.42 | -3.71 | FALSE | TRUE | TRUE |
| **595** | P30153 | Serine/threonine-protein phosphat | unknown | 14.14 | 12.10 | FALSE | TRUE | TRUE |
| **596** | P48047 | ATP synthase subunit O, mitochond | MITOCHONDRIA | 34.79 | -1.49 | FALSE | TRUE | TRUE |
| **597** | O75083 | WD repeat-containing protein 1 | unknown | 5.31 | 9.53 | FALSE | TRUE | TRUE |
| **598** | P42167 | Lamina-associated polypeptide 2, is | unknown | 23.58 | -12.73 | TRUE | TRUE | TRUE |
| **599** | Q8TCJ2 | Dolichyl-diphosphooligosaccharide- | ER | 3.43 | 35.58 | FALSE | TRUE | TRUE |
| **600** | P31689 | DnaJ homolog subfamily A membe | unknown | -4.93 | -7.47 | FALSE | TRUE | TRUE |
| **601** | P31942 | Heterogeneous nuclear ribonucleop | NUCLEUS | -13.58 | -36.48 | FALSE | TRUE | TRUE |
| **602** | Q8NBS9 | Thioredoxin domain-containing prot | ER | -1.25 | 38.95 | FALSE | TRUE | TRUE |
| **603** | Q8WY36 | HMG box transcription factor BBX | unknown | -23.93 | -21.73 | TRUE | TRUE | TRUE |
| **604** | P00374 | Dihydrofolate reductase | CYTOSOL | 16.28 | 17.12 | FALSE | TRUE | TRUE |

| | | | | | | | | |
|---|---|---|---|---|---|---|---|---|
| **605** | O43399 | Tumor protein D54 | unknown | 10.24 | 10.28 | TRUE | TRUE | TRUE |
| **606** | Q04446 | 1,4-alpha-glucan-branching enzyme | CYTOSOL | 23.31 | 19.83 | FALSE | TRUE | TRUE |
| **607** | P09972 | Fructose-bisphosphate aldolase C | unknown | 9.38 | 4.75 | FALSE | TRUE | TRUE |
| **608** | Q9H7B2 | Ribosome production factor 2 homo | NUCLEUS | -0.15 | -35.77 | FALSE | TRUE | TRUE |
| **609** | P61158 | Actin-related protein 3 | unknown | 1.65 | -14.22 | FALSE | TRUE | TRUE |
| **610** | Q14562 | ATP-dependent RNA helicase DHX8 | NUCLEUS | -4.35 | -38.66 | FALSE | TRUE | TRUE |
| **611** | P43897 | Elongation factor Ts, mitochondrial | MITOCHONDRIA | 44.04 | -13.28 | FALSE | TRUE | TRUE |
| **612** | P62834 | Ras-related protein Rap-1A | PM | -24.96 | 15.33 | FALSE | TRUE | FALSE |
| **613** | P53992 | Protein transport protein Sec24C | unknown | -10.82 | 7.52 | FALSE | TRUE | TRUE |
| **614** | P05166 | Propionyl-CoA carboxylase beta cha | MITOCHONDRIA | 37.35 | -9.66 | FALSE | TRUE | TRUE |
| **615** | Q99829 | Copine-1 | unknown | 11.97 | 16.57 | FALSE | TRUE | TRUE |
| **616** | Q9BQ67 | Glutamate-rich WD repeat-containi | NUCLEUS | -10.38 | -29.83 | FALSE | TRUE | TRUE |
| **617** | P17980 | 26S proteasome regulatory subunit | PROTEASOME | 20.81 | 6.93 | TRUE | TRUE | TRUE |
| **618** | Q8WYP5 | Protein ELYS | unknown | -20.53 | -15.65 | TRUE | TRUE | TRUE |
| **619** | Q9NW13 | RNA-binding protein 28 | NUCLEUS | -1.28 | -39.78 | FALSE | TRUE | TRUE |
| **620** | P54577 | Tyrosine--tRNA ligase, cytoplasmic | unknown | 16.32 | 3.37 | FALSE | TRUE | TRUE |
| **621** | Q99460 | 26S proteasome non-ATPase regula | PROTEASOME | 21.40 | 6.54 | FALSE | TRUE | TRUE |
| **622** | Q9NSD9 | Phenylalanine--tRNA ligase beta su | unknown | -2.95 | -11.71 | FALSE | TRUE | TRUE |
| **623** | Q99613 | Eukaryotic translation initiation fact | unknown | 13.69 | -10.96 | FALSE | TRUE | TRUE |
| **624** | Q53H96 | Pyrroline-5-carboxylate reductase 3 | unknown | 0.11 | -4.42 | FALSE | TRUE | TRUE |
| **625** | P55809 | Succinyl-CoA:3-ketoacid coenzyme | MITOCHONDRIA | 36.79 | -4.71 | FALSE | TRUE | TRUE |
| **626** | Q9NX24 | H/ACA ribonucleoprotein complex s | NUCLEUS | -4.61 | -36.98 | FALSE | TRUE | TRUE |
| **627** | Q13427 | Peptidyl-prolyl cis-trans isomerase ( | NUCLEUS | -5.65 | -31.39 | FALSE | TRUE | TRUE |
| **628** | P43490 | Nicotinamide phosphoribosyltransfe | unknown | 8.09 | -2.23 | FALSE | TRUE | TRUE |
| **629** | Q3YEC7 | Rab-like protein 6 | unknown | 15.86 | 13.67 | TRUE | TRUE | TRUE |
| **630** | P43034 | Platelet-activating factor acetylhyd | unknown | 9.22 | -2.29 | FALSE | TRUE | TRUE |
| **631** | Q16531 | DNA damage-binding protein 1 | NUCLEUS | -15.33 | -29.11 | FALSE | TRUE | TRUE |
| **632** | P23381 | Tryptophan--tRNA ligase, cytoplasm | CYTOSOL | 21.79 | 22.36 | FALSE | TRUE | TRUE |
| **633** | O15160 | DNA-directed RNA polymerases I ar | NUCLEUS | -15.83 | -28.66 | FALSE | TRUE | TRUE |
| **634** | Q13619 | Cullin-4A | NUCLEUS | -14.64 | -28.40 | FALSE | TRUE | TRUE |
| **635** | Q8IWS0 | PHD finger protein 6 | unknown | -14.76 | -20.53 | FALSE | TRUE | TRUE |
| **636** | Q9Y4L1 | Hypoxia up-regulated protein 1 | ER | -1.18 | 39.27 | FALSE | TRUE | TRUE |
| **637** | Q53GS9 | U4/U6.U5 tri-snRNP-associated pro | NUCLEUS | -2.57 | -31.51 | FALSE | TRUE | TRUE |
| **638** | O43592 | Exportin-T | unknown | 19.34 | 12.54 | FALSE | TRUE | TRUE |
| **639** | O14929 | Histone acetyltransferase type B ca | unknown | 27.36 | 22.83 | FALSE | TRUE | TRUE |
| **640** | Q16851 | UTP--glucose-1-phosphate uridylylt | unknown | 9.76 | -13.07 | FALSE | TRUE | TRUE |
| **641** | Q9BQ52 | Zinc phosphodiesterase ELAC prote | unknown | 21.00 | -3.61 | FALSE | TRUE | TRUE |
| **642** | P30040 | Endoplasmic reticulum resident pro | ER | -4.32 | 37.06 | FALSE | TRUE | TRUE |

| | | | | | | | | |
|---|---|---|---|---|---|---|---|---|
| **643** | P63151 | Serine/threonine-protein phosphata | unknown | 15.88 | 6.46 | FALSE | TRUE | TRUE |
| **644** | P63173 | 60S ribosomal protein L38 | RIBOSOME 60S | 15.63 | -22.97 | FALSE | TRUE | TRUE |
| **645** | P26006 | Integrin alpha-3 | PM | -28.41 | 8.06 | FALSE | TRUE | TRUE |
| **646** | O75844 | CAAX prenyl protease 1 homolog | unknown | -8.08 | 29.37 | FALSE | TRUE | TRUE |
| **647** | Q9Y678 | Coatomer subunit gamma-1 | unknown | 10.20 | 4.88 | FALSE | TRUE | TRUE |
| **648** | O43172 | U4/U6 small nuclear ribonucleoprot | NUCLEUS | 3.12 | -34.82 | TRUE | TRUE | TRUE |
| **649** | P49748 | Very long-chain specific acyl-CoA de | MITOCHONDRIA | 42.52 | -11.99 | FALSE | TRUE | TRUE |
| **650** | Q08170 | Serine/arginine-rich splicing factor 4 | unknown | -12.09 | -31.39 | FALSE | TRUE | FALSE |
| **651** | Q13148 | TAR DNA-binding protein 43 | NUCLEUS | -20.21 | -32.96 | TRUE | TRUE | TRUE |
| **652** | Q8WUM4 | Programmed cell death 6-interactin | unknown | 9.30 | 13.60 | FALSE | TRUE | TRUE |
| **653** | Q9UBM7 | 7-dehydrocholesterol reductase | ER | -11.51 | 20.76 | FALSE | TRUE | TRUE |
| **654** | P48444 | Coatomer subunit delta | unknown | 10.22 | 3.81 | TRUE | TRUE | TRUE |
| **655** | Q16543 | Hsp90 co-chaperone Cdc37 | CYTOSOL | 18.65 | 19.16 | FALSE | TRUE | TRUE |
| **656** | P56537 | Eukaryotic translation initiation fact | unknown | 14.62 | -11.50 | FALSE | TRUE | TRUE |
| **657** | P07339 | Cathepsin D | LYSOSOME | -40.54 | 8.94 | FALSE | TRUE | TRUE |
| **658** | P42166 | Lamina-associated polypeptide 2, is | NUCLEUS-CHROMATIN | -19.72 | -14.81 | TRUE | TRUE | TRUE |
| **659** | P28074 | Proteasome subunit beta type-5 | PROTEASOME | 22.43 | 6.36 | FALSE | TRUE | TRUE |
| **660** | P49189 | 4-trimethylaminobutyraldehyde de | unknown | 20.27 | -3.13 | FALSE | TRUE | TRUE |
| **661** | Q13547 | Histone deacetylase 1 | NUCLEUS | -25.88 | -26.91 | FALSE | TRUE | FALSE |
| **662** | Q92945 | Far upstream element-binding prot | NUCLEUS | -13.96 | -31.73 | TRUE | TRUE | TRUE |
| **663** | Q15436 | Protein transport protein Sec23A | unknown | -10.85 | 7.49 | FALSE | TRUE | TRUE |
| **664** | Q99798 | Aconitate hydratase, mitochondrial | MITOCHONDRIA | 39.01 | -7.48 | FALSE | TRUE | TRUE |
| **665** | O95478 | Ribosome biogenesis protein NSA2 | NUCLEUS | 1.62 | -34.83 | FALSE | TRUE | TRUE |
| **666** | Q07666 | KH domain-containing, RNA-binding | NUCLEUS | -10.82 | -36.47 | FALSE | TRUE | TRUE |
| **667** | P52434 | DNA-directed RNA polymerases I, II | NUCLEUS | -15.89 | -29.45 | FALSE | TRUE | TRUE |
| **668** | O75533 | Splicing factor 3B subunit 1 | NUCLEUS | 4.45 | -35.17 | TRUE | TRUE | TRUE |
| **669** | Q9UJS0 | Calcium-binding mitochondrial carri | MITOCHONDRIA | 35.89 | -15.14 | FALSE | TRUE | TRUE |
| **670** | O75340 | Programmed cell death protein 6 | unknown | 12.56 | 14.85 | FALSE | TRUE | TRUE |
| **671** | Q02809 | Procollagen-lysine,2-oxoglutarate 5 | ER | 4.19 | 38.91 | FALSE | TRUE | TRUE |
| **672** | O60568 | Multifunctional procollagen lysine h | ER | 4.57 | 39.08 | FALSE | TRUE | TRUE |
| **673** | P16949 | Stathmin | unknown | 20.50 | 24.76 | TRUE | TRUE | TRUE |
| **674** | P07954 | Fumarate hydratase, mitochondrial | MITOCHONDRIA | 38.38 | -4.90 | FALSE | TRUE | TRUE |
| **675** | Q969Z0 | FAST kinase domain-containing pro | MITOCHONDRIA | 39.99 | -10.24 | FALSE | TRUE | TRUE |
| **676** | Q12792 | Twinfilin-1 | unknown | 11.78 | 9.89 | TRUE | TRUE | TRUE |
| **677** | P11766 | Alcohol dehydrogenase class-3 | CYTOSOL | 25.01 | 21.94 | FALSE | TRUE | TRUE |
| **678** | Q9H2U1 | ATP-dependent DNA/RNA helicase | NUCLEUS | -0.91 | -22.78 | FALSE | TRUE | TRUE |
| **679** | P54136 | Arginine--tRNA ligase, cytoplasmic | unknown | 5.71 | -3.26 | FALSE | TRUE | TRUE |
| **680** | P19367 | Hexokinase-1 | unknown | 26.64 | -13.40 | FALSE | TRUE | TRUE |

| | | | | | | | | |
|---|---|---|---|---|---|---|---|---|
| **681** | P11166 | Solute carrier family 2, facilitated gl | PM | -21.78 | 11.34 | FALSE | TRUE | TRUE |
| **682** | O60231 | Pre-mRNA-splicing factor ATP-depe | NUCLEUS | -1.83 | -24.50 | FALSE | TRUE | TRUE |
| **683** | Q12873 | Chromodomain-helicase-DNA-bindi | NUCLEUS | -23.86 | -24.75 | FALSE | TRUE | TRUE |
| **684** | Q9Y262 | Eukaryotic translation initiation fact | unknown | 13.67 | -10.95 | FALSE | TRUE | TRUE |
| **685** | O00116 | Alkyldihydroxyacetonephosphate s | PEROXISOME | 52.45 | -3.44 | FALSE | TRUE | TRUE |
| **686** | Q15691 | Microtubule-associated protein RP/ | unknown | 2.23 | -2.33 | TRUE | TRUE | TRUE |
| **687** | P28340 | DNA polymerase delta catalytic sub | unknown | 15.86 | 2.94 | FALSE | TRUE | TRUE |
| **688** | O15260 | Surfeit locus protein 4 | unknown | -6.46 | 32.15 | FALSE | TRUE | TRUE |
| **689** | P30405 | Peptidyl-prolyl cis-trans isomerase I | MITOCHONDRIA | 41.08 | -4.24 | FALSE | TRUE | TRUE |
| **690** | P21980 | Protein-glutamine gamma-glutamy | unknown | 12.78 | 7.67 | FALSE | TRUE | TRUE |
| **691** | Q15424 | Scaffold attachment factor B1 | NUCLEUS | 2.07 | -38.35 | TRUE | TRUE | TRUE |
| **692** | Q14166 | Tubulin--tyrosine ligase-like protein | CYTOSOL | 18.40 | 17.43 | TRUE | TRUE | TRUE |
| **693** | O75347 | Tubulin-specific chaperone A | unknown | 14.90 | 16.83 | FALSE | TRUE | TRUE |
| **694** | Q8TA86 | Retinitis pigmentosa 9 protein | unknown | 6.98 | -10.84 | TRUE | TRUE | TRUE |
| **695** | Q9H6R4 | Nucleolar protein 6 | NUCLEUS | -1.08 | -40.51 | TRUE | TRUE | TRUE |
| **696** | Q7L0Y3 | tRNA methyltransferase 10 homolo | MITOCHONDRIA | 42.63 | -8.43 | FALSE | TRUE | TRUE |
| **697** | P12277 | Creatine kinase B-type | CYTOSOL | 22.42 | 23.13 | FALSE | TRUE | TRUE |
| **698** | P46013 | Proliferation marker protein Ki-67 | unknown | -21.92 | -12.83 | TRUE | TRUE | TRUE |
| **699** | Q9H4A4 | Aminopeptidase B | unknown | 24.07 | 23.54 | FALSE | TRUE | TRUE |
| **700** | P30876 | DNA-directed RNA polymerase II su | NUCLEUS | -15.61 | -29.04 | FALSE | TRUE | TRUE |
| **701** | Q9BR76 | Coronin-1B | unknown | 5.76 | 7.43 | FALSE | TRUE | TRUE |
| **702** | P60228 | Eukaryotic translation initiation fact | unknown | 13.66 | -10.91 | FALSE | TRUE | TRUE |
| **703** | P35606 | Coatomer subunit beta' | unknown | 10.00 | 5.09 | FALSE | TRUE | TRUE |
| **704** | Q13404 | Ubiquitin-conjugating enzyme E2 va | unknown | 16.81 | 15.90 | FALSE | TRUE | TRUE |
| **705** | P40763 | Signal transducer and activator of tr | NUCLEUS | 1.88 | -2.89 | TRUE | TRUE | TRUE |
| **706** | P99999 | Cytochrome c | unknown | 27.22 | -13.03 | FALSE | TRUE | TRUE |
| **707** | P53597 | Succinate--CoA ligase [ADP/GDP-fo | MITOCHONDRIA | 40.80 | -5.43 | FALSE | TRUE | TRUE |
| **708** | P39748 | Flap endonuclease 1 | unknown | -20.95 | -21.50 | TRUE | TRUE | TRUE |
| **709** | P62195 | 26S proteasome regulatory subunit | PROTEASOME | 20.40 | 6.06 | TRUE | TRUE | TRUE |
| **710** | Q9HD20 | Manganese-transporting ATPase 1 | unknown | 3.66 | 34.87 | FALSE | TRUE | TRUE |
| **711** | Q9NTK5 | Obg-like ATPase 1 | CYTOSOL | 23.03 | 21.49 | FALSE | TRUE | TRUE |
| **712** | Q13724 | Mannosyl-oligosaccharide glucosid | ER | 4.58 | 36.42 | FALSE | TRUE | TRUE |
| **713** | P30084 | Enoyl-CoA hydratase, mitochondria | MITOCHONDRIA | 37.43 | -9.69 | FALSE | TRUE | TRUE |
| **714** | P61088 | Ubiquitin-conjugating enzyme E2 N | CYTOSOL | 19.94 | 19.44 | FALSE | TRUE | TRUE |
| **715** | P13804 | Electron transfer flavoprotein subur | MITOCHONDRIA | 39.29 | -9.73 | FALSE | TRUE | TRUE |
| **716** | Q4G0J3 | La-related protein 7 | NUCLEUS | 0.36 | -26.70 | FALSE | TRUE | TRUE |
| **717** | P27816 | Microtubule-associated protein 4 | unknown | -1.56 | -12.91 | TRUE | TRUE | TRUE |
| **718** | P27816 | Microtubule-associated protein 4 | unknown | -7.85 | -9.43 | TRUE | TRUE | TRUE |

| | | | | | | | | |
|---|---|---|---|---|---|---|---|---|
| **719** | O43491 | Band 4.1-like protein 2 | unknown | -27.66 | -0.03 | TRUE | TRUE | TRUE |
| **720** | O43491 | Band 4.1-like protein 2 | unknown | -27.53 | 1.57 | TRUE | TRUE | TRUE |
| **721** | P24941 | Cyclin-dependent kinase 2 | NUCLEUS | -4.37 | -9.78 | FALSE | TRUE | TRUE |
| **722** | P40121 | Macrophage-capping protein | CYTOSOL | 19.52 | 21.63 | FALSE | TRUE | TRUE |
| **723** | P61289 | Proteasome activator complex subu | unknown | 27.62 | 22.44 | TRUE | TRUE | TRUE |
| **724** | O15213 | WD repeat-containing protein 46 | NUCLEUS | -1.94 | -40.85 | FALSE | TRUE | TRUE |
| **725** | Q9P275 | Ubiquitin carboxyl-terminal hydrola | NUCLEUS | -0.39 | -35.91 | TRUE | TRUE | TRUE |
| **726** | Q9NS69 | Mitochondrial import receptor subu | MITOCHONDRIA | 32.22 | -15.60 | FALSE | TRUE | TRUE |
| **727** | Q14137 | Ribosome biogenesis protein BOP1 | NUCLEUS | -1.61 | -38.01 | FALSE | TRUE | TRUE |
| **728** | Q8WWQ0 | PH-interacting protein | unknown | -21.69 | -13.63 | TRUE | TRUE | TRUE |
| **729** | P63010 | AP-2 complex subunit beta | unknown | -10.30 | 6.46 | FALSE | TRUE | TRUE |
| **730** | Q9NQR4 | Omega-amidase NIT2 | unknown | 9.70 | 8.99 | FALSE | TRUE | TRUE |
| **731** | Q14444 | Caprin-1 | RIBOSOME 40S | 8.23 | -23.15 | TRUE | TRUE | TRUE |
| **732** | P42765 | 3-ketoacyl-CoA thiolase, mitochond | MITOCHONDRIA | 39.41 | -7.52 | FALSE | TRUE | TRUE |
| **733** | P54709 | Sodium/potassium-transporting AT | unknown | -21.19 | 19.64 | FALSE | TRUE | FALSE |
| **734** | Q9NWT1 | p21-activated protein kinase-intera | NUCLEUS | -0.66 | -37.39 | FALSE | TRUE | TRUE |
| **735** | Q96AC1 | Fermitin family homolog 2 | unknown | -13.76 | 14.47 | TRUE | TRUE | TRUE |
| **736** | O00541 | Pescadillo homolog | NUCLEUS | 2.22 | -36.66 | TRUE | TRUE | TRUE |
| **737** | P41227 | N-alpha-acetyltransferase 10 | unknown | 10.04 | -13.02 | FALSE | TRUE | TRUE |
| **738** | Q02218 | 2-oxoglutarate dehydrogenase, mit | MITOCHONDRIA | 39.34 | -7.23 | FALSE | TRUE | TRUE |
| **739** | P62191 | 26S proteasome regulatory subunit | PROTEASOME | 21.12 | 6.43 | FALSE | TRUE | TRUE |
| **740** | Q96KR1 | Zinc finger RNA-binding protein | NUCLEUS | -11.33 | -35.88 | TRUE | TRUE | TRUE |
| **741** | Q9UK76 | Jupiter microtubule associated hom | unknown | 10.01 | 21.20 | TRUE | TRUE | TRUE |
| **742** | Q14151 | Scaffold attachment factor B2 | NUCLEUS | 1.94 | -38.58 | TRUE | TRUE | TRUE |
| **743** | P83881 | 60S ribosomal protein L36a | RIBOSOME 60S | 16.37 | -22.48 | TRUE | FALSE | TRUE |
| **744** | P23588 | Eukaryotic translation initiation fact | unknown | 12.31 | -2.84 | TRUE | TRUE | TRUE |
| **745** | Q9H3K6 | BolA-like protein 2 | unknown | 19.65 | 21.72 | FALSE | TRUE | TRUE |
| **746** | Q1KMD3 | Heterogeneous nuclear ribonucleop | NUCLEUS | -13.31 | -30.51 | FALSE | TRUE | TRUE |
| **747** | P49792 | E3 SUMO-protein ligase RanBP2 | unknown | -6.93 | -23.19 | TRUE | TRUE | TRUE |
| **748** | Q9UKV8 | Protein argonaute-2 | unknown | 6.95 | -23.07 | FALSE | TRUE | TRUE |
| **749** | A1L0T0 | Acetolactate synthase-like protein | unknown | -7.60 | 30.90 | FALSE | TRUE | TRUE |
| **750** | P37108 | Signal recognition particle 14 kDa p | NUCLEUS | 9.16 | -6.76 | TRUE | TRUE | TRUE |
| **751** | P24752 | Acetyl-CoA acetyltransferase, mito | MITOCHONDRIA | 43.29 | -8.29 | FALSE | TRUE | TRUE |
| **752** | P28072 | Proteasome subunit beta type-6 | PROTEASOME | 22.72 | 6.31 | FALSE | TRUE | TRUE |
| **753** | P30085 | UMP-CMP kinase | CYTOSOL | 20.17 | 21.01 | FALSE | TRUE | TRUE |
| **754** | Q9Y5M8 | Signal recognition particle receptor | ER | 3.17 | 35.09 | FALSE | TRUE | TRUE |
| **755** | Q9UQE7 | Structural maintenance of chromos | NUCLEUS-CHROMATIN | -18.13 | -15.95 | FALSE | TRUE | TRUE |
| **756** | Q13177 | Serine/threonine-protein kinase PA | unknown | 15.96 | 10.40 | TRUE | TRUE | TRUE |

| | | | | | | | | |
|---|---|---|---|---|---|---|---|---|
| **757** | P60981 | Destrin | unknown | 4.14 | 9.37 | FALSE | TRUE | TRUE |
| **758** | Q99805 | Transmembrane 9 superfamily mer | unknown | -22.33 | 30.18 | FALSE | TRUE | TRUE |
| **759** | Q96SB4 | SRSF protein kinase 1 | unknown | 6.52 | -24.49 | FALSE | TRUE | TRUE |
| **760** | P68032 | Actin, alpha cardiac muscle 1 | unknown | 5.36 | 7.81 | FALSE | FALSE | TRUE |
| **761** | Q8IWA0 | WD repeat-containing protein 75 | NUCLEUS | -2.57 | -40.99 | FALSE | TRUE | TRUE |
| **762** | P51610 | Host cell factor 1 | NUCLEUS | -14.15 | -22.44 | TRUE | TRUE | TRUE |
| **763** | Q92900 | Regulator of nonsense transcripts 1 | NUCLEUS | -2.07 | -14.06 | FALSE | TRUE | TRUE |
| **764** | O95340 | Bifunctional 3'-phosphoadenosine 5 | unknown | 8.48 | -3.98 | FALSE | TRUE | TRUE |
| **765** | O14737 | Programmed cell death protein 5 | CYTOSOL | 22.67 | 23.31 | FALSE | TRUE | TRUE |
| **766** | Q9BUN8 | Derlin-1 | unknown | -10.73 | 27.71 | FALSE | TRUE | TRUE |
| **767** | P61081 | NEDD8-conjugating enzyme Ubc12 | CYTOSOL | 15.51 | 23.01 | TRUE | TRUE | TRUE |
| **768** | Q9NZJ7 | Mitochondrial carrier homolog 1 | MITOCHONDRIA | 29.40 | -10.12 | FALSE | TRUE | TRUE |
| **769** | P11279 | Lysosome-associated membrane g | LYSOSOME | -39.03 | 8.59 | FALSE | TRUE | TRUE |
| **770** | P55769 | NHP2-like protein 1 | NUCLEUS | -3.56 | -40.70 | FALSE | TRUE | TRUE |
| **771** | Q15758 | Neutral amino acid transporter B(0) | PM | -24.23 | 10.31 | TRUE | TRUE | TRUE |
| **772** | P06737 | Glycogen phosphorylase, liver form | unknown | 18.18 | 18.25 | FALSE | TRUE | FALSE |
| **773** | P45974 | Ubiquitin carboxyl-terminal hydrola | CYTOSOL | 23.65 | 20.42 | FALSE | TRUE | TRUE |
| **774** | P41567 | Eukaryotic translation initiation fact | NUCLEUS | -3.61 | -16.51 | FALSE | TRUE | FALSE |
| **775** | P25788 | Proteasome subunit alpha type-3 | PROTEASOME | 22.48 | 6.56 | FALSE | TRUE | TRUE |
| **776** | Q14258 | E3 ubiquitin/ISG15 ligase TRIM25 | unknown | 8.15 | -20.79 | TRUE | TRUE | TRUE |
| **777** | Q8TEQ6 | Gem-associated protein 5 | unknown | 13.45 | 0.86 | TRUE | TRUE | TRUE |
| **778** | Q3ZCQ8 | Mitochondrial import inner membra | MITOCHONDRIA | 35.43 | -9.00 | FALSE | TRUE | TRUE |
| **779** | Q93009 | Ubiquitin carboxyl-terminal hydrola | unknown | 12.25 | 3.45 | FALSE | TRUE | TRUE |
| **780** | P25786 | Proteasome subunit alpha type-1 | PROTEASOME | 22.61 | 5.88 | FALSE | TRUE | TRUE |
| **781** | Q8N3C0 | Activating signal cointegrator 1 com | unknown | 1.23 | -15.60 | FALSE | TRUE | TRUE |
| **782** | P84095 | Rho-related GTP-binding protein Rh | PM | -22.68 | 10.59 | FALSE | TRUE | TRUE |
| **783** | P48729 | Casein kinase I isoform alpha | unknown | 1.97 | -10.93 | TRUE | TRUE | TRUE |
| **784** | Q5JTH9 | RRP12-like protein | NUCLEUS | -0.81 | -40.23 | FALSE | TRUE | TRUE |
| **785** | O00487 | 26S proteasome non-ATPase regula | PROTEASOME | 20.28 | 6.71 | FALSE | TRUE | TRUE |
| **786** | Q8NI27 | THO complex subunit 2 | unknown | -10.98 | -22.75 | TRUE | TRUE | TRUE |
| **787** | P09622 | Dihydrolipoyl dehydrogenase, mito | MITOCHONDRIA | 39.74 | -7.37 | FALSE | TRUE | TRUE |
| **788** | P46060 | Ran GTPase-activating protein 1 | NUCLEUS | -19.36 | -29.76 | TRUE | TRUE | TRUE |
| **789** | P15559 | NAD(P)H dehydrogenase [quinone] | unknown | 17.47 | 7.31 | FALSE | TRUE | FALSE |
| **790** | P28288 | ATP-binding cassette sub-family D r | PEROXISOME | 52.56 | -3.19 | FALSE | TRUE | TRUE |
| **791** | Q9BYG3 | MKI67 FHA domain-interacting nuc | NUCLEUS | 0.92 | -36.78 | TRUE | TRUE | TRUE |
| **792** | O00264 | Membrane-associated progesteror | ER | -11.05 | 27.95 | FALSE | TRUE | TRUE |
| **793** | Q9NUU7 | ATP-dependent RNA helicase DDX1 | unknown | 11.75 | 0.38 | FALSE | TRUE | TRUE |
| **794** | O60664 | Perilipin-3 | CYTOSOL | 18.65 | 15.87 | TRUE | TRUE | TRUE |

| | | | | | | | | |
|---|---|---|---|---|---|---|---|---|
| **795** | P47813 | Eukaryotic translation initiation fact | unknown | 13.71 | -11.07 | FALSE | TRUE | TRUE |
| **796** | Q9BSC4 | Nucleolar protein 10 | NUCLEUS | 0.47 | -39.29 | FALSE | TRUE | TRUE |
| **797** | P54819 | Adenylate kinase 2, mitochondrial | MITOCHONDRIA | 38.77 | -5.66 | FALSE | TRUE | TRUE |
| **798** | Q8TAT6 | Nuclear protein localization protein | NUCLEUS | -3.39 | -9.78 | FALSE | TRUE | TRUE |
| **799** | Q92804 | TATA-binding protein-associated fa | NUCLEUS | -9.00 | -35.87 | TRUE | TRUE | TRUE |
| **800** | Q15437 | Protein transport protein Sec23B | unknown | 4.96 | -9.98 | FALSE | TRUE | TRUE |
| **801** | Q13423 | NAD(P) transhydrogenase, mitocho | MITOCHONDRIA | 36.22 | -8.75 | FALSE | TRUE | TRUE |
| **802** | Q6DKI1 | 60S ribosomal protein L7-like 1 | NUCLEUS | -0.39 | -37.37 | FALSE | TRUE | FALSE |
| **803** | P42696 | RNA-binding protein 34 | NUCLEUS | -0.93 | -35.29 | FALSE | TRUE | TRUE |
| **804** | Q9NV06 | DDB1- and CUL4-associated factor 1 | NUCLEUS | -1.74 | -38.03 | FALSE | TRUE | TRUE |
| **805** | P08240 | Signal recognition particle receptor | ER | 3.51 | 36.60 | TRUE | TRUE | TRUE |
| **806** | Q15269 | Periodic tryptophan protein 2 homo | NUCLEUS | -2.76 | -39.98 | FALSE | TRUE | TRUE |
| **807** | Q96PZ0 | Pseudouridylate synthase 7 homolo | unknown | 15.74 | 13.36 | FALSE | TRUE | TRUE |
| **808** | P51532 | Transcription activator BRG1 | unknown | -25.74 | -23.52 | TRUE | TRUE | TRUE |
| **809** | P49257 | Protein ERGIC-53 | unknown | -10.65 | 29.16 | FALSE | TRUE | TRUE |
| **810** | Q9Y6M5 | Zinc transporter 1 | PM | -25.15 | 14.56 | TRUE | TRUE | TRUE |
| **811** | Q9UM00 | Calcium load-activated calcium cha | ER | 3.02 | 36.16 | FALSE | TRUE | TRUE |
| **812** | P21281 | V-type proton ATPase subunit B, bra | unknown | -30.59 | 3.27 | FALSE | TRUE | TRUE |
| **813** | Q5T4S7 | E3 ubiquitin-protein ligase UBR4 | unknown | 10.67 | 2.85 | FALSE | TRUE | TRUE |
| **814** | O43615 | Mitochondrial import inner membra | MITOCHONDRIA | 39.97 | -8.96 | FALSE | TRUE | TRUE |
| **815** | P49591 | Serine--tRNA ligase, cytoplasmic | CYTOSOL | 21.85 | 18.79 | TRUE | TRUE | TRUE |
| **816** | O95782 | AP-2 complex subunit alpha-1 | unknown | -10.31 | 6.46 | FALSE | TRUE | TRUE |
| **817** | Q9UHD1 | Cysteine and histidine-rich domain- | CYTOSOL | 21.98 | 18.14 | FALSE | TRUE | TRUE |
| **818** | P62333 | 26S proteasome regulatory subunit | PROTEASOME | 20.19 | 6.80 | FALSE | TRUE | TRUE |
| **819** | Q9Y2T2 | AP-3 complex subunit mu-1 | unknown | -14.26 | 4.08 | FALSE | TRUE | TRUE |
| **820** | Q13620 | Cullin-4B | NUCLEUS | -15.44 | -29.19 | FALSE | TRUE | TRUE |
| **821** | P62304 | Small nuclear ribonucleoprotein E | NUCLEUS | 5.31 | -35.80 | TRUE | TRUE | TRUE |
| **822** | Q9GZZ1 | N-alpha-acetyltransferase 50 | unknown | 9.31 | -13.26 | FALSE | TRUE | TRUE |
| **823** | O75439 | Mitochondrial-processing peptidase | MITOCHONDRIA | 40.85 | -10.39 | FALSE | TRUE | TRUE |
| **824** | Q7L2H7 | Eukaryotic translation initiation fact | unknown | 13.18 | -10.93 | FALSE | TRUE | TRUE |
| **825** | P48147 | Prolyl endopeptidase | unknown | 24.79 | 22.67 | FALSE | TRUE | TRUE |
| **826** | Q15003 | Condensin complex subunit 2 | unknown | -7.95 | -9.04 | TRUE | TRUE | TRUE |
| **827** | Q9BZE1 | 39S ribosomal protein L37, mitocho | MITOCHONDRIA | 42.97 | -12.90 | FALSE | TRUE | TRUE |
| **828** | P55735 | Protein SEC13 homolog | NUCLEUS | 9.10 | -6.87 | FALSE | TRUE | TRUE |
| **829** | Q8TEM1 | Nuclear pore membrane glycoprote | unknown | 24.05 | -11.48 | FALSE | TRUE | TRUE |
| **830** | Q9HCC0 | Methylcrotonoyl-CoA carboxylase b | MITOCHONDRIA | 36.77 | -4.39 | FALSE | TRUE | TRUE |
| **831** | P05455 | Lupus La protein | unknown | 27.29 | 22.99 | TRUE | TRUE | TRUE |
| **832** | Q2TAY7 | WD40 repeat-containing protein SM | NUCLEUS | -3.99 | -38.39 | FALSE | TRUE | TRUE |

| | | | | | | | | |
|---|---|---|---|---|---|---|---|---|
| **833** | Q66PJ3 | ADP-ribosylation factor-like protein | unknown | 18.15 | -19.49 | FALSE | TRUE | TRUE |
| **834** | Q66PJ3 | ADP-ribosylation factor-like protein | unknown | 18.13 | -19.52 | FALSE | TRUE | TRUE |
| **835** | Q9UKD2 | mRNA turnover protein 4 homolog | NUCLEUS | 3.63 | -29.02 | FALSE | TRUE | TRUE |
| **836** | P46926 | Glucosamine-6-phosphate isomera | unknown | 23.95 | 19.73 | FALSE | TRUE | TRUE |
| **837** | P45973 | Chromobox protein homolog 5 | unknown | -20.13 | -18.08 | TRUE | TRUE | TRUE |
| **838** | O15446 | DNA-directed RNA polymerase I sul | NUCLEUS | -24.69 | -25.76 | FALSE | TRUE | TRUE |
| **839** | Q9H6S0 | 3'-5' RNA helicase YTHDC2 | unknown | 1.67 | -15.75 | FALSE | TRUE | TRUE |
| **840** | P78406 | mRNA export factor | NUCLEUS | -4.91 | -30.97 | FALSE | TRUE | TRUE |
| **841** | Q9BQA1 | Methylosome protein 50 | unknown | 11.24 | 18.90 | FALSE | TRUE | TRUE |
| **842** | Q16836 | Hydroxyacyl-coenzyme A dehydrog | MITOCHONDRIA | 43.55 | -6.65 | FALSE | TRUE | TRUE |
| **843** | P61077 | Ubiquitin-conjugating enzyme E2 D | unknown | 14.33 | 11.45 | FALSE | TRUE | FALSE |
| **844** | Q12904 | Aminoacyl tRNA synthase complex- | unknown | 5.60 | -3.32 | FALSE | TRUE | TRUE |
| **845** | P32322 | Pyrroline-5-carboxylate reductase 1 | MITOCHONDRIA | 35.40 | -14.27 | FALSE | TRUE | TRUE |
| **846** | Q9BRJ6 | Uncharacterized protein C7orf50 | unknown | 5.92 | -26.28 | FALSE | TRUE | TRUE |
| **847** | P51665 | 26S proteasome non-ATPase regula | PROTEASOME | 18.87 | 5.15 | FALSE | TRUE | TRUE |
| **848** | P12081 | Histidine--tRNA ligase, cytoplasmic | unknown | 13.51 | -1.86 | FALSE | TRUE | TRUE |
| **849** | O43852 | Calumenin | unknown | -1.11 | 40.52 | FALSE | TRUE | TRUE |
| **850** | O43852 | Calumenin | unknown | -0.73 | 40.81 | FALSE | TRUE | TRUE |
| **851** | P31350 | Ribonucleoside-diphosphate reduct | unknown | 16.26 | 16.13 | FALSE | TRUE | TRUE |
| **852** | Q04760 | Lactoylglutathione lyase | CYTOSOL | 23.48 | 24.51 | TRUE | TRUE | TRUE |
| **853** | O94906 | Pre-mRNA-processing factor 6 | NUCLEUS | 2.84 | -35.33 | FALSE | TRUE | TRUE |
| **854** | Q15392 | Delta(24)-sterol reductase | unknown | -5.11 | 33.36 | FALSE | TRUE | TRUE |
| **855** | Q03701 | CCAAT/enhancer-binding protein ze | NUCLEUS | 0.13 | -39.77 | TRUE | TRUE | TRUE |
| **856** | Q9Y295 | Developmentally-regulated GTP-bi | unknown | 3.90 | -17.45 | FALSE | TRUE | TRUE |
| **857** | Q9UIF9 | Bromodomain adjacent to zinc finge | unknown | -20.96 | -19.19 | TRUE | TRUE | TRUE |
| **858** | Q8N1G2 | Cap-specific mRNA (nucleoside-2'-C | NUCLEUS | -0.37 | -24.12 | FALSE | TRUE | TRUE |
| **859** | Q9NZ45 | CDGSH iron-sulfur domain-containi | unknown | 36.43 | -1.83 | FALSE | TRUE | TRUE |
| **860** | Q9H2U2 | Inorganic pyrophosphatase 2, mito | unknown | 45.16 | -5.87 | FALSE | TRUE | TRUE |
| **861** | O00232 | 26S proteasome non-ATPase regula | PROTEASOME | 20.61 | 5.79 | FALSE | TRUE | TRUE |
| **862** | P29144 | Tripeptidyl-peptidase 2 | unknown | 13.30 | -6.46 | FALSE | TRUE | TRUE |
| **863** | Q10713 | Mitochondrial-processing peptidase | MITOCHONDRIA | 39.25 | -8.85 | FALSE | TRUE | TRUE |
| **864** | P09661 | U2 small nuclear ribonucleoprotein | NUCLEUS | 3.83 | -35.99 | FALSE | TRUE | TRUE |
| **865** | O60493 | Sorting nexin-3 | unknown | -10.84 | 16.69 | TRUE | TRUE | TRUE |
| **866** | Q15293 | Reticulocalbin-1 | ER | -1.97 | 38.79 | FALSE | TRUE | TRUE |
| **867** | Q96KP4 | Cytosolic non-specific dipeptidase | CYTOSOL | 21.71 | 23.76 | FALSE | TRUE | TRUE |
| **868** | P54578 | Ubiquitin carboxyl-terminal hydrola | unknown | 23.37 | 21.90 | FALSE | TRUE | TRUE |
| **869** | P04818 | Thymidylate synthase | unknown | 7.85 | -6.36 | FALSE | TRUE | TRUE |
| **870** | Q9UKK9 | ADP-sugar pyrophosphatase | CYTOSOL | 23.72 | 19.99 | TRUE | TRUE | TRUE |

| | | | | | | | | |
|---|---|---|---|---|---|---|---|---|
| **871** | Q5VTL8 | Pre-mRNA-splicing factor 38B | NUCLEUS | 1.46 | -30.25 | FALSE | TRUE | TRUE |
| **872** | Q9UN86 | Ras GTPase-activating protein-bind | unknown | 9.38 | -20.82 | TRUE | TRUE | TRUE |
| **873** | P30419 | Glycylpeptide N-tetradecanoyltrans | unknown | 7.03 | -23.55 | TRUE | TRUE | TRUE |
| **874** | Q00534 | Cyclin-dependent kinase 6 | unknown | 19.09 | 13.54 | FALSE | TRUE | TRUE |
| **875** | Q15008 | 26S proteasome non-ATPase regula | PROTEASOME | 21.01 | 6.30 | FALSE | TRUE | TRUE |
| **876** | Q16630 | Cleavage and polyadenylation spec | NUCLEUS | 4.93 | -32.64 | FALSE | TRUE | TRUE |
| **877** | Q96EE3 | Nucleoporin SEH1 | NUCLEUS | -5.46 | -24.34 | FALSE | TRUE | TRUE |
| **878** | Q86X55 | Histone-arginine methyltransferase | unknown | 19.57 | 12.44 | FALSE | TRUE | TRUE |
| **879** | Q9P265 | Disco-interacting protein 2 homolog | unknown | -8.71 | 4.50 | TRUE | TRUE | TRUE |
| **880** | Q4G0N4 | NAD kinase 2, mitochondrial | MITOCHONDRIA | 33.96 | -6.18 | FALSE | TRUE | TRUE |
| **881** | P42285 | Exosome RNA helicase MTR4 | NUCLEUS | -11.16 | -26.18 | FALSE | TRUE | TRUE |
| **882** | Q07065 | Cytoskeleton-associated protein 4 | unknown | 5.12 | 38.50 | TRUE | TRUE | TRUE |
| **883** | P53582 | Methionine aminopeptidase 1 | unknown | -9.27 | -20.91 | FALSE | TRUE | TRUE |
| **884** | Q9BVK6 | Transmembrane emp24 domain-co | unknown | -10.06 | 32.53 | FALSE | TRUE | TRUE |
| **885** | Q15363 | Transmembrane emp24 domain-co | unknown | -3.85 | 31.62 | FALSE | TRUE | TRUE |
| **886** | P28070 | Proteasome subunit beta type-4 | PROTEASOME | 22.54 | 6.54 | FALSE | TRUE | TRUE |
| **887** | Q9Y305 | Acyl-coenzyme A thioesterase 9, m | MITOCHONDRIA | 36.45 | -10.64 | FALSE | TRUE | TRUE |
| **888** | Q96FW1 | Ubiquitin thioesterase OTUB1 | unknown | 25.38 | 21.84 | FALSE | TRUE | TRUE |
| **889** | P20020 | Plasma membrane calcium-transpo | PM | -24.76 | 8.89 | TRUE | TRUE | TRUE |
| **890** | Q9BXS5 | AP-1 complex subunit mu-1 | unknown | -33.33 | 19.56 | FALSE | TRUE | FALSE |
| **891** | Q9UBF2 | Coatomer subunit gamma-2 | unknown | 6.04 | 4.28 | FALSE | TRUE | TRUE |
| **892** | Q9Y2Z9 | Ubiquinone biosynthesis monooxyg | MITOCHONDRIA | 43.34 | -11.49 | FALSE | TRUE | FALSE |
| **893** | Q9NPE3 | H/ACA ribonucleoprotein complex s | NUCLEUS | -5.49 | -37.14 | FALSE | TRUE | FALSE |
| **894** | Q9Y5A9 | YTH domain-containing family prot | unknown | 5.92 | -23.67 | FALSE | TRUE | TRUE |
| **895** | P13073 | Cytochrome c oxidase subunit 4 iso | MITOCHONDRIA | 31.48 | -14.92 | FALSE | TRUE | TRUE |
| **896** | P78347 | General transcription factor II-I | NUCLEUS | -24.21 | -22.79 | TRUE | TRUE | TRUE |
| **897** | P25787 | Proteasome subunit alpha type-2 | PROTEASOME | 21.78 | 6.10 | FALSE | TRUE | TRUE |
| **898** | Q86UP2 | Kinectin | ER | 0.85 | 37.47 | FALSE | TRUE | TRUE |
| **899** | P19525 | Interferon-induced, double-strande | unknown | 6.95 | -19.98 | FALSE | TRUE | TRUE |
| **900** | P47755 | F-actin-capping protein subunit alph | unknown | 6.17 | 4.83 | FALSE | TRUE | TRUE |
| **901** | Q6DD88 | Atlastin-3 | unknown | -9.87 | 32.75 | FALSE | TRUE | TRUE |
| **902** | P82675 | 28S ribosomal protein S5, mitochon | MITOCHONDRIA | 41.62 | -12.74 | FALSE | TRUE | TRUE |
| **903** | O60884 | DnaJ homolog subfamily A membe | unknown | -4.77 | -7.34 | TRUE | TRUE | TRUE |
| **904** | Q9UNQ2 | Probable dimethyladenosine transf | NUCLEUS | 3.84 | -28.64 | FALSE | TRUE | TRUE |
| **905** | Q14157 | Ubiquitin-associated protein 2-like | unknown | 8.47 | -25.74 | TRUE | TRUE | TRUE |
| **906** | P63208 | S-phase kinase-associated protein | unknown | 12.01 | 1.48 | FALSE | TRUE | TRUE |
| **907** | O15226 | NF-kappa-B-repressing factor | NUCLEUS | 1.14 | -34.93 | FALSE | TRUE | TRUE |
| **908** | Q969X6 | U3 small nucleolar RNA-associated | NUCLEUS | -2.52 | -41.39 | FALSE | TRUE | TRUE |

| | | | | | | | | |
|---|---|---|---|---|---|---|---|---|
| **909** | Q15738 | Sterol-4-alpha-carboxylate 3-dehyd | ER | -6.83 | 31.02 | FALSE | TRUE | TRUE |
| **910** | Q15646 | 2'-5'-oligoadenylate synthase-like p | NUCLEUS | 0.53 | -22.96 | FALSE | TRUE | TRUE |
| **911** | Q96N66 | Lysophospholipid acyltransferase 7 | unknown | -0.14 | 35.57 | FALSE | TRUE | TRUE |
| **912** | P10398 | Serine/threonine-protein kinase A-I | unknown | -0.88 | -9.06 | FALSE | TRUE | TRUE |
| **913** | Q9NW82 | WD repeat-containing protein 70 | NUCLEUS | -2.54 | -13.88 | TRUE | TRUE | TRUE |
| **914** | Q9Y2S7 | Polymerase delta-interacting protei | MITOCHONDRIA | 40.23 | -12.39 | FALSE | TRUE | TRUE |
| **915** | O60814 | Histone H2B type 1-K | NUCLEUS-CHROMATIN | -22.88 | -13.82 | TRUE | FALSE | TRUE |
| **916** | Q9Y6C9 | Mitochondrial carrier homolog 2 | MITOCHONDRIA | 34.94 | -9.87 | FALSE | TRUE | TRUE |
| **917** | Q5JVF3 | PCI domain-containing protein 2 | NUCLEUS | -1.20 | -26.01 | FALSE | TRUE | TRUE |
| **918** | Q6YN16 | Hydroxysteroid dehydrogenase-like | unknown | 44.91 | -7.63 | FALSE | TRUE | TRUE |
| **919** | Q5TFE4 | 5'-nucleotidase domain-containing | unknown | 24.16 | 24.55 | TRUE | TRUE | TRUE |
| **920** | P27105 | Erythrocyte band 7 integral membr | unknown | -31.49 | -0.36 | TRUE | TRUE | TRUE |
| **921** | P00568 | Adenylate kinase isoenzyme 1 | unknown | 19.53 | 22.35 | FALSE | TRUE | TRUE |
| **922** | Q8WXF1 | Paraspeckle component 1 | NUCLEUS | 2.33 | -33.36 | TRUE | TRUE | TRUE |
| **923** | Q7L2J0 | 7SK snRNA methylphosphate cappi | NUCLEUS | 0.67 | -26.56 | FALSE | TRUE | TRUE |
| **924** | Q96C36 | Pyrroline-5-carboxylate reductase 2 | MITOCHONDRIA | 34.17 | -12.01 | FALSE | TRUE | TRUE |
| **925** | P54727 | UV excision repair protein RAD23 ho | unknown | 23.61 | 13.09 | FALSE | TRUE | TRUE |
| **926** | P11172 | Uridine 5'-monophosphate synthas | unknown | 9.08 | 7.55 | FALSE | TRUE | TRUE |
| **927** | Q16666 | Gamma-interferon-inducible protei | NUCLEUS-CHROMATIN | -16.24 | -12.37 | FALSE | TRUE | TRUE |
| **928** | Q96T37 | RNA-binding protein 15 | NUCLEUS | -1.16 | -34.92 | FALSE | TRUE | TRUE |
| **929** | Q9UL25 | Ras-related protein Rab-21 | unknown | -14.92 | 21.48 | FALSE | TRUE | TRUE |
| **930** | P49720 | Proteasome subunit beta type-3 | PROTEASOME | 22.10 | 6.93 | FALSE | TRUE | TRUE |
| **931** | Q96QK1 | Vacuolar protein sorting-associated | unknown | -14.62 | 8.67 | FALSE | TRUE | TRUE |
| **932** | P16070 | CD44 antigen | PM | -21.25 | 17.27 | TRUE | TRUE | TRUE |
| **933** | P50402 | Emerin | unknown | 24.23 | -10.77 | TRUE | TRUE | TRUE |
| **934** | Q96KB5 | Lymphokine-activated killer T-cell-o | unknown | 20.28 | 14.09 | FALSE | TRUE | TRUE |
| **935** | Q13616 | Cullin-1 | unknown | -3.53 | -3.52 | FALSE | TRUE | TRUE |
| **936** | P38117 | Electron transfer flavoprotein subur | MITOCHONDRIA | 39.00 | -5.70 | FALSE | TRUE | TRUE |
| **937** | Q99733 | Nucleosome assembly protein 1-lik | CYTOSOL | 19.73 | 17.29 | TRUE | TRUE | TRUE |
| **938** | Q92973 | Transportin-1 | unknown | 10.51 | 8.40 | FALSE | TRUE | TRUE |
| **939** | P35659 | Protein DEK | unknown | -21.84 | -21.49 | FALSE | TRUE | TRUE |
| **940** | P52294 | Importin subunit alpha-5 | unknown | 12.46 | 2.94 | FALSE | TRUE | TRUE |
| **941** | Q03252 | Lamin-B2 | unknown | 23.63 | -12.63 | FALSE | TRUE | TRUE |
| **942** | A0FGR8 | Extended synaptotagmin-2 | unknown | -11.43 | 29.26 | TRUE | TRUE | TRUE |
| **943** | Q8WWI1 | LIM domain only protein 7 | unknown | -3.10 | 2.22 | TRUE | TRUE | TRUE |
| **944** | Q96CT7 | Coiled-coil domain-containing prote | unknown | 18.16 | -19.53 | TRUE | TRUE | TRUE |
| **945** | P55263 | Adenosine kinase | unknown | 19.46 | 22.03 | FALSE | TRUE | TRUE |
| **946** | P43686 | 26S proteasome regulatory subunit | PROTEASOME | 21.18 | 6.67 | FALSE | TRUE | TRUE |

| | | | | | | | | |
|---|---|---|---|---|---|---|---|---|
| **947** | P25685 | DnaJ homolog subfamily B member | unknown | -3.97 | -8.87 | TRUE | TRUE | TRUE |
| **948** | Q9UNM6 | 26S proteasome non-ATPase regula | PROTEASOME | 21.19 | 6.34 | FALSE | TRUE | TRUE |
| **949** | P23368 | NAD-dependent malic enzyme, mit | MITOCHONDRIA | 38.12 | -3.63 | FALSE | TRUE | TRUE |
| **950** | Q9BW60 | Elongation of very long chain fatty a | unknown | -4.90 | 24.17 | FALSE | TRUE | TRUE |
| **951** | P55327 | Tumor protein D52 | CYTOSOL | 12.76 | 24.32 | TRUE | TRUE | TRUE |
| **952** | Q9Y2Z0 | Protein SGT1 homolog | CYTOSOL | 20.71 | 22.17 | TRUE | TRUE | TRUE |
| **953** | O43396 | Thioredoxin-like protein 1 | unknown | 20.52 | 20.22 | TRUE | TRUE | TRUE |
| **954** | P61163 | Alpha-centractin | unknown | 9.91 | 1.43 | FALSE | TRUE | FALSE |
| **955** | O43809 | Cleavage and polyadenylation spec | NUCLEUS | 4.86 | -33.38 | TRUE | TRUE | TRUE |
| **956** | Q7Z739 | YTH domain-containing family prot | unknown | -4.90 | 19.90 | FALSE | TRUE | TRUE |
| **957** | Q5VYK3 | Proteasome adapter and scaffold p | unknown | 5.62 | -16.81 | FALSE | TRUE | TRUE |
| **958** | Q04837 | Single-stranded DNA-binding protei | MITOCHONDRIA | 29.42 | -8.72 | FALSE | TRUE | TRUE |
| **959** | Q02978 | Mitochondrial 2-oxoglutarate/mala | MITOCHONDRIA | 28.88 | -4.12 | FALSE | TRUE | TRUE |
| **960** | Q15056 | Eukaryotic translation initiation fact | unknown | 24.57 | 22.04 | TRUE | TRUE | TRUE |
| **961** | Q9UKN8 | General transcription factor 3C poly | unknown | -21.63 | -21.36 | FALSE | TRUE | TRUE |
| **962** | Q5SSJ5 | Heterochromatin protein 1-binding | NUCLEUS-CHROMATIN | -20.94 | -13.00 | TRUE | TRUE | TRUE |
| **963** | Q6ZUT1 | Uncharacterized protein NKAPD1 | NUCLEUS | 1.52 | -28.18 | FALSE | TRUE | TRUE |
| **964** | Q9NQC3 | Reticulon-4 | unknown | -19.25 | 23.00 | TRUE | TRUE | TRUE |
| **965** | Q96HS1 | Serine/threonine-protein phosphata | MITOCHONDRIA | 31.42 | -14.80 | FALSE | TRUE | TRUE |
| **966** | P11216 | Glycogen phosphorylase, brain form | CYTOSOL | 18.19 | 18.22 | FALSE | TRUE | TRUE |
| **967** | Q9UBQ0 | Vacuolar protein sorting-associated | unknown | -14.61 | 8.50 | FALSE | TRUE | TRUE |
| **968** | Q7L576 | Cytoplasmic FMR1-interacting prot | unknown | -9.56 | 3.53 | FALSE | TRUE | TRUE |
| **969** | Q9UHV9 | Prefoldin subunit 2 | unknown | 20.58 | 9.55 | FALSE | TRUE | TRUE |
| **970** | Q9Y490 | Talin-1 | unknown | 1.53 | 10.73 | TRUE | TRUE | TRUE |
| **971** | Q96G23 | Ceramide synthase 2 | unknown | -5.57 | 25.51 | FALSE | TRUE | FALSE |
| **972** | P15927 | Replication protein A 32 kDa subuni | unknown | 23.49 | 12.83 | FALSE | TRUE | TRUE |
| **973** | Q16186 | Proteasomal ubiquitin receptor ADR | PROTEASOME | 12.79 | 23.29 | FALSE | TRUE | TRUE |
| **974** | Q15370 | Elongin-B | unknown | 17.12 | 6.66 | TRUE | TRUE | TRUE |
| **975** | Q9NY12 | H/ACA ribonucleoprotein complex s | NUCLEUS | -3.40 | -38.55 | FALSE | TRUE | TRUE |
| **976** | Q9Y4P3 | Transducin beta-like protein 2 | ER | 5.14 | 37.98 | FALSE | TRUE | TRUE |
| **977** | Q14847 | LIM and SH3 domain protein 1 | unknown | -3.25 | 8.83 | TRUE | TRUE | TRUE |
| **978** | O75874 | Isocitrate dehydrogenase [NADP] c | CYTOSOL | 21.47 | 22.66 | FALSE | TRUE | TRUE |
| **979** | P30046 | D-dopachrome decarboxylase | unknown | 19.47 | 13.14 | FALSE | TRUE | TRUE |
| **980** | P52888 | Thimet oligopeptidase | CYTOSOL | 20.84 | 24.46 | FALSE | TRUE | TRUE |
| **981** | P26885 | Peptidyl-prolyl cis-trans isomerase I | ER | -6.29 | 35.85 | FALSE | TRUE | FALSE |
| **982** | Q9BTV4 | Transmembrane protein 43 | ER | 2.43 | 32.24 | FALSE | TRUE | TRUE |
| **983** | P43246 | DNA mismatch repair protein Msh2 | NUCLEUS | -10.11 | -14.73 | FALSE | TRUE | TRUE |
| **984** | P67812 | Signal peptidase complex catalytic s | unknown | 2.86 | 33.30 | FALSE | TRUE | TRUE |

| | | | | | | | | | |
|---|---|---|---|---|---|---|---|---|---|
| **985** | P67870 | Casein kinase II subunit beta | unknown | 12.24 | 4.01 | FALSE | TRUE | TRUE |
| **986** | Q96HC4 | PDZ and LIM domain protein 5 | unknown | -5.00 | -6.56 | TRUE | TRUE | TRUE |
| **987** | P22061 | Protein-L-isoaspartate(D-aspartate) | unknown | 8.49 | 10.00 | TRUE | TRUE | TRUE |
| **988** | Q8WUM0 | Nuclear pore complex protein Nup1 | NUCLEUS | -20.89 | -29.60 | FALSE | TRUE | TRUE |
| **989** | P53004 | Biliverdin reductase A | CYTOSOL | 22.27 | 22.74 | FALSE | TRUE | TRUE |
| **990** | P42330 | Aldo-keto reductase family 1 meml | unknown | 12.44 | 22.55 | FALSE | TRUE | TRUE |
| **991** | Q7Z478 | ATP-dependent RNA helicase DHX2 | RIBOSOME 40S | 7.79 | -24.75 | TRUE | TRUE | TRUE |
| **992** | Q9HC38 | Glyoxalase domain-containing prot | CYTOSOL | 21.59 | 21.49 | FALSE | TRUE | TRUE |
| **993** | Q96KA5 | Cleft lip and palate transmembrane | ER | -3.63 | 32.97 | FALSE | TRUE | TRUE |
| **994** | Q8N5M9 | Protein jagunal homolog 1 | ER | 4.33 | 30.13 | FALSE | TRUE | TRUE |
| **995** | Q9Y3B4 | Splicing factor 3B subunit 6 | NUCLEUS | 4.04 | -35.57 | FALSE | TRUE | TRUE |
| **996** | P13807 | Glycogen [starch] synthase, muscle | unknown | 3.26 | 5.94 | FALSE | TRUE | FALSE |
| **997** | Q8IX12 | Cell division cycle and apoptosis reg | NUCLEUS | -20.32 | -32.86 | FALSE | TRUE | TRUE |
| **998** | Q9UBE0 | SUMO-activating enzyme subunit 1 | unknown | 27.29 | 23.06 | FALSE | TRUE | TRUE |
| **999** | Q06265 | Exosome complex component RRP4 | NUCLEUS | -10.56 | -26.27 | FALSE | TRUE | TRUE |
| **1000** | Q9H0S4 | Probable ATP-dependent RNA helic | NUCLEUS | -0.99 | -29.99 | FALSE | TRUE | TRUE |
| **1001** | Q16222 | UDP-N-acetylhexosamine pyrophos | CYTOSOL | 20.71 | 21.41 | FALSE | TRUE | TRUE |
| **1002** | Q14683 | Structural maintenance of chromos | NUCLEUS-CHROMATIN | -18.20 | -16.04 | FALSE | TRUE | TRUE |
| **1003** | P49005 | DNA polymerase delta subunit 2 | unknown | 16.14 | 2.97 | FALSE | TRUE | TRUE |
| **1004** | Q9NUQ8 | ATP-binding cassette sub-family F r | unknown | -2.35 | -9.77 | FALSE | TRUE | TRUE |
| **1005** | Q9Y3B3 | Transmembrane emp24 domain-co | unknown | -10.90 | 32.76 | FALSE | TRUE | FALSE |
| **1006** | P55036 | 26S proteasome non-ATPase regul | PROTEASOME | 19.89 | 7.00 | TRUE | TRUE | TRUE |
| **1007** | O15372 | Eukaryotic translation initiation fact | unknown | 13.67 | -10.93 | TRUE | TRUE | TRUE |
| **1008** | Q14573 | Inositol 1,4,5-trisphosphate recepto | ER | -4.28 | 30.57 | FALSE | TRUE | TRUE |
| **1009** | Q14694 | Ubiquitin carboxyl-terminal hydrola | unknown | 6.76 | -20.23 | FALSE | TRUE | TRUE |
| **1010** | Q9NVI1 | Fanconi anemia group I protein | NUCLEUS | -9.66 | -15.53 | FALSE | TRUE | TRUE |
| **1011** | P26583 | High mobility group protein B2 | NUCLEUS-CHROMATIN | -16.52 | -12.19 | FALSE | TRUE | TRUE |
| **1012** | P68400 | Casein kinase II subunit alpha | unknown | 12.32 | 3.74 | FALSE | TRUE | FALSE |
| **1013** | Q99570 | Phosphoinositide 3-kinase regulato | unknown | 4.63 | -12.44 | FALSE | TRUE | TRUE |
| **1014** | Q9HCE1 | Helicase MOV-10 | unknown | 4.19 | -22.44 | FALSE | TRUE | TRUE |
| **1015** | P51570 | Galactokinase | CYTOSOL | 19.77 | 16.43 | FALSE | TRUE | TRUE |
| **1016** | Q8IYB8 | ATP-dependent RNA helicase SUPV | MITOCHONDRIA | 30.84 | -13.63 | FALSE | TRUE | TRUE |
| **1017** | P10515 | Dihydrolipoyllysine-residue acetyltr | MITOCHONDRIA | 42.51 | -11.30 | FALSE | TRUE | TRUE |
| **1018** | P51648 | Fatty aldehyde dehydrogenase | ER | -0.06 | 33.77 | FALSE | TRUE | TRUE |
| **1019** | Q9H0H5 | Rac GTPase-activating protein 1 | unknown | -14.04 | -11.19 | TRUE | TRUE | TRUE |
| **1020** | P56545 | C-terminal-binding protein 2 | NUCLEUS | -8.38 | -15.77 | FALSE | TRUE | FALSE |
| **1021** | Q9BRU9 | rRNA-processing protein UTP23 hor | NUCLEUS | 1.42 | -29.35 | FALSE | TRUE | FALSE |
| **1022** | P30566 | Adenylosuccinate lyase | unknown | 4.38 | -11.36 | FALSE | TRUE | TRUE |

| | | | | | | | | |
|---|---|---|---|---|---|---|---|---|
| **1023** | O95816 | BAG family molecular chaperone re | unknown | 1.74 | 26.29 | FALSE | TRUE | TRUE |
| **1024** | Q9HB07 | UPF0160 protein MYG1, mitochond | CYTOSOL | 16.72 | 16.01 | FALSE | TRUE | TRUE |
| **1025** | Q9P2R7 | Succinate--CoA ligase [ADP-forming | MITOCHONDRIA | 38.15 | -7.43 | FALSE | TRUE | TRUE |
| **1026** | Q7L1Q6 | Basic leucine zipper and W2 domair | NUCLEUS | -3.14 | -9.69 | FALSE | TRUE | TRUE |
| **1027** | Q9BWM7 | Sideroflexin-3 | MITOCHONDRIA | 35.15 | -7.30 | FALSE | TRUE | TRUE |
| **1028** | Q9H488 | GDP-fucose protein O-fucosyltransf | ER | -1.37 | 39.16 | FALSE | TRUE | TRUE |
| **1029** | Q9H8H0 | Nucleolar protein 11 | NUCLEUS | -2.66 | -41.38 | FALSE | TRUE | TRUE |
| **1030** | P78344 | Eukaryotic translation initiation fact | unknown | 5.84 | -22.38 | TRUE | TRUE | TRUE |
| **1031** | P30043 | Flavin reductase (NADPH) | CYTOSOL | 21.17 | 14.55 | FALSE | TRUE | TRUE |
| **1032** | Q02790 | Peptidyl-prolyl cis-trans isomerase I | CYTOSOL | 22.98 | 21.71 | FALSE | TRUE | TRUE |
| **1033** | Q13045 | Protein flightless-1 homolog | unknown | 5.05 | 0.70 | FALSE | TRUE | TRUE |
| **1034** | Q8N766 | ER membrane protein complex subu | ER | 3.00 | 35.04 | FALSE | TRUE | TRUE |
| **1035** | P98082 | Disabled homolog 2 | unknown | -8.86 | 14.36 | TRUE | TRUE | TRUE |
| **1036** | Q13442 | 28 kDa heat- and acid-stable phosp | unknown | 3.70 | -22.54 | TRUE | TRUE | TRUE |
| **1037** | P35270 | Sepiapterin reductase | unknown | 20.09 | -3.03 | FALSE | TRUE | TRUE |
| **1038** | Q13501 | Sequestosome-1 | unknown | -30.72 | 3.10 | FALSE | TRUE | TRUE |
| **1039** | Q15020 | Squamous cell carcinoma antigen re | unknown | 21.63 | 11.91 | TRUE | TRUE | TRUE |
| **1040** | Q16637 | Survival motor neuron protein | NUCLEUS | -1.00 | -22.62 | FALSE | TRUE | TRUE |
| **1041** | Q14247 | Src substrate cortactin | NUCLEUS | 3.91 | 3.73 | TRUE | TRUE | TRUE |
| **1042** | Q9BTX1 | Nucleoporin NDC1 | unknown | 24.27 | -10.12 | FALSE | TRUE | TRUE |
| **1043** | Q9BUB7 | Transmembrane protein 70, mitoch | unknown | 31.96 | -3.87 | FALSE | TRUE | TRUE |
| **1044** | P49321 | Nuclear autoantigenic sperm protei | unknown | 27.33 | 23.13 | TRUE | TRUE | TRUE |
| **1045** | P19388 | DNA-directed RNA polymerases I, II | NUCLEUS | -13.40 | -29.35 | FALSE | TRUE | TRUE |
| **1046** | O60610 | Protein diaphanous homolog 1 | unknown | 5.39 | 9.32 | TRUE | TRUE | TRUE |
| **1047** | P35244 | Replication protein A 14 kDa subuni | unknown | 18.17 | 11.03 | FALSE | TRUE | TRUE |
| **1048** | Q13867 | Bleomycin hydrolase | unknown | 13.89 | 17.54 | FALSE | TRUE | TRUE |
| **1049** | P24666 | Low molecular weight phosphotyro | CYTOSOL | 17.99 | 15.25 | FALSE | TRUE | TRUE |
| **1050** | O43670 | BUB3-interacting and GLEBS motif-c | NUCLEUS | 2.08 | -31.61 | FALSE | TRUE | TRUE |
| **1051** | Q9NPI1 | Bromodomain-containing protein 7 | unknown | -18.46 | -11.22 | FALSE | TRUE | TRUE |
| **1052** | O00422 | Histone deacetylase complex subur | NUCLEUS | -4.47 | -40.20 | FALSE | TRUE | TRUE |
| **1053** | Q12797 | Aspartyl/asparaginyl beta-hydroxy | ER | 4.42 | 36.63 | FALSE | TRUE | TRUE |
| **1054** | P20073 | Annexin A7 | unknown | 13.25 | 17.14 | FALSE | TRUE | TRUE |
| **1055** | O75396 | Vesicle-trafficking protein SEC22b | ER | -4.36 | 32.65 | TRUE | TRUE | TRUE |
| **1056** | O14579 | Coatomer subunit epsilon | unknown | 6.48 | 4.07 | FALSE | TRUE | TRUE |
| **1057** | P49721 | Proteasome subunit beta type-2 | PROTEASOME | 22.17 | 6.77 | FALSE | TRUE | TRUE |
| **1058** | O43252 | Bifunctional 3'-phosphoadenosine 5 | unknown | 17.49 | 6.69 | FALSE | TRUE | TRUE |
| **1059** | O60524 | Nuclear export mediator factor NEM | unknown | 9.26 | -3.93 | TRUE | TRUE | TRUE |
| **1060** | Q9H4L4 | Sentrin-specific protease 3 | NUCLEUS | -15.58 | -25.20 | FALSE | TRUE | TRUE |

| | | | | | | | | |
|---|---|---|---|---|---|---|---|---|
| **1061** | Q15054 | DNA polymerase delta subunit 3 | NUCLEUS | 3.06 | -3.01 | TRUE | TRUE | TRUE |
| **1062** | O95347 | Structural maintenance of chromos | unknown | -7.71 | -9.03 | TRUE | TRUE | TRUE |
| **1063** | Q15155 | Nodal modulator 1 | unknown | 3.41 | 38.26 | FALSE | TRUE | TRUE |
| **1064** | P05556 | Integrin beta-1 | PM | -20.13 | 18.88 | TRUE | TRUE | TRUE |
| **1065** | Q6UW68 | Transmembrane protein 205 | ER | -4.09 | 27.40 | FALSE | TRUE | FALSE |
| **1066** | O96000 | NADH dehydrogenase [ubiquinone] | MITOCHONDRIA | 31.05 | -14.33 | FALSE | TRUE | TRUE |
| **1067** | Q9NQ55 | Suppressor of SWI4 1 homolog | NUCLEUS | -0.55 | -40.86 | FALSE | TRUE | TRUE |
| **1068** | Q96D46 | 60S ribosomal export protein NMD3 | unknown | 3.38 | -17.72 | FALSE | TRUE | TRUE |
| **1069** | P50995 | Annexin A11 | unknown | 8.98 | 13.59 | FALSE | TRUE | TRUE |
| **1070** | Q9H9J4 | Ubiquitin carboxyl-terminal hydrola | NUCLEUS | 1.25 | -36.56 | FALSE | TRUE | TRUE |
| **1071** | Q9NR50 | Translation initiation factor eIF-2B s | unknown | 12.84 | 6.80 | FALSE | TRUE | FALSE |
| **1072** | Q9Y2R5 | 28S ribosomal protein S17, mitocho | MITOCHONDRIA | 40.62 | -5.85 | FALSE | TRUE | TRUE |
| **1073** | Q9BPX3 | Condensin complex subunit 3 | unknown | -8.09 | -9.03 | FALSE | TRUE | FALSE |
| **1074** | O60684 | Importin subunit alpha-7 | unknown | 12.70 | 6.87 | FALSE | TRUE | TRUE |
| **1075** | O75223 | Gamma-glutamylcyclotransferase | unknown | 19.69 | 25.27 | FALSE | TRUE | TRUE |
| **1076** | Q9HD45 | Transmembrane 9 superfamily mer | GOLGI | -18.00 | 32.81 | FALSE | TRUE | TRUE |
| **1077** | P62330 | ADP-ribosylation factor 6 | unknown | -11.47 | 19.80 | FALSE | TRUE | FALSE |
| **1078** | Q00765 | Receptor expression-enhancing pro | unknown | -13.08 | 29.78 | FALSE | TRUE | TRUE |
| **1079** | P10155 | 60 kDa SS-A/Ro ribonucleoprotein | unknown | 8.74 | 2.95 | FALSE | TRUE | FALSE |
| **1080** | Q9P0L0 | Vesicle-associated membrane prot | unknown | -8.39 | 35.74 | FALSE | TRUE | TRUE |
| **1081** | Q6UXN9 | WD repeat-containing protein 82 | NUCLEUS | -6.92 | -30.61 | FALSE | TRUE | TRUE |
| **1082** | O60443 | Gasdermin-E | unknown | 7.69 | 12.71 | FALSE | TRUE | TRUE |
| **1083** | Q9NYU2 | UDP-glucose:glycoprotein glucosylt | ER | -2.33 | 37.69 | FALSE | TRUE | TRUE |
| **1084** | Q96A35 | 39S ribosomal protein L24, mitocho | MITOCHONDRIA | 43.04 | -6.65 | FALSE | TRUE | FALSE |
| **1085** | Q9UIG0 | Tyrosine-protein kinase BAZ1B | NUCLEUS-CHROMATIN | -21.88 | -14.05 | TRUE | TRUE | TRUE |
| **1086** | Q9BRP8 | Partner of Y14 and mago | unknown | 12.21 | -21.62 | TRUE | TRUE | TRUE |
| **1087** | Q9H1A4 | Anaphase-promoting complex subu | NUCLEUS | -14.46 | -27.11 | FALSE | TRUE | TRUE |
| **1088** | P82979 | SAP domain-containing ribonucleop | NUCLEUS | -11.16 | -24.27 | FALSE | TRUE | TRUE |
| **1089** | Q00341 | Vigilin | RIBOSOME 60S | 16.60 | -23.92 | TRUE | TRUE | TRUE |
| **1090** | Q8IY67 | Ribonucleoprotein PTB-binding 1 | NUCLEUS | -18.19 | -27.58 | TRUE | TRUE | TRUE |
| **1091** | Q9UBT2 | SUMO-activating enzyme subunit 2 | unknown | 27.33 | 23.03 | FALSE | TRUE | TRUE |
| **1092** | Q08257 | Quinone oxidoreductase | MITOCHONDRIA | 33.62 | -5.67 | FALSE | TRUE | TRUE |
| **1093** | O94905 | Erlin-2 | ER | -4.83 | 30.93 | FALSE | TRUE | TRUE |
| **1094** | Q99615 | DnaJ homolog subfamily C member | unknown | 11.60 | 4.18 | FALSE | TRUE | TRUE |
| **1095** | Q13011 | Delta(3,5)-Delta(2,4)-dienoyl-CoA is | MITOCHONDRIA | 36.50 | -6.04 | FALSE | TRUE | TRUE |
| **1096** | Q9BRJ2 | 39S ribosomal protein L45, mitocho | MITOCHONDRIA | 39.77 | -5.12 | FALSE | TRUE | TRUE |
| **1097** | O14745 | Na(+)/H(+) exchange regulatory cof | PM | -27.16 | 11.02 | TRUE | TRUE | TRUE |
| **1098** | Q9BTT0 | Acidic leucine-rich nuclear phospho | unknown | 22.07 | 13.13 | FALSE | TRUE | TRUE |

| | | | | | | | | |
|---|---|---|---|---|---|---|---|---|
| **1099** | Q99808 | Equilibrative nucleoside transporter | unknown | -21.57 | 20.29 | TRUE | TRUE | TRUE |
| **1100** | Q9Y512 | Sorting and assembly machinery co | unknown | 27.87 | -13.10 | FALSE | TRUE | TRUE |
| **1101** | Q13435 | Splicing factor 3B subunit 2 | NUCLEUS | 4.57 | -35.16 | TRUE | TRUE | TRUE |
| **1102** | Q99536 | Synaptic vesicle membrane protein | unknown | -7.69 | 18.74 | FALSE | TRUE | TRUE |
| **1103** | P52788 | Spermine synthase | CYTOSOL | 20.23 | 22.96 | FALSE | TRUE | TRUE |
| **1104** | P35080 | Profilin-2 | CYTOSOL | 24.03 | 19.78 | FALSE | TRUE | TRUE |
| **1105** | P21266 | Glutathione S-transferase Mu 3 | unknown | 21.52 | 13.72 | FALSE | TRUE | TRUE |
| **1106** | Q09161 | Nuclear cap-binding protein subunit | NUCLEUS | -12.48 | -29.33 | FALSE | TRUE | TRUE |
| **1107** | Q9Y5J1 | U3 small nucleolar RNA-associated | NUCLEUS | -2.18 | -40.64 | FALSE | TRUE | TRUE |
| **1108** | P49773 | Histidine triad nucleotide-binding pr | unknown | 23.17 | 22.70 | FALSE | TRUE | TRUE |
| **1109** | Q00796 | Sorbitol dehydrogenase | unknown | 2.52 | -13.33 | FALSE | TRUE | TRUE |
| **1110** | Q01844 | RNA-binding protein EWS | NUCLEUS | -6.57 | -30.25 | FALSE | TRUE | TRUE |
| **1111** | O75947 | ATP synthase subunit d, mitochond | MITOCHONDRIA | 41.94 | -12.78 | FALSE | TRUE | TRUE |
| **1112** | Q8NBQ5 | Estradiol 17-beta-dehydrogenase 1 | unknown | 5.73 | 33.38 | FALSE | TRUE | TRUE |
| **1113** | Q9Y3E5 | Peptidyl-tRNA hydrolase 2, mitocho | unknown | 2.81 | 34.47 | FALSE | TRUE | TRUE |
| **1114** | Q12789 | General transcription factor 3C poly | unknown | -22.55 | -22.15 | FALSE | TRUE | TRUE |
| **1115** | Q96IU4 | Protein ABHD14B | unknown | 22.87 | 23.00 | FALSE | TRUE | TRUE |
| **1116** | P23258 | Tubulin gamma-1 chain | unknown | 5.99 | -24.53 | FALSE | TRUE | TRUE |
| **1117** | P52815 | 39S ribosomal protein L12, mitocho | MITOCHONDRIA | 39.80 | -10.93 | FALSE | TRUE | TRUE |
| **1118** | Q6RFH5 | WD repeat-containing protein 74 | NUCLEUS | -1.20 | -37.52 | FALSE | TRUE | TRUE |
| **1119** | O15258 | Protein RER1 | unknown | -0.11 | 33.57 | FALSE | TRUE | TRUE |
| **1120** | Q5W0B1 | RING finger protein 219 | unknown | 9.60 | -19.31 | TRUE | TRUE | TRUE |
| **1121** | Q15942 | Zyxin | unknown | -3.78 | 9.37 | TRUE | TRUE | TRUE |
| **1122** | P09543 | 2',3'-cyclic-nucleotide 3'-phosphodi | unknown | -14.91 | 15.75 | FALSE | TRUE | TRUE |
| **1123** | Q92544 | Transmembrane 9 superfamily mer | unknown | -22.31 | 30.88 | FALSE | TRUE | TRUE |
| **1124** | Q9NUQ9 | Protein FAM49B | unknown | 11.11 | 14.60 | FALSE | TRUE | FALSE |
| **1125** | P51116 | Fragile X mental retardation syndro | unknown | 8.21 | -25.48 | FALSE | TRUE | TRUE |
| **1126** | Q9GZR7 | ATP-dependent RNA helicase DDX2 | NUCLEUS | -1.36 | -36.83 | FALSE | TRUE | TRUE |
| **1127** | Q9NWB6 | Arginine and glutamate-rich protein | NUCLEUS | 2.41 | -31.92 | TRUE | FALSE | TRUE |
| **1128** | P09001 | 39S ribosomal protein L3, mitochon | MITOCHONDRIA | 42.91 | -11.63 | FALSE | TRUE | TRUE |
| **1129** | Q9UKY7 | Protein CDV3 homolog | unknown | 11.40 | -1.16 | TRUE | TRUE | TRUE |
| **1130** | Q9H936 | Mitochondrial glutamate carrier 1 | unknown | 44.28 | -13.16 | FALSE | TRUE | FALSE |
| **1131** | Q9H2P0 | Activity-dependent neuroprotector | NUCLEUS | -25.54 | -25.92 | TRUE | TRUE | TRUE |
| **1132** | Q9BXJ9 | N-alpha-acetyltransferase 15, NatA | unknown | 10.13 | -13.00 | FALSE | TRUE | TRUE |
| **1133** | Q92879 | CUGBP Elav-like family member 1 | NUCLEUS | -19.91 | -32.82 | FALSE | TRUE | TRUE |
| **1134** | Q969H8 | Myeloid-derived growth factor | ER | -2.19 | 37.48 | FALSE | TRUE | FALSE |
| **1135** | Q13409 | Cytoplasmic dynein 1 intermediate | unknown | -11.34 | -7.39 | FALSE | TRUE | TRUE |
| **1136** | Q9Y570 | Protein phosphatase methylesteras | CYTOSOL | 19.41 | 22.42 | FALSE | TRUE | TRUE |

| | | | | | | | | |
|---|---|---|---|---|---|---|---|---|
| **1137** | Q9H490 | Phosphatidylinositol glycan anchor | unknown | 2.56 | 31.19 | FALSE | TRUE | TRUE |
| **1138** | P00387 | NADH-cytochrome b5 reductase 3 | unknown | 1.79 | 33.20 | FALSE | TRUE | TRUE |
| **1139** | O95602 | DNA-directed RNA polymerase I sul | NUCLEUS | -24.52 | -26.16 | FALSE | TRUE | TRUE |
| **1140** | Q9NTJ5 | Phosphatidylinositide phosphatase | unknown | -10.49 | 30.00 | FALSE | TRUE | TRUE |
| **1141** | P30626 | Sorcin | unknown | 18.35 | 18.94 | FALSE | TRUE | TRUE |
| **1142** | Q16401 | 26S proteasome non-ATPase regula | unknown | 20.77 | 19.27 | FALSE | TRUE | TRUE |
| **1143** | Q9ULC4 | Malignant T-cell-amplified sequenc | unknown | -11.54 | -10.70 | FALSE | TRUE | TRUE |
| **1144** | O75251 | NADH dehydrogenase [ubiquinone] | MITOCHONDRIA | 30.94 | -9.96 | FALSE | TRUE | FALSE |
| **1145** | Q8WW12 | PEST proteolytic signal-containing r | unknown | 12.12 | -11.12 | TRUE | TRUE | TRUE |
| **1146** | Q96RS6 | NudC domain-containing protein 1 | unknown | 20.99 | 16.41 | FALSE | TRUE | TRUE |
| **1147** | Q32P28 | Prolyl 3-hydroxylase 1 | unknown | 2.48 | 39.54 | FALSE | TRUE | TRUE |
| **1148** | O76070 | Gamma-synuclein | CYTOSOL | 17.89 | 22.50 | TRUE | TRUE | TRUE |
| **1149** | Q9Y2A7 | Nck-associated protein 1 | unknown | -9.56 | 3.67 | FALSE | TRUE | TRUE |
| **1150** | Q9HAV4 | Exportin-5 | unknown | 12.97 | -2.13 | FALSE | TRUE | TRUE |
| **1151** | Q9UBU9 | Nuclear RNA export factor 1 | NUCLEUS | -5.30 | -23.95 | FALSE | TRUE | TRUE |
| **1152** | Q8WTT2 | Nucleolar complex protein 3 homolo | NUCLEUS | 2.27 | -35.70 | FALSE | TRUE | TRUE |
| **1153** | Q92597 | Protein NDRG1 | unknown | 8.95 | 13.46 | FALSE | TRUE | TRUE |
| **1154** | Q15843 | NEDD8 | unknown | -33.55 | -1.90 | FALSE | TRUE | FALSE |
| **1155** | Q6P1J9 | Parafibromin | NUCLEUS | -13.32 | -27.07 | FALSE | TRUE | TRUE |
| **1156** | Q8TD26 | Chromodomain-helicase-DNA-bindi | NUCLEUS | -20.44 | -22.49 | TRUE | TRUE | TRUE |
| **1157** | Q9H832 | Ubiquitin-conjugating enzyme E2 Z | unknown | 17.16 | 12.91 | FALSE | TRUE | TRUE |
| **1158** | Q96J01 | THO complex subunit 3 | NUCLEUS | -11.49 | -25.75 | FALSE | TRUE | FALSE |
| **1159** | Q13144 | Translation initiation factor eIF-2B s | unknown | 12.99 | 6.87 | FALSE | TRUE | TRUE |
| **1160** | P10768 | S-formylglutathione hydrolase | unknown | 20.89 | 24.63 | FALSE | TRUE | TRUE |
| **1161** | O60762 | Dolichol-phosphate mannosyltransf | ER | 4.82 | 34.86 | FALSE | TRUE | TRUE |
| **1162** | Q9H6R0 | ATP-dependent RNA helicase DHX3 | NUCLEUS | -0.86 | -38.61 | FALSE | TRUE | TRUE |
| **1163** | P46459 | Vesicle-fusing ATPase | unknown | -3.76 | 4.75 | FALSE | TRUE | TRUE |
| **1164** | Q06323 | Proteasome activator complex subu | unknown | 25.29 | 23.49 | FALSE | TRUE | TRUE |
| **1165** | P33176 | Kinesin-1 heavy chain | unknown | 0.37 | -4.72 | TRUE | TRUE | TRUE |
| **1166** | Q9HDC9 | Adipocyte plasma membrane-asso | ER | -5.92 | 32.27 | FALSE | TRUE | TRUE |
| **1167** | Q9Y316 | Protein MEMO1 | unknown | 11.99 | 22.70 | FALSE | TRUE | TRUE |
| **1168** | O00767 | Acyl-CoA desaturase | unknown | -2.51 | 31.80 | FALSE | TRUE | TRUE |
| **1169** | P49406 | 39S ribosomal protein L19, mitocho | unknown | 44.10 | -13.36 | FALSE | TRUE | TRUE |
| **1170** | P23634 | Plasma membrane calcium-transpo | PM | -23.48 | 16.18 | TRUE | TRUE | TRUE |
| **1171** | Q96QD8 | Sodium-coupled neutral amino acid | unknown | -34.16 | 8.03 | TRUE | TRUE | TRUE |
| **1172** | O15355 | Protein phosphatase 1G | unknown | 27.17 | 22.58 | TRUE | TRUE | TRUE |
| **1173** | P36776 | Lon protease homolog, mitochondri | MITOCHONDRIA | 36.20 | -2.47 | FALSE | TRUE | TRUE |
| **1174** | O94826 | Mitochondrial import receptor subu | MITOCHONDRIA | 42.63 | -6.37 | FALSE | TRUE | TRUE |

| | | | | | | | | | |
|---|---|---|---|---|---|---|---|---|---|
| **1175** | P17612 | cAMP-dependent protein kinase ca | unknown | 1.89 | 11.56 | FALSE | TRUE | FALSE |
| **1176** | P82650 | 28S ribosomal protein S22, mitocho | MITOCHONDRIA | 40.39 | -10.06 | FALSE | TRUE | TRUE |
| **1177** | O15145 | Actin-related protein 2/3 complex s | unknown | 1.53 | -14.57 | FALSE | TRUE | TRUE |
| **1178** | O14776 | Transcription elongation regulator 1 | NUCLEUS | -1.76 | -34.06 | TRUE | TRUE | TRUE |
| **1179** | Q9NP72 | Ras-related protein Rab-18 | unknown | -19.28 | 22.78 | FALSE | TRUE | FALSE |
| **1180** | P57678 | Gem-associated protein 4 | NUCLEUS | -0.88 | -22.42 | FALSE | TRUE | TRUE |
| **1181** | Q9GZT3 | SRA stem-loop-interacting RNA-bin | MITOCHONDRIA | 40.86 | -1.81 | FALSE | TRUE | TRUE |
| **1182** | P48960 | CD97 antigen | unknown | -30.69 | 13.99 | FALSE | TRUE | TRUE |
| **1183** | Q9NTX5 | Ethylmalonyl-CoA decarboxylase | unknown | 24.14 | -6.55 | TRUE | TRUE | TRUE |
| **1184** | Q96C19 | EF-hand domain-containing protein | unknown | 19.04 | 16.40 | TRUE | TRUE | TRUE |
| **1185** | P47985 | Cytochrome b-c1 complex subunit R | MITOCHONDRIA | 39.03 | -0.19 | FALSE | TRUE | FALSE |
| **1186** | Q93008 | Probable ubiquitin carboxyl-termina | unknown | 14.41 | 8.12 | FALSE | TRUE | TRUE |
| **1187** | P09012 | U1 small nuclear ribonucleoprotein | NUCLEUS | 6.09 | -34.32 | FALSE | TRUE | TRUE |
| **1188** | Q16698 | 2,4-dienoyl-CoA reductase, mitocho | MITOCHONDRIA | 32.36 | -12.54 | FALSE | TRUE | TRUE |
| **1189** | Q96CN7 | Isochorismatase domain-containing | unknown | 10.11 | 8.49 | FALSE | TRUE | TRUE |
| **1190** | P61086 | Ubiquitin-conjugating enzyme E2 K | unknown | 13.64 | 23.94 | FALSE | TRUE | TRUE |
| **1191** | O43747 | AP-1 complex subunit gamma-1 | unknown | -33.26 | 19.63 | FALSE | TRUE | TRUE |
| **1192** | Q9Y6A9 | Signal peptidase complex subunit 1 | unknown | 5.12 | 35.26 | FALSE | TRUE | FALSE |
| **1193** | P43307 | Translocon-associated protein subu | ER | 5.16 | 37.74 | FALSE | TRUE | TRUE |
| **1194** | Q01780 | Exosome component 10 | NUCLEUS | -10.90 | -25.29 | FALSE | TRUE | TRUE |
| **1195** | O95486 | Protein transport protein Sec24A | unknown | 9.59 | -18.27 | FALSE | TRUE | TRUE |
| **1196** | Q8WXA9 | Splicing regulatory glutamine/lysin | NUCLEUS | 3.53 | -31.38 | FALSE | TRUE | TRUE |
| **1197** | O15173 | Membrane-associated progesteror | unknown | -7.32 | 31.90 | FALSE | TRUE | TRUE |
| **1198** | P0C0S5 | Histone H2A.Z | NUCLEUS-CHROMATIN | -23.02 | -13.69 | FALSE | FALSE | TRUE |
| **1199** | Q9Y6K5 | 2'-5'-oligoadenylate synthase 3 | unknown | 9.79 | -20.45 | FALSE | TRUE | TRUE |
| **1200** | Q99436 | Proteasome subunit beta type-7 | PROTEASOME | 22.43 | 6.86 | FALSE | TRUE | TRUE |
| **1201** | Q14116 | Interleukin-18 | CYTOSOL | 22.51 | 19.61 | FALSE | TRUE | TRUE |
| **1202** | Q7RTV0 | PHD finger-like domain-containing | NUCLEUS | 3.89 | -35.20 | FALSE | TRUE | TRUE |
| **1203** | Q9NP97 | Dynein light chain roadblock-type 1 | NUCLEUS | -11.24 | -7.31 | FALSE | TRUE | FALSE |
| **1204** | O95747 | Serine/threonine-protein kinase | unknown | 13.95 | 4.95 | TRUE | TRUE | TRUE |
| **1205** | Q53H12 | Acylglycerol kinase, mitochondrial | MITOCHONDRIA | 36.04 | -15.10 | FALSE | TRUE | FALSE |
| **1206** | Q9UNZ2 | NSFL1 cofactor p47 | unknown | 15.27 | 21.78 | TRUE | TRUE | TRUE |
| **1207** | P68036 | Ubiquitin-conjugating enzyme E2 L3 | unknown | 24.37 | 19.86 | FALSE | TRUE | TRUE |
| **1208** | Q8N684 | Cleavage and polyadenylation spec | NUCLEUS | -9.35 | -35.99 | FALSE | TRUE | TRUE |
| **1209** | P58546 | Myotrophin | unknown | 12.41 | 18.62 | FALSE | TRUE | TRUE |
| **1210** | Q08380 | Galectin-3-binding protein | PM | -25.99 | 17.88 | FALSE | TRUE | FALSE |
| **1211** | Q9NRG9 | Aladin | unknown | 24.30 | -9.99 | FALSE | TRUE | FALSE |
| **1212** | Q0VDF9 | Heat shock 70 kDa protein 14 | NUCLEUS | -3.20 | -14.09 | FALSE | TRUE | TRUE |

| | | | | | | | | |
|---|---|---|---|---|---|---|---|---|
| **1213** | Q7Z417 | Nuclear fragile X mental retardatior | unknown | 8.20 | -22.32 | TRUE | TRUE | TRUE |
| **1214** | O14880 | Microsomal glutathione S-transfera | unknown | 27.41 | -12.93 | FALSE | TRUE | TRUE |
| **1215** | Q92734 | Protein TFG | CYTOSOL | 15.58 | 23.02 | FALSE | TRUE | TRUE |
| **1216** | O75608 | Acyl-protein thioesterase 1 | unknown | 20.01 | -2.98 | FALSE | TRUE | TRUE |
| **1217** | Q9Y3A6 | Transmembrane emp24 domain-cc | unknown | -12.93 | 31.75 | FALSE | TRUE | FALSE |
| **1218** | Q9H299 | SH3 domain-binding glutamic acid-r | unknown | 14.54 | 16.32 | FALSE | TRUE | TRUE |
| **1219** | P36957 | Dihydrolipoyllysine-residue succiny | MITOCHONDRIA | 39.69 | -9.46 | FALSE | TRUE | TRUE |
| **1220** | Q8NF37 | Lysophosphatidylcholine acyltransf | unknown | -9.44 | 29.62 | FALSE | TRUE | TRUE |
| **1221** | Q14203 | Dynactin subunit 1 | unknown | 9.89 | 0.96 | FALSE | TRUE | TRUE |
| **1222** | Q15021 | Condensin complex subunit 1 | unknown | -7.93 | -9.12 | TRUE | TRUE | TRUE |
| **1223** | Q9UI30 | Multifunctional methyltransferase s | unknown | 11.29 | 4.06 | FALSE | TRUE | TRUE |
| **1224** | Q9Y285 | Phenylalanine--tRNA ligase alpha su | unknown | -4.90 | -10.90 | FALSE | TRUE | TRUE |
| **1225** | P08574 | Cytochrome c1, heme protein, mito | MITOCHONDRIA | 32.89 | -11.13 | FALSE | TRUE | TRUE |
| **1226** | Q6PIU2 | Neutral cholesterol ester hydrolase | ER | -2.93 | 32.05 | FALSE | TRUE | TRUE |
| **1227** | Q13885 | Tubulin beta-2A chain | unknown | 12.04 | 22.35 | FALSE | FALSE | TRUE |
| **1228** | P69905 | Hemoglobin subunit alpha | unknown | -13.83 | -8.97 | FALSE | TRUE | FALSE |
| **1229** | Q9BZG1 | Ras-related protein Rab-34 | unknown | -20.90 | 19.89 | FALSE | TRUE | TRUE |
| **1230** | Q6L8Q7 | 2',5'-phosphodiesterase 12 | unknown | 21.13 | -3.73 | FALSE | TRUE | TRUE |
| **1231** | Q9NSE4 | Isoleucine--tRNA ligase, mitochondr | MITOCHONDRIA | 38.16 | -8.36 | FALSE | TRUE | TRUE |
| **1232** | O95336 | 6-phosphogluconolactonase | CYTOSOL | 19.10 | 24.50 | FALSE | TRUE | FALSE |
| **1233** | Q9Y2R4 | Probable ATP-dependent RNA helic | NUCLEUS | -0.20 | -40.08 | FALSE | TRUE | FALSE |
| **1234** | Q00535 | Cyclin-dependent-like kinase 5 | unknown | -11.95 | 14.57 | FALSE | TRUE | FALSE |
| **1235** | Q9Y5K5 | Ubiquitin carboxyl-terminal hydrola | NUCLEUS | -14.91 | -28.63 | FALSE | TRUE | TRUE |
| **1236** | Q9NYY8 | FAST kinase domain-containing pro | MITOCHONDRIA | 42.93 | -13.95 | FALSE | TRUE | TRUE |
| **1237** | O75822 | Eukaryotic translation initiation fact | unknown | 13.41 | -10.96 | FALSE | TRUE | TRUE |
| **1238** | Q9H9Y6 | DNA-directed RNA polymerase I sul | NUCLEUS | -24.73 | -26.12 | FALSE | TRUE | TRUE |
| **1239** | Q15165 | Serum paraoxonase/arylesterase 2 | unknown | 3.24 | 33.76 | FALSE | TRUE | TRUE |
| **1240** | Q53EL6 | Programmed cell death protein 4 | unknown | 14.13 | -10.74 | TRUE | TRUE | TRUE |
| **1241** | Q99567 | Nuclear pore complex protein Nup8 | unknown | -6.70 | -22.66 | TRUE | TRUE | TRUE |
| **1242** | O43324 | Eukaryotic translation elongation fa | unknown | 5.70 | -3.29 | FALSE | TRUE | TRUE |
| **1243** | Q9Y4W2 | Ribosomal biogenesis protein LAS1I | NUCLEUS | 0.83 | -34.63 | FALSE | TRUE | TRUE |
| **1244** | Q9H845 | Acyl-CoA dehydrogenase family me | MITOCHONDRIA | 37.88 | -14.34 | FALSE | TRUE | TRUE |
| **1245** | Q9UHB9 | Signal recognition particle subunit S | unknown | 5.34 | -4.02 | FALSE | TRUE | TRUE |
| **1246** | Q00688 | Peptidyl-prolyl cis-trans isomerase I | RIBOSOME 60S | 16.32 | -22.03 | FALSE | TRUE | TRUE |
| **1247** | P04183 | Thymidine kinase, cytosolic | unknown | -3.27 | -8.31 | FALSE | TRUE | TRUE |
| **1248** | P48637 | Glutathione synthetase | CYTOSOL | 15.34 | 19.91 | FALSE | TRUE | TRUE |
| **1249** | Q9BSH4 | Translational activator of cytochron | unknown | 35.12 | -1.79 | FALSE | TRUE | FALSE |
| **1250** | Q9NR45 | Sialic acid synthase | CYTOSOL | 20.42 | 9.45 | FALSE | TRUE | FALSE |

| | | | | | | | | |
|---|---|---|---|---|---|---|---|---|
| **1251** | O95163 | Elongator complex protein 1 | unknown | 14.57 | 8.19 | FALSE | TRUE | TRUE |
| **1252** | O15121 | Sphingolipid delta(4)-desaturase DE | unknown | -7.71 | 32.26 | FALSE | TRUE | FALSE |
| **1253** | Q86XP3 | ATP-dependent RNA helicase DDX4 | NUCLEUS | -7.22 | -29.68 | TRUE | TRUE | TRUE |
| **1254** | O60749 | Sorting nexin-2 | unknown | -10.88 | 16.58 | TRUE | TRUE | TRUE |
| **1255** | O00186 | Syntaxin-binding protein 3 | unknown | -21.12 | 18.15 | TRUE | TRUE | TRUE |
| **1256** | O15498 | Synaptobrevin homolog YKT6 | unknown | 3.74 | 9.81 | TRUE | TRUE | TRUE |
| **1257** | O75616 | GTPase Era, mitochondrial | MITOCHONDRIA | 35.96 | -14.16 | FALSE | TRUE | TRUE |
| **1258** | Q9NX20 | 39S ribosomal protein L16, mitocho | MITOCHONDRIA | 43.14 | -5.62 | FALSE | TRUE | FALSE |
| **1259** | P02794 | Ferritin heavy chain | unknown | -36.38 | 8.89 | FALSE | TRUE | TRUE |
| **1260** | Q9UBS4 | DnaJ homolog subfamily B member | ER | -5.29 | 35.10 | FALSE | TRUE | TRUE |
| **1261** | O43290 | U4/U6.U5 tri-snRNP-associated pro | NUCLEUS | 3.08 | -33.52 | TRUE | TRUE | TRUE |
| **1262** | Q96E11 | Ribosome-recycling factor, mitocho | unknown | 43.12 | -2.53 | FALSE | TRUE | TRUE |
| **1263** | Q9P289 | Serine/threonine-protein kinase 26 | unknown | 1.69 | 10.76 | FALSE | TRUE | TRUE |
| **1264** | P13798 | Acylamino-acid-releasing enzyme | CYTOSOL | 14.84 | 18.99 | FALSE | TRUE | FALSE |
| **1265** | O00203 | AP-3 complex subunit beta-1 | unknown | -12.93 | 3.51 | FALSE | TRUE | TRUE |
| **1266** | P24928 | DNA-directed RNA polymerase II su | NUCLEUS | -17.72 | -28.31 | FALSE | TRUE | TRUE |
| **1267** | O94979 | Protein transport protein Sec31A | unknown | 8.58 | -13.69 | FALSE | TRUE | TRUE |
| **1268** | P63279 | SUMO-conjugating enzyme UBC9 | NUCLEUS | -3.95 | -15.89 | FALSE | TRUE | TRUE |
| **1269** | P14735 | Insulin-degrading enzyme | unknown | 9.60 | 8.61 | FALSE | TRUE | TRUE |
| **1270** | Q06203 | Amidophosphoribosyltransferase | unknown | 14.74 | 17.61 | FALSE | TRUE | TRUE |
| **1271** | P09525 | Annexin A4 | unknown | 13.76 | 18.13 | FALSE | TRUE | TRUE |
| **1272** | Q9HAV7 | GrpE protein homolog 1, mitochond | unknown | 44.83 | -12.50 | FALSE | TRUE | TRUE |
| **1273** | Q9NVJ2 | ADP-ribosylation factor-like protein | unknown | -41.10 | 9.13 | FALSE | TRUE | TRUE |
| **1274** | Q9H2G2 | STE20-like serine/threonine-protein | unknown | 1.45 | 10.53 | TRUE | TRUE | TRUE |
| **1275** | Q8IV08 | Phospholipase D3 | unknown | -40.79 | 8.40 | FALSE | TRUE | FALSE |
| **1276** | P14324 | Farnesyl pyrophosphate synthase | CYTOSOL | 17.34 | 15.07 | FALSE | TRUE | TRUE |
| **1277** | Q13561 | Dynactin subunit 2 | unknown | 10.25 | 1.89 | FALSE | TRUE | TRUE |
| **1278** | P63165 | Small ubiquitin-related modifier 1 | NUCLEUS | -20.53 | -32.93 | TRUE | TRUE | FALSE |
| **1279** | Q13618 | Cullin-3 | NUCLEUS | -2.47 | -13.17 | FALSE | TRUE | TRUE |
| **1280** | Q16555 | Dihydropyrimidinase-related protei | unknown | 9.12 | 18.47 | FALSE | TRUE | TRUE |
| **1281** | P48507 | Glutamate--cysteine ligase regulato | unknown | 15.60 | 7.67 | FALSE | TRUE | TRUE |
| **1282** | P08133 | Annexin A6 | unknown | 9.24 | 13.94 | FALSE | TRUE | TRUE |
| **1283** | Q9Y314 | Nitric oxide synthase-interacting pro | NUCLEUS | -3.85 | -16.40 | FALSE | TRUE | TRUE |
| **1284** | Q8WVX9 | Fatty acyl-CoA reductase 1 | PEROXISOME | 53.02 | -3.56 | FALSE | TRUE | FALSE |
| **1285** | Q13564 | NEDD8-activating enzyme E1 regul | unknown | 25.02 | 18.68 | FALSE | TRUE | TRUE |
| **1286** | Q96FJ2 | Dynein light chain 2, cytoplasmic | NUCLEUS | -11.17 | -17.88 | FALSE | TRUE | TRUE |
| **1287** | Q9H0U3 | Magnesium transporter protein 1 | unknown | 6.67 | 35.06 | FALSE | TRUE | TRUE |
| **1288** | Q9NVI7 | ATPase family AAA domain-contain | MITOCHONDRIA | 29.73 | -11.69 | FALSE | TRUE | TRUE |

| | | | | | | | | |
|---|---|---|---|---|---|---|---|---|
| **1289** | Q14914 | Prostaglandin reductase 1 | unknown | 24.00 | 22.53 | FALSE | TRUE | TRUE |
| **1290** | Q9Y2B0 | Protein canopy homolog 2 | ER | -4.55 | 36.48 | FALSE | TRUE | TRUE |
| **1291** | Q99439 | Calponin-2 | unknown | 3.93 | 3.83 | TRUE | TRUE | TRUE |
| **1292** | Q86TI2 | Dipeptidyl peptidase 9 | unknown | 4.96 | -0.11 | FALSE | TRUE | TRUE |
| **1293** | Q9BW27 | Nuclear pore complex protein Nup8 | NUCLEUS | -19.63 | -29.81 | FALSE | TRUE | TRUE |
| **1294** | P50213 | Isocitrate dehydrogenase [NAD] su | MITOCHONDRIA | 41.50 | -7.48 | FALSE | TRUE | TRUE |
| **1295** | Q15070 | Mitochondrial inner membrane pro | MITOCHONDRIA | 35.23 | -15.49 | FALSE | TRUE | TRUE |
| **1296** | Q5SY16 | Polynucleotide 5'-hydroxyl-kinase N | NUCLEUS | 0.59 | -34.55 | FALSE | TRUE | FALSE |
| **1297** | Q9NYK5 | 39S ribosomal protein L39, mitocho | MITOCHONDRIA | 41.87 | -11.54 | FALSE | TRUE | TRUE |
| **1298** | Q9Y2P8 | RNA 3'-terminal phosphate cyclase- | NUCLEUS | -2.30 | -39.64 | FALSE | TRUE | TRUE |
| **1299** | P48739 | Phosphatidylinositol transfer protei | unknown | 21.35 | 14.68 | FALSE | TRUE | TRUE |
| **1300** | P10606 | Cytochrome c oxidase subunit 5B, m | MITOCHONDRIA | 33.73 | -11.27 | FALSE | TRUE | TRUE |
| **1301** | P36404 | ADP-ribosylation factor-like protein | unknown | 19.85 | -2.86 | FALSE | TRUE | FALSE |
| **1302** | P22059 | Oxysterol-binding protein 1 | unknown | 2.29 | 9.82 | FALSE | TRUE | TRUE |
| **1303** | P14923 | Junction plakoglobin | PM | -26.54 | 5.99 | TRUE | TRUE | TRUE |
| **1304** | P01130 | Low-density lipoprotein receptor | unknown | -34.17 | 8.07 | FALSE | TRUE | TRUE |
| **1305** | O95453 | Poly(A)-specific ribonuclease PARN | NUCLEUS | -17.49 | -29.46 | FALSE | TRUE | TRUE |
| **1306** | Q9GZR2 | RNA exonuclease 4 | NUCLEUS | -0.86 | -38.50 | FALSE | TRUE | TRUE |
| **1307** | Q13131 | 5'-AMP-activated protein kinase ca | unknown | 6.75 | 8.33 | FALSE | TRUE | FALSE |
| **1308** | P18858 | DNA ligase 1 | unknown | 16.18 | 2.90 | TRUE | TRUE | TRUE |
| **1309** | Q9Y221 | 60S ribosome subunit biogenesis pr | NUCLEUS | -3.47 | -35.54 | FALSE | TRUE | FALSE |
| **1310** | Q969S3 | Zinc finger protein 622 | unknown | 3.25 | -18.25 | FALSE | TRUE | TRUE |
| **1311** | Q6P1A2 | Lysophospholipid acyltransferase 5 | unknown | 0.04 | 32.84 | FALSE | TRUE | TRUE |
| **1312** | Q9NZB2 | Constitutive coactivator of PPAR-ga | unknown | -0.05 | -22.64 | TRUE | TRUE | TRUE |
| **1313** | Q9UGP8 | Translocation protein SEC63 homol | ER | 4.39 | 37.18 | FALSE | TRUE | TRUE |
| **1314** | Q9NR09 | Baculoviral IAP repeat-containing p | unknown | 8.76 | 1.93 | TRUE | TRUE | TRUE |
| **1315** | Q5T653 | 39S ribosomal protein L2, mitochon | MITOCHONDRIA | 38.95 | -4.40 | FALSE | TRUE | TRUE |
| **1316** | Q03135 | Caveolin-1 | unknown | -19.57 | 16.07 | FALSE | TRUE | TRUE |
| **1317** | Q8WXX5 | DnaJ homolog subfamily C member | unknown | -12.64 | -8.35 | FALSE | TRUE | TRUE |
| **1318** | O00178 | GTP-binding protein 1 | unknown | 5.12 | -18.44 | FALSE | TRUE | FALSE |
| **1319** | Q9Y696 | Chloride intracellular channel protei | unknown | 2.37 | 11.58 | FALSE | TRUE | TRUE |
| **1320** | Q15796 | Mothers against decapentaplegic h | unknown | -1.36 | -12.75 | FALSE | TRUE | FALSE |
| **1321** | Q9NVH1 | DnaJ homolog subfamily C member | unknown | 27.78 | -13.06 | FALSE | TRUE | TRUE |
| **1322** | Q9UH17 | DNA dC->dU-editing enzyme APOB | NUCLEUS | -10.79 | -36.51 | FALSE | TRUE | FALSE |
| **1323** | Q9HC21 | Mitochondrial thiamine pyrophosph | MITOCHONDRIA | 34.42 | -13.56 | FALSE | TRUE | FALSE |
| **1324** | Q9UBQ5 | Eukaryotic translation initiation fact | unknown | 14.34 | -10.63 | FALSE | TRUE | FALSE |
| **1325** | O00743 | Serine/threonine-protein phosphata | unknown | 13.92 | 12.35 | FALSE | TRUE | TRUE |
| **1326** | Q9UHI6 | Probable ATP-dependent RNA helic | NUCLEUS | -0.86 | -22.46 | FALSE | TRUE | TRUE |

| | | | | | | | | |
|---|---|---|---|---|---|---|---|---|
| **1327** | O60313 | Dynamin-like 120 kDa protein, mitc | MITOCHONDRIA | 35.35 | -5.03 | FALSE | TRUE | TRUE |
| **1328** | Q9Y3C6 | Peptidyl-prolyl cis-trans isomerase- | NUCLEUS | -9.98 | -30.59 | FALSE | TRUE | TRUE |
| **1329** | Q9NR12 | PDZ and LIM domain protein 7 | unknown | -6.22 | -1.14 | TRUE | TRUE | FALSE |
| **1330** | P78310 | Coxsackievirus and adenovirus rece | PM | -28.38 | 7.62 | TRUE | FALSE | TRUE |
| **1331** | Q9Y673 | Dolichyl-phosphate beta-glucosyltr | ER | -3.19 | 33.01 | FALSE | TRUE | FALSE |
| **1332** | Q9BYD1 | 39S ribosomal protein L13, mitocho | MITOCHONDRIA | 43.00 | -6.10 | FALSE | TRUE | FALSE |
| **1333** | P24539 | ATP synthase F(0) complex subunit | MITOCHONDRIA | 37.00 | -14.31 | FALSE | TRUE | TRUE |
| **1334** | Q7L5N1 | COP9 signalosome complex subuni | unknown | 17.96 | 13.45 | FALSE | TRUE | FALSE |
| **1335** | P61009 | Signal peptidase complex subunit 3 | ER | 5.86 | 36.43 | FALSE | TRUE | TRUE |
| **1336** | P53701 | Cytochrome c-type heme lyase | unknown | 39.69 | -12.64 | FALSE | TRUE | TRUE |
| **1337** | Q9BZZ5 | Apoptosis inhibitor 5 | NUCLEUS | -11.05 | -23.69 | FALSE | TRUE | TRUE |
| **1338** | Q13637 | Ras-related protein Rab-32 | unknown | -11.86 | 20.35 | FALSE | TRUE | TRUE |
| **1339** | O60563 | Cyclin-T1 | NUCLEUS | 3.99 | -31.45 | FALSE | TRUE | TRUE |
| **1340** | Q14677 | Clathrin interactor 1 | unknown | -13.78 | 27.80 | TRUE | TRUE | TRUE |
| **1341** | O43292 | Glycosylphosphatidylinositol ancho | ER | -4.48 | 27.25 | FALSE | TRUE | FALSE |
| **1342** | A3KMH1 | von Willebrand factor A domain-co | MITOCHONDRIA | 32.64 | -12.22 | TRUE | TRUE | TRUE |
| **1343** | Q92643 | GPI-anchor transamidase | ER | 2.00 | 32.63 | FALSE | TRUE | FALSE |
| **1344** | O75521 | Enoyl-CoA delta isomerase 2, mitoc | PEROXISOME | 51.80 | -3.77 | FALSE | TRUE | TRUE |
| **1345** | Q9Y450 | HBS1-like protein | NUCLEUS | -0.98 | -5.15 | TRUE | TRUE | TRUE |
| **1346** | Q8TEX9 | Importin-4 | unknown | 13.91 | 9.49 | FALSE | TRUE | FALSE |
| **1347** | Q13546 | Receptor-interacting serine/threoni | unknown | 11.18 | 2.19 | FALSE | TRUE | TRUE |
| **1348** | Q14146 | Unhealthy ribosome biogenesis pro | NUCLEUS | -0.96 | -39.01 | FALSE | TRUE | TRUE |
| **1349** | Q9Y2W1 | Thyroid hormone receptor-associat | NUCLEUS | -2.10 | -25.70 | TRUE | TRUE | TRUE |
| **1350** | Q9BT22 | Chitobiosyldiphosphodolichol beta- | ER | -6.93 | 33.45 | FALSE | TRUE | TRUE |
| **1351** | P11233 | Ras-related protein Ral-A | PM | -26.43 | 13.24 | TRUE | TRUE | TRUE |
| **1352** | O60256 | Phosphoribosyl pyrophosphate syn | unknown | 4.75 | -0.41 | FALSE | TRUE | TRUE |
| **1353** | Q15031 | Probable leucine--tRNA ligase, mito | MITOCHONDRIA | 41.24 | -9.13 | FALSE | TRUE | TRUE |
| **1354** | Q9BRX2 | Protein pelota homolog | unknown | -4.08 | -10.21 | FALSE | TRUE | TRUE |
| **1355** | Q8N6H7 | ADP-ribosylation factor GTPase-act | NUCLEUS | -10.39 | -16.21 | TRUE | TRUE | FALSE |
| **1356** | P06730 | Eukaryotic translation initiation fact | unknown | 9.94 | -3.31 | FALSE | TRUE | TRUE |
| **1357** | P49419 | Alpha-aminoadipic semialdehyde d | unknown | 22.23 | -3.89 | FALSE | TRUE | TRUE |
| **1358** | Q96L92 | Sorting nexin-27 | unknown | -10.59 | 9.83 | FALSE | TRUE | TRUE |
| **1359** | O60343 | TBC1 domain family member 4 | unknown | 1.38 | -1.70 | FALSE | TRUE | TRUE |
| **1360** | P10586 | Receptor-type tyrosine-protein pho | PM | -24.25 | 12.38 | FALSE | TRUE | TRUE |
| **1361** | P54105 | Methylosome subunit pICln | CYTOSOL | 18.18 | 21.81 | FALSE | TRUE | TRUE |
| **1362** | P17301 | Integrin alpha-2 | PM | -23.17 | 16.65 | TRUE | TRUE | TRUE |
| **1363** | Q99661 | Kinesin-like protein KIF2C | unknown | -14.98 | -17.08 | FALSE | TRUE | TRUE |
| **1364** | P01111 | GTPase NRas | unknown | -28.94 | 1.81 | FALSE | TRUE | TRUE |

| | | | | | | | | |
|---|---|---|---|---|---|---|---|---|
| **1365** | Q16795 | NADH dehydrogenase [ubiquinone] | MITOCHONDRIA | 30.72 | -14.88 | FALSE | TRUE | FALSE |
| **1366** | P42126 | Enoyl-CoA delta isomerase 1, mitoc | MITOCHONDRIA | 32.51 | -8.66 | FALSE | TRUE | FALSE |
| **1367** | Q01581 | Hydroxymethylglutaryl-CoA syntha | CYTOSOL | 24.06 | 23.47 | FALSE | TRUE | TRUE |
| **1368** | Q5BKZ1 | DBIRD complex subunit ZNF326 | NUCLEUS | -11.05 | -36.28 | FALSE | TRUE | TRUE |
| **1369** | O75152 | Zinc finger CCCH domain-containing | unknown | -11.15 | -23.37 | TRUE | TRUE | TRUE |
| **1370** | Q9NWU5 | 39S ribosomal protein L22, mitocho | MITOCHONDRIA | 43.11 | -5.92 | FALSE | TRUE | TRUE |
| **1371** | Q15459 | Splicing factor 3A subunit 1 | NUCLEUS | 5.01 | -34.84 | TRUE | TRUE | TRUE |
| **1372** | Q9Y3D6 | Mitochondrial fission 1 protein | unknown | -0.19 | 23.86 | FALSE | TRUE | TRUE |
| **1373** | Q15785 | Mitochondrial import receptor subu | unknown | 6.95 | -6.08 | FALSE | TRUE | TRUE |
| **1374** | P15374 | Ubiquitin carboxyl-terminal hydrola | CYTOSOL | 23.78 | 24.10 | FALSE | TRUE | TRUE |
| **1375** | Q9BWD1 | Acetyl-CoA acetyltransferase, cytos | CYTOSOL | 19.09 | 23.46 | FALSE | TRUE | TRUE |
| **1376** | Q14C86 | GTPase-activating protein and VPS9 | unknown | 8.75 | 5.13 | TRUE | TRUE | TRUE |
| **1377** | P14854 | Cytochrome c oxidase subunit 6B1 | unknown | 29.27 | -7.73 | FALSE | TRUE | TRUE |
| **1378** | Q6IBS0 | Twinfilin-2 | unknown | 9.64 | 4.97 | FALSE | TRUE | FALSE |
| **1379** | P18031 | Tyrosine-protein phosphatase non- | ER | 1.81 | 33.82 | TRUE | TRUE | TRUE |
| **1380** | Q96TA1 | Niban-like protein 1 | unknown | 0.17 | 10.25 | TRUE | TRUE | TRUE |
| **1381** | Q9NW64 | Pre-mRNA-splicing factor RBM22 | NUCLEUS | -2.94 | -39.09 | TRUE | TRUE | TRUE |
| **1382** | P35251 | Replication factor C subunit 1 | NUCLEUS-CHROMATIN | -21.93 | -15.19 | TRUE | TRUE | TRUE |
| **1383** | Q5M775 | Cytospin-B | unknown | -18.33 | 14.79 | TRUE | TRUE | TRUE |
| **1384** | O75116 | Rho-associated protein kinase 2 | unknown | -9.13 | 11.47 | TRUE | TRUE | TRUE |
| **1385** | Q9NY93 | Probable ATP-dependent RNA helic | NUCLEUS | -0.50 | -37.76 | FALSE | TRUE | TRUE |
| **1386** | P17174 | Aspartate aminotransferase, cytopl | CYTOSOL | 20.24 | 24.75 | FALSE | TRUE | TRUE |
| **1387** | Q01085 | Nucleolysin TIAR | NUCLEUS | -20.32 | -32.91 | FALSE | TRUE | TRUE |
| **1388** | P11169 | Solute carrier family 2, facilitated gl | PM | -24.99 | 8.74 | FALSE | TRUE | TRUE |
| **1389** | Q9Y606 | tRNA pseudouridine synthase A | MITOCHONDRIA | 33.40 | -5.95 | FALSE | TRUE | FALSE |
| **1390** | Q15629 | Translocating chain-associated mem | unknown | 5.37 | 38.41 | FALSE | TRUE | TRUE |
| **1391** | O43847 | Nardilysin | CYTOSOL | 18.00 | 16.41 | FALSE | TRUE | TRUE |
| **1392** | P43121 | Cell surface glycoprotein MUC18 | PM | -27.03 | 9.83 | TRUE | TRUE | TRUE |
| **1393** | Q9NPD3 | Exosome complex component RRP4 | NUCLEUS | -7.61 | -24.51 | FALSE | TRUE | TRUE |
| **1394** | P56199 | Integrin alpha-1 | PM | -25.47 | 9.47 | TRUE | TRUE | TRUE |
| **1395** | Q6WKZ4 | Rab11 family-interacting protein 1 | unknown | -21.61 | 5.50 | TRUE | TRUE | TRUE |
| **1396** | Q96I24 | Far upstream element-binding prot | NUCLEUS | -12.60 | -35.96 | TRUE | TRUE | TRUE |
| **1397** | Q9Y6G9 | Cytoplasmic dynein 1 light intermed | unknown | -11.32 | -7.40 | TRUE | TRUE | TRUE |
| **1398** | P62487 | DNA-directed RNA polymerase II su | NUCLEUS | -16.76 | -29.54 | FALSE | TRUE | FALSE |
| **1399** | P61964 | WD repeat-containing protein 5 | unknown | -15.92 | -22.68 | FALSE | TRUE | TRUE |
| **1400** | O00592 | Podocalyxin | unknown | -28.60 | 7.13 | TRUE | TRUE | TRUE |
| **1401** | Q9BXB5 | Oxysterol-binding protein-related p | unknown | -7.02 | 3.83 | TRUE | TRUE | TRUE |
| **1402** | Q9NVM9 | Integrator complex subunit 13 | NUCLEUS | -16.05 | -21.89 | FALSE | TRUE | TRUE |

| | | | | | | | | |
|---|---|---|---|---|---|---|---|---|
| **1403** | O00566 | U3 small nucleolar ribonucleoprotei | NUCLEUS | -0.64 | -40.52 | FALSE | TRUE | TRUE |
| **1404** | P61966 | AP-1 complex subunit sigma-1A | unknown | -33.39 | 19.53 | FALSE | TRUE | TRUE |
| **1405** | Q9NUJ1 | Mycophenolic acid acyl-glucuronide | MITOCHONDRIA | 43.44 | -7.14 | FALSE | TRUE | TRUE |
| **1406** | O60216 | Double-strand-break repair protein | NUCLEUS-CHROMATIN | -18.40 | -16.08 | FALSE | TRUE | TRUE |
| **1407** | Q71RC2 | La-related protein 4 | unknown | 6.83 | -18.79 | TRUE | TRUE | TRUE |
| **1408** | Q9BUL8 | Programmed cell death protein 10 | unknown | 1.39 | 10.63 | FALSE | TRUE | TRUE |
| **1409** | A3KN83 | Protein strawberry notch homolog 1 | NUCLEUS | -9.17 | -13.82 | FALSE | TRUE | TRUE |
| **1410** | Q92552 | 28S ribosomal protein S27, mitocho | unknown | 35.77 | -2.37 | FALSE | TRUE | TRUE |
| **1411** | P51151 | Ras-related protein Rab-9A | PM | -22.04 | 14.23 | FALSE | TRUE | FALSE |
| **1412** | Q7KZN9 | Cytochrome c oxidase assembly pro | MITOCHONDRIA | 35.30 | -15.13 | FALSE | TRUE | FALSE |
| **1413** | Q9NQW6 | Anillin | unknown | -10.57 | -10.09 | TRUE | TRUE | TRUE |
| **1414** | Q13868 | Exosome complex component RRP4 | NUCLEUS | -10.72 | -26.21 | FALSE | TRUE | FALSE |
| **1415** | O95202 | Mitochondrial proton/calcium excha | MITOCHONDRIA | 37.01 | -9.77 | FALSE | TRUE | TRUE |
| **1416** | Q96A26 | Protein FAM162A | MITOCHONDRIA | 35.85 | -11.05 | FALSE | TRUE | TRUE |
| **1417** | Q9BXP5 | Serrate RNA effector molecule hom | unknown | -15.70 | -31.24 | TRUE | TRUE | TRUE |
| **1418** | Q9P0V9 | Septin-10 | unknown | -1.91 | 4.51 | FALSE | TRUE | TRUE |
| **1419** | O15084 | Serine/threonine-protein phosphata | unknown | 9.88 | 5.18 | FALSE | TRUE | FALSE |
| **1420** | Q6P158 | Putative ATP-dependent RNA helica | unknown | 7.97 | -20.71 | FALSE | TRUE | TRUE |
| **1421** | P55010 | Eukaryotic translation initiation fact | unknown | 18.13 | 10.06 | FALSE | TRUE | TRUE |
| **1422** | Q96SU4 | Oxysterol-binding protein-related p | unknown | -0.25 | 9.43 | FALSE | TRUE | TRUE |
| **1423** | Q9NZL4 | Hsp70-binding protein 1 | unknown | 21.56 | 17.33 | FALSE | TRUE | TRUE |
| **1424** | P61927 | 60S ribosomal protein L37 | RIBOSOME 60S | 15.77 | -19.32 | TRUE | FALSE | TRUE |
| **1425** | O95071 | E3 ubiquitin-protein ligase UBR5 | unknown | 21.28 | 11.28 | FALSE | TRUE | TRUE |
| **1426** | Q9NP92 | 39S ribosomal protein S30, mitocho | unknown | 41.50 | -13.92 | FALSE | TRUE | TRUE |
| **1427** | P51553 | Isocitrate dehydrogenase [NAD] su | MITOCHONDRIA | 37.42 | -4.05 | FALSE | TRUE | FALSE |
| **1428** | O60869 | Endothelial differentiation-related f | unknown | 8.25 | -21.79 | TRUE | TRUE | FALSE |
| **1429** | P61201 | COP9 signalosome complex subunit | unknown | 17.06 | 8.07 | FALSE | TRUE | FALSE |
| **1430** | Q04206 | Transcription factor p65 | unknown | 15.45 | 5.62 | FALSE | TRUE | TRUE |
| **1431** | Q9Y2Q3 | Glutathione S-transferase kappa 1 | PEROXISOME | 53.10 | -3.70 | FALSE | TRUE | TRUE |
| **1432** | P78362 | SRSF protein kinase 2 | unknown | 6.67 | -24.62 | FALSE | TRUE | TRUE |
| **1433** | Q9Y3A4 | Ribosomal RNA-processing protein 7 | NUCLEUS | -0.42 | -41.16 | FALSE | TRUE | FALSE |
| **1434** | O14646 | Chromodomain-helicase-DNA-bindi | NUCLEUS | -22.79 | -23.55 | FALSE | TRUE | TRUE |
| **1435** | Q15428 | Splicing factor 3A subunit 2 | NUCLEUS | 5.00 | -34.83 | FALSE | TRUE | FALSE |
| **1436** | Q8NE86 | Calcium uniporter protein, mitochor | MITOCHONDRIA | 30.31 | -14.59 | FALSE | TRUE | TRUE |
| **1437** | Q96G03 | Phosphoglucomutase-2 | unknown | 25.26 | 21.78 | FALSE | TRUE | TRUE |
| **1438** | Q9H078 | Caseinolytic peptidase B protein hor | MITOCHONDRIA | 34.63 | -11.51 | FALSE | TRUE | TRUE |
| **1439** | Q96I51 | RCC1-like G exchanging factor-like p | MITOCHONDRIA | 34.82 | -8.32 | FALSE | TRUE | TRUE |
| **1440** | Q9H0D6 | 5'-3' exoribonuclease 2 | NUCLEUS | -6.79 | -31.32 | FALSE | TRUE | TRUE |

| | | | | | | | | |
|---|---|---|---|---|---|---|---|---|
| **1441** | P00390 | Glutathione reductase, mitochondri | unknown | 6.96 | -3.47 | FALSE | TRUE | TRUE |
| **1442** | Q6IA86 | Elongator complex protein 2 | unknown | 15.26 | 5.99 | FALSE | TRUE | TRUE |
| **1443** | Q8NBM4 | Ubiquitin-associated domain-contai | ER | -9.53 | 30.80 | FALSE | TRUE | TRUE |
| **1444** | Q9UJZ1 | Stomatin-like protein 2, mitochondr | MITOCHONDRIA | 35.88 | -8.71 | FALSE | TRUE | TRUE |
| **1445** | Q8WUY1 | Protein THEM6 | ER | -4.69 | 27.64 | FALSE | TRUE | TRUE |
| **1446** | P13995 | Bifunctional methylenetetrahydrofo | MITOCHONDRIA | 42.36 | -6.24 | FALSE | TRUE | TRUE |
| **1447** | Q16643 | Drebrin | unknown | 1.23 | 9.28 | TRUE | TRUE | TRUE |
| **1448** | P20810 | Calpastatin | unknown | 4.10 | -6.36 | TRUE | TRUE | TRUE |
| **1449** | Q9NQW7 | Xaa-Pro aminopeptidase 1 | CYTOSOL | 17.04 | 17.74 | FALSE | TRUE | TRUE |
| **1450** | Q16822 | Phosphoenolpyruvate carboxykinas | MITOCHONDRIA | 37.73 | -1.81 | FALSE | TRUE | FALSE |
| **1451** | O14908 | PDZ domain-containing protein GIP | unknown | -14.10 | 14.72 | FALSE | TRUE | TRUE |
| **1452** | Q8TC12 | Retinol dehydrogenase 11 | ER | -0.42 | 33.69 | FALSE | TRUE | TRUE |
| **1453** | O43813 | LanC-like protein 1 | unknown | -9.91 | 4.56 | FALSE | TRUE | TRUE |
| **1454** | Q99584 | Protein S100-A13 | NUCLEUS | -8.11 | -15.76 | FALSE | TRUE | TRUE |
| **1455** | Q96EK6 | Glucosamine 6-phosphate N-acetyl | NUCLEUS | -0.59 | -24.85 | FALSE | TRUE | TRUE |
| **1456** | P51809 | Vesicle-associated membrane prot | unknown | -39.00 | 7.90 | FALSE | TRUE | TRUE |
| **1457** | Q96BW9 | Phosphatidate cytidylyltransferase, | MITOCHONDRIA | 37.89 | -14.62 | FALSE | TRUE | FALSE |
| **1458** | Q96CS3 | FAS-associated factor 2 | ER | 1.52 | 33.27 | FALSE | TRUE | FALSE |
| **1459** | P23193 | Transcription elongation factor A pr | NUCLEUS | -5.47 | -15.72 | FALSE | TRUE | TRUE |
| **1460** | Q9NRX4 | 14 kDa phosphohistidine phosphata | CYTOSOL | 10.44 | 20.98 | FALSE | TRUE | TRUE |
| **1461** | P09496 | Clathrin light chain A | unknown | -10.08 | 13.85 | FALSE | TRUE | TRUE |
| **1462** | Q13330 | Metastasis-associated protein MTA | NUCLEUS | -22.20 | -26.81 | FALSE | TRUE | FALSE |
| **1463** | P62273 | 40S ribosomal protein S29 | RIBOSOME 40S | 11.00 | -24.40 | FALSE | TRUE | TRUE |
| **1464** | Q92905 | COP9 signalosome complex subunit | unknown | 21.78 | 9.89 | FALSE | TRUE | TRUE |
| **1465** | P06396 | Gelsolin | unknown | 13.01 | 16.32 | FALSE | TRUE | TRUE |
| **1466** | Q9BS26 | Endoplasmic reticulum resident pro | ER | -3.87 | 33.82 | FALSE | TRUE | TRUE |
| **1467** | Q99459 | Cell division cycle 5-like protein | NUCLEUS | -2.92 | -32.30 | TRUE | TRUE | TRUE |
| **1468** | Q14558 | Phosphoribosyl pyrophosphate syn | unknown | 0.00 | -5.79 | FALSE | TRUE | FALSE |
| **1469** | Q9UNF1 | Melanoma-associated antigen D2 | unknown | 11.30 | -2.86 | TRUE | TRUE | TRUE |
| **1470** | O75165 | DnaJ homolog subfamily C member | unknown | -35.26 | 15.93 | FALSE | TRUE | TRUE |
| **1471** | Q12872 | Splicing factor, suppressor of white- | NUCLEUS | 1.67 | -30.54 | TRUE | TRUE | TRUE |
| **1472** | O43795 | Unconventional myosin-Ib | unknown | -9.76 | 25.23 | FALSE | TRUE | FALSE |
| **1473** | Q00587 | Cdc42 effector protein 1 | unknown | -7.30 | 10.42 | TRUE | FALSE | TRUE |
| **1474** | Q06136 | 3-ketodihydrosphingosine reductas | unknown | -10.02 | 28.71 | FALSE | TRUE | FALSE |
| **1475** | Q96S97 | Myeloid-associated differentiation | PM | -28.17 | 1.48 | FALSE | TRUE | TRUE |
| **1476** | Q9Y4C2 | TRPM8 channel-associated factor 1 | unknown | 4.36 | 2.83 | FALSE | TRUE | TRUE |
| **1477** | Q8NCW5 | NAD(P)H-hydrate epimerase | unknown | 19.80 | -2.94 | FALSE | TRUE | FALSE |
| **1478** | Q9H1E3 | Nuclear ubiquitous casein and cyclir | unknown | 24.90 | 25.03 | FALSE | TRUE | TRUE |

| | | | | | | | | |
|---|---|---|---|---|---|---|---|---|
| **1479** | O43395 | U4/U6 small nuclear ribonucleoprot | NUCLEUS | 3.34 | -36.04 | FALSE | TRUE | TRUE |
| **1480** | P61758 | Prefoldin subunit 3 | unknown | 21.93 | 23.42 | FALSE | TRUE | TRUE |
| **1481** | Q8N357 | Solute carrier family 35 member F6 | unknown | -37.40 | 13.86 | FALSE | TRUE | TRUE |
| **1482** | Q03519 | Antigen peptide transporter 2 | unknown | -6.22 | 32.73 | FALSE | TRUE | FALSE |
| **1483** | Q9Y224 | RNA transcription, translation and tr | unknown | 5.63 | -25.04 | FALSE | TRUE | TRUE |
| **1484** | Q10570 | Cleavage and polyadenylation spec | NUCLEUS | -10.02 | -35.96 | FALSE | TRUE | TRUE |
| **1485** | Q9NT62 | Ubiquitin-like-conjugating enzyme | unknown | 16.59 | 8.89 | FALSE | TRUE | TRUE |
| **1486** | Q13630 | GDP-L-fucose synthase | CYTOSOL | 19.14 | 24.43 | FALSE | TRUE | TRUE |
| **1487** | Q9NP79 | Vacuolar protein sorting-associated | unknown | 14.56 | 12.64 | FALSE | TRUE | FALSE |
| **1488** | Q9UBB4 | Ataxin-10 | unknown | 17.37 | 13.00 | FALSE | TRUE | TRUE |
| **1489** | Q8TB61 | Adenosine 3'-phospho 5'-phosphos | unknown | -21.82 | 32.35 | FALSE | TRUE | TRUE |
| **1490** | O75179 | Ankyrin repeat domain-containing | unknown | 5.11 | -19.10 | TRUE | TRUE | TRUE |
| **1491** | P49790 | Nuclear pore complex protein Nup1 | NUCLEUS | -20.70 | -28.96 | TRUE | TRUE | TRUE |
| **1492** | P61421 | V-type proton ATPase subunit d 1 | unknown | -40.53 | 9.64 | FALSE | TRUE | TRUE |
| **1493** | Q9UJW0 | Dynactin subunit 4 | unknown | 9.45 | 1.36 | FALSE | TRUE | TRUE |
| **1494** | Q6UWP7 | Lysocardiolipin acyltransferase 1 | unknown | -8.26 | 31.01 | FALSE | TRUE | FALSE |
| **1495** | P21359 | Neurofibromin | unknown | 2.60 | -11.72 | FALSE | TRUE | TRUE |
| **1496** | P38432 | Coilin | NUCLEUS | -19.36 | -22.89 | TRUE | TRUE | TRUE |
| **1497** | Q9UBQ7 | Glyoxylate reductase/hydroxypyru | unknown | 20.46 | -3.19 | FALSE | TRUE | TRUE |
| **1498** | Q9UKX7 | Nuclear pore complex protein Nup5 | unknown | -16.10 | -31.27 | TRUE | TRUE | TRUE |
| **1499** | Q9Y3T9 | Nucleolar complex protein 2 homol | NUCLEUS | 0.16 | -38.95 | TRUE | TRUE | TRUE |
| **1500** | O76003 | Glutaredoxin-3 | CYTOSOL | 23.64 | 21.51 | FALSE | TRUE | TRUE |
| **1501** | Q99426 | Tubulin-folding cofactor B | unknown | 23.49 | 23.66 | TRUE | TRUE | TRUE |
| **1502** | Q96G21 | U3 small nucleolar ribonucleoprotei | NUCLEUS | -1.11 | -40.23 | FALSE | TRUE | TRUE |
| **1503** | P50552 | Vasodilator-stimulated phosphopro | unknown | -5.41 | 10.33 | TRUE | TRUE | TRUE |
| **1504** | Q99543 | DnaJ homolog subfamily C membe | NUCLEUS | -0.73 | -22.40 | FALSE | TRUE | TRUE |
| **1505** | Q14318 | Peptidyl-prolyl cis-trans isomerase | ER | -5.20 | 32.66 | FALSE | TRUE | TRUE |
| **1506** | O75419 | Cell division control protein 45 hom | unknown | 4.51 | 2.78 | FALSE | TRUE | FALSE |
| **1507** | O15347 | High mobility group protein B3 | NUCLEUS | -22.42 | -23.14 | FALSE | TRUE | TRUE |
| **1508** | O00443 | Phosphatidylinositol 4-phosphate 3 | unknown | 8.14 | -20.54 | FALSE | TRUE | TRUE |
| **1509** | Q9P0J0 | NADH dehydrogenase [ubiquinone] | MITOCHONDRIA | 30.66 | -11.04 | FALSE | TRUE | FALSE |
| **1510** | Q8TAD8 | Smad nuclear-interacting protein 1 | NUCLEUS | -2.37 | -22.87 | FALSE | TRUE | TRUE |
| **1511** | Q9UBU8 | Mortality factor 4-like protein 1 | NUCLEUS-CHROMATIN | -17.85 | -18.20 | FALSE | TRUE | TRUE |
| **1512** | Q16850 | Lanosterol 14-alpha demethylase | ER | -7.21 | 32.66 | FALSE | TRUE | TRUE |
| **1513** | O60701 | UDP-glucose 6-dehydrogenase | unknown | 17.57 | 6.96 | FALSE | TRUE | TRUE |
| **1514** | Q92620 | Pre-mRNA-splicing factor ATP-depe | NUCLEUS | -3.26 | -16.13 | FALSE | TRUE | TRUE |
| **1515** | Q15024 | Exosome complex component RRP4 | NUCLEUS | -10.67 | -26.23 | FALSE | TRUE | FALSE |
| **1516** | O00267 | Transcription elongation factor SPT | NUCLEUS | -16.56 | -30.60 | FALSE | TRUE | TRUE |

| | | | | | | | | |
|---|---|---|---|---|---|---|---|---|
| **1517** | P35221 | Catenin alpha-1 | PM | -24.77 | 9.80 | FALSE | TRUE | TRUE |
| **1518** | O60934 | Nibrin | unknown | -16.03 | -18.52 | FALSE | TRUE | TRUE |
| **1519** | P61962 | DDB1- and CUL4-associated factor 7 | NUCLEUS | -0.13 | -22.49 | FALSE | TRUE | FALSE |
| **1520** | Q9NX63 | MICOS complex subunit MIC19 | unknown | 27.39 | -11.22 | FALSE | TRUE | TRUE |
| **1521** | Q15043 | Zinc transporter ZIP14 | unknown | -35.14 | 16.28 | TRUE | TRUE | TRUE |
| **1522** | Q15257 | Serine/threonine-protein phosphata | unknown | 24.87 | 22.40 | FALSE | TRUE | TRUE |
| **1523** | P53350 | Serine/threonine-protein kinase PL | unknown | -12.12 | -11.30 | FALSE | TRUE | TRUE |
| **1524** | Q49A26 | Putative oxidoreductase GLYR1 | NUCLEUS-CHROMATIN | -21.48 | -13.05 | FALSE | TRUE | FALSE |
| **1525** | Q5T6V5 | Queuosine salvage protein | unknown | 24.01 | 23.76 | FALSE | TRUE | TRUE |
| **1526** | P36639 | 7,8-dihydro-8-oxoguanine triphosph | unknown | 16.06 | 11.58 | FALSE | TRUE | TRUE |
| **1527** | Q9Y619 | Mitochondrial ornithine transporter | MITOCHONDRIA | 34.16 | -9.77 | FALSE | TRUE | FALSE |
| **1528** | O75663 | TIP41-like protein | CYTOSOL | 17.85 | 17.69 | FALSE | TRUE | TRUE |
| **1529** | Q9UL46 | Proteasome activator complex subu | unknown | 23.43 | 25.19 | FALSE | TRUE | TRUE |
| **1530** | P13473 | Lysosome-associated membrane g | unknown | -36.54 | 16.66 | FALSE | TRUE | FALSE |
| **1531** | P17252 | Protein kinase C alpha type | unknown | 12.50 | 16.48 | FALSE | TRUE | FALSE |
| **1532** | Q8TED0 | U3 small nucleolar RNA-associated | NUCLEUS | -2.20 | -38.55 | FALSE | TRUE | FALSE |
| **1533** | Q9H2H8 | Peptidyl-prolyl cis-trans isomerase- | unknown | 20.63 | 4.50 | FALSE | TRUE | FALSE |
| **1534** | P49841 | Glycogen synthase kinase-3 beta | unknown | -9.22 | 8.65 | FALSE | TRUE | TRUE |
| **1535** | Q8NBU5 | ATPase family AAA domain-contair | unknown | 32.84 | -14.49 | FALSE | TRUE | FALSE |
| **1536** | Q8WVV9 | Heterogeneous nuclear ribonucleop | NUCLEUS | -12.41 | -28.91 | FALSE | TRUE | TRUE |
| **1537** | Q9BWF3 | RNA-binding protein 4 | NUCLEUS | -18.65 | -31.99 | FALSE | TRUE | TRUE |
| **1538** | Q8NEJ9 | Neuroguidin | NUCLEUS | -0.14 | -37.80 | FALSE | TRUE | TRUE |
| **1539** | Q5SWX8 | Protein odr-4 homolog | unknown | 2.25 | 34.12 | FALSE | TRUE | FALSE |
| **1540** | Q14331 | Protein FRG1 | NUCLEUS | -7.06 | -20.15 | FALSE | TRUE | TRUE |
| **1541** | Q9BUP3 | Oxidoreductase HTATIP2 | ER | 0.15 | 32.78 | FALSE | TRUE | TRUE |
| **1542** | P36507 | Dual specificity mitogen-activated p | unknown | 14.71 | 9.06 | FALSE | TRUE | FALSE |
| **1543** | O00170 | AH receptor-interacting protein | unknown | 16.49 | 10.02 | FALSE | TRUE | TRUE |
| **1544** | Q9BTE3 | Mini-chromosome maintenance co | unknown | 18.64 | 7.89 | FALSE | TRUE | TRUE |
| **1545** | O60306 | RNA helicase aquarius | NUCLEUS | -3.63 | -39.06 | FALSE | TRUE | TRUE |
| **1546** | Q16513 | Serine/threonine-protein kinase N2 | unknown | -8.60 | 10.94 | TRUE | TRUE | FALSE |
| **1547** | Q08379 | Golgin subfamily A member 2 | NUCLEUS | -8.65 | -18.21 | TRUE | TRUE | TRUE |
| **1548** | Q8WYA6 | Beta-catenin-like protein 1 | unknown | 13.21 | -0.95 | FALSE | TRUE | TRUE |
| **1549** | Q9Y6A4 | Cilia- and flagella-associated protei | NUCLEUS | -3.26 | -15.74 | FALSE | TRUE | TRUE |
| **1550** | Q9BZF1 | Oxysterol-binding protein-related p | ER | -1.91 | 35.51 | FALSE | TRUE | FALSE |
| **1551** | O15533 | Tapasin | unknown | -10.07 | 27.92 | FALSE | TRUE | FALSE |
| **1552** | O75691 | Small subunit processome compon | NUCLEUS | -0.70 | -41.24 | FALSE | TRUE | TRUE |
| **1553** | P29353 | SHC-transforming protein 1 | unknown | 19.14 | -3.46 | FALSE | TRUE | FALSE |
| **1554** | P34896 | Serine hydroxymethyltransferase, c | unknown | 12.48 | 2.82 | FALSE | TRUE | TRUE |

| | | | | | | | | |
|---|---|---|---|---|---|---|---|---|
| **1555** | O00217 | NADH dehydrogenase [ubiquinone] | MITOCHONDRIA | 30.75 | -11.10 | FALSE | TRUE | TRUE |
| **1556** | P17813 | Endoglin | PM | -25.25 | 3.03 | FALSE | TRUE | TRUE |
| **1557** | Q9H9Q2 | COP9 signalosome complex subunit | unknown | 14.12 | 2.94 | FALSE | FALSE | TRUE |
| **1558** | Q96C86 | m7GpppX diphosphatase | unknown | 26.74 | 23.47 | FALSE | TRUE | TRUE |
| **1559** | Q658Y4 | Protein FAM91A1 | unknown | -14.18 | 7.96 | FALSE | TRUE | FALSE |
| **1560** | P78330 | Phosphoserine phosphatase | unknown | 19.34 | 21.78 | FALSE | TRUE | FALSE |
| **1561** | O95989 | Diphosphoinositol polyphosphate p | unknown | 26.03 | 23.38 | FALSE | TRUE | FALSE |
| **1562** | O43913 | Origin recognition complex subunit | NUCLEUS-CHROMATIN | -19.05 | -15.67 | FALSE | TRUE | FALSE |
| **1563** | Q9NRK6 | ATP-binding cassette sub-family B r | unknown | 35.14 | -0.74 | FALSE | TRUE | TRUE |
| **1564** | O60287 | Nucleolar pre-ribosomal-associated | NUCLEUS | -3.57 | -37.12 | FALSE | TRUE | FALSE |
| **1565** | O95861 | 3'(2'),5'-bisphosphate nucleotidase | unknown | 18.45 | 15.25 | FALSE | TRUE | TRUE |
| **1566** | P06703 | Protein S100-A6 | unknown | 19.75 | 23.06 | TRUE | FALSE | TRUE |
| **1567** | Q8WU90 | Zinc finger CCCH domain-containing | unknown | 4.00 | -17.71 | FALSE | TRUE | TRUE |
| **1568** | Q96B26 | Exosome complex component RRP4 | NUCLEUS | -10.73 | -26.21 | FALSE | TRUE | TRUE |
| **1569** | Q9Y3Y2 | Chromatin target of PRMT1 protein | NUCLEUS | -0.53 | -41.82 | FALSE | TRUE | TRUE |
| **1570** | Q3MHD2 | Protein LSM12 homolog | unknown | 2.12 | -16.97 | FALSE | TRUE | FALSE |
| **1571** | Q8NCA5 | Protein FAM98A | unknown | 6.22 | -25.21 | FALSE | TRUE | FALSE |
| **1572** | Q96N67 | Dedicator of cytokinesis protein 7 | unknown | 2.23 | -11.31 | FALSE | TRUE | TRUE |
| **1573** | Q9BV20 | Methylthioribose-1-phosphate ison | unknown | 17.86 | 8.83 | FALSE | TRUE | FALSE |
| **1574** | Q15631 | Translin | unknown | 19.00 | 10.40 | FALSE | TRUE | FALSE |
| **1575** | Q12874 | Splicing factor 3A subunit 3 | NUCLEUS | 4.87 | -34.92 | FALSE | TRUE | FALSE |
| **1576** | Q9BPX5 | Actin-related protein 2/3 complex s | unknown | 1.31 | -14.28 | FALSE | TRUE | FALSE |
| **1577** | O43837 | Isocitrate dehydrogenase [NAD] su | MITOCHONDRIA | 40.45 | -5.26 | FALSE | TRUE | TRUE |
| **1578** | Q6P996 | Pyridoxal-dependent decarboxylase | unknown | 20.06 | 15.81 | TRUE | TRUE | TRUE |
| **1579** | Q9H3N1 | Thioredoxin-related transmembran | ER | 0.96 | 33.08 | TRUE | TRUE | TRUE |
| **1580** | P82673 | 28S ribosomal protein S35, mitocho | MITOCHONDRIA | 43.11 | -6.96 | FALSE | TRUE | TRUE |
| **1581** | Q15005 | Signal peptidase complex subunit 2 | ER | 4.31 | 33.16 | FALSE | TRUE | FALSE |
| **1582** | O96005 | Cleft lip and palate transmembrane | ER | -6.02 | 32.43 | TRUE | TRUE | TRUE |
| **1583** | P21283 | V-type proton ATPase subunit C 1 | unknown | -12.78 | 3.51 | FALSE | TRUE | TRUE |
| **1584** | P56182 | Ribosomal RNA processing protein 1 | NUCLEUS | -0.30 | -37.91 | FALSE | TRUE | TRUE |
| **1585** | Q9UPU5 | Ubiquitin carboxyl-terminal hydrola | unknown | 5.31 | 3.32 | FALSE | TRUE | TRUE |
| **1586** | P14927 | Cytochrome b-c1 complex subunit 7 | MITOCHONDRIA | 32.03 | -13.43 | FALSE | TRUE | TRUE |
| **1587** | Q9NRW7 | Vacuolar protein sorting-associated | unknown | -35.01 | 15.20 | FALSE | TRUE | TRUE |
| **1588** | P07741 | Adenine phosphoribosyltransferase | CYTOSOL | 25.61 | 24.58 | FALSE | TRUE | FALSE |
| **1589** | Q8TC07 | TBC1 domain family member 15 | unknown | 12.95 | 8.80 | FALSE | TRUE | TRUE |
| **1590** | P21953 | 2-oxoisovalerate dehydrogenase su | MITOCHONDRIA | 36.71 | -8.81 | FALSE | TRUE | FALSE |
| **1591** | Q9BXW7 | Haloacid dehalogenase-like hydrola | MITOCHONDRIA | 37.54 | -8.77 | FALSE | TRUE | TRUE |
| **1592** | P82933 | 28S ribosomal protein S9, mitochon | MITOCHONDRIA | 43.07 | -6.38 | FALSE | TRUE | TRUE |

| | | | | | | | | |
|---|---|---|---|---|---|---|---|---|
| **1593** | Q96GC5 | 39S ribosomal protein L48, mitocho | unknown | 42.11 | -3.13 | FALSE | TRUE | FALSE |
| **1594** | Q15836 | Vesicle-associated membrane prot | unknown | -31.89 | 0.15 | TRUE | TRUE | TRUE |
| **1595** | Q9UPP1 | Histone lysine demethylase PHF8 | NUCLEUS-CHROMATIN | -17.31 | -16.33 | FALSE | TRUE | TRUE |
| **1596** | P82930 | 28S ribosomal protein S34, mitocho | MITOCHONDRIA | 39.74 | -7.73 | FALSE | TRUE | TRUE |
| **1597** | P12270 | Nucleoprotein TPR | NUCLEUS | -18.15 | -27.77 | TRUE | TRUE | TRUE |
| **1598** | Q9Y676 | 28S ribosomal protein S18b, mitoch | unknown | 37.61 | -9.67 | FALSE | TRUE | TRUE |
| **1599** | Q9H7N4 | Splicing factor, arginine/serine-rich | NUCLEUS | 1.95 | -33.21 | TRUE | TRUE | TRUE |
| **1600** | O75489 | NADH dehydrogenase [ubiquinone] | MITOCHONDRIA | 31.32 | -12.87 | FALSE | TRUE | FALSE |
| **1601** | Q9NQ88 | Fructose-2,6-bisphosphatase TIGA | unknown | 20.64 | 21.68 | FALSE | TRUE | FALSE |
| **1602** | P52594 | Arf-GAP domain and FG repeat-con | unknown | -0.11 | -14.09 | TRUE | TRUE | TRUE |
| **1603** | Q9NQP4 | Prefoldin subunit 4 | CYTOSOL | 21.63 | 23.54 | FALSE | TRUE | TRUE |
| **1604** | Q05655 | Protein kinase C delta type | unknown | -14.66 | 14.25 | FALSE | TRUE | TRUE |
| **1605** | Q9NU22 | Midasin | NUCLEUS | -4.33 | -31.43 | FALSE | TRUE | TRUE |
| **1606** | Q9H089 | Large subunit GTPase 1 homolog | ER | -2.39 | 35.91 | FALSE | TRUE | FALSE |
| **1607** | Q9BRS2 | Serine/threonine-protein kinase RI | unknown | 12.25 | -9.55 | FALSE | TRUE | TRUE |
| **1608** | Q5GLZ8 | Probable E3 ubiquitin-protein ligase | unknown | 12.82 | 10.88 | FALSE | TRUE | FALSE |
| **1609** | Q9H9A6 | Leucine-rich repeat-containing prot | unknown | 8.43 | -0.41 | FALSE | TRUE | FALSE |
| **1610** | O75475 | PC4 and SFRS1-interacting protein | NUCLEUS | -25.98 | -23.59 | FALSE | TRUE | TRUE |
| **1611** | P49916 | DNA ligase 3 | unknown | -23.42 | -22.45 | FALSE | TRUE | TRUE |
| **1612** | Q15022 | Polycomb protein SUZ12 | unknown | -22.32 | -18.39 | FALSE | TRUE | TRUE |
| **1613** | Q01968 | Inositol polyphosphate 5-phosphat | unknown | -9.25 | 6.00 | FALSE | TRUE | TRUE |
| **1614** | P36405 | ADP-ribosylation factor-like protein | unknown | 17.59 | 18.66 | FALSE | TRUE | FALSE |
| **1615** | Q13405 | 39S ribosomal protein L49, mitocho | MITOCHONDRIA | 40.71 | -11.80 | FALSE | TRUE | TRUE |
| **1616** | Q9NPF4 | Probable tRNA N6-adenosine threo | unknown | 20.78 | 13.06 | FALSE | TRUE | TRUE |
| **1617** | Q9UNZ5 | Leydig cell tumor 10 kDa protein ho | NUCLEUS | -3.18 | -22.47 | FALSE | TRUE | TRUE |
| **1618** | P57772 | Selenocysteine-specific elongation | unknown | -0.55 | -13.58 | FALSE | TRUE | TRUE |
| **1619** | Q9UJ83 | 2-hydroxyacyl-CoA lyase 1 | PEROXISOME | 52.51 | -3.48 | FALSE | TRUE | TRUE |
| **1620** | P33897 | ATP-binding cassette sub-family D | PEROXISOME | 52.88 | -3.56 | FALSE | TRUE | TRUE |
| **1621** | Q6ZNB6 | NF-X1-type zinc finger protein NFXL | unknown | 3.31 | 36.94 | FALSE | TRUE | TRUE |
| **1622** | Q6FI81 | Anamorsin | CYTOSOL | 22.16 | 23.37 | TRUE | TRUE | TRUE |
| **1623** | Q9HCD5 | Nuclear receptor coactivator 5 | NUCLEUS | 1.60 | -35.27 | FALSE | TRUE | TRUE |
| **1624** | Q9Y2Q9 | 28S ribosomal protein S28, mitocho | MITOCHONDRIA | 39.98 | -11.12 | TRUE | TRUE | FALSE |
| **1625** | Q96P70 | Importin-9 | unknown | 19.02 | 15.10 | FALSE | TRUE | FALSE |
| **1626** | Q8IUF8 | Ribosomal oxygenase 2 | unknown | -15.81 | -17.42 | FALSE | TRUE | FALSE |
| **1627** | Q92747 | Actin-related protein 2/3 complex s | unknown | 2.08 | -14.07 | FALSE | TRUE | FALSE |
| **1628** | P19174 | 1-phosphatidylinositol 4,5-bisphosp | unknown | 12.27 | 5.24 | FALSE | TRUE | FALSE |
| **1629** | O43719 | HIV Tat-specific factor 1 | NUCLEUS | 10.74 | -12.23 | TRUE | TRUE | TRUE |
| **1630** | O94992 | Protein HEXIM1 | unknown | 11.82 | -11.38 | FALSE | TRUE | FALSE |

| | | | | | | | | |
|---|---|---|---|---|---|---|---|---|
| **1631** | Q04323 | UBX domain-containing protein 1 | unknown | 18.85 | 20.55 | FALSE | TRUE | TRUE |
| **1632** | Q969X5 | Endoplasmic reticulum-Golgi interm | unknown | -6.71 | 32.56 | FALSE | TRUE | TRUE |
| **1633** | Q96A33 | Coiled-coil domain-containing prote | ER | 2.64 | 35.98 | TRUE | TRUE | TRUE |
| **1634** | Q8WVY7 | Ubiquitin-like domain-containing CT | unknown | 17.37 | 6.35 | FALSE | TRUE | FALSE |
| **1635** | Q9Y446 | Plakophilin-3 | unknown | -33.39 | -1.63 | TRUE | TRUE | TRUE |
| **1636** | Q69YN2 | CWF19-like protein 1 | NUCLEUS | -4.06 | -16.52 | FALSE | TRUE | TRUE |
| **1637** | P08651 | Nuclear factor 1 C-type | NUCLEUS | -22.76 | -23.57 | FALSE | TRUE | TRUE |
| **1638** | P51452 | Dual specificity protein phosphatas | CYTOSOL | 20.95 | 20.90 | FALSE | TRUE | FALSE |
| **1639** | Q96CW5 | Gamma-tubulin complex componer | unknown | 6.34 | -24.83 | FALSE | TRUE | TRUE |
| **1640** | P40937 | Replication factor C subunit 5 | unknown | -15.57 | -16.70 | FALSE | TRUE | FALSE |
| **1641** | O95394 | Phosphoacetylglucosamine mutase | CYTOSOL | 21.78 | 21.88 | FALSE | TRUE | TRUE |
| **1642** | O60341 | Lysine-specific histone demethylas | NUCLEUS | -23.93 | -24.99 | FALSE | TRUE | TRUE |
| **1643** | Q6NZI2 | Caveolae-associated protein 1 | unknown | -0.84 | 1.48 | FALSE | TRUE | TRUE |
| **1644** | Q14126 | Desmoglein-2 | PM | -29.26 | 2.40 | TRUE | TRUE | TRUE |
| **1645** | Q9H2J4 | Phosducin-like protein 3 | CYTOSOL | 11.42 | 18.73 | TRUE | TRUE | TRUE |
| **1646** | Q92614 | Unconventional myosin-XVIIIa | unknown | -1.44 | 3.95 | TRUE | TRUE | TRUE |
| **1647** | Q9UHG3 | Prenylcysteine oxidase 1 | ER | -5.93 | 31.39 | FALSE | TRUE | TRUE |
| **1648** | Q96T76 | MMS19 nucleotide excision repair p | unknown | 10.23 | 3.73 | FALSE | TRUE | FALSE |
| **1649** | Q9Y6E2 | Basic leucine zipper and W2 domair | NUCLEUS | -2.88 | -14.04 | FALSE | TRUE | TRUE |
| **1650** | O14672 | Disintegrin and metalloproteinase c | unknown | -19.86 | 19.15 | FALSE | TRUE | FALSE |
| **1651** | Q9Y697 | Cysteine desulfurase, mitochondria | MITOCHONDRIA | 36.38 | -5.99 | FALSE | TRUE | TRUE |
| **1652** | P14384 | Carboxypeptidase M | PM | -25.95 | 15.36 | FALSE | TRUE | TRUE |
| **1653** | Q9Y2K7 | Lysine-specific demethylase 2A | NUCLEUS | -18.86 | -26.26 | TRUE | TRUE | FALSE |
| **1654** | P37198 | Nuclear pore glycoprotein p62 | unknown | -6.79 | -23.07 | FALSE | TRUE | TRUE |
| **1655** | P07108 | Acyl-CoA-binding protein | unknown | 25.42 | 22.60 | FALSE | TRUE | FALSE |
| **1656** | Q8WWM7 | Ataxin-2-like protein | unknown | 6.52 | -20.52 | TRUE | TRUE | TRUE |
| **1657** | Q9NQG5 | Regulation of nuclear pre-mRNA do | NUCLEUS | -14.83 | -29.56 | FALSE | TRUE | FALSE |
| **1658** | Q5K651 | Sterile alpha motif domain-containi | unknown | -5.02 | -10.03 | FALSE | TRUE | TRUE |
| **1659** | Q96GM5 | SWI/SNF-related matrix-associated | unknown | -24.46 | -22.19 | FALSE | TRUE | TRUE |
| **1660** | O75955 | Flotillin-1 | unknown | -32.41 | 4.70 | TRUE | TRUE | TRUE |
| **1661** | Q92878 | DNA repair protein RAD50 | unknown | -16.56 | -17.01 | FALSE | TRUE | TRUE |
| **1662** | Q9H074 | Polyadenylate-binding protein-inter | unknown | 19.72 | 11.48 | FALSE | TRUE | TRUE |
| **1663** | P28331 | NADH-ubiquinone oxidoreductase 7 | MITOCHONDRIA | 31.09 | -14.57 | FALSE | TRUE | TRUE |
| **1664** | Q9BVJ6 | U3 small nucleolar RNA-associated | NUCLEUS | -0.71 | -41.11 | TRUE | TRUE | TRUE |
| **1665** | O95299 | NADH dehydrogenase [ubiquinone] | MITOCHONDRIA | 30.95 | -13.51 | FALSE | TRUE | TRUE |
| **1666** | P60510 | Serine/threonine-protein phosphata | unknown | 24.91 | 18.82 | FALSE | TRUE | FALSE |
| **1667** | Q6P2E9 | Enhancer of mRNA-decapping prote | unknown | 2.74 | -24.22 | TRUE | TRUE | TRUE |
| **1668** | Q9BV38 | WD repeat-containing protein 18 | NUCLEUS | 0.67 | -34.53 | FALSE | TRUE | TRUE |

| | | | | | | | | |
|---|---|---|---|---|---|---|---|---|
| **1669** | Q96BP3 | Peptidylprolyl isomerase domain ar | NUCLEUS | -1.22 | -26.83 | FALSE | TRUE | FALSE |
| **1670** | Q9BQ69 | O-acetyl-ADP-ribose deacetylase M | MITOCHONDRIA | 31.23 | -11.19 | FALSE | TRUE | FALSE |
| **1671** | Q9NY33 | Dipeptidyl peptidase 3 | CYTOSOL | 21.48 | 23.12 | TRUE | TRUE | TRUE |
| **1672** | Q13895 | Bystin | NUCLEUS | -2.69 | -38.72 | FALSE | TRUE | TRUE |
| **1673** | Q9BTC0 | Death-inducer obliterator 1 | NUCLEUS | -13.85 | -22.64 | TRUE | TRUE | TRUE |
| **1674** | Q92890 | Ubiquitin recognition factor in ER-as | NUCLEUS | -0.97 | -5.38 | FALSE | TRUE | FALSE |
| **1675** | Q8N122 | Regulatory-associated protein of m | unknown | -15.21 | 4.51 | FALSE | TRUE | TRUE |
| **1676** | Q12765 | Secernin-1 | CYTOSOL | 22.18 | 21.21 | FALSE | TRUE | TRUE |
| **1677** | P18887 | DNA repair protein XRCC1 | NUCLEUS | -22.45 | -21.55 | FALSE | TRUE | TRUE |
| **1678** | P42695 | Condensin-2 complex subunit D3 | NUCLEUS-CHROMATIN | -9.53 | -21.36 | FALSE | TRUE | FALSE |
| **1679** | P04179 | Superoxide dismutase [Mn], mitoch | MITOCHONDRIA | 38.13 | -11.34 | FALSE | TRUE | TRUE |
| **1680** | Q6P1L8 | 39S ribosomal protein L14, mitocho | MITOCHONDRIA | 40.39 | -0.75 | FALSE | TRUE | FALSE |
| **1681** | O43681 | ATPase ASNA1 | unknown | 13.99 | 10.14 | FALSE | TRUE | TRUE |
| **1682** | Q9BU61 | NADH dehydrogenase [ubiquinone] | MITOCHONDRIA | 36.21 | -8.84 | FALSE | TRUE | TRUE |
| **1683** | O00469 | Procollagen-lysine,2-oxoglutarate 5 | unknown | -0.69 | 39.20 | FALSE | TRUE | TRUE |
| **1684** | Q13112 | Chromatin assembly factor 1 subun | NUCLEUS | -21.77 | -25.06 | FALSE | TRUE | TRUE |
| **1685** | O43148 | mRNA cap guanine-N7 methyltrans | unknown | 23.88 | 12.86 | FALSE | TRUE | TRUE |
| **1686** | Q86XI2 | Condensin-2 complex subunit G2 | unknown | -5.05 | -13.33 | FALSE | TRUE | TRUE |
| **1687** | Q14807 | Kinesin-like protein KIF22 | NUCLEUS-CHROMATIN | -19.66 | -14.66 | FALSE | TRUE | TRUE |
| **1688** | Q9ULF5 | Zinc transporter ZIP10 | PM | -27.35 | 11.58 | TRUE | TRUE | TRUE |
| **1689** | Q9H0C8 | Integrin-linked kinase-associated se | NUCLEUS | 0.77 | -24.77 | TRUE | TRUE | FALSE |
| **1690** | Q6NUK1 | Calcium-binding mitochondrial carri | MITOCHONDRIA | 43.81 | -10.15 | FALSE | TRUE | TRUE |
| **1691** | O43237 | Cytoplasmic dynein 1 light intermed | unknown | -11.22 | -7.33 | TRUE | TRUE | TRUE |
| **1692** | Q16706 | Alpha-mannosidase 2 | GOLGI | -18.97 | 32.52 | FALSE | TRUE | TRUE |
| **1693** | Q9Y4E8 | Ubiquitin carboxyl-terminal hydrola | unknown | 17.66 | 14.40 | FALSE | TRUE | TRUE |
| **1694** | Q8N0X7 | Spartin | unknown | -3.35 | -2.15 | TRUE | TRUE | TRUE |
| **1695** | P55196 | Afadin | unknown | -28.14 | -0.23 | TRUE | TRUE | TRUE |
| **1696** | Q01970 | 1-phosphatidylinositol 4,5-bisphosp | unknown | -11.53 | 12.68 | TRUE | TRUE | TRUE |
| **1697** | A6NHR9 | Structural maintenance of chromos | NUCLEUS-CHROMATIN | -21.01 | -12.23 | FALSE | TRUE | TRUE |
| **1698** | Q8IZ83 | Aldehyde dehydrogenase family 16 | unknown | 13.79 | -11.02 | FALSE | TRUE | TRUE |
| **1699** | Q9H3K2 | Growth hormone-inducible transme | MITOCHONDRIA | 31.55 | -15.16 | FALSE | TRUE | TRUE |
| **1700** | Q8N1G4 | Leucine-rich repeat-containing prote | unknown | 13.60 | -10.51 | FALSE | TRUE | TRUE |
| **1701** | Q2M389 | WASH complex subunit 4 | unknown | -14.48 | 9.02 | FALSE | TRUE | TRUE |
| **1702** | Q8IXI1 | Mitochondrial Rho GTPase 2 | unknown | 31.66 | -3.37 | FALSE | TRUE | FALSE |
| **1703** | Q9ULK4 | Mediator of RNA polymerase II trar | NUCLEUS | -1.48 | -30.45 | FALSE | TRUE | FALSE |
| **1704** | O14578 | Citron Rho-interacting kinase | unknown | 2.30 | -11.35 | FALSE | TRUE | TRUE |
| **1705** | Q9Y263 | Phospholipase A-2-activating prote | unknown | 18.85 | 15.46 | TRUE | TRUE | TRUE |
| **1706** | P29218 | Inositol monophosphatase 1 | CYTOSOL | 17.82 | 9.06 | FALSE | TRUE | TRUE |

| | | | | | | | | |
|---|---|---|---|---|---|---|---|---|
| **1707** | O15294 | UDP-N-acetylglucosamine--peptide | NUCLEUS | -12.72 | -28.41 | FALSE | TRUE | TRUE |
| **1708** | O94925 | Glutaminase kidney isoform, mitocl | MITOCHONDRIA | 34.88 | -6.86 | FALSE | TRUE | TRUE |
| **1709** | Q16204 | Coiled-coil domain-containing prote | unknown | 13.62 | -1.98 | FALSE | TRUE | TRUE |
| **1710** | Q9H444 | Charged multivesicular body protei | unknown | -17.46 | 3.85 | TRUE | TRUE | TRUE |
| **1711** | Q99961 | Endophilin-A2 | unknown | 15.26 | 11.02 | TRUE | TRUE | TRUE |
| **1712** | P40938 | Replication factor C subunit 3 | unknown | -15.58 | -16.94 | FALSE | TRUE | TRUE |
| **1713** | Q5QJE6 | Deoxynucleotidyltransferase termi | NUCLEUS | -6.14 | -39.13 | TRUE | TRUE | TRUE |
| **1714** | Q9NXF1 | Testis-expressed protein 10 | NUCLEUS | 1.12 | -34.78 | FALSE | TRUE | FALSE |
| **1715** | P49711 | Transcriptional repressor CTCF | NUCLEUS | -25.63 | -24.34 | TRUE | TRUE | TRUE |
| **1716** | Q8ND56 | Protein LSM14 homolog A | NUCLEUS | -2.06 | -14.76 | FALSE | TRUE | TRUE |
| **1717** | Q7L8L6 | FAST kinase domain-containing pro | MITOCHONDRIA | 38.46 | -9.64 | FALSE | TRUE | TRUE |
| **1718** | Q9GZS3 | WD repeat-containing protein 61 | unknown | 4.99 | -18.79 | FALSE | TRUE | TRUE |
| **1719** | Q9NQT5 | Exosome complex component RRP4 | NUCLEUS | -10.88 | -25.96 | FALSE | TRUE | FALSE |
| **1720** | Q9H5Q4 | Dimethyladenosine transferase 2, r | MITOCHONDRIA | 34.89 | -12.06 | FALSE | TRUE | FALSE |
| **1721** | O60832 | H/ACA ribonucleoprotein complex s | NUCLEUS | -4.14 | -39.00 | TRUE | TRUE | TRUE |
| **1722** | Q9NTI5 | Sister chromatid cohesion protein P | NUCLEUS-CHROMATIN | -18.27 | -16.06 | FALSE | TRUE | TRUE |
| **1723** | Q9UNS2 | COP9 signalosome complex subunit | unknown | 11.06 | 19.65 | FALSE | TRUE | TRUE |
| **1724** | Q9BZK7 | F-box-like/WD repeat-containing pr | NUCLEUS | -13.71 | -29.75 | FALSE | TRUE | TRUE |
| **1725** | Q9UIA9 | Exportin-7 | CYTOSOL | 20.20 | 16.95 | FALSE | TRUE | TRUE |
| **1726** | Q9NYL9 | Tropomodulin-3 | unknown | 5.37 | 7.66 | TRUE | TRUE | FALSE |
| **1727** | Q8IZH2 | 5'-3' exoribonuclease 1 | unknown | 1.33 | -14.36 | TRUE | TRUE | TRUE |
| **1728** | P61956 | Small ubiquitin-related modifier 2 | unknown | -17.49 | -31.36 | TRUE | FALSE | TRUE |
| **1729** | Q9Y3E0 | Vesicle transport protein GOT1B | unknown | -8.53 | 32.78 | FALSE | TRUE | FALSE |
| **1730** | P36915 | Guanine nucleotide-binding protein | unknown | 18.89 | 10.56 | FALSE | TRUE | TRUE |
| **1731** | Q92692 | Nectin-2 | PM | -26.30 | 14.27 | TRUE | FALSE | TRUE |
| **1732** | Q92692 | Nectin-2 | PM | -24.71 | 4.58 | TRUE | FALSE | TRUE |
| **1733** | Q8WUA4 | General transcription factor 3C poly | NUCLEUS | -23.60 | -23.11 | FALSE | TRUE | FALSE |
| **1734** | Q86UK7 | E3 ubiquitin-protein ligase ZNF598 | unknown | 7.52 | -20.58 | TRUE | TRUE | TRUE |
| **1735** | Q9Y232 | Chromodomain Y-like protein | NUCLEUS-CHROMATIN | -20.25 | -14.65 | TRUE | TRUE | TRUE |
| **1736** | P33527 | Multidrug resistance-associated pro | PM | -21.63 | 11.46 | TRUE | TRUE | TRUE |
| **1737** | Q9H9Y2 | Ribosome production factor 1 | NUCLEUS | -1.02 | -37.66 | FALSE | TRUE | FALSE |
| **1738** | Q12849 | G-rich sequence factor 1 | MITOCHONDRIA | 38.00 | -8.34 | FALSE | TRUE | TRUE |
| **1739** | Q92665 | 28S ribosomal protein S31, mitocho | MITOCHONDRIA | 40.33 | -6.81 | FALSE | TRUE | TRUE |
| **1740** | O00193 | Small acidic protein | unknown | 27.37 | 23.29 | TRUE | TRUE | TRUE |
| **1741** | Q15654 | Thyroid receptor-interacting protei | unknown | -4.74 | -7.09 | FALSE | TRUE | TRUE |
| **1742** | Q9C0J8 | pre-mRNA 3' end processing protei | NUCLEUS | -9.81 | -36.08 | TRUE | TRUE | TRUE |
| **1743** | P35249 | Replication factor C subunit 4 | unknown | -15.80 | -17.05 | TRUE | TRUE | FALSE |
| **1744** | O00233 | 26S proteasome non-ATPase regula | unknown | 14.30 | 15.42 | FALSE | TRUE | TRUE |

| | | | | | | | | |
|---|---|---|---|---|---|---|---|---|
| **1745** | Q9NNW5 | WD repeat-containing protein 6 | NUCLEUS | 9.37 | -4.17 | FALSE | TRUE | FALSE |
| **1746** | P36897 | TGF-beta receptor type-1 | unknown | -15.65 | 15.90 | FALSE | TRUE | FALSE |
| **1747** | Q8N0U8 | Vitamin K epoxide reductase compl | unknown | -0.32 | 32.22 | FALSE | TRUE | FALSE |
| **1748** | Q96GG9 | DCN1-like protein 1 | unknown | 9.95 | 0.08 | FALSE | TRUE | TRUE |
| **1749** | Q6ZRV2 | Protein FAM83H | unknown | -4.25 | 14.36 | FALSE | TRUE | TRUE |
| **1750** | Q14008 | Cytoskeleton-associated protein 5 | unknown | -12.74 | -8.35 | FALSE | TRUE | TRUE |
| **1751** | Q15058 | Kinesin-like protein KIF14 | unknown | -7.16 | 0.11 | FALSE | TRUE | TRUE |
| **1752** | O95251 | Histone acetyltransferase KAT7 | unknown | -17.30 | -17.88 | FALSE | TRUE | TRUE |
| **1753** | Q8N6R0 | Methyltransferase-like protein 13 | unknown | -4.27 | -10.03 | FALSE | TRUE | TRUE |
| **1754** | O15212 | Prefoldin subunit 6 | unknown | 19.94 | 9.27 | FALSE | TRUE | TRUE |
| **1755** | P54920 | Alpha-soluble NSF attachment prot | unknown | -35.37 | 17.02 | FALSE | TRUE | FALSE |
| **1756** | P32004 | Neural cell adhesion molecule L1 | PM | -26.67 | 12.45 | FALSE | TRUE | TRUE |
| **1757** | P21399 | Cytoplasmic aconitate hydratase | CYTOSOL | 20.49 | 22.45 | FALSE | TRUE | FALSE |
| **1758** | Q9BZH6 | WD repeat-containing protein 11 | unknown | -14.32 | 8.02 | FALSE | TRUE | FALSE |
| **1759** | P10301 | Ras-related protein R-Ras | PM | -26.00 | 2.56 | FALSE | TRUE | TRUE |
| **1760** | Q6PKG0 | La-related protein 1 | unknown | 7.08 | -24.70 | TRUE | TRUE | TRUE |
| **1761** | Q8TBX8 | Phosphatidylinositol 5-phosphate 4 | unknown | -2.04 | 6.91 | FALSE | TRUE | TRUE |
| **1762** | Q8NFH3 | Nucleoporin Nup43 | NUCLEUS | -19.03 | -29.81 | FALSE | TRUE | FALSE |
| **1763** | O76071 | Probable cytosolic iron-sulfur protei | unknown | 16.24 | 12.73 | FALSE | TRUE | TRUE |
| **1764** | Q8WVC6 | Dephospho-CoA kinase domain-cor | ER | 2.01 | 33.58 | FALSE | TRUE | FALSE |
| **1765** | Q9ULW0 | Targeting protein for Xklp2 | NUCLEUS-CHROMATIN | -21.54 | -12.98 | TRUE | TRUE | TRUE |
| **1766** | Q6PL18 | ATPase family AAA domain-contair | unknown | -21.01 | -12.19 | FALSE | TRUE | TRUE |
| **1767** | Q9BYT8 | Neurolysin, mitochondrial | unknown | 21.95 | -3.81 | FALSE | TRUE | FALSE |
| **1768** | Q6PI48 | Aspartate--tRNA ligase, mitochondr | MITOCHONDRIA | 35.61 | -14.36 | FALSE | TRUE | FALSE |
| **1769** | P08648 | Integrin alpha-5 | PM | -30.87 | 13.33 | FALSE | TRUE | TRUE |
| **1770** | Q96HE7 | ERO1-like protein alpha | unknown | -0.74 | 40.04 | FALSE | TRUE | TRUE |
| **1771** | Q9P2I0 | Cleavage and polyadenylation spec | NUCLEUS | -10.06 | -36.06 | FALSE | TRUE | FALSE |
| **1772** | Q99959 | Plakophilin-2 | unknown | -23.11 | 3.95 | TRUE | TRUE | TRUE |
| **1773** | Q2NKX8 | DNA excision repair protein ERCC-6- | NUCLEUS | 2.15 | -3.06 | TRUE | TRUE | TRUE |
| **1774** | Q96RL7 | Vacuolar protein sorting-associated | unknown | -3.68 | 1.65 | FALSE | TRUE | FALSE |
| **1775** | Q96TA2 | ATP-dependent zinc metalloprotea: | MITOCHONDRIA | 34.38 | -12.14 | FALSE | TRUE | FALSE |
| **1776** | P60983 | Glia maturation factor beta | unknown | 10.13 | 21.50 | FALSE | TRUE | FALSE |
| **1777** | Q13526 | Peptidyl-prolyl cis-trans isomerase I | unknown | 10.30 | -0.96 | FALSE | TRUE | TRUE |
| **1778** | Q9Y6Y8 | SEC23-interacting protein | unknown | 3.46 | 11.38 | FALSE | TRUE | TRUE |
| **1779** | Q8WWY3 | U4/U6 small nuclear ribonucleoprot | NUCLEUS | 3.70 | -35.58 | FALSE | TRUE | TRUE |
| **1780** | Q9Y3B2 | Exosome complex component CSL4 | NUCLEUS | -10.70 | -26.18 | FALSE | TRUE | TRUE |
| **1781** | P14174 | Macrophage migration inhibitory fa | unknown | 14.33 | 23.29 | FALSE | FALSE | TRUE |
| **1782** | Q8NEZ5 | F-box only protein 22 | unknown | 25.19 | 21.10 | FALSE | TRUE | TRUE |

| | | | | | | | | |
|---|---|---|---|---|---|---|---|---|
| **1783** | P23458 | Tyrosine-protein kinase JAK1 | PM | -24.56 | 13.37 | FALSE | TRUE | FALSE |
| **1784** | P35250 | Replication factor C subunit 2 | unknown | -15.74 | -17.23 | FALSE | TRUE | TRUE |
| **1785** | Q9BQ70 | Transcription factor 25 | unknown | -7.58 | -0.52 | FALSE | TRUE | TRUE |
| **1786** | Q9UBC2 | Epidermal growth factor receptor su | unknown | 2.37 | -10.37 | TRUE | TRUE | TRUE |
| **1787** | Q8N6T3 | ADP-ribosylation factor GTPase-act | unknown | -3.16 | 6.34 | TRUE | TRUE | TRUE |
| **1788** | Q92997 | Segment polarity protein dishevelle | unknown | 2.52 | 1.70 | TRUE | TRUE | TRUE |
| **1789** | P28838 | Cytosol aminopeptidase | unknown | 20.21 | -3.21 | FALSE | TRUE | TRUE |
| **1790** | Q9NRN7 | L-aminoadipate-semialdehyde deh | unknown | 22.55 | 11.82 | FALSE | TRUE | FALSE |
| **1791** | O43818 | U3 small nucleolar RNA-interacting | NUCLEUS | -3.44 | -40.78 | FALSE | TRUE | TRUE |
| **1792** | P09417 | Dihydropteridine reductase | unknown | 9.05 | 7.48 | FALSE | TRUE | FALSE |
| **1793** | Q03001 | Dystonin | unknown | -22.92 | 8.22 | TRUE | FALSE | TRUE |
| **1794** | Q03001 | Dystonin | unknown | -1.70 | 1.86 | TRUE | FALSE | TRUE |
| **1795** | Q63HN8 | E3 ubiquitin-protein ligase RNF213 | unknown | 6.29 | -24.74 | FALSE | TRUE | TRUE |
| **1796** | Q6P1N9 | Putative deoxyribonuclease TATDN | unknown | 9.75 | 8.91 | FALSE | TRUE | FALSE |
| **1797** | P19404 | NADH dehydrogenase [ubiquinone] | MITOCHONDRIA | 31.02 | -14.95 | FALSE | TRUE | FALSE |
| **1798** | Q03468 | DNA excision repair protein ERCC-6 | NUCLEUS | -12.25 | -18.53 | FALSE | TRUE | TRUE |
| **1799** | P50897 | Palmitoyl-protein thioesterase 1 | unknown | -40.92 | 9.33 | FALSE | TRUE | TRUE |
| **1800** | Q9H0U6 | 39S ribosomal protein L18, mitocho | MITOCHONDRIA | 36.47 | -4.67 | FALSE | TRUE | FALSE |
| **1801** | Q9H4A6 | Golgi phosphoprotein 3 | unknown | -22.09 | 29.43 | FALSE | TRUE | FALSE |
| **1802** | Q9BYD3 | 39S ribosomal protein L4, mitochon | MITOCHONDRIA | 36.86 | -6.20 | FALSE | TRUE | TRUE |
| **1803** | Q96LD4 | E3 ubiquitin-protein ligase TRIM47 | unknown | 2.21 | -1.99 | FALSE | TRUE | TRUE |
| **1804** | Q96FZ2 | Embryonic stem cell-specific 5-hydr | unknown | -2.34 | -1.97 | FALSE | TRUE | TRUE |
| **1805** | P52948 | Nuclear pore complex protein Nup9 | NUCLEUS | -20.70 | -29.05 | TRUE | TRUE | TRUE |
| **1806** | Q9UKF6 | Cleavage and polyadenylation spec | NUCLEUS | -10.58 | -35.85 | FALSE | TRUE | TRUE |
| **1807** | Q8TBC4 | NEDD8-activating enzyme E1 catal | unknown | 26.14 | 23.17 | FALSE | TRUE | FALSE |
| **1808** | P00533 | Epidermal growth factor receptor | PM | -27.77 | 7.51 | TRUE | TRUE | TRUE |
| **1809** | O75380 | NADH dehydrogenase [ubiquinone] | MITOCHONDRIA | 29.10 | -7.88 | FALSE | TRUE | FALSE |
| **1810** | Q96H79 | Zinc finger CCCH-type antiviral prot | unknown | -2.25 | -10.02 | FALSE | TRUE | TRUE |
| **1811** | Q14232 | Translation initiation factor eIF-2B s | unknown | 21.64 | 12.32 | FALSE | TRUE | TRUE |
| **1812** | P61225 | Ras-related protein Rap-2b | unknown | -28.32 | 10.79 | FALSE | TRUE | FALSE |
| **1813** | O75027 | ATP-binding cassette sub-family B r | MITOCHONDRIA | 35.50 | -3.30 | FALSE | TRUE | FALSE |
| **1814** | O95140 | Mitofusin-2 | MITOCHONDRIA | 34.43 | -4.07 | FALSE | TRUE | TRUE |
| **1815** | P49757 | Protein numb homolog | unknown | -9.11 | 8.70 | TRUE | FALSE | FALSE |
| **1816** | O95302 | Peptidyl-prolyl cis-trans isomerase I | ER | 2.69 | 39.60 | FALSE | TRUE | FALSE |
| **1817** | Q96P11 | Probable 28S rRNA (cytosine-C(5))-r | NUCLEUS | 2.58 | -27.38 | FALSE | TRUE | FALSE |
| **1818** | Q96GM8 | Target of EGR1 protein 1 | NUCLEUS | 2.08 | -27.94 | FALSE | TRUE | TRUE |
| **1819** | Q9H7Z7 | Prostaglandin E synthase 2 | MITOCHONDRIA | 38.93 | -12.09 | FALSE | TRUE | FALSE |
| **1820** | O00534 | von Willebrand factor A domain-co | unknown | 4.08 | 1.39 | FALSE | TRUE | TRUE |

| | | | | | | | | | |
|---|---|---|---|---|---|---|---|---|---|
| **1821** | Q6P1M0 | Long-chain fatty acid transport prot | ER | -5.97 | 32.55 | FALSE | TRUE | FALSE |
| **1822** | Q13356 | RING-type E3 ubiquitin-protein ligas | NUCLEUS | -1.63 | -26.90 | FALSE | TRUE | TRUE |
| **1823** | Q04726 | Transducin-like enhancer protein 3 | unknown | 8.45 | -13.86 | FALSE | TRUE | TRUE |
| **1824** | Q07864 | DNA polymerase epsilon catalytic s | NUCLEUS | -9.92 | -15.86 | FALSE | TRUE | TRUE |
| **1825** | Q32P41 | tRNA (guanine(37)-N1)-methyltrans | unknown | 20.00 | -2.84 | FALSE | TRUE | FALSE |
| **1826** | Q9H6Z4 | Ran-binding protein 3 | unknown | 20.64 | 15.16 | FALSE | TRUE | TRUE |
| **1827** | Q9BU23 | Lipase maturation factor 2 | ER | 4.26 | 35.78 | FALSE | TRUE | TRUE |
| **1828** | Q96EY7 | Pentatricopeptide repeat domain-c | MITOCHONDRIA | 43.42 | -11.72 | FALSE | TRUE | TRUE |
| **1829** | Q96GD4 | Aurora kinase B | unknown | -17.08 | -13.65 | FALSE | TRUE | FALSE |
| **1830** | Q99442 | Translocation protein SEC62 | ER | 2.35 | 35.34 | FALSE | TRUE | TRUE |
| **1831** | Q9Y2X0 | Mediator of RNA polymerase II trar | NUCLEUS | -8.32 | -21.34 | FALSE | TRUE | FALSE |
| **1832** | P49821 | NADH dehydrogenase [ubiquinone] | MITOCHONDRIA | 30.84 | -14.16 | FALSE | TRUE | FALSE |
| **1833** | Q8NFF5 | FAD synthase | unknown | 21.62 | -3.75 | FALSE | TRUE | FALSE |
| **1834** | Q05397 | Focal adhesion kinase 1 | unknown | 9.28 | -3.15 | FALSE | TRUE | TRUE |
| **1835** | P28482 | Mitogen-activated protein kinase 1 | unknown | 7.54 | -5.44 | FALSE | TRUE | TRUE |
| **1836** | Q96Q11 | CCA tRNA nucleotidyltransferase 1, | unknown | 21.34 | -3.67 | FALSE | TRUE | FALSE |
| **1837** | Q9BZJ0 | Crooked neck-like protein 1 | NUCLEUS | -4.13 | -38.19 | FALSE | TRUE | TRUE |
| **1838** | Q05048 | Cleavage stimulation factor subunit | NUCLEUS | -10.42 | -35.03 | FALSE | TRUE | FALSE |
| **1839** | Q9NV31 | U3 small nucleolar ribonucleoprotei | NUCLEUS | -2.16 | -38.61 | FALSE | TRUE | FALSE |
| **1840** | Q8N8A6 | ATP-dependent RNA helicase DDX5 | NUCLEUS | -1.73 | -34.54 | FALSE | TRUE | TRUE |
| **1841** | Q14738 | Serine/threonine-protein phosphata | unknown | 15.87 | 9.55 | TRUE | TRUE | TRUE |
| **1842** | P52732 | Kinesin-like protein KIF11 | unknown | -10.75 | -7.02 | FALSE | TRUE | TRUE |
| **1843** | P40189 | Interleukin-6 receptor subunit beta | unknown | -33.02 | 12.67 | TRUE | FALSE | FALSE |
| **1844** | Q9H3U1 | Protein unc-45 homolog A | unknown | 6.41 | 4.50 | FALSE | TRUE | FALSE |
| **1845** | Q8IY17 | Neuropathy target esterase | unknown | -7.41 | 37.35 | FALSE | TRUE | TRUE |
| **1846** | Q96RE7 | Nucleus accumbens-associated pro | NUCLEUS | -9.37 | -16.24 | FALSE | TRUE | TRUE |
| **1847** | Q9BT78 | COP9 signalosome complex subunit | unknown | 17.30 | 12.98 | FALSE | TRUE | FALSE |
| **1848** | Q5T9L3 | Protein wntless homolog | unknown | -9.80 | 33.52 | FALSE | TRUE | FALSE |
| **1849** | Q9Y2W2 | WW domain-binding protein 11 | NUCLEUS | 3.58 | -34.52 | TRUE | TRUE | TRUE |
| **1850** | A4D1E9 | GTP-binding protein 10 | MITOCHONDRIA | 42.93 | -12.31 | FALSE | TRUE | TRUE |
| **1851** | O75695 | Protein XRP2 | PM | -21.91 | 9.76 | FALSE | TRUE | FALSE |
| **1852** | Q53EP0 | Fibronectin type III domain-contain | unknown | 5.02 | 37.83 | FALSE | TRUE | TRUE |
| **1853** | Q13617 | Cullin-2 | unknown | 15.72 | 7.35 | FALSE | TRUE | TRUE |
| **1854** | P20645 | Cation-dependent mannose-6-phos | unknown | -34.51 | 19.06 | FALSE | TRUE | TRUE |
| **1855** | Q9NVD7 | Alpha-parvin | unknown | -11.52 | 12.99 | TRUE | TRUE | TRUE |
| **1856** | P82663 | 28S ribosomal protein S25, mitocho | unknown | 44.06 | -6.23 | FALSE | TRUE | FALSE |
| **1857** | Q5H9R7 | Serine/threonine-protein phosphata | unknown | 20.15 | 14.70 | TRUE | TRUE | TRUE |
| **1858** | P04040 | Catalase | PEROXISOME | 52.86 | -3.53 | FALSE | TRUE | TRUE |

| | | | | | | | | |
|---|---|---|---|---|---|---|---|---|
| **1859** | Q9NYH9 | U3 small nucleolar RNA-associated | NUCLEUS | -2.57 | -40.99 | FALSE | TRUE | TRUE |
| **1860** | Q96K37 | Solute carrier family 35 member E1 | unknown | -16.62 | 31.62 | FALSE | TRUE | FALSE |
| **1861** | P41223 | Protein BUD31 homolog | NUCLEUS | -2.09 | -26.81 | FALSE | TRUE | FALSE |
| **1862** | Q9NX47 | E3 ubiquitin-protein ligase MARCH5 | unknown | 34.99 | -2.99 | FALSE | TRUE | FALSE |
| **1863** | Q9UJ68 | Mitochondrial peptide methionine s | unknown | 18.42 | -3.14 | FALSE | TRUE | FALSE |
| **1864** | Q969N2 | GPI transamidase component PIG-T | ER | 1.86 | 33.43 | FALSE | TRUE | TRUE |
| **1865** | Q9H4M9 | EH domain-containing protein 1 | unknown | -18.63 | 10.42 | TRUE | TRUE | TRUE |
| **1866** | A6NDG6 | Glycerol-3-phosphate phosphatase | CYTOSOL | 17.94 | 16.43 | FALSE | TRUE | TRUE |
| **1867** | Q13765 | Nascent polypeptide-associated co | unknown | 11.59 | -7.34 | TRUE | FALSE | TRUE |
| **1868** | O60244 | Mediator of RNA polymerase II tra | NUCLEUS | -1.68 | -30.81 | FALSE | TRUE | TRUE |
| **1869** | P31937 | 3-hydroxyisobutyrate dehydrogena | MITOCHONDRIA | 36.97 | -3.40 | FALSE | TRUE | TRUE |
| **1870** | Q9BTW9 | Tubulin-specific chaperone D | unknown | 19.59 | 14.97 | FALSE | TRUE | TRUE |
| **1871** | Q96CU9 | FAD-dependent oxidoreductase do | MITOCHONDRIA | 34.80 | -14.33 | FALSE | TRUE | FALSE |
| **1872** | Q29RF7 | Sister chromatid cohesion protein P | unknown | -16.11 | -21.53 | TRUE | TRUE | TRUE |
| **1873** | O75832 | 26S proteasome non-ATPase regula | PROTEASOME | 18.11 | 21.40 | FALSE | TRUE | TRUE |
| **1874** | O95391 | Pre-mRNA-splicing factor SLU7 | NUCLEUS | -5.49 | -37.41 | TRUE | TRUE | TRUE |
| **1875** | O96028 | Histone-lysine N-methyltransferase | NUCLEUS-CHROMATIN | -19.50 | -15.76 | FALSE | TRUE | TRUE |
| **1876** | Q96MX6 | WD repeat-containing protein 92 | unknown | 6.12 | -21.59 | FALSE | TRUE | FALSE |
| **1877** | P22307 | Non-specific lipid-transfer protein | PEROXISOME | 52.99 | -3.59 | FALSE | TRUE | TRUE |
| **1878** | Q99700 | Ataxin-2 | unknown | 6.42 | -22.18 | FALSE | TRUE | FALSE |
| **1879** | Q6PK04 | Coiled-coil domain-containing prote | NUCLEUS | 1.49 | -29.41 | FALSE | TRUE | TRUE |
| **1880** | Q8IYU8 | Calcium uptake protein 2, mitochon | unknown | 28.69 | -12.26 | FALSE | TRUE | FALSE |
| **1881** | Q9P0I2 | ER membrane protein complex subu | ER | 4.24 | 36.00 | FALSE | TRUE | FALSE |
| **1882** | Q5VWQ0 | Lysine-specific demethylase 9 | NUCLEUS | 0.05 | -30.06 | FALSE | TRUE | TRUE |
| **1883** | Q9UNF0 | Protein kinase C and casein kinase s | unknown | -17.30 | 11.77 | TRUE | TRUE | FALSE |
| **1884** | O60763 | General vesicular transport factor p | unknown | 19.13 | 15.85 | FALSE | TRUE | TRUE |
| **1885** | O76031 | ATP-dependent Clp protease ATP-b | MITOCHONDRIA | 38.14 | -9.51 | FALSE | TRUE | TRUE |
| **1886** | O00764 | Pyridoxal kinase | unknown | 24.75 | 24.67 | FALSE | TRUE | FALSE |
| **1887** | P36551 | Oxygen-dependent coproporphyrin | MITOCHONDRIA | 40.12 | -9.03 | FALSE | TRUE | FALSE |
| **1888** | Q14CX7 | N-alpha-acetyltransferase 25, NatB | unknown | 5.11 | -19.46 | FALSE | TRUE | FALSE |
| **1889** | O75592 | E3 ubiquitin-protein ligase MYCBP2 | unknown | 4.26 | -15.02 | FALSE | TRUE | TRUE |
| **1890** | Q9NUL3 | Double-stranded RNA-binding prote | unknown | 6.53 | -19.53 | TRUE | TRUE | TRUE |
| **1891** | Q9NUI1 | Peroxisomal 2,4-dienoyl-CoA reduct | PEROXISOME | 52.47 | -3.43 | FALSE | TRUE | FALSE |
| **1892** | Q9NZJ4 | Sacsin | unknown | 7.52 | -20.71 | FALSE | TRUE | FALSE |
| **1893** | P62993 | Growth factor receptor-bound prote | unknown | 8.87 | 13.90 | FALSE | TRUE | TRUE |
| **1894** | Q9HC07 | Transmembrane protein 165 | unknown | -20.95 | 30.91 | FALSE | TRUE | TRUE |
| **1895** | P17706 | Tyrosine-protein phosphatase non- | unknown | -0.46 | 26.70 | FALSE | TRUE | TRUE |
| **1896** | O75818 | Ribonuclease P protein subunit p40 | NUCLEUS | -1.21 | -26.00 | FALSE | TRUE | FALSE |

| | | | | | | | | |
|---|---|---|---|---|---|---|---|---|
| **1897** | O60502 | Protein O-GlcNAcase | unknown | 15.97 | 14.97 | FALSE | TRUE | FALSE |
| **1898** | P61204 | ADP-ribosylation factor 3 | unknown | 18.46 | 11.16 | TRUE | FALSE | TRUE |
| **1899** | Q9NRV9 | Heme-binding protein 1 | unknown | 19.73 | -2.81 | FALSE | TRUE | TRUE |
| **1900** | Q86X10 | Ral GTPase-activating protein subu | unknown | -7.15 | -6.58 | FALSE | TRUE | FALSE |
| **1901** | Q8IX18 | Probable ATP-dependent RNA helic | NUCLEUS | -0.76 | -24.90 | FALSE | TRUE | FALSE |
| **1902** | Q9H9J2 | 39S ribosomal protein L44, mitocho | MITOCHONDRIA | 42.94 | -6.80 | FALSE | TRUE | FALSE |
| **1903** | Q5T8P6 | RNA-binding protein 26 | NUCLEUS | -3.08 | -31.91 | TRUE | TRUE | TRUE |
| **1904** | Q8IX01 | SURP and G-patch domain-containi | NUCLEUS | -8.65 | -33.10 | TRUE | TRUE | TRUE |
| **1905** | Q52LJ0 | Protein FAM98B | NUCLEUS | 4.05 | -26.30 | FALSE | TRUE | TRUE |
| **1906** | Q9BSF4 | Mitochondrial import inner membra | MITOCHONDRIA | 35.70 | -12.10 | FALSE | TRUE | FALSE |
| **1907** | Q16718 | NADH dehydrogenase [ubiquinone] | MITOCHONDRIA | 31.74 | -13.04 | FALSE | TRUE | FALSE |
| **1908** | Q9C0B5 | Palmitoyltransferase ZDHHC5 | unknown | -27.25 | 1.73 | TRUE | FALSE | FALSE |
| **1909** | P12694 | 2-oxoisovalerate dehydrogenase su | MITOCHONDRIA | 34.27 | -7.96 | FALSE | TRUE | FALSE |
| **1910** | Q9Y5Q8 | General transcription factor 3C poly | NUCLEUS | -22.99 | -23.17 | FALSE | TRUE | FALSE |
| **1911** | O14757 | Serine/threonine-protein kinase Ch | unknown | 0.30 | -12.74 | FALSE | TRUE | TRUE |
| **1912** | Q96I99 | Succinate--CoA ligase [GDP-forming | MITOCHONDRIA | 41.35 | -10.50 | FALSE | TRUE | TRUE |
| **1913** | P51398 | 28S ribosomal protein S29, mitocho | MITOCHONDRIA | 40.21 | -15.19 | FALSE | TRUE | TRUE |
| **1914** | P11717 | Cation-independent mannose-6-ph | unknown | -34.57 | 18.78 | TRUE | TRUE | TRUE |
| **1915** | Q96RT1 | Erbin | unknown | -23.90 | 4.91 | TRUE | TRUE | TRUE |
| **1916** | Q9H307 | Pinin | NUCLEUS | -4.94 | -39.86 | TRUE | TRUE | TRUE |
| **1917** | O00401 | Neural Wiskott-Aldrich syndrome p | unknown | 2.01 | -10.29 | TRUE | TRUE | TRUE |
| **1918** | Q96HY7 | Probable 2-oxoglutarate dehydroge | unknown | 35.71 | -1.35 | FALSE | TRUE | TRUE |
| **1919** | O00411 | DNA-directed RNA polymerase, mit | MITOCHONDRIA | 29.46 | -13.58 | FALSE | TRUE | FALSE |
| **1920** | O43688 | Phospholipid phosphatase 2 | unknown | -35.53 | 15.56 | FALSE | TRUE | TRUE |
| **1921** | Q13433 | Zinc transporter ZIP6 | PM | -21.61 | 10.90 | TRUE | TRUE | TRUE |
| **1922** | P16333 | Cytoplasmic protein NCK1 | unknown | 3.88 | 0.88 | TRUE | TRUE | TRUE |
| **1923** | O75530 | Polycomb protein EED | NUCLEUS-CHROMATIN | -16.99 | -16.80 | FALSE | TRUE | TRUE |
| **1924** | O14974 | Protein phosphatase 1 regulatory su | unknown | -2.79 | -0.45 | TRUE | TRUE | TRUE |
| **1925** | Q8WW59 | SPRY domain-containing protein 4 | MITOCHONDRIA | 43.80 | -11.14 | FALSE | TRUE | TRUE |
| **1926** | Q32MZ4 | Leucine-rich repeat flightless-intera | unknown | 15.57 | 5.60 | TRUE | TRUE | TRUE |
| **1927** | P04626 | Receptor tyrosine-protein kinase er | PM | -27.08 | 1.63 | TRUE | FALSE | TRUE |
| **1928** | Q7Z3B4 | Nucleoporin p54 | NUCLEUS | -21.12 | -29.57 | FALSE | TRUE | FALSE |
| **1929** | Q9Y4A5 | Transformation/transcription doma | unknown | -10.40 | -22.12 | FALSE | TRUE | TRUE |
| **1930** | Q86VI3 | Ras GTPase-activating-like protein I | unknown | -8.59 | 6.78 | FALSE | TRUE | FALSE |
| **1931** | P06756 | Integrin alpha-V | PM | -26.58 | 14.13 | FALSE | TRUE | TRUE |
| **1932** | Q9Y6N5 | Sulfide:quinone oxidoreductase, mi | MITOCHONDRIA | 36.39 | -4.25 | FALSE | TRUE | TRUE |
| **1933** | Q9Y6R0 | Numb-like protein | unknown | -9.62 | 2.57 | TRUE | FALSE | TRUE |
| **1934** | P84101 | Small EDRK-rich factor 2 | NUCLEUS | -6.40 | -19.27 | FALSE | TRUE | TRUE |

| | | | | | | | | |
|---|---|---|---|---|---|---|---|---|
| **1935** | P43304 | Glycerol-3-phosphate dehydrogena | MITOCHONDRIA | 43.43 | -11.01 | FALSE | TRUE | TRUE |
| **1936** | P07602 | Prosaposin | unknown | -34.46 | 18.16 | FALSE | TRUE | TRUE |
| **1937** | Q9UDY2 | Tight junction protein ZO-2 | unknown | -7.32 | 7.36 | TRUE | TRUE | TRUE |
| **1938** | Q14674 | Separin | unknown | -4.04 | -1.24 | FALSE | TRUE | TRUE |
| **1939** | Q9P2R3 | Rabankyrin-5 | unknown | 3.80 | 1.74 | FALSE | TRUE | FALSE |
| **1940** | P13674 | Prolyl 4-hydroxylase subunit alpha- | unknown | 4.30 | 39.69 | FALSE | TRUE | TRUE |
| **1941** | O95396 | Adenylyltransferase and sulfurtrans | unknown | 16.16 | 13.62 | FALSE | TRUE | TRUE |
| **1942** | Q9H8V3 | Protein ECT2 | unknown | 4.24 | -18.74 | FALSE | TRUE | FALSE |
| **1943** | Q7Z7H8 | 39S ribosomal protein L10, mitocho | MITOCHONDRIA | 42.81 | -4.76 | FALSE | TRUE | FALSE |
| **1944** | Q96SK2 | Transmembrane protein 209 | unknown | 23.68 | -12.29 | FALSE | TRUE | FALSE |
| **1945** | Q15477 | Helicase SKI2W | unknown | 8.34 | -20.74 | FALSE | TRUE | TRUE |
| **1946** | Q9NQS3 | Nectin-3 | unknown | -21.70 | 15.70 | TRUE | FALSE | FALSE |
| **1947** | O15091 | Mitochondrial ribonuclease P cataly | MITOCHONDRIA | 29.87 | -12.52 | FALSE | TRUE | FALSE |
| **1948** | Q6NUM9 | All-trans-retinol 13,14-reductase | ER | -5.84 | 33.13 | FALSE | TRUE | FALSE |
| **1949** | P41214 | Eukaryotic translation initiation fact | unknown | 1.82 | -16.13 | FALSE | TRUE | TRUE |
| **1950** | O60499 | Syntaxin-10 | unknown | -35.06 | 14.25 | TRUE | FALSE | FALSE |
| **1951** | O60942 | mRNA-capping enzyme | unknown | 0.16 | -14.30 | FALSE | TRUE | TRUE |
| **1952** | Q96GK7 | Fumarylacetoacetate hydrolase do | unknown | 22.06 | -3.76 | FALSE | TRUE | FALSE |
| **1953** | O95104 | Splicing factor, arginine/serine-rich | NUCLEUS | 1.25 | -33.59 | FALSE | TRUE | FALSE |
| **1954** | O95235 | Kinesin-like protein KIF20A | unknown | -17.70 | -14.16 | FALSE | TRUE | TRUE |
| **1955** | Q6NW34 | Nucleolus and neural progenitor pro | NUCLEUS | -4.63 | -30.81 | FALSE | TRUE | TRUE |
| **1956** | Q8NBK3 | Sulfatase-modifying factor 1 | unknown | -5.37 | 26.17 | FALSE | FALSE | TRUE |
| **1957** | Q96ME7 | Zinc finger protein 512 | NUCLEUS-CHROMATIN | -19.31 | -14.85 | TRUE | TRUE | TRUE |
| **1958** | Q92995 | Ubiquitin carboxyl-terminal hydrola | unknown | 17.46 | 9.14 | FALSE | TRUE | TRUE |
| **1959** | Q12768 | WASH complex subunit 5 | unknown | -14.37 | 9.11 | TRUE | TRUE | TRUE |
| **1960** | Q6DKJ4 | Nucleoredoxin | unknown | 10.59 | 6.29 | FALSE | TRUE | TRUE |
| **1961** | P82912 | 28S ribosomal protein S11, mitocho | MITOCHONDRIA | 39.97 | -5.31 | FALSE | TRUE | FALSE |
| **1962** | Q9Y2Z4 | Tyrosine--tRNA ligase, mitochondria | MITOCHONDRIA | 30.30 | -15.19 | FALSE | TRUE | FALSE |
| **1963** | Q6P1N0 | Coiled-coil and C2 domain-containir | unknown | 3.93 | -14.72 | FALSE | TRUE | FALSE |
| **1964** | P36543 | V-type proton ATPase subunit E 1 | unknown | -17.01 | 5.10 | FALSE | TRUE | FALSE |
| **1965** | Q9Y223 | Bifunctional UDP-N-acetylglucosam | unknown | 8.92 | 2.44 | FALSE | TRUE | FALSE |
| **1966** | O95671 | N-acetylserotonin O-methyltransfe | unknown | 10.71 | 9.49 | FALSE | TRUE | TRUE |
| **1967** | Q68CZ2 | Tensin-3 | unknown | -4.64 | 6.16 | TRUE | TRUE | TRUE |
| **1968** | Q9NZC9 | SWI/SNF-related matrix-associated | unknown | -11.65 | -15.64 | FALSE | TRUE | FALSE |
| **1969** | Q6YP21 | Kynurenine--oxoglutarate transami | unknown | 21.16 | -3.64 | FALSE | TRUE | FALSE |
| **1970** | Q03164 | Histone-lysine N-methyltransferase | unknown | -17.83 | -13.81 | TRUE | TRUE | TRUE |
| **1971** | Q96L91 | E1A-binding protein p400 | unknown | -10.77 | -22.33 | TRUE | TRUE | TRUE |
| **1972** | Q92738 | USP6 N-terminal-like protein | unknown | -27.93 | 0.00 | TRUE | FALSE | TRUE |

| | | | | | | | | |
|---|---|---|---|---|---|---|---|---|
| **1973** | P04150 | Glucocorticoid receptor | NUCLEUS | 10.53 | 0.04 | TRUE | TRUE | TRUE |
| **1974** | O15321 | Transmembrane 9 superfamily mer | unknown | -21.86 | 31.50 | FALSE | FALSE | TRUE |
| **1975** | O75717 | WD repeat and HMG-box DNA-binc | NUCLEUS | -8.82 | -14.68 | TRUE | FALSE | TRUE |
| **1976** | Q9BSJ2 | Gamma-tubulin complex componer | unknown | 6.24 | -24.80 | FALSE | TRUE | FALSE |
| **1977** | P08134 | Rho-related GTP-binding protein Rh | unknown | -18.68 | 2.91 | FALSE | FALSE | TRUE |
| **1978** | Q12913 | Receptor-type tyrosine-protein pho | PM | -26.99 | 10.87 | TRUE | FALSE | TRUE |
| **1979** | P16144 | Integrin beta-4 | PM | -25.54 | 7.33 | FALSE | TRUE | TRUE |
| **1980** | Q96BN8 | Ubiquitin thioesterase otulin | CYTOSOL | 16.98 | 9.64 | FALSE | TRUE | TRUE |
| **1981** | Q9H2V7 | Protein spinster homolog 1 | unknown | -35.41 | 8.83 | FALSE | TRUE | TRUE |
| **1982** | O00161 | Synaptosomal-associated protein 2 | PM | -23.19 | 16.37 | TRUE | FALSE | TRUE |
| **1983** | Q9Y3L5 | Ras-related protein Rap-2c | unknown | -28.08 | 1.80 | FALSE | TRUE | FALSE |
| **1984** | O95721 | Synaptosomal-associated protein 2 | PM | -22.29 | 11.67 | TRUE | FALSE | TRUE |
| **1985** | Q15042 | Rab3 GTPase-activating protein cat | unknown | 3.97 | 10.62 | FALSE | TRUE | FALSE |
| **1986** | Q86YP4 | Transcriptional repressor p66-alpha | NUCLEUS | -23.94 | -26.67 | TRUE | TRUE | TRUE |
| **1987** | Q27J81 | Inverted formin-2 | NUCLEUS | 1.91 | -2.36 | TRUE | TRUE | TRUE |
| **1988** | Q08AF3 | Schlafen family member 5 | unknown | -12.11 | -11.22 | FALSE | TRUE | FALSE |
| **1989** | Q96BI3 | Gamma-secretase subunit APH-1A | unknown | -16.08 | 16.67 | FALSE | TRUE | FALSE |
| **1990** | Q9ULC3 | Ras-related protein Rab-23 | PM | -23.08 | 10.73 | TRUE | TRUE | FALSE |
| **1991** | P07951 | Tropomyosin beta chain | unknown | 11.14 | 11.51 | FALSE | FALSE | TRUE |
| **1992** | P08397 | Porphobilinogen deaminase | CYTOSOL | 18.60 | 21.36 | FALSE | TRUE | FALSE |
| **1993** | Q13443 | Disintegrin and metalloproteinase c | PM | -30.71 | 6.70 | TRUE | TRUE | TRUE |
| **1994** | Q8WX93 | Palladin | unknown | -6.17 | -1.43 | TRUE | FALSE | TRUE |
| **1995** | Q07617 | Sperm-associated antigen 1 | unknown | -17.79 | 8.08 | TRUE | FALSE | TRUE |
| **1996** | Q9Y6M7 | Sodium bicarbonate cotransporter 3 | unknown | -23.80 | 3.89 | TRUE | FALSE | TRUE |
| **1997** | P26572 | Alpha-1,3-mannosyl-glycoprotein 2 | GOLGI | -18.74 | 32.51 | FALSE | TRUE | FALSE |
| **1998** | Q7Z5K2 | Wings apart-like protein homolog | NUCLEUS | -25.16 | -26.20 | TRUE | FALSE | TRUE |
| **1999** | Q8IZL8 | Proline-, glutamic acid- and leucine- | NUCLEUS | 1.14 | -34.88 | FALSE | TRUE | TRUE |
| **2000** | P16278 | Beta-galactosidase | LYSOSOME | -40.54 | 6.72 | FALSE | TRUE | FALSE |
| **2001** | Q9Y6M9 | NADH dehydrogenase [ubiquinone] | MITOCHONDRIA | 30.31 | -13.68 | FALSE | TRUE | FALSE |
| **2002** | Q9BYJ9 | YTH domain-containing family prot | unknown | 4.57 | -23.00 | TRUE | FALSE | FALSE |
| **2003** | Q9HD26 | Golgi-associated PDZ and coiled-coi | unknown | 3.53 | 0.18 | TRUE | FALSE | TRUE |
| **2004** | Q8NC60 | Nitric oxide-associated protein 1 | MITOCHONDRIA | 39.92 | -7.40 | FALSE | TRUE | TRUE |
| **2005** | Q92783 | Signal transducing adapter molecul | unknown | 2.48 | 12.03 | TRUE | FALSE | FALSE |
| **2006** | P48735 | Isocitrate dehydrogenase [NADP], r | MITOCHONDRIA | 43.13 | -7.47 | FALSE | TRUE | FALSE |
| **2007** | Q92974 | Rho guanine nucleotide exchange fa | unknown | 1.63 | -13.21 | FALSE | TRUE | TRUE |
| **2008** | Q9BVG9 | Phosphatidylserine synthase 2 | unknown | -6.49 | 22.21 | FALSE | TRUE | TRUE |
| **2009** | P23229 | Integrin alpha-6 | unknown | -32.18 | 11.13 | TRUE | TRUE | TRUE |
| **2010** | Q9Y5T5 | Ubiquitin carboxyl-terminal hydrola | unknown | 0.41 | -22.03 | FALSE | TRUE | FALSE |

| | | | | | | | | |
|---|---|---|---|---|---|---|---|---|
| **2011** | Q6P9B6 | TLD domain-containing protein 1 | unknown | -22.43 | 6.74 | FALSE | TRUE | FALSE |
| **2012** | P51812 | Ribosomal protein S6 kinase alpha- | unknown | 4.10 | 5.83 | FALSE | TRUE | TRUE |
| **2013** | Q9ULJ7 | Ankyrin repeat domain-containing | unknown | -5.10 | 6.56 | TRUE | FALSE | TRUE |
| **2014** | Q15819 | Ubiquitin-conjugating enzyme E2 va | unknown | 11.88 | 12.02 | FALSE | FALSE | TRUE |
| **2015** | P14678 | Small nuclear ribonucleoprotein-ass | NUCLEUS | 4.96 | -34.86 | FALSE | FALSE | TRUE |
| **2016** | P04920 | Anion exchange protein 2 | PM | -24.32 | 6.58 | TRUE | FALSE | TRUE |
| **2017** | Q96A65 | Exocyst complex component 4 | unknown | -7.26 | 0.69 | TRUE | TRUE | TRUE |
| **2018** | Q04721 | Neurogenic locus notch homolog pr | unknown | -19.62 | 13.24 | TRUE | TRUE | TRUE |
| **2019** | P18084 | Integrin beta-5 | PM | -23.07 | 15.23 | TRUE | FALSE | TRUE |
| **2020** | Q9P0K7 | Ankycorbin | unknown | -2.64 | 5.13 | TRUE | FALSE | TRUE |
| **2021** | Q9P0K7 | Ankycorbin | unknown | -2.84 | 4.23 | TRUE | FALSE | TRUE |
| **2022** | Q96JM3 | Chromosome alignment-maintainir | NUCLEUS | -23.50 | -27.20 | TRUE | FALSE | FALSE |
| **2023** | Q9H223 | EH domain-containing protein 4 | unknown | -10.94 | 11.94 | TRUE | TRUE | FALSE |
| **2024** | P50750 | Cyclin-dependent kinase 9 | NUCLEUS | 3.85 | -31.32 | FALSE | FALSE | TRUE |
| **2025** | Q658P3 | Metalloreductase STEAP3 | unknown | -34.37 | 11.31 | TRUE | FALSE | FALSE |
| **2026** | Q8IZT6 | Abnormal spindle-like microcephaly | unknown | 0.20 | -14.18 | FALSE | TRUE | FALSE |
| **2027** | Q15057 | Arf-GAP with coiled-coil, ANK repea | unknown | -7.25 | 4.33 | FALSE | TRUE | TRUE |
| **2028** | Q9NYV4 | Cyclin-dependent kinase 12 | NUCLEUS | 3.56 | -32.40 | TRUE | FALSE | TRUE |
| **2029** | O95155 | Ubiquitin conjugation factor E4 B | unknown | 10.72 | 11.01 | FALSE | TRUE | FALSE |
| **2030** | Q13492 | Phosphatidylinositol-binding clathri | unknown | -5.41 | -9.20 | TRUE | FALSE | TRUE |
| **2031** | O95239 | Chromosome-associated kinesin KI | NUCLEUS-CHROMATIN | -20.98 | -12.38 | TRUE | FALSE | TRUE |
| **2032** | Q9H0V1 | Transmembrane protein 168 | unknown | -22.29 | 29.22 | FALSE | TRUE | TRUE |
| **2033** | Q86YZ3 | Hornerin | unknown | -2.16 | 11.78 | FALSE | FALSE | TRUE |
| **2034** | P54725 | UV excision repair protein RAD23 ho | unknown | 13.26 | 24.72 | FALSE | FALSE | TRUE |
| **2035** | O60645 | Exocyst complex component 3 | unknown | -7.40 | 0.51 | TRUE | FALSE | FALSE |
| **2036** | Q96Q45 | Transmembrane protein 237 | unknown | -17.47 | 13.20 | TRUE | FALSE | FALSE |
| **2037** | Q9UIQ6 | Leucyl-cystinyl aminopeptidase | unknown | -35.81 | 14.57 | TRUE | FALSE | FALSE |
| **2038** | P23434 | Glycine cleavage system H protein, | MITOCHONDRIA | 33.46 | -8.18 | FALSE | FALSE | TRUE |
| **2039** | Q8IWZ3 | Ankyrin repeat and KH domain-con | unknown | 8.56 | -13.07 | TRUE | FALSE | TRUE |
| **2040** | P83916 | Chromobox protein homolog 1 | NUCLEUS | -15.95 | -29.62 | TRUE | FALSE | TRUE |
| **2041** | P51003 | Poly(A) polymerase alpha | unknown | 18.23 | 5.37 | FALSE | TRUE | FALSE |
| **2042** | P63096 | Guanine nucleotide-binding protein | unknown | -28.99 | 10.20 | FALSE | FALSE | TRUE |
| **2043** | Q86W92 | Liprin-beta-1 | unknown | -2.40 | 0.59 | TRUE | FALSE | TRUE |
| **2044** | Q6UW63 | KDEL motif-containing protein 1 | unknown | -0.98 | 38.03 | FALSE | TRUE | TRUE |
| **2045** | Q01469 | Fatty acid-binding protein, epiderm | unknown | 19.13 | 24.17 | TRUE | FALSE | TRUE |
| **2046** | Q2PPJ7 | Ral GTPase-activating protein subu | unknown | -5.94 | 3.05 | FALSE | FALSE | TRUE |
| **2047** | Q96PD2 | Discoidin, CUB and LCCL domain-con | PM | -26.27 | 1.82 | TRUE | FALSE | TRUE |
| **2048** | Q9BZF9 | Uveal autoantigen with coiled-coil c | unknown | -2.26 | 4.21 | TRUE | FALSE | TRUE |

| | | | | | | | | |
|---|---|---|---|---|---|---|---|---|
| **2049** | P46531 | Neurogenic locus notch homolog pr | unknown | -24.02 | 6.49 | TRUE | FALSE | TRUE |
| **2050** | Q9UJF2 | Ras GTPase-activating protein nGAI | unknown | -6.23 | 3.08 | TRUE | FALSE | TRUE |
| **2051** | Q96R06 | Sperm-associated antigen 5 | NUCLEUS | -0.99 | -5.08 | TRUE | FALSE | FALSE |
| **2052** | Q9BY42 | Protein RTF2 homolog | NUCLEUS | -12.46 | -15.47 | TRUE | TRUE | TRUE |
| **2053** | Q92609 | TBC1 domain family member 5 | unknown | -12.59 | 5.94 | TRUE | FALSE | FALSE |
| **2054** | Q9Y4W6 | AFG3-like protein 2 | MITOCHONDRIA | 34.26 | -11.68 | FALSE | TRUE | FALSE |
| **2055** | P35240 | Merlin | unknown | -17.21 | 13.49 | TRUE | FALSE | TRUE |
| **2056** | Q15334 | Lethal(2) giant larvae protein homo | unknown | -20.36 | 5.39 | TRUE | FALSE | TRUE |
| **2057** | P60903 | Protein S100-A10 | unknown | -11.11 | 30.31 | FALSE | FALSE | TRUE |
| **2058** | Q01974 | Tyrosine-protein kinase transmemb | PM | -25.69 | 3.54 | TRUE | FALSE | TRUE |
| **2059** | Q8IW52 | SLIT and NTRK-like protein 4 | unknown | -20.21 | 10.94 | TRUE | FALSE | TRUE |
| **2060** | O96013 | Serine/threonine-protein kinase PA | unknown | -5.65 | -1.21 | TRUE | FALSE | TRUE |
| **2061** | Q9H7C9 | Mth938 domain-containing protein | unknown | 18.83 | 11.29 | TRUE | FALSE | FALSE |
| **2062** | O75976 | Carboxypeptidase D | unknown | -36.13 | 14.02 | TRUE | FALSE | TRUE |
| **2063** | Q9HCS7 | Pre-mRNA-splicing factor SYF1 | NUCLEUS | -3.73 | -38.89 | TRUE | FALSE | TRUE |
| **2064** | Q99986 | Serine/threonine-protein kinase VR | NUCLEUS-CHROMATIN | -19.22 | -13.92 | FALSE | TRUE | FALSE |
| **2065** | P63092 | Guanine nucleotide-binding protein | unknown | -23.58 | 18.03 | FALSE | FALSE | TRUE |
| **2066** | Q9Y618 | Nuclear receptor corepressor 2 | NUCLEUS | -2.30 | -31.59 | TRUE | FALSE | FALSE |
| **2067** | Q53LP3 | Ankyrin repeat domain-containing | unknown | 2.58 | 4.29 | TRUE | FALSE | TRUE |
| **2068** | P01892 | HLA class I histocompatibility antige | PM | -27.65 | 12.19 | TRUE | FALSE | TRUE |
| **2069** | Q9NRL2 | Bromodomain adjacent to zinc finge | unknown | -21.81 | -20.13 | FALSE | FALSE | TRUE |
| **2070** | Q5T5Y3 | Calmodulin-regulated spectrin-asso | NUCLEUS | -9.96 | -6.60 | TRUE | FALSE | FALSE |
| **2071** | Q9NZN8 | CCR4-NOT transcription complex su | unknown | 8.48 | -25.69 | TRUE | FALSE | FALSE |
| **2072** | O75477 | Erlin-1 | ER | -9.19 | 31.83 | FALSE | FALSE | TRUE |
| **2073** | Q56VL3 | OCIA domain-containing protein 2 | MITOCHONDRIA | 31.64 | -12.90 | FALSE | TRUE | FALSE |
| **2074** | Q15599 | Na(+)/H(+) exchange regulatory co | unknown | -24.04 | 4.18 | TRUE | FALSE | FALSE |
| **2075** | P55011 | Solute carrier family 12 member 2 | PM | -26.66 | 16.38 | TRUE | FALSE | FALSE |
| **2076** | Q9P2N5 | RNA-binding protein 27 | NUCLEUS | -0.55 | -32.68 | TRUE | FALSE | TRUE |
| **2077** | Q9UQB8 | Brain-specific angiogenesis inhibitor | unknown | -8.04 | 13.98 | TRUE | FALSE | FALSE |
| **2078** | Q15811 | Intersectin-1 | unknown | -5.69 | 8.46 | TRUE | FALSE | FALSE |
| **2079** | P06213 | Insulin receptor | PM | -24.28 | 12.46 | TRUE | FALSE | FALSE |
| **2080** | Q96JM7 | Lethal(3)malignant brain tumor-like | unknown | -33.51 | -1.84 | TRUE | FALSE | FALSE |
| **2081** | Q9H2U1 | ATP-dependent RNA helicase DHX3 | NUCLEUS | -0.91 | -22.78 | FALSE | TRUE | TRUE |
| **2082** | P28799 | Granulins | unknown | -37.53 | 13.48 | FALSE | FALSE | TRUE |
| **2083** | P60468 | Protein transport protein Sec61 sub | ER | 2.37 | 29.35 | FALSE | FALSE | TRUE |
| **2084** | O15440 | Multidrug resistance-associated pro | PM | -32.90 | 13.50 | TRUE | FALSE | TRUE |
| **2085** | P05165 | Propionyl-CoA carboxylase alpha ch | MITOCHONDRIA | 35.72 | -4.91 | TRUE | FALSE | TRUE |
| **2086** | Q96T37 | Putative RNA-binding protein 15 | NUCLEUS | -1.16 | -34.92 | FALSE | TRUE | TRUE |

| | | | | | | | | |
|---|---|---|---|---|---|---|---|---|
| **2087** | Q8NCN4 | E3 ubiquitin-protein ligase RNF169 | unknown | -6.49 | -22.81 | TRUE | FALSE | FALSE |
| **2088** | Q9BQ48 | 39S ribosomal protein L34, mitocho | unknown | 42.69 | -2.06 | TRUE | FALSE | TRUE |
| **2089** | Q9GZU8 | Protein FAM192A | unknown | 27.42 | 23.04 | TRUE | FALSE | FALSE |
| **2090** | O14925 | Mitochondrial import inner membra | MITOCHONDRIA | 36.22 | -1.24 | FALSE | FALSE | TRUE |
| **2091** | Q7L2E3 | Putative ATP-dependent RNA helica | unknown | -7.93 | -3.13 | FALSE | TRUE | TRUE |
| **2092** | Q9Y520 | Protein PRRC2C | unknown | 6.86 | -20.04 | TRUE | FALSE | TRUE |
| **2093** | P61769 | Beta-2-microglobulin | PM | -27.94 | 12.67 | FALSE | FALSE | TRUE |
| **2094** | O43432 | Eukaryotic translation initiation fact | unknown | 4.74 | -18.46 | TRUE | FALSE | TRUE |
| **2095** | Q5UIP0 | Telomere-associated protein RIF1 | NUCLEUS-CHROMATIN | -20.84 | -15.11 | TRUE | FALSE | TRUE |
| **2096** | Q9P206 | Uncharacterized protein KIAA1522 | unknown | -22.38 | 4.06 | TRUE | FALSE | FALSE |
| **2097** | Q9Y2S6 | Translation machinery-associated p | unknown | -6.43 | -19.29 | FALSE | FALSE | TRUE |
| **2098** | O75581 | Low-density lipoprotein receptor-re | PM | -34.21 | 3.93 | TRUE | FALSE | FALSE |
| **2099** | O43663 | Protein regulator of cytokinesis 1 | unknown | -15.97 | -12.51 | FALSE | TRUE | FALSE |
| **2100** | Q9UPN3 | Microtubule-actin cross-linking fact | unknown | -3.54 | 2.40 | TRUE | FALSE | TRUE |
| **2101** | Q9UPN3 | Microtubule-actin cross-linking fact | unknown | -3.54 | 2.11 | TRUE | FALSE | TRUE |
| **2102** | Q6KC79 | Nipped-B-like protein | NUCLEUS-CHROMATIN | -21.24 | -13.69 | TRUE | FALSE | TRUE |
| **2103** | Q13873 | Bone morphogenetic protein recept | PM | -22.90 | 12.03 | TRUE | FALSE | TRUE |
| **2104** | Q9UGU0 | Transcription factor 20 | unknown | -18.87 | -23.70 | TRUE | FALSE | FALSE |
| **2105** | Q96RL1 | BRCA1-A complex subunit RAP80 | unknown | -15.98 | -19.93 | TRUE | FALSE | FALSE |
| **2106** | Q13190 | Syntaxin-5 | unknown | -11.11 | 32.25 | TRUE | FALSE | FALSE |
| **2107** | P20674 | Cytochrome c oxidase subunit 5A, n | MITOCHONDRIA | 33.43 | -11.52 | FALSE | FALSE | TRUE |
| **2108** | Q6P4E1 | Protein CASC4 | GOLGI | -21.12 | 31.65 | TRUE | FALSE | FALSE |
| **2109** | Q06546 | GA-binding protein alpha chain | NUCLEUS | -15.89 | -28.51 | TRUE | FALSE | FALSE |
| **2110** | Q99747 | Gamma-soluble NSF attachment pr | unknown | -33.35 | 14.48 | TRUE | FALSE | FALSE |
| **2111** | Q96FZ7 | Charged multivesicular body protei | PM | -27.30 | 8.20 | TRUE | FALSE | FALSE |
| **2112** | Q86YS7 | C2 domain-containing protein 5 | unknown | -14.31 | 14.63 | TRUE | FALSE | TRUE |
| **2113** | P54252 | Ataxin-3 | unknown | 14.19 | 2.96 | TRUE | FALSE | FALSE |
| **2114** | Q8IVF2 | Protein AHNAK2 | unknown | -10.50 | 23.14 | TRUE | FALSE | TRUE |
| **2115** | Q92733 | Proline-rich protein PRCC | NUCLEUS | -14.45 | -24.62 | TRUE | FALSE | TRUE |
| **2116** | Q86Y07 | Serine/threonine-protein kinase VR | unknown | 5.94 | 33.25 | FALSE | TRUE | FALSE |
| **2117** | Q7L4I2 | Arginine/serine-rich coiled-coil prot | NUCLEUS | 1.55 | -28.08 | FALSE | FALSE | TRUE |
| **2118** | Q9ULH0 | Kinase D-interacting substrate of 22 | unknown | -19.91 | 15.57 | TRUE | FALSE | TRUE |
| **2119** | Q06787 | Synaptic functional regulator FMR1 | unknown | 8.42 | -25.76 | FALSE | FALSE | TRUE |
| **2120** | O75410 | Transforming acidic coiled-coil-cont | unknown | -3.73 | 12.74 | TRUE | FALSE | TRUE |
| **2121** | Q9C0C2 | 182 kDa tankyrase-1-binding prote | NUCLEUS | -1.02 | -4.69 | TRUE | FALSE | TRUE |
| **2122** | Q9Y4C4 | Malignant fibrous histiocytoma-am | unknown | -5.15 | 4.71 | FALSE | FALSE | TRUE |
| **2123** | Q99618 | Cell division cycle-associated protei | unknown | -23.56 | 5.34 | TRUE | FALSE | FALSE |
| **2124** | Q6UVK1 | Chondroitin sulfate proteoglycan 4 | PM | -27.60 | 1.68 | TRUE | FALSE | FALSE |

| | | | | | | | | |
|---|---|---|---|---|---|---|---|---|
| **2125** | P33981 | Dual specificity protein kinase TTK | unknown | -5.33 | -9.03 | TRUE | FALSE | TRUE |
| **2126** | Q9BZD4 | Kinetochore protein Nuf2 | NUCLEUS | -3.74 | -13.82 | FALSE | FALSE | TRUE |
| **2127** | Q9P246 | Stromal interaction molecule 2 | ER | -5.27 | 26.98 | TRUE | FALSE | FALSE |
| **2128** | Q9UMZ2 | Synergin gamma | unknown | -14.94 | 8.67 | TRUE | FALSE | FALSE |
| **2129** | Q8WY22 | BRI3-binding protein | unknown | 3.13 | 34.15 | FALSE | FALSE | TRUE |
| **2130** | P07919 | Cytochrome b-c1 complex subunit 6 | MITOCHONDRIA | 42.37 | -2.98 | FALSE | FALSE | TRUE |
| **2131** | Q8IWZ8 | SURP and G-patch domain-containi | NUCLEUS | -1.85 | -36.41 | TRUE | FALSE | FALSE |
| **2132** | P27448 | MAP/microtubule affinity-regulatin | unknown | 2.10 | -10.43 | TRUE | FALSE | TRUE |
| **2133** | Q9BRD0 | BUD13 homolog | NUCLEUS | 2.78 | -33.54 | TRUE | FALSE | FALSE |
| **2134** | Q92540 | Protein SMG7 | unknown | 2.06 | -14.27 | TRUE | FALSE | FALSE |
| **2135** | O95466 | Formin-like protein 1 | unknown | -13.77 | 14.55 | TRUE | FALSE | FALSE |
| **2136** | Q9Y4B5 | Microtubule cross-linking factor 1 | unknown | 0.63 | -10.23 | TRUE | FALSE | FALSE |
| **2137** | O94854 | Uncharacterized protein KIAA0754 | unknown | -10.57 | 23.51 | TRUE | FALSE | FALSE |
| **2138** | Q9NPJ3 | Acyl-coenzyme A thioesterase 13 | unknown | -4.98 | 20.05 | FALSE | FALSE | TRUE |
| **2139** | O43765 | Small glutamine-rich tetratricopepti | CYTOSOL | 23.88 | 22.79 | FALSE | FALSE | TRUE |
| **2140** | Q8N201 | Integrator complex subunit 1 | unknown | -14.59 | -21.96 | TRUE | FALSE | FALSE |
| **2141** | Q99471 | Prefoldin subunit 5 | unknown | 24.94 | 23.56 | FALSE | FALSE | TRUE |
| **2142** | Q8NFX7 | Syntaxin-binding protein 6 | unknown | -23.84 | 5.95 | TRUE | FALSE | FALSE |
| **2143** | Q5T2T1 | MAGUK p55 subfamily member 7 | unknown | -16.99 | 16.58 | TRUE | FALSE | FALSE |
| **2144** | Q9NYJ8 | TGF-beta-activated kinase 1 and M | unknown | -3.05 | 8.19 | TRUE | FALSE | FALSE |
| **2145** | Q13123 | Protein Red | NUCLEUS | -4.09 | -38.93 | TRUE | FALSE | TRUE |
| **2146** | Q9H6A9 | Pecanex-like protein 3 | unknown | -17.06 | 14.49 | TRUE | FALSE | TRUE |
| **2147** | Q76FK4 | Nucleolar protein 8 | NUCLEUS | -4.19 | -36.31 | TRUE | FALSE | TRUE |
| **2148** | P09132 | Signal recognition particle 19 kDa p | unknown | 5.33 | -4.06 | FALSE | FALSE | TRUE |
| **2149** | O75937 | DnaJ homolog subfamily C member | NUCLEUS | -13.60 | -29.56 | FALSE | FALSE | TRUE |
| **2150** | Q9ULJ8 | Neurabin-1 | unknown | -2.06 | 1.96 | TRUE | FALSE | TRUE |
| **2151** | Q96T23 | Remodeling and spacing factor 1 | NUCLEUS | -15.63 | -29.26 | TRUE | FALSE | FALSE |
| **2152** | Q14011 | Cold-inducible RNA-binding protein | NUCLEUS | -17.94 | -32.57 | FALSE | FALSE | TRUE |
| **2153** | Q92797 | Symplekin | NUCLEUS | -10.12 | -35.84 | FALSE | FALSE | TRUE |
| **2154** | Q9H0G5 | Nuclear speckle splicing regulatory | NUCLEUS | -1.30 | -23.52 | TRUE | FALSE | FALSE |
| **2155** | P61011 | Signal recognition particle 54 kDa p | unknown | 5.20 | -4.20 | TRUE | FALSE | FALSE |
| **2156** | O60568 | Procollagen-lysine,2-oxoglutarate 5 | ER | 4.57 | 39.08 | FALSE | TRUE | TRUE |
| **2157** | Q8IUW5 | RELT-like protein 1 | PM | -24.23 | 4.53 | TRUE | FALSE | FALSE |
| **2158** | Q14696 | LRP chaperone MESD | unknown | -1.84 | 39.37 | FALSE | FALSE | TRUE |
| **2159** | Q53EZ4 | Centrosomal protein of 55 kDa | unknown | 3.38 | -11.60 | TRUE | FALSE | FALSE |
| **2160** | Q9HCE1 | Putative helicase MOV-10 | unknown | 4.19 | -22.44 | FALSE | TRUE | TRUE |
| **2161** | Q96C24 | Synaptotagmin-like protein 4 | unknown | -17.69 | 13.35 | TRUE | FALSE | FALSE |
| **2162** | Q13098 | COP9 signalosome complex subunit | unknown | 19.48 | 11.74 | FALSE | FALSE | TRUE |

| | | | | | | | | |
|---|---|---|---|---|---|---|---|---|
| **2163** | Q9Y4E1 | WASH complex subunit 2C | unknown | -15.65 | 10.38 | TRUE | FALSE | TRUE |
| **2164** | Q96FQ6 | Protein S100-A16 | NUCLEUS | 2.38 | 6.19 | FALSE | FALSE | TRUE |
| **2165** | Q08AD1 | Calmodulin-regulated spectrin-asso | unknown | -3.47 | -6.45 | TRUE | FALSE | TRUE |
| **2166** | P09669 | Cytochrome c oxidase subunit 6C | MITOCHONDRIA | 27.53 | -10.73 | FALSE | FALSE | TRUE |
| **2167** | Q9UHD9 | Ubiquilin-2 | unknown | 20.63 | 13.32 | FALSE | FALSE | TRUE |
| **2168** | O43583 | Density-regulated protein | unknown | -11.60 | -10.79 | FALSE | FALSE | TRUE |
| **2169** | Q96CB8 | Integrator complex subunit 12 | unknown | -14.82 | -21.03 | TRUE | FALSE | TRUE |
| **2170** | O43760 | Synaptogyrin-2 | PM | -34.78 | 16.94 | FALSE | FALSE | TRUE |
| **2171** | Q8TF68 | Zinc finger protein 384 | NUCLEUS | -23.35 | -23.33 | TRUE | FALSE | FALSE |
| **2172** | Q9Y6D5 | Brefeldin A-inhibited guanine nucle | unknown | -2.67 | -11.14 | TRUE | FALSE | FALSE |
| **2173** | Q3V6T2 | Girdin | unknown | -1.98 | -7.28 | TRUE | FALSE | TRUE |
| **2174** | P33240 | Cleavage stimulation factor subunit | NUCLEUS | -11.22 | -35.67 | FALSE | FALSE | TRUE |
| **2175** | Q92917 | G-patch domain and KOW motifs-c | NUCLEUS | -14.79 | -29.97 | TRUE | FALSE | TRUE |
| **2176** | Q92520 | Protein FAM3C | unknown | -22.34 | 30.75 | FALSE | FALSE | TRUE |
| **2177** | Q8IWW6 | Rho GTPase-activating protein 12 | unknown | -13.89 | 13.44 | TRUE | FALSE | TRUE |
| **2178** | O75915 | PRA1 family protein 3 | unknown | -2.80 | 28.72 | FALSE | FALSE | TRUE |
| **2179** | Q9H9B1 | Histone-lysine N-methyltransferase | unknown | -22.85 | -18.48 | TRUE | FALSE | TRUE |
| **2180** | Q15032 | R3H domain-containing protein 1 | unknown | 4.75 | -8.76 | TRUE | FALSE | FALSE |
| **2181** | Q9H6X2 | Anthrax toxin receptor 1 | PM | -32.62 | -0.64 | TRUE | FALSE | TRUE |
| **2182** | Q9Y333 | U6 snRNA-associated Sm-like prote | NUCLEUS | -9.95 | -30.57 | FALSE | FALSE | TRUE |
| **2183** | P52943 | Cysteine-rich protein 2 | unknown | -8.27 | 6.62 | TRUE | FALSE | FALSE |
| **2184** | Q9Y217 | Myotubularin-related protein 6 | unknown | 5.04 | -11.10 | TRUE | FALSE | FALSE |
| **2185** | Q96QR8 | Transcriptional activator protein Pu | unknown | 13.26 | 0.80 | TRUE | FALSE | TRUE |
| **2186** | Q99614 | Tetratricopeptide repeat protein 1 | unknown | 17.11 | 14.16 | TRUE | FALSE | TRUE |
| **2187** | Q5T6F2 | Ubiquitin-associated protein 2 | unknown | 8.22 | -21.32 | TRUE | FALSE | TRUE |
| **2188** | O75431 | Metaxin-2 | unknown | 28.77 | -14.90 | FALSE | FALSE | TRUE |
| **2189** | Q9H501 | ESF1 homolog | NUCLEUS | -4.94 | -38.69 | TRUE | FALSE | FALSE |
| **2190** | Q13488 | V-type proton ATPase 116 kDa sub | LYSOSOME | -39.40 | 8.76 | TRUE | FALSE | FALSE |
| **2191** | Q86VM9 | Zinc finger CCCH domain-containing | NUCLEUS | -12.78 | -28.73 | TRUE | FALSE | TRUE |
| **2192** | Q5JSZ5 | Protein PRRC2B | unknown | 4.91 | -21.48 | TRUE | FALSE | TRUE |
| **2193** | Q9NZT2 | Opioid growth factor receptor | unknown | 15.44 | 23.24 | FALSE | FALSE | TRUE |
| **2194** | P08174 | Complement decay-accelerating fa | PM | -23.23 | 16.81 | FALSE | FALSE | TRUE |
| **2195** | O14907 | Tax1-binding protein 3 | unknown | 3.78 | 0.93 | FALSE | FALSE | TRUE |
| **2196** | Q9UNN5 | FAS-associated factor 1 | unknown | 18.92 | 15.66 | FALSE | FALSE | TRUE |
| **2197** | Q9UHB7 | AF4/FMR2 family member 4 | unknown | -14.72 | -18.84 | TRUE | FALSE | FALSE |
| **2198** | Q8IWI9 | MAX gene-associated protein | NUCLEUS | -24.18 | -22.73 | TRUE | FALSE | FALSE |
| **2199** | P20962 | Parathymosin | unknown | 27.15 | 19.40 | FALSE | FALSE | TRUE |
| **2200** | Q9H6F5 | Coiled-coil domain-containing prote | NUCLEUS | -4.08 | -32.76 | FALSE | FALSE | TRUE |

| | | | | | | | | |
|---|---|---|---|---|---|---|---|---|
| **2201** | Q8ND82 | Zinc finger protein 280C | NUCLEUS-CHROMATIN | -21.37 | -14.60 | TRUE | FALSE | TRUE |
| **2202** | Q9HD33 | 39S ribosomal protein L47, mitocho | MITOCHONDRIA | 40.87 | -7.15 | FALSE | FALSE | TRUE |
| **2203** | Q9Y2X7 | ARF GTPase-activating protein GIT1 | unknown | -3.24 | -6.38 | TRUE | FALSE | FALSE |
| **2204** | Q9UEY8 | Gamma-adducin | NUCLEUS | -10.90 | -17.69 | TRUE | FALSE | TRUE |
| **2205** | P13861 | cAMP-dependent protein kinase ty | unknown | -19.70 | 5.65 | TRUE | FALSE | TRUE |
| **2206** | Q70E73 | Ras-associated and pleckstrin hom | unknown | -3.29 | 10.45 | TRUE | FALSE | FALSE |
| **2207** | Q9NRX1 | RNA-binding protein PNO1 | NUCLEUS | -0.88 | -37.21 | FALSE | FALSE | TRUE |
| **2208** | O95630 | STAM-binding protein | unknown | 9.94 | 14.21 | TRUE | FALSE | FALSE |
| **2209** | Q13625 | Apoptosis-stimulating of p53 protei | unknown | -7.41 | 7.00 | TRUE | FALSE | FALSE |
| **2210** | Q9H6S0 | Probable ATP-dependent RNA helic | unknown | 1.67 | -15.75 | FALSE | TRUE | TRUE |
| **2211** | Q9Y3D3 | 28S ribosomal protein S16, mitocho | unknown | 44.41 | -13.82 | FALSE | FALSE | TRUE |
| **2212** | Q92576 | PHD finger protein 3 | unknown | -14.64 | -22.19 | TRUE | FALSE | TRUE |
| **2213** | Q9NUP9 | Protein lin-7 homolog C | unknown | -21.30 | 11.06 | TRUE | FALSE | FALSE |
| **2214** | Q8NHQ9 | ATP-dependent RNA helicase DDX5 | NUCLEUS | 2.40 | -27.39 | TRUE | FALSE | FALSE |
| **2215** | P84157 | Matrix-remodeling-associated prot | unknown | -9.11 | 35.38 | TRUE | FALSE | FALSE |
| **2216** | Q12846 | Syntaxin-4 | PM | -24.54 | 15.69 | TRUE | FALSE | FALSE |
| **2217** | Q8TEA8 | D-aminoacyl-tRNA deacylase 1 | unknown | 8.93 | 0.70 | FALSE | FALSE | TRUE |
| **2218** | Q92575 | UBX domain-containing protein 4 | unknown | -12.49 | 29.80 | TRUE | FALSE | TRUE |
| **2219** | A2RU67 | Protein FAM234B | unknown | -36.55 | 11.90 | TRUE | FALSE | FALSE |
| **2220** | O43815 | Striatin | unknown | -9.89 | 13.87 | TRUE | FALSE | TRUE |
| **2221** | Q15773 | Myeloid leukemia factor 2 | unknown | -4.58 | -8.11 | FALSE | FALSE | TRUE |
| **2222** | Q8TDM6 | Disks large homolog 5 | unknown | -8.58 | 1.60 | TRUE | FALSE | TRUE |
| **2223** | Q9H582 | Zinc finger protein 644 | NUCLEUS-CHROMATIN | -22.25 | -18.11 | TRUE | FALSE | FALSE |
| **2224** | P42892 | Endothelin-converting enzyme 1 | unknown | -31.29 | 14.50 | TRUE | FALSE | FALSE |
| **2225** | Q9GZL7 | Ribosome biogenesis protein WDR1 | NUCLEUS | -9.59 | -31.19 | FALSE | FALSE | TRUE |
| **2226** | Q8IWB9 | Testis-expressed protein 2 | unknown | -11.72 | 28.79 | TRUE | FALSE | FALSE |
| **2227** | Q7Z2T5 | TRMT1-like protein | NUCLEUS | 0.70 | -24.75 | FALSE | FALSE | TRUE |
| **2228** | Q15121 | Astrocytic phosphoprotein PEA-15 | unknown | 14.01 | 23.58 | FALSE | FALSE | TRUE |
| **2229** | Q13416 | Origin recognition complex subunit | NUCLEUS-CHROMATIN | -22.28 | -17.10 | TRUE | FALSE | TRUE |
| **2230** | P29084 | Transcription initiation factor IIE sul | NUCLEUS | -5.38 | -15.31 | TRUE | FALSE | FALSE |
| **2231** | Q5VZK9 | F-actin-uncapping protein LRRC16A | unknown | -8.28 | 10.94 | TRUE | FALSE | TRUE |
| **2232** | O95835 | Serine/threonine-protein kinase LA | unknown | 0.49 | -9.28 | TRUE | FALSE | FALSE |
| **2233** | P98175 | RNA-binding protein 10 | NUCLEUS | 4.77 | -32.69 | TRUE | FALSE | TRUE |
| **2234** | Q8WWK9 | Cytoskeleton-associated protein 2 | unknown | -10.52 | -11.12 | FALSE | FALSE | TRUE |
| **2235** | Q8TD16 | Protein bicaudal D homolog 2 | unknown | -17.20 | 2.82 | TRUE | FALSE | TRUE |
| **2236** | Q7KZ85 | Transcription elongation factor SPT( | NUCLEUS | -17.10 | -30.01 | FALSE | FALSE | TRUE |
| **2237** | Q9H5V9 | UPF0428 protein CXorf56 | NUCLEUS | -1.94 | -26.80 | TRUE | FALSE | TRUE |
| **2238** | Q9UHY1 | Nuclear receptor-binding protein | unknown | 15.78 | 22.21 | FALSE | FALSE | TRUE |

| | | | | | | | | |
|---|---|---|---|---|---|---|---|---|
| **2239** | Q9Y3C1 | Nucleolar protein 16 | NUCLEUS | 2.07 | -36.20 | FALSE | FALSE | TRUE |
| **2240** | Q7Z4V5 | Hepatoma-derived growth factor-re | unknown | 13.32 | 24.88 | TRUE | FALSE | FALSE |
| **2241** | Q86V48 | Leucine zipper protein 1 | unknown | -7.80 | -4.32 | TRUE | FALSE | TRUE |
| **2242** | Q7LBC6 | Lysine-specific demethylase 3B | unknown | -11.89 | -16.27 | TRUE | FALSE | TRUE |
| **2243** | Q14966 | Zinc finger protein 638 | NUCLEUS | -13.97 | -22.63 | TRUE | FALSE | TRUE |
| **2244** | Q5SW79 | Centrosomal protein of 170 kDa | unknown | -11.19 | -16.39 | TRUE | FALSE | TRUE |
| **2245** | Q92896 | Golgi apparatus protein 1 | GOLGI | -20.89 | 30.82 | FALSE | FALSE | TRUE |
| **2246** | Q8WXI9 | Transcriptional repressor p66-beta | NUCLEUS | -26.01 | -27.03 | TRUE | FALSE | TRUE |
| **2247** | Q9H3Q1 | Cdc42 effector protein 4 | unknown | -5.76 | 10.46 | TRUE | FALSE | TRUE |
| **2248** | P62312 | U6 snRNA-associated Sm-like prote | NUCLEUS | -9.78 | -30.91 | FALSE | FALSE | TRUE |
| **2249** | Q13610 | Periodic tryptophan protein 1 homo | NUCLEUS | -5.90 | -25.74 | FALSE | FALSE | TRUE |
| **2250** | P05204 | Non-histone chromosomal protein H | NUCLEUS | -26.69 | -23.25 | FALSE | FALSE | TRUE |
| **2251** | Q8IUR0 | Trafficking protein particle complex | unknown | -11.32 | 6.22 | TRUE | FALSE | FALSE |
| **2252** | Q9C0C9 | (E3-independent) E2 ubiquitin-conju | unknown | 14.06 | 22.97 | FALSE | FALSE | TRUE |
| **2253** | P38935 | DNA-binding protein SMUBP-2 | unknown | 3.68 | -18.63 | FALSE | FALSE | TRUE |
| **2254** | Q6PJT7 | Zinc finger CCCH domain-containing | NUCLEUS | -6.31 | -38.93 | TRUE | FALSE | TRUE |
| **2255** | P08962 | CD63 antigen | unknown | -34.82 | 17.01 | FALSE | FALSE | TRUE |
| **2256** | O15020 | Spectrin beta chain, non-erythrocyt | unknown | 1.59 | 26.08 | TRUE | FALSE | TRUE |
| **2257** | P40222 | Alpha-taxilin | unknown | -11.33 | -10.62 | FALSE | FALSE | TRUE |
| **2258** | P09497 | Clathrin light chain B | unknown | -10.10 | 13.83 | FALSE | FALSE | TRUE |
| **2259** | Q96BZ9 | TBC1 domain family member 20 | unknown | -5.36 | 20.83 | FALSE | FALSE | TRUE |
| **2260** | O15305 | Phosphomannomutase 2 | unknown | 19.73 | 19.48 | FALSE | FALSE | TRUE |
| **2261** | Q9Y4I1 | Unconventional myosin-Va | unknown | -8.39 | -7.80 | FALSE | FALSE | TRUE |
| **2262** | Q99755 | Phosphatidylinositol 4-phosphate 5 | unknown | -23.19 | 1.95 | TRUE | FALSE | FALSE |
| **2263** | Q9NZM5 | Ribosome biogenesis protein NOP5 | NUCLEUS | 2.36 | -32.93 | FALSE | FALSE | TRUE |
| **2264** | P62310 | U6 snRNA-associated Sm-like prote | NUCLEUS | -10.02 | -30.71 | FALSE | FALSE | TRUE |
| **2265** | P49903 | Selenide, water dikinase 1 | NUCLEUS | -9.61 | -14.59 | FALSE | FALSE | TRUE |
| **2266** | Q14517 | Protocadherin Fat 1 | unknown | -17.76 | 18.68 | TRUE | FALSE | TRUE |
| **2267** | O94760 | N(G),N(G)-dimethylarginine dimeth | unknown | 9.43 | 16.33 | TRUE | FALSE | TRUE |
| **2268** | Q9H8Y8 | Golgi reassembly-stacking protein 2 | unknown | 21.58 | 19.86 | FALSE | FALSE | TRUE |
| **2269** | Q9NZZ3 | Charged multivesicular body protei | unknown | 8.71 | 13.99 | TRUE | FALSE | TRUE |
| **2270** | Q96K76 | Ubiquitin carboxyl-terminal hydrola | unknown | 17.68 | 13.14 | TRUE | FALSE | FALSE |
| **2271** | Q9BXF6 | Rab11 family-interacting protein 5 | unknown | -21.34 | 7.14 | TRUE | FALSE | FALSE |
| **2272** | Q92625 | Ankyrin repeat and SAM domain-co | unknown | -3.72 | 8.29 | TRUE | FALSE | TRUE |
| **2273** | Q6ZSR9 | Uncharacterized protein FLJ45252 | unknown | -6.36 | 8.40 | TRUE | FALSE | FALSE |
| **2274** | O60333 | Kinesin-like protein KIF1B | unknown | 3.28 | -16.17 | FALSE | FALSE | TRUE |
| **2275** | Q86UL3 | Glycerol-3-phosphate acyltransfera | ER | -9.46 | 30.57 | FALSE | FALSE | TRUE |
| **2276** | Q9P0V3 | SH3 domain-binding protein 4 | unknown | -18.95 | 14.59 | TRUE | FALSE | FALSE |

| | | | | | | | | | |
|---|---|---|---|---|---|---|---|---|---|
| **2277** | Q96C01 | Protein FAM136A | | MITOCHONDRIA | 36.73 | -1.59 | FALSE | FALSE | TRUE |
| **2278** | P27707 | Deoxycytidine kinase | | unknown | 19.38 | 22.03 | FALSE | FALSE | TRUE |
| **2279** | Q9BVC6 | Transmembrane protein 109 | | unknown | 1.37 | 31.38 | FALSE | FALSE | TRUE |
| **2280** | Q9C0D5 | Protein TANC1 | | unknown | 3.14 | -15.99 | TRUE | FALSE | TRUE |
| **2281** | Q16610 | Extracellular matrix protein 1 | | unknown | -7.89 | 28.88 | FALSE | FALSE | TRUE |
| **2282** | Q8IW35 | Centrosomal protein of 97 kDa | | unknown | 11.23 | 3.80 | TRUE | FALSE | FALSE |
| **2283** | Q6ZS17 | Rho family-interacting cell polarizat | | unknown | -29.15 | -0.22 | TRUE | FALSE | TRUE |
| **2284** | P20594 | Atrial natriuretic peptide receptor 2 | | PM | -22.06 | 15.49 | FALSE | FALSE | TRUE |
| **2285** | Q9H910 | Jupiter microtubule associated hom | | unknown | 2.10 | 6.66 | TRUE | FALSE | TRUE |
| **2286** | O95400 | CD2 antigen cytoplasmic tail-bindin | | NUCLEUS | -10.00 | -30.46 | TRUE | FALSE | TRUE |
| **2287** | Q9BZE9 | Tether containing UBX domain for G | | unknown | 11.35 | 11.45 | FALSE | FALSE | TRUE |
| **2288** | Q13444 | Disintegrin and metalloproteinase d | | PM | -26.13 | 4.45 | TRUE | FALSE | FALSE |
| **2289** | P49674 | Casein kinase I isoform epsilon | | unknown | 7.27 | -11.07 | FALSE | FALSE | TRUE |
| **2290** | P35520 | Cystathionine beta-synthase | | unknown | 14.92 | 16.19 | FALSE | FALSE | TRUE |
| **2291** | O94901 | SUN domain-containing protein 1 | | unknown | 23.63 | -12.58 | FALSE | FALSE | TRUE |
| **2292** | Q8N3D4 | EH domain-binding protein 1-like pr | | unknown | -16.14 | 12.59 | TRUE | FALSE | FALSE |
| **2293** | P62942 | Peptidyl-prolyl cis-trans isomerase I | | unknown | 23.35 | 24.24 | FALSE | FALSE | TRUE |
| **2294** | Q9NZ63 | Telomere length and silencing prote | | NUCLEUS | -9.50 | -29.24 | TRUE | FALSE | FALSE |
| **2295** | Q99590 | Protein SCAF11 | | NUCLEUS | -17.31 | -24.12 | TRUE | FALSE | TRUE |
| **2296** | Q8IZP0 | Abl interactor 1 | | unknown | -7.16 | 13.33 | TRUE | FALSE | TRUE |
| **2297** | Q9Y5L4 | Mitochondrial import inner membra | | MITOCHONDRIA | 33.79 | -14.86 | FALSE | FALSE | TRUE |
| **2298** | Q00059 | Transcription factor A, mitochondria | | unknown | 27.33 | -11.54 | FALSE | FALSE | TRUE |
| **2299** | Q14197 | Peptidyl-tRNA hydrolase ICT1, mito | | MITOCHONDRIA | 42.01 | -5.51 | FALSE | FALSE | TRUE |
| **2300** | Q8NE01 | Metal transporter CNNM3 | | PM | -21.56 | 18.53 | TRUE | FALSE | TRUE |
| **2301** | P31431 | Syndecan-4 | | unknown | -38.75 | 10.98 | TRUE | FALSE | TRUE |
| **2302** | Q96QE3 | ATPase family AAA domain-contair | | unknown | -15.86 | -13.44 | TRUE | FALSE | TRUE |
| **2303** | P41236 | Protein phosphatase inhibitor 2 | | CYTOSOL | 15.48 | 23.07 | TRUE | FALSE | TRUE |
| **2304** | O95208 | Epsin-2 | | unknown | -10.87 | 24.73 | TRUE | FALSE | FALSE |
| **2305** | O43768 | Alpha-endosulfine | | unknown | 10.14 | 20.58 | TRUE | FALSE | TRUE |
| **2306** | Q8N3X1 | Formin-binding protein 4 | | NUCLEUS | -6.98 | -30.46 | TRUE | FALSE | TRUE |
| **2307** | Q15648 | Mediator of RNA polymerase II trar | | NUCLEUS | -1.70 | -30.70 | TRUE | FALSE | TRUE |
| **2308** | O14976 | Cyclin-G-associated kinase | | unknown | -3.38 | 9.50 | FALSE | FALSE | TRUE |
| **2309** | Q9Y371 | Endophilin-B1 | | unknown | -18.30 | 2.54 | FALSE | FALSE | TRUE |
| **2310** | Q13363 | C-terminal-binding protein 1 | | unknown | 12.34 | 6.74 | FALSE | FALSE | TRUE |
| **2311** | Q15014 | Mortality factor 4-like protein 2 | | unknown | -14.19 | -20.12 | FALSE | FALSE | TRUE |
| **2312** | Q9H792 | Pseudopodium-enriched atypical ki | | unknown | -4.10 | 1.05 | TRUE | FALSE | FALSE |
| **2313** | Q8NDA8 | Maestro heat-like repeat-containin | | unknown | -6.10 | 7.18 | FALSE | FALSE | TRUE |
| **2314** | Q14108 | Lysosome membrane protein 2 | | LYSOSOME | -40.55 | 9.97 | FALSE | FALSE | TRUE |

| | | | | | | | | |
|---|---|---|---|---|---|---|---|---|
| **2315** | Q9Y237 | Peptidyl-prolyl cis-trans isomerase I | unknown | -6.36 | -18.98 | FALSE | FALSE | TRUE |
| **2316** | Q00653 | Nuclear factor NF-kappa-B p100 sul | unknown | 1.34 | -2.33 | FALSE | FALSE | TRUE |
| **2317** | P0C7U0 | Protein ELFN1 | PM | -24.96 | 7.27 | TRUE | FALSE | FALSE |
| **2318** | P48634 | Protein PRRC2A | unknown | 6.05 | -18.69 | TRUE | FALSE | TRUE |
| **2319** | Q96I25 | Splicing factor 45 | NUCLEUS | 4.76 | -34.00 | TRUE | FALSE | TRUE |
| **2320** | Q8NBF2 | NHL repeat-containing protein 2 | unknown | 19.15 | 21.62 | FALSE | FALSE | TRUE |
| **2321** | Q9NVS2 | 39S ribosomal protein S18a, mitoch | MITOCHONDRIA | 43.29 | -5.35 | FALSE | FALSE | TRUE |
| **2322** | O60658 | High affinity cAMP-specific and IBN | unknown | -27.29 | 2.17 | TRUE | FALSE | FALSE |
| **2323** | P62070 | Ras-related protein R-Ras2 | PM | -25.89 | 15.72 | FALSE | FALSE | TRUE |
| **2324** | Q8N183 | NADH dehydrogenase [ubiquinone] | MITOCHONDRIA | 42.63 | -9.75 | TRUE | FALSE | TRUE |
| **2325** | Q13573 | SNW domain-containing protein 1 | NUCLEUS | -2.43 | -38.19 | TRUE | FALSE | TRUE |
| **2326** | Q14554 | Protein disulfide-isomerase A5 | ER | 4.84 | 37.70 | FALSE | FALSE | TRUE |
| **2327** | P19447 | General transcription and DNA repa | unknown | -9.42 | -22.88 | FALSE | FALSE | TRUE |
| **2328** | Q8TAE8 | Growth arrest and DNA damage-inc | unknown | 41.51 | -1.42 | FALSE | FALSE | TRUE |
| **2329** | Q6NYC8 | Phostensin | unknown | -5.24 | 10.91 | TRUE | FALSE | FALSE |
| **2330** | Q8IWC1 | MAP7 domain-containing protein 3 | unknown | -5.82 | -8.98 | TRUE | FALSE | TRUE |
| **2331** | P09884 | DNA polymerase alpha catalytic sul | unknown | 16.26 | 3.21 | FALSE | FALSE | TRUE |
| **2332** | O94913 | Pre-mRNA cleavage complex 2 prot | NUCLEUS | -13.85 | -22.86 | TRUE | FALSE | TRUE |
| **2333** | Q9BW19 | Kinesin-like protein KIFC1 | unknown | -15.98 | -12.32 | FALSE | FALSE | TRUE |
| **2334** | Q9NUQ6 | SPATS2-like protein | unknown | 9.90 | -19.06 | FALSE | FALSE | TRUE |
| **2335** | Q15652 | Probable JmjC domain-containing h | unknown | -14.77 | -19.24 | TRUE | FALSE | FALSE |
| **2336** | Q8N3V7 | Synaptopodin | unknown | -6.98 | -4.47 | TRUE | FALSE | TRUE |
| **2337** | P18859 | ATP synthase-coupling factor 6, mit | MITOCHONDRIA | 39.26 | -11.93 | FALSE | FALSE | TRUE |
| **2338** | Q9Y276 | Mitochondrial chaperone BCS1 | MITOCHONDRIA | 41.27 | -13.54 | FALSE | FALSE | TRUE |
| **2339** | Q04656 | Copper-transporting ATPase 1 | unknown | -35.29 | 14.84 | TRUE | FALSE | FALSE |
| **2340** | Q9UKS6 | Protein kinase C and casein kinase : | PM | -24.42 | 8.11 | TRUE | FALSE | TRUE |
| **2341** | Q9UHR4 | Brain-specific angiogenesis inhibitor | PM | -24.74 | 14.65 | TRUE | FALSE | TRUE |
| **2342** | Q9NRA8 | Eukaryotic translation initiation fact | unknown | 1.66 | -18.87 | TRUE | FALSE | TRUE |
| **2343** | Q8IYS1 | Peptidase M20 domain-containing | unknown | 16.35 | 18.37 | FALSE | FALSE | TRUE |
| **2344** | P04062 | Glucosylceramidase | LYSOSOME | -40.62 | 8.83 | FALSE | FALSE | TRUE |
| **2345** | Q5VT25 | Serine/threonine-protein kinase MI | unknown | -7.79 | 7.68 | TRUE | FALSE | FALSE |
| **2346** | Q8N9T8 | Protein KRI1 homolog | NUCLEUS | -8.54 | -33.18 | TRUE | FALSE | TRUE |
| **2347** | Q9NUM4 | Transmembrane protein 106B | unknown | -40.35 | 8.96 | FALSE | FALSE | TRUE |
| **2348** | Q9Y6E0 | Serine/threonine-protein kinase 24 | unknown | 1.35 | 10.79 | FALSE | FALSE | TRUE |
| **2349** | Q14254 | Flotillin-2 | unknown | -39.75 | 9.72 | TRUE | FALSE | TRUE |
| **2350** | Q9ULT8 | E3 ubiquitin-protein ligase HECTD1 | unknown | 13.59 | 9.31 | FALSE | FALSE | TRUE |
| **2351** | Q8IXB1 | DnaJ homolog subfamily C member | ER | 2.55 | 37.03 | FALSE | FALSE | TRUE |
| **2352** | Q6ZU35 | Uncharacterized protein KIAA1211 | unknown | -4.14 | 9.88 | TRUE | FALSE | FALSE |

| | | | | | | | | |
|---|---|---|---|---|---|---|---|---|
| **2353** | Q96RT7 | Gamma-tubulin complex componer | unknown | 6.95 | -24.88 | FALSE | FALSE | TRUE |
| **2354** | O95372 | Acyl-protein thioesterase 2 | unknown | 10.50 | 11.57 | TRUE | FALSE | FALSE |
| **2355** | Q9H6R0 | Putative ATP-dependent RNA helic | NUCLEUS | -0.86 | -38.61 | FALSE | TRUE | TRUE |
| **2356** | Q8WUA2 | Peptidyl-prolyl cis-trans isomerase- | NUCLEUS | -0.63 | -24.87 | FALSE | FALSE | TRUE |
| **2357** | P46379 | Large proline-rich protein BAG6 | unknown | 10.88 | 2.93 | FALSE | FALSE | TRUE |
| **2358** | Q99417 | c-Myc-binding protein | NUCLEUS | 8.37 | -4.82 | FALSE | FALSE | TRUE |
| **2359** | Q9BQ61 | Telomerase RNA component intera | NUCLEUS | -1.29 | -32.99 | TRUE | FALSE | TRUE |
| **2360** | Q13464 | Rho-associated protein kinase 1 | unknown | -8.35 | 10.94 | FALSE | FALSE | TRUE |
| **2361** | Q9NZJ0 | Denticleless protein homolog | unknown | -0.68 | -2.38 | FALSE | FALSE | TRUE |
| **2362** | Q9UNY4 | Transcription termination factor 2 | unknown | -11.26 | -16.98 | TRUE | FALSE | TRUE |
| **2363** | Q13643 | Four and a half LIM domains protei | unknown | 3.27 | -2.30 | FALSE | FALSE | TRUE |
| **2364** | Q9UPQ0 | LIM and calponin homology domai | unknown | 5.51 | 2.28 | TRUE | FALSE | TRUE |
| **2365** | Q15061 | WD repeat-containing protein 43 | NUCLEUS | -2.40 | -41.21 | FALSE | FALSE | TRUE |
| **2366** | Q8WUQ7 | Cactin | NUCLEUS | -2.55 | -36.00 | FALSE | FALSE | TRUE |
| **2367** | O60271 | C-Jun-amino-terminal kinase-intera | CYTOSOL | 16.30 | 17.07 | FALSE | FALSE | TRUE |
| **2368** | P18615 | Negative elongation factor E | NUCLEUS | 10.08 | -5.66 | TRUE | FALSE | TRUE |
| **2369** | Q9H330 | Transmembrane protein 245 | unknown | -11.45 | 34.22 | FALSE | FALSE | TRUE |
| **2370** | Q9BY77 | Polymerase delta-interacting protei | NUCLEUS | -1.45 | -39.96 | TRUE | FALSE | TRUE |
| **2371** | Q7Z333 | Probable helicase senataxin | unknown | -14.83 | -17.23 | TRUE | FALSE | TRUE |
| **2372** | P35269 | General transcription factor IIF subu | NUCLEUS | -9.16 | -15.39 | FALSE | FALSE | TRUE |
| **2373** | Q14457 | Beclin-1 | unknown | -11.66 | 3.24 | TRUE | FALSE | FALSE |
| **2374** | Q8TEP8 | Centrosomal protein of 192 kDa | NUCLEUS | -2.55 | -4.33 | TRUE | FALSE | FALSE |
| **2375** | P49750 | YLP motif-containing protein 1 | NUCLEUS | 1.79 | -34.11 | TRUE | FALSE | TRUE |
| **2376** | Q8IUD2 | ELKS/Rab6-interacting/CAST family | NUCLEUS | -3.54 | -10.33 | TRUE | FALSE | FALSE |
| **2377** | P49247 | Ribose-5-phosphate isomerase | unknown | 4.84 | 1.43 | FALSE | FALSE | TRUE |
| **2378** | O60282 | Kinesin heavy chain isoform 5C | unknown | 0.76 | -4.52 | FALSE | FALSE | TRUE |
| **2379** | Q9H425 | Uncharacterized protein C1orf198 | unknown | -5.06 | 11.93 | TRUE | FALSE | FALSE |
| **2380** | Q3LXA3 | Triokinase/FMN cyclase | unknown | 17.64 | 17.31 | FALSE | FALSE | TRUE |
| **2381** | Q9NVX2 | Notchless protein homolog 1 | NUCLEUS | 0.39 | -24.25 | FALSE | FALSE | TRUE |
| **2382** | Q8IWX8 | Calcium homeostasis endoplasmic r | NUCLEUS | 4.77 | -33.73 | TRUE | FALSE | TRUE |
| **2383** | Q96JH7 | Deubiquitinating protein VCIP135 | unknown | -0.04 | -3.63 | TRUE | FALSE | TRUE |
| **2384** | Q8IVM0 | Coiled-coil domain-containing prote | unknown | -4.15 | 11.98 | TRUE | FALSE | TRUE |
| **2385** | Q9NYZ3 | G2 and S phase-expressed protein 1 | unknown | -7.17 | -11.67 | TRUE | FALSE | FALSE |
| **2386** | P85037 | Forkhead box protein K1 | NUCLEUS | -9.27 | -13.95 | TRUE | FALSE | FALSE |
| **2387** | Q9H0X4 | Protein FAM234A | PM | -21.30 | 15.74 | TRUE | FALSE | TRUE |
| **2388** | Q12923 | Tyrosine-protein phosphatase non- | unknown | -7.19 | 10.73 | TRUE | FALSE | TRUE |
| **2389** | O15397 | Importin-8 | unknown | 16.30 | 7.16 | FALSE | FALSE | TRUE |
| **2390** | P49848 | Transcription initiation factor TFIID | unknown | -15.35 | -21.72 | TRUE | FALSE | FALSE |

| | | | | | | | | |
|---|---|---|---|---|---|---|---|---|
| **2391** | P49959 | Double-strand break repair protein | unknown | -16.34 | -17.43 | TRUE | FALSE | TRUE |
| **2392** | P19387 | DNA-directed RNA polymerase II su | NUCLEUS | -15.37 | -29.07 | FALSE | FALSE | TRUE |
| **2393** | Q15075 | Early endosome antigen 1 | unknown | 7.74 | 0.04 | FALSE | FALSE | TRUE |
| **2394** | P10619 | Lysosomal protective protein | LYSOSOME | -35.83 | 3.86 | FALSE | FALSE | TRUE |
| **2395** | Q9NQZ2 | Something about silencing protein 1 | NUCLEUS | -1.01 | -40.29 | FALSE | FALSE | TRUE |
| **2396** | P08579 | U2 small nuclear ribonucleoprotein | NUCLEUS | -5.98 | -33.71 | TRUE | FALSE | FALSE |
| **2397** | Q9NUQ3 | Gamma-taxilin | unknown | -7.79 | -9.27 | TRUE | FALSE | TRUE |
| **2398** | P30260 | Cell division cycle protein 27 homol | unknown | -17.11 | -19.25 | FALSE | FALSE | TRUE |
| **2399** | A8MVW0 | Protein FAM171A2 | unknown | -30.11 | -0.63 | TRUE | FALSE | FALSE |
| **2400** | O60830 | Mitochondrial import inner membra | MITOCHONDRIA | 41.47 | -13.26 | FALSE | FALSE | TRUE |
| **2401** | P61916 | NPC intracellular cholesterol transp | unknown | -40.82 | 10.58 | FALSE | FALSE | TRUE |
| **2402** | P62875 | DNA-directed RNA polymerases I, II | NUCLEUS | -16.57 | -28.96 | FALSE | FALSE | TRUE |
| **2403** | Q9BVI4 | Nucleolar complex protein 4 homol | NUCLEUS | -2.49 | -40.59 | FALSE | FALSE | TRUE |
| **2404** | Q13451 | Peptidyl-prolyl cis-trans isomerase | unknown | 21.01 | 16.34 | FALSE | FALSE | TRUE |
| **2405** | Q9H4G0 | Band 4.1-like protein 1 | PM | -27.38 | 1.07 | TRUE | FALSE | TRUE |
| **2406** | Q9UGP4 | LIM domain-containing protein 1 | unknown | -3.57 | 11.61 | TRUE | FALSE | TRUE |
| **2407** | Q6RW13 | Type-1 angiotensin II receptor-asso | unknown | -36.11 | 10.30 | FALSE | FALSE | TRUE |
| **2408** | Q96AQ6 | Pre-B-cell leukemia transcription fa | unknown | -10.33 | 30.53 | TRUE | FALSE | FALSE |
| **2409** | O95817 | BAG family molecular chaperone re | unknown | -2.35 | -6.67 | TRUE | FALSE | TRUE |
| **2410** | O14964 | Hepatocyte growth factor-regulate | PM | -23.11 | 13.92 | TRUE | FALSE | FALSE |
| **2411** | Q9BQ75 | Protein CMSS1 | NUCLEUS | 3.61 | -27.10 | TRUE | FALSE | TRUE |
| **2412** | Q9NPQ8 | Synembryn-A | unknown | 10.43 | 12.17 | TRUE | FALSE | FALSE |
| **2413** | Q9NRZ9 | Lymphoid-specific helicase | unknown | -14.75 | -20.69 | FALSE | FALSE | TRUE |
| **2414** | P49023 | Paxillin | unknown | 7.76 | 18.15 | TRUE | FALSE | TRUE |
| **2415** | P53794 | Sodium/myo-inositol cotransporter | PM | -21.22 | 16.64 | TRUE | FALSE | FALSE |
| **2416** | O14562 | Ubiquitin domain-containing protein | unknown | 15.75 | 4.48 | FALSE | FALSE | TRUE |
| **2417** | Q92925 | SWI/SNF-related matrix-associated | NUCLEUS | -25.53 | -23.92 | FALSE | FALSE | TRUE |
| **2418** | Q9UID3 | Vacuolar protein sorting-associated | unknown | -8.37 | -7.86 | TRUE | FALSE | TRUE |
| **2419** | P52298 | Nuclear cap-binding protein subunit | NUCLEUS | -12.50 | -29.67 | FALSE | FALSE | TRUE |
| **2420** | P01137 | Transforming growth factor beta-1 | ER | -3.95 | 34.99 | FALSE | FALSE | TRUE |
| **2421** | Q52LW3 | Rho GTPase-activating protein 29 | unknown | -2.59 | 0.48 | TRUE | FALSE | FALSE |
| **2422** | O94804 | Serine/threonine-protein kinase 10 | unknown | -13.47 | 13.24 | TRUE | FALSE | TRUE |
| **2423** | Q9NS86 | LanC-like protein 2 | unknown | 9.04 | -3.87 | FALSE | FALSE | TRUE |
| **2424** | Q9UPT8 | Zinc finger CCCH domain-containing | NUCLEUS | -18.37 | -32.81 | TRUE | FALSE | TRUE |
| **2425** | P48681 | Nestin | NUCLEUS | -19.98 | -32.30 | TRUE | FALSE | TRUE |
| **2426** | Q8ND04 | Protein SMG8 | unknown | 5.58 | -20.98 | FALSE | FALSE | TRUE |
| **2427** | Q12996 | Cleavage stimulation factor subunit | NUCLEUS | -10.43 | -35.14 | FALSE | FALSE | TRUE |
| **2428** | Q96B97 | SH3 domain-containing kinase-bind | NUCLEUS | -10.46 | -13.81 | FALSE | FALSE | TRUE |

| | | | | | | | | |
|---|---|---|---|---|---|---|---|---|
| **2429** | P01033 | Metalloproteinase inhibitor 1 | PM | -28.33 | 13.94 | FALSE | FALSE | TRUE |
| **2430** | Q9GZZ9 | Ubiquitin-like modifier-activating er | unknown | 10.90 | 18.35 | FALSE | FALSE | TRUE |
| **2431** | O75348 | V-type proton ATPase subunit G 1 | unknown | -17.83 | 3.98 | FALSE | FALSE | TRUE |
| **2432** | Q13459 | Unconventional myosin-IXb | unknown | -2.93 | -1.36 | TRUE | FALSE | TRUE |
| **2433** | Q96SY0 | Integrator complex subunit 14 | unknown | -17.22 | -21.98 | FALSE | FALSE | TRUE |
| **2434** | Q8N3U4 | Cohesin subunit SA-2 | NUCLEUS-CHROMATIN | -18.20 | -16.06 | TRUE | FALSE | FALSE |
| **2435** | Q96IJ6 | Mannose-1-phosphate guanyltrans | unknown | 15.51 | 9.15 | FALSE | FALSE | TRUE |
| **2436** | Q14165 | Malectin | unknown | 5.36 | 36.80 | FALSE | FALSE | TRUE |
| **2437** | Q9H4I3 | TraB domain-containing protein | MITOCHONDRIA | 37.88 | -7.54 | FALSE | FALSE | TRUE |
| **2438** | Q13505 | Metaxin-1 | MITOCHONDRIA | 38.89 | -0.19 | FALSE | FALSE | TRUE |
| **2439** | O15511 | Actin-related protein 2/3 complex s | unknown | 1.72 | -14.98 | FALSE | FALSE | TRUE |
| **2440** | Q93052 | Lipoma-preferred partner | unknown | -2.76 | 0.24 | TRUE | FALSE | TRUE |
| **2441** | P55081 | Microfibrillar-associated protein 1 | NUCLEUS | 0.64 | -40.66 | TRUE | FALSE | TRUE |
| **2442** | Q96IZ0 | PRKC apoptosis WT1 regulator prot | unknown | -2.54 | -0.31 | FALSE | FALSE | TRUE |
| **2443** | Q9H814 | Phosphorylated adapter RNA expor | unknown | 23.19 | 15.25 | FALSE | FALSE | TRUE |
| **2444** | Q9UKJ3 | G patch domain-containing protein | NUCLEUS | -6.60 | -30.09 | TRUE | FALSE | TRUE |
| **2445** | P29992 | Guanine nucleotide-binding protein | PM | -27.33 | 10.22 | FALSE | FALSE | TRUE |
| **2446** | Q5VT52 | Regulation of nuclear pre-mRNA do | NUCLEUS | -18.65 | -26.28 | TRUE | FALSE | TRUE |
| **2447** | Q8NFH4 | Nucleoporin Nup37 | NUCLEUS | -19.75 | -29.81 | FALSE | FALSE | TRUE |
| **2448** | Q9BY89 | Uncharacterized protein KIAA1671 | unknown | -3.88 | 2.60 | TRUE | FALSE | TRUE |
| **2449** | Q92600 | CCR4-NOT transcription complex su | unknown | 8.63 | -25.42 | FALSE | FALSE | TRUE |
| **2450** | Q15678 | Tyrosine-protein phosphatase non- | unknown | -7.12 | -0.06 | FALSE | FALSE | TRUE |
| **2451** | Q86UU1 | Pleckstrin homology-like domain fa | unknown | 0.36 | -9.21 | TRUE | FALSE | FALSE |
| **2452** | Q9UPN4 | Centrosomal protein of 131 kDa | NUCLEUS | -1.36 | -26.83 | TRUE | FALSE | TRUE |
| **2453** | Q8NG31 | Kinetochore scaffold 1 | unknown | -12.39 | -17.63 | TRUE | FALSE | TRUE |
| **2454** | Q14BN4 | Sarcolemmal membrane-associate | unknown | -12.76 | 29.09 | FALSE | FALSE | TRUE |
| **2455** | O43823 | A-kinase anchor protein 8 | NUCLEUS | -14.75 | -30.53 | FALSE | FALSE | TRUE |
| **2456** | Q9Y672 | Dolichyl pyrophosphate Man9GlcN/ | unknown | 6.48 | 37.18 | FALSE | FALSE | TRUE |
| **2457** | O60716 | Catenin delta-1 | PM | -26.29 | 2.14 | TRUE | FALSE | TRUE |
| **2458** | P41743 | Protein kinase C iota type | unknown | -8.37 | 14.31 | FALSE | FALSE | TRUE |
| **2459** | Q13445 | Transmembrane emp24 domain-cc | unknown | -10.43 | 32.54 | FALSE | FALSE | TRUE |
| **2460** | P10644 | cAMP-dependent protein kinase ty | unknown | 7.45 | 13.85 | FALSE | FALSE | TRUE |
| **2461** | P41240 | Tyrosine-protein kinase CSK | unknown | 0.00 | -4.35 | FALSE | FALSE | TRUE |
| **2462** | Q8NFJ5 | Retinoic acid-induced protein 3 | PM | -20.53 | 15.11 | TRUE | FALSE | TRUE |
| **2463** | Q9UKZ1 | CCR4-NOT transcription complex su | RIBOSOME 40S | 9.09 | -24.63 | TRUE | FALSE | TRUE |
| **2464** | Q8IWE4 | DCN1-like protein 3 | PM | -26.83 | 11.53 | TRUE | FALSE | FALSE |
| **2465** | Q9NSV4 | Protein diaphanous homolog 3 | unknown | -4.19 | -4.94 | TRUE | FALSE | TRUE |
| **2466** | O95197 | Reticulon-3 | unknown | -6.80 | 21.76 | FALSE | FALSE | TRUE |

| | | | | | | | | |
|---|---|---|---|---|---|---|---|---|
| **2467** | Q5JTD0 | Tight junction-associated protein 1 | unknown | -0.22 | -2.76 | TRUE | FALSE | FALSE |
| **2468** | Q9ULH1 | Arf-GAP with SH3 domain, ANK rep | unknown | -4.80 | 2.53 | TRUE | FALSE | FALSE |
| **2469** | Q96LD4 | Tripartite motif-containing protein 4 | unknown | 2.21 | -1.99 | FALSE | TRUE | TRUE |
| **2470** | Q8N4C8 | Misshapen-like kinase 1 | unknown | -5.99 | 13.02 | TRUE | FALSE | TRUE |
| **2471** | Q9Y2H6 | Fibronectin type-III domain-contair | ER | 2.69 | 36.43 | FALSE | FALSE | TRUE |
| **2472** | O43493 | Trans-Golgi network integral memb | unknown | -35.85 | 15.29 | FALSE | FALSE | TRUE |
| **2473** | P30042 | ES1 protein homolog, mitochondria | MITOCHONDRIA | 42.05 | -11.54 | FALSE | FALSE | TRUE |
| **2474** | Q8WXE0 | Caskin-2 | unknown | -5.68 | 12.85 | TRUE | FALSE | FALSE |
| **2475** | Q9H0E9 | Bromodomain-containing protein 8 | NUCLEUS-CHROMATIN | -19.44 | -16.51 | TRUE | FALSE | FALSE |
| **2476** | P19784 | Casein kinase II subunit alpha' | unknown | 12.27 | 3.75 | FALSE | FALSE | TRUE |
| **2477** | Q9BQE5 | Apolipoprotein L2 | unknown | -3.66 | 5.84 | FALSE | FALSE | TRUE |
| **2478** | P40855 | Peroxisomal biogenesis factor 19 | unknown | 10.63 | 8.37 | FALSE | FALSE | TRUE |
| **2479** | Q96S66 | Chloride channel CLIC-like protein 1 | unknown | -7.51 | 33.47 | TRUE | FALSE | TRUE |
| **2480** | O43772 | Mitochondrial carnitine/acylcarnitir | unknown | 43.77 | -13.80 | FALSE | FALSE | TRUE |
| **2481** | P82094 | TATA element modulatory factor | unknown | -20.80 | 32.55 | TRUE | FALSE | FALSE |
| **2482** | Q9UJ70 | N-acetyl-D-glucosamine kinase | unknown | 24.55 | 21.21 | FALSE | FALSE | TRUE |
| **2483** | P25490 | Transcriptional repressor protein YY | NUCLEUS | -25.43 | -26.18 | FALSE | FALSE | TRUE |
| **2484** | Q92538 | Golgi-specific brefeldin A-resistance | unknown | -16.72 | 7.02 | FALSE | FALSE | TRUE |
| **2485** | Q9UEE9 | Craniofacial development protein 1 | unknown | 22.60 | 14.67 | TRUE | FALSE | TRUE |
| **2486** | O60508 | Pre-mRNA-processing factor 17 | NUCLEUS | -2.56 | -37.75 | FALSE | FALSE | TRUE |
| **2487** | P62745 | Rho-related GTP-binding protein Rh | unknown | -28.40 | 10.59 | FALSE | FALSE | TRUE |
| **2488** | P30622 | CAP-Gly domain-containing linker p | unknown | 2.26 | -1.68 | FALSE | FALSE | TRUE |
| **2489** | Q8NBJ7 | Sulfatase-modifying factor 2 | ER | -5.04 | 26.72 | FALSE | FALSE | TRUE |
| **2490** | O43164 | E3 ubiquitin-protein ligase Praja-2 | unknown | 14.22 | 3.15 | TRUE | FALSE | FALSE |
| **2491** | Q9HB58 | Sp110 nuclear body protein | NUCLEUS-CHROMATIN | -20.19 | -13.27 | FALSE | FALSE | TRUE |
| **2492** | Q9BY43 | Charged multivesicular body protei | unknown | 10.50 | 14.85 | FALSE | FALSE | TRUE |
| **2493** | Q9Y4D1 | Disheveled-associated activator of | unknown | -24.12 | 5.71 | TRUE | FALSE | FALSE |
| **2494** | Q96FK6 | WD repeat-containing protein 89 | NUCLEUS | -2.88 | -30.37 | FALSE | FALSE | TRUE |
| **2495** | O60870 | DNA/RNA-binding protein KIN17 | NUCLEUS | 3.06 | -32.46 | FALSE | FALSE | TRUE |
| **2496** | Q13439 | Golgin subfamily A member 4 | unknown | -1.02 | -0.30 | TRUE | FALSE | FALSE |
| **2497** | P48556 | 26S proteasome non-ATPase regul | PROTEASOME | 21.49 | 6.64 | FALSE | FALSE | TRUE |
| **2498** | Q8NDI1 | EH domain-binding protein 1 | unknown | 2.82 | 9.20 | TRUE | FALSE | TRUE |
| **2499** | Q5JRX3 | Presequence protease, mitochondri | MITOCHONDRIA | 39.56 | -7.88 | FALSE | FALSE | TRUE |
| **2500** | O15126 | Secretory carrier-associated memb | unknown | -34.52 | 11.35 | TRUE | FALSE | TRUE |
| **2501** | P12955 | Xaa-Pro dipeptidase | unknown | 23.24 | 20.62 | TRUE | FALSE | FALSE |
| **2502** | Q9NXV6 | CDKN2A-interacting protein | NUCLEUS | -4.02 | -30.62 | TRUE | FALSE | TRUE |
| **2503** | Q9H6T3 | RNA polymerase II-associated prot | unknown | 8.52 | -14.12 | TRUE | FALSE | TRUE |
| **2504** | P82970 | High mobility group nucleosome-bi | NUCLEUS | -19.83 | -21.83 | FALSE | FALSE | TRUE |

| | | | | | | | | |
|---|---|---|---|---|---|---|---|---|
| **2505** | P28290 | Sperm-specific antigen 2 | unknown | -2.64 | 2.18 | TRUE | FALSE | TRUE |
| **2506** | Q9H4L7 | SWI/SNF-related matrix-associatec | unknown | 16.29 | 3.08 | FALSE | FALSE | TRUE |
| **2507** | Q9UH65 | Switch-associated protein 70 | unknown | 6.44 | 0.41 | TRUE | FALSE | TRUE |
| **2508** | P42345 | Serine/threonine-protein kinase m | unknown | -10.34 | 3.35 | FALSE | FALSE | TRUE |
| **2509** | Q8NFC6 | Biorientation of chromosomes in ce | NUCLEUS | -22.13 | -25.08 | TRUE | FALSE | TRUE |
| **2510** | P11047 | Laminin subunit gamma-1 | ER | -0.71 | 37.32 | FALSE | FALSE | TRUE |
| **2511** | Q969G3 | SWI/SNF-related matrix-associatec | NUCLEUS | -25.78 | -24.15 | TRUE | FALSE | TRUE |
| **2512** | Q8IWA4 | Mitofusin-1 | unknown | 34.29 | -3.50 | FALSE | FALSE | TRUE |
| **2513** | Q6PGP7 | Tetratricopeptide repeat protein 37 | unknown | 8.13 | -21.13 | FALSE | FALSE | TRUE |
| **2514** | Q86UU0 | B-cell CLL/lymphoma 9-like protein | unknown | 0.39 | -8.74 | TRUE | FALSE | FALSE |
| **2515** | Q9UN37 | Vacuolar protein sorting-associated | unknown | -17.66 | 6.42 | FALSE | FALSE | TRUE |
| **2516** | A8CG34 | Nuclear envelope pore membrane | unknown | 23.65 | -12.16 | TRUE | FALSE | TRUE |
| **2517** | Q9P273 | Teneurin-3 | PM | -26.01 | 8.05 | TRUE | FALSE | FALSE |
| **2518** | Q6XZF7 | Dynamin-binding protein | unknown | -0.78 | -1.96 | TRUE | FALSE | TRUE |
| **2519** | P46736 | Lys-63-specific deubiquitinase BRC | NUCLEUS | 12.94 | 1.43 | FALSE | FALSE | TRUE |
| **2520** | Q13136 | Liprin-alpha-1 | unknown | -4.03 | 2.48 | TRUE | FALSE | TRUE |
| **2521** | Q9H3P7 | Golgi resident protein GCP60 | unknown | 19.32 | 15.81 | FALSE | FALSE | TRUE |
| **2522** | A6NJ78 | Probable methyltransferase-like pr | MITOCHONDRIA | 33.51 | -8.68 | FALSE | FALSE | TRUE |
| **2523** | Q9BWU0 | Kanadaptin | unknown | -12.05 | -31.41 | TRUE | FALSE | TRUE |
| **2524** | P16930 | Fumarylacetoacetase | CYTOSOL | 19.02 | 24.44 | TRUE | FALSE | TRUE |
| **2525** | P40692 | DNA mismatch repair protein Mlh1 | unknown | -12.67 | -15.39 | FALSE | FALSE | TRUE |
| **2526** | Q9H5Z1 | Probable ATP-dependent RNA helic | NUCLEUS | -1.08 | -24.27 | FALSE | FALSE | TRUE |
| **2527** | Q96QC0 | Serine/threonine-protein phosphat | NUCLEUS | -12.27 | -35.49 | TRUE | FALSE | TRUE |
| **2528** | O43159 | Ribosomal RNA-processing protein | NUCLEUS | -0.95 | -38.78 | FALSE | FALSE | TRUE |
| **2529** | Q15435 | Protein phosphatase 1 regulatory su | unknown | 19.48 | 20.60 | FALSE | FALSE | TRUE |
| **2530** | Q7Z2W9 | 39S ribosomal protein L21, mitocho | unknown | 44.62 | -12.19 | FALSE | FALSE | TRUE |
| **2531** | Q8NBL1 | Protein O-glucosyltransferase 1 | ER | 2.33 | 36.51 | FALSE | FALSE | TRUE |
| **2532** | Q9NXR1 | Nuclear distribution protein nudE hc | unknown | -1.86 | -12.50 | FALSE | FALSE | TRUE |
| **2533** | P29083 | General transcription factor IIE subu | NUCLEUS | -7.94 | -15.57 | FALSE | FALSE | TRUE |
| **2534** | Q5JRA6 | Transport and Golgi organization pr | ER | 0.70 | 39.32 | TRUE | FALSE | FALSE |
| **2535** | Q9Y3B7 | 39S ribosomal protein L11, mitocho | MITOCHONDRIA | 42.88 | -4.68 | FALSE | FALSE | TRUE |
| **2536** | P35611 | Alpha-adducin | NUCLEUS | -10.87 | -17.73 | TRUE | FALSE | TRUE |
| **2537** | Q8NEB9 | Phosphatidylinositol 3-kinase cataly | unknown | 4.53 | -12.23 | TRUE | FALSE | TRUE |
| **2538** | Q9Y6A5 | Transforming acidic coiled-coil-cont | unknown | 12.38 | 8.94 | FALSE | FALSE | TRUE |
| **2539** | Q9Y679 | Ancient ubiquitous protein 1 | unknown | -4.41 | 31.20 | FALSE | FALSE | TRUE |
| **2540** | Q5JTJ3 | Cytochrome c oxidase assembly fac | unknown | 34.93 | 0.87 | FALSE | TRUE | TRUE |
| **2541** | Q9NWU1 | 3-oxoacyl-[acyl-carrier-protein] syn | MITOCHONDRIA | 41.89 | -8.71 | FALSE | FALSE | TRUE |
| **2542** | Q6NUQ4 | Transmembrane protein 214 | unknown | 5.62 | 38.04 | FALSE | FALSE | TRUE |

| | | | | | | | | |
|---|---|---|---|---|---|---|---|---|
| **2543** | P52306 | Rap1 GTPase-GDP dissociation stim | unknown | 20.17 | 21.78 | FALSE | FALSE | TRUE |
| **2544** | Q9UPQ9 | Trinucleotide repeat-containing ger | unknown | 8.81 | -15.39 | TRUE | FALSE | TRUE |
| **2545** | P57740 | Nuclear pore complex protein Nup1 | NUCLEUS | -20.91 | -29.69 | FALSE | FALSE | TRUE |
| **2546** | Q86XZ4 | Spermatogenesis-associated serine | unknown | 8.30 | -21.97 | FALSE | FALSE | TRUE |
| **2547** | Q13472 | DNA topoisomerase 3-alpha | unknown | -14.50 | -13.17 | FALSE | FALSE | TRUE |
| **2548** | O15400 | Syntaxin-7 | unknown | -36.46 | 7.30 | FALSE | FALSE | TRUE |
| **2549** | Q9H1C4 | Protein unc-93 homolog B1 | PM | -28.29 | 13.58 | FALSE | FALSE | TRUE |
| **2550** | O43824 | Putative GTP-binding protein 6 | MITOCHONDRIA | 29.80 | -12.41 | FALSE | FALSE | TRUE |
| **2551** | P57737 | Coronin-7 | unknown | 30.58 | -16.37 | FALSE | FALSE | TRUE |
| **2552** | Q9UNH7 | Sorting nexin-6 | unknown | -10.77 | 16.41 | FALSE | FALSE | TRUE |
| **2553** | P08069 | Insulin-like growth factor 1 receptor | PM | -22.71 | 17.15 | TRUE | FALSE | TRUE |
| **2554** | Q96GD0 | Pyridoxal phosphate phosphatase | unknown | 14.69 | 9.28 | FALSE | FALSE | TRUE |
| **2555** | Q6Y7W6 | GRB10-interacting GYF protein 2 | unknown | 8.22 | -25.36 | TRUE | FALSE | TRUE |
| **2556** | P06400 | Retinoblastoma-associated protein | unknown | -14.40 | -15.78 | FALSE | FALSE | TRUE |
| **2557** | O94842 | TOX high mobility group box family | NUCLEUS | -20.14 | -27.96 | TRUE | FALSE | TRUE |
| **2558** | Q96EV2 | RNA-binding protein 33 | NUCLEUS | 1.88 | -32.03 | FALSE | FALSE | TRUE |
| **2559** | Q8TAF3 | WD repeat-containing protein 48 | unknown | -8.79 | 4.80 | FALSE | FALSE | TRUE |
| **2560** | Q9BVV7 | Mitochondrial import inner membra | MITOCHONDRIA | 29.94 | -14.37 | FALSE | FALSE | TRUE |
| **2561** | Q12830 | Nucleosome-remodeling factor sub | NUCLEUS | -24.24 | -23.44 | TRUE | FALSE | TRUE |
| **2562** | O95218 | Zinc finger Ran-binding domain-con | unknown | 21.29 | 11.46 | TRUE | FALSE | TRUE |
| **2563** | P46734 | Dual specificity mitogen-activated p | unknown | 0.24 | -4.33 | FALSE | FALSE | TRUE |
| **2564** | Q15059 | Bromodomain-containing protein 3 | NUCLEUS-CHROMATIN | -25.85 | -23.30 | FALSE | FALSE | TRUE |
| **2565** | O75781 | Paralemmin-1 | PM | -24.48 | 4.68 | FALSE | FALSE | TRUE |
| **2566** | P07942 | Laminin subunit beta-1 | ER | -0.75 | 37.38 | FALSE | FALSE | TRUE |
| **2567** | Q15006 | ER membrane protein complex subu | ER | 0.51 | 35.47 | FALSE | FALSE | TRUE |
| **2568** | Q12834 | Cell division cycle protein 20 homol | unknown | -4.91 | -13.45 | FALSE | FALSE | TRUE |
| **2569** | Q9H3P2 | Negative elongation factor A | unknown | 13.97 | 2.43 | TRUE | FALSE | TRUE |
| **2570** | Q9C0F1 | Centrosomal protein of 44 kDa | unknown | -11.27 | -1.46 | FALSE | FALSE | TRUE |
| **2571** | Q5T5U3 | Rho GTPase-activating protein 21 | unknown | -3.50 | -0.61 | TRUE | FALSE | TRUE |
| **2572** | Q8NFH5 | Nucleoporin NUP35 | unknown | 23.39 | -12.29 | FALSE | FALSE | TRUE |
| **2573** | Q96T58 | Msx2-interacting protein | unknown | -17.84 | -19.00 | TRUE | FALSE | TRUE |
| **2574** | Q2KHR3 | Glutamine and serine-rich protein 1 | NUCLEUS-CHROMATIN | -19.97 | -15.07 | TRUE | FALSE | TRUE |
| **2575** | O43819 | Protein SCO2 homolog, mitochondr | MITOCHONDRIA | 35.11 | -6.95 | FALSE | FALSE | TRUE |
| **2576** | Q9NPL8 | Complex I assembly factor TIMMD | MITOCHONDRIA | 43.41 | -13.44 | FALSE | FALSE | TRUE |
| **2577** | Q15036 | Sorting nexin-17 | unknown | -10.80 | 9.68 | FALSE | FALSE | TRUE |
| **2578** | Q13111 | Chromatin assembly factor 1 subun | NUCLEUS | -24.43 | -22.33 | TRUE | FALSE | TRUE |
| **2579** | Q8IWR0 | Zinc finger CCCH domain-containing | unknown | 8.43 | -25.64 | FALSE | FALSE | TRUE |
| **2580** | O00461 | Golgi integral membrane protein 4 | GOLGI | -20.05 | 32.12 | FALSE | FALSE | TRUE |

| | | | | | | | | |
|---|---|---|---|---|---|---|---|---|
| **2581** | Q9H4K7 | Mitochondrial ribosome-associated | MITOCHONDRIA | 35.40 | -14.24 | FALSE | FALSE | TRUE |
| **2582** | Q9UK59 | Lariat debranching enzyme | unknown | 11.20 | -6.54 | FALSE | FALSE | TRUE |
| **2583** | Q96KQ7 | Histone-lysine N-methyltransferase | unknown | -23.43 | -18.78 | FALSE | FALSE | TRUE |
| **2584** | Q96LJ7 | Dehydrogenase/reductase SDR fan | unknown | -1.34 | 26.05 | FALSE | FALSE | TRUE |
| **2585** | Q5C9Z4 | Nucleolar MIF4G domain-containin | NUCLEUS | -3.46 | -34.27 | FALSE | FALSE | TRUE |
| **2586** | Q9BVC5 | Ashwin | NUCLEUS | 0.43 | -36.92 | FALSE | FALSE | TRUE |
| **2587** | Q70CQ2 | Ubiquitin carboxyl-terminal hydrola | unknown | 16.25 | 7.82 | FALSE | FALSE | TRUE |
| **2588** | O95865 | N(G),N(G)-dimethylarginine dimeth | unknown | 8.82 | 14.28 | FALSE | FALSE | TRUE |
| **2589** | Q7Z5L9 | Interferon regulatory factor 2-bindi | NUCLEUS | -13.08 | -19.92 | TRUE | FALSE | TRUE |
| **2590** | Q9NRR4 | Ribonuclease 3 | NUCLEUS | 0.84 | -25.09 | FALSE | FALSE | TRUE |
| **2591** | Q13206 | Probable ATP-dependent RNA helic | NUCLEUS | -2.77 | -37.15 | FALSE | FALSE | TRUE |
| **2592** | Q9ULR0 | Pre-mRNA-splicing factor ISY1 hom | NUCLEUS | -3.56 | -39.05 | FALSE | FALSE | TRUE |
| **2593** | Q9BRK4 | Leucine zipper putative tumor supp | unknown | -15.02 | 13.08 | TRUE | FALSE | FALSE |
| **2594** | O00257 | E3 SUMO-protein ligase CBX4 | unknown | -4.58 | -22.60 | FALSE | FALSE | TRUE |
| **2595** | Q4KMP7 | TBC1 domain family member 10B | unknown | -15.12 | 13.54 | TRUE | FALSE | FALSE |
| **2596** | P02765 | Alpha-2-HS-glycoprotein | unknown | -19.25 | 4.56 | FALSE | FALSE | TRUE |
| **2597** | P48509 | CD151 antigen | unknown | -21.04 | 21.11 | FALSE | FALSE | TRUE |
| **2598** | Q9BVS5 | tRNA (adenine(58)-N(1))-methyltra | unknown | 44.28 | -12.75 | FALSE | FALSE | TRUE |
| **2599** | P08572 | Collagen alpha-2(IV) chain | unknown | 6.07 | 32.14 | FALSE | FALSE | TRUE |
| **2600** | Q6YHK3 | CD109 antigen | PM | -22.20 | 14.85 | FALSE | FALSE | TRUE |
| **2601** | Q12802 | A-kinase anchor protein 13 | unknown | -3.47 | -5.58 | TRUE | FALSE | TRUE |
| **2602** | Q9UQN3 | Charged multivesicular body protei | unknown | -18.48 | 6.60 | TRUE | FALSE | TRUE |
| **2603** | Q6WCQ1 | Myosin phosphatase Rho-interactir | unknown | -1.74 | 4.00 | FALSE | FALSE | TRUE |
| **2604** | Q9UBI6 | Guanine nucleotide-binding protein | unknown | -27.40 | 6.21 | FALSE | FALSE | TRUE |
| **2605** | O75330 | Hyaluronan mediated motility rece | unknown | -3.92 | -11.30 | FALSE | FALSE | TRUE |
| **2606** | Q69YH5 | Cell division cycle-associated protei | NUCLEUS | -23.76 | -24.24 | TRUE | FALSE | TRUE |
| **2607** | Q03518 | Antigen peptide transporter 1 | unknown | -5.59 | 23.30 | FALSE | FALSE | TRUE |
| **2608** | Q969P0 | Immunoglobulin superfamily meml | PM | -27.34 | 6.19 | FALSE | FALSE | TRUE |
| **2609** | P46976 | Glycogenin-1 | unknown | 12.25 | -9.33 | FALSE | FALSE | TRUE |
| **2610** | Q9UI12 | V-type proton ATPase subunit H | unknown | -17.54 | 3.89 | FALSE | FALSE | TRUE |
| **2611** | P78316 | Nucleolar protein 14 | NUCLEUS | -0.59 | -41.28 | TRUE | FALSE | TRUE |
| **2612** | Q12888 | TP53-binding protein 1 | NUCLEUS | -20.52 | -32.54 | FALSE | FALSE | TRUE |
| **2613** | Q9NVH2 | Integrator complex subunit 7 | NUCLEUS | -18.09 | -23.66 | FALSE | FALSE | TRUE |
| **2614** | Q14676 | Mediator of DNA damage checkpoi | unknown | -22.84 | -15.72 | TRUE | FALSE | TRUE |
| **2615** | Q9NVU7 | Protein SDA1 homolog | NUCLEUS | 3.57 | -27.75 | FALSE | FALSE | TRUE |
| **2616** | P31751 | RAC-beta serine/threonine-protein | unknown | 9.73 | 5.69 | FALSE | FALSE | TRUE |
| **2617** | O43678 | NADH dehydrogenase [ubiquinone] | MITOCHONDRIA | 31.13 | -12.30 | FALSE | FALSE | TRUE |
| **2618** | Q8TF74 | WAS/WASL-interacting protein fan | unknown | 1.65 | -9.93 | TRUE | FALSE | TRUE |

| | | | | | | | | |
|---|---|---|---|---|---|---|---|---|
| **2619** | Q6ZUT6 | Coiled-coil domain-containing prote | NUCLEUS | -1.63 | -27.40 | FALSE | FALSE | TRUE |
| **2620** | Q96ST3 | Paired amphipathic helix protein Sir | NUCLEUS | -25.41 | -24.88 | FALSE | FALSE | TRUE |
| **2621** | Q9Y5J9 | Mitochondrial import inner membra | MITOCHONDRIA | 32.35 | -14.03 | FALSE | FALSE | TRUE |
| **2622** | Q6ULP2 | Aftiphilin | unknown | -32.64 | 16.24 | TRUE | FALSE | TRUE |
| **2623** | O14735 | CDP-diacylglycerol--inositol 3-phos | ER | -0.25 | 32.48 | FALSE | FALSE | TRUE |
| **2624** | Q6UXH1 | Cysteine-rich with EGF-like domain | unknown | -1.83 | 39.26 | FALSE | FALSE | TRUE |
| **2625** | Q9UPN6 | Protein SCAF8 | NUCLEUS | 3.85 | -31.21 | FALSE | FALSE | TRUE |
| **2626** | Q8N5K1 | CDGSH iron-sulfur domain-containi | ER | 1.07 | 31.61 | FALSE | FALSE | TRUE |
| **2627** | O95297 | Myelin protein zero-like protein 1 | PM | -21.83 | 10.09 | TRUE | FALSE | TRUE |
| **2628** | P38435 | Vitamin K-dependent gamma-carb | ER | 1.12 | 33.93 | FALSE | FALSE | TRUE |
| **2629** | Q9UMY1 | Nucleolar protein 7 | NUCLEUS | -1.41 | -39.51 | FALSE | FALSE | TRUE |
| **2630** | Q15011 | Homocysteine-responsive endopla | ER | -3.59 | 36.45 | TRUE | FALSE | FALSE |
| **2631** | Q9BRR6 | ADP-dependent glucokinase | ER | -3.99 | 32.71 | FALSE | FALSE | TRUE |
| **2632** | Q9NX46 | Poly(ADP-ribose) glycohydrolase A | unknown | 9.65 | 9.04 | FALSE | FALSE | TRUE |
| **2633** | Q5F1R6 | DnaJ homolog subfamily C membe | unknown | 11.73 | -20.39 | FALSE | FALSE | TRUE |
| **2634** | Q13535 | Serine/threonine-protein kinase AT | unknown | -12.22 | -11.67 | FALSE | FALSE | TRUE |
| **2635** | P49840 | Glycogen synthase kinase-3 alpha | unknown | -0.39 | -8.26 | FALSE | FALSE | TRUE |
| **2636** | P29317 | Ephrin type-A receptor 2 | PM | -26.25 | 13.89 | TRUE | FALSE | TRUE |
| **2637** | Q86TU7 | Histone-lysine N-methyltransferase | NUCLEUS | -1.17 | -15.62 | FALSE | FALSE | TRUE |
| **2638** | O95487 | Protein transport protein Sec24B | unknown | -10.94 | 7.32 | TRUE | FALSE | TRUE |
| **2639** | Q8ND24 | RING finger protein 214 | unknown | 6.60 | -22.78 | FALSE | FALSE | TRUE |
| **2640** | Q6ZU80 | Centrosomal protein of 128 kDa | unknown | -15.55 | -11.32 | FALSE | FALSE | TRUE |
| **2641** | P04156 | Major prion protein | unknown | -25.15 | 10.49 | FALSE | FALSE | TRUE |
| **2642** | Q8N8S7 | Protein enabled homolog | unknown | 1.39 | 9.45 | FALSE | FALSE | TRUE |
| **2643** | Q6R327 | Rapamycin-insensitive companion | unknown | 1.82 | -12.68 | TRUE | FALSE | TRUE |
| **2644** | Q68CP9 | AT-rich interactive domain-containi | unknown | -22.30 | -16.41 | TRUE | FALSE | TRUE |
| **2645** | Q5T200 | Zinc finger CCCH domain-containing | unknown | -13.20 | -18.88 | FALSE | FALSE | TRUE |
| **2646** | Q8WX92 | Negative elongation factor B | NUCLEUS | 10.08 | -5.27 | FALSE | FALSE | TRUE |
| **2647** | Q8IYB7 | DIS3-like exonuclease 2 | unknown | 11.75 | 4.74 | FALSE | FALSE | TRUE |
| **2648** | Q9NRY4 | Rho GTPase-activating protein 35 | unknown | -3.24 | -1.46 | FALSE | FALSE | TRUE |
| **2649** | Q9H1B7 | Interferon regulatory factor 2-bindi | NUCLEUS | -13.07 | -19.87 | FALSE | FALSE | TRUE |
| **2650** | P61764 | Syntaxin-binding protein 1 | unknown | -20.59 | 5.55 | FALSE | FALSE | TRUE |
| **2651** | Q9H4F8 | SPARC-related modular calcium-bin | unknown | -31.23 | 10.78 | FALSE | FALSE | TRUE |
| **2652** | Q99816 | Tumor susceptibility gene 101 prot | unknown | -9.06 | 4.13 | FALSE | FALSE | TRUE |
| **2653** | Q9BYD2 | 39S ribosomal protein L9, mitochon | unknown | 44.66 | -12.64 | FALSE | FALSE | TRUE |
| **2654** | O95248 | Myotubularin-related protein 5 | unknown | -1.41 | -7.72 | TRUE | FALSE | TRUE |
| **2655** | P05067 | Amyloid-beta A4 protein | unknown | -21.73 | 13.94 | FALSE | FALSE | TRUE |
| **2656** | Q9NP81 | Serine--tRNA ligase, mitochondrial | MITOCHONDRIA | 38.76 | -8.37 | FALSE | FALSE | TRUE |

| | | | | | | | | |
|---|---|---|---|---|---|---|---|---|
| **2657** | P11310 | Medium-chain specific acyl-CoA de | MITOCHONDRIA | 38.03 | -7.35 | FALSE | FALSE | TRUE |
| **2658** | Q8TAA9 | Vang-like protein 1 | PM | -24.79 | 18.00 | TRUE | FALSE | TRUE |
| **2659** | O94915 | Protein furry homolog-like | unknown | -1.79 | -7.18 | FALSE | FALSE | TRUE |
| **2660** | Q66K74 | Microtubule-associated protein 1S | NUCLEUS | -8.49 | -15.37 | FALSE | FALSE | TRUE |
| **2661** | Q5VUA4 | Zinc finger protein 318 | NUCLEUS | -9.04 | -23.03 | TRUE | FALSE | TRUE |
| **2662** | Q13596 | Sorting nexin-1 | unknown | -10.82 | 16.49 | TRUE | FALSE | TRUE |
| **2663** | O43556 | Epsilon-sarcoglycan | unknown | -15.52 | 15.90 | FALSE | FALSE | TRUE |
| **2664** | Q9NPA0 | ER membrane protein complex sub | unknown | 0.20 | 35.64 | FALSE | FALSE | TRUE |
| **2665** | Q7Z3K3 | Pogo transposable element with ZN | NUCLEUS | -24.77 | -23.80 | FALSE | FALSE | TRUE |
| **2666** | Q8WWN8 | Arf-GAP with Rho-GAP domain, ANK | unknown | -3.59 | 9.97 | FALSE | FALSE | TRUE |
| **2667** | O00400 | Acetyl-coenzyme A transporter 1 | ER | 2.76 | 31.38 | FALSE | FALSE | TRUE |
| **2668** | Q92541 | RNA polymerase-associated protein | unknown | 22.26 | 15.53 | FALSE | FALSE | TRUE |
| **2669** | Q9Y6I4 | Ubiquitin carboxyl-terminal hydrola | NUCLEUS-CHROMATIN | -21.33 | -13.44 | FALSE | FALSE | TRUE |
| **2670** | Q06481 | Amyloid-like protein 2 | PM | -29.87 | 12.50 | FALSE | FALSE | TRUE |
| **2671** | Q99720 | Sigma non-opioid intracellular recep | unknown | -0.89 | 32.57 | FALSE | FALSE | TRUE |
| **2672** | P54619 | 5'-AMP-activated protein kinase su | unknown | 9.70 | 4.65 | FALSE | FALSE | TRUE |
| **2673** | Q7Z2Z2 | Elongation factor-like GTPase 1 | unknown | 0.92 | -5.98 | FALSE | FALSE | TRUE |
| **2674** | Q92820 | Gamma-glutamyl hydrolase | LYSOSOME | -39.57 | 8.50 | FALSE | FALSE | TRUE |
| **2675** | P30825 | High affinity cationic amino acid tra | PM | -20.76 | 23.21 | TRUE | FALSE | TRUE |
| **2676** | O95149 | Snurportin-1 | unknown | 15.99 | 7.19 | FALSE | FALSE | TRUE |
| **2677** | Q02750 | Dual specificity mitogen-activated p | unknown | 15.12 | 5.63 | FALSE | FALSE | TRUE |
| **2678** | Q9NWV8 | BRISC and BRCA1-A complex memb | unknown | 12.43 | 1.68 | FALSE | FALSE | TRUE |
| **2679** | Q8NI35 | InaD-like protein | unknown | -15.70 | 13.53 | TRUE | FALSE | TRUE |
| **2680** | Q6IAN0 | Dehydrogenase/reductase SDR fam | ER | 2.62 | 31.96 | FALSE | FALSE | TRUE |
| **2681** | Q9HC52 | Chromobox protein homolog 8 | NUCLEUS-CHROMATIN | -21.73 | -13.90 | FALSE | FALSE | TRUE |
| **2682** | Q01995 | Transgelin | unknown | 13.09 | 10.79 | TRUE | FALSE | TRUE |
| **2683** | Q70J99 | Protein unc-13 homolog D | unknown | -2.34 | -7.24 | FALSE | FALSE | TRUE |
| **2684** | Q8NEY8 | Periphilin-1 | NUCLEUS | -2.52 | -32.59 | TRUE | FALSE | TRUE |
| **2685** | Q9Y6N7 | Roundabout homolog 1 | PM | -26.17 | 4.56 | TRUE | FALSE | TRUE |
| **2686** | Q7Z589 | BRCA2-interacting transcriptional re | NUCLEUS | -7.77 | -24.03 | TRUE | FALSE | TRUE |
| **2687** | Q5VYS8 | Terminal uridylyltransferase 7 | unknown | 3.29 | -17.20 | FALSE | FALSE | TRUE |
| **2688** | Q9UK45 | U6 snRNA-associated Sm-like prote | NUCLEUS | -10.29 | -30.49 | FALSE | FALSE | TRUE |
| **2689** | Q9H857 | 5'-nucleotidase domain-containing | MITOCHONDRIA | 40.79 | -10.29 | FALSE | FALSE | TRUE |
| **2690** | Q7Z4H3 | HD domain-containing protein 2 | unknown | 14.61 | 9.47 | FALSE | FALSE | TRUE |
| **2691** | Q6IN84 | rRNA methyltransferase 1, mitocho | MITOCHONDRIA | 36.80 | -5.92 | FALSE | FALSE | TRUE |
| **2692** | Q15742 | NGFI-A-binding protein 2 | NUCLEUS | -9.30 | -14.07 | TRUE | FALSE | FALSE |
| **2693** | Q15154 | Pericentriolar material 1 protein | unknown | -14.78 | -16.22 | TRUE | FALSE | TRUE |
| **2694** | Q9Y2X9 | Zinc finger protein 281 | NUCLEUS | -6.01 | -30.80 | TRUE | FALSE | TRUE |

| | | | | | | | | |
|---|---|---|---|---|---|---|---|---|
| **2695** | P11171 | Protein 4.1 | unknown | -16.99 | 14.33 | TRUE | FALSE | TRUE |
| **2696** | O43617 | Trafficking protein particle complex | unknown | -11.68 | 5.39 | FALSE | FALSE | TRUE |
| **2697** | Q5JTZ9 | Alanine--tRNA ligase, mitochondrial | MITOCHONDRIA | 37.23 | -7.72 | FALSE | FALSE | TRUE |
| **2698** | P54132 | Bloom syndrome protein | unknown | -22.93 | -19.37 | TRUE | FALSE | TRUE |
| **2699** | O43379 | WD repeat-containing protein 62 | unknown | 13.91 | 0.74 | FALSE | FALSE | TRUE |
| **2700** | Q9HCD6 | Protein TANC2 | unknown | -6.65 | 2.73 | TRUE | FALSE | TRUE |
| **2701** | Q9Y3A2 | Probable U3 small nucleolar RNA-as | NUCLEUS | -1.36 | -40.83 | FALSE | FALSE | TRUE |
| **2702** | Q12986 | Transcriptional repressor NF-X1 | unknown | 13.70 | -20.67 | TRUE | FALSE | TRUE |
| **2703** | O75962 | Triple functional domain protein | unknown | -5.04 | -7.52 | FALSE | FALSE | TRUE |
| **2704** | A1X283 | SH3 and PX domain-containing prot | unknown | -11.33 | -16.22 | FALSE | FALSE | TRUE |
| **2705** | O94855 | Protein transport protein Sec24D | unknown | 4.95 | -9.85 | FALSE | FALSE | TRUE |
| **2706** | Q5VTR2 | E3 ubiquitin-protein ligase BRE1A | NUCLEUS | -7.76 | -26.87 | FALSE | FALSE | TRUE |
| **2707** | O75146 | Huntingtin-interacting protein 1-rela | unknown | -6.41 | -7.83 | FALSE | FALSE | TRUE |
| **2708** | Q2TBE0 | CWF19-like protein 2 | NUCLEUS | -4.09 | -23.85 | TRUE | FALSE | TRUE |
| **2709** | Q8IZ21 | Phosphatase and actin regulator 4 | unknown | -1.26 | 12.44 | TRUE | FALSE | TRUE |
| **2710** | Q14118 | Dystroglycan | PM | -19.49 | 16.58 | TRUE | FALSE | FALSE |
| **2711** | Q7Z2K8 | G protein-regulated inducer of neur | PM | -24.30 | 4.61 | TRUE | FALSE | TRUE |
| **2712** | Q8IZA0 | Dyslexia-associated protein KIAA03 | unknown | -35.41 | 14.57 | TRUE | FALSE | TRUE |
| **2713** | Q13586 | Stromal interaction molecule 1 | ER | -2.35 | 34.28 | TRUE | FALSE | TRUE |
| **2714** | P46109 | Crk-like protein | unknown | 14.35 | 15.51 | TRUE | FALSE | FALSE |
| **2715** | Q8TF72 | Protein Shroom3 | unknown | -4.95 | 13.80 | TRUE | FALSE | TRUE |
| **2716** | P42566 | Epidermal growth factor receptor su | unknown | 7.80 | 12.65 | TRUE | FALSE | TRUE |
| **2717** | P07947 | Tyrosine-protein kinase Yes | PM | -25.63 | 12.21 | TRUE | FALSE | FALSE |
| **2718** | Q9Y5K6 | CD2-associated protein | NUCLEUS | -9.95 | -13.22 | TRUE | FALSE | FALSE |
| **2719** | Q2M2I8 | AP2-associated protein kinase 1 | unknown | -6.18 | 8.44 | TRUE | FALSE | FALSE |
| **2720** | P25054 | Adenomatous polyposis coli protein | unknown | -6.36 | 3.05 | TRUE | FALSE | TRUE |
| **2721** | Q9H1K0 | Rabenosyn-5 | unknown | 7.69 | -18.50 | TRUE | FALSE | TRUE |
| **2722** | Q641Q2 | WASH complex subunit 2A | unknown | -15.61 | 10.30 | TRUE | FALSE | FALSE |
| **2723** | Q96AT1 | Uncharacterized protein KIAA1143 | unknown | 10.77 | -3.22 | TRUE | FALSE | FALSE |

| | Abundances | | | | | | | | | |
|---|---|---|---|---|---|---|---|---|---|---|
| **Row Number** | Elute.GR | Elute.PCFT | Wash01.GR | Wash01.PCFT | Wash02.GR | Wash02.PCFT | EluteGRvsWash01GR | EluteGRvsWash02GR | ElutePCFTvsWash01PCFT | ElutePCFTvsWash02PCFT |
| **1** | 6.53E+08 | 8.00E+08 | 1.55E+12 | 1.50E+12 | 8.75E+09 | 9.96E+09 | 4.20E-04 | 7.46E-02 | 5.32E-04 | 8.04E-02 |
| **2** | NA | NA | 1.55E+12 | 1.50E+12 | 8.75E+09 | 9.96E+09 | NA | NA | NA | NA |
| **3** | 2.62E+09 | 7.88E+09 | 2.34E+11 | 2.78E+11 | 6.64E+09 | 9.11E+09 | 1.12E-02 | 3.95E-01 | 2.84E-02 | 8.65E-01 |
| **4** | 1.55E+07 | 6.02E+06 | 9.03E+10 | 9.05E+10 | 2.32E+08 | 1.70E+08 | 1.71E-04 | 6.69E-02 | 6.66E-05 | 3.54E-02 |
| **5** | 7.69E+06 | 1.06E+06 | 8.40E+10 | 8.49E+10 | 2.91E+08 | 2.53E+08 | 9.16E-05 | 2.65E-02 | 1.24E-05 | 4.18E-03 |
| **6** | 7.62E+07 | 4.07E+06 | 7.24E+10 | 8.43E+10 | 1.06E+08 | 1.44E+08 | 1.05E-03 | 7.16E-01 | 4.83E-05 | 2.83E-02 |
| **7** | 5.32E+07 | 6.42E+07 | 7.56E+10 | 7.99E+10 | 4.16E+08 | 3.21E+08 | 7.04E-04 | 1.28E-01 | 8.04E-04 | 2.00E-01 |
| **8** | 1.62E+08 | 2.23E+08 | 7.08E+10 | 7.95E+10 | 6.76E+08 | 1.01E+09 | 2.29E-03 | 2.40E-01 | 2.80E-03 | 2.22E-01 |
| **9** | 2.58E+08 | 7.05E+07 | 6.46E+10 | 8.38E+10 | 6.88E+08 | 1.28E+09 | 4.00E-03 | 3.75E-01 | 8.41E-04 | 5.50E-02 |
| **10** | 1.12E+07 | 1.27E+07 | 7.42E+10 | 6.74E+10 | 7.77E+07 | 9.97E+07 | 1.50E-04 | 1.44E-01 | 1.89E-04 | 1.28E-01 |
| **11** | 5.34E+06 | 1.59E+07 | 6.66E+10 | 5.98E+10 | 1.35E+08 | 1.48E+08 | 8.02E-05 | 3.96E-02 | 2.66E-04 | 1.07E-01 |
| **12** | 2.38E+07 | 3.05E+07 | 5.86E+10 | 6.42E+10 | 5.01E+08 | 4.69E+08 | 4.06E-04 | 4.75E-02 | 4.76E-04 | 6.51E-02 |
| **13** | 1.58E+07 | 1.90E+06 | 5.98E+10 | 5.97E+10 | 1.51E+08 | 1.47E+08 | 2.65E-04 | 1.05E-01 | 3.18E-05 | 1.30E-02 |
| **14** | 1.33E+07 | 3.77E+07 | 5.97E+10 | 5.84E+10 | 1.58E+08 | 1.87E+08 | 2.22E-04 | 8.41E-02 | 6.46E-04 | 2.02E-01 |
| **15** | 1.54E+07 | 5.83E+06 | 5.84E+10 | 5.82E+10 | 2.00E+08 | 1.42E+08 | 2.63E-04 | 7.68E-02 | 1.00E-04 | 4.10E-02 |
| **16** | NA | NA | 5.56E+10 | 5.35E+10 | 2.03E+08 | 1.13E+08 | NA | NA | NA | NA |
| **17** | 4.32E+07 | 3.39E+07 | 5.26E+10 | 5.26E+10 | 1.51E+08 | 1.76E+08 | 8.20E-04 | 2.85E-01 | 6.45E-04 | 1.93E-01 |
| **18** | 4.80E+07 | 7.93E+06 | 4.93E+10 | 5.27E+10 | 3.13E+08 | 2.26E+08 | 9.74E-04 | 1.54E-01 | 1.50E-04 | 3.51E-02 |
| **19** | 1.58E+07 | 1.90E+06 | 5.16E+10 | 5.06E+10 | 1.33E+08 | 1.22E+08 | 3.07E-04 | 1.19E-01 | 3.75E-05 | 1.55E-02 |
| **20** | 1.83E+06 | 6.19E+05 | 4.30E+10 | 4.41E+10 | 4.66E+07 | 6.17E+07 | 4.24E-05 | 3.92E-02 | 1.41E-05 | 1.00E-02 |
| **21** | 8.41E+05 | 1.29E+06 | 3.93E+10 | 4.38E+10 | 2.66E+07 | 2.86E+07 | 2.14E-05 | 3.17E-02 | 2.95E-05 | 4.53E-02 |
| **22** | 6.41E+07 | 1.81E+06 | 3.70E+10 | 3.69E+10 | 4.49E+07 | 5.95E+07 | 1.73E-03 | 1.43E+00 | 4.89E-05 | 3.03E-02 |
| **23** | NA | NA | 3.63E+10 | 3.60E+10 | 2.32E+07 | 4.11E+07 | NA | NA | NA | NA |
| **24** | 0.00E+00 | 4.48E+06 | 3.16E+10 | 3.64E+10 | 7.50E+07 | 1.07E+08 | 0.00E+00 | 0.00E+00 | 1.23E-04 | 4.19E-02 |
| **25** | 6.76E+06 | 1.32E+06 | 3.15E+10 | 3.62E+10 | 7.14E+07 | 8.78E+07 | 2.15E-04 | 9.47E-02 | 3.65E-05 | 1.50E-02 |
| **26** | NA | NA | 2.11E+10 | 4.38E+10 | 4.37E+08 | 3.32E+08 | NA | NA | NA | NA |
| **27** | 1.81E+07 | 3.14E+06 | 2.91E+10 | 3.16E+10 | 3.86E+07 | 5.03E+07 | 6.22E-04 | 4.70E-01 | 9.91E-05 | 6.23E-02 |
| **28** | NA | NA | 3.02E+10 | 3.00E+10 | 1.11E+08 | 1.10E+08 | NA | NA | NA | NA |
| **29** | NA | NA | 2.78E+10 | 2.98E+10 | 1.09E+08 | 1.15E+08 | NA | NA | NA | NA |
| **30** | 3.99E+05 | 9.55E+05 | 2.93E+10 | 2.75E+10 | 8.32E+07 | 7.45E+07 | 1.37E-05 | 4.80E-03 | 3.48E-05 | 1.28E-02 |
| **31** | 1.11E+07 | 6.98E+06 | 2.74E+10 | 2.85E+10 | 4.74E+07 | 4.69E+07 | 4.06E-04 | 2.35E-01 | 2.45E-04 | 1.49E-01 |
| **32** | 1.65E+07 | 1.46E+06 | 2.27E+10 | 3.13E+10 | 1.01E+08 | 7.98E+07 | 7.25E-04 | 1.63E-01 | 4.66E-05 | 1.83E-02 |
| **33** | NA | NA | 2.45E+10 | 2.63E+10 | 1.03E+08 | 7.37E+07 | NA | NA | NA | NA |
| **34** | 3.23E+08 | 1.05E+08 | 2.03E+10 | 2.45E+10 | 1.25E+09 | 1.49E+09 | 1.59E-02 | 2.58E-01 | 4.30E-03 | 7.09E-02 |

| | | | | | | | | | | |
|---|---|---|---|---|---|---|---|---|---|---|
| **35** | 6.09E+05 | 8.38E+05 | 2.23E+10 | 2.39E+10 | 6.67E+07 | 9.42E+07 | 2.73E-05 | 9.13E-03 | 3.51E-05 | 8.90E-03 |
| **36** | 1.87E+07 | 7.99E+06 | 2.32E+10 | 2.23E+10 | 8.34E+07 | 1.01E+08 | 8.07E-04 | 2.25E-01 | 3.58E-04 | 7.92E-02 |
| **37** | 1.97E+07 | 7.40E+06 | 2.20E+10 | 2.34E+10 | 6.76E+07 | 5.01E+07 | 8.96E-04 | 2.92E-01 | 3.17E-04 | 1.48E-01 |
| **38** | 8.36E+05 | 3.38E+06 | 2.23E+10 | 2.26E+10 | 5.45E+07 | 8.26E+07 | 3.75E-05 | 1.53E-02 | 1.49E-04 | 4.09E-02 |
| **39** | 0.00E+00 | 0.00E+00 | 2.04E+10 | 2.32E+10 | 3.44E+07 | 4.40E+07 | 0.00E+00 | 0.00E+00 | 0.00E+00 | 0.00E+00 |
| **40** | 6.95E+06 | 1.28E+07 | 2.17E+10 | 2.12E+10 | 7.91E+07 | 4.71E+07 | 3.21E-04 | 8.78E-02 | 6.01E-04 | 2.71E-01 |
| **41** | NA | NA | 2.11E+10 | 2.05E+10 | 2.70E+07 | 3.15E+07 | NA | NA | NA | NA |
| **42** | 2.67E+06 | 0.00E+00 | 1.99E+10 | 2.12E+10 | 2.28E+07 | 2.87E+07 | 1.34E-04 | 1.17E-01 | 0.00E+00 | 0.00E+00 |
| **43** | NA | NA | 1.95E+10 | 2.09E+10 | 6.72E+07 | 9.56E+07 | NA | NA | NA | NA |
| **44** | NA | NA | 1.90E+10 | 2.11E+10 | 3.57E+07 | 5.15E+07 | NA | NA | NA | NA |
| **45** | 1.20E+08 | 6.29E+07 | 1.79E+10 | 2.09E+10 | 4.64E+08 | 5.03E+08 | 6.68E-03 | 2.59E-01 | 3.01E-03 | 1.25E-01 |
| **46** | 2.34E+05 | 2.29E+06 | 1.84E+10 | 1.92E+10 | 6.47E+07 | 9.57E+07 | 1.28E-05 | 3.62E-03 | 1.20E-04 | 2.40E-02 |
| **47** | 0.00E+00 | 0.00E+00 | 1.78E+10 | 1.95E+10 | 1.07E+07 | 1.43E+07 | 0.00E+00 | 0.00E+00 | 0.00E+00 | 0.00E+00 |
| **48** | 8.25E+06 | 1.47E+06 | 1.72E+10 | 1.83E+10 | 2.36E+07 | 2.41E+07 | 4.80E-04 | 3.50E-01 | 8.06E-05 | 6.12E-02 |
| **49** | 5.44E+06 | 2.50E+06 | 1.67E+10 | 1.82E+10 | 1.93E+07 | 2.41E+07 | 3.26E-04 | 2.83E-01 | 1.37E-04 | 1.04E-01 |
| **50** | 1.30E+07 | 2.06E+07 | 1.53E+10 | 1.84E+10 | 1.90E+08 | 2.24E+08 | 8.45E-04 | 6.83E-02 | 1.12E-03 | 9.22E-02 |
| **51** | NA | NA | 1.63E+10 | 1.69E+10 | 6.77E+07 | 6.03E+07 | NA | NA | NA | NA |
| **52** | 7.50E+06 | 2.95E+06 | 1.54E+10 | 1.78E+10 | 5.49E+07 | 2.69E+07 | 4.87E-04 | 1.37E-01 | 1.66E-04 | 1.09E-01 |
| **53** | NA | NA | 1.64E+10 | 1.67E+10 | 3.92E+07 | 4.73E+07 | NA | NA | NA | NA |
| **54** | NA | NA | 1.68E+10 | 1.60E+10 | 2.79E+07 | 2.23E+07 | NA | NA | NA | NA |
| **55** | NA | NA | 1.71E+10 | 1.55E+10 | 1.22E+07 | 2.61E+07 | NA | NA | NA | NA |
| **56** | 0.00E+00 | 0.00E+00 | 1.46E+10 | 1.71E+10 | 9.33E+07 | 6.17E+07 | 0.00E+00 | 0.00E+00 | 0.00E+00 | 0.00E+00 |
| **57** | 8.55E+06 | 3.70E+05 | 1.39E+10 | 1.73E+10 | 7.78E+07 | 8.08E+07 | 6.14E-04 | 1.10E-01 | 2.14E-05 | 4.58E-03 |
| **58** | 8.55E+06 | 3.70E+05 | 1.39E+10 | 1.73E+10 | 7.78E+07 | 8.08E+07 | 6.14E-04 | 1.10E-01 | 2.14E-05 | 4.58E-03 |
| **59** | NA | NA | 1.29E+10 | 1.66E+10 | 5.73E+07 | 8.54E+07 | NA | NA | NA | NA |
| **60** | 2.57E+08 | 7.95E+07 | 1.43E+10 | 1.50E+10 | 6.25E+06 | 3.57E+07 | 1.80E-02 | 4.12E+01 | 5.31E-03 | 2.22E+00 |
| **61** | NA | NA | 1.38E+10 | 1.43E+10 | 4.43E+07 | 4.52E+07 | NA | NA | NA | NA |
| **62** | 0.00E+00 | 0.00E+00 | 1.36E+10 | 1.42E+10 | 4.37E+07 | 7.94E+07 | 0.00E+00 | 0.00E+00 | 0.00E+00 | 0.00E+00 |
| **63** | NA | NA | 1.38E+10 | 1.34E+10 | 4.01E+07 | 5.77E+07 | NA | NA | NA | NA |
| **64** | NA | NA | 1.38E+10 | 1.22E+10 | 7.49E+07 | 6.79E+07 | NA | NA | NA | NA |
| **65** | 8.94E+07 | 2.34E+08 | 1.23E+10 | 1.27E+10 | 4.03E+07 | 2.66E+07 | 7.24E-03 | 2.22E+00 | 1.84E-02 | 8.80E+00 |
| **66** | 1.69E+09 | 7.18E+09 | 1.31E+10 | 1.17E+10 | 2.17E+07 | 2.01E+08 | 1.29E-01 | 7.77E+01 | 6.16E-01 | 3.57E+01 |
| **67** | 0.00E+00 | 1.69E+05 | 1.20E+10 | 1.28E+10 | 2.09E+07 | 2.04E+07 | 0.00E+00 | 0.00E+00 | 1.33E-05 | 8.32E-03 |
| **68** | 1.69E+07 | 2.10E+06 | 1.22E+10 | 1.25E+10 | 4.01E+07 | 3.62E+07 | 1.39E-03 | 4.21E-01 | 1.69E-04 | 5.81E-02 |
| **69** | NA | NA | 1.07E+10 | 1.29E+10 | NA | NA | NA | NA | NA | NA |
| **70** | 7.47E+08 | 1.75E+08 | 1.25E+10 | 1.08E+10 | 1.01E+08 | 9.48E+07 | 5.98E-02 | 7.39E+00 | 1.63E-02 | 1.84E+00 |
| **71** | 0.00E+00 | 1.31E+06 | 1.15E+10 | 1.13E+10 | 1.83E+07 | 2.95E+07 | 0.00E+00 | 0.00E+00 | 1.15E-04 | 4.44E-02 |
| **72** | 0.00E+00 | 9.48E+05 | 9.79E+09 | 1.22E+10 | 3.70E+07 | 4.98E+07 | 0.00E+00 | 0.00E+00 | 7.77E-05 | 1.90E-02 |

| | | | | | | | | | | |
|---|---|---|---|---|---|---|---|---|---|---|
| **73** | NA | NA | 1.13E+10 | 1.05E+10 | 3.74E+07 | 5.12E+07 | NA | NA | NA | NA |
| **74** | 4.60E+05 | 9.81E+06 | 1.14E+10 | 1.05E+10 | 1.51E+07 | 3.86E+07 | 4.05E-05 | 3.04E-02 | 9.37E-04 | 2.54E-01 |
| **75** | 4.02E+05 | 0.00E+00 | 1.05E+10 | 1.10E+10 | 2.75E+07 | 5.20E+07 | 3.83E-05 | 1.46E-02 | 0.00E+00 | 0.00E+00 |
| **76** | NA | NA | 1.10E+10 | 1.05E+10 | 2.92E+07 | 3.64E+07 | NA | NA | NA | NA |
| **77** | NA | NA | 1.02E+10 | 1.13E+10 | 1.97E+07 | 2.41E+07 | NA | NA | NA | NA |
| **78** | 6.84E+06 | 3.25E+06 | 1.03E+10 | 1.11E+10 | 1.49E+07 | 1.69E+07 | 6.64E-04 | 4.59E-01 | 2.93E-04 | 1.92E-01 |
| **79** | 0.00E+00 | 1.53E+06 | 1.02E+10 | 1.10E+10 | 3.65E+07 | 4.30E+07 | 0.00E+00 | 0.00E+00 | 1.40E-04 | 3.57E-02 |
| **80** | 2.92E+06 | 3.19E+06 | 1.01E+10 | 1.09E+10 | 2.48E+07 | 3.38E+07 | 2.90E-04 | 1.18E-01 | 2.92E-04 | 9.44E-02 |
| **81** | 1.61E+09 | 7.09E+08 | 1.04E+10 | 1.04E+10 | 4.12E+07 | 1.80E+08 | 1.55E-01 | 3.91E+01 | 6.81E-02 | 3.93E+00 |
| **82** | 6.21E+07 | 4.03E+07 | 9.86E+09 | 1.11E+10 | 1.83E+07 | 2.71E+07 | 6.30E-03 | 3.40E+00 | 3.64E-03 | 1.49E+00 |
| **83** | 0.00E+00 | 3.50E+06 | 1.02E+10 | 1.04E+10 | 4.56E+07 | 5.30E+07 | 0.00E+00 | 0.00E+00 | 3.38E-04 | 6.60E-02 |
| **84** | 6.46E+06 | 4.01E+06 | 9.70E+09 | 1.08E+10 | 2.70E+07 | 2.70E+07 | 6.65E-04 | 2.39E-01 | 3.72E-04 | 1.49E-01 |
| **85** | NA | NA | 9.69E+09 | 1.07E+10 | 3.90E+07 | 6.29E+07 | NA | NA | NA | NA |
| **86** | NA | NA | 9.50E+09 | 1.08E+10 | 3.13E+07 | 3.67E+07 | NA | NA | NA | NA |
| **87** | NA | NA | 9.04E+09 | 1.08E+10 | 2.05E+07 | 2.20E+07 | NA | NA | NA | NA |
| **88** | 1.50E+07 | 1.44E+07 | 8.42E+09 | 1.10E+10 | 1.86E+07 | 2.45E+07 | 1.78E-03 | 8.05E-01 | 1.32E-03 | 5.90E-01 |
| **89** | 4.39E+06 | 0.00E+00 | 9.71E+09 | 9.55E+09 | 8.52E+06 | 1.02E+07 | 4.52E-04 | 5.15E-01 | 0.00E+00 | 0.00E+00 |
| **90** | 0.00E+00 | 1.42E+07 | 1.00E+10 | 8.54E+09 | 3.40E+07 | 5.03E+07 | 0.00E+00 | 0.00E+00 | 1.66E-03 | 2.82E-01 |
| **91** | NA | NA | 9.35E+09 | 9.24E+09 | 9.65E+06 | 7.62E+06 | NA | NA | NA | NA |
| **92** | NA | NA | 9.64E+09 | 8.83E+09 | 1.69E+07 | 8.64E+06 | NA | NA | NA | NA |
| **93** | NA | NA | 9.56E+09 | 8.87E+09 | 4.26E+07 | 1.84E+07 | NA | NA | NA | NA |
| **94** | 7.98E+06 | 2.65E+06 | 8.99E+09 | 9.35E+09 | 5.67E+07 | 5.85E+07 | 8.88E-04 | 1.41E-01 | 2.83E-04 | 4.53E-02 |
| **95** | NA | NA | 8.72E+09 | 9.51E+09 | 5.26E+06 | 5.22E+06 | NA | NA | NA | NA |
| **96** | NA | NA | 9.66E+09 | 8.50E+09 | 1.05E+07 | 1.74E+07 | NA | NA | NA | NA |
| **97** | NA | NA | 9.04E+09 | 8.93E+09 | 2.00E+07 | 3.46E+07 | NA | NA | NA | NA |
| **98** | NA | NA | 9.04E+09 | 8.94E+09 | 1.77E+07 | 1.29E+07 | NA | NA | NA | NA |
| **99** | 0.00E+00 | 5.90E+05 | 8.90E+09 | 8.87E+09 | 2.19E+07 | 1.98E+07 | 0.00E+00 | 0.00E+00 | 6.65E-05 | 2.98E-02 |
| **100** | NA | NA | 9.24E+09 | 8.37E+09 | 1.12E+07 | 9.77E+06 | NA | NA | NA | NA |
| **101** | 0.00E+00 | 0.00E+00 | 8.94E+09 | 8.34E+09 | 2.27E+07 | 1.32E+07 | 0.00E+00 | 0.00E+00 | 0.00E+00 | 0.00E+00 |
| **102** | 1.88E+07 | 3.45E+06 | 8.31E+09 | 8.97E+09 | 2.05E+07 | 8.49E+06 | 2.27E-03 | 9.17E-01 | 3.84E-04 | 4.06E-01 |
| **103** | NA | NA | 9.08E+09 | 8.11E+09 | 3.65E+07 | 1.42E+07 | NA | NA | NA | NA |
| **104** | NA | NA | 8.64E+09 | 8.50E+09 | 9.38E+06 | 1.64E+07 | NA | NA | NA | NA |
| **105** | NA | NA | 8.74E+09 | 8.37E+09 | 2.06E+07 | 1.66E+07 | NA | NA | NA | NA |
| **106** | 8.75E+07 | 0.00E+00 | 7.29E+09 | 9.37E+09 | 3.41E+07 | 2.51E+07 | 1.20E-02 | 2.57E+00 | 0.00E+00 | 0.00E+00 |
| **107** | 3.09E+05 | 0.00E+00 | 9.84E+09 | 6.82E+09 | 8.08E+06 | 4.62E+06 | 3.14E-05 | 3.83E-02 | 0.00E+00 | 0.00E+00 |
| **108** | 3.08E+06 | 1.79E+06 | 8.66E+09 | 7.72E+09 | 2.76E+07 | 2.41E+07 | 3.56E-04 | 1.12E-01 | 2.32E-04 | 7.44E-02 |
| **109** | NA | NA | 8.69E+09 | 7.59E+09 | 3.60E+07 | 6.82E+07 | NA | NA | NA | NA |
| **110** | NA | NA | 7.90E+09 | 8.29E+09 | 1.30E+07 | 1.72E+07 | NA | NA | NA | NA |

| | | | | | | | | | | |
|---|---|---|---|---|---|---|---|---|---|---|
| **111** | NA | NA | 7.49E+09 | 8.47E+09 | 2.89E+07 | 8.95E+06 | NA | NA | NA | NA |
| **112** | 2.94E+06 | 1.66E+07 | 7.96E+09 | 7.86E+09 | 2.47E+07 | 9.65E+06 | 3.70E-04 | 1.19E-01 | 2.11E-03 | 1.72E+00 |
| **113** | NA | NA | 6.46E+09 | 8.64E+09 | 2.49E+07 | 2.95E+07 | NA | NA | NA | NA |
| **114** | 3.26E+05 | 0.00E+00 | 7.65E+09 | 7.45E+09 | 1.43E+07 | 1.53E+07 | 4.26E-05 | 2.29E-02 | 0.00E+00 | 0.00E+00 |
| **115** | NA | NA | 7.56E+09 | 7.49E+09 | 1.16E+07 | 1.73E+07 | NA | NA | NA | NA |
| **116** | NA | NA | 7.53E+09 | 7.42E+09 | 1.50E+07 | 1.96E+07 | NA | NA | NA | NA |
| **117** | 3.57E+07 | 0.00E+00 | 7.30E+09 | 7.40E+09 | 5.10E+06 | 1.14E+07 | 4.89E-03 | 7.00E+00 | 0.00E+00 | 0.00E+00 |
| **118** | 2.64E+07 | 2.07E+07 | 7.39E+09 | 7.16E+09 | 2.90E+07 | 2.79E+07 | 3.57E-03 | 9.09E-01 | 2.89E-03 | 7.43E-01 |
| **119** | 4.26E+05 | 0.00E+00 | 7.13E+09 | 6.91E+09 | 1.08E+07 | 7.92E+06 | 5.97E-05 | 3.96E-02 | 0.00E+00 | 0.00E+00 |
| **120** | 1.31E+06 | 0.00E+00 | 6.92E+09 | 7.08E+09 | 1.68E+07 | 1.59E+07 | 1.90E-04 | 7.82E-02 | 0.00E+00 | 0.00E+00 |
| **121** | NA | NA | 6.85E+09 | 7.07E+09 | 5.03E+07 | 1.99E+07 | NA | NA | NA | NA |
| **122** | 0.00E+00 | 1.13E+06 | 6.22E+09 | 7.65E+09 | 3.16E+07 | 5.00E+07 | 0.00E+00 | 0.00E+00 | 1.48E-04 | 2.26E-02 |
| **123** | NA | NA | 6.82E+09 | 7.01E+09 | 3.16E+07 | 4.08E+07 | NA | NA | NA | NA |
| **124** | 2.07E+06 | 0.00E+00 | 7.50E+09 | 6.30E+09 | 1.58E+07 | 1.65E+07 | 2.76E-04 | 1.32E-01 | 0.00E+00 | 0.00E+00 |
| **125** | 1.55E+05 | 4.30E+07 | 7.37E+09 | 6.36E+09 | 1.71E+07 | 9.17E+06 | 2.11E-05 | 9.08E-03 | 6.77E-03 | 4.69E+00 |
| **126** | 5.18E+05 | 0.00E+00 | 7.16E+09 | 6.39E+09 | 1.63E+07 | 1.36E+07 | 7.23E-05 | 3.18E-02 | 0.00E+00 | 0.00E+00 |
| **127** | 2.76E+06 | 0.00E+00 | 6.74E+09 | 6.60E+09 | 2.28E+07 | 1.90E+07 | 4.09E-04 | 1.21E-01 | 0.00E+00 | 0.00E+00 |
| **128** | 1.88E+07 | 4.31E+05 | 6.32E+09 | 7.04E+09 | 1.48E+07 | 1.11E+07 | 2.97E-03 | 1.26E+00 | 6.13E-05 | 3.87E-02 |
| **129** | 1.88E+07 | 4.31E+05 | 6.32E+09 | 7.04E+09 | 1.48E+07 | 1.11E+07 | 2.97E-03 | 1.26E+00 | 6.13E-05 | 3.87E-02 |
| **130** | NA | NA | 6.67E+09 | 6.62E+09 | 9.00E+06 | 1.19E+07 | NA | NA | NA | NA |
| **131** | NA | NA | 5.94E+09 | 7.19E+09 | 2.54E+07 | 2.34E+07 | NA | NA | NA | NA |
| **132** | NA | NA | 6.62E+09 | 6.42E+09 | 2.11E+07 | 2.02E+07 | NA | NA | NA | NA |
| **133** | NA | NA | 6.59E+09 | 6.15E+09 | 2.40E+07 | 3.12E+07 | NA | NA | NA | NA |
| **134** | NA | NA | 6.26E+09 | 6.33E+09 | 1.50E+07 | 1.48E+07 | NA | NA | NA | NA |
| **135** | NA | NA | 5.39E+09 | 7.04E+09 | 7.41E+06 | 6.07E+06 | NA | NA | NA | NA |
| **136** | NA | NA | 6.90E+09 | 5.47E+09 | 1.74E+07 | 1.20E+07 | NA | NA | NA | NA |
| **137** | 0.00E+00 | 0.00E+00 | 5.86E+09 | 6.44E+09 | 1.20E+07 | 2.04E+07 | 0.00E+00 | 0.00E+00 | 0.00E+00 | 0.00E+00 |
| **138** | NA | NA | 6.08E+09 | 5.99E+09 | 4.43E+07 | 2.51E+07 | NA | NA | NA | NA |
| **139** | NA | NA | 5.71E+09 | 6.28E+09 | 2.69E+07 | 3.14E+07 | NA | NA | NA | NA |
| **140** | 0.00E+00 | 1.30E+05 | 5.95E+09 | 5.97E+09 | 1.85E+07 | 2.33E+07 | 0.00E+00 | 0.00E+00 | 2.18E-05 | 5.59E-03 |
| **141** | NA | NA | 6.23E+09 | 5.70E+09 | 1.06E+07 | 1.30E+07 | NA | NA | NA | NA |
| **142** | 0.00E+00 | 1.15E+07 | 5.18E+09 | 6.60E+09 | 2.66E+07 | 2.79E+07 | 0.00E+00 | 0.00E+00 | 1.75E-03 | 4.15E-01 |
| **143** | NA | NA | 5.90E+09 | 5.83E+09 | 2.57E+07 | 5.16E+07 | NA | NA | NA | NA |
| **144** | NA | NA | 5.71E+09 | 6.03E+09 | 1.54E+07 | 9.13E+06 | NA | NA | NA | NA |
| **145** | NA | NA | 6.14E+09 | 5.36E+09 | 4.09E+07 | 1.87E+07 | NA | NA | NA | NA |
| **146** | NA | NA | 6.07E+09 | 5.41E+09 | 7.47E+06 | 7.37E+06 | NA | NA | NA | NA |
| **147** | 0.00E+00 | 3.35E+06 | 5.37E+09 | 6.06E+09 | 1.57E+07 | 1.35E+07 | 0.00E+00 | 0.00E+00 | 5.53E-04 | 2.49E-01 |
| **148** | 8.59E+06 | 0.00E+00 | 5.88E+09 | 5.53E+09 | 5.08E+06 | 7.82E+06 | 1.46E-03 | 1.69E+00 | 0.00E+00 | 0.00E+00 |

| | | | | | | | | | | |
|---|---|---|---|---|---|---|---|---|---|---|
| **149** | 2.01E+07 | 7.16E+05 | 5.71E+09 | 5.68E+09 | 1.15E+07 | 8.96E+06 | 3.52E-03 | 1.75E+00 | 1.26E-04 | 7.99E-02 |
| **150** | 2.68E+06 | 0.00E+00 | 5.56E+09 | 5.79E+09 | 1.60E+07 | 1.33E+07 | 4.82E-04 | 1.67E-01 | 0.00E+00 | 0.00E+00 |
| **151** | 2.39E+07 | 3.69E+06 | 5.66E+09 | 5.55E+09 | 1.55E+07 | 1.25E+07 | 4.23E-03 | 1.54E+00 | 6.65E-04 | 2.96E-01 |
| **152** | NA | NA | 5.72E+09 | 5.48E+09 | 2.30E+07 | 9.26E+06 | NA | NA | NA | NA |
| **153** | 2.15E+06 | 1.40E+05 | 5.41E+09 | 5.73E+09 | 2.14E+07 | 1.44E+07 | 3.98E-04 | 1.01E-01 | 2.45E-05 | 9.77E-03 |
| **154** | 4.55E+06 | 1.74E+05 | 5.71E+09 | 5.45E+09 | 8.61E+06 | 8.17E+06 | 7.96E-04 | 5.29E-01 | 3.20E-05 | 2.13E-02 |
| **155** | NA | NA | 6.04E+09 | 5.05E+09 | 1.13E+07 | 5.41E+06 | NA | NA | NA | NA |
| **156** | 8.33E+07 | 0.00E+00 | 5.51E+09 | 5.53E+09 | 2.17E+07 | 2.92E+07 | 1.51E-02 | 3.84E+00 | 0.00E+00 | 0.00E+00 |
| **157** | 0.00E+00 | 4.97E+06 | 5.18E+09 | 5.84E+09 | 1.63E+07 | 1.38E+07 | 0.00E+00 | 0.00E+00 | 8.51E-04 | 3.59E-01 |
| **158** | 5.38E+06 | 0.00E+00 | 5.20E+09 | 5.42E+09 | 3.69E+07 | 4.97E+07 | 1.03E-03 | 1.46E-01 | 0.00E+00 | 0.00E+00 |
| **159** | 1.25E+07 | 3.94E+06 | 5.43E+09 | 5.21E+09 | 9.14E+06 | 1.63E+07 | 2.30E-03 | 1.37E+00 | 7.57E-04 | 2.42E-01 |
| **160** | 0.00E+00 | 4.51E+06 | 5.49E+09 | 5.04E+09 | 4.43E+07 | 2.53E+07 | 0.00E+00 | 0.00E+00 | 8.94E-04 | 1.78E-01 |
| **161** | NA | NA | 5.17E+09 | 5.33E+09 | 5.10E+06 | 8.27E+06 | NA | NA | NA | NA |
| **162** | NA | NA | 4.53E+09 | 5.86E+09 | 2.71E+07 | 2.47E+07 | NA | NA | NA | NA |
| **163** | NA | NA | 5.49E+09 | 4.88E+09 | 1.84E+07 | 2.00E+07 | NA | NA | NA | NA |
| **164** | NA | NA | 5.61E+09 | 4.66E+09 | 1.00E+07 | 1.03E+07 | NA | NA | NA | NA |
| **165** | NA | NA | 5.27E+09 | 4.98E+09 | 5.85E+06 | 8.59E+06 | NA | NA | NA | NA |
| **166** | NA | NA | 4.49E+09 | 5.67E+09 | 1.79E+07 | 8.53E+06 | NA | NA | NA | NA |
| **167** | 2.22E+06 | 0.00E+00 | 5.13E+09 | 5.04E+09 | 8.47E+06 | 7.29E+06 | 4.32E-04 | 2.62E-01 | 0.00E+00 | 0.00E+00 |
| **168** | NA | NA | 5.94E+09 | 4.09E+09 | 5.17E+06 | 2.80E+07 | NA | NA | NA | NA |
| **169** | 0.00E+00 | 1.71E+06 | 4.80E+09 | 5.12E+09 | 3.55E+07 | 4.19E+07 | 0.00E+00 | 0.00E+00 | 3.34E-04 | 4.08E-02 |
| **170** | 0.00E+00 | 0.00E+00 | 5.07E+09 | 4.87E+09 | 2.08E+07 | 2.46E+07 | 0.00E+00 | 0.00E+00 | 0.00E+00 | 0.00E+00 |
| **171** | 0.00E+00 | 3.81E+05 | 4.33E+09 | 5.65E+09 | 2.97E+06 | 3.46E+06 | 0.00E+00 | 0.00E+00 | 6.76E-05 | 1.10E-01 |
| **172** | 2.73E+06 | 0.00E+00 | 4.44E+09 | 5.44E+09 | 1.27E+07 | 1.17E+07 | 6.14E-04 | 2.15E-01 | 0.00E+00 | 0.00E+00 |
| **173** | NA | NA | 5.05E+09 | 4.81E+09 | 1.72E+07 | 1.39E+07 | NA | NA | NA | NA |
| **174** | NA | NA | 5.27E+09 | 4.31E+09 | 1.65E+07 | 1.77E+07 | NA | NA | NA | NA |
| **175** | NA | NA | 5.08E+09 | 4.45E+09 | 1.02E+07 | 6.45E+06 | NA | NA | NA | NA |
| **176** | NA | NA | 4.76E+09 | 4.69E+09 | 4.84E+07 | 2.86E+07 | NA | NA | NA | NA |
| **177** | 0.00E+00 | 0.00E+00 | 5.02E+09 | 4.46E+09 | 2.68E+07 | 1.84E+07 | 0.00E+00 | 0.00E+00 | 0.00E+00 | 0.00E+00 |
| **178** | NA | NA | 4.92E+09 | 4.42E+09 | 6.10E+07 | 6.96E+07 | NA | NA | NA | NA |
| **179** | NA | NA | 4.57E+09 | 4.86E+09 | 2.94E+07 | 1.13E+07 | NA | NA | NA | NA |
| **180** | NA | NA | 4.14E+09 | 5.30E+09 | 1.21E+07 | 1.10E+07 | NA | NA | NA | NA |
| **181** | 1.96E+07 | 4.93E+05 | 4.74E+09 | 4.70E+09 | 8.75E+06 | 1.31E+07 | 4.13E-03 | 2.24E+00 | 1.05E-04 | 3.78E-02 |
| **182** | 0.00E+00 | 1.14E+07 | 5.08E+09 | 4.34E+09 | 2.09E+07 | 1.23E+07 | 0.00E+00 | 0.00E+00 | 2.62E-03 | 9.28E-01 |
| **183** | NA | NA | 4.88E+09 | 4.46E+09 | 8.59E+06 | 9.55E+06 | NA | NA | NA | NA |
| **184** | NA | NA | 4.15E+09 | 5.11E+09 | 1.87E+07 | 1.61E+07 | NA | NA | NA | NA |
| **185** | NA | NA | 4.85E+09 | 4.35E+09 | 1.48E+07 | 1.23E+07 | NA | NA | NA | NA |
| **186** | NA | NA | 4.90E+09 | 4.25E+09 | 8.50E+06 | 2.91E+07 | NA | NA | NA | NA |

| | | | | | | | | | | |
|---|---|---|---|---|---|---|---|---|---|---|
| **187** | NA | NA | 4.54E+09 | 4.64E+09 | 3.86E+06 | 4.96E+06 | NA | NA | NA | NA |
| **188** | NA | NA | 4.85E+09 | 4.28E+09 | 1.76E+07 | 1.72E+07 | NA | NA | NA | NA |
| **189** | NA | NA | 4.12E+09 | 4.98E+09 | 1.58E+07 | 2.33E+07 | NA | NA | NA | NA |
| **190** | 6.06E+05 | 0.00E+00 | 4.57E+09 | 4.43E+09 | 1.25E+07 | 1.15E+07 | 1.33E-04 | 4.84E-02 | 0.00E+00 | 0.00E+00 |
| **191** | NA | NA | 3.76E+09 | 5.19E+09 | 2.51E+07 | 2.50E+07 | NA | NA | NA | NA |
| **192** | NA | NA | 4.18E+09 | 4.72E+09 | 1.57E+07 | 1.44E+07 | NA | NA | NA | NA |
| **193** | 1.41E+07 | 0.00E+00 | 4.61E+09 | 4.28E+09 | 8.97E+06 | 5.65E+06 | 3.05E-03 | 1.57E+00 | 0.00E+00 | 0.00E+00 |
| **194** | NA | NA | 4.53E+09 | 4.25E+09 | 4.43E+07 | 2.48E+07 | NA | NA | NA | NA |
| **195** | 3.31E+06 | 0.00E+00 | 4.79E+09 | 3.97E+09 | 2.58E+07 | 2.07E+07 | 6.92E-04 | 1.28E-01 | 0.00E+00 | 0.00E+00 |
| **196** | NA | NA | 4.26E+09 | 4.45E+09 | 7.52E+06 | 2.88E+06 | NA | NA | NA | NA |
| **197** | NA | NA | 4.27E+09 | 4.41E+09 | 1.39E+07 | 1.80E+07 | NA | NA | NA | NA |
| **198** | NA | NA | 4.44E+09 | 4.18E+09 | 8.14E+06 | 9.81E+06 | NA | NA | NA | NA |
| **199** | 1.97E+07 | 3.12E+07 | 4.36E+09 | 4.24E+09 | 1.97E+07 | 1.19E+07 | 4.53E-03 | 1.00E+00 | 7.37E-03 | 2.62E+00 |
| **200** | NA | NA | 4.31E+09 | 4.20E+09 | 4.03E+07 | 3.10E+07 | NA | NA | NA | NA |
| **201** | NA | NA | 4.13E+09 | 4.31E+09 | 1.12E+07 | 2.99E+07 | NA | NA | NA | NA |
| **202** | NA | NA | 4.55E+09 | 3.86E+09 | 1.94E+07 | 7.56E+06 | NA | NA | NA | NA |
| **203** | NA | NA | 4.56E+09 | 3.78E+09 | 1.41E+07 | 2.09E+07 | NA | NA | NA | NA |
| **204** | 7.29E+06 | 0.00E+00 | 4.65E+09 | 3.66E+09 | 1.05E+07 | 1.66E+07 | 1.57E-03 | 6.93E-01 | 0.00E+00 | 0.00E+00 |
| **205** | NA | NA | 3.57E+09 | 4.68E+09 | 1.65E+07 | 1.23E+07 | NA | NA | NA | NA |
| **206** | 2.83E+06 | 0.00E+00 | 4.02E+09 | 4.21E+09 | 1.34E+07 | 1.46E+07 | 7.04E-04 | 2.11E-01 | 0.00E+00 | 0.00E+00 |
| **207** | NA | NA | 3.84E+09 | 4.39E+09 | 1.17E+07 | 1.62E+07 | NA | NA | NA | NA |
| **208** | 0.00E+00 | 5.98E+06 | 4.29E+09 | 3.88E+09 | 7.06E+06 | 6.85E+06 | 0.00E+00 | 0.00E+00 | 1.54E-03 | 8.73E-01 |
| **209** | NA | NA | 4.22E+09 | 3.92E+09 | 9.82E+06 | 7.75E+06 | NA | NA | NA | NA |
| **210** | NA | NA | 4.49E+09 | 3.55E+09 | 2.24E+07 | 1.16E+07 | NA | NA | NA | NA |
| **211** | NA | NA | 2.64E+09 | 5.26E+09 | 3.21E+07 | 3.51E+07 | NA | NA | NA | NA |
| **212** | NA | NA | 3.85E+09 | 4.10E+09 | 6.32E+06 | 5.52E+06 | NA | NA | NA | NA |
| **213** | NA | NA | 4.38E+09 | 3.57E+09 | NA | NA | NA | NA | NA | NA |
| **214** | NA | NA | 3.52E+09 | 4.36E+09 | 2.75E+07 | 2.87E+07 | NA | NA | NA | NA |
| **215** | NA | NA | 4.18E+09 | 3.74E+09 | 9.98E+06 | 7.75E+06 | NA | NA | NA | NA |
| **216** | 2.21E+06 | 1.26E+06 | 4.29E+09 | 3.50E+09 | 1.76E+07 | 1.88E+07 | 5.14E-04 | 1.25E-01 | 3.59E-04 | 6.68E-02 |
| **217** | NA | NA | 4.01E+09 | 3.77E+09 | 5.38E+06 | 4.20E+06 | NA | NA | NA | NA |
| **218** | 1.09E+07 | 4.83E+06 | 3.73E+09 | 3.98E+09 | 1.99E+07 | 2.44E+07 | 2.91E-03 | 5.46E-01 | 1.21E-03 | 1.98E-01 |
| **219** | NA | NA | 3.97E+09 | 3.74E+09 | 2.22E+07 | 1.38E+07 | NA | NA | NA | NA |
| **220** | 7.88E+05 | 0.00E+00 | 3.73E+09 | 3.97E+09 | 9.42E+06 | 9.11E+06 | 2.11E-04 | 8.37E-02 | 0.00E+00 | 0.00E+00 |
| **221** | NA | NA | 3.25E+09 | 4.41E+09 | 2.18E+07 | 2.06E+07 | NA | NA | NA | NA |
| **222** | 0.00E+00 | 5.59E+06 | 3.84E+09 | 3.80E+09 | 1.21E+07 | 4.79E+06 | 0.00E+00 | 0.00E+00 | 1.47E-03 | 1.17E+00 |
| **223** | NA | NA | 3.92E+09 | 3.69E+09 | 5.56E+06 | 6.09E+06 | NA | NA | NA | NA |
| **224** | NA | NA | 3.78E+09 | 3.76E+09 | 6.21E+06 | 9.68E+06 | NA | NA | NA | NA |

| | | | | | | | | | | |
|---|---|---|---|---|---|---|---|---|---|---|
| **225** | NA | NA | 3.86E+09 | 3.61E+09 | 1.21E+07 | 3.66E+06 | NA | NA | NA | NA |
| **226** | NA | NA | 4.07E+09 | 3.36E+09 | 6.62E+06 | 8.27E+06 | NA | NA | NA | NA |
| **227** | 0.00E+00 | 5.22E+06 | 3.57E+09 | 3.76E+09 | 1.02E+07 | 9.08E+07 | 0.00E+00 | 0.00E+00 | 1.39E-03 | 5.75E-02 |
| **228** | 2.23E+06 | 0.00E+00 | 3.71E+09 | 3.66E+09 | 1.97E+07 | 8.74E+06 | 6.02E-04 | 1.14E-01 | 0.00E+00 | 0.00E+00 |
| **229** | NA | NA | 3.84E+09 | 3.45E+09 | 6.67E+06 | 5.79E+06 | NA | NA | NA | NA |
| **230** | NA | NA | NA | NA | 3.45E+09 | 3.82E+09 | NA | NA | NA | NA |
| **231** | 3.00E+05 | 7.47E+05 | 3.82E+09 | 3.39E+09 | 8.24E+06 | 2.60E+06 | 7.83E-05 | 3.63E-02 | 2.21E-04 | 2.87E-01 |
| **232** | NA | NA | 3.71E+09 | 3.50E+09 | 4.05E+06 | 6.15E+06 | NA | NA | NA | NA |
| **233** | NA | NA | 3.86E+09 | 3.34E+09 | 1.45E+07 | 4.51E+06 | NA | NA | NA | NA |
| **234** | NA | NA | 3.67E+09 | 3.48E+09 | 5.23E+06 | 9.31E+06 | NA | NA | NA | NA |
| **235** | NA | NA | 4.09E+09 | 3.04E+09 | 1.01E+07 | 1.72E+07 | NA | NA | NA | NA |
| **236** | NA | NA | 3.59E+09 | 3.48E+09 | 4.43E+07 | 2.41E+07 | NA | NA | NA | NA |
| **237** | NA | NA | 3.88E+09 | 3.17E+09 | 9.02E+06 | 1.36E+07 | NA | NA | NA | NA |
| **238** | NA | NA | 3.52E+09 | 3.52E+09 | 1.37E+07 | 8.00E+06 | NA | NA | NA | NA |
| **239** | NA | NA | 3.27E+09 | 3.64E+09 | 1.19E+07 | 1.35E+07 | NA | NA | NA | NA |
| **240** | 0.00E+00 | 0.00E+00 | 3.08E+09 | 3.84E+09 | 1.33E+07 | 6.58E+06 | 0.00E+00 | 0.00E+00 | 0.00E+00 | 0.00E+00 |
| **241** | NA | NA | 3.49E+09 | 3.38E+09 | 4.43E+07 | 2.39E+07 | NA | NA | NA | NA |
| **242** | 1.35E+06 | 0.00E+00 | 3.76E+09 | 3.12E+09 | 5.03E+06 | 7.58E+06 | 3.60E-04 | 2.69E-01 | 0.00E+00 | 0.00E+00 |
| **243** | NA | NA | 3.83E+09 | 2.91E+09 | 1.19E+07 | 1.91E+07 | NA | NA | NA | NA |
| **244** | 5.42E+06 | 0.00E+00 | 3.29E+09 | 3.39E+09 | 2.74E+07 | 1.24E+07 | 1.65E-03 | 1.98E-01 | 0.00E+00 | 0.00E+00 |
| **245** | NA | NA | 3.58E+09 | 2.98E+09 | 1.04E+07 | 5.86E+06 | NA | NA | NA | NA |
| **246** | 5.25E+06 | 1.35E+05 | 3.34E+09 | 3.10E+09 | 9.05E+06 | 3.97E+06 | 1.57E-03 | 5.80E-01 | 4.34E-05 | 3.39E-02 |
| **247** | 3.80E+07 | 0.00E+00 | 3.15E+09 | 3.26E+09 | 1.93E+07 | 1.99E+07 | 1.20E-02 | 1.96E+00 | 0.00E+00 | 0.00E+00 |
| **248** | 9.41E+06 | 0.00E+00 | 3.05E+09 | 3.35E+09 | 4.06E+06 | 3.42E+06 | 3.08E-03 | 2.32E+00 | 0.00E+00 | 0.00E+00 |
| **249** | NA | NA | 3.13E+09 | 3.19E+09 | 1.92E+07 | 1.01E+07 | NA | NA | NA | NA |
| **250** | 1.58E+06 | 0.00E+00 | 3.16E+09 | 3.01E+09 | 7.69E+06 | 9.29E+06 | 5.01E-04 | 2.06E-01 | 0.00E+00 | 0.00E+00 |
| **251** | 9.29E+06 | 0.00E+00 | 2.88E+09 | 3.17E+09 | 6.51E+06 | 6.74E+06 | 3.23E-03 | 1.43E+00 | 0.00E+00 | 0.00E+00 |
| **252** | NA | NA | 2.80E+09 | 3.22E+09 | 3.49E+06 | 1.66E+06 | NA | NA | NA | NA |
| **253** | NA | NA | 2.77E+09 | 3.23E+09 | 4.19E+06 | 3.38E+06 | NA | NA | NA | NA |
| **254** | NA | NA | 3.49E+09 | 2.46E+09 | 7.49E+06 | 1.40E+07 | NA | NA | NA | NA |
| **255** | NA | NA | 2.83E+09 | 3.11E+09 | 1.36E+07 | 2.09E+06 | NA | NA | NA | NA |
| **256** | NA | NA | 2.83E+09 | 3.11E+09 | 1.36E+07 | 2.09E+06 | NA | NA | NA | NA |
| **257** | 0.00E+00 | 1.38E+06 | 2.99E+09 | 2.89E+09 | 1.18E+07 | 1.73E+07 | 0.00E+00 | 0.00E+00 | 4.77E-04 | 7.99E-02 |
| **258** | NA | NA | 2.95E+09 | 2.88E+09 | 1.47E+07 | 9.07E+06 | NA | NA | NA | NA |
| **259** | 6.87E+07 | 3.82E+06 | 2.84E+09 | 2.98E+09 | 4.94E+06 | 7.30E+06 | 2.42E-02 | 1.39E+01 | 1.28E-03 | 5.24E-01 |
| **260** | 0.00E+00 | 3.27E+06 | 3.07E+09 | 2.71E+09 | 8.79E+06 | 9.68E+06 | 0.00E+00 | 0.00E+00 | 1.21E-03 | 3.38E-01 |
| **261** | 1.42E+05 | 1.61E+05 | 3.14E+09 | 2.63E+09 | 4.62E+06 | 2.35E+06 | 4.51E-05 | 3.06E-02 | 6.11E-05 | 6.83E-02 |
| **262** | NA | NA | 2.93E+09 | 2.79E+09 | 4.50E+06 | 5.55E+06 | NA | NA | NA | NA |

| | | | | | | | | | | |
|---|---|---|---|---|---|---|---|---|---|---|
| **263** | 0.00E+00 | 2.57E+06 | 3.06E+09 | 2.64E+09 | 3.55E+06 | 3.36E+06 | 0.00E+00 | 0.00E+00 | 9.76E-04 | 7.66E-01 |
| **264** | NA | NA | 2.89E+09 | 2.73E+09 | 1.05E+07 | 4.96E+06 | NA | NA | NA | NA |
| **265** | NA | NA | 2.96E+09 | 2.67E+09 | 4.86E+06 | 3.75E+06 | NA | NA | NA | NA |
| **266** | 8.16E+05 | 0.00E+00 | 2.85E+09 | 2.76E+09 | 2.67E+06 | 4.11E+06 | 2.86E-04 | 3.06E-01 | 0.00E+00 | 0.00E+00 |
| **267** | NA | NA | 3.31E+09 | 2.28E+09 | 8.85E+06 | 5.33E+06 | NA | NA | NA | NA |
| **268** | NA | NA | 2.75E+09 | 2.83E+09 | 1.10E+07 | 9.35E+06 | NA | NA | NA | NA |
| **269** | 6.09E+06 | 0.00E+00 | 2.80E+09 | 2.78E+09 | 5.69E+06 | 3.85E+06 | 2.17E-03 | 1.07E+00 | 0.00E+00 | 0.00E+00 |
| **270** | NA | NA | 2.80E+09 | 2.78E+09 | 3.15E+06 | 3.69E+06 | NA | NA | NA | NA |
| **271** | 0.00E+00 | 0.00E+00 | 1.92E+09 | 3.53E+09 | 2.93E+07 | 4.26E+07 | 0.00E+00 | 0.00E+00 | 0.00E+00 | 0.00E+00 |
| **272** | NA | NA | 2.47E+09 | 3.03E+09 | 1.05E+07 | 3.92E+06 | NA | NA | NA | NA |
| **273** | 9.67E+04 | 0.00E+00 | 2.57E+09 | 2.85E+09 | 1.80E+07 | 3.83E+06 | 3.77E-05 | 5.38E-03 | 0.00E+00 | 0.00E+00 |
| **274** | 4.37E+07 | 3.02E+06 | 3.06E+09 | 2.29E+09 | 2.21E+07 | 2.49E+07 | 1.43E-02 | 1.98E+00 | 1.32E-03 | 1.21E-01 |
| **275** | NA | NA | 2.77E+09 | 2.55E+09 | 6.36E+06 | 4.73E+06 | NA | NA | NA | NA |
| **276** | NA | NA | 2.58E+09 | 2.65E+09 | 1.17E+07 | 1.73E+07 | NA | NA | NA | NA |
| **277** | NA | NA | 2.78E+09 | 2.46E+09 | 3.50E+06 | 5.51E+06 | NA | NA | NA | NA |
| **278** | NA | NA | 2.64E+09 | 2.59E+09 | 6.94E+06 | 3.04E+06 | NA | NA | NA | NA |
| **279** | NA | NA | 2.70E+09 | 2.51E+09 | 2.85E+06 | 3.61E+06 | NA | NA | NA | NA |
| **280** | NA | NA | 2.77E+09 | 2.39E+09 | 3.52E+06 | 4.06E+06 | NA | NA | NA | NA |
| **281** | 4.16E+06 | 1.28E+05 | 2.50E+09 | 2.64E+09 | 1.38E+07 | 9.25E+06 | 1.66E-03 | 3.02E-01 | 4.85E-05 | 1.38E-02 |
| **282** | NA | NA | 2.56E+09 | 2.57E+09 | 8.74E+06 | 1.09E+07 | NA | NA | NA | NA |
| **283** | NA | NA | 2.89E+09 | 2.22E+09 | 7.95E+06 | 1.09E+07 | NA | NA | NA | NA |
| **284** | NA | NA | 2.62E+09 | 2.39E+09 | 4.76E+06 | 3.22E+06 | NA | NA | NA | NA |
| **285** | NA | NA | 2.49E+09 | 2.50E+09 | 1.42E+07 | 9.51E+06 | NA | NA | NA | NA |
| **286** | 0.00E+00 | 1.95E+09 | 3.37E+09 | 1.64E+09 | 1.12E+05 | 0.00E+00 | 0.00E+00 | 0.00E+00 | 1.19E+00 | Inf |
| **287** | NA | NA | 2.82E+09 | 2.17E+09 | 3.80E+06 | 6.14E+06 | NA | NA | NA | NA |
| **288** | NA | NA | 2.64E+09 | 2.34E+09 | 5.33E+06 | 3.56E+06 | NA | NA | NA | NA |
| **289** | 2.05E+05 | 0.00E+00 | 2.13E+09 | 2.82E+09 | 1.26E+07 | 1.47E+07 | 9.61E-05 | 1.62E-02 | 0.00E+00 | 0.00E+00 |
| **290** | NA | NA | 2.37E+09 | 2.56E+09 | 1.11E+07 | 5.86E+06 | NA | NA | NA | NA |
| **291** | 3.46E+07 | 5.87E+05 | 2.24E+09 | 2.67E+09 | 2.14E+07 | 1.23E+07 | 1.55E-02 | 1.62E+00 | 2.20E-04 | 4.79E-02 |
| **292** | NA | NA | 2.53E+09 | 2.39E+09 | 5.83E+06 | 5.35E+06 | NA | NA | NA | NA |
| **293** | NA | NA | 2.61E+09 | 2.29E+09 | 6.60E+06 | 4.32E+06 | NA | NA | NA | NA |
| **294** | NA | NA | 2.27E+09 | 2.62E+09 | 4.42E+06 | 3.96E+06 | NA | NA | NA | NA |
| **295** | 2.49E+08 | 1.57E+08 | 2.17E+09 | 2.63E+09 | 3.32E+07 | 3.47E+07 | 1.15E-01 | 7.50E+00 | 5.98E-02 | 4.54E+00 |
| **296** | 3.26E+05 | 0.00E+00 | 2.61E+09 | 2.14E+09 | 1.08E+07 | 5.13E+06 | 1.25E-04 | 3.01E-02 | 0.00E+00 | 0.00E+00 |
| **297** | NA | NA | 2.39E+09 | 2.36E+09 | 3.78E+06 | 4.00E+06 | NA | NA | NA | NA |
| **298** | NA | NA | 2.26E+09 | 2.48E+09 | 7.51E+06 | 3.25E+06 | NA | NA | NA | NA |
| **299** | NA | NA | 2.33E+09 | 2.36E+09 | 2.87E+07 | 1.38E+07 | NA | NA | NA | NA |
| **300** | NA | NA | 2.49E+09 | 2.22E+09 | 1.40E+07 | 5.31E+06 | NA | NA | NA | NA |

| | | | | | | | | | | |
|---|---|---|---|---|---|---|---|---|---|---|
| **301** | NA | NA | 2.49E+09 | 2.13E+09 | 2.51E+06 | 3.94E+06 | NA | NA | NA | NA |
| **302** | NA | NA | 2.40E+09 | 2.19E+09 | 3.07E+06 | 2.74E+06 | NA | NA | NA | NA |
| **303** | NA | NA | 2.55E+09 | 2.01E+09 | 6.43E+06 | 7.18E+06 | NA | NA | NA | NA |
| **304** | 5.18E+06 | 0.00E+00 | 2.40E+09 | 2.13E+09 | 6.33E+06 | 4.72E+06 | 2.16E-03 | 8.18E-01 | 0.00E+00 | 0.00E+00 |
| **305** | 1.97E+06 | 2.94E+06 | 2.26E+09 | 2.22E+09 | 2.04E+07 | 4.24E+06 | 8.71E-04 | 9.66E-02 | 1.32E-03 | 6.93E-01 |
| **306** | NA | NA | 2.40E+09 | 2.09E+09 | 5.08E+06 | 4.19E+06 | NA | NA | NA | NA |
| **307** | NA | NA | 2.23E+09 | 2.24E+09 | 7.52E+06 | 6.72E+05 | NA | NA | NA | NA |
| **308** | NA | NA | 2.07E+09 | 2.40E+09 | 3.75E+06 | 4.42E+06 | NA | NA | NA | NA |
| **309** | NA | NA | 2.15E+09 | 2.31E+09 | 7.74E+06 | 5.31E+06 | NA | NA | NA | NA |
| **310** | 4.75E+06 | 0.00E+00 | 2.08E+09 | 2.35E+09 | 5.74E+06 | 5.44E+06 | 2.28E-03 | 8.29E-01 | 0.00E+00 | 0.00E+00 |
| **311** | NA | NA | 2.16E+09 | 2.25E+09 | 4.15E+06 | 1.39E+06 | NA | NA | NA | NA |
| **312** | NA | NA | 2.41E+09 | 2.00E+09 | 5.73E+06 | 2.85E+06 | NA | NA | NA | NA |
| **313** | NA | NA | 2.16E+09 | 2.23E+09 | 2.95E+06 | 1.86E+06 | NA | NA | NA | NA |
| **314** | NA | NA | 2.30E+09 | 2.04E+09 | 7.48E+06 | 8.39E+06 | NA | NA | NA | NA |
| **315** | NA | NA | 1.95E+09 | 2.37E+09 | 6.84E+06 | 2.43E+06 | NA | NA | NA | NA |
| **316** | NA | NA | 2.20E+09 | 2.09E+09 | 4.29E+05 | 6.46E+05 | NA | NA | NA | NA |
| **317** | NA | NA | 2.04E+09 | 2.22E+09 | 6.46E+06 | 3.08E+06 | NA | NA | NA | NA |
| **318** | NA | NA | 2.40E+09 | 1.86E+09 | 1.32E+06 | 2.57E+06 | NA | NA | NA | NA |
| **319** | NA | NA | 2.18E+09 | 2.04E+09 | 7.94E+06 | 7.18E+06 | NA | NA | NA | NA |
| **320** | NA | NA | 2.04E+09 | 2.16E+09 | 1.43E+06 | 2.45E+06 | NA | NA | NA | NA |
| **321** | NA | NA | 2.09E+09 | 2.07E+09 | 5.00E+06 | 3.19E+06 | NA | NA | NA | NA |
| **322** | NA | NA | 2.31E+09 | 1.82E+09 | 3.01E+06 | 2.55E+06 | NA | NA | NA | NA |
| **323** | NA | NA | 2.22E+09 | 1.86E+09 | 4.67E+06 | 8.63E+06 | NA | NA | NA | NA |
| **324** | 0.00E+00 | 4.67E+05 | 1.95E+09 | 2.02E+09 | 8.64E+06 | 8.97E+06 | 0.00E+00 | 0.00E+00 | 2.31E-04 | 5.20E-02 |
| **325** | 4.14E+05 | 0.00E+00 | 1.98E+09 | 1.99E+09 | 7.14E+06 | 8.34E+06 | 2.09E-04 | 5.80E-02 | 0.00E+00 | 0.00E+00 |
| **326** | NA | NA | 2.01E+09 | 1.94E+09 | 1.14E+07 | 7.66E+06 | NA | NA | NA | NA |
| **327** | 0.00E+00 | 1.66E+05 | 1.42E+09 | 2.53E+09 | 4.50E+06 | 1.44E+06 | 0.00E+00 | 0.00E+00 | 6.56E-05 | 1.15E-01 |
| **328** | 4.06E+06 | 0.00E+00 | 1.92E+09 | 2.02E+09 | 1.37E+07 | 3.87E+06 | 2.12E-03 | 2.97E-01 | 0.00E+00 | 0.00E+00 |
| **329** | NA | NA | 1.81E+09 | 2.11E+09 | 7.82E+06 | 6.40E+06 | NA | NA | NA | NA |
| **330** | NA | NA | 1.86E+09 | 2.04E+09 | 8.96E+06 | 6.20E+06 | NA | NA | NA | NA |
| **331** | NA | NA | 2.11E+09 | 1.80E+09 | 4.29E+06 | 3.67E+06 | NA | NA | NA | NA |
| **332** | 0.00E+00 | 0.00E+00 | 2.13E+09 | 1.77E+09 | 4.46E+06 | 4.47E+06 | 0.00E+00 | 0.00E+00 | 0.00E+00 | 0.00E+00 |
| **333** | 0.00E+00 | 6.82E+05 | 2.03E+09 | 1.86E+09 | 1.22E+07 | 4.85E+06 | 0.00E+00 | 0.00E+00 | 3.67E-04 | 1.41E-01 |
| **334** | NA | NA | 1.87E+09 | 1.98E+09 | 4.83E+06 | 5.41E+06 | NA | NA | NA | NA |
| **335** | 1.49E+07 | 6.78E+05 | 1.99E+09 | 1.78E+09 | 4.41E+06 | 4.18E+06 | 7.49E-03 | 3.38E+00 | 3.81E-04 | 1.62E-01 |
| **336** | NA | NA | 1.79E+09 | 1.95E+09 | 9.94E+06 | 1.40E+07 | NA | NA | NA | NA |
| **337** | NA | NA | 2.01E+09 | 1.74E+09 | 3.64E+06 | 2.73E+06 | NA | NA | NA | NA |
| **338** | 0.00E+00 | 6.13E+07 | 1.87E+09 | 1.86E+09 | 1.31E+07 | 1.14E+07 | 0.00E+00 | 0.00E+00 | 3.30E-02 | 5.37E+00 |

| | | | | | | | | | | |
|---|---|---|---|---|---|---|---|---|---|---|
| **339** | NA | NA | 1.96E+09 | 1.77E+09 | 1.20E+07 | 8.64E+06 | NA | NA | NA | NA |
| **340** | NA | NA | 1.95E+09 | 1.78E+09 | 2.48E+06 | 2.49E+06 | NA | NA | NA | NA |
| **341** | NA | NA | 1.98E+09 | 1.74E+09 | 2.77E+06 | 4.68E+06 | NA | NA | NA | NA |
| **342** | 1.70E+07 | 4.87E+05 | 2.02E+09 | 1.69E+09 | 5.08E+06 | 4.62E+06 | 8.43E-03 | 3.34E+00 | 2.89E-04 | 1.05E-01 |
| **343** | NA | NA | 1.98E+09 | 1.72E+09 | 2.81E+06 | 4.11E+06 | NA | NA | NA | NA |
| **344** | 6.08E+06 | 0.00E+00 | 1.79E+09 | 1.90E+09 | 6.38E+06 | 5.19E+06 | 3.40E-03 | 9.53E-01 | 0.00E+00 | 0.00E+00 |
| **345** | NA | NA | 1.94E+09 | 1.74E+09 | 7.35E+06 | 1.57E+07 | NA | NA | NA | NA |
| **346** | NA | NA | 2.03E+09 | 1.63E+09 | 8.87E+06 | 1.52E+07 | NA | NA | NA | NA |
| **347** | 6.10E+05 | 0.00E+00 | 1.78E+09 | 1.89E+09 | 3.84E+06 | 2.44E+06 | 3.42E-04 | 1.59E-01 | 0.00E+00 | 0.00E+00 |
| **348** | NA | NA | 1.91E+09 | 1.75E+09 | 3.59E+06 | 3.73E+06 | NA | NA | NA | NA |
| **349** | NA | NA | 1.88E+09 | 1.75E+09 | 4.75E+06 | 4.81E+06 | NA | NA | NA | NA |
| **350** | NA | NA | 2.08E+09 | 1.52E+09 | 3.01E+06 | 1.29E+06 | NA | NA | NA | NA |
| **351** | NA | NA | 1.57E+09 | 2.02E+09 | 2.84E+06 | 4.38E+06 | NA | NA | NA | NA |
| **352** | NA | NA | 1.79E+09 | 1.78E+09 | 2.44E+06 | 3.06E+06 | NA | NA | NA | NA |
| **353** | NA | NA | 1.94E+09 | 1.59E+09 | 2.81E+06 | 3.45E+06 | NA | NA | NA | NA |
| **354** | 7.96E+06 | 3.35E+05 | 1.53E+09 | 1.97E+09 | 5.44E+06 | 5.43E+06 | 5.19E-03 | 1.46E+00 | 1.70E-04 | 6.16E-02 |
| **355** | NA | NA | 1.73E+09 | 1.78E+09 | 3.12E+06 | 5.32E+06 | NA | NA | NA | NA |
| **356** | NA | NA | 1.45E+09 | 2.03E+09 | 1.01E+07 | 3.21E+06 | NA | NA | NA | NA |
| **357** | NA | NA | 1.82E+09 | 1.67E+09 | 2.10E+06 | 2.69E+06 | NA | NA | NA | NA |
| **358** | NA | NA | 1.70E+09 | 1.76E+09 | 1.82E+07 | 9.63E+06 | NA | NA | NA | NA |
| **359** | 3.18E+05 | 0.00E+00 | 1.73E+09 | 1.73E+09 | 2.91E+06 | 4.92E+06 | 1.84E-04 | 1.09E-01 | 0.00E+00 | 0.00E+00 |
| **360** | NA | NA | 1.72E+09 | 1.72E+09 | 7.40E+06 | 4.81E+06 | NA | NA | NA | NA |
| **361** | NA | NA | 1.58E+09 | 1.86E+09 | 7.08E+06 | 9.50E+06 | NA | NA | NA | NA |
| **362** | NA | NA | 1.63E+09 | 1.80E+09 | 7.67E+06 | 7.08E+06 | NA | NA | NA | NA |
| **363** | 1.26E+07 | 2.09E+07 | 2.12E+09 | 1.30E+09 | 2.09E+06 | 2.72E+06 | 5.96E-03 | 6.04E+00 | 1.61E-02 | 7.69E+00 |
| **364** | 1.26E+07 | 2.09E+07 | 2.12E+09 | 1.30E+09 | 2.09E+06 | 2.72E+06 | 5.96E-03 | 6.04E+00 | 1.61E-02 | 7.69E+00 |
| **365** | 0.00E+00 | 3.07E+06 | 1.79E+09 | 1.61E+09 | 7.95E+06 | 9.10E+06 | 0.00E+00 | 0.00E+00 | 1.90E-03 | 3.37E-01 |
| **366** | NA | NA | 1.58E+09 | 1.81E+09 | 1.13E+07 | 4.53E+06 | NA | NA | NA | NA |
| **367** | NA | NA | 1.72E+09 | 1.66E+09 | 6.94E+06 | 3.17E+06 | NA | NA | NA | NA |
| **368** | NA | NA | 1.64E+09 | 1.75E+09 | 2.09E+06 | 3.30E+06 | NA | NA | NA | NA |
| **369** | NA | NA | 1.50E+09 | 1.88E+09 | 2.73E+06 | 2.53E+06 | NA | NA | NA | NA |
| **370** | NA | NA | 1.78E+09 | 1.60E+09 | 3.52E+06 | 3.20E+06 | NA | NA | NA | NA |
| **371** | NA | NA | 1.82E+09 | 1.53E+09 | 5.15E+06 | 1.36E+06 | NA | NA | NA | NA |
| **372** | NA | NA | 1.50E+09 | 1.86E+09 | 1.51E+06 | 3.43E+06 | NA | NA | NA | NA |
| **373** | NA | NA | 1.42E+09 | 1.93E+09 | 6.33E+06 | 6.45E+06 | NA | NA | NA | NA |
| **374** | NA | NA | 1.72E+09 | 1.62E+09 | 1.00E+06 | 2.93E+06 | NA | NA | NA | NA |
| **375** | 0.00E+00 | 9.57E+06 | 1.56E+09 | 1.70E+09 | 3.23E+06 | 3.18E+06 | 0.00E+00 | 0.00E+00 | 5.63E-03 | 3.01E+00 |
| **376** | NA | NA | 1.61E+09 | 1.58E+09 | 3.89E+06 | 5.15E+06 | NA | NA | NA | NA |

| | | | | | | | | | | |
|---|---|---|---|---|---|---|---|---|---|---|
| **377** | NA | NA | 1.69E+09 | 1.47E+09 | 5.06E+06 | 4.09E+06 | NA | NA | NA | NA |
| **378** | NA | NA | 1.49E+09 | 1.66E+09 | 6.86E+06 | 5.55E+06 | NA | NA | NA | NA |
| **379** | 2.23E+06 | 0.00E+00 | 1.46E+09 | 1.68E+09 | 5.11E+06 | 6.20E+06 | 1.53E-03 | 4.36E-01 | 0.00E+00 | 0.00E+00 |
| **380** | NA | NA | 1.45E+09 | 1.69E+09 | 2.72E+06 | 2.56E+06 | NA | NA | NA | NA |
| **381** | NA | NA | 1.60E+09 | 1.52E+09 | 5.16E+06 | 2.19E+06 | NA | NA | NA | NA |
| **382** | NA | NA | 1.56E+09 | 1.48E+09 | 4.12E+06 | 4.15E+06 | NA | NA | NA | NA |
| **383** | NA | NA | 1.23E+09 | 1.79E+09 | 9.58E+06 | 1.20E+07 | NA | NA | NA | NA |
| **384** | NA | NA | 1.50E+09 | 1.53E+09 | 2.68E+06 | 3.87E+06 | NA | NA | NA | NA |
| **385** | 1.21E+08 | 1.56E+07 | 1.33E+09 | 1.69E+09 | 7.56E+06 | 9.00E+06 | 9.16E-02 | 1.61E+01 | 9.22E-03 | 1.73E+00 |
| **386** | 4.17E+06 | 1.33E+07 | 1.58E+09 | 1.42E+09 | 3.26E+06 | 3.20E+06 | 2.64E-03 | 1.28E+00 | 9.38E-03 | 4.16E+00 |
| **387** | NA | NA | 1.46E+09 | 1.52E+09 | 9.15E+06 | 1.47E+07 | NA | NA | NA | NA |
| **388** | NA | NA | 1.58E+09 | 1.41E+09 | NA | NA | NA | NA | NA | NA |
| **389** | NA | NA | 1.47E+09 | 1.51E+09 | 8.57E+06 | 2.97E+06 | NA | NA | NA | NA |
| **390** | NA | NA | 1.64E+09 | 1.33E+09 | 1.05E+06 | 5.48E+06 | NA | NA | NA | NA |
| **391** | NA | NA | 1.64E+09 | 1.32E+09 | NA | NA | NA | NA | NA | NA |
| **392** | NA | NA | 1.55E+09 | 1.39E+09 | 3.15E+06 | 3.17E+06 | NA | NA | NA | NA |
| **393** | NA | NA | 1.63E+09 | 1.29E+09 | 4.96E+06 | 4.69E+06 | NA | NA | NA | NA |
| **394** | NA | NA | 1.23E+09 | 1.64E+09 | 3.37E+07 | 1.63E+07 | NA | NA | NA | NA |
| **395** | NA | NA | 1.51E+09 | 1.39E+09 | 3.79E+06 | 2.13E+06 | NA | NA | NA | NA |
| **396** | NA | NA | 1.48E+09 | 1.42E+09 | 1.84E+06 | 2.13E+06 | NA | NA | NA | NA |
| **397** | NA | NA | 1.40E+09 | 1.50E+09 | 3.53E+06 | 2.57E+06 | NA | NA | NA | NA |
| **398** | NA | NA | 1.32E+09 | 1.58E+09 | 2.12E+06 | 1.87E+06 | NA | NA | NA | NA |
| **399** | 2.26E+06 | 5.09E+06 | 1.66E+09 | 1.22E+09 | 6.36E+06 | 2.15E+06 | 1.36E-03 | 3.56E-01 | 4.16E-03 | 2.37E+00 |
| **400** | NA | NA | 1.95E+09 | 8.92E+08 | 2.80E+07 | 2.59E+07 | NA | NA | NA | NA |
| **401** | NA | NA | 1.47E+09 | 1.38E+09 | 2.51E+06 | 1.86E+06 | NA | NA | NA | NA |
| **402** | NA | NA | 1.57E+09 | 1.28E+09 | 5.50E+06 | 4.19E+06 | NA | NA | NA | NA |
| **403** | NA | NA | 1.48E+09 | 1.38E+09 | 1.75E+06 | 1.11E+06 | NA | NA | NA | NA |
| **404** | 8.59E+06 | 3.26E+07 | 1.37E+09 | 1.46E+09 | 4.18E+06 | 5.40E+06 | 6.28E-03 | 2.06E+00 | 2.24E-02 | 6.03E+00 |
| **405** | 2.03E+06 | 0.00E+00 | 1.33E+09 | 1.47E+09 | 1.72E+07 | 1.44E+07 | 1.53E-03 | 1.19E-01 | 0.00E+00 | 0.00E+00 |
| **406** | NA | NA | 1.29E+09 | 1.53E+09 | NA | NA | NA | NA | NA | NA |
| **407** | 1.91E+07 | 0.00E+00 | 1.32E+09 | 1.49E+09 | 4.70E+06 | 2.87E+06 | 1.45E-02 | 4.06E+00 | 0.00E+00 | 0.00E+00 |
| **408** | NA | NA | 1.42E+09 | 1.39E+09 | 3.29E+06 | 5.27E+06 | NA | NA | NA | NA |
| **409** | 0.00E+00 | 4.72E+07 | 1.46E+09 | 1.33E+09 | 6.45E+06 | 2.74E+06 | 0.00E+00 | 0.00E+00 | 3.56E-02 | 1.72E+01 |
| **410** | NA | NA | 1.34E+09 | 1.43E+09 | 2.24E+06 | 2.23E+06 | NA | NA | NA | NA |
| **411** | 2.12E+05 | 0.00E+00 | 1.43E+09 | 1.29E+09 | 1.88E+07 | 1.61E+07 | 1.48E-04 | 1.13E-02 | 0.00E+00 | 0.00E+00 |
| **412** | NA | NA | 1.24E+09 | 1.47E+09 | 1.27E+07 | 7.27E+06 | NA | NA | NA | NA |
| **413** | NA | NA | 1.57E+09 | 1.16E+09 | 1.24E+06 | 7.48E+05 | NA | NA | NA | NA |
| **414** | 0.00E+00 | 0.00E+00 | 1.43E+09 | 1.27E+09 | 1.04E+07 | 1.12E+07 | 0.00E+00 | 0.00E+00 | 0.00E+00 | 0.00E+00 |

| | | | | | | | | | | |
|---|---|---|---|---|---|---|---|---|---|---|
| **415** | NA | NA | 1.29E+09 | 1.42E+09 | 2.24E+06 | 1.98E+06 | NA | NA | NA | NA |
| **416** | NA | NA | 1.39E+09 | 1.32E+09 | 3.13E+06 | 5.23E+06 | NA | NA | NA | NA |
| **417** | NA | NA | 1.67E+09 | 1.02E+09 | 7.87E+06 | 6.21E+06 | NA | NA | NA | NA |
| **418** | 4.28E+05 | 1.02E+06 | 1.34E+09 | 1.35E+09 | 3.33E+06 | 4.00E+06 | 3.20E-04 | 1.29E-01 | 7.56E-04 | 2.54E-01 |
| **419** | NA | NA | 1.42E+09 | 1.26E+09 | 4.57E+06 | 5.56E+06 | NA | NA | NA | NA |
| **420** | 2.88E+05 | 0.00E+00 | 1.19E+09 | 1.48E+09 | 3.52E+06 | 3.05E+06 | 2.42E-04 | 8.19E-02 | 0.00E+00 | 0.00E+00 |
| **421** | NA | NA | 1.46E+09 | 1.18E+09 | 3.48E+06 | 1.50E+06 | NA | NA | NA | NA |
| **422** | NA | NA | 1.23E+09 | 1.40E+09 | 4.73E+06 | 6.09E+05 | NA | NA | NA | NA |
| **423** | NA | NA | 1.31E+09 | 1.30E+09 | 5.81E+06 | 6.19E+06 | NA | NA | NA | NA |
| **424** | NA | NA | 1.35E+09 | 1.23E+09 | 2.41E+06 | 1.23E+06 | NA | NA | NA | NA |
| **425** | NA | NA | 1.15E+09 | 1.42E+09 | 2.50E+06 | 1.88E+06 | NA | NA | NA | NA |
| **426** | NA | NA | 1.24E+09 | 1.31E+09 | 9.22E+06 | 7.72E+06 | NA | NA | NA | NA |
| **427** | 1.57E+07 | 0.00E+00 | 1.25E+09 | 1.30E+09 | 5.72E+06 | 3.33E+06 | 1.26E-02 | 2.74E+00 | 0.00E+00 | 0.00E+00 |
| **428** | NA | NA | 1.07E+09 | 1.46E+09 | 7.58E+06 | 9.13E+06 | NA | NA | NA | NA |
| **429** | NA | NA | 1.17E+09 | 1.33E+09 | 8.69E+06 | 8.58E+06 | NA | NA | NA | NA |
| **430** | 1.23E+07 | 1.43E+06 | 1.30E+09 | 1.17E+09 | 1.39E+07 | 5.83E+06 | 9.48E-03 | 8.87E-01 | 1.23E-03 | 2.46E-01 |
| **431** | NA | NA | 1.26E+09 | 1.16E+09 | 7.90E+06 | 3.19E+06 | NA | NA | NA | NA |
| **432** | NA | NA | 1.31E+09 | 1.11E+09 | 8.51E+06 | 4.15E+06 | NA | NA | NA | NA |
| **433** | NA | NA | 1.36E+09 | 1.05E+09 | 6.43E+05 | 1.07E+06 | NA | NA | NA | NA |
| **434** | 9.02E+06 | 5.08E+06 | 1.36E+09 | 1.04E+09 | 2.97E+06 | 2.29E+06 | 6.62E-03 | 3.04E+00 | 4.89E-03 | 2.22E+00 |
| **435** | 4.29E+06 | 8.13E+06 | 9.78E+08 | 1.37E+09 | 2.86E+07 | 1.39E+07 | 4.39E-03 | 1.50E-01 | 5.92E-03 | 5.84E-01 |
| **436** | 0.00E+00 | 3.22E+06 | 1.27E+09 | 1.11E+09 | 5.92E+06 | 6.27E+06 | 0.00E+00 | 0.00E+00 | 2.90E-03 | 5.14E-01 |
| **437** | NA | NA | 1.22E+09 | 1.15E+09 | 7.75E+06 | 4.83E+06 | NA | NA | NA | NA |
| **438** | NA | NA | 1.18E+09 | 1.19E+09 | 4.52E+06 | 2.62E+06 | NA | NA | NA | NA |
| **439** | NA | NA | 1.26E+09 | 1.11E+09 | 7.03E+06 | 3.67E+06 | NA | NA | NA | NA |
| **440** | NA | NA | 4.34E+08 | 1.94E+09 | 1.97E+06 | 1.87E+06 | NA | NA | NA | NA |
| **441** | NA | NA | 1.43E+09 | 9.33E+08 | 2.99E+06 | 5.01E+05 | NA | NA | NA | NA |
| **442** | 6.83E+06 | 0.00E+00 | 1.08E+09 | 1.27E+09 | 6.85E+06 | 2.51E+06 | 6.30E-03 | 9.96E-01 | 0.00E+00 | 0.00E+00 |
| **443** | 6.83E+06 | 0.00E+00 | 1.08E+09 | 1.27E+09 | 6.85E+06 | 2.51E+06 | 6.30E-03 | 9.96E-01 | 0.00E+00 | 0.00E+00 |
| **444** | NA | NA | 1.24E+09 | 1.10E+09 | 5.69E+06 | 7.25E+06 | NA | NA | NA | NA |
| **445** | NA | NA | 1.28E+09 | 1.05E+09 | 6.97E+06 | 2.46E+06 | NA | NA | NA | NA |
| **446** | 1.98E+07 | 5.80E+06 | 1.20E+09 | 1.13E+09 | 3.46E+06 | 5.65E+06 | 1.66E-02 | 5.74E+00 | 5.11E-03 | 1.03E+00 |
| **447** | NA | NA | 1.23E+09 | 1.10E+09 | 4.05E+06 | 1.98E+06 | NA | NA | NA | NA |
| **448** | 1.40E+06 | 0.00E+00 | 1.09E+09 | 1.24E+09 | 6.53E+06 | 3.74E+06 | 1.28E-03 | 2.14E-01 | 0.00E+00 | 0.00E+00 |
| **449** | NA | NA | 1.11E+09 | 1.21E+09 | 8.57E+06 | 2.88E+06 | NA | NA | NA | NA |
| **450** | 3.04E+06 | 0.00E+00 | 1.10E+09 | 1.22E+09 | 8.42E+06 | 3.22E+06 | 2.77E-03 | 3.61E-01 | 0.00E+00 | 0.00E+00 |
| **451** | 0.00E+00 | 1.25E+07 | 1.22E+09 | 1.10E+09 | 2.00E+06 | 1.25E+06 | 0.00E+00 | 0.00E+00 | 1.13E-02 | 9.97E+00 |
| **452** | 0.00E+00 | 1.25E+07 | 1.22E+09 | 1.10E+09 | 2.00E+06 | 1.25E+06 | 0.00E+00 | 0.00E+00 | 1.13E-02 | 9.97E+00 |

| | | | | | | | | | | |
|---|---|---|---|---|---|---|---|---|---|---|
| **453** | NA | NA | 1.32E+09 | 9.96E+08 | 4.64E+06 | 3.75E+06 | NA | NA | NA | NA |
| **454** | NA | NA | 1.17E+09 | 1.13E+09 | 1.91E+06 | 2.27E+06 | NA | NA | NA | NA |
| **455** | NA | NA | 1.27E+09 | 1.03E+09 | 2.88E+06 | 2.28E+06 | NA | NA | NA | NA |
| **456** | NA | NA | 1.24E+09 | 1.05E+09 | 2.35E+06 | 1.74E+06 | NA | NA | NA | NA |
| **457** | NA | NA | 1.21E+09 | 1.07E+09 | 2.91E+06 | 1.95E+06 | NA | NA | NA | NA |
| **458** | NA | NA | 1.14E+09 | 1.14E+09 | 7.06E+05 | 2.10E+06 | NA | NA | NA | NA |
| **459** | 3.14E+05 | 0.00E+00 | 1.19E+09 | 1.07E+09 | 3.85E+06 | 2.54E+06 | 2.64E-04 | 8.16E-02 | 0.00E+00 | 0.00E+00 |
| **460** | 5.44E+06 | 1.09E+07 | 1.25E+09 | 1.02E+09 | 2.08E+06 | 0.00E+00 | 4.36E-03 | 2.62E+00 | 1.07E-02 | Inf |
| **461** | 1.84E+06 | 0.00E+00 | 1.11E+09 | 1.15E+09 | 1.90E+06 | 1.24E+06 | 1.66E-03 | 9.67E-01 | 0.00E+00 | 0.00E+00 |
| **462** | NA | NA | 1.17E+09 | 1.09E+09 | 3.08E+06 | 7.97E+05 | NA | NA | NA | NA |
| **463** | NA | NA | 9.14E+08 | 1.34E+09 | 4.46E+06 | 2.85E+06 | NA | NA | NA | NA |
| **464** | 2.28E+05 | 5.25E+07 | 1.34E+09 | 9.12E+08 | 2.12E+06 | 1.52E+06 | 1.70E-04 | 1.08E-01 | 5.75E-02 | 3.45E+01 |
| **465** | 2.28E+05 | 5.25E+07 | 1.34E+09 | 9.12E+08 | 2.12E+06 | 1.52E+06 | 1.70E-04 | 1.08E-01 | 5.75E-02 | 3.45E+01 |
| **466** | NA | NA | 1.11E+09 | 1.11E+09 | 8.74E+06 | 2.51E+06 | NA | NA | NA | NA |
| **467** | NA | NA | 1.34E+09 | 8.86E+08 | 2.86E+06 | 2.91E+06 | NA | NA | NA | NA |
| **468** | 0.00E+00 | 3.87E+05 | 1.32E+09 | 8.94E+08 | 1.59E+06 | 1.73E+06 | 0.00E+00 | 0.00E+00 | 4.33E-04 | 2.24E-01 |
| **469** | 1.09E+06 | 0.00E+00 | 1.04E+09 | 1.14E+09 | 2.53E+06 | 7.55E+06 | 1.05E-03 | 4.31E-01 | 0.00E+00 | 0.00E+00 |
| **470** | NA | NA | 1.32E+09 | 8.55E+08 | 5.20E+06 | 6.85E+06 | NA | NA | NA | NA |
| **471** | NA | NA | 1.34E+09 | 8.41E+08 | 1.57E+06 | 1.63E+06 | NA | NA | NA | NA |
| **472** | NA | NA | 1.15E+09 | 1.03E+09 | 1.64E+06 | 1.01E+06 | NA | NA | NA | NA |
| **473** | NA | NA | 1.16E+09 | 1.00E+09 | 2.79E+06 | 2.67E+06 | NA | NA | NA | NA |
| **474** | NA | NA | 1.18E+09 | 9.65E+08 | 3.17E+06 | 5.00E+06 | NA | NA | NA | NA |
| **475** | 7.67E+06 | 0.00E+00 | 1.09E+09 | 1.04E+09 | 5.71E+06 | 6.07E+06 | 7.03E-03 | 1.34E+00 | 0.00E+00 | 0.00E+00 |
| **476** | NA | NA | 9.74E+08 | 1.15E+09 | 5.80E+06 | 4.53E+06 | NA | NA | NA | NA |
| **477** | 1.04E+06 | 0.00E+00 | 1.04E+09 | 1.07E+09 | 3.63E+06 | 2.60E+06 | 9.98E-04 | 2.86E-01 | 0.00E+00 | 0.00E+00 |
| **478** | NA | NA | 1.01E+09 | 1.09E+09 | 3.89E+06 | 2.87E+06 | NA | NA | NA | NA |
| **479** | NA | NA | 1.11E+09 | 9.90E+08 | 2.05E+06 | 3.41E+05 | NA | NA | NA | NA |
| **480** | NA | NA | 1.12E+09 | 9.76E+08 | 5.33E+06 | 1.36E+06 | NA | NA | NA | NA |
| **481** | NA | NA | 1.05E+09 | 1.04E+09 | 6.57E+06 | 2.82E+06 | NA | NA | NA | NA |
| **482** | NA | NA | 1.02E+09 | 1.06E+09 | 5.04E+06 | 6.78E+06 | NA | NA | NA | NA |
| **483** | NA | NA | 9.84E+08 | 1.10E+09 | 4.82E+06 | 2.93E+06 | NA | NA | NA | NA |
| **484** | 8.68E+06 | 3.86E+06 | 9.17E+08 | 1.15E+09 | 3.93E+06 | 7.78E+06 | 9.46E-03 | 2.21E+00 | 3.34E-03 | 4.96E-01 |
| **485** | NA | NA | 1.14E+09 | 9.30E+08 | 1.75E+06 | 9.86E+05 | NA | NA | NA | NA |
| **486** | NA | NA | 1.05E+09 | 9.97E+08 | 5.05E+06 | 8.63E+06 | NA | NA | NA | NA |
| **487** | NA | NA | 9.98E+08 | 1.05E+09 | 1.90E+06 | 1.09E+06 | NA | NA | NA | NA |
| **488** | NA | NA | 1.05E+09 | 9.85E+08 | 4.38E+06 | 4.97E+06 | NA | NA | NA | NA |
| **489** | 4.61E+06 | 1.12E+07 | 9.87E+08 | 1.05E+09 | 7.31E+06 | 4.50E+06 | 4.67E-03 | 6.30E-01 | 1.07E-02 | 2.48E+00 |
| **490** | NA | NA | 1.10E+09 | 9.34E+08 | 3.57E+06 | 2.14E+06 | NA | NA | NA | NA |

| | | | | | | | | | | |
|---|---|---|---|---|---|---|---|---|---|---|
| **491** | NA | NA | 9.84E+08 | 1.03E+09 | 3.31E+06 | 3.79E+06 | NA | NA | NA | NA |
| **492** | NA | NA | 1.02E+09 | 1.00E+09 | 4.15E+06 | 2.98E+06 | NA | NA | NA | NA |
| **493** | NA | NA | 9.88E+08 | 1.03E+09 | 1.48E+06 | 1.23E+06 | NA | NA | NA | NA |
| **494** | 3.11E+07 | 2.47E+07 | 1.02E+09 | 9.77E+08 | 4.43E+06 | 6.32E+06 | 3.04E-02 | 7.01E+00 | 2.53E-02 | 3.91E+00 |
| **495** | 5.87E+06 | 4.01E+07 | 1.00E+09 | 9.93E+08 | 3.16E+06 | 2.37E+06 | 5.85E-03 | 1.86E+00 | 4.04E-02 | 1.69E+01 |
| **496** | NA | NA | 1.08E+09 | 9.06E+08 | 1.36E+06 | 1.68E+06 | NA | NA | NA | NA |
| **497** | NA | NA | 1.14E+09 | 8.36E+08 | 4.66E+06 | 2.73E+06 | NA | NA | NA | NA |
| **498** | 3.04E+06 | 0.00E+00 | 1.06E+09 | 9.03E+08 | 2.61E+06 | 2.67E+06 | 2.86E-03 | 1.17E+00 | 0.00E+00 | 0.00E+00 |
| **499** | NA | NA | 8.02E+08 | 1.13E+09 | 8.17E+06 | 6.13E+06 | NA | NA | NA | NA |
| **500** | NA | NA | 7.82E+08 | 1.15E+09 | 2.69E+06 | 1.83E+06 | NA | NA | NA | NA |
| **501** | NA | NA | 9.15E+08 | 9.96E+08 | 2.18E+06 | 2.53E+06 | NA | NA | NA | NA |
| **502** | NA | NA | 9.42E+08 | 9.68E+08 | 2.84E+06 | 1.84E+06 | NA | NA | NA | NA |
| **503** | NA | NA | 1.06E+09 | 8.42E+08 | 3.55E+06 | 2.61E+06 | NA | NA | NA | NA |
| **504** | NA | NA | 1.43E+09 | 4.60E+08 | 3.31E+06 | 1.82E+06 | NA | NA | NA | NA |
| **505** | NA | NA | 9.22E+08 | 9.57E+08 | 3.85E+06 | 3.84E+06 | NA | NA | NA | NA |
| **506** | NA | NA | 9.72E+08 | 8.98E+08 | 2.37E+06 | 1.83E+06 | NA | NA | NA | NA |
| **507** | NA | NA | 1.30E+09 | 5.26E+08 | 2.52E+07 | 1.90E+07 | NA | NA | NA | NA |
| **508** | NA | NA | 1.06E+09 | 8.03E+08 | 2.97E+06 | 2.81E+06 | NA | NA | NA | NA |
| **509** | 1.04E+06 | 0.00E+00 | 8.52E+08 | 9.85E+08 | 4.61E+06 | 1.11E+07 | 1.22E-03 | 2.26E-01 | 0.00E+00 | 0.00E+00 |
| **510** | NA | NA | 9.88E+08 | 8.57E+08 | 1.85E+06 | 1.42E+06 | NA | NA | NA | NA |
| **511** | NA | NA | 1.10E+09 | 7.45E+08 | 1.53E+06 | 1.63E+06 | NA | NA | NA | NA |
| **512** | NA | NA | 7.28E+08 | 1.10E+09 | 2.46E+06 | 6.15E+06 | NA | NA | NA | NA |
| **513** | NA | NA | 9.63E+08 | 8.63E+08 | 2.43E+06 | 2.58E+06 | NA | NA | NA | NA |
| **514** | NA | NA | 8.38E+08 | 9.40E+08 | 1.55E+07 | 1.56E+07 | NA | NA | NA | NA |
| **515** | 3.07E+05 | 0.00E+00 | 9.00E+08 | 9.01E+08 | 1.25E+06 | 2.09E+06 | 3.41E-04 | 2.46E-01 | 0.00E+00 | 0.00E+00 |
| **516** | NA | NA | 8.58E+08 | 9.30E+08 | 4.36E+06 | 1.32E+06 | NA | NA | NA | NA |
| **517** | NA | NA | 8.75E+08 | 9.01E+08 | 8.49E+06 | 7.23E+06 | NA | NA | NA | NA |
| **518** | NA | NA | 8.65E+08 | 9.09E+08 | 2.77E+06 | 2.91E+06 | NA | NA | NA | NA |
| **519** | NA | NA | 8.27E+08 | 9.38E+08 | 6.55E+05 | 0.00E+00 | NA | NA | NA | NA |
| **520** | NA | NA | 7.80E+08 | 9.81E+08 | 2.23E+06 | 1.13E+06 | NA | NA | NA | NA |
| **521** | NA | NA | 9.78E+08 | 7.69E+08 | 2.39E+06 | 3.03E+06 | NA | NA | NA | NA |
| **522** | NA | NA | 8.37E+08 | 8.98E+08 | 4.08E+06 | 1.41E+06 | NA | NA | NA | NA |
| **523** | 7.94E+05 | 2.90E+05 | 9.08E+08 | 8.17E+08 | 3.29E+06 | 1.78E+06 | 8.74E-04 | 2.41E-01 | 3.54E-04 | 1.62E-01 |
| **524** | NA | NA | 8.89E+08 | 8.37E+08 | 6.66E+05 | 1.39E+06 | NA | NA | NA | NA |
| **525** | NA | NA | 9.42E+08 | 7.75E+08 | 2.14E+06 | 2.16E+06 | NA | NA | NA | NA |
| **526** | 2.97E+07 | 8.79E+05 | 8.73E+08 | 8.24E+08 | 6.32E+06 | 6.97E+06 | 3.41E-02 | 4.70E+00 | 1.07E-03 | 1.26E-01 |
| **527** | NA | NA | 9.18E+08 | 7.83E+08 | 2.16E+06 | 5.34E+06 | NA | NA | NA | NA |
| **528** | NA | NA | 8.88E+08 | 8.14E+08 | 2.63E+06 | 1.05E+06 | NA | NA | NA | NA |

| | | | | | | | | | | |
|---|---|---|---|---|---|---|---|---|---|---|
| **529** | 4.95E+05 | 0.00E+00 | 8.81E+08 | 8.03E+08 | 9.20E+06 | 9.53E+06 | 5.62E-04 | 5.38E-02 | 0.00E+00 | 0.00E+00 |
| **530** | NA | NA | 9.00E+08 | 7.87E+08 | 5.23E+06 | 3.79E+06 | NA | NA | NA | NA |
| **531** | NA | NA | 7.79E+08 | 9.09E+08 | 3.49E+06 | 3.79E+06 | NA | NA | NA | NA |
| **532** | NA | NA | 9.17E+08 | 7.69E+08 | 1.63E+06 | 1.02E+06 | NA | NA | NA | NA |
| **533** | NA | NA | 8.00E+08 | 8.78E+08 | 3.40E+06 | 2.57E+06 | NA | NA | NA | NA |
| **534** | NA | NA | 8.61E+08 | 8.18E+08 | 2.68E+06 | 2.16E+06 | NA | NA | NA | NA |
| **535** | 0.00E+00 | 2.03E+05 | 7.74E+08 | 8.95E+08 | 2.35E+06 | 2.38E+06 | 0.00E+00 | 0.00E+00 | 2.27E-04 | 8.54E-02 |
| **536** | NA | NA | 8.27E+08 | 8.34E+08 | 5.51E+06 | 1.49E+06 | NA | NA | NA | NA |
| **537** | NA | NA | 8.49E+08 | 8.16E+08 | 1.12E+06 | 3.75E+05 | NA | NA | NA | NA |
| **538** | NA | NA | 8.11E+08 | 8.52E+08 | 2.11E+06 | 3.55E+05 | NA | NA | NA | NA |
| **539** | NA | NA | 9.06E+08 | 7.54E+08 | 2.10E+06 | 1.18E+06 | NA | NA | NA | NA |
| **540** | NA | NA | 8.15E+08 | 8.43E+08 | 1.58E+06 | 2.24E+06 | NA | NA | NA | NA |
| **541** | NA | NA | 1.10E+09 | 5.57E+08 | 0.00E+00 | 1.57E+05 | NA | NA | NA | NA |
| **542** | NA | NA | 8.91E+08 | 7.61E+08 | 2.70E+06 | 2.19E+06 | NA | NA | NA | NA |
| **543** | 5.11E+06 | 0.00E+00 | 8.09E+08 | 8.44E+08 | 1.30E+06 | 2.64E+06 | 6.32E-03 | 3.94E+00 | 0.00E+00 | 0.00E+00 |
| **544** | 1.11E+07 | 0.00E+00 | 8.41E+08 | 8.06E+08 | 3.98E+06 | 1.71E+06 | 1.32E-02 | 2.80E+00 | 0.00E+00 | 0.00E+00 |
| **545** | NA | NA | 6.76E+08 | 9.69E+08 | NA | NA | NA | NA | NA | NA |
| **546** | NA | NA | 7.58E+08 | 8.80E+08 | 2.58E+06 | 8.07E+05 | NA | NA | NA | NA |
| **547** | NA | NA | 9.13E+08 | 7.19E+08 | 5.26E+06 | 2.65E+06 | NA | NA | NA | NA |
| **548** | NA | NA | 7.92E+08 | 8.37E+08 | 4.01E+06 | 2.98E+06 | NA | NA | NA | NA |
| **549** | NA | NA | 1.20E+09 | 4.24E+08 | 5.35E+06 | 6.30E+06 | NA | NA | NA | NA |
| **550** | NA | NA | 7.43E+08 | 8.88E+08 | 1.44E+06 | 2.00E+06 | NA | NA | NA | NA |
| **551** | 6.44E+05 | 0.00E+00 | 8.52E+08 | 7.70E+08 | 2.49E+06 | 2.65E+06 | 7.57E-04 | 2.59E-01 | 0.00E+00 | 0.00E+00 |
| **552** | NA | NA | 7.22E+08 | 9.01E+08 | 1.56E+06 | 7.60E+05 | NA | NA | NA | NA |
| **553** | 4.16E+05 | 0.00E+00 | 7.84E+08 | 8.34E+08 | 8.85E+05 | 1.98E+06 | 5.31E-04 | 4.71E-01 | 0.00E+00 | 0.00E+00 |
| **554** | NA | NA | 9.10E+08 | 7.06E+08 | 3.19E+06 | 8.06E+05 | NA | NA | NA | NA |
| **555** | NA | NA | 9.36E+08 | 6.76E+08 | 1.48E+06 | 1.25E+06 | NA | NA | NA | NA |
| **556** | NA | NA | 9.38E+08 | 6.69E+08 | 1.88E+06 | 0.00E+00 | NA | NA | NA | NA |
| **557** | NA | NA | 7.57E+08 | 8.44E+08 | 2.45E+06 | 8.30E+05 | NA | NA | NA | NA |
| **558** | NA | NA | 7.53E+08 | 8.37E+08 | 2.98E+06 | 1.98E+06 | NA | NA | NA | NA |
| **559** | NA | NA | 8.29E+08 | 7.62E+08 | 1.37E+06 | 2.60E+06 | NA | NA | NA | NA |
| **560** | NA | NA | 8.26E+08 | 7.61E+08 | 3.36E+06 | 1.16E+06 | NA | NA | NA | NA |
| **561** | NA | NA | 8.17E+08 | 7.65E+08 | 3.65E+06 | 1.14E+06 | NA | NA | NA | NA |
| **562** | NA | NA | 7.30E+08 | 8.49E+08 | 3.98E+06 | 3.53E+05 | NA | NA | NA | NA |
| **563** | NA | NA | 7.12E+08 | 8.65E+08 | 1.08E+06 | 1.07E+06 | NA | NA | NA | NA |
| **564** | NA | NA | 8.37E+08 | 7.38E+08 | 2.82E+06 | 6.02E+05 | NA | NA | NA | NA |
| **565** | NA | NA | 7.82E+08 | 7.81E+08 | 1.44E+06 | 2.69E+06 | NA | NA | NA | NA |
| **566** | NA | NA | 7.27E+08 | 8.21E+08 | 1.50E+06 | 7.83E+05 | NA | NA | NA | NA |

| | | | | | | | | | | |
|---|---|---|---|---|---|---|---|---|---|---|
| **567** | NA | NA | 7.12E+08 | 8.09E+08 | 7.68E+06 | 8.03E+06 | NA | NA | NA | NA |
| **568** | NA | NA | 7.82E+08 | 7.40E+08 | 7.62E+06 | 3.36E+06 | NA | NA | NA | NA |
| **569** | NA | NA | 7.89E+08 | 7.39E+08 | 2.03E+06 | 1.72E+06 | NA | NA | NA | NA |
| **570** | 2.22E+06 | 0.00E+00 | 8.01E+08 | 7.21E+08 | 5.81E+06 | 2.41E+06 | 2.77E-03 | 3.82E-01 | 0.00E+00 | 0.00E+00 |
| **571** | NA | NA | 8.63E+08 | 6.59E+08 | 1.07E+06 | 1.03E+06 | NA | NA | NA | NA |
| **572** | NA | NA | 7.60E+08 | 7.31E+08 | 1.08E+07 | 6.42E+06 | NA | NA | NA | NA |
| **573** | NA | NA | 5.79E+08 | 9.17E+08 | 3.95E+06 | 5.05E+06 | NA | NA | NA | NA |
| **574** | NA | NA | 6.70E+08 | 8.30E+08 | 3.72E+06 | 1.58E+06 | NA | NA | NA | NA |
| **575** | NA | NA | 6.86E+08 | 7.96E+08 | 4.82E+06 | 2.26E+06 | NA | NA | NA | NA |
| **576** | NA | NA | 6.74E+08 | 8.10E+08 | 1.34E+06 | 2.63E+06 | NA | NA | NA | NA |
| **577** | NA | NA | 7.36E+08 | 7.47E+08 | 1.44E+06 | 2.04E+06 | NA | NA | NA | NA |
| **578** | NA | NA | 7.27E+08 | 7.51E+08 | 3.72E+06 | 3.87E+06 | NA | NA | NA | NA |
| **579** | NA | NA | 7.06E+08 | 7.68E+08 | 1.80E+06 | 1.39E+06 | NA | NA | NA | NA |
| **580** | NA | NA | 7.02E+08 | 7.64E+08 | 3.14E+06 | 2.69E+06 | NA | NA | NA | NA |
| **581** | NA | NA | 7.39E+08 | 7.23E+08 | 1.52E+06 | 2.41E+06 | NA | NA | NA | NA |
| **582** | NA | NA | 6.19E+08 | 8.41E+08 | 1.34E+06 | 1.13E+06 | NA | NA | NA | NA |
| **583** | 2.23E+07 | 4.76E+05 | 6.40E+08 | 8.07E+08 | 7.61E+06 | 6.34E+06 | 3.48E-02 | 2.93E+00 | 5.90E-04 | 7.51E-02 |
| **584** | NA | NA | 8.83E+08 | 5.70E+08 | 3.19E+06 | 2.23E+06 | NA | NA | NA | NA |
| **585** | NA | NA | 9.35E+08 | 5.17E+08 | 2.18E+06 | 1.60E+06 | NA | NA | NA | NA |
| **586** | NA | NA | 7.37E+08 | 7.06E+08 | 2.69E+06 | 1.22E+06 | NA | NA | NA | NA |
| **587** | NA | NA | 7.63E+08 | 6.79E+08 | 1.09E+06 | 9.75E+05 | NA | NA | NA | NA |
| **588** | NA | NA | 8.07E+08 | 6.23E+08 | 3.14E+05 | 2.88E+05 | NA | NA | NA | NA |
| **589** | NA | NA | 7.26E+08 | 7.01E+08 | 1.97E+06 | 1.89E+06 | NA | NA | NA | NA |
| **590** | NA | NA | 7.47E+08 | 6.78E+08 | 6.71E+05 | 4.76E+05 | NA | NA | NA | NA |
| **591** | NA | NA | 7.77E+08 | 6.41E+08 | 9.46E+05 | 1.85E+06 | NA | NA | NA | NA |
| **592** | NA | NA | 6.93E+08 | 7.18E+08 | 1.50E+06 | 2.25E+06 | NA | NA | NA | NA |
| **593** | NA | NA | 7.77E+08 | 6.25E+08 | 1.22E+06 | 3.62E+06 | NA | NA | NA | NA |
| **594** | NA | NA | 7.65E+08 | 6.33E+08 | 1.41E+06 | 1.02E+06 | NA | NA | NA | NA |
| **595** | NA | NA | 6.54E+08 | 7.39E+08 | 3.72E+06 | 2.03E+06 | NA | NA | NA | NA |
| **596** | NA | NA | 6.29E+08 | 7.61E+08 | 2.66E+06 | 0.00E+00 | NA | NA | NA | NA |
| **597** | NA | NA | 7.03E+08 | 6.80E+08 | 2.05E+06 | 8.20E+05 | NA | NA | NA | NA |
| **598** | 5.68E+07 | 4.01E+07 | 6.45E+08 | 7.30E+08 | 3.93E+06 | 2.80E+06 | 8.81E-02 | 1.44E+01 | 5.50E-02 | 1.43E+01 |
| **599** | NA | NA | 7.31E+08 | 6.43E+08 | 1.56E+06 | 3.62E+06 | NA | NA | NA | NA |
| **600** | NA | NA | 7.48E+08 | 6.27E+08 | 2.37E+06 | 4.62E+05 | NA | NA | NA | NA |
| **601** | NA | NA | 8.08E+08 | 5.55E+08 | 5.06E+06 | 3.55E+06 | NA | NA | NA | NA |
| **602** | NA | NA | 6.40E+08 | 7.23E+08 | 5.81E+06 | 1.80E+06 | NA | NA | NA | NA |
| **603** | 1.52E+07 | 0.00E+00 | 7.36E+08 | 6.27E+08 | 2.80E+06 | 4.25E+06 | 2.06E-02 | 5.42E+00 | 0.00E+00 | 0.00E+00 |
| **604** | NA | NA | 6.39E+08 | 7.26E+08 | 1.48E+06 | 3.55E+06 | NA | NA | NA | NA |

| | | | | | | | | | | |
|---|---|---|---|---|---|---|---|---|---|---|
| **605** | 1.68E+07 | 2.18E+08 | 7.87E+08 | 5.81E+08 | 2.03E+06 | 3.21E+05 | 2.13E-02 | 8.28E+00 | 3.75E-01 | 6.79E+02 |
| **606** | NA | NA | 8.32E+08 | 5.29E+08 | 1.58E+06 | 3.21E+06 | NA | NA | NA | NA |
| **607** | NA | NA | 7.17E+08 | 6.27E+08 | 1.24E+07 | 6.92E+06 | NA | NA | NA | NA |
| **608** | NA | NA | 7.09E+08 | 6.40E+08 | 3.01E+06 | 8.89E+05 | NA | NA | NA | NA |
| **609** | NA | NA | 6.62E+08 | 6.83E+08 | 4.00E+06 | 1.04E+06 | NA | NA | NA | NA |
| **610** | NA | NA | 6.62E+08 | 6.85E+08 | 1.15E+06 | 1.11E+06 | NA | NA | NA | NA |
| **611** | NA | NA | 6.33E+08 | 7.11E+08 | 1.47E+06 | 2.02E+06 | NA | NA | NA | NA |
| **612** | NA | NA | 6.40E+08 | 7.03E+08 | NA | NA | NA | NA | NA | NA |
| **613** | NA | NA | 6.55E+08 | 6.82E+08 | 2.06E+06 | 2.33E+06 | NA | NA | NA | NA |
| **614** | NA | NA | 7.77E+08 | 5.60E+08 | 1.69E+06 | 1.34E+06 | NA | NA | NA | NA |
| **615** | NA | NA | 8.28E+08 | 5.07E+08 | 2.93E+06 | 1.04E+06 | NA | NA | NA | NA |
| **616** | NA | NA | 6.13E+08 | 7.12E+08 | 1.54E+06 | 2.21E+06 | NA | NA | NA | NA |
| **617** | 2.01E+05 | 0.00E+00 | 7.27E+08 | 5.96E+08 | 1.68E+06 | 1.08E+06 | 2.76E-04 | 1.20E-01 | 0.00E+00 | 0.00E+00 |
| **618** | 1.55E+08 | 6.21E+06 | 5.30E+08 | 7.77E+08 | 1.14E+06 | 1.53E+07 | 2.92E-01 | 1.35E+02 | 7.99E-03 | 4.05E-01 |
| **619** | NA | NA | 6.41E+08 | 6.75E+08 | 3.26E+06 | 2.25E+06 | NA | NA | NA | NA |
| **620** | NA | NA | 7.51E+08 | 5.65E+08 | 2.27E+06 | 1.76E+06 | NA | NA | NA | NA |
| **621** | NA | NA | 6.81E+08 | 6.35E+08 | 1.38E+06 | 4.87E+05 | NA | NA | NA | NA |
| **622** | NA | NA | 7.89E+08 | 5.24E+08 | 9.98E+05 | 1.42E+06 | NA | NA | NA | NA |
| **623** | NA | NA | 6.59E+08 | 6.50E+08 | 2.97E+06 | 1.99E+06 | NA | NA | NA | NA |
| **624** | NA | NA | 5.43E+08 | 7.69E+08 | 3.13E+05 | 1.33E+06 | NA | NA | NA | NA |
| **625** | NA | NA | 7.21E+08 | 5.82E+08 | 1.25E+06 | 4.23E+05 | NA | NA | NA | NA |
| **626** | NA | NA | 6.28E+08 | 6.61E+08 | 2.25E+06 | 3.93E+06 | NA | NA | NA | NA |
| **627** | NA | NA | 5.37E+08 | 7.45E+08 | 5.35E+06 | 4.88E+06 | NA | NA | NA | NA |
| **628** | NA | NA | 5.51E+08 | 7.25E+08 | 1.56E+07 | 3.19E+05 | NA | NA | NA | NA |
| **629** | 7.86E+05 | 6.33E+05 | 6.85E+08 | 6.00E+08 | 3.02E+06 | 4.05E+06 | 1.15E-03 | 2.60E-01 | 1.06E-03 | 1.56E-01 |
| **630** | NA | NA | 6.74E+08 | 6.05E+08 | 2.20E+06 | 8.43E+06 | NA | NA | NA | NA |
| **631** | NA | NA | 7.43E+08 | 5.45E+08 | 1.45E+06 | 8.63E+05 | NA | NA | NA | NA |
| **632** | NA | NA | 6.44E+08 | 6.42E+08 | 1.32E+06 | 1.88E+06 | NA | NA | NA | NA |
| **633** | NA | NA | 6.84E+08 | 6.03E+08 | 7.69E+05 | 4.00E+05 | NA | NA | NA | NA |
| **634** | NA | NA | 7.43E+08 | 5.32E+08 | 1.66E+06 | 1.34E+06 | NA | NA | NA | NA |
| **635** | NA | NA | 6.03E+08 | 6.70E+08 | 1.44E+06 | 1.42E+06 | NA | NA | NA | NA |
| **636** | NA | NA | 7.65E+08 | 5.05E+08 | 1.66E+06 | 1.41E+06 | NA | NA | NA | NA |
| **637** | NA | NA | 5.72E+08 | 6.98E+08 | 7.63E+05 | 1.46E+06 | NA | NA | NA | NA |
| **638** | NA | NA | 7.31E+08 | 5.38E+08 | 3.21E+05 | 9.89E+05 | NA | NA | NA | NA |
| **639** | NA | NA | 6.58E+08 | 6.02E+08 | 2.89E+06 | 3.16E+06 | NA | NA | NA | NA |
| **640** | NA | NA | 7.12E+08 | 5.49E+08 | 7.83E+05 | 7.71E+05 | NA | NA | NA | NA |
| **641** | NA | NA | 5.46E+08 | 6.94E+08 | 2.52E+06 | 1.67E+06 | NA | NA | NA | NA |
| **642** | NA | NA | 6.53E+08 | 5.85E+08 | 3.74E+06 | 1.28E+06 | NA | NA | NA | NA |

| | | | | | | | | | | |
|---|---|---|---|---|---|---|---|---|---|---|
| **643** | NA | NA | 6.74E+08 | 5.59E+08 | 3.22E+06 | 2.77E+06 | NA | NA | NA | NA |
| **644** | NA | NA | 7.39E+08 | 4.91E+08 | 6.11E+06 | 2.54E+06 | NA | NA | NA | NA |
| **645** | NA | NA | 8.48E+08 | 3.79E+08 | 3.12E+06 | 7.66E+05 | NA | NA | NA | NA |
| **646** | NA | NA | 6.68E+08 | 5.54E+08 | 3.94E+06 | 3.78E+06 | NA | NA | NA | NA |
| **647** | NA | NA | 6.53E+08 | 5.71E+08 | 1.49E+06 | 9.93E+05 | NA | NA | NA | NA |
| **648** | 1.26E+06 | 0.00E+00 | 6.86E+08 | 5.36E+08 | 1.17E+06 | 1.65E+06 | 1.83E-03 | 1.07E+00 | 0.00E+00 | 0.00E+00 |
| **649** | NA | NA | 6.06E+08 | 6.09E+08 | 2.29E+06 | 2.77E+06 | NA | NA | NA | NA |
| **650** | NA | NA | 3.91E+08 | 8.27E+08 | NA | NA | NA | NA | NA | NA |
| **651** | 8.41E+05 | 0.00E+00 | 6.18E+08 | 5.92E+08 | 2.68E+06 | 2.59E+06 | 1.36E-03 | 3.14E-01 | 0.00E+00 | 0.00E+00 |
| **652** | NA | NA | 5.81E+08 | 6.27E+08 | 1.87E+06 | 1.06E+06 | NA | NA | NA | NA |
| **653** | NA | NA | 6.13E+08 | 5.85E+08 | 2.68E+06 | 4.53E+06 | NA | NA | NA | NA |
| **654** | 6.36E+05 | 0.00E+00 | 6.01E+08 | 6.01E+08 | 1.76E+06 | 1.54E+06 | 1.06E-03 | 3.62E-01 | 0.00E+00 | 0.00E+00 |
| **655** | NA | NA | 5.79E+08 | 5.92E+08 | 8.89E+06 | 2.35E+07 | NA | NA | NA | NA |
| **656** | NA | NA | 6.27E+08 | 5.72E+08 | 3.57E+06 | 7.11E+05 | NA | NA | NA | NA |
| **657** | NA | NA | 5.80E+08 | 6.17E+08 | 4.38E+06 | 1.51E+06 | NA | NA | NA | NA |
| **658** | 6.47E+07 | 3.70E+07 | 5.73E+08 | 6.20E+08 | 3.90E+06 | 2.68E+06 | 1.13E-01 | 1.66E+01 | 5.97E-02 | 1.38E+01 |
| **659** | NA | NA | 6.63E+08 | 5.30E+08 | 4.73E+06 | 1.43E+06 | NA | NA | NA | NA |
| **660** | NA | NA | 5.89E+08 | 6.02E+08 | 2.08E+06 | 1.89E+06 | NA | NA | NA | NA |
| **661** | NA | NA | 7.11E+08 | 4.78E+08 | NA | NA | NA | NA | NA | NA |
| **662** | 5.91E+06 | 0.00E+00 | 6.04E+08 | 5.80E+08 | 3.39E+06 | 1.10E+06 | 9.79E-03 | 1.74E+00 | 0.00E+00 | 0.00E+00 |
| **663** | NA | NA | 6.06E+08 | 5.77E+08 | 1.82E+06 | 2.27E+06 | NA | NA | NA | NA |
| **664** | NA | NA | 7.03E+08 | 4.82E+08 | 1.09E+06 | 5.34E+05 | NA | NA | NA | NA |
| **665** | NA | NA | 4.85E+08 | 6.96E+08 | 0.00E+00 | 2.32E+06 | NA | NA | NA | NA |
| **666** | NA | NA | 5.94E+08 | 5.83E+08 | 4.64E+06 | 2.03E+06 | NA | NA | NA | NA |
| **667** | NA | NA | 5.62E+08 | 6.13E+08 | 2.75E+06 | 3.59E+06 | NA | NA | NA | NA |
| **668** | 3.25E+07 | 0.00E+00 | 6.29E+08 | 5.47E+08 | 2.55E+06 | 2.69E+06 | 5.16E-02 | 1.27E+01 | 0.00E+00 | 0.00E+00 |
| **669** | NA | NA | 5.30E+08 | 6.45E+08 | 1.78E+06 | 7.25E+05 | NA | NA | NA | NA |
| **670** | NA | NA | 6.38E+08 | 5.39E+08 | 3.98E+05 | 0.00E+00 | NA | NA | NA | NA |
| **671** | NA | NA | 6.58E+08 | 5.16E+08 | 9.89E+05 | 8.27E+05 | NA | NA | NA | NA |
| **672** | NA | NA | 6.87E+08 | 4.86E+08 | NA | NA | NA | NA | NA | NA |
| **673** | 5.36E+07 | 5.25E+07 | 6.09E+08 | 5.49E+08 | 7.75E+06 | 4.71E+06 | 8.79E-02 | 6.91E+00 | 9.55E-02 | 1.11E+01 |
| **674** | NA | NA | 6.21E+08 | 5.42E+08 | 4.56E+06 | 2.40E+06 | NA | NA | NA | NA |
| **675** | NA | NA | 7.09E+08 | 4.53E+08 | 2.42E+06 | 1.30E+06 | NA | NA | NA | NA |
| **676** | 1.22E+05 | 0.00E+00 | 5.74E+08 | 5.87E+08 | 1.72E+06 | 1.53E+06 | 2.13E-04 | 7.13E-02 | 0.00E+00 | 0.00E+00 |
| **677** | NA | NA | 6.14E+08 | 5.46E+08 | 1.19E+06 | 5.99E+05 | NA | NA | NA | NA |
| **678** | NA | NA | 5.85E+08 | 5.71E+08 | NA | NA | NA | NA | NA | NA |
| **679** | NA | NA | 6.69E+08 | 4.83E+08 | 1.13E+06 | 2.06E+06 | NA | NA | NA | NA |
| **680** | NA | NA | 7.09E+08 | 4.40E+08 | 3.67E+06 | 1.26E+06 | NA | NA | NA | NA |

| | | | | | | | | | | |
|---|---|---|---|---|---|---|---|---|---|---|
| **681** | NA | NA | 5.60E+08 | 5.91E+08 | 1.03E+06 | 0.00E+00 | NA | NA | NA | NA |
| **682** | NA | NA | 5.91E+08 | 5.59E+08 | 6.83E+05 | 5.35E+05 | NA | NA | NA | NA |
| **683** | NA | NA | 5.99E+08 | 5.40E+08 | 1.31E+06 | 3.23E+06 | NA | NA | NA | NA |
| **684** | NA | NA | 6.34E+08 | 5.00E+08 | 3.74E+06 | 2.87E+06 | NA | NA | NA | NA |
| **685** | NA | NA | 6.05E+08 | 5.26E+08 | 1.32E+06 | 1.25E+06 | NA | NA | NA | NA |
| **686** | 1.73E+06 | 0.00E+00 | 5.38E+08 | 5.84E+08 | 4.68E+06 | 1.45E+06 | 3.21E-03 | 3.69E-01 | 0.00E+00 | 0.00E+00 |
| **687** | NA | NA | 5.66E+08 | 5.47E+08 | 6.76E+06 | 3.92E+06 | NA | NA | NA | NA |
| **688** | NA | NA | 6.53E+08 | 4.61E+08 | 2.53E+06 | 5.06E+06 | NA | NA | NA | NA |
| **689** | NA | NA | 4.98E+08 | 6.17E+08 | 1.47E+06 | 2.33E+05 | NA | NA | NA | NA |
| **690** | NA | NA | 6.00E+08 | 5.14E+08 | 2.59E+06 | 7.67E+05 | NA | NA | NA | NA |
| **691** | 9.98E+06 | 0.00E+00 | 4.77E+08 | 6.32E+08 | 1.24E+06 | 3.27E+06 | 2.09E-02 | 8.05E+00 | 0.00E+00 | 0.00E+00 |
| **692** | 0.00E+00 | 5.80E+06 | 5.76E+08 | 5.35E+08 | 6.53E+05 | 1.60E+06 | 0.00E+00 | 0.00E+00 | 1.09E-02 | 3.62E+00 |
| **693** | NA | NA | 5.04E+08 | 5.97E+08 | 7.20E+06 | 3.03E+06 | NA | NA | NA | NA |
| **694** | 2.05E+06 | 0.00E+00 | 5.12E+08 | 5.91E+08 | 3.18E+06 | 3.62E+06 | 4.01E-03 | 6.46E-01 | 0.00E+00 | 0.00E+00 |
| **695** | 1.38E+07 | 0.00E+00 | 4.88E+08 | 6.15E+08 | 2.59E+06 | 3.51E+06 | 2.84E-02 | 5.33E+00 | 0.00E+00 | 0.00E+00 |
| **696** | NA | NA | 6.67E+08 | 4.39E+08 | 1.12E+06 | 1.36E+06 | NA | NA | NA | NA |
| **697** | NA | NA | 5.84E+08 | 5.18E+08 | 2.48E+06 | 0.00E+00 | NA | NA | NA | NA |
| **698** | 7.51E+07 | 0.00E+00 | 4.90E+08 | 6.04E+08 | 8.59E+05 | 8.57E+06 | 1.53E-01 | 8.74E+01 | 0.00E+00 | 0.00E+00 |
| **699** | NA | NA | 5.46E+08 | 5.44E+08 | 2.13E+06 | 2.28E+06 | NA | NA | NA | NA |
| **700** | NA | NA | 5.12E+08 | 5.79E+08 | 8.69E+05 | 7.63E+05 | NA | NA | NA | NA |
| **701** | NA | NA | 5.26E+08 | 5.64E+08 | 6.55E+05 | 1.31E+06 | NA | NA | NA | NA |
| **702** | NA | NA | 5.08E+08 | 5.77E+08 | 2.97E+06 | 6.15E+05 | NA | NA | NA | NA |
| **703** | NA | NA | 6.05E+08 | 4.80E+08 | 5.90E+05 | 4.25E+05 | NA | NA | NA | NA |
| **704** | NA | NA | 4.68E+08 | 6.07E+08 | 6.25E+06 | 2.46E+06 | NA | NA | NA | NA |
| **705** | 1.42E+06 | 0.00E+00 | 5.74E+08 | 5.02E+08 | 1.37E+06 | 5.88E+05 | 2.48E-03 | 1.04E+00 | 0.00E+00 | 0.00E+00 |
| **706** | NA | NA | 5.72E+08 | 4.87E+08 | 3.02E+06 | 1.94E+06 | NA | NA | NA | NA |
| **707** | NA | NA | 5.07E+08 | 5.50E+08 | 3.07E+06 | 2.55E+06 | NA | NA | NA | NA |
| **708** | 3.95E+05 | 0.00E+00 | 6.25E+08 | 4.35E+08 | 1.26E+06 | 7.99E+05 | 6.31E-04 | 3.13E-01 | 0.00E+00 | 0.00E+00 |
| **709** | 3.90E+06 | 3.31E+05 | 4.90E+08 | 5.65E+08 | 3.40E+06 | 2.51E+06 | 7.95E-03 | 1.15E+00 | 5.86E-04 | 1.32E-01 |
| **710** | NA | NA | 5.66E+08 | 4.90E+08 | 3.96E+05 | 1.07E+06 | NA | NA | NA | NA |
| **711** | NA | NA | 4.95E+08 | 5.48E+08 | 3.64E+06 | 1.90E+06 | NA | NA | NA | NA |
| **712** | NA | NA | 6.25E+08 | 4.18E+08 | 4.14E+05 | 0.00E+00 | NA | NA | NA | NA |
| **713** | NA | NA | 5.85E+08 | 4.46E+08 | 2.99E+06 | 2.87E+06 | NA | NA | NA | NA |
| **714** | NA | NA | 4.96E+08 | 5.27E+08 | 7.17E+06 | 5.46E+06 | NA | NA | NA | NA |
| **715** | NA | NA | 5.04E+08 | 5.29E+08 | 2.19E+06 | 1.01E+06 | NA | NA | NA | NA |
| **716** | NA | NA | 5.40E+08 | 4.88E+08 | 2.15E+06 | 2.91E+06 | NA | NA | NA | NA |
| **717** | 8.05E+07 | 4.90E+06 | 5.12E+08 | 5.10E+08 | 2.53E+06 | 5.61E+06 | 1.57E-01 | 3.19E+01 | 9.61E-03 | 8.73E-01 |
| **718** | 8.05E+07 | 4.90E+06 | 5.12E+08 | 5.10E+08 | 2.53E+06 | 5.61E+06 | 1.57E-01 | 3.19E+01 | 9.61E-03 | 8.73E-01 |

| | | | | | | | | | | |
|---|---|---|---|---|---|---|---|---|---|---|
| **719** | 5.50E+06 | 2.52E+08 | 5.37E+08 | 4.87E+08 | 1.46E+06 | 1.11E+06 | 1.03E-02 | 3.77E+00 | 5.18E-01 | 2.26E+02 |
| **720** | 5.50E+06 | 2.52E+08 | 5.37E+08 | 4.87E+08 | 1.46E+06 | 1.11E+06 | 1.03E-02 | 3.77E+00 | 5.18E-01 | 2.26E+02 |
| **721** | NA | NA | 5.54E+08 | 4.60E+08 | 3.73E+06 | 2.96E+06 | NA | NA | NA | NA |
| **722** | NA | NA | 4.88E+08 | 5.22E+08 | 2.33E+06 | 6.79E+05 | NA | NA | NA | NA |
| **723** | 3.16E+06 | 0.00E+00 | 4.61E+08 | 5.43E+08 | 3.93E+06 | 1.58E+06 | 6.86E-03 | 8.04E-01 | 0.00E+00 | 0.00E+00 |
| **724** | NA | NA | 4.66E+08 | 5.42E+08 | 5.47E+05 | 4.86E+05 | NA | NA | NA | NA |
| **725** | 2.62E+06 | 0.00E+00 | 3.77E+08 | 6.22E+08 | 1.62E+06 | 8.44E+06 | 6.94E-03 | 1.61E+00 | 0.00E+00 | 0.00E+00 |
| **726** | NA | NA | 4.95E+08 | 4.96E+08 | 2.90E+06 | 1.32E+06 | NA | NA | NA | NA |
| **727** | NA | NA | 4.76E+08 | 5.10E+08 | 2.01E+06 | 1.71E+06 | NA | NA | NA | NA |
| **728** | 1.49E+06 | 0.00E+00 | 4.70E+08 | 5.16E+08 | 1.65E+06 | 9.98E+05 | 3.17E-03 | 9.01E-01 | 0.00E+00 | 0.00E+00 |
| **729** | NA | NA | 5.23E+08 | 4.62E+08 | 1.86E+06 | 8.57E+05 | NA | NA | NA | NA |
| **730** | NA | NA | 4.29E+08 | 5.52E+08 | 1.77E+06 | 1.54E+06 | NA | NA | NA | NA |
| **731** | 6.92E+06 | 0.00E+00 | 4.27E+08 | 5.47E+08 | 4.32E+06 | 2.42E+06 | 1.62E-02 | 1.60E+00 | 0.00E+00 | 0.00E+00 |
| **732** | NA | NA | 5.91E+08 | 3.83E+08 | 1.88E+06 | 6.00E+05 | NA | NA | NA | NA |
| **733** | NA | NA | 6.26E+08 | 3.40E+08 | NA | NA | NA | NA | NA | NA |
| **734** | NA | NA | 6.03E+08 | 3.52E+08 | 1.01E+06 | 2.16E+06 | NA | NA | NA | NA |
| **735** | 0.00E+00 | 1.46E+07 | 5.20E+08 | 4.31E+08 | 1.93E+06 | 1.72E+06 | 0.00E+00 | 0.00E+00 | 3.38E-02 | 8.49E+00 |
| **736** | 2.02E+06 | 0.00E+00 | 4.38E+08 | 5.02E+08 | 3.51E+06 | 3.79E+06 | 4.62E-03 | 5.75E-01 | 0.00E+00 | 0.00E+00 |
| **737** | NA | NA | 4.15E+08 | 5.27E+08 | 1.18E+06 | 5.34E+05 | NA | NA | NA | NA |
| **738** | NA | NA | 4.32E+08 | 5.07E+08 | 1.33E+06 | 1.96E+06 | NA | NA | NA | NA |
| **739** | NA | NA | 4.50E+08 | 4.84E+08 | 3.64E+06 | 1.39E+06 | NA | NA | NA | NA |
| **740** | 1.77E+06 | 0.00E+00 | 8.81E+07 | 8.43E+08 | 1.49E+06 | 1.15E+06 | 2.01E-02 | 1.19E+00 | 0.00E+00 | 0.00E+00 |
| **741** | 3.38E+06 | 5.27E+06 | 4.40E+08 | 4.89E+08 | 2.71E+06 | 2.66E+05 | 7.68E-03 | 1.25E+00 | 1.08E-02 | 1.99E+01 |
| **742** | 8.21E+06 | 0.00E+00 | 3.66E+08 | 5.59E+08 | 1.06E+06 | 2.79E+06 | 2.24E-02 | 7.74E+00 | 0.00E+00 | 0.00E+00 |
| **743** | 1.65E+08 | 9.05E+07 | NA | NA | 4.48E+08 | 4.81E+08 | NA | 3.68E-01 | NA | 1.88E-01 |
| **744** | 3.52E+07 | 2.17E+07 | 4.06E+08 | 5.06E+08 | 9.06E+06 | 4.29E+06 | 8.67E-02 | 3.88E+00 | 4.30E-02 | 5.07E+00 |
| **745** | NA | NA | 4.18E+08 | 5.06E+08 | 1.59E+06 | 0.00E+00 | NA | NA | NA | NA |
| **746** | NA | NA | 5.26E+08 | 3.95E+08 | 2.14E+05 | 1.33E+06 | NA | NA | NA | NA |
| **747** | 3.37E+07 | 0.00E+00 | 3.74E+08 | 5.29E+08 | 1.72E+06 | 5.08E+06 | 9.00E-02 | 1.96E+01 | 0.00E+00 | 0.00E+00 |
| **748** | NA | NA | 4.10E+08 | 4.98E+08 | 1.06E+06 | 4.83E+05 | NA | NA | NA | NA |
| **749** | NA | NA | 5.27E+08 | 3.81E+08 | 0.00E+00 | 3.34E+05 | NA | NA | NA | NA |
| **750** | 5.09E+06 | 0.00E+00 | 4.97E+08 | 3.86E+08 | 1.19E+07 | 1.20E+07 | 1.02E-02 | 4.27E-01 | 0.00E+00 | 0.00E+00 |
| **751** | NA | NA | 5.82E+08 | 3.15E+08 | 1.15E+06 | 1.72E+06 | NA | NA | NA | NA |
| **752** | NA | NA | 4.34E+08 | 4.51E+08 | 6.14E+06 | 2.77E+06 | NA | NA | NA | NA |
| **753** | NA | NA | 4.45E+08 | 4.45E+08 | 2.15E+06 | 1.28E+06 | NA | NA | NA | NA |
| **754** | NA | NA | 5.04E+08 | 3.83E+08 | 3.67E+06 | 1.58E+06 | NA | NA | NA | NA |
| **755** | NA | NA | 4.70E+08 | 4.17E+08 | 1.62E+06 | 1.40E+06 | NA | NA | NA | NA |
| **756** | 1.80E+06 | 2.96E+07 | 3.86E+08 | 4.99E+08 | 1.58E+06 | 1.01E+06 | 4.66E-03 | 1.14E+00 | 5.94E-02 | 2.95E+01 |

| | | | | | | | | | | |
|---|---|---|---|---|---|---|---|---|---|---|
| **757** | NA | NA | 3.82E+08 | 4.97E+08 | 1.33E+06 | 1.22E+06 | NA | NA | NA | NA |
| **758** | NA | NA | 4.39E+08 | 4.38E+08 | 9.92E+05 | 2.88E+06 | NA | NA | NA | NA |
| **759** | NA | NA | 4.47E+08 | 4.28E+08 | 1.10E+06 | 5.34E+05 | NA | NA | NA | NA |
| **760** | NA | NA | NA | NA | 5.34E+08 | 3.43E+08 | NA | NA | NA | NA |
| **761** | NA | NA | 5.36E+08 | 3.35E+08 | 1.64E+06 | 2.78E+06 | NA | NA | NA | NA |
| **762** | 2.42E+07 | 0.00E+00 | 3.81E+08 | 4.88E+08 | 1.30E+06 | 3.45E+06 | 6.35E-02 | 1.86E+01 | 0.00E+00 | 0.00E+00 |
| **763** | NA | NA | 5.02E+08 | 3.65E+08 | 1.13E+06 | 1.59E+06 | NA | NA | NA | NA |
| **764** | NA | NA | 4.43E+08 | 4.27E+08 | 0.00E+00 | 2.72E+05 | NA | NA | NA | NA |
| **765** | NA | NA | 4.31E+08 | 4.31E+08 | 0.00E+00 | 0.00E+00 | NA | NA | NA | NA |
| **766** | NA | NA | 4.36E+08 | 4.24E+08 | 1.19E+06 | 0.00E+00 | NA | NA | NA | NA |
| **767** | 7.16E+06 | 5.57E+06 | 3.76E+08 | 4.74E+08 | 2.82E+06 | 1.83E+06 | 1.90E-02 | 2.54E+00 | 1.18E-02 | 3.05E+00 |
| **768** | NA | NA | 4.28E+08 | 4.24E+08 | 1.01E+06 | 2.50E+05 | NA | NA | NA | NA |
| **769** | NA | NA | 4.40E+08 | 4.09E+08 | 3.85E+06 | 4.18E+05 | NA | NA | NA | NA |
| **770** | NA | NA | 4.39E+08 | 4.08E+08 | 3.65E+06 | 0.00E+00 | NA | NA | NA | NA |
| **771** | 5.14E+05 | 6.45E+07 | 4.57E+08 | 3.86E+08 | 2.78E+06 | 1.74E+06 | 1.13E-03 | 1.85E-01 | 1.67E-01 | 3.70E+01 |
| **772** | NA | NA | 4.81E+08 | 3.63E+08 | NA | NA | NA | NA | NA | NA |
| **773** | NA | NA | 4.45E+08 | 3.91E+08 | 1.67E+06 | 1.61E+06 | NA | NA | NA | NA |
| **774** | NA | NA | 3.82E+08 | 4.49E+08 | NA | NA | NA | NA | NA | NA |
| **775** | NA | NA | 4.17E+08 | 4.03E+08 | 5.57E+06 | 1.64E+06 | NA | NA | NA | NA |
| **776** | 1.62E+06 | 0.00E+00 | 4.49E+08 | 3.74E+08 | 2.31E+05 | 9.16E+05 | 3.61E-03 | 7.03E+00 | 0.00E+00 | 0.00E+00 |
| **777** | 8.28E+06 | 0.00E+00 | 4.60E+08 | 3.55E+08 | 1.72E+06 | 3.46E+06 | 1.80E-02 | 4.83E+00 | 0.00E+00 | 0.00E+00 |
| **778** | NA | NA | 4.04E+08 | 4.09E+08 | 2.52E+06 | 3.30E+06 | NA | NA | NA | NA |
| **779** | NA | NA | 5.08E+08 | 3.08E+08 | 1.60E+06 | 9.63E+05 | NA | NA | NA | NA |
| **780** | NA | NA | 4.31E+08 | 3.76E+08 | 9.06E+06 | 1.47E+06 | NA | NA | NA | NA |
| **781** | NA | NA | 3.72E+08 | 4.45E+08 | 5.26E+05 | 3.72E+05 | NA | NA | NA | NA |
| **782** | NA | NA | 3.20E+08 | 4.83E+08 | 4.59E+06 | 7.67E+06 | NA | NA | NA | NA |
| **783** | 1.79E+06 | 3.91E+06 | 4.14E+08 | 3.99E+08 | 2.87E+05 | 1.05E+06 | 4.31E-03 | 6.23E+00 | 9.79E-03 | 3.71E+00 |
| **784** | NA | NA | 3.99E+08 | 4.11E+08 | 1.21E+06 | 8.49E+05 | NA | NA | NA | NA |
| **785** | NA | NA | 4.35E+08 | 3.67E+08 | 2.36E+06 | 2.72E+06 | NA | NA | NA | NA |
| **786** | 0.00E+00 | 0.00E+00 | 3.97E+08 | 4.08E+08 | 4.51E+05 | 1.07E+06 | 0.00E+00 | 0.00E+00 | 0.00E+00 | 0.00E+00 |
| **787** | NA | NA | 4.48E+08 | 3.50E+08 | 3.46E+06 | 2.60E+06 | NA | NA | NA | NA |
| **788** | 2.25E+07 | 2.55E+05 | 3.86E+08 | 4.13E+08 | 1.83E+06 | 1.98E+06 | 5.82E-02 | 1.23E+01 | 6.16E-04 | 1.29E-01 |
| **789** | NA | NA | 4.78E+08 | 3.23E+08 | NA | NA | NA | NA | NA | NA |
| **790** | NA | NA | 4.52E+08 | 3.44E+08 | 1.08E+06 | 7.19E+05 | NA | NA | NA | NA |
| **791** | 6.72E+06 | 0.00E+00 | 4.10E+08 | 3.84E+08 | 1.10E+06 | 1.49E+06 | 1.64E-02 | 6.10E+00 | 0.00E+00 | 0.00E+00 |
| **792** | NA | NA | 4.11E+08 | 3.77E+08 | 3.20E+06 | 1.23E+06 | NA | NA | NA | NA |
| **793** | NA | NA | 4.27E+08 | 3.59E+08 | 2.81E+06 | 1.76E+06 | NA | NA | NA | NA |
| **794** | 3.81E+05 | 0.00E+00 | 4.25E+08 | 3.61E+08 | 1.54E+06 | 1.85E+05 | 8.97E-04 | 2.48E-01 | 0.00E+00 | 0.00E+00 |

| | | | | | | | | | | |
|---|---|---|---|---|---|---|---|---|---|---|
| **795** | NA | NA | 2.13E+08 | 5.67E+08 | 4.21E+06 | 2.66E+06 | NA | NA | NA | NA |
| **796** | NA | NA | 3.66E+08 | 4.18E+08 | 5.31E+05 | 1.26E+06 | NA | NA | NA | NA |
| **797** | NA | NA | 4.29E+08 | 3.48E+08 | 1.12E+06 | 0.00E+00 | NA | NA | NA | NA |
| **798** | NA | NA | 3.99E+08 | 3.78E+08 | 0.00E+00 | 0.00E+00 | NA | NA | NA | NA |
| **799** | 8.17E+06 | 0.00E+00 | 2.19E+08 | 5.41E+08 | 6.71E+06 | 5.77E+06 | 3.74E-02 | 1.22E+00 | 0.00E+00 | 0.00E+00 |
| **800** | NA | NA | 4.01E+08 | 3.67E+08 | 1.60E+06 | 1.97E+06 | NA | NA | NA | NA |
| **801** | NA | NA | 4.65E+08 | 3.01E+08 | 0.00E+00 | 6.16E+05 | NA | NA | NA | NA |
| **802** | NA | NA | 3.15E+08 | 4.51E+08 | NA | NA | NA | NA | NA | NA |
| **803** | NA | NA | 3.84E+08 | 3.78E+08 | 1.18E+06 | 1.85E+06 | NA | NA | NA | NA |
| **804** | NA | NA | 3.97E+08 | 3.64E+08 | 8.98E+04 | 0.00E+00 | NA | NA | NA | NA |
| **805** | 0.00E+00 | 1.21E+06 | 4.25E+08 | 3.29E+08 | 5.41E+05 | 3.18E+06 | 0.00E+00 | 0.00E+00 | 3.69E-03 | 3.81E-01 |
| **806** | NA | NA | 4.32E+08 | 3.22E+08 | 7.60E+05 | 4.36E+05 | NA | NA | NA | NA |
| **807** | NA | NA | 3.88E+08 | 3.65E+08 | 2.41E+05 | 5.55E+05 | NA | NA | NA | NA |
| **808** | 2.22E+06 | 0.00E+00 | 4.12E+08 | 3.38E+08 | 1.31E+06 | 1.40E+06 | 5.39E-03 | 1.70E+00 | 0.00E+00 | 0.00E+00 |
| **809** | NA | NA | 4.31E+08 | 3.19E+08 | 1.30E+06 | 0.00E+00 | NA | NA | NA | NA |
| **810** | 0.00E+00 | 1.19E+08 | 4.51E+08 | 2.97E+08 | 1.29E+06 | 1.36E+05 | 0.00E+00 | 0.00E+00 | 4.00E-01 | 8.74E+02 |
| **811** | NA | NA | 3.94E+08 | 3.50E+08 | 2.31E+06 | 2.87E+06 | NA | NA | NA | NA |
| **812** | NA | NA | 3.89E+08 | 3.58E+08 | 1.64E+06 | 7.55E+05 | NA | NA | NA | NA |
| **813** | NA | NA | 3.01E+08 | 4.46E+08 | 9.83E+05 | 5.08E+05 | NA | NA | NA | NA |
| **814** | NA | NA | 4.01E+08 | 3.44E+08 | 6.87E+05 | 6.85E+05 | NA | NA | NA | NA |
| **815** | 3.07E+06 | 0.00E+00 | 4.55E+08 | 2.88E+08 | 2.04E+06 | 7.26E+05 | 6.75E-03 | 1.51E+00 | 0.00E+00 | 0.00E+00 |
| **816** | NA | NA | 4.85E+08 | 2.57E+08 | 1.64E+06 | 1.09E+06 | NA | NA | NA | NA |
| **817** | NA | NA | 2.87E+08 | 4.56E+08 | 1.04E+06 | 1.40E+05 | NA | NA | NA | NA |
| **818** | NA | NA | 4.51E+08 | 2.93E+08 | 8.36E+05 | 0.00E+00 | NA | NA | NA | NA |
| **819** | NA | NA | 4.33E+08 | 3.08E+08 | 0.00E+00 | 3.12E+05 | NA | NA | NA | NA |
| **820** | NA | NA | 4.39E+08 | 3.00E+08 | 1.06E+06 | 1.31E+06 | NA | NA | NA | NA |
| **821** | 2.65E+06 | 0.00E+00 | 3.90E+08 | 3.33E+08 | 6.66E+06 | 1.08E+07 | 6.80E-03 | 3.98E-01 | 0.00E+00 | 0.00E+00 |
| **822** | NA | NA | 3.62E+08 | 3.77E+08 | 9.56E+05 | 5.60E+05 | NA | NA | NA | NA |
| **823** | NA | NA | 4.53E+08 | 2.82E+08 | 8.13E+05 | 9.29E+05 | NA | NA | NA | NA |
| **824** | NA | NA | 4.46E+08 | 2.91E+08 | 1.54E+05 | 0.00E+00 | NA | NA | NA | NA |
| **825** | NA | NA | 4.22E+08 | 3.13E+08 | 0.00E+00 | 7.01E+05 | NA | NA | NA | NA |
| **826** | 8.67E+06 | 0.00E+00 | 3.45E+08 | 3.87E+08 | 6.04E+05 | 1.79E+06 | 2.52E-02 | 1.44E+01 | 0.00E+00 | 0.00E+00 |
| **827** | NA | NA | 3.88E+08 | 3.42E+08 | 1.10E+06 | 1.24E+06 | NA | NA | NA | NA |
| **828** | NA | NA | 4.25E+08 | 3.01E+08 | 1.72E+06 | 3.43E+06 | NA | NA | NA | NA |
| **829** | NA | NA | 3.55E+08 | 3.75E+08 | 4.02E+05 | 7.35E+05 | NA | NA | NA | NA |
| **830** | NA | NA | 2.88E+08 | 4.40E+08 | 4.45E+05 | 1.46E+06 | NA | NA | NA | NA |
| **831** | 2.41E+06 | 0.00E+00 | 4.02E+08 | 3.20E+08 | 3.99E+06 | 2.50E+06 | 5.99E-03 | 6.04E-01 | 0.00E+00 | 0.00E+00 |
| **832** | NA | NA | 3.75E+08 | 3.46E+08 | 1.88E+06 | 2.28E+06 | NA | NA | NA | NA |

| | | | | | | | | | | |
|---|---|---|---|---|---|---|---|---|---|---|
| **833** | NA | NA | 3.42E+08 | 3.64E+08 | 6.62E+06 | 1.01E+07 | NA | NA | NA | NA |
| **834** | NA | NA | 3.42E+08 | 3.64E+08 | 6.62E+06 | 1.01E+07 | NA | NA | NA | NA |
| **835** | NA | NA | 4.26E+08 | 2.88E+08 | 3.40E+06 | 3.89E+06 | NA | NA | NA | NA |
| **836** | NA | NA | 3.95E+08 | 3.24E+08 | 1.27E+06 | 7.53E+05 | NA | NA | NA | NA |
| **837** | 3.06E+06 | 0.00E+00 | 4.16E+08 | 3.01E+08 | 8.66E+05 | 1.58E+06 | 7.36E-03 | 3.53E+00 | 0.00E+00 | 0.00E+00 |
| **838** | NA | NA | 2.22E+08 | 4.93E+08 | 7.64E+05 | 1.57E+05 | NA | NA | NA | NA |
| **839** | NA | NA | 4.38E+08 | 2.78E+08 | NA | NA | NA | NA | NA | NA |
| **840** | NA | NA | 3.79E+08 | 3.31E+08 | 2.87E+05 | 4.57E+05 | NA | NA | NA | NA |
| **841** | NA | NA | 3.96E+08 | 3.12E+08 | 1.50E+06 | 0.00E+00 | NA | NA | NA | NA |
| **842** | NA | NA | 3.13E+08 | 3.92E+08 | 9.04E+05 | 3.05E+06 | NA | NA | NA | NA |
| **843** | NA | NA | 3.57E+08 | 3.49E+08 | NA | NA | NA | NA | NA | NA |
| **844** | NA | NA | 3.62E+08 | 3.42E+08 | 0.00E+00 | 0.00E+00 | NA | NA | NA | NA |
| **845** | NA | NA | 3.14E+08 | 3.89E+08 | 3.09E+04 | 6.44E+05 | NA | NA | NA | NA |
| **846** | NA | NA | 3.59E+08 | 3.36E+08 | 8.90E+05 | 6.58E+05 | NA | NA | NA | NA |
| **847** | NA | NA | 3.61E+08 | 3.30E+08 | 2.44E+06 | 1.56E+06 | NA | NA | NA | NA |
| **848** | NA | NA | 4.26E+08 | 2.66E+08 | 1.12E+06 | 6.21E+05 | NA | NA | NA | NA |
| **849** | NA | NA | 4.23E+08 | 2.63E+08 | 4.58E+05 | 6.00E+05 | NA | NA | NA | NA |
| **850** | NA | NA | 4.23E+08 | 2.63E+08 | 4.58E+05 | 6.00E+05 | NA | NA | NA | NA |
| **851** | NA | NA | 3.85E+08 | 2.97E+08 | 1.29E+06 | 1.80E+05 | NA | NA | NA | NA |
| **852** | 0.00E+00 | 1.81E+06 | 3.68E+08 | 3.13E+08 | 1.08E+06 | 0.00E+00 | 0.00E+00 | 0.00E+00 | 5.77E-03 | Inf |
| **853** | NA | NA | 3.62E+08 | 3.16E+08 | 1.02E+06 | 9.26E+05 | NA | NA | NA | NA |
| **854** | NA | NA | 4.12E+08 | 2.64E+08 | 2.21E+06 | 1.30E+06 | NA | NA | NA | NA |
| **855** | 2.07E+05 | 0.00E+00 | 3.26E+08 | 3.51E+08 | 5.52E+05 | 4.36E+05 | 6.36E-04 | 3.75E-01 | 0.00E+00 | 0.00E+00 |
| **856** | NA | NA | 4.13E+08 | 2.62E+08 | 1.12E+06 | 8.68E+05 | NA | NA | NA | NA |
| **857** | 0.00E+00 | 7.32E+05 | 3.15E+08 | 3.53E+08 | 2.24E+06 | 2.13E+06 | 0.00E+00 | 0.00E+00 | 2.07E-03 | 3.44E-01 |
| **858** | NA | NA | 3.36E+08 | 3.33E+08 | 3.98E+05 | 0.00E+00 | NA | NA | NA | NA |
| **859** | NA | NA | 3.40E+08 | 3.28E+08 | 1.97E+06 | 0.00E+00 | NA | NA | NA | NA |
| **860** | NA | NA | 3.45E+08 | 3.22E+08 | 1.67E+06 | 0.00E+00 | NA | NA | NA | NA |
| **861** | NA | NA | 3.36E+08 | 3.25E+08 | 3.71E+06 | 1.56E+06 | NA | NA | NA | NA |
| **862** | NA | NA | 3.52E+08 | 3.07E+08 | 1.56E+06 | 1.43E+06 | NA | NA | NA | NA |
| **863** | NA | NA | 3.86E+08 | 2.72E+08 | 1.33E+06 | 1.51E+06 | NA | NA | NA | NA |
| **864** | NA | NA | 3.19E+08 | 3.37E+08 | 1.15E+06 | 7.21E+05 | NA | NA | NA | NA |
| **865** | 0.00E+00 | 1.64E+07 | 3.58E+08 | 2.97E+08 | 1.41E+06 | 3.87E+05 | 0.00E+00 | 0.00E+00 | 5.53E-02 | 4.25E+01 |
| **866** | NA | NA | 4.12E+08 | 2.42E+08 | 1.41E+06 | 0.00E+00 | NA | NA | NA | NA |
| **867** | NA | NA | 3.11E+08 | 3.43E+08 | 8.51E+05 | 0.00E+00 | NA | NA | NA | NA |
| **868** | NA | NA | 3.17E+08 | 3.35E+08 | 1.37E+06 | 0.00E+00 | NA | NA | NA | NA |
| **869** | NA | NA | 3.11E+08 | 3.37E+08 | 1.15E+06 | 1.00E+06 | NA | NA | NA | NA |
| **870** | 0.00E+00 | 2.79E+06 | 3.74E+08 | 2.69E+08 | 1.88E+06 | 1.08E+06 | 0.00E+00 | 0.00E+00 | 1.04E-02 | 2.60E+00 |

| | | | | | | | | | | |
|---|---|---|---|---|---|---|---|---|---|---|
| **871** | NA | NA | 3.48E+08 | 2.94E+08 | 6.75E+05 | 1.41E+06 | NA | NA | NA | NA |
| **872** | 4.40E+06 | 0.00E+00 | 3.31E+08 | 3.07E+08 | 3.62E+06 | 2.02E+06 | 1.33E-02 | 1.21E+00 | 0.00E+00 | 0.00E+00 |
| **873** | 3.45E+06 | 2.23E+06 | 2.44E+08 | 3.91E+08 | 2.41E+06 | 1.78E+06 | 1.41E-02 | 1.43E+00 | 5.70E-03 | 1.25E+00 |
| **874** | NA | NA | 3.11E+08 | 3.20E+08 | 4.52E+06 | 3.97E+06 | NA | NA | NA | NA |
| **875** | NA | NA | 3.41E+08 | 2.97E+08 | 4.94E+05 | 0.00E+00 | NA | NA | NA | NA |
| **876** | NA | NA | 3.47E+08 | 2.88E+08 | 1.00E+06 | 2.23E+06 | NA | NA | NA | NA |
| **877** | NA | NA | 3.97E+08 | 2.38E+08 | 9.67E+05 | 5.18E+05 | NA | NA | NA | NA |
| **878** | NA | NA | 3.57E+08 | 2.71E+08 | 5.14E+05 | 0.00E+00 | NA | NA | NA | NA |
| **879** | 2.33E+06 | 9.74E+05 | 2.83E+08 | 3.40E+08 | 1.31E+06 | 2.23E+06 | 8.24E-03 | 1.78E+00 | 2.87E-03 | 4.37E-01 |
| **880** | NA | NA | 3.82E+08 | 2.40E+08 | 1.18E+06 | 1.55E+06 | NA | NA | NA | NA |
| **881** | NA | NA | 3.05E+08 | 3.18E+08 | 8.32E+05 | 6.09E+05 | NA | NA | NA | NA |
| **882** | 0.00E+00 | 5.96E+05 | 3.63E+08 | 2.54E+08 | 2.23E+06 | 4.14E+05 | 0.00E+00 | 0.00E+00 | 2.35E-03 | 1.44E+00 |
| **883** | NA | NA | 4.05E+08 | 2.13E+08 | 0.00E+00 | 2.30E+05 | NA | NA | NA | NA |
| **884** | NA | NA | 3.28E+08 | 2.88E+08 | 1.63E+06 | 4.51E+05 | NA | NA | NA | NA |
| **885** | NA | NA | 3.19E+08 | 2.95E+08 | 1.35E+06 | 1.54E+06 | NA | NA | NA | NA |
| **886** | NA | NA | 2.63E+08 | 3.45E+08 | 4.67E+06 | 2.17E+06 | NA | NA | NA | NA |
| **887** | NA | NA | 3.05E+08 | 3.07E+08 | 8.23E+04 | 1.21E+05 | NA | NA | NA | NA |
| **888** | NA | NA | 3.96E+08 | 2.13E+08 | 1.48E+06 | 1.19E+06 | NA | NA | NA | NA |
| **889** | 1.12E+05 | 3.42E+07 | 3.19E+08 | 2.87E+08 | 1.32E+06 | 1.22E+06 | 3.51E-04 | 8.45E-02 | 1.19E-01 | 2.81E+01 |
| **890** | NA | NA | 3.19E+08 | 2.88E+08 | NA | NA | NA | NA | NA | NA |
| **891** | NA | NA | 3.23E+08 | 2.78E+08 | 1.48E+06 | 9.87E+05 | NA | NA | NA | NA |
| **892** | NA | NA | 2.60E+08 | 3.43E+08 | NA | NA | NA | NA | NA | NA |
| **893** | NA | NA | 2.19E+08 | 3.81E+08 | NA | NA | NA | NA | NA | NA |
| **894** | NA | NA | 2.45E+08 | 3.47E+08 | 4.33E+06 | 3.87E+06 | NA | NA | NA | NA |
| **895** | NA | NA | 3.06E+08 | 2.90E+08 | 2.13E+06 | 7.17E+05 | NA | NA | NA | NA |
| **896** | 3.37E+06 | 0.00E+00 | 3.19E+08 | 2.77E+08 | 1.84E+06 | 8.23E+05 | 1.06E-02 | 1.83E+00 | 0.00E+00 | 0.00E+00 |
| **897** | NA | NA | 2.81E+08 | 3.15E+08 | 2.39E+06 | 0.00E+00 | NA | NA | NA | NA |
| **898** | NA | NA | 3.10E+08 | 2.87E+08 | 7.01E+05 | 5.25E+05 | NA | NA | NA | NA |
| **899** | NA | NA | 3.47E+08 | 2.49E+08 | 4.91E+05 | 3.00E+05 | NA | NA | NA | NA |
| **900** | NA | NA | 3.05E+08 | 2.91E+08 | 2.12E+05 | 5.02E+05 | NA | NA | NA | NA |
| **901** | NA | NA | 3.74E+08 | 2.21E+08 | 6.87E+05 | 1.18E+05 | NA | NA | NA | NA |
| **902** | NA | NA | 2.89E+08 | 3.06E+08 | 7.80E+05 | 0.00E+00 | NA | NA | NA | NA |
| **903** | 4.50E+05 | 0.00E+00 | 2.96E+08 | 2.95E+08 | 1.71E+06 | 2.23E+06 | 1.52E-03 | 2.63E-01 | 0.00E+00 | 0.00E+00 |
| **904** | NA | NA | 3.17E+08 | 2.75E+08 | 1.67E+05 | 1.47E+05 | NA | NA | NA | NA |
| **905** | 8.80E+07 | 2.67E+06 | 2.55E+08 | 3.28E+08 | 2.16E+06 | 5.59E+06 | 3.46E-01 | 4.08E+01 | 8.14E-03 | 4.78E-01 |
| **906** | NA | NA | 2.48E+08 | 3.37E+08 | 1.92E+06 | 6.07E+05 | NA | NA | NA | NA |
| **907** | NA | NA | 2.53E+08 | 3.32E+08 | 7.39E+05 | 1.51E+06 | NA | NA | NA | NA |
| **908** | NA | NA | 2.96E+08 | 2.91E+08 | 1.53E+05 | 2.09E+05 | NA | NA | NA | NA |

| | | | | | | | | | | |
|---|---|---|---|---|---|---|---|---|---|---|
| **909** | NA | NA | 3.38E+08 | 2.47E+08 | 1.03E+06 | 7.70E+05 | NA | NA | NA | NA |
| **910** | NA | NA | 3.39E+08 | 2.45E+08 | 1.91E+05 | 1.39E+06 | NA | NA | NA | NA |
| **911** | NA | NA | 3.30E+08 | 2.49E+08 | 2.29E+06 | 2.11E+06 | NA | NA | NA | NA |
| **912** | NA | NA | 2.83E+08 | 3.00E+08 | 2.57E+05 | 0.00E+00 | NA | NA | NA | NA |
| **913** | 6.26E+06 | 0.00E+00 | 4.80E+07 | 5.32E+08 | 0.00E+00 | 1.50E+05 | 1.30E-01 | Inf | 0.00E+00 | 0.00E+00 |
| **914** | NA | NA | 2.76E+08 | 3.00E+08 | 6.16E+05 | 6.27E+05 | NA | NA | NA | NA |
| **915** | 4.48E+07 | 1.47E+07 | NA | NA | 2.83E+08 | 2.93E+08 | NA | 1.58E-01 | NA | 5.00E-02 |
| **916** | NA | NA | 2.32E+08 | 3.43E+08 | 3.15E+05 | 0.00E+00 | NA | NA | NA | NA |
| **917** | NA | NA | 2.98E+08 | 2.72E+08 | 1.24E+06 | 8.95E+05 | NA | NA | NA | NA |
| **918** | NA | NA | 3.19E+08 | 2.48E+08 | 1.60E+06 | 9.95E+05 | NA | NA | NA | NA |
| **919** | 5.32E+05 | 0.00E+00 | 4.76E+08 | 9.19E+07 | 6.46E+05 | 0.00E+00 | 1.12E-03 | 8.23E-01 | 0.00E+00 | NA |
| **920** | 0.00E+00 | 5.39E+06 | 3.09E+08 | 2.58E+08 | 8.65E+05 | 0.00E+00 | 0.00E+00 | 0.00E+00 | 2.09E-02 | Inf |
| **921** | NA | NA | 3.05E+08 | 2.55E+08 | 2.90E+06 | 2.08E+06 | NA | NA | NA | NA |
| **922** | 2.98E+07 | 0.00E+00 | 3.43E+08 | 2.12E+08 | 3.26E+06 | 5.50E+06 | 8.69E-02 | 9.15E+00 | 0.00E+00 | 0.00E+00 |
| **923** | NA | NA | 2.84E+08 | 2.75E+08 | 8.21E+05 | 1.22E+06 | NA | NA | NA | NA |
| **924** | NA | NA | 2.76E+08 | 2.84E+08 | 6.44E+05 | 5.38E+05 | NA | NA | NA | NA |
| **925** | NA | NA | 2.77E+08 | 2.76E+08 | 4.37E+06 | 2.16E+06 | NA | NA | NA | NA |
| **926** | NA | NA | 3.22E+08 | 2.37E+08 | 1.83E+05 | 3.26E+05 | NA | NA | NA | NA |
| **927** | NA | NA | 1.97E+08 | 3.55E+08 | 2.96E+06 | 1.92E+06 | NA | NA | NA | NA |
| **928** | NA | NA | 3.05E+08 | 2.49E+08 | NA | NA | NA | NA | NA | NA |
| **929** | NA | NA | 3.02E+08 | 2.50E+08 | 4.99E+05 | 1.99E+06 | NA | NA | NA | NA |
| **930** | NA | NA | 2.93E+08 | 2.57E+08 | 2.87E+06 | 1.59E+06 | NA | NA | NA | NA |
| **931** | NA | NA | 2.82E+08 | 2.71E+08 | 1.25E+06 | 0.00E+00 | NA | NA | NA | NA |
| **932** | 0.00E+00 | 3.70E+07 | 2.92E+08 | 2.54E+08 | 2.94E+06 | 1.32E+06 | 0.00E+00 | 0.00E+00 | 1.46E-01 | 2.81E+01 |
| **933** | 6.77E+06 | 9.96E+06 | 2.48E+08 | 2.97E+08 | 3.27E+06 | 1.63E+06 | 2.73E-02 | 2.07E+00 | 3.36E-02 | 6.09E+00 |
| **934** | NA | NA | 2.68E+08 | 2.80E+08 | 1.52E+06 | 0.00E+00 | NA | NA | NA | NA |
| **935** | NA | NA | 3.19E+08 | 2.28E+08 | 7.51E+05 | 8.56E+04 | NA | NA | NA | NA |
| **936** | NA | NA | 3.00E+08 | 2.46E+08 | 1.64E+06 | 2.67E+05 | NA | NA | NA | NA |
| **937** | 2.28E+06 | 0.00E+00 | 2.89E+08 | 2.57E+08 | 1.57E+06 | 1.13E+05 | 7.87E-03 | 1.45E+00 | 0.00E+00 | 0.00E+00 |
| **938** | NA | NA | 2.21E+08 | 3.18E+08 | 3.36E+06 | 7.92E+05 | NA | NA | NA | NA |
| **939** | NA | NA | 2.86E+08 | 2.55E+08 | 2.15E+06 | 0.00E+00 | NA | NA | NA | NA |
| **940** | NA | NA | 2.85E+08 | 2.48E+08 | 1.58E+06 | 2.47E+06 | NA | NA | NA | NA |
| **941** | NA | NA | 3.43E+08 | 1.80E+08 | 7.11E+06 | 6.10E+06 | NA | NA | NA | NA |
| **942** | 0.00E+00 | 5.15E+05 | 3.27E+08 | 2.07E+08 | 3.75E+05 | 1.03E+06 | 0.00E+00 | 0.00E+00 | 2.49E-03 | 4.99E-01 |
| **943** | 2.43E+07 | 1.36E+07 | 2.03E+08 | 3.26E+08 | 2.70E+06 | 1.52E+06 | 1.20E-01 | 9.01E+00 | 4.17E-02 | 8.93E+00 |
| **944** | 7.48E+05 | 2.71E+06 | 2.60E+08 | 2.68E+08 | 2.06E+06 | 2.72E+06 | 2.87E-03 | 3.63E-01 | 1.01E-02 | 9.98E-01 |
| **945** | NA | NA | 2.52E+08 | 2.81E+08 | 9.01E+05 | 0.00E+00 | NA | NA | NA | NA |
| **946** | NA | NA | 2.77E+08 | 2.53E+08 | 1.42E+06 | 2.11E+05 | NA | NA | NA | NA |

| | | | | | | | | | | |
|---|---|---|---|---|---|---|---|---|---|---|
| **947** | 1.49E+07 | 6.74E+06 | 3.18E+08 | 2.11E+08 | 1.64E+06 | 1.21E+06 | 4.68E-02 | 9.08E+00 | 3.20E-02 | 5.56E+00 |
| **948** | NA | NA | 3.23E+08 | 2.02E+08 | 1.89E+06 | 6.99E+05 | NA | NA | NA | NA |
| **949** | NA | NA | 2.29E+08 | 2.97E+08 | 3.12E+05 | 2.83E+05 | NA | NA | NA | NA |
| **950** | NA | NA | 2.13E+08 | 3.12E+08 | 8.59E+05 | 7.28E+05 | NA | NA | NA | NA |
| **951** | 0.00E+00 | 6.01E+06 | 2.91E+08 | 2.33E+08 | 7.53E+05 | 0.00E+00 | 0.00E+00 | 0.00E+00 | 2.58E-02 | Inf |
| **952** | 1.36E+07 | 5.37E+05 | 2.75E+08 | 2.44E+08 | 2.05E+06 | 1.23E+06 | 4.94E-02 | 6.63E+00 | 2.20E-03 | 4.36E-01 |
| **953** | 2.08E+06 | 0.00E+00 | 2.50E+08 | 2.64E+08 | 1.93E+06 | 2.18E+05 | 8.33E-03 | 1.08E+00 | 0.00E+00 | 0.00E+00 |
| **954** | NA | NA | 2.65E+08 | 2.48E+08 | NA | NA | NA | NA | NA | NA |
| **955** | 1.05E+06 | 0.00E+00 | 2.49E+08 | 2.59E+08 | 7.06E+05 | 2.09E+06 | 4.20E-03 | 1.48E+00 | 0.00E+00 | 0.00E+00 |
| **956** | NA | NA | 2.47E+08 | 2.54E+08 | 3.03E+06 | 3.68E+06 | NA | NA | NA | NA |
| **957** | NA | NA | 2.21E+08 | 2.84E+08 | 8.28E+05 | 4.43E+05 | NA | NA | NA | NA |
| **958** | NA | NA | 2.51E+08 | 2.46E+08 | 6.18E+06 | 2.50E+06 | NA | NA | NA | NA |
| **959** | NA | NA | 2.70E+08 | 2.35E+08 | 0.00E+00 | 0.00E+00 | NA | NA | NA | NA |
| **960** | 3.06E+06 | 2.79E+05 | 2.82E+08 | 2.16E+08 | 4.54E+06 | 1.89E+06 | 1.08E-02 | 6.74E-01 | 1.29E-03 | 1.48E-01 |
| **961** | NA | NA | 2.72E+08 | 2.26E+08 | 4.50E+05 | 3.76E+05 | NA | NA | NA | NA |
| **962** | 3.53E+05 | 0.00E+00 | 2.69E+08 | 2.26E+08 | 3.76E+05 | 1.52E+06 | 1.31E-03 | 9.38E-01 | 0.00E+00 | 0.00E+00 |
| **963** | NA | NA | 2.53E+08 | 2.34E+08 | 1.30E+06 | 3.11E+06 | NA | NA | NA | NA |
| **964** | 4.50E+06 | 0.00E+00 | 2.48E+08 | 2.41E+08 | 3.33E+06 | 0.00E+00 | 1.82E-02 | 1.35E+00 | 0.00E+00 | NA |
| **965** | NA | NA | 2.77E+08 | 2.02E+08 | 3.99E+06 | 4.07E+06 | NA | NA | NA | NA |
| **966** | NA | NA | 2.49E+08 | 2.37E+08 | 4.89E+05 | 4.66E+05 | NA | NA | NA | NA |
| **967** | NA | NA | 2.93E+08 | 1.89E+08 | 2.61E+06 | 2.08E+06 | NA | NA | NA | NA |
| **968** | NA | NA | 2.62E+08 | 2.23E+08 | 3.27E+05 | 9.93E+05 | NA | NA | NA | NA |
| **969** | NA | NA | 2.48E+08 | 2.34E+08 | 1.40E+06 | 7.69E+04 | NA | NA | NA | NA |
| **970** | 1.36E+06 | 1.82E+06 | 2.57E+08 | 2.24E+08 | 1.19E+06 | 9.75E+05 | 5.30E-03 | 1.14E+00 | 8.09E-03 | 1.86E+00 |
| **971** | NA | NA | 2.71E+08 | 2.10E+08 | NA | NA | NA | NA | NA | NA |
| **972** | NA | NA | 2.60E+08 | 2.20E+08 | 1.38E+06 | 0.00E+00 | NA | NA | NA | NA |
| **973** | NA | NA | 2.58E+08 | 2.11E+08 | 4.36E+06 | 5.90E+06 | NA | NA | NA | NA |
| **974** | 3.90E+06 | 3.17E+06 | 2.43E+08 | 2.30E+08 | 4.11E+06 | 1.99E+06 | 1.61E-02 | 9.49E-01 | 1.38E-02 | 1.59E+00 |
| **975** | NA | NA | 2.67E+08 | 2.10E+08 | 0.00E+00 | 1.64E+06 | NA | NA | NA | NA |
| **976** | NA | NA | 2.55E+08 | 2.19E+08 | 8.70E+05 | 1.26E+06 | NA | NA | NA | NA |
| **977** | 7.79E+06 | 1.10E+07 | 1.54E+08 | 3.18E+08 | 3.19E+06 | 1.33E+06 | 5.07E-02 | 2.44E+00 | 3.45E-02 | 8.22E+00 |
| **978** | NA | NA | 2.71E+08 | 2.00E+08 | 1.68E+06 | 8.74E+05 | NA | NA | NA | NA |
| **979** | NA | NA | 2.42E+08 | 2.20E+08 | 4.48E+06 | 3.12E+06 | NA | NA | NA | NA |
| **980** | NA | NA | 2.10E+08 | 2.57E+08 | 8.49E+05 | 7.15E+05 | NA | NA | NA | NA |
| **981** | NA | NA | 2.38E+08 | 2.31E+08 | NA | NA | NA | NA | NA | NA |
| **982** | NA | NA | 2.52E+08 | 2.16E+08 | 0.00E+00 | 0.00E+00 | NA | NA | NA | NA |
| **983** | NA | NA | 2.14E+08 | 2.49E+08 | 1.10E+06 | 9.94E+05 | NA | NA | NA | NA |
| **984** | NA | NA | 2.40E+08 | 2.24E+08 | 0.00E+00 | 0.00E+00 | NA | NA | NA | NA |

| | | | | | | | | | | |
|---|---|---|---|---|---|---|---|---|---|---|
| **985** | NA | NA | 2.53E+08 | 2.08E+08 | 8.39E+05 | 0.00E+00 | NA | NA | NA | NA |
| **986** | 1.18E+07 | 8.46E+06 | 2.28E+08 | 2.33E+08 | 8.97E+05 | 8.61E+04 | 5.16E-02 | 1.31E+01 | 3.63E-02 | 9.83E+01 |
| **987** | 3.11E+05 | 0.00E+00 | 2.30E+08 | 2.23E+08 | 1.95E+06 | 2.65E+05 | 1.35E-03 | 1.60E-01 | 0.00E+00 | 0.00E+00 |
| **988** | NA | NA | 2.84E+08 | 1.71E+08 | 2.23E+05 | 3.32E+05 | NA | NA | NA | NA |
| **989** | NA | NA | 2.66E+08 | 1.85E+08 | 9.66E+05 | 1.74E+06 | NA | NA | NA | NA |
| **990** | NA | NA | 1.74E+08 | 2.76E+08 | 2.34E+06 | 0.00E+00 | NA | NA | NA | NA |
| **991** | 9.10E+07 | 5.30E+06 | 2.19E+08 | 2.28E+08 | 3.43E+05 | 4.64E+06 | 4.16E-01 | 2.65E+02 | 2.32E-02 | 1.14E+00 |
| **992** | NA | NA | 2.44E+08 | 2.06E+08 | 1.29E+06 | 2.16E+05 | NA | NA | NA | NA |
| **993** | NA | NA | 2.07E+08 | 2.40E+08 | 2.45E+06 | 9.59E+05 | NA | NA | NA | NA |
| **994** | NA | NA | 2.51E+08 | 1.98E+08 | 1.47E+05 | 9.30E+04 | NA | NA | NA | NA |
| **995** | NA | NA | 2.61E+08 | 1.85E+08 | 0.00E+00 | 3.01E+06 | NA | NA | NA | NA |
| **996** | NA | NA | 2.51E+08 | 1.98E+08 | NA | NA | NA | NA | NA | NA |
| **997** | NA | NA | 2.12E+08 | 2.34E+08 | 0.00E+00 | 7.23E+05 | NA | NA | NA | NA |
| **998** | NA | NA | 2.40E+08 | 1.99E+08 | 1.52E+06 | 8.61E+05 | NA | NA | NA | NA |
| **999** | NA | NA | 2.29E+08 | 2.11E+08 | 9.47E+05 | 1.05E+06 | NA | NA | NA | NA |
| **1000** | NA | NA | 2.64E+08 | 1.76E+08 | 6.34E+05 | 6.11E+05 | NA | NA | NA | NA |
| **1001** | NA | NA | 2.31E+08 | 2.10E+08 | 0.00E+00 | 1.35E+05 | NA | NA | NA | NA |
| **1002** | NA | NA | 2.43E+08 | 1.93E+08 | 2.04E+06 | 1.26E+06 | NA | NA | NA | NA |
| **1003** | NA | NA | 2.53E+08 | 1.86E+08 | 0.00E+00 | 6.25E+05 | NA | NA | NA | NA |
| **1004** | NA | NA | 2.00E+08 | 2.33E+08 | 1.20E+06 | 5.51E+05 | NA | NA | NA | NA |
| **1005** | NA | NA | 2.82E+08 | 1.53E+08 | NA | NA | NA | NA | NA | NA |
| **1006** | 3.24E+06 | 0.00E+00 | 2.45E+08 | 1.86E+08 | 7.44E+05 | 0.00E+00 | 1.33E-02 | 4.36E+00 | 0.00E+00 | NA |
| **1007** | 2.97E+05 | 1.12E+06 | 2.07E+08 | 2.18E+08 | 2.46E+06 | 9.02E+05 | 1.44E-03 | 1.21E-01 | 5.14E-03 | 1.25E+00 |
| **1008** | NA | NA | 1.72E+08 | 2.55E+08 | 7.51E+05 | 2.81E+05 | NA | NA | NA | NA |
| **1009** | NA | NA | 1.80E+08 | 2.48E+08 | 3.66E+05 | 0.00E+00 | NA | NA | NA | NA |
| **1010** | NA | NA | 2.29E+08 | 1.94E+08 | 1.02E+06 | 1.35E+06 | NA | NA | NA | NA |
| **1011** | NA | NA | 2.04E+08 | 2.19E+08 | 1.70E+06 | 0.00E+00 | NA | NA | NA | NA |
| **1012** | NA | NA | 2.64E+08 | 1.58E+08 | NA | NA | NA | NA | NA | NA |
| **1013** | NA | NA | 1.87E+08 | 2.33E+08 | 2.27E+05 | 4.71E+05 | NA | NA | NA | NA |
| **1014** | NA | NA | 2.03E+08 | 2.17E+08 | NA | NA | NA | NA | NA | NA |
| **1015** | NA | NA | 2.09E+08 | 2.07E+08 | 3.59E+05 | 3.73E+05 | NA | NA | NA | NA |
| **1016** | NA | NA | 2.36E+08 | 1.78E+08 | 0.00E+00 | 1.77E+05 | NA | NA | NA | NA |
| **1017** | NA | NA | 2.51E+08 | 1.62E+08 | 5.04E+05 | 0.00E+00 | NA | NA | NA | NA |
| **1018** | NA | NA | 2.06E+08 | 2.06E+08 | 1.06E+06 | 3.44E+05 | NA | NA | NA | NA |
| **1019** | 9.01E+05 | 0.00E+00 | 2.71E+08 | 1.40E+08 | 5.95E+05 | 1.10E+06 | 3.32E-03 | 1.51E+00 | 0.00E+00 | 0.00E+00 |
| **1020** | NA | NA | 2.61E+08 | 1.51E+08 | NA | NA | NA | NA | NA | NA |
| **1021** | NA | NA | 2.50E+08 | 1.62E+08 | NA | NA | NA | NA | NA | NA |
| **1022** | NA | NA | 2.30E+08 | 1.80E+08 | 5.49E+05 | 5.09E+05 | NA | NA | NA | NA |

| | | | | | | | | | | |
|---|---|---|---|---|---|---|---|---|---|---|
| **1023** | NA | NA | 2.07E+08 | 1.98E+08 | 2.02E+06 | 3.74E+05 | NA | NA | NA | NA |
| **1024** | NA | NA | 2.24E+08 | 1.83E+08 | 0.00E+00 | 0.00E+00 | NA | NA | NA | NA |
| **1025** | NA | NA | 2.06E+08 | 1.97E+08 | 1.61E+06 | 1.38E+06 | NA | NA | NA | NA |
| **1026** | NA | NA | 2.18E+08 | 1.83E+08 | 2.79E+06 | 6.96E+05 | NA | NA | NA | NA |
| **1027** | NA | NA | 1.97E+08 | 2.06E+08 | 0.00E+00 | 1.07E+05 | NA | NA | NA | NA |
| **1028** | NA | NA | 2.24E+08 | 1.78E+08 | 0.00E+00 | 2.66E+05 | NA | NA | NA | NA |
| **1029** | NA | NA | 1.87E+08 | 2.13E+08 | 0.00E+00 | 0.00E+00 | NA | NA | NA | NA |
| **1030** | 6.84E+06 | 1.04E+06 | 2.00E+08 | 1.98E+08 | 1.33E+06 | 8.97E+05 | 3.42E-02 | 5.15E+00 | 5.27E-03 | 1.16E+00 |
| **1031** | NA | NA | 2.00E+08 | 1.98E+08 | 7.83E+05 | 0.00E+00 | NA | NA | NA | NA |
| **1032** | NA | NA | 1.87E+08 | 2.06E+08 | 3.41E+06 | 1.45E+06 | NA | NA | NA | NA |
| **1033** | NA | NA | 2.04E+08 | 1.93E+08 | 8.18E+05 | 9.16E+04 | NA | NA | NA | NA |
| **1034** | NA | NA | 2.19E+08 | 1.76E+08 | 8.48E+05 | 2.78E+05 | NA | NA | NA | NA |
| **1035** | 0.00E+00 | 7.84E+07 | 2.64E+08 | 1.32E+08 | 0.00E+00 | 0.00E+00 | 0.00E+00 | NA | 5.94E-01 | Inf |
| **1036** | 2.96E+06 | 9.49E+06 | 1.98E+08 | 1.91E+08 | 1.29E+06 | 9.07E+05 | 1.49E-02 | 2.29E+00 | 4.97E-02 | 1.05E+01 |
| **1037** | NA | NA | 2.24E+08 | 1.66E+08 | 4.96E+05 | 0.00E+00 | NA | NA | NA | NA |
| **1038** | NA | NA | 1.80E+08 | 2.06E+08 | 9.81E+05 | 3.16E+06 | NA | NA | NA | NA |
| **1039** | 3.90E+06 | 0.00E+00 | 2.25E+08 | 1.61E+08 | 9.34E+05 | 1.99E+06 | 1.73E-02 | 4.18E+00 | 0.00E+00 | 0.00E+00 |
| **1040** | NA | NA | 2.03E+08 | 1.85E+08 | 0.00E+00 | 7.74E+05 | NA | NA | NA | NA |
| **1041** | 1.23E+08 | 1.77E+08 | 1.92E+08 | 1.87E+08 | 2.46E+06 | 3.40E+06 | 6.42E-01 | 5.02E+01 | 9.49E-01 | 5.22E+01 |
| **1042** | NA | NA | 1.68E+08 | 2.14E+08 | 1.38E+06 | 9.75E+05 | NA | NA | NA | NA |
| **1043** | NA | NA | 2.02E+08 | 1.80E+08 | 0.00E+00 | 2.10E+05 | NA | NA | NA | NA |
| **1044** | 1.28E+07 | 0.00E+00 | 1.66E+08 | 2.09E+08 | 2.52E+06 | 2.84E+06 | 7.70E-02 | 5.06E+00 | 0.00E+00 | 0.00E+00 |
| **1045** | NA | NA | 1.92E+08 | 1.86E+08 | 9.06E+05 | 7.85E+05 | NA | NA | NA | NA |
| **1046** | 0.00E+00 | 6.62E+05 | 1.58E+08 | 2.20E+08 | 8.12E+05 | 4.44E+05 | 0.00E+00 | 0.00E+00 | 3.01E-03 | 1.49E+00 |
| **1047** | NA | NA | 2.55E+08 | 1.20E+08 | 1.10E+06 | 0.00E+00 | NA | NA | NA | NA |
| **1048** | NA | NA | 1.91E+08 | 1.84E+08 | 0.00E+00 | 0.00E+00 | NA | NA | NA | NA |
| **1049** | NA | NA | 1.61E+08 | 2.11E+08 | 1.77E+06 | 1.30E+06 | NA | NA | NA | NA |
| **1050** | NA | NA | 1.78E+08 | 1.94E+08 | 1.55E+06 | 2.01E+05 | NA | NA | NA | NA |
| **1051** | NA | NA | 1.72E+08 | 2.00E+08 | 4.97E+05 | 4.08E+05 | NA | NA | NA | NA |
| **1052** | NA | NA | 1.62E+08 | 2.01E+08 | 1.60E+06 | 6.38E+06 | NA | NA | NA | NA |
| **1053** | NA | NA | 1.77E+08 | 1.90E+08 | 3.20E+06 | 1.62E+06 | NA | NA | NA | NA |
| **1054** | NA | NA | 1.64E+08 | 2.04E+08 | 2.26E+06 | 1.31E+05 | NA | NA | NA | NA |
| **1055** | 0.00E+00 | 0.00E+00 | 2.28E+08 | 1.37E+08 | 2.55E+06 | 2.11E+06 | 0.00E+00 | 0.00E+00 | 0.00E+00 | 0.00E+00 |
| **1056** | NA | NA | 2.39E+08 | 1.30E+08 | 0.00E+00 | 0.00E+00 | NA | NA | NA | NA |
| **1057** | NA | NA | 1.59E+08 | 2.07E+08 | 2.30E+06 | 3.68E+05 | NA | NA | NA | NA |
| **1058** | NA | NA | 1.72E+08 | 1.97E+08 | 0.00E+00 | 2.46E+05 | NA | NA | NA | NA |
| **1059** | 2.90E+05 | 0.00E+00 | 1.56E+08 | 2.10E+08 | 1.42E+05 | 1.25E+06 | 1.86E-03 | 2.04E+00 | 0.00E+00 | 0.00E+00 |
| **1060** | NA | NA | 1.97E+08 | 1.69E+08 | 9.93E+05 | 4.17E+05 | NA | NA | NA | NA |

| | | | | | | | | | | |
|---|---|---|---|---|---|---|---|---|---|---|
| **1061** | 2.02E+06 | 0.00E+00 | 2.12E+08 | 1.55E+08 | 0.00E+00 | 0.00E+00 | 9.55E-03 | Inf | 0.00E+00 | NA |
| **1062** | 2.57E+05 | 0.00E+00 | 2.42E+08 | 1.21E+08 | 1.07E+06 | 7.64E+05 | 1.06E-03 | 2.39E-01 | 0.00E+00 | 0.00E+00 |
| **1063** | NA | NA | 1.92E+08 | 1.71E+08 | 5.73E+05 | 2.27E+05 | NA | NA | NA | NA |
| **1064** | 8.98E+05 | 2.56E+08 | 1.50E+08 | 2.09E+08 | 2.10E+06 | 3.86E+05 | 5.97E-03 | 4.28E-01 | 1.22E+00 | 6.62E+02 |
| **1065** | NA | NA | 2.19E+08 | 1.42E+08 | NA | NA | NA | NA | NA | NA |
| **1066** | NA | NA | 2.03E+08 | 1.55E+08 | 1.35E+06 | 8.26E+05 | NA | NA | NA | NA |
| **1067** | NA | NA | 1.21E+08 | 2.35E+08 | 1.36E+06 | 2.46E+06 | NA | NA | NA | NA |
| **1068** | NA | NA | 1.89E+08 | 1.65E+08 | 2.15E+06 | 1.10E+06 | NA | NA | NA | NA |
| **1069** | NA | NA | 2.04E+08 | 1.52E+08 | 8.83E+05 | 0.00E+00 | NA | NA | NA | NA |
| **1070** | NA | NA | 1.79E+08 | 1.75E+08 | 1.49E+06 | 1.35E+06 | NA | NA | NA | NA |
| **1071** | NA | NA | 2.01E+08 | 1.54E+08 | NA | NA | NA | NA | NA | NA |
| **1072** | NA | NA | 1.67E+08 | 1.86E+08 | 0.00E+00 | 0.00E+00 | NA | NA | NA | NA |
| **1073** | NA | NA | 2.17E+08 | 1.36E+08 | NA | NA | NA | NA | NA | NA |
| **1074** | NA | NA | 2.02E+08 | 1.50E+08 | 5.79E+05 | 1.59E+05 | NA | NA | NA | NA |
| **1075** | NA | NA | 2.03E+08 | 1.44E+08 | 2.69E+06 | 2.96E+06 | NA | NA | NA | NA |
| **1076** | NA | NA | 1.85E+08 | 1.65E+08 | 1.22E+06 | 2.46E+05 | NA | NA | NA | NA |
| **1077** | NA | NA | 1.38E+08 | 2.13E+08 | NA | NA | NA | NA | NA | NA |
| **1078** | NA | NA | 1.73E+08 | 1.75E+08 | 0.00E+00 | 1.11E+06 | NA | NA | NA | NA |
| **1079** | NA | NA | 1.99E+08 | 1.49E+08 | NA | NA | NA | NA | NA | NA |
| **1080** | NA | NA | 1.27E+08 | 2.20E+08 | 1.02E+06 | 0.00E+00 | NA | NA | NA | NA |
| **1081** | NA | NA | 2.05E+08 | 1.42E+08 | 3.93E+05 | 0.00E+00 | NA | NA | NA | NA |
| **1082** | NA | NA | 2.42E+08 | 1.03E+08 | 4.70E+05 | 7.99E+05 | NA | NA | NA | NA |
| **1083** | NA | NA | 2.13E+08 | 1.32E+08 | 7.76E+05 | 0.00E+00 | NA | NA | NA | NA |
| **1084** | NA | NA | 1.58E+08 | 1.88E+08 | NA | NA | NA | NA | NA | NA |
| **1085** | 1.81E+07 | 0.00E+00 | 1.75E+08 | 1.68E+08 | 5.40E+05 | 1.28E+06 | 1.03E-01 | 3.35E+01 | 0.00E+00 | 0.00E+00 |
| **1086** | 6.09E+06 | 1.34E+06 | 1.75E+08 | 1.64E+08 | 2.27E+06 | 2.74E+06 | 3.48E-02 | 2.68E+00 | 8.17E-03 | 4.90E-01 |
| **1087** | NA | NA | 1.57E+08 | 1.87E+08 | 1.44E+05 | 3.54E+05 | NA | NA | NA | NA |
| **1088** | NA | NA | 1.74E+08 | 1.64E+08 | 2.36E+06 | 6.90E+05 | NA | NA | NA | NA |
| **1089** | 2.40E+07 | 4.40E+06 | 1.39E+08 | 1.97E+08 | 1.90E+06 | 2.75E+06 | 1.73E-01 | 1.26E+01 | 2.23E-02 | 1.60E+00 |
| **1090** | 9.91E+05 | 0.00E+00 | 1.71E+08 | 1.67E+08 | 4.97E+05 | 1.93E+06 | 5.79E-03 | 1.99E+00 | 0.00E+00 | 0.00E+00 |
| **1091** | NA | NA | 1.93E+08 | 1.43E+08 | 2.22E+06 | 1.27E+06 | NA | NA | NA | NA |
| **1092** | NA | NA | 2.33E+08 | 1.06E+08 | 1.56E+05 | 0.00E+00 | NA | NA | NA | NA |
| **1093** | NA | NA | 1.73E+08 | 1.61E+08 | 2.18E+06 | 1.70E+06 | NA | NA | NA | NA |
| **1094** | NA | NA | 1.64E+08 | 1.72E+08 | 1.43E+06 | 0.00E+00 | NA | NA | NA | NA |
| **1095** | NA | NA | 1.58E+08 | 1.79E+08 | 0.00E+00 | 5.54E+05 | NA | NA | NA | NA |
| **1096** | NA | NA | 2.04E+08 | 1.33E+08 | 2.14E+05 | 0.00E+00 | NA | NA | NA | NA |
| **1097** | 0.00E+00 | 1.91E+07 | 1.96E+08 | 1.37E+08 | 2.37E+06 | 2.03E+05 | 0.00E+00 | 0.00E+00 | 1.39E-01 | 9.42E+01 |
| **1098** | NA | NA | 1.81E+08 | 1.52E+08 | 1.13E+06 | 1.65E+05 | NA | NA | NA | NA |

| | | | | | | | | | | |
|---|---|---|---|---|---|---|---|---|---|---|
| **1099** | 0.00E+00 | 3.63E+07 | 9.49E+07 | 2.37E+08 | 1.61E+06 | 1.60E+06 | 0.00E+00 | 0.00E+00 | 1.53E-01 | 2.26E+01 |
| **1100** | NA | NA | 2.01E+08 | 1.29E+08 | 0.00E+00 | 1.51E+06 | NA | NA | NA | NA |
| **1101** | 9.42E+06 | 0.00E+00 | 1.64E+08 | 1.63E+08 | 2.22E+06 | 1.79E+06 | 5.76E-02 | 4.25E+00 | 0.00E+00 | 0.00E+00 |
| **1102** | NA | NA | 2.03E+08 | 1.26E+08 | 1.78E+06 | 6.37E+05 | NA | NA | NA | NA |
| **1103** | NA | NA | 1.51E+08 | 1.78E+08 | 1.68E+06 | 0.00E+00 | NA | NA | NA | NA |
| **1104** | NA | NA | 1.77E+08 | 1.50E+08 | 2.45E+06 | 4.13E+05 | NA | NA | NA | NA |
| **1105** | NA | NA | 1.61E+08 | 1.66E+08 | 9.90E+05 | 4.06E+05 | NA | NA | NA | NA |
| **1106** | NA | NA | 1.65E+08 | 1.60E+08 | 8.33E+05 | 6.84E+05 | NA | NA | NA | NA |
| **1107** | NA | NA | 1.69E+08 | 1.55E+08 | 9.11E+05 | 1.59E+06 | NA | NA | NA | NA |
| **1108** | NA | NA | 7.33E+07 | 2.43E+08 | 6.71E+06 | 3.51E+06 | NA | NA | NA | NA |
| **1109** | NA | NA | 1.65E+08 | 1.60E+08 | 1.08E+06 | 0.00E+00 | NA | NA | NA | NA |
| **1110** | NA | NA | 1.17E+08 | 2.00E+08 | 5.67E+06 | 3.69E+06 | NA | NA | NA | NA |
| **1111** | NA | NA | 1.65E+08 | 1.56E+08 | 2.61E+06 | 0.00E+00 | NA | NA | NA | NA |
| **1112** | NA | NA | 1.42E+08 | 1.81E+08 | 8.62E+04 | 2.24E+05 | NA | NA | NA | NA |
| **1113** | NA | NA | 1.61E+08 | 1.60E+08 | 0.00E+00 | 6.47E+04 | NA | NA | NA | NA |
| **1114** | NA | NA | 1.78E+08 | 1.40E+08 | 3.24E+05 | 1.34E+06 | NA | NA | NA | NA |
| **1115** | NA | NA | 2.13E+08 | 1.06E+08 | 0.00E+00 | 4.97E+05 | NA | NA | NA | NA |
| **1116** | NA | NA | 1.91E+08 | 1.27E+08 | 2.89E+05 | 1.11E+05 | NA | NA | NA | NA |
| **1117** | NA | NA | 1.72E+08 | 1.40E+08 | 5.55E+06 | 0.00E+00 | NA | NA | NA | NA |
| **1118** | NA | NA | 1.80E+08 | 1.37E+08 | 0.00E+00 | 9.12E+04 | NA | NA | NA | NA |
| **1119** | NA | NA | 1.74E+08 | 1.40E+08 | 1.64E+06 | 9.65E+04 | NA | NA | NA | NA |
| **1120** | 5.28E+07 | 0.00E+00 | 1.39E+08 | 1.70E+08 | 0.00E+00 | 6.39E+06 | 3.79E-01 | Inf | 0.00E+00 | 0.00E+00 |
| **1121** | 1.45E+07 | 1.37E+07 | 1.62E+08 | 1.48E+08 | 2.52E+06 | 1.41E+06 | 8.96E-02 | 5.78E+00 | 9.23E-02 | 9.70E+00 |
| **1122** | NA | NA | 1.84E+08 | 1.30E+08 | 3.11E+05 | 4.91E+05 | NA | NA | NA | NA |
| **1123** | NA | NA | 2.60E+08 | 5.17E+07 | 2.49E+06 | 1.83E+05 | NA | NA | NA | NA |
| **1124** | NA | NA | 1.67E+08 | 1.46E+08 | NA | NA | NA | NA | NA | NA |
| **1125** | NA | NA | 1.40E+08 | 1.67E+08 | 2.79E+06 | 1.17E+06 | NA | NA | NA | NA |
| **1126** | NA | NA | 1.10E+08 | 1.97E+08 | 1.57E+06 | 2.08E+06 | NA | NA | NA | NA |
| **1127** | 2.31E+07 | 8.25E+06 | NA | NA | 1.63E+08 | 1.47E+08 | NA | 1.42E-01 | NA | 5.61E-02 |
| **1128** | NA | NA | 2.23E+08 | 8.62E+07 | 5.36E+05 | 0.00E+00 | NA | NA | NA | NA |
| **1129** | 4.04E+06 | 5.00E+06 | 1.26E+08 | 1.80E+08 | 1.80E+06 | 3.38E+05 | 3.20E-02 | 2.25E+00 | 2.78E-02 | 1.48E+01 |
| **1130** | NA | NA | 1.60E+08 | 1.48E+08 | NA | NA | NA | NA | NA | NA |
| **1131** | 7.54E+06 | 0.00E+00 | 1.27E+08 | 1.79E+08 | 7.92E+05 | 1.38E+06 | 5.91E-02 | 9.52E+00 | 0.00E+00 | 0.00E+00 |
| **1132** | NA | NA | 1.43E+08 | 1.63E+08 | 1.41E+06 | 6.83E+05 | NA | NA | NA | NA |
| **1133** | NA | NA | 1.55E+08 | 1.52E+08 | 5.21E+05 | 0.00E+00 | NA | NA | NA | NA |
| **1134** | NA | NA | 1.62E+08 | 1.46E+08 | NA | NA | NA | NA | NA | NA |
| **1135** | NA | NA | 1.64E+08 | 1.36E+08 | 2.68E+06 | 1.69E+06 | NA | NA | NA | NA |
| **1136** | NA | NA | 1.41E+08 | 1.59E+08 | 1.06E+05 | 8.21E+05 | NA | NA | NA | NA |

| | | | | | | | | | | |
|---|---|---|---|---|---|---|---|---|---|---|
| **1137** | NA | NA | 1.54E+08 | 1.47E+08 | 0.00E+00 | 2.62E+05 | NA | NA | NA | NA |
| **1138** | NA | NA | 1.87E+08 | 1.11E+08 | 1.43E+06 | 1.69E+05 | NA | NA | NA | NA |
| **1139** | NA | NA | 1.23E+08 | 1.75E+08 | 4.73E+05 | 7.96E+05 | NA | NA | NA | NA |
| **1140** | NA | NA | 2.05E+08 | 9.34E+07 | 0.00E+00 | 5.23E+05 | NA | NA | NA | NA |
| **1141** | NA | NA | 1.82E+08 | 1.14E+08 | 1.75E+06 | 8.18E+05 | NA | NA | NA | NA |
| **1142** | NA | NA | 1.74E+08 | 1.23E+08 | 6.10E+05 | 0.00E+00 | NA | NA | NA | NA |
| **1143** | NA | NA | 9.96E+07 | 1.94E+08 | 1.03E+06 | 2.04E+06 | NA | NA | NA | NA |
| **1144** | NA | NA | 1.21E+08 | 1.75E+08 | NA | NA | NA | NA | NA | NA |
| **1145** | 5.38E+07 | 5.98E+06 | 1.54E+08 | 1.38E+08 | 2.37E+06 | 1.95E+06 | 3.49E-01 | 2.26E+01 | 4.31E-02 | 3.07E+00 |
| **1146** | NA | NA | 1.50E+08 | 1.44E+08 | 1.28E+06 | 4.04E+05 | NA | NA | NA | NA |
| **1147** | NA | NA | 1.60E+08 | 1.34E+08 | 1.21E+06 | 1.01E+06 | NA | NA | NA | NA |
| **1148** | 1.57E+07 | 1.30E+07 | 1.38E+08 | 1.53E+08 | 3.10E+06 | 1.69E+06 | 1.14E-01 | 5.08E+00 | 8.48E-02 | 7.65E+00 |
| **1149** | NA | NA | 1.68E+08 | 1.26E+08 | 0.00E+00 | 2.93E+05 | NA | NA | NA | NA |
| **1150** | NA | NA | 1.81E+08 | 1.12E+08 | 1.15E+06 | 1.95E+05 | NA | NA | NA | NA |
| **1151** | NA | NA | 2.03E+08 | 9.03E+07 | 5.08E+04 | 8.84E+04 | NA | NA | NA | NA |
| **1152** | NA | NA | 1.74E+08 | 1.18E+08 | 1.06E+06 | 5.15E+05 | NA | NA | NA | NA |
| **1153** | NA | NA | 1.59E+08 | 1.32E+08 | 1.37E+06 | 0.00E+00 | NA | NA | NA | NA |
| **1154** | NA | NA | 1.48E+08 | 1.42E+08 | NA | NA | NA | NA | NA | NA |
| **1155** | NA | NA | 1.66E+08 | 1.23E+08 | 6.27E+05 | 1.00E+06 | NA | NA | NA | NA |
| **1156** | 3.29E+05 | 0.00E+00 | 2.09E+08 | 8.01E+07 | 0.00E+00 | 6.61E+05 | 1.57E-03 | Inf | 0.00E+00 | 0.00E+00 |
| **1157** | NA | NA | 1.55E+08 | 1.33E+08 | 9.55E+05 | 0.00E+00 | NA | NA | NA | NA |
| **1158** | NA | NA | 1.47E+08 | 1.41E+08 | NA | NA | NA | NA | NA | NA |
| **1159** | NA | NA | 1.44E+08 | 1.41E+08 | 0.00E+00 | 9.28E+05 | NA | NA | NA | NA |
| **1160** | NA | NA | 1.34E+08 | 1.49E+08 | 1.51E+06 | 0.00E+00 | NA | NA | NA | NA |
| **1161** | NA | NA | 2.13E+08 | 6.97E+07 | 0.00E+00 | 1.06E+06 | NA | NA | NA | NA |
| **1162** | NA | NA | 1.38E+08 | 1.45E+08 | NA | NA | NA | NA | NA | NA |
| **1163** | NA | NA | 1.65E+08 | 1.15E+08 | 8.59E+05 | 5.56E+05 | NA | NA | NA | NA |
| **1164** | NA | NA | 1.69E+08 | 1.11E+08 | 4.97E+05 | 0.00E+00 | NA | NA | NA | NA |
| **1165** | 0.00E+00 | 2.56E+06 | 1.50E+08 | 1.29E+08 | 1.27E+06 | 1.08E+06 | 0.00E+00 | 0.00E+00 | 1.98E-02 | 2.37E+00 |
| **1166** | NA | NA | 1.57E+08 | 1.23E+08 | 5.60E+05 | 0.00E+00 | NA | NA | NA | NA |
| **1167** | NA | NA | 1.63E+08 | 1.17E+08 | 0.00E+00 | 0.00E+00 | NA | NA | NA | NA |
| **1168** | NA | NA | 1.24E+08 | 1.53E+08 | 3.09E+06 | 0.00E+00 | NA | NA | NA | NA |
| **1169** | NA | NA | 1.58E+08 | 1.22E+08 | 1.52E+05 | 1.47E+05 | NA | NA | NA | NA |
| **1170** | 1.12E+05 | 2.59E+07 | 1.61E+08 | 1.16E+08 | 1.06E+06 | 1.08E+06 | 6.96E-04 | 1.05E-01 | 2.24E-01 | 2.41E+01 |
| **1171** | 0.00E+00 | 1.09E+07 | 1.42E+08 | 1.37E+08 | 0.00E+00 | 0.00E+00 | 0.00E+00 | NA | 7.96E-02 | Inf |
| **1172** | 8.69E+06 | 0.00E+00 | 1.57E+08 | 1.17E+08 | 1.69E+06 | 1.48E+06 | 5.53E-02 | 5.14E+00 | 0.00E+00 | 0.00E+00 |
| **1173** | NA | NA | 1.75E+08 | 1.01E+08 | 6.49E+05 | 2.16E+05 | NA | NA | NA | NA |
| **1174** | NA | NA | 1.24E+08 | 1.49E+08 | 1.05E+06 | 7.83E+05 | NA | NA | NA | NA |

| | | | | | | | | | | |
|---|---|---|---|---|---|---|---|---|---|---|
| **1175** | NA | NA | 1.91E+08 | 8.44E+07 | NA | NA | NA | NA | NA | NA |
| **1176** | NA | NA | 1.82E+08 | 9.25E+07 | 5.30E+05 | 0.00E+00 | NA | NA | NA | NA |
| **1177** | NA | NA | 1.17E+08 | 1.54E+08 | 1.36E+06 | 0.00E+00 | NA | NA | NA | NA |
| **1178** | 4.67E+06 | 0.00E+00 | 1.38E+08 | 1.33E+08 | 5.29E+05 | 3.70E+05 | 3.39E-02 | 8.82E+00 | 0.00E+00 | 0.00E+00 |
| **1179** | NA | NA | 1.39E+08 | 1.33E+08 | NA | NA | NA | NA | NA | NA |
| **1180** | NA | NA | 1.67E+08 | 9.98E+07 | 4.63E+05 | 2.88E+06 | NA | NA | NA | NA |
| **1181** | NA | NA | 1.55E+08 | 1.15E+08 | 1.90E+05 | 0.00E+00 | NA | NA | NA | NA |
| **1182** | NA | NA | 1.23E+08 | 1.42E+08 | 1.05E+06 | 1.37E+06 | NA | NA | NA | NA |
| **1183** | 0.00E+00 | 0.00E+00 | 1.31E+08 | 1.36E+08 | 0.00E+00 | 0.00E+00 | 0.00E+00 | NA | 0.00E+00 | NA |
| **1184** | 7.02E+06 | 1.30E+07 | 1.66E+08 | 9.94E+07 | 1.24E+06 | 1.61E+05 | 4.22E-02 | 5.67E+00 | 1.31E-01 | 8.08E+01 |
| **1185** | NA | NA | 1.57E+08 | 1.10E+08 | NA | NA | NA | NA | NA | NA |
| **1186** | NA | NA | 1.29E+08 | 1.36E+08 | 6.87E+05 | 8.13E+05 | NA | NA | NA | NA |
| **1187** | NA | NA | 1.54E+08 | 1.07E+08 | 3.25E+06 | 1.40E+06 | NA | NA | NA | NA |
| **1188** | NA | NA | 1.68E+08 | 9.66E+07 | 4.27E+05 | 4.90E+05 | NA | NA | NA | NA |
| **1189** | NA | NA | 1.55E+08 | 1.10E+08 | 0.00E+00 | 0.00E+00 | NA | NA | NA | NA |
| **1190** | NA | NA | 1.55E+08 | 1.07E+08 | 2.41E+06 | 0.00E+00 | NA | NA | NA | NA |
| **1191** | NA | NA | 1.47E+08 | 1.18E+08 | 0.00E+00 | 1.57E+05 | NA | NA | NA | NA |
| **1192** | NA | NA | 1.66E+08 | 9.83E+07 | NA | NA | NA | NA | NA | NA |
| **1193** | NA | NA | 1.54E+08 | 1.07E+08 | 2.53E+06 | 1.52E+05 | NA | NA | NA | NA |
| **1194** | NA | NA | 1.54E+08 | 1.02E+08 | 7.41E+05 | 1.38E+06 | NA | NA | NA | NA |
| **1195** | NA | NA | 1.32E+08 | 1.25E+08 | 0.00E+00 | 2.28E+05 | NA | NA | NA | NA |
| **1196** | NA | NA | 1.23E+08 | 1.35E+08 | 0.00E+00 | 0.00E+00 | NA | NA | NA | NA |
| **1197** | NA | NA | 1.30E+08 | 1.24E+08 | 1.46E+06 | 1.20E+05 | NA | NA | NA | NA |
| **1198** | NA | NA | NA | NA | 1.62E+08 | 9.38E+07 | NA | NA | NA | NA |
| **1199** | NA | NA | 1.34E+08 | 1.19E+08 | 2.44E+05 | 9.15E+05 | NA | NA | NA | NA |
| **1200** | NA | NA | 1.15E+08 | 1.34E+08 | 4.47E+06 | 1.14E+06 | NA | NA | NA | NA |
| **1201** | NA | NA | 1.41E+08 | 1.10E+08 | 0.00E+00 | 2.55E+06 | NA | NA | NA | NA |
| **1202** | NA | NA | 6.20E+07 | 1.89E+08 | 2.10E+06 | 7.26E+05 | NA | NA | NA | NA |
| **1203** | NA | NA | 9.50E+07 | 1.56E+08 | NA | NA | NA | NA | NA | NA |
| **1204** | 0.00E+00 | 7.37E+05 | 1.49E+08 | 9.97E+07 | 1.53E+06 | 6.21E+05 | 0.00E+00 | 0.00E+00 | 7.40E-03 | 1.19E+00 |
| **1205** | NA | NA | 1.36E+08 | 1.14E+08 | NA | NA | NA | NA | NA | NA |
| **1206** | 2.57E+08 | 1.33E+07 | 9.35E+07 | 1.52E+08 | 1.04E+06 | 2.21E+06 | 2.75E+00 | 2.47E+02 | 8.77E-02 | 6.05E+00 |
| **1207** | NA | NA | 1.62E+08 | 8.22E+07 | 2.25E+06 | 1.09E+06 | NA | NA | NA | NA |
| **1208** | NA | NA | 1.08E+08 | 1.36E+08 | 1.46E+06 | 1.93E+06 | NA | NA | NA | NA |
| **1209** | NA | NA | 1.03E+08 | 1.36E+08 | 6.33E+06 | 1.50E+06 | NA | NA | NA | NA |
| **1210** | NA | NA | 1.37E+08 | 1.08E+08 | NA | NA | NA | NA | NA | NA |
| **1211** | NA | NA | 1.33E+08 | 1.12E+08 | NA | NA | NA | NA | NA | NA |
| **1212** | NA | NA | 1.44E+08 | 9.96E+07 | 2.75E+05 | 1.35E+05 | NA | NA | NA | NA |

| | | | | | | | | | | |
|---|---|---|---|---|---|---|---|---|---|---|
| **1213** | 5.74E+07 | 0.00E+00 | 1.22E+08 | 1.19E+08 | 1.11E+06 | 2.05E+06 | 4.72E-01 | 5.18E+01 | 0.00E+00 | 0.00E+00 |
| **1214** | NA | NA | 1.94E+08 | 4.93E+07 | 1.65E+05 | 0.00E+00 | NA | NA | NA | NA |
| **1215** | NA | NA | 1.62E+08 | 7.68E+07 | 3.14E+06 | 7.78E+05 | NA | NA | NA | NA |
| **1216** | NA | NA | 1.46E+08 | 9.35E+07 | 1.37E+06 | 0.00E+00 | NA | NA | NA | NA |
| **1217** | NA | NA | 1.44E+08 | 9.62E+07 | NA | NA | NA | NA | NA | NA |
| **1218** | NA | NA | 9.36E+07 | 1.41E+08 | 4.13E+06 | 8.64E+05 | NA | NA | NA | NA |
| **1219** | NA | NA | 1.22E+08 | 1.16E+08 | 3.28E+05 | 3.98E+05 | NA | NA | NA | NA |
| **1220** | NA | NA | 1.30E+08 | 1.06E+08 | 1.21E+06 | 0.00E+00 | NA | NA | NA | NA |
| **1221** | NA | NA | 1.68E+08 | 6.67E+07 | 1.42E+06 | 1.12E+06 | NA | NA | NA | NA |
| **1222** | 9.86E+05 | 0.00E+00 | 1.11E+08 | 1.24E+08 | 5.30E+05 | 4.18E+05 | 8.88E-03 | 1.86E+00 | 0.00E+00 | 0.00E+00 |
| **1223** | NA | NA | 1.35E+08 | 9.67E+07 | 1.00E+06 | 3.17E+06 | NA | NA | NA | NA |
| **1224** | NA | NA | 1.10E+08 | 1.22E+08 | 1.87E+06 | 1.18E+06 | NA | NA | NA | NA |
| **1225** | NA | NA | 1.21E+08 | 1.13E+08 | 6.81E+05 | 0.00E+00 | NA | NA | NA | NA |
| **1226** | NA | NA | 7.10E+07 | 1.62E+08 | 6.06E+05 | 0.00E+00 | NA | NA | NA | NA |
| **1227** | NA | NA | NA | NA | 1.20E+08 | 1.12E+08 | NA | NA | NA | NA |
| **1228** | NA | NA | 1.48E+08 | 8.35E+07 | NA | NA | NA | NA | NA | NA |
| **1229** | NA | NA | 1.12E+08 | 1.18E+08 | 2.74E+05 | 0.00E+00 | NA | NA | NA | NA |
| **1230** | NA | NA | 9.47E+07 | 1.36E+08 | 0.00E+00 | 1.93E+05 | NA | NA | NA | NA |
| **1231** | NA | NA | 1.14E+08 | 1.15E+08 | 9.63E+05 | 2.51E+05 | NA | NA | NA | NA |
| **1232** | NA | NA | 1.29E+08 | 1.02E+08 | NA | NA | NA | NA | NA | NA |
| **1233** | NA | NA | 1.22E+08 | 1.08E+08 | NA | NA | NA | NA | NA | NA |
| **1234** | NA | NA | 1.02E+08 | 1.27E+08 | NA | NA | NA | NA | NA | NA |
| **1235** | NA | NA | 1.26E+08 | 1.01E+08 | 9.75E+05 | 0.00E+00 | NA | NA | NA | NA |
| **1236** | NA | NA | 1.39E+08 | 8.80E+07 | 0.00E+00 | 1.97E+05 | NA | NA | NA | NA |
| **1237** | NA | NA | 1.01E+08 | 1.25E+08 | 1.84E+06 | 0.00E+00 | NA | NA | NA | NA |
| **1238** | NA | NA | 9.66E+07 | 1.27E+08 | 5.72E+05 | 4.38E+05 | NA | NA | NA | NA |
| **1239** | NA | NA | 1.27E+08 | 9.62E+07 | 8.20E+05 | 0.00E+00 | NA | NA | NA | NA |
| **1240** | 9.70E+05 | 0.00E+00 | 1.43E+08 | 7.81E+07 | 3.55E+05 | 2.04E+05 | 6.79E-03 | 2.73E+00 | 0.00E+00 | 0.00E+00 |
| **1241** | 9.91E+05 | 0.00E+00 | 1.13E+08 | 1.06E+08 | 1.28E+06 | 1.46E+06 | 8.80E-03 | 7.73E-01 | 0.00E+00 | 0.00E+00 |
| **1242** | NA | NA | 1.08E+08 | 1.03E+08 | 1.18E+06 | 8.79E+06 | NA | NA | NA | NA |
| **1243** | NA | NA | 1.05E+08 | 1.14E+08 | 9.15E+05 | 6.60E+05 | NA | NA | NA | NA |
| **1244** | NA | NA | 1.19E+08 | 1.01E+08 | 5.34E+05 | 0.00E+00 | NA | NA | NA | NA |
| **1245** | NA | NA | 1.31E+08 | 8.73E+07 | 8.13E+05 | 3.68E+05 | NA | NA | NA | NA |
| **1246** | NA | NA | 9.91E+07 | 1.19E+08 | 4.86E+05 | 4.93E+05 | NA | NA | NA | NA |
| **1247** | NA | NA | 1.05E+08 | 1.14E+08 | 1.47E+05 | 0.00E+00 | NA | NA | NA | NA |
| **1248** | NA | NA | 1.80E+08 | 3.74E+07 | 7.35E+04 | 2.13E+05 | NA | NA | NA | NA |
| **1249** | NA | NA | 1.30E+08 | 8.78E+07 | NA | NA | NA | NA | NA | NA |
| **1250** | NA | NA | 1.64E+08 | 5.37E+07 | NA | NA | NA | NA | NA | NA |

| | | | | | | | | | | |
|---|---|---|---|---|---|---|---|---|---|---|
| **1251** | NA | NA | 1.34E+08 | 8.12E+07 | 4.43E+05 | 1.16E+05 | NA | NA | NA | NA |
| **1252** | NA | NA | 1.16E+08 | 9.95E+07 | NA | NA | NA | NA | NA | NA |
| **1253** | 1.63E+06 | 0.00E+00 | 1.13E+08 | 1.01E+08 | 9.36E+05 | 2.70E+05 | 1.45E-02 | 1.74E+00 | 0.00E+00 | 0.00E+00 |
| **1254** | 1.82E+05 | 6.31E+06 | 1.08E+08 | 1.05E+08 | 3.70E+05 | 9.73E+04 | 1.68E-03 | 4.93E-01 | 6.00E-02 | 6.49E+01 |
| **1255** | 0.00E+00 | 5.08E+05 | 9.25E+07 | 1.21E+08 | 2.56E+05 | 2.43E+05 | 0.00E+00 | 0.00E+00 | 4.21E-03 | 2.09E+00 |
| **1256** | 0.00E+00 | 8.88E+06 | 1.35E+08 | 7.42E+07 | 4.00E+05 | 1.10E+06 | 0.00E+00 | 0.00E+00 | 1.20E-01 | 8.09E+00 |
| **1257** | NA | NA | 9.48E+07 | 1.14E+08 | 5.29E+05 | 1.17E+06 | NA | NA | NA | NA |
| **1258** | NA | NA | 1.09E+08 | 1.02E+08 | NA | NA | NA | NA | NA | NA |
| **1259** | NA | NA | 1.79E+08 | 3.06E+07 | 8.26E+05 | 0.00E+00 | NA | NA | NA | NA |
| **1260** | NA | NA | 1.24E+08 | 8.42E+07 | 5.11E+05 | 2.90E+05 | NA | NA | NA | NA |
| **1261** | 1.62E+07 | 0.00E+00 | 1.29E+08 | 7.75E+07 | 8.34E+05 | 2.29E+06 | 1.26E-01 | 1.94E+01 | 0.00E+00 | 0.00E+00 |
| **1262** | NA | NA | 8.41E+07 | 1.23E+08 | 9.86E+05 | 2.05E+05 | NA | NA | NA | NA |
| **1263** | NA | NA | 1.13E+08 | 9.45E+07 | 7.92E+05 | 0.00E+00 | NA | NA | NA | NA |
| **1264** | NA | NA | 1.14E+08 | 9.42E+07 | NA | NA | NA | NA | NA | NA |
| **1265** | NA | NA | 1.36E+08 | 7.06E+07 | 3.38E+05 | 5.36E+05 | NA | NA | NA | NA |
| **1266** | NA | NA | 1.09E+08 | 9.60E+07 | 3.95E+05 | 2.38E+05 | NA | NA | NA | NA |
| **1267** | NA | NA | 1.41E+08 | 6.11E+07 | 7.02E+04 | 6.87E+04 | NA | NA | NA | NA |
| **1268** | NA | NA | 1.81E+08 | 2.02E+07 | 1.12E+06 | 1.80E+05 | NA | NA | NA | NA |
| **1269** | NA | NA | 1.44E+08 | 5.76E+07 | 6.24E+05 | 1.74E+05 | NA | NA | NA | NA |
| **1270** | NA | NA | 8.65E+07 | 1.14E+08 | 1.17E+06 | 0.00E+00 | NA | NA | NA | NA |
| **1271** | NA | NA | 1.25E+08 | 7.53E+07 | 4.75E+05 | 3.31E+05 | NA | NA | NA | NA |
| **1272** | NA | NA | 9.80E+07 | 9.97E+07 | 3.10E+06 | 7.96E+05 | NA | NA | NA | NA |
| **1273** | NA | NA | 1.39E+08 | 6.01E+07 | 8.79E+05 | 1.14E+06 | NA | NA | NA | NA |
| **1274** | 4.04E+06 | 2.90E+07 | 1.38E+08 | 6.14E+07 | 8.61E+05 | 6.22E+05 | 2.93E-02 | 4.68E+00 | 4.72E-01 | 4.66E+01 |
| **1275** | NA | NA | 1.09E+08 | 9.15E+07 | NA | NA | NA | NA | NA | NA |
| **1276** | NA | NA | 1.04E+08 | 9.10E+07 | 2.86E+06 | 2.08E+06 | NA | NA | NA | NA |
| **1277** | NA | NA | 1.27E+08 | 7.09E+07 | 2.40E+06 | 0.00E+00 | NA | NA | NA | NA |
| **1278** | 6.31E+06 | 0.00E+00 | 5.20E+07 | 1.47E+08 | NA | NA | 1.21E-01 | NA | 0.00E+00 | NA |
| **1279** | NA | NA | 1.43E+08 | 5.51E+07 | 5.96E+05 | 2.71E+05 | NA | NA | NA | NA |
| **1280** | NA | NA | 8.82E+07 | 1.09E+08 | 8.11E+04 | 1.27E+06 | NA | NA | NA | NA |
| **1281** | NA | NA | 1.06E+08 | 9.17E+07 | 6.97E+05 | 0.00E+00 | NA | NA | NA | NA |
| **1282** | NA | NA | 1.02E+08 | 9.64E+07 | 0.00E+00 | 6.31E+04 | NA | NA | NA | NA |
| **1283** | NA | NA | 8.26E+07 | 1.13E+08 | 8.14E+05 | 8.36E+04 | NA | NA | NA | NA |
| **1284** | NA | NA | 8.08E+07 | 1.15E+08 | NA | NA | NA | NA | NA | NA |
| **1285** | NA | NA | 9.15E+07 | 1.03E+08 | 7.65E+05 | 0.00E+00 | NA | NA | NA | NA |
| **1286** | NA | NA | 8.14E+07 | 1.13E+08 | 0.00E+00 | 2.22E+05 | NA | NA | NA | NA |
| **1287** | NA | NA | 1.01E+08 | 9.09E+07 | 9.60E+05 | 6.06E+05 | NA | NA | NA | NA |
| **1288** | NA | NA | 1.14E+08 | 7.77E+07 | 5.55E+05 | 5.19E+04 | NA | NA | NA | NA |

| | | | | | | | | | | |
|---|---|---|---|---|---|---|---|---|---|---|
| **1289** | NA | NA | 1.17E+08 | 7.32E+07 | 7.98E+05 | 9.57E+05 | NA | NA | NA | NA |
| **1290** | NA | NA | 1.03E+08 | 8.61E+07 | 2.15E+06 | 2.79E+05 | NA | NA | NA | NA |
| **1291** | 1.32E+07 | 1.89E+07 | 1.33E+08 | 5.77E+07 | 7.46E+05 | 0.00E+00 | 9.92E-02 | 1.77E+01 | 3.28E-01 | Inf |
| **1292** | NA | NA | 1.06E+08 | 8.37E+07 | 1.96E+05 | 1.30E+06 | NA | NA | NA | NA |
| **1293** | NA | NA | 1.14E+08 | 7.48E+07 | 9.07E+05 | 0.00E+00 | NA | NA | NA | NA |
| **1294** | NA | NA | 7.22E+07 | 1.16E+08 | 1.18E+06 | 2.31E+05 | NA | NA | NA | NA |
| **1295** | NA | NA | 9.61E+07 | 9.16E+07 | 1.18E+06 | 2.70E+05 | NA | NA | NA | NA |
| **1296** | NA | NA | 1.15E+08 | 7.40E+07 | NA | NA | NA | NA | NA | NA |
| **1297** | NA | NA | 9.56E+07 | 9.10E+07 | 0.00E+00 | 1.58E+05 | NA | NA | NA | NA |
| **1298** | NA | NA | 7.93E+07 | 1.06E+08 | 7.05E+05 | 0.00E+00 | NA | NA | NA | NA |
| **1299** | NA | NA | 1.20E+08 | 6.36E+07 | 1.03E+06 | 4.75E+05 | NA | NA | NA | NA |
| **1300** | NA | NA | 1.13E+08 | 7.01E+07 | 2.02E+06 | 0.00E+00 | NA | NA | NA | NA |
| **1301** | NA | NA | 1.00E+08 | 8.37E+07 | NA | NA | NA | NA | NA | NA |
| **1302** | NA | NA | 1.04E+08 | 7.86E+07 | 8.65E+05 | 4.30E+05 | NA | NA | NA | NA |
| **1303** | 1.65E+06 | 4.11E+06 | 6.72E+07 | 1.10E+08 | 2.64E+06 | 3.80E+06 | 2.46E-02 | 6.25E-01 | 3.74E-02 | 1.08E+00 |
| **1304** | NA | NA | 1.13E+08 | 7.02E+07 | 0.00E+00 | 0.00E+00 | NA | NA | NA | NA |
| **1305** | NA | NA | 1.25E+08 | 5.85E+07 | 0.00E+00 | 8.81E+04 | NA | NA | NA | NA |
| **1306** | NA | NA | 9.41E+07 | 8.82E+07 | 9.54E+04 | 0.00E+00 | NA | NA | NA | NA |
| **1307** | NA | NA | 8.65E+07 | 9.56E+07 | NA | NA | NA | NA | NA | NA |
| **1308** | 2.05E+05 | 0.00E+00 | 6.75E+07 | 1.13E+08 | 5.78E+05 | 6.85E+05 | 3.04E-03 | 3.55E-01 | 0.00E+00 | 0.00E+00 |
| **1309** | NA | NA | 1.05E+08 | 7.62E+07 | NA | NA | NA | NA | NA | NA |
| **1310** | NA | NA | 5.33E+07 | 1.26E+08 | 4.59E+05 | 5.76E+05 | NA | NA | NA | NA |
| **1311** | NA | NA | 9.61E+07 | 8.08E+07 | 1.24E+06 | 2.43E+06 | NA | NA | NA | NA |
| **1312** | 3.48E+06 | 0.00E+00 | 9.60E+07 | 8.17E+07 | 1.19E+06 | 1.57E+06 | 3.62E-02 | 2.93E+00 | 0.00E+00 | 0.00E+00 |
| **1313** | NA | NA | 9.83E+07 | 8.04E+07 | 9.80E+05 | 6.06E+05 | NA | NA | NA | NA |
| **1314** | 0.00E+00 | 0.00E+00 | 7.06E+07 | 1.08E+08 | 2.97E+05 | 3.88E+05 | 0.00E+00 | 0.00E+00 | 0.00E+00 | 0.00E+00 |
| **1315** | NA | NA | 8.21E+07 | 9.70E+07 | 0.00E+00 | 0.00E+00 | NA | NA | NA | NA |
| **1316** | NA | NA | 1.17E+08 | 5.97E+07 | 1.71E+06 | 9.81E+05 | NA | NA | NA | NA |
| **1317** | NA | NA | 9.62E+07 | 8.14E+07 | 7.67E+05 | 1.16E+05 | NA | NA | NA | NA |
| **1318** | NA | NA | 1.05E+08 | 7.32E+07 | NA | NA | NA | NA | NA | NA |
| **1319** | NA | NA | 1.09E+08 | 6.72E+07 | 6.95E+05 | 0.00E+00 | NA | NA | NA | NA |
| **1320** | NA | NA | 8.99E+07 | 8.68E+07 | NA | NA | NA | NA | NA | NA |
| **1321** | NA | NA | 1.13E+08 | 6.19E+07 | 0.00E+00 | 0.00E+00 | NA | NA | NA | NA |
| **1322** | NA | NA | 1.08E+08 | 6.72E+07 | NA | NA | NA | NA | NA | NA |
| **1323** | NA | NA | 1.01E+08 | 7.37E+07 | NA | NA | NA | NA | NA | NA |
| **1324** | NA | NA | 8.66E+07 | 8.84E+07 | NA | NA | NA | NA | NA | NA |
| **1325** | NA | NA | 6.89E+07 | 1.05E+08 | 1.00E+06 | 0.00E+00 | NA | NA | NA | NA |
| **1326** | NA | NA | 1.01E+08 | 7.27E+07 | 0.00E+00 | 1.27E+05 | NA | NA | NA | NA |

| | | | | | | | | | | |
|---|---|---|---|---|---|---|---|---|---|---|
| **1327** | NA | NA | 7.38E+07 | 9.84E+07 | 8.30E+05 | 0.00E+00 | NA | NA | NA | NA |
| **1328** | NA | NA | 9.21E+07 | 8.04E+07 | 4.74E+05 | 0.00E+00 | NA | NA | NA | NA |
| **1329** | 2.78E+06 | 2.71E+06 | 7.67E+07 | 9.57E+07 | NA | NA | 3.62E-02 | NA | 2.83E-02 | NA |
| **1330** | 0.00E+00 | 1.72E+08 | NA | NA | 0.00E+00 | 0.00E+00 | NA | NA | NA | Inf |
| **1331** | NA | NA | 9.70E+07 | 7.52E+07 | NA | NA | NA | NA | NA | NA |
| **1332** | NA | NA | 9.76E+07 | 7.43E+07 | NA | NA | NA | NA | NA | NA |
| **1333** | NA | NA | 1.09E+08 | 6.11E+07 | 7.91E+05 | 9.32E+05 | NA | NA | NA | NA |
| **1334** | NA | NA | 1.16E+08 | 5.58E+07 | NA | NA | NA | NA | NA | NA |
| **1335** | NA | NA | 7.80E+07 | 9.26E+07 | 0.00E+00 | 8.44E+05 | NA | NA | NA | NA |
| **1336** | NA | NA | 1.01E+08 | 6.78E+07 | 1.30E+06 | 9.39E+05 | NA | NA | NA | NA |
| **1337** | NA | NA | 9.19E+07 | 7.82E+07 | 9.70E+05 | 0.00E+00 | NA | NA | NA | NA |
| **1338** | NA | NA | 6.06E+07 | 1.07E+08 | 1.17E+06 | 1.46E+06 | NA | NA | NA | NA |
| **1339** | NA | NA | 1.01E+08 | 6.85E+07 | 8.66E+04 | 7.96E+04 | NA | NA | NA | NA |
| **1340** | 6.78E+06 | 4.71E+07 | 1.08E+08 | 5.99E+07 | 4.03E+05 | 6.63E+05 | 6.27E-02 | 1.68E+01 | 7.87E-01 | 7.10E+01 |
| **1341** | NA | NA | 6.73E+07 | 1.01E+08 | NA | NA | NA | NA | NA | NA |
| **1342** | 0.00E+00 | 0.00E+00 | 7.14E+07 | 9.67E+07 | 2.51E+05 | 0.00E+00 | 0.00E+00 | 0.00E+00 | 0.00E+00 | NA |
| **1343** | NA | NA | 9.19E+07 | 7.63E+07 | NA | NA | NA | NA | NA | NA |
| **1344** | NA | NA | 1.03E+08 | 6.40E+07 | 7.19E+05 | 0.00E+00 | NA | NA | NA | NA |
| **1345** | 2.97E+06 | 0.00E+00 | 9.32E+07 | 7.46E+07 | 1.56E+05 | 1.20E+05 | 3.19E-02 | 1.90E+01 | 0.00E+00 | 0.00E+00 |
| **1346** | NA | NA | 1.07E+08 | 6.01E+07 | NA | NA | NA | NA | NA | NA |
| **1347** | NA | NA | 7.46E+06 | 1.60E+08 | 0.00E+00 | 0.00E+00 | NA | NA | NA | NA |
| **1348** | NA | NA | 9.96E+07 | 6.65E+07 | 1.19E+05 | 7.43E+05 | NA | NA | NA | NA |
| **1349** | 3.27E+07 | 0.00E+00 | 6.59E+07 | 9.61E+07 | 1.94E+06 | 2.29E+06 | 4.97E-01 | 1.69E+01 | 0.00E+00 | 0.00E+00 |
| **1350** | NA | NA | 5.90E+07 | 1.06E+08 | 0.00E+00 | 1.16E+06 | NA | NA | NA | NA |
| **1351** | 0.00E+00 | 1.49E+07 | 1.09E+08 | 5.62E+07 | 9.17E+05 | 2.01E+05 | 0.00E+00 | 0.00E+00 | 2.66E-01 | 7.45E+01 |
| **1352** | NA | NA | 6.14E+07 | 1.02E+08 | 9.78E+05 | 9.17E+05 | NA | NA | NA | NA |
| **1353** | NA | NA | 1.10E+08 | 5.40E+07 | 4.96E+05 | 3.39E+05 | NA | NA | NA | NA |
| **1354** | NA | NA | 8.00E+07 | 8.22E+07 | 1.07E+06 | 9.17E+05 | NA | NA | NA | NA |
| **1355** | 4.00E+05 | 2.99E+05 | 8.29E+07 | 8.10E+07 | NA | NA | 4.82E-03 | NA | 3.69E-03 | NA |
| **1356** | NA | NA | 6.77E+07 | 9.50E+07 | 1.01E+06 | 0.00E+00 | NA | NA | NA | NA |
| **1357** | NA | NA | 8.42E+07 | 7.84E+07 | 8.64E+05 | 1.80E+05 | NA | NA | NA | NA |
| **1358** | NA | NA | 9.47E+07 | 6.85E+07 | 0.00E+00 | 0.00E+00 | NA | NA | NA | NA |
| **1359** | NA | NA | 6.57E+07 | 9.68E+07 | 5.07E+05 | 0.00E+00 | NA | NA | NA | NA |
| **1360** | NA | NA | 8.56E+07 | 7.63E+07 | 4.33E+05 | 9.33E+04 | NA | NA | NA | NA |
| **1361** | NA | NA | 7.44E+07 | 8.75E+07 | 2.45E+05 | 0.00E+00 | NA | NA | NA | NA |
| **1362** | 0.00E+00 | 8.44E+06 | 8.64E+07 | 6.87E+07 | 6.46E+06 | 1.73E+05 | 0.00E+00 | 0.00E+00 | 1.23E-01 | 4.87E+01 |
| **1363** | NA | NA | 8.08E+07 | 7.94E+07 | 5.76E+05 | 3.09E+05 | NA | NA | NA | NA |
| **1364** | NA | NA | 8.69E+07 | 7.28E+07 | 9.06E+05 | 2.57E+05 | NA | NA | NA | NA |

| | | | | | | | | | | |
|---|---|---|---|---|---|---|---|---|---|---|
| **1365** | NA | NA | 1.05E+08 | 5.53E+07 | NA | NA | NA | NA | NA | NA |
| **1366** | NA | NA | 1.02E+08 | 5.80E+07 | NA | NA | NA | NA | NA | NA |
| **1367** | NA | NA | 7.31E+07 | 8.54E+07 | 1.01E+06 | 3.82E+05 | NA | NA | NA | NA |
| **1368** | NA | NA | 7.89E+07 | 7.85E+07 | 8.57E+05 | 1.00E+06 | NA | NA | NA | NA |
| **1369** | 1.46E+07 | 0.00E+00 | 4.86E+07 | 1.08E+08 | 7.86E+05 | 1.51E+06 | 3.00E-01 | 1.86E+01 | 0.00E+00 | 0.00E+00 |
| **1370** | NA | NA | 7.28E+07 | 8.46E+07 | 8.14E+05 | 2.43E+05 | NA | NA | NA | NA |
| **1371** | 1.02E+07 | 0.00E+00 | 1.05E+08 | 5.00E+07 | 1.69E+06 | 4.82E+05 | 9.74E-02 | 6.06E+00 | 0.00E+00 | 0.00E+00 |
| **1372** | NA | NA | 9.20E+07 | 6.10E+07 | 2.44E+06 | 1.47E+06 | NA | NA | NA | NA |
| **1373** | NA | NA | 8.16E+07 | 7.49E+07 | 0.00E+00 | 0.00E+00 | NA | NA | NA | NA |
| **1374** | NA | NA | 5.64E+07 | 9.98E+07 | 0.00E+00 | 0.00E+00 | NA | NA | NA | NA |
| **1375** | NA | NA | 1.03E+08 | 5.18E+07 | 9.44E+05 | 0.00E+00 | NA | NA | NA | NA |
| **1376** | 3.71E+06 | 3.52E+05 | 6.98E+07 | 8.39E+07 | 5.95E+05 | 6.54E+05 | 5.32E-02 | 6.25E+00 | 4.20E-03 | 5.38E-01 |
| **1377** | NA | NA | 1.36E+08 | 1.85E+07 | 0.00E+00 | 0.00E+00 | NA | NA | NA | NA |
| **1378** | NA | NA | 1.03E+08 | 5.13E+07 | NA | NA | NA | NA | NA | NA |
| **1379** | 0.00E+00 | 2.32E+05 | 1.01E+08 | 4.96E+07 | 2.75E+06 | 0.00E+00 | 0.00E+00 | 0.00E+00 | 4.67E-03 | Inf |
| **1380** | 4.59E+06 | 2.55E+08 | 1.10E+08 | 4.25E+07 | 5.29E+05 | 7.40E+04 | 4.18E-02 | 8.68E+00 | 6.02E+00 | 3.45E+03 |
| **1381** | 6.92E+05 | 0.00E+00 | 4.98E+07 | 1.02E+08 | 2.42E+05 | 0.00E+00 | 1.39E-02 | 2.86E+00 | 0.00E+00 | NA |
| **1382** | 5.88E+06 | 0.00E+00 | 7.52E+07 | 7.53E+07 | 3.36E+05 | 1.41E+06 | 7.82E-02 | 1.75E+01 | 0.00E+00 | 0.00E+00 |
| **1383** | 0.00E+00 | 1.39E+07 | 1.04E+08 | 4.84E+07 | 0.00E+00 | 0.00E+00 | 0.00E+00 | NA | 2.87E-01 | Inf |
| **1384** | 0.00E+00 | 3.98E+05 | 9.34E+07 | 5.73E+07 | 4.88E+05 | 0.00E+00 | 0.00E+00 | 0.00E+00 | 6.94E-03 | Inf |
| **1385** | NA | NA | 7.50E+07 | 7.55E+07 | 5.54E+05 | 7.64E+04 | NA | NA | NA | NA |
| **1386** | NA | NA | 9.16E+07 | 5.83E+07 | 9.29E+05 | 0.00E+00 | NA | NA | NA | NA |
| **1387** | NA | NA | 8.18E+07 | 6.78E+07 | 1.05E+06 | 2.17E+05 | NA | NA | NA | NA |
| **1388** | NA | NA | 1.08E+08 | 4.28E+07 | 0.00E+00 | 2.78E+05 | NA | NA | NA | NA |
| **1389** | NA | NA | 7.93E+07 | 7.10E+07 | NA | NA | NA | NA | NA | NA |
| **1390** | NA | NA | 7.41E+07 | 7.51E+07 | 6.35E+05 | 2.83E+05 | NA | NA | NA | NA |
| **1391** | NA | NA | 9.29E+07 | 5.64E+07 | 2.73E+05 | 2.80E+05 | NA | NA | NA | NA |
| **1392** | 0.00E+00 | 1.30E+07 | 6.46E+07 | 8.43E+07 | 8.73E+05 | 0.00E+00 | 0.00E+00 | 0.00E+00 | 1.55E-01 | Inf |
| **1393** | NA | NA | 6.88E+07 | 7.93E+07 | 4.47E+05 | 6.82E+05 | NA | NA | NA | NA |
| **1394** | 0.00E+00 | 8.04E+05 | 8.92E+07 | 5.82E+07 | 4.74E+05 | 4.38E+05 | 0.00E+00 | 0.00E+00 | 1.38E-02 | 1.83E+00 |
| **1395** | 3.17E+06 | 6.47E+06 | 7.30E+07 | 7.52E+07 | 1.25E+05 | 0.00E+00 | 4.34E-02 | 2.53E+01 | 8.60E-02 | Inf |
| **1396** | 1.85E+07 | 0.00E+00 | 5.79E+07 | 8.92E+07 | 7.09E+05 | 2.52E+05 | 3.20E-01 | 2.62E+01 | 0.00E+00 | 0.00E+00 |
| **1397** | 1.58E+06 | 0.00E+00 | 9.53E+07 | 5.11E+07 | 9.93E+05 | 6.27E+05 | 1.66E-02 | 1.59E+00 | 0.00E+00 | 0.00E+00 |
| **1398** | NA | NA | 8.61E+07 | 6.15E+07 | NA | NA | NA | NA | NA | NA |
| **1399** | NA | NA | 7.46E+07 | 7.21E+07 | 4.91E+05 | 4.03E+05 | NA | NA | NA | NA |
| **1400** | 0.00E+00 | 1.13E+07 | 8.52E+07 | 5.54E+07 | 4.26E+06 | 1.11E+06 | 0.00E+00 | 0.00E+00 | 2.04E-01 | 1.02E+01 |
| **1401** | 0.00E+00 | 1.54E+06 | 8.48E+07 | 6.01E+07 | 2.98E+04 | 1.52E+05 | 0.00E+00 | 0.00E+00 | 2.56E-02 | 1.01E+01 |
| **1402** | NA | NA | 7.45E+07 | 6.90E+07 | 0.00E+00 | 6.01E+05 | NA | NA | NA | NA |

| | | | | | | | | | | |
|---|---|---|---|---|---|---|---|---|---|---|
| **1403** | NA | NA | 8.33E+07 | 5.96E+07 | 6.89E+05 | 4.21E+05 | NA | NA | NA | NA |
| **1404** | NA | NA | 7.30E+07 | 6.95E+07 | 8.60E+05 | 4.62E+05 | NA | NA | NA | NA |
| **1405** | NA | NA | 7.75E+07 | 6.45E+07 | 6.14E+05 | 2.47E+05 | NA | NA | NA | NA |
| **1406** | NA | NA | 7.53E+07 | 6.69E+07 | 2.73E+05 | 2.38E+05 | NA | NA | NA | NA |
| **1407** | 2.09E+06 | 0.00E+00 | 5.94E+07 | 8.20E+07 | 8.01E+05 | 1.18E+05 | 3.52E-02 | 2.61E+00 | 0.00E+00 | 0.00E+00 |
| **1408** | NA | NA | 5.62E+07 | 8.39E+07 | 1.79E+06 | 0.00E+00 | NA | NA | NA | NA |
| **1409** | NA | NA | 1.07E+08 | 3.40E+07 | 3.05E+05 | 7.13E+05 | NA | NA | NA | NA |
| **1410** | NA | NA | 8.49E+07 | 5.53E+07 | 4.80E+05 | 7.16E+05 | NA | NA | NA | NA |
| **1411** | NA | NA | 1.02E+08 | 3.83E+07 | NA | NA | NA | NA | NA | NA |
| **1412** | NA | NA | 7.74E+07 | 6.22E+07 | NA | NA | NA | NA | NA | NA |
| **1413** | 1.50E+06 | 4.59E+05 | 3.84E+07 | 9.95E+07 | 8.45E+05 | 6.29E+05 | 3.91E-02 | 1.78E+00 | 4.62E-03 | 7.31E-01 |
| **1414** | NA | NA | 8.70E+07 | 5.23E+07 | NA | NA | NA | NA | NA | NA |
| **1415** | NA | NA | 5.71E+07 | 7.96E+07 | 1.61E+06 | 8.30E+05 | NA | NA | NA | NA |
| **1416** | NA | NA | 5.25E+07 | 8.57E+07 | 7.07E+05 | 0.00E+00 | NA | NA | NA | NA |
| **1417** | 4.42E+06 | 0.00E+00 | 6.04E+07 | 7.57E+07 | 1.46E+06 | 5.93E+05 | 7.32E-02 | 3.03E+00 | 0.00E+00 | 0.00E+00 |
| **1418** | NA | NA | 8.27E+07 | 5.46E+07 | 7.36E+05 | 1.60E+05 | NA | NA | NA | NA |
| **1419** | NA | NA | 8.39E+07 | 5.41E+07 | NA | NA | NA | NA | NA | NA |
| **1420** | NA | NA | 7.21E+07 | 6.47E+07 | 2.17E+05 | 8.99E+05 | NA | NA | NA | NA |
| **1421** | NA | NA | 5.48E+07 | 8.20E+07 | 7.66E+05 | 0.00E+00 | NA | NA | NA | NA |
| **1422** | NA | NA | 6.72E+07 | 7.02E+07 | 0.00E+00 | 5.89E+04 | NA | NA | NA | NA |
| **1423** | NA | NA | 6.60E+07 | 7.07E+07 | 6.01E+05 | 0.00E+00 | NA | NA | NA | NA |
| **1424** | 2.67E+08 | 0.00E+00 | NA | NA | 1.03E+08 | 3.44E+07 | NA | 2.60E+00 | NA | 0.00E+00 |
| **1425** | NA | NA | 6.48E+07 | 7.21E+07 | 1.38E+05 | 9.14E+04 | NA | NA | NA | NA |
| **1426** | NA | NA | 7.56E+07 | 6.14E+07 | 0.00E+00 | 0.00E+00 | NA | NA | NA | NA |
| **1427** | NA | NA | 8.00E+07 | 5.67E+07 | NA | NA | NA | NA | NA | NA |
| **1428** | 0.00E+00 | 9.19E+07 | 6.68E+07 | 6.90E+07 | NA | NA | 0.00E+00 | NA | 1.33E+00 | NA |
| **1429** | NA | NA | 5.63E+07 | 7.79E+07 | NA | NA | NA | NA | NA | NA |
| **1430** | NA | NA | 7.29E+07 | 6.09E+07 | 0.00E+00 | 0.00E+00 | NA | NA | NA | NA |
| **1431** | NA | NA | 6.51E+07 | 6.69E+07 | 6.96E+05 | 0.00E+00 | NA | NA | NA | NA |
| **1432** | NA | NA | 7.56E+07 | 5.53E+07 | 7.18E+05 | 0.00E+00 | NA | NA | NA | NA |
| **1433** | NA | NA | 4.74E+07 | 8.41E+07 | NA | NA | NA | NA | NA | NA |
| **1434** | NA | NA | 5.72E+07 | 7.41E+07 | 0.00E+00 | 9.56E+04 | NA | NA | NA | NA |
| **1435** | NA | NA | 1.11E+08 | 2.00E+07 | NA | NA | NA | NA | NA | NA |
| **1436** | NA | NA | 8.03E+07 | 4.90E+07 | 3.62E+05 | 2.01E+05 | NA | NA | NA | NA |
| **1437** | NA | NA | 8.38E+07 | 4.46E+07 | 8.58E+04 | 2.72E+05 | NA | NA | NA | NA |
| **1438** | NA | NA | 9.78E+07 | 3.00E+07 | 3.47E+05 | 6.43E+05 | NA | NA | NA | NA |
| **1439** | NA | NA | 8.62E+07 | 4.22E+07 | 0.00E+00 | 0.00E+00 | NA | NA | NA | NA |
| **1440** | NA | NA | 6.94E+07 | 5.80E+07 | 0.00E+00 | 6.00E+05 | NA | NA | NA | NA |

| | | | | | | | | | | |
|---|---|---|---|---|---|---|---|---|---|---|
| **1441** | NA | NA | 6.61E+07 | 6.02E+07 | 1.24E+06 | 0.00E+00 | NA | NA | NA | NA |
| **1442** | NA | NA | 5.40E+07 | 7.13E+07 | 1.70E+06 | 2.64E+05 | NA | NA | NA | NA |
| **1443** | NA | NA | 8.38E+07 | 4.27E+07 | 0.00E+00 | 2.29E+05 | NA | NA | NA | NA |
| **1444** | NA | NA | 7.10E+07 | 5.44E+07 | 1.15E+06 | 7.29E+04 | NA | NA | NA | NA |
| **1445** | NA | NA | 7.72E+07 | 4.91E+07 | 0.00E+00 | 0.00E+00 | NA | NA | NA | NA |
| **1446** | NA | NA | 8.52E+07 | 3.97E+07 | 5.69E+05 | 0.00E+00 | NA | NA | NA | NA |
| **1447** | 0.00E+00 | 4.10E+06 | 7.31E+07 | 5.09E+07 | 1.28E+06 | 0.00E+00 | 0.00E+00 | 0.00E+00 | 8.04E-02 | Inf |
| **1448** | 1.58E+07 | 2.93E+06 | 6.66E+07 | 5.64E+07 | 1.44E+06 | 5.68E+05 | 2.37E-01 | 1.10E+01 | 5.19E-02 | 5.15E+00 |
| **1449** | NA | NA | 6.58E+07 | 5.90E+07 | 1.81E+05 | 0.00E+00 | NA | NA | NA | NA |
| **1450** | NA | NA | 6.53E+07 | 5.95E+07 | NA | NA | NA | NA | NA | NA |
| **1451** | NA | NA | 6.63E+07 | 5.83E+07 | 0.00E+00 | 0.00E+00 | NA | NA | NA | NA |
| **1452** | NA | NA | 5.96E+07 | 6.43E+07 | 4.09E+05 | 3.60E+04 | NA | NA | NA | NA |
| **1453** | NA | NA | 8.62E+07 | 3.70E+07 | 9.82E+05 | 0.00E+00 | NA | NA | NA | NA |
| **1454** | NA | NA | 5.82E+07 | 6.20E+07 | 3.04E+06 | 3.62E+05 | NA | NA | NA | NA |
| **1455** | NA | NA | 6.36E+07 | 5.96E+07 | 0.00E+00 | 3.56E+05 | NA | NA | NA | NA |
| **1456** | NA | NA | 8.40E+07 | 3.93E+07 | 0.00E+00 | 1.87E+05 | NA | NA | NA | NA |
| **1457** | NA | NA | 9.27E+07 | 3.02E+07 | NA | NA | NA | NA | NA | NA |
| **1458** | NA | NA | 5.28E+07 | 6.99E+07 | NA | NA | NA | NA | NA | NA |
| **1459** | NA | NA | 4.80E+07 | 7.39E+07 | 4.14E+05 | 7.87E+04 | NA | NA | NA | NA |
| **1460** | NA | NA | 6.73E+07 | 5.04E+07 | 2.21E+06 | 1.69E+06 | NA | NA | NA | NA |
| **1461** | NA | NA | 7.50E+07 | 4.38E+07 | 2.41E+06 | 4.60E+05 | NA | NA | NA | NA |
| **1462** | NA | NA | 7.20E+07 | 4.96E+07 | NA | NA | NA | NA | NA | NA |
| **1463** | NA | NA | 5.28E+07 | 6.62E+07 | 1.45E+06 | 4.34E+05 | NA | NA | NA | NA |
| **1464** | NA | NA | 6.25E+07 | 5.53E+07 | 1.27E+06 | 9.72E+05 | NA | NA | NA | NA |
| **1465** | NA | NA | 3.22E+07 | 8.57E+07 | 1.24E+06 | 0.00E+00 | NA | NA | NA | NA |
| **1466** | NA | NA | 9.62E+07 | 2.20E+07 | 2.95E+05 | 4.68E+05 | NA | NA | NA | NA |
| **1467** | 2.39E+06 | 0.00E+00 | 6.04E+07 | 5.67E+07 | 1.07E+06 | 3.74E+05 | 3.95E-02 | 2.23E+00 | 0.00E+00 | 0.00E+00 |
| **1468** | NA | NA | 6.74E+07 | 5.10E+07 | NA | NA | NA | NA | NA | NA |
| **1469** | 1.11E+06 | 1.03E+06 | 4.07E+07 | 7.47E+07 | 1.37E+06 | 1.43E+06 | 2.74E-02 | 8.15E-01 | 1.38E-02 | 7.18E-01 |
| **1470** | NA | NA | 6.30E+07 | 5.43E+07 | 5.35E+05 | 2.14E+05 | NA | NA | NA | NA |
| **1471** | 2.97E+05 | 0.00E+00 | 7.60E+07 | 4.04E+07 | 6.28E+05 | 9.66E+05 | 3.90E-03 | 4.72E-01 | 0.00E+00 | 0.00E+00 |
| **1472** | NA | NA | 5.25E+07 | 6.52E+07 | NA | NA | NA | NA | NA | NA |
| **1473** | 0.00E+00 | 1.17E+08 | NA | NA | 0.00E+00 | 0.00E+00 | NA | NA | NA | Inf |
| **1474** | NA | NA | 6.66E+07 | 4.94E+07 | NA | NA | NA | NA | NA | NA |
| **1475** | NA | NA | 7.96E+07 | 3.57E+07 | 4.13E+04 | 2.62E+05 | NA | NA | NA | NA |
| **1476** | NA | NA | 5.68E+07 | 5.84E+07 | 0.00E+00 | 1.75E+05 | NA | NA | NA | NA |
| **1477** | NA | NA | 6.90E+07 | 4.58E+07 | NA | NA | NA | NA | NA | NA |
| **1478** | NA | NA | 4.84E+07 | 6.51E+07 | 7.55E+05 | 0.00E+00 | NA | NA | NA | NA |

| | | | | | | | | | | |
|---|---|---|---|---|---|---|---|---|---|---|
| **1479** | NA | NA | 7.09E+07 | 4.14E+07 | 5.71E+05 | 1.35E+06 | NA | NA | NA | NA |
| **1480** | NA | NA | 5.90E+07 | 5.28E+07 | 1.21E+06 | 6.41E+05 | NA | NA | NA | NA |
| **1481** | NA | NA | 7.28E+07 | 3.76E+07 | 8.29E+05 | 1.41E+06 | NA | NA | NA | NA |
| **1482** | NA | NA | 5.64E+07 | 5.61E+07 | NA | NA | NA | NA | NA | NA |
| **1483** | NA | NA | 6.53E+07 | 4.69E+07 | 0.00E+00 | 1.81E+05 | NA | NA | NA | NA |
| **1484** | NA | NA | 5.24E+07 | 5.85E+07 | 3.41E+05 | 5.18E+05 | NA | NA | NA | NA |
| **1485** | NA | NA | 5.39E+07 | 5.69E+07 | 8.83E+05 | 0.00E+00 | NA | NA | NA | NA |
| **1486** | NA | NA | 4.39E+07 | 6.50E+07 | 8.61E+05 | 1.38E+06 | NA | NA | NA | NA |
| **1487** | NA | NA | 5.79E+07 | 5.28E+07 | NA | NA | NA | NA | NA | NA |
| **1488** | NA | NA | 5.41E+07 | 5.53E+07 | 5.39E+05 | 6.17E+05 | NA | NA | NA | NA |
| **1489** | NA | NA | 3.86E+07 | 6.98E+07 | 8.69E+05 | 8.93E+05 | NA | NA | NA | NA |
| **1490** | 7.80E+06 | 2.43E+06 | 4.52E+07 | 2.87E+07 | 1.54E+07 | 2.05E+07 | 1.73E-01 | 5.05E-01 | 8.47E-02 | 1.19E-01 |
| **1491** | 1.23E+07 | 0.00E+00 | 3.18E+07 | 7.49E+07 | 8.53E+05 | 1.07E+06 | 3.88E-01 | 1.45E+01 | 0.00E+00 | 0.00E+00 |
| **1492** | NA | NA | 3.66E+07 | 7.17E+07 | 0.00E+00 | 2.28E+05 | NA | NA | NA | NA |
| **1493** | NA | NA | 5.19E+07 | 5.51E+07 | 1.89E+05 | 3.12E+05 | NA | NA | NA | NA |
| **1494** | NA | NA | 4.41E+07 | 6.31E+07 | NA | NA | NA | NA | NA | NA |
| **1495** | NA | NA | 4.12E+07 | 6.55E+07 | 5.85E+04 | 3.64E+05 | NA | NA | NA | NA |
| **1496** | 2.82E+06 | 0.00E+00 | 5.80E+07 | 4.84E+07 | 2.11E+05 | 3.65E+05 | 4.87E-02 | 1.34E+01 | 0.00E+00 | 0.00E+00 |
| **1497** | NA | NA | 7.86E+07 | 2.78E+07 | 0.00E+00 | 4.20E+05 | NA | NA | NA | NA |
| **1498** | 1.13E+07 | 0.00E+00 | 4.74E+07 | 5.85E+07 | 5.91E+05 | 2.31E+05 | 2.38E-01 | 1.91E+01 | 0.00E+00 | 0.00E+00 |
| **1499** | 5.52E+05 | 0.00E+00 | 3.68E+07 | 6.75E+07 | 1.62E+06 | 6.69E+05 | 1.50E-02 | 3.41E-01 | 0.00E+00 | 0.00E+00 |
| **1500** | NA | NA | 6.35E+07 | 4.08E+07 | 1.64E+06 | 4.06E+05 | NA | NA | NA | NA |
| **1501** | 2.83E+06 | 3.07E+06 | 7.14E+07 | 3.12E+07 | 8.81E+05 | 2.52E+06 | 3.96E-02 | 3.21E+00 | 9.85E-02 | 1.22E+00 |
| **1502** | NA | NA | 4.44E+07 | 6.08E+07 | 5.34E+05 | 1.30E+05 | NA | NA | NA | NA |
| **1503** | 0.00E+00 | 1.89E+05 | 3.41E+07 | 6.79E+07 | 1.78E+06 | 9.76E+05 | 0.00E+00 | 0.00E+00 | 2.79E-03 | 1.94E-01 |
| **1504** | NA | NA | 4.98E+07 | 5.42E+07 | 3.60E+05 | 1.23E+05 | NA | NA | NA | NA |
| **1505** | NA | NA | 4.94E+07 | 5.44E+07 | 4.74E+05 | 0.00E+00 | NA | NA | NA | NA |
| **1506** | NA | NA | 4.86E+07 | 5.57E+07 | NA | NA | NA | NA | NA | NA |
| **1507** | NA | NA | 3.83E+07 | 6.50E+07 | 8.12E+05 | 1.74E+05 | NA | NA | NA | NA |
| **1508** | NA | NA | 5.55E+07 | 4.70E+07 | 2.34E+05 | 7.14E+05 | NA | NA | NA | NA |
| **1509** | NA | NA | 6.13E+07 | 4.20E+07 | NA | NA | NA | NA | NA | NA |
| **1510** | NA | NA | 2.49E+07 | 7.82E+07 | 6.87E+04 | 9.84E+04 | NA | NA | NA | NA |
| **1511** | NA | NA | 5.23E+07 | 5.03E+07 | 5.30E+05 | 0.00E+00 | NA | NA | NA | NA |
| **1512** | NA | NA | 4.14E+07 | 6.00E+07 | 6.20E+05 | 9.33E+05 | NA | NA | NA | NA |
| **1513** | NA | NA | 4.63E+07 | 5.60E+07 | 1.59E+05 | 2.03E+05 | NA | NA | NA | NA |
| **1514** | NA | NA | 5.84E+07 | 4.34E+07 | 0.00E+00 | 2.53E+05 | NA | NA | NA | NA |
| **1515** | NA | NA | 5.27E+07 | 4.89E+07 | NA | NA | NA | NA | NA | NA |
| **1516** | NA | NA | 3.08E+07 | 7.01E+07 | 5.48E+05 | 6.33E+04 | NA | NA | NA | NA |

| | | | | | | | | | | |
|---|---|---|---|---|---|---|---|---|---|---|
| **1517** | NA | NA | 7.14E+07 | 2.91E+07 | 6.04E+05 | 4.45E+05 | NA | NA | NA | NA |
| **1518** | NA | NA | 6.15E+07 | 3.95E+07 | 0.00E+00 | 3.09E+05 | NA | NA | NA | NA |
| **1519** | NA | NA | 5.26E+07 | 4.80E+07 | NA | NA | NA | NA | NA | NA |
| **1520** | NA | NA | 4.64E+07 | 5.29E+07 | 2.77E+05 | 0.00E+00 | NA | NA | NA | NA |
| **1521** | 0.00E+00 | 2.09E+06 | 5.65E+07 | 4.14E+07 | 1.52E+05 | 2.00E+05 | 0.00E+00 | 0.00E+00 | 5.04E-02 | 1.05E+01 |
| **1522** | NA | NA | 6.30E+07 | 3.43E+07 | 5.77E+05 | 0.00E+00 | NA | NA | NA | NA |
| **1523** | NA | NA | 4.45E+07 | 5.25E+07 | 4.59E+05 | 0.00E+00 | NA | NA | NA | NA |
| **1524** | NA | NA | 4.83E+07 | 4.85E+07 | NA | NA | NA | NA | NA | NA |
| **1525** | NA | NA | 4.23E+07 | 5.38E+07 | 0.00E+00 | 5.65E+05 | NA | NA | NA | NA |
| **1526** | NA | NA | 4.17E+07 | 5.05E+07 | 4.00E+06 | 2.53E+05 | NA | NA | NA | NA |
| **1527** | NA | NA | 5.39E+07 | 4.23E+07 | NA | NA | NA | NA | NA | NA |
| **1528** | NA | NA | 4.74E+07 | 4.73E+07 | 9.05E+05 | 6.20E+05 | NA | NA | NA | NA |
| **1529** | NA | NA | 5.48E+07 | 3.95E+07 | 1.48E+06 | 2.35E+05 | NA | NA | NA | NA |
| **1530** | NA | NA | 9.50E+07 | 0.00E+00 | NA | NA | NA | NA | NA | NA |
| **1531** | NA | NA | 5.33E+07 | 4.16E+07 | NA | NA | NA | NA | NA | NA |
| **1532** | NA | NA | 5.29E+07 | 4.17E+07 | NA | NA | NA | NA | NA | NA |
| **1533** | NA | NA | 5.60E+07 | 3.86E+07 | NA | NA | NA | NA | NA | NA |
| **1534** | NA | NA | 6.27E+07 | 3.12E+07 | 3.47E+05 | 1.72E+05 | NA | NA | NA | NA |
| **1535** | NA | NA | 4.69E+07 | 4.69E+07 | NA | NA | NA | NA | NA | NA |
| **1536** | NA | NA | 4.78E+07 | 2.34E+07 | 1.06E+07 | 1.18E+07 | NA | NA | NA | NA |
| **1537** | NA | NA | 4.39E+07 | 4.81E+07 | 7.87E+05 | 4.91E+05 | NA | NA | NA | NA |
| **1538** | NA | NA | 3.06E+07 | 6.25E+07 | 0.00E+00 | 0.00E+00 | NA | NA | NA | NA |
| **1539** | NA | NA | 3.71E+07 | 5.60E+07 | NA | NA | NA | NA | NA | NA |
| **1540** | NA | NA | 4.78E+07 | 4.44E+07 | 1.16E+05 | 7.37E+05 | NA | NA | NA | NA |
| **1541** | NA | NA | 6.00E+07 | 3.28E+07 | 0.00E+00 | 1.90E+05 | NA | NA | NA | NA |
| **1542** | NA | NA | 6.50E+07 | 2.79E+07 | NA | NA | NA | NA | NA | NA |
| **1543** | NA | NA | 5.02E+07 | 4.21E+07 | 5.82E+05 | 0.00E+00 | NA | NA | NA | NA |
| **1544** | NA | NA | 5.22E+07 | 3.92E+07 | 9.88E+05 | 4.50E+05 | NA | NA | NA | NA |
| **1545** | NA | NA | 5.64E+07 | 3.53E+07 | 1.99E+05 | 6.06E+05 | NA | NA | NA | NA |
| **1546** | 0.00E+00 | 4.14E+05 | 5.66E+07 | 3.57E+07 | NA | NA | 0.00E+00 | NA | 1.16E-02 | NA |
| **1547** | 7.25E+06 | 5.20E+06 | 5.25E+07 | 3.81E+07 | 4.02E+05 | 9.56E+05 | 1.38E-01 | 1.80E+01 | 1.36E-01 | 5.44E+00 |
| **1548** | NA | NA | 5.33E+07 | 3.71E+07 | 4.35E+05 | 1.67E+05 | NA | NA | NA | NA |
| **1549** | NA | NA | 3.20E+07 | 5.74E+07 | 0.00E+00 | 1.12E+06 | NA | NA | NA | NA |
| **1550** | NA | NA | 4.67E+07 | 4.36E+07 | NA | NA | NA | NA | NA | NA |
| **1551** | NA | NA | 2.22E+07 | 6.78E+07 | NA | NA | NA | NA | NA | NA |
| **1552** | NA | NA | 3.22E+07 | 5.66E+07 | 3.91E+05 | 7.45E+05 | NA | NA | NA | NA |
| **1553** | NA | NA | 5.03E+07 | 3.96E+07 | NA | NA | NA | NA | NA | NA |
| **1554** | NA | NA | 7.53E+07 | 1.28E+07 | 1.11E+06 | 3.55E+05 | NA | NA | NA | NA |

| | | | | | | | | | | |
|---|---|---|---|---|---|---|---|---|---|---|
| **1555** | NA | NA | 5.98E+07 | 2.97E+07 | 1.35E+05 | 0.00E+00 | NA | NA | NA | NA |
| **1556** | NA | NA | 5.37E+07 | 3.41E+07 | 6.99E+05 | 3.63E+05 | NA | NA | NA | NA |
| **1557** | NA | NA | NA | NA | 2.78E+07 | 6.07E+07 | NA | NA | NA | NA |
| **1558** | NA | NA | 6.63E+07 | 2.09E+07 | 3.85E+05 | 3.10E+05 | NA | NA | NA | NA |
| **1559** | NA | NA | 3.50E+07 | 5.27E+07 | NA | NA | NA | NA | NA | NA |
| **1560** | NA | NA | 4.85E+07 | 3.91E+07 | NA | NA | NA | NA | NA | NA |
| **1561** | NA | NA | 4.71E+07 | 4.04E+07 | NA | NA | NA | NA | NA | NA |
| **1562** | NA | NA | 5.66E+07 | 3.06E+07 | NA | NA | NA | NA | NA | NA |
| **1563** | NA | NA | 4.43E+07 | 4.28E+07 | 0.00E+00 | 0.00E+00 | NA | NA | NA | NA |
| **1564** | NA | NA | 6.93E+07 | 1.78E+07 | NA | NA | NA | NA | NA | NA |
| **1565** | NA | NA | 4.78E+07 | 3.89E+07 | 0.00E+00 | 0.00E+00 | NA | NA | NA | NA |
| **1566** | 2.57E+08 | 3.21E+08 | NA | NA | 5.05E+07 | 3.55E+07 | NA | 5.08E+00 | NA | 9.04E+00 |
| **1567** | NA | NA | 5.03E+07 | 3.54E+07 | 8.39E+04 | 0.00E+00 | NA | NA | NA | NA |
| **1568** | NA | NA | 5.22E+07 | 3.30E+07 | 0.00E+00 | 3.94E+05 | NA | NA | NA | NA |
| **1569** | NA | NA | 4.52E+07 | 3.81E+07 | 1.28E+06 | 7.26E+05 | NA | NA | NA | NA |
| **1570** | NA | NA | 4.97E+07 | 3.56E+07 | NA | NA | NA | NA | NA | NA |
| **1571** | NA | NA | 4.37E+07 | 4.06E+07 | NA | NA | NA | NA | NA | NA |
| **1572** | NA | NA | 5.11E+07 | 3.30E+07 | 5.48E+04 | 0.00E+00 | NA | NA | NA | NA |
| **1573** | NA | NA | 5.70E+07 | 2.69E+07 | NA | NA | NA | NA | NA | NA |
| **1574** | NA | NA | 4.99E+07 | 3.40E+07 | NA | NA | NA | NA | NA | NA |
| **1575** | NA | NA | 4.22E+07 | 4.13E+07 | NA | NA | NA | NA | NA | NA |
| **1576** | NA | NA | 2.31E+07 | 5.86E+07 | NA | NA | NA | NA | NA | NA |
| **1577** | NA | NA | 4.64E+07 | 3.46E+07 | 4.65E+05 | 0.00E+00 | NA | NA | NA | NA |
| **1578** | 0.00E+00 | 1.20E+06 | 1.34E+07 | 6.77E+07 | 0.00E+00 | 0.00E+00 | 0.00E+00 | NA | 1.78E-02 | Inf |
| **1579** | 0.00E+00 | 4.22E+06 | 3.98E+07 | 3.98E+07 | 1.37E+06 | 8.08E+04 | 0.00E+00 | 0.00E+00 | 1.06E-01 | 5.22E+01 |
| **1580** | NA | NA | 5.42E+07 | 2.61E+07 | 0.00E+00 | 0.00E+00 | NA | NA | NA | NA |
| **1581** | NA | NA | 5.89E+07 | 2.14E+07 | NA | NA | NA | NA | NA | NA |
| **1582** | 0.00E+00 | 4.97E+05 | 5.20E+07 | 2.60E+07 | 8.00E+05 | 4.09E+05 | 0.00E+00 | 0.00E+00 | 1.91E-02 | 1.22E+00 |
| **1583** | NA | NA | 4.03E+07 | 3.80E+07 | 3.88E+05 | 2.57E+05 | NA | NA | NA | NA |
| **1584** | NA | NA | 4.84E+07 | 2.93E+07 | 6.69E+05 | 1.65E+05 | NA | NA | NA | NA |
| **1585** | NA | NA | 4.52E+07 | 3.29E+07 | 9.27E+04 | 1.49E+05 | NA | NA | NA | NA |
| **1586** | NA | NA | 3.90E+07 | 3.84E+07 | 1.07E+05 | 5.75E+05 | NA | NA | NA | NA |
| **1587** | NA | NA | 6.02E+07 | 1.64E+07 | 0.00E+00 | 0.00E+00 | NA | NA | NA | NA |
| **1588** | NA | NA | 4.60E+07 | 3.06E+07 | NA | NA | NA | NA | NA | NA |
| **1589** | NA | NA | 3.48E+07 | 4.15E+07 | 3.21E+05 | 0.00E+00 | NA | NA | NA | NA |
| **1590** | NA | NA | 4.30E+07 | 3.31E+07 | NA | NA | NA | NA | NA | NA |
| **1591** | NA | NA | 3.99E+07 | 3.59E+07 | 0.00E+00 | 0.00E+00 | NA | NA | NA | NA |
| **1592** | NA | NA | 3.23E+07 | 4.31E+07 | 0.00E+00 | 7.14E+04 | NA | NA | NA | NA |

| | | | | | | | | | | |
|---|---|---|---|---|---|---|---|---|---|---|
| **1593** | NA | NA | 2.94E+07 | 4.58E+07 | NA | NA | NA | NA | NA | NA |
| **1594** | 0.00E+00 | 2.86E+06 | 3.87E+07 | 3.58E+07 | 6.31E+05 | 0.00E+00 | 0.00E+00 | 0.00E+00 | 7.99E-02 | Inf |
| **1595** | NA | NA | 4.28E+07 | 3.10E+07 | 5.20E+05 | 8.83E+05 | NA | NA | NA | NA |
| **1596** | NA | NA | 3.21E+07 | 4.28E+07 | 0.00E+00 | 9.83E+04 | NA | NA | NA | NA |
| **1597** | 2.46E+06 | 0.00E+00 | 5.64E+07 | 1.74E+07 | 6.37E+05 | 5.02E+05 | 4.36E-02 | 3.85E+00 | 0.00E+00 | 0.00E+00 |
| **1598** | NA | NA | 4.90E+07 | 2.47E+07 | 4.64E+05 | 6.90E+05 | NA | NA | NA | NA |
| **1599** | 1.78E+06 | 0.00E+00 | 2.77E+07 | 4.45E+07 | 8.68E+05 | 1.55E+06 | 6.43E-02 | 2.05E+00 | 0.00E+00 | 0.00E+00 |
| **1600** | NA | NA | 7.00E+07 | 4.14E+06 | NA | NA | NA | NA | NA | NA |
| **1601** | NA | NA | 4.91E+07 | 2.49E+07 | NA | NA | NA | NA | NA | NA |
| **1602** | 6.92E+06 | 1.69E+07 | 3.51E+07 | 3.73E+07 | 9.81E+05 | 5.05E+05 | 1.97E-01 | 7.06E+00 | 4.54E-01 | 3.36E+01 |
| **1603** | NA | NA | 4.57E+07 | 2.75E+07 | 5.63E+05 | 0.00E+00 | NA | NA | NA | NA |
| **1604** | NA | NA | 2.86E+07 | 4.47E+07 | 0.00E+00 | 1.13E+05 | NA | NA | NA | NA |
| **1605** | NA | NA | 1.89E+07 | 5.44E+07 | 1.08E+05 | 0.00E+00 | NA | NA | NA | NA |
| **1606** | NA | NA | 4.22E+07 | 3.08E+07 | NA | NA | NA | NA | NA | NA |
| **1607** | NA | NA | 5.70E+07 | 1.59E+07 | 0.00E+00 | 5.31E+04 | NA | NA | NA | NA |
| **1608** | NA | NA | 6.47E+07 | 7.68E+06 | NA | NA | NA | NA | NA | NA |
| **1609** | NA | NA | 2.81E+07 | 4.33E+07 | NA | NA | NA | NA | NA | NA |
| **1610** | NA | NA | 3.52E+07 | 1.99E+07 | 6.13E+06 | 1.00E+07 | NA | NA | NA | NA |
| **1611** | NA | NA | 3.44E+07 | 3.67E+07 | 0.00E+00 | 0.00E+00 | NA | NA | NA | NA |
| **1612** | NA | NA | 5.57E+07 | 1.39E+07 | 3.53E+05 | 1.01E+06 | NA | NA | NA | NA |
| **1613** | NA | NA | 5.95E+07 | 1.01E+07 | 0.00E+00 | 2.13E+05 | NA | NA | NA | NA |
| **1614** | NA | NA | 4.72E+07 | 2.21E+07 | NA | NA | NA | NA | NA | NA |
| **1615** | NA | NA | 4.18E+07 | 2.67E+07 | 6.05E+05 | 0.00E+00 | NA | NA | NA | NA |
| **1616** | NA | NA | 3.89E+07 | 2.98E+07 | 0.00E+00 | 2.32E+05 | NA | NA | NA | NA |
| **1617** | NA | NA | 2.73E+07 | 1.95E+07 | 1.14E+07 | 1.08E+07 | NA | NA | NA | NA |
| **1618** | NA | NA | 2.78E+07 | 4.08E+07 | 1.47E+05 | 7.28E+04 | NA | NA | NA | NA |
| **1619** | NA | NA | 3.29E+07 | 3.59E+07 | 0.00E+00 | 0.00E+00 | NA | NA | NA | NA |
| **1620** | NA | NA | 3.09E+07 | 3.72E+07 | 1.32E+05 | 5.22E+05 | NA | NA | NA | NA |
| **1621** | NA | NA | 1.61E+07 | 5.21E+07 | 2.61E+05 | 1.48E+05 | NA | NA | NA | NA |
| **1622** | 6.64E+06 | 6.10E+06 | 5.20E+07 | 1.49E+07 | 9.26E+05 | 7.19E+05 | 1.28E-01 | 7.17E+00 | 4.10E-01 | 8.49E+00 |
| **1623** | NA | NA | 3.43E+07 | 3.33E+07 | 0.00E+00 | 9.67E+05 | NA | NA | NA | NA |
| **1624** | 0.00E+00 | 1.29E+06 | 2.98E+07 | 3.86E+07 | NA | NA | 0.00E+00 | NA | 3.35E-02 | NA |
| **1625** | NA | NA | 3.22E+07 | 3.60E+07 | NA | NA | NA | NA | NA | NA |
| **1626** | NA | NA | 3.15E+07 | 3.67E+07 | NA | NA | NA | NA | NA | NA |
| **1627** | NA | NA | 4.93E+07 | 1.85E+07 | NA | NA | NA | NA | NA | NA |
| **1628** | NA | NA | 3.31E+07 | 3.47E+07 | NA | NA | NA | NA | NA | NA |
| **1629** | 6.48E+05 | 0.00E+00 | 3.84E+07 | 2.68E+07 | 1.39E+06 | 1.22E+06 | 1.69E-02 | 4.66E-01 | 0.00E+00 | 0.00E+00 |
| **1630** | NA | NA | 3.34E+07 | 3.44E+07 | NA | NA | NA | NA | NA | NA |

| | | | | | | | | | | |
|---|---|---|---|---|---|---|---|---|---|---|
| **1631** | NA | NA | 4.59E+07 | 2.08E+07 | 6.39E+05 | 4.28E+05 | NA | NA | NA | NA |
| **1632** | NA | NA | 4.96E+07 | 1.71E+07 | 6.39E+05 | 0.00E+00 | NA | NA | NA | NA |
| **1633** | 0.00E+00 | 0.00E+00 | 3.95E+07 | 2.73E+07 | 4.80E+05 | 0.00E+00 | 0.00E+00 | 0.00E+00 | 0.00E+00 | NA |
| **1634** | NA | NA | 4.35E+07 | 2.32E+07 | NA | NA | NA | NA | NA | NA |
| **1635** | 0.00E+00 | 5.63E+06 | 2.63E+07 | 3.98E+07 | 3.24E+05 | 0.00E+00 | 0.00E+00 | 0.00E+00 | 1.41E-01 | Inf |
| **1636** | NA | NA | 2.66E+07 | 3.97E+07 | 0.00E+00 | 0.00E+00 | NA | NA | NA | NA |
| **1637** | NA | NA | 5.43E+07 | 1.11E+07 | 0.00E+00 | 6.99E+05 | NA | NA | NA | NA |
| **1638** | NA | NA | 2.31E+07 | 4.27E+07 | NA | NA | NA | NA | NA | NA |
| **1639** | NA | NA | 3.33E+07 | 2.82E+07 | 8.63E+04 | 3.95E+06 | NA | NA | NA | NA |
| **1640** | NA | NA | 2.44E+07 | 4.11E+07 | NA | NA | NA | NA | NA | NA |
| **1641** | NA | NA | 4.92E+07 | 1.56E+07 | 2.89E+05 | 1.29E+05 | NA | NA | NA | NA |
| **1642** | NA | NA | 4.13E+07 | 2.33E+07 | 0.00E+00 | 3.34E+05 | NA | NA | NA | NA |
| **1643** | NA | NA | 3.88E+07 | 2.58E+07 | 4.25E+05 | 0.00E+00 | NA | NA | NA | NA |
| **1644** | 2.99E+06 | 6.61E+07 | 1.63E+07 | 4.78E+07 | 7.25E+05 | 0.00E+00 | 1.83E-01 | 4.12E+00 | 1.38E+00 | Inf |
| **1645** | 2.55E+06 | 0.00E+00 | 4.15E+07 | 2.25E+07 | 6.50E+05 | 4.64E+04 | 6.15E-02 | 3.93E+00 | 0.00E+00 | 0.00E+00 |
| **1646** | 1.16E+05 | 2.68E+06 | 0.00E+00 | 1.58E+07 | 3.05E+05 | 4.87E+07 | Inf | 3.80E-01 | 1.70E-01 | 5.52E-02 |
| **1647** | NA | NA | 4.94E+07 | 1.53E+07 | 0.00E+00 | 0.00E+00 | NA | NA | NA | NA |
| **1648** | NA | NA | 5.73E+07 | 7.28E+06 | NA | NA | NA | NA | NA | NA |
| **1649** | NA | NA | 2.52E+07 | 3.73E+07 | 1.27E+06 | 4.91E+05 | NA | NA | NA | NA |
| **1650** | NA | NA | 3.54E+07 | 2.88E+07 | NA | NA | NA | NA | NA | NA |
| **1651** | NA | NA | 4.18E+07 | 2.20E+07 | 1.92E+05 | 1.44E+05 | NA | NA | NA | NA |
| **1652** | NA | NA | 4.22E+07 | 2.02E+07 | 7.66E+05 | 5.45E+05 | NA | NA | NA | NA |
| **1653** | 1.85E+06 | 0.00E+00 | 3.38E+07 | 2.97E+07 | NA | NA | 5.48E-02 | NA | 0.00E+00 | NA |
| **1654** | NA | NA | 3.10E+07 | 3.19E+07 | 1.97E+05 | 0.00E+00 | NA | NA | NA | NA |
| **1655** | NA | NA | 2.70E+07 | 3.55E+07 | NA | NA | NA | NA | NA | NA |
| **1656** | 2.47E+07 | 0.00E+00 | 2.21E+07 | 3.75E+07 | 1.03E+06 | 1.66E+06 | 1.12E+00 | 2.39E+01 | 0.00E+00 | 0.00E+00 |
| **1657** | NA | NA | 4.36E+07 | 1.85E+07 | NA | NA | NA | NA | NA | NA |
| **1658** | NA | NA | 1.15E+07 | 5.04E+07 | 8.26E+04 | 0.00E+00 | NA | NA | NA | NA |
| **1659** | NA | NA | 3.35E+07 | 2.70E+07 | 6.77E+05 | 5.03E+05 | NA | NA | NA | NA |
| **1660** | 0.00E+00 | 6.58E+05 | 3.13E+07 | 2.97E+07 | 4.83E+05 | 1.46E+05 | 0.00E+00 | 0.00E+00 | 2.22E-02 | 4.51E+00 |
| **1661** | NA | NA | 2.22E+07 | 3.87E+07 | 0.00E+00 | 2.84E+05 | NA | NA | NA | NA |
| **1662** | NA | NA | 4.14E+07 | 1.89E+07 | 7.69E+05 | 0.00E+00 | NA | NA | NA | NA |
| **1663** | NA | NA | 3.29E+07 | 2.78E+07 | 0.00E+00 | 3.69E+04 | NA | NA | NA | NA |
| **1664** | 8.75E+06 | 0.00E+00 | 4.11E+07 | 1.81E+07 | 6.86E+05 | 8.48E+05 | 2.13E-01 | 1.28E+01 | 0.00E+00 | 0.00E+00 |
| **1665** | NA | NA | 2.08E+07 | 3.93E+07 | 4.14E+05 | 0.00E+00 | NA | NA | NA | NA |
| **1666** | NA | NA | 5.52E+07 | 5.25E+06 | NA | NA | NA | NA | NA | NA |
| **1667** | 6.44E+06 | 0.00E+00 | 2.03E+07 | 3.85E+07 | 1.25E+05 | 1.26E+06 | 3.18E-01 | 5.13E+01 | 0.00E+00 | 0.00E+00 |
| **1668** | NA | NA | 2.82E+07 | 3.12E+07 | 6.61E+04 | 3.30E+05 | NA | NA | NA | NA |

| | | | | | | | | | | |
|---|---|---|---|---|---|---|---|---|---|---|
| **1669** | NA | NA | 2.01E+07 | 3.93E+07 | NA | NA | NA | NA | NA | NA |
| **1670** | NA | NA | 5.05E+07 | 8.88E+06 | NA | NA | NA | NA | NA | NA |
| **1671** | 2.99E+06 | 0.00E+00 | 4.64E+07 | 1.26E+07 | 3.01E+05 | 0.00E+00 | 6.45E-02 | 9.93E+00 | 0.00E+00 | NA |
| **1672** | NA | NA | 3.21E+07 | 2.60E+07 | 8.28E+05 | 1.75E+05 | NA | NA | NA | NA |
| **1673** | 4.46E+06 | 0.00E+00 | 2.73E+07 | 3.06E+07 | 4.46E+05 | 6.92E+05 | 1.63E-01 | 1.00E+01 | 0.00E+00 | 0.00E+00 |
| **1674** | NA | NA | 4.31E+07 | 1.59E+07 | NA | NA | NA | NA | NA | NA |
| **1675** | NA | NA | 3.37E+07 | 2.49E+07 | 0.00E+00 | 1.99E+05 | NA | NA | NA | NA |
| **1676** | NA | NA | 2.30E+07 | 3.55E+07 | 0.00E+00 | 0.00E+00 | NA | NA | NA | NA |
| **1677** | NA | NA | 2.98E+07 | 2.81E+07 | 2.43E+05 | 1.83E+05 | NA | NA | NA | NA |
| **1678** | NA | NA | 4.29E+07 | 1.52E+07 | NA | NA | NA | NA | NA | NA |
| **1679** | NA | NA | 3.01E+07 | 2.69E+07 | 1.07E+06 | 0.00E+00 | NA | NA | NA | NA |
| **1680** | NA | NA | 3.90E+07 | 1.77E+07 | NA | NA | NA | NA | NA | NA |
| **1681** | NA | NA | 4.82E+07 | 8.44E+06 | 0.00E+00 | 0.00E+00 | NA | NA | NA | NA |
| **1682** | NA | NA | 2.24E+07 | 3.42E+07 | 0.00E+00 | 7.34E+04 | NA | NA | NA | NA |
| **1683** | NA | NA | 2.80E+07 | 2.81E+07 | 4.45E+05 | 0.00E+00 | NA | NA | NA | NA |
| **1684** | NA | NA | 2.98E+07 | 2.56E+07 | 4.16E+04 | 5.35E+05 | NA | NA | NA | NA |
| **1685** | NA | NA | 3.73E+07 | 1.78E+07 | 4.07E+05 | 2.59E+05 | NA | NA | NA | NA |
| **1686** | NA | NA | 3.37E+07 | 2.18E+07 | 0.00E+00 | 2.16E+05 | NA | NA | NA | NA |
| **1687** | NA | NA | 3.91E+07 | 1.65E+07 | 0.00E+00 | 0.00E+00 | NA | NA | NA | NA |
| **1688** | 0.00E+00 | 5.97E+07 | 3.70E+07 | 1.77E+07 | 0.00E+00 | 0.00E+00 | 0.00E+00 | NA | 3.37E+00 | Inf |
| **1689** | 2.00E+06 | 0.00E+00 | 4.52E+07 | 9.08E+06 | NA | NA | 4.43E-02 | NA | 0.00E+00 | NA |
| **1690** | NA | NA | 2.36E+07 | 2.99E+07 | 4.71E+05 | 0.00E+00 | NA | NA | NA | NA |
| **1691** | 7.26E+05 | 0.00E+00 | 3.75E+07 | 1.52E+07 | 5.77E+05 | 5.95E+05 | 1.93E-02 | 1.26E+00 | 0.00E+00 | 0.00E+00 |
| **1692** | NA | NA | 3.34E+07 | 2.00E+07 | 2.72E+05 | 8.45E+04 | NA | NA | NA | NA |
| **1693** | NA | NA | 3.05E+07 | 2.29E+07 | 0.00E+00 | 2.36E+05 | NA | NA | NA | NA |
| **1694** | 9.10E+06 | 5.90E+06 | 3.81E+07 | 1.42E+07 | 3.96E+05 | 7.70E+05 | 2.39E-01 | 2.30E+01 | 4.16E-01 | 7.67E+00 |
| **1695** | 1.21E+07 | 1.85E+08 | 1.06E+07 | 4.10E+07 | 4.63E+05 | 1.29E+06 | 1.14E+00 | 2.62E+01 | 4.50E+00 | 1.43E+02 |
| **1696** | 0.00E+00 | 2.82E+06 | 2.88E+07 | 2.44E+07 | 0.00E+00 | 1.42E+05 | 0.00E+00 | NA | 1.15E-01 | 1.99E+01 |
| **1697** | NA | NA | 2.46E+07 | 2.85E+07 | 0.00E+00 | 0.00E+00 | NA | NA | NA | NA |
| **1698** | NA | NA | 3.47E+07 | 1.77E+07 | 0.00E+00 | 6.92E+05 | NA | NA | NA | NA |
| **1699** | NA | NA | 4.52E+07 | 5.26E+06 | 1.74E+06 | 9.45E+05 | NA | NA | NA | NA |
| **1700** | NA | NA | 2.25E+07 | 3.04E+07 | 5.53E+04 | 1.34E+05 | NA | NA | NA | NA |
| **1701** | NA | NA | 3.30E+07 | 1.91E+07 | 3.35E+05 | 0.00E+00 | NA | NA | NA | NA |
| **1702** | NA | NA | 4.21E+07 | 1.01E+07 | NA | NA | NA | NA | NA | NA |
| **1703** | NA | NA | 2.98E+07 | 2.24E+07 | NA | NA | NA | NA | NA | NA |
| **1704** | NA | NA | 4.06E+07 | 1.05E+07 | 2.62E+05 | 8.28E+05 | NA | NA | NA | NA |
| **1705** | 5.79E+06 | 0.00E+00 | 2.27E+07 | 2.80E+07 | 9.11E+05 | 2.90E+05 | 2.55E-01 | 6.36E+00 | 0.00E+00 | 0.00E+00 |
| **1706** | NA | NA | 1.87E+07 | 3.32E+07 | 0.00E+00 | 9.28E+04 | NA | NA | NA | NA |

| | | | | | | | | | | |
|---|---|---|---|---|---|---|---|---|---|---|
| **1707** | NA | NA | 1.73E+07 | 3.37E+07 | 3.79E+05 | 1.68E+05 | NA | NA | NA | NA |
| **1708** | NA | NA | 0.00E+00 | 5.05E+07 | 8.48E+05 | 1.48E+05 | NA | NA | NA | NA |
| **1709** | NA | NA | 3.35E+07 | 1.75E+07 | 1.40E+05 | 0.00E+00 | NA | NA | NA | NA |
| **1710** | 1.59E+07 | 1.97E+07 | 1.39E+07 | 3.51E+07 | 1.66E+06 | 5.64E+05 | 1.14E+00 | 9.57E+00 | 5.61E-01 | 3.49E+01 |
| **1711** | 3.15E+05 | 4.14E+06 | 4.25E+07 | 8.42E+06 | 0.00E+00 | 0.00E+00 | 7.42E-03 | Inf | 4.91E-01 | Inf |
| **1712** | NA | NA | 3.60E+07 | 1.30E+07 | 1.16E+06 | 6.81E+05 | NA | NA | NA | NA |
| **1713** | 9.20E+06 | 0.00E+00 | 1.36E+07 | 3.56E+07 | 4.13E+05 | 4.84E+05 | 6.78E-01 | 2.23E+01 | 0.00E+00 | 0.00E+00 |
| **1714** | NA | NA | 2.45E+07 | 2.51E+07 | NA | NA | NA | NA | NA | NA |
| **1715** | 1.05E+06 | 0.00E+00 | 1.81E+07 | 3.14E+07 | 0.00E+00 | 0.00E+00 | 5.78E-02 | Inf | 0.00E+00 | NA |
| **1716** | NA | NA | 3.50E+07 | 1.34E+07 | 9.15E+05 | 0.00E+00 | NA | NA | NA | NA |
| **1717** | NA | NA | 3.46E+07 | 1.41E+07 | 0.00E+00 | 5.87E+05 | NA | NA | NA | NA |
| **1718** | NA | NA | 1.49E+07 | 3.41E+07 | 2.74E+05 | 0.00E+00 | NA | NA | NA | NA |
| **1719** | NA | NA | 4.01E+07 | 8.98E+06 | NA | NA | NA | NA | NA | NA |
| **1720** | NA | NA | 2.20E+07 | 2.69E+07 | NA | NA | NA | NA | NA | NA |
| **1721** | 1.63E+06 | 6.13E+05 | NA | NA | 1.85E+07 | 3.02E+07 | NA | 8.84E-02 | NA | 2.03E-02 |
| **1722** | NA | NA | 2.90E+07 | 1.81E+07 | 9.02E+05 | 3.90E+05 | NA | NA | NA | NA |
| **1723** | NA | NA | 1.82E+07 | 2.75E+07 | 1.44E+06 | 9.30E+05 | NA | NA | NA | NA |
| **1724** | NA | NA | 3.93E+07 | 8.26E+06 | 2.96E+05 | 0.00E+00 | NA | NA | NA | NA |
| **1725** | NA | NA | 1.59E+07 | 3.09E+07 | 8.08E+05 | 5.15E+04 | NA | NA | NA | NA |
| **1726** | 3.99E+06 | 0.00E+00 | 1.32E+07 | 3.45E+07 | NA | NA | 3.03E-01 | NA | 0.00E+00 | NA |
| **1727** | 0.00E+00 | 1.18E+05 | 2.37E+07 | 2.29E+07 | 7.67E+04 | 3.69E+05 | 0.00E+00 | 0.00E+00 | 5.14E-03 | 3.19E-01 |
| **1728** | 2.44E+07 | 0.00E+00 | NA | NA | 3.05E+07 | 1.65E+07 | NA | 7.99E-01 | NA | 0.00E+00 |
| **1729** | NA | NA | 3.53E+07 | 1.17E+07 | NA | NA | NA | NA | NA | NA |
| **1730** | NA | NA | 3.40E+07 | 1.26E+07 | 2.61E+05 | 0.00E+00 | NA | NA | NA | NA |
| **1731** | 0.00E+00 | 4.68E+07 | NA | NA | 0.00E+00 | 0.00E+00 | NA | NA | NA | Inf |
| **1732** | 0.00E+00 | 4.68E+07 | NA | NA | 0.00E+00 | 0.00E+00 | NA | NA | NA | Inf |
| **1733** | NA | NA | 1.88E+07 | 2.80E+07 | NA | NA | NA | NA | NA | NA |
| **1734** | 1.76E+06 | 0.00E+00 | 2.61E+07 | 2.02E+07 | 2.25E+05 | 1.01E+05 | 6.73E-02 | 7.82E+00 | 0.00E+00 | 0.00E+00 |
| **1735** | 1.89E+06 | 0.00E+00 | 2.54E+07 | 2.08E+07 | 2.55E+05 | 2.50E+05 | 7.44E-02 | 7.40E+00 | 0.00E+00 | 0.00E+00 |
| **1736** | 0.00E+00 | 1.30E+06 | 1.82E+07 | 2.84E+07 | 0.00E+00 | 0.00E+00 | 0.00E+00 | NA | 4.56E-02 | Inf |
| **1737** | NA | NA | 2.27E+07 | 2.36E+07 | NA | NA | NA | NA | NA | NA |
| **1738** | NA | NA | 1.18E+07 | 3.25E+07 | 1.16E+06 | 6.39E+05 | NA | NA | NA | NA |
| **1739** | NA | NA | 1.01E+07 | 4.04E+06 | 3.19E+07 | 0.00E+00 | NA | NA | NA | NA |
| **1740** | 2.42E+07 | 0.00E+00 | 9.92E+06 | 3.06E+07 | 3.09E+06 | 2.33E+06 | 2.43E+00 | 7.81E+00 | 0.00E+00 | 0.00E+00 |
| **1741** | NA | NA | 1.03E+07 | 3.51E+07 | 4.12E+05 | 0.00E+00 | NA | NA | NA | NA |
| **1742** | 5.25E+06 | 0.00E+00 | 2.73E+07 | 1.37E+07 | 3.85E+06 | 6.49E+05 | 1.93E-01 | 1.36E+00 | 0.00E+00 | 0.00E+00 |
| **1743** | 3.95E+06 | 0.00E+00 | 3.96E+07 | 5.60E+06 | NA | NA | 9.96E-02 | NA | 0.00E+00 | NA |
| **1744** | NA | NA | 1.64E+07 | 2.78E+07 | 9.63E+05 | 0.00E+00 | NA | NA | NA | NA |

| | | | | | | | | | | |
|---|---|---|---|---|---|---|---|---|---|---|
| **1745** | NA | NA | 3.69E+07 | 8.24E+06 | NA | NA | NA | NA | NA | NA |
| **1746** | NA | NA | 2.59E+07 | 1.90E+07 | NA | NA | NA | NA | NA | NA |
| **1747** | NA | NA | 2.74E+07 | 1.73E+07 | NA | NA | NA | NA | NA | NA |
| **1748** | NA | NA | 3.11E+07 | 1.36E+07 | 0.00E+00 | 0.00E+00 | NA | NA | NA | NA |
| **1749** | NA | NA | 0.00E+00 | 4.36E+07 | 2.15E+05 | 8.74E+04 | NA | NA | NA | NA |
| **1750** | NA | NA | 3.96E+06 | 3.50E+07 | 4.85E+06 | 0.00E+00 | NA | NA | NA | NA |
| **1751** | NA | NA | 2.25E+07 | 2.10E+07 | 0.00E+00 | 3.31E+05 | NA | NA | NA | NA |
| **1752** | NA | NA | 2.59E+07 | 1.74E+07 | 0.00E+00 | 3.89E+05 | NA | NA | NA | NA |
| **1753** | NA | NA | 3.75E+07 | 5.96E+06 | 0.00E+00 | 1.17E+05 | NA | NA | NA | NA |
| **1754** | NA | NA | 8.02E+06 | 3.46E+07 | 8.73E+05 | 0.00E+00 | NA | NA | NA | NA |
| **1755** | NA | NA | 3.12E+07 | 1.22E+07 | NA | NA | NA | NA | NA | NA |
| **1756** | NA | NA | 1.83E+07 | 2.44E+07 | 6.93E+05 | 5.18E+04 | NA | NA | NA | NA |
| **1757** | NA | NA | 2.22E+07 | 2.11E+07 | NA | NA | NA | NA | NA | NA |
| **1758** | NA | NA | 3.34E+07 | 9.82E+06 | NA | NA | NA | NA | NA | NA |
| **1759** | NA | NA | 2.83E+07 | 1.49E+07 | 0.00E+00 | 0.00E+00 | NA | NA | NA | NA |
| **1760** | 1.18E+07 | 5.29E+06 | 2.32E+07 | 1.86E+07 | 7.06E+04 | 1.09E+06 | 5.07E-01 | 1.67E+02 | 2.85E-01 | 4.84E+00 |
| **1761** | NA | NA | 3.02E+07 | 1.24E+07 | 0.00E+00 | 0.00E+00 | NA | NA | NA | NA |
| **1762** | NA | NA | 2.05E+07 | 2.19E+07 | NA | NA | NA | NA | NA | NA |
| **1763** | NA | NA | 2.78E+07 | 1.39E+07 | 3.97E+05 | 0.00E+00 | NA | NA | NA | NA |
| **1764** | NA | NA | 2.02E+07 | 2.17E+07 | NA | NA | NA | NA | NA | NA |
| **1765** | 1.38E+07 | 1.10E+07 | 3.29E+07 | 8.52E+06 | 4.20E+05 | 0.00E+00 | 4.21E-01 | 3.30E+01 | 1.29E+00 | Inf |
| **1766** | NA | NA | 1.94E+07 | 2.18E+07 | 3.42E+05 | 5.62E+04 | NA | NA | NA | NA |
| **1767** | NA | NA | 1.47E+07 | 2.66E+07 | NA | NA | NA | NA | NA | NA |
| **1768** | NA | NA | 2.06E+07 | 2.07E+07 | NA | NA | NA | NA | NA | NA |
| **1769** | NA | NA | 2.84E+07 | 1.29E+07 | 0.00E+00 | 0.00E+00 | NA | NA | NA | NA |
| **1770** | NA | NA | 1.87E+07 | 2.16E+07 | 9.77E+05 | 0.00E+00 | NA | NA | NA | NA |
| **1771** | NA | NA | 3.15E+07 | 9.64E+06 | NA | NA | NA | NA | NA | NA |
| **1772** | 0.00E+00 | 2.23E+06 | 8.16E+06 | 3.21E+07 | 4.76E+05 | 2.66E+05 | 0.00E+00 | 0.00E+00 | 6.94E-02 | 8.40E+00 |
| **1773** | 4.34E+06 | 3.55E+05 | 1.46E+07 | 2.54E+07 | 3.86E+05 | 3.06E+05 | 2.98E-01 | 1.13E+01 | 1.39E-02 | 1.16E+00 |
| **1774** | NA | NA | 2.71E+07 | 1.36E+07 | NA | NA | NA | NA | NA | NA |
| **1775** | NA | NA | 2.30E+07 | 1.77E+07 | NA | NA | NA | NA | NA | NA |
| **1776** | NA | NA | 8.90E+06 | 3.17E+07 | NA | NA | NA | NA | NA | NA |
| **1777** | NA | NA | 3.28E+07 | 7.48E+06 | 2.01E+05 | 0.00E+00 | NA | NA | NA | NA |
| **1778** | NA | NA | 3.07E+07 | 9.58E+06 | 0.00E+00 | 0.00E+00 | NA | NA | NA | NA |
| **1779** | NA | NA | 2.30E+07 | 1.67E+07 | 3.74E+05 | 0.00E+00 | NA | NA | NA | NA |
| **1780** | NA | NA | 1.91E+07 | 1.91E+07 | 6.94E+05 | 5.05E+05 | NA | NA | NA | NA |
| **1781** | NA | NA | NA | NA | 1.72E+07 | 2.18E+07 | NA | NA | NA | NA |
| **1782** | NA | NA | 3.11E+07 | 7.60E+06 | 0.00E+00 | 0.00E+00 | NA | NA | NA | NA |

| | | | | | | | | | | |
|---|---|---|---|---|---|---|---|---|---|---|
| **1783** | NA | NA | 2.70E+07 | 1.16E+07 | NA | NA | NA | NA | NA | NA |
| **1784** | NA | NA | 7.50E+06 | 3.01E+07 | 9.05E+05 | 6.40E+04 | NA | NA | NA | NA |
| **1785** | NA | NA | 1.91E+07 | 1.91E+07 | 0.00E+00 | 3.19E+05 | NA | NA | NA | NA |
| **1786** | 8.13E+06 | 2.42E+07 | 2.95E+07 | 8.38E+06 | 4.27E+05 | 2.66E+05 | 2.76E-01 | 1.90E+01 | 2.89E+00 | 9.08E+01 |
| **1787** | 5.12E+06 | 9.87E+06 | 3.33E+07 | 4.96E+06 | 0.00E+00 | 0.00E+00 | 1.54E-01 | Inf | 1.99E+00 | Inf |
| **1788** | 7.79E+07 | 3.03E+07 | 9.28E+06 | 2.72E+07 | 6.45E+04 | 1.58E+06 | 8.40E+00 | 1.21E+03 | 1.11E+00 | 1.92E+01 |
| **1789** | NA | NA | 2.10E+07 | 1.53E+07 | 4.11E+05 | 1.20E+06 | NA | NA | NA | NA |
| **1790** | NA | NA | 2.79E+07 | 9.86E+06 | NA | NA | NA | NA | NA | NA |
| **1791** | NA | NA | 1.63E+07 | 2.04E+07 | 2.25E+05 | 5.09E+05 | NA | NA | NA | NA |
| **1792** | NA | NA | 1.06E+07 | 2.68E+07 | NA | NA | NA | NA | NA | NA |
| **1793** | 0.00E+00 | 3.73E+07 | NA | NA | 0.00E+00 | 0.00E+00 | NA | NA | NA | Inf |
| **1794** | 0.00E+00 | 3.73E+07 | NA | NA | 0.00E+00 | 0.00E+00 | NA | NA | NA | Inf |
| **1795** | NA | NA | 1.41E+07 | 2.06E+07 | 0.00E+00 | 2.60E+06 | NA | NA | NA | NA |
| **1796** | NA | NA | 1.33E+07 | 2.38E+07 | NA | NA | NA | NA | NA | NA |
| **1797** | NA | NA | 1.98E+07 | 1.73E+07 | NA | NA | NA | NA | NA | NA |
| **1798** | NA | NA | 1.59E+07 | 2.01E+07 | 7.43E+05 | 2.85E+05 | NA | NA | NA | NA |
| **1799** | NA | NA | 8.48E+06 | 2.75E+07 | 7.14E+05 | 0.00E+00 | NA | NA | NA | NA |
| **1800** | NA | NA | 2.78E+07 | 8.60E+06 | NA | NA | NA | NA | NA | NA |
| **1801** | NA | NA | 1.04E+07 | 2.59E+07 | NA | NA | NA | NA | NA | NA |
| **1802** | NA | NA | 1.87E+07 | 1.74E+07 | 0.00E+00 | 1.75E+05 | NA | NA | NA | NA |
| **1803** | NA | NA | 1.17E+07 | 2.43E+07 | NA | NA | NA | NA | NA | NA |
| **1804** | NA | NA | 1.40E+07 | 2.19E+07 | 0.00E+00 | 5.41E+04 | NA | NA | NA | NA |
| **1805** | 6.97E+06 | 2.95E+05 | 2.74E+07 | 7.12E+06 | 6.75E+05 | 6.98E+05 | 2.54E-01 | 1.03E+01 | 4.14E-02 | 4.23E-01 |
| **1806** | NA | NA | 1.63E+07 | 1.94E+07 | 2.79E+04 | 0.00E+00 | NA | NA | NA | NA |
| **1807** | NA | NA | 1.88E+07 | 1.68E+07 | NA | NA | NA | NA | NA | NA |
| **1808** | 9.73E+05 | 7.51E+07 | 2.63E+07 | 9.14E+06 | 3.71E+04 | 0.00E+00 | 3.70E-02 | 2.62E+01 | 8.22E+00 | Inf |
| **1809** | NA | NA | 1.43E+07 | 2.08E+07 | NA | NA | NA | NA | NA | NA |
| **1810** | NA | NA | 1.72E+07 | 1.73E+07 | 5.13E+05 | 0.00E+00 | NA | NA | NA | NA |
| **1811** | NA | NA | 2.40E+07 | 9.92E+06 | 9.16E+05 | 1.04E+05 | NA | NA | NA | NA |
| **1812** | NA | NA | 1.71E+07 | 1.77E+07 | NA | NA | NA | NA | NA | NA |
| **1813** | NA | NA | 3.46E+07 | 0.00E+00 | NA | NA | NA | NA | NA | NA |
| **1814** | NA | NA | 2.58E+07 | 8.54E+06 | 1.70E+05 | 0.00E+00 | NA | NA | NA | NA |
| **1815** | 0.00E+00 | 3.43E+07 | NA | NA | NA | NA | NA | NA | NA | NA |
| **1816** | NA | NA | 1.86E+07 | 1.56E+07 | NA | NA | NA | NA | NA | NA |
| **1817** | NA | NA | 2.44E+07 | 9.74E+06 | NA | NA | NA | NA | NA | NA |
| **1818** | NA | NA | 3.12E+07 | 2.66E+06 | 0.00E+00 | 0.00E+00 | NA | NA | NA | NA |
| **1819** | NA | NA | 2.28E+07 | 1.06E+07 | NA | NA | NA | NA | NA | NA |
| **1820** | NA | NA | 2.61E+07 | 6.88E+06 | 0.00E+00 | 2.45E+05 | NA | NA | NA | NA |

| | | | | | | | | | | |
|---|---|---|---|---|---|---|---|---|---|---|
| **1821** | NA | NA | 2.43E+07 | 8.86E+06 | NA | NA | NA | NA | NA | NA |
| **1822** | NA | NA | 1.23E+07 | 2.08E+07 | NA | NA | NA | NA | NA | NA |
| **1823** | NA | NA | 1.17E+07 | 2.08E+07 | 4.19E+05 | 0.00E+00 | NA | NA | NA | NA |
| **1824** | NA | NA | 1.23E+07 | 2.01E+07 | 1.10E+05 | 1.26E+05 | NA | NA | NA | NA |
| **1825** | NA | NA | 1.30E+07 | 1.92E+07 | NA | NA | NA | NA | NA | NA |
| **1826** | NA | NA | 1.58E+07 | 1.57E+07 | 2.91E+05 | 2.77E+05 | NA | NA | NA | NA |
| **1827** | NA | NA | 2.62E+07 | 5.16E+06 | 0.00E+00 | 6.39E+05 | NA | NA | NA | NA |
| **1828** | NA | NA | 2.56E+07 | 5.84E+06 | 3.70E+05 | 0.00E+00 | NA | NA | NA | NA |
| **1829** | NA | NA | 1.50E+07 | 1.68E+07 | NA | NA | NA | NA | NA | NA |
| **1830** | NA | NA | 2.24E+07 | 7.80E+06 | 8.35E+05 | 7.67E+05 | NA | NA | NA | NA |
| **1831** | NA | NA | 2.35E+07 | 8.26E+06 | NA | NA | NA | NA | NA | NA |
| **1832** | NA | NA | 1.50E+07 | 1.66E+07 | NA | NA | NA | NA | NA | NA |
| **1833** | NA | NA | 1.95E+07 | 1.19E+07 | NA | NA | NA | NA | NA | NA |
| **1834** | NA | NA | 2.68E+07 | 4.20E+06 | 3.67E+05 | 9.52E+04 | NA | NA | NA | NA |
| **1835** | NA | NA | 9.40E+06 | 2.11E+07 | 6.30E+05 | 1.62E+05 | NA | NA | NA | NA |
| **1836** | NA | NA | 1.78E+07 | 1.33E+07 | NA | NA | NA | NA | NA | NA |
| **1837** | NA | NA | 1.76E+07 | 1.30E+07 | 0.00E+00 | 1.86E+05 | NA | NA | NA | NA |
| **1838** | NA | NA | 2.19E+07 | 8.88E+06 | NA | NA | NA | NA | NA | NA |
| **1839** | NA | NA | 1.07E+07 | 2.00E+07 | NA | NA | NA | NA | NA | NA |
| **1840** | NA | NA | 2.43E+07 | 5.52E+06 | 3.99E+05 | 4.65E+05 | NA | NA | NA | NA |
| **1841** | 0.00E+00 | 6.59E+05 | 2.42E+07 | 6.20E+06 | 0.00E+00 | 1.31E+05 | 0.00E+00 | NA | 1.06E-01 | 5.02E+00 |
| **1842** | NA | NA | 2.07E+07 | 9.44E+06 | 4.16E+05 | 0.00E+00 | NA | NA | NA | NA |
| **1843** | 0.00E+00 | 3.05E+07 | NA | NA | NA | NA | NA | NA | NA | NA |
| **1844** | NA | NA | 2.14E+07 | 9.10E+06 | NA | NA | NA | NA | NA | NA |
| **1845** | NA | NA | 2.04E+07 | 9.90E+06 | 0.00E+00 | 0.00E+00 | NA | NA | NA | NA |
| **1846** | NA | NA | 2.98E+07 | 0.00E+00 | 1.47E+05 | 5.78E+04 | NA | NA | NA | NA |
| **1847** | NA | NA | 1.23E+07 | 1.76E+07 | NA | NA | NA | NA | NA | NA |
| **1848** | NA | NA | 2.96E+07 | 0.00E+00 | NA | NA | NA | NA | NA | NA |
| **1849** | 4.82E+06 | 0.00E+00 | 2.37E+07 | 5.66E+06 | 0.00E+00 | 2.21E+05 | 2.03E-01 | Inf | 0.00E+00 | 0.00E+00 |
| **1850** | NA | NA | 1.60E+07 | 1.31E+07 | 0.00E+00 | 0.00E+00 | NA | NA | NA | NA |
| **1851** | NA | NA | 1.23E+07 | 1.68E+07 | NA | NA | NA | NA | NA | NA |
| **1852** | NA | NA | 6.50E+06 | 2.25E+07 | 0.00E+00 | 0.00E+00 | NA | NA | NA | NA |
| **1853** | NA | NA | 1.75E+07 | 1.11E+07 | 1.25E+05 | 2.87E+05 | NA | NA | NA | NA |
| **1854** | NA | NA | 0.00E+00 | 2.88E+07 | 1.45E+05 | 0.00E+00 | NA | NA | NA | NA |
| **1855** | 0.00E+00 | 1.13E+06 | 1.82E+07 | 1.04E+07 | 0.00E+00 | 0.00E+00 | 0.00E+00 | NA | 1.08E-01 | Inf |
| **1856** | NA | NA | 2.27E+07 | 5.68E+06 | NA | NA | NA | NA | NA | NA |
| **1857** | 3.23E+06 | 0.00E+00 | 1.74E+07 | 1.09E+07 | 0.00E+00 | 7.97E+04 | 1.86E-01 | Inf | 0.00E+00 | 0.00E+00 |
| **1858** | NA | NA | 1.15E+07 | 1.48E+07 | 6.73E+05 | 1.25E+06 | NA | NA | NA | NA |

| | | | | | | | | | | |
|---|---|---|---|---|---|---|---|---|---|---|
| **1859** | NA | NA | 1.76E+07 | 1.02E+07 | 1.24E+05 | 1.16E+05 | NA | NA | NA | NA |
| **1860** | NA | NA | 1.77E+07 | 1.03E+07 | NA | NA | NA | NA | NA | NA |
| **1861** | NA | NA | 8.18E+06 | 1.97E+07 | NA | NA | NA | NA | NA | NA |
| **1862** | NA | NA | 2.79E+07 | 0.00E+00 | NA | NA | NA | NA | NA | NA |
| **1863** | NA | NA | 0.00E+00 | 2.78E+07 | NA | NA | NA | NA | NA | NA |
| **1864** | NA | NA | 2.67E+07 | 0.00E+00 | 2.55E+05 | 6.29E+05 | NA | NA | NA | NA |
| **1865** | 0.00E+00 | 8.39E+06 | 2.30E+07 | 3.96E+06 | 5.58E+05 | 0.00E+00 | 0.00E+00 | 0.00E+00 | 2.12E+00 | Inf |
| **1866** | NA | NA | 1.84E+07 | 8.36E+06 | 7.89E+05 | 0.00E+00 | NA | NA | NA | NA |
| **1867** | 4.61E+06 | 0.00E+00 | NA | NA | 1.26E+07 | 1.49E+07 | NA | 3.64E-01 | NA | 0.00E+00 |
| **1868** | NA | NA | 7.68E+06 | 1.96E+07 | 5.15E+04 | 1.51E+05 | NA | NA | NA | NA |
| **1869** | NA | NA | 1.70E+07 | 1.02E+07 | 0.00E+00 | 1.26E+05 | NA | NA | NA | NA |
| **1870** | NA | NA | 1.88E+07 | 8.28E+06 | 1.83E+05 | 0.00E+00 | NA | NA | NA | NA |
| **1871** | NA | NA | 1.70E+07 | 9.96E+06 | NA | NA | NA | NA | NA | NA |
| **1872** | 2.14E+06 | 0.00E+00 | 1.28E+07 | 1.30E+07 | 5.91E+05 | 4.27E+05 | 1.67E-01 | 3.63E+00 | 0.00E+00 | 0.00E+00 |
| **1873** | NA | NA | 1.81E+07 | 7.66E+06 | 6.01E+05 | 0.00E+00 | NA | NA | NA | NA |
| **1874** | 2.48E+06 | 0.00E+00 | 0.00E+00 | 2.62E+07 | 0.00E+00 | 1.97E+05 | Inf | Inf | 0.00E+00 | 0.00E+00 |
| **1875** | NA | NA | 7.32E+06 | 1.85E+07 | 1.39E+05 | 3.95E+05 | NA | NA | NA | NA |
| **1876** | NA | NA | 1.50E+07 | 1.13E+07 | NA | NA | NA | NA | NA | NA |
| **1877** | NA | NA | 1.14E+07 | 1.48E+07 | 0.00E+00 | 0.00E+00 | NA | NA | NA | NA |
| **1878** | NA | NA | 1.47E+07 | 1.15E+07 | NA | NA | NA | NA | NA | NA |
| **1879** | NA | NA | 1.07E+07 | 1.54E+07 | 7.80E+04 | 0.00E+00 | NA | NA | NA | NA |
| **1880** | NA | NA | 2.07E+07 | 5.30E+06 | NA | NA | NA | NA | NA | NA |
| **1881** | NA | NA | 2.59E+07 | 0.00E+00 | NA | NA | NA | NA | NA | NA |
| **1882** | NA | NA | 2.23E+07 | 3.30E+06 | 0.00E+00 | 2.97E+05 | NA | NA | NA | NA |
| **1883** | 0.00E+00 | 1.02E+06 | 1.50E+07 | 1.08E+07 | NA | NA | 0.00E+00 | NA | 9.44E-02 | NA |
| **1884** | NA | NA | 7.90E+06 | 1.63E+07 | 9.60E+05 | 4.90E+05 | NA | NA | NA | NA |
| **1885** | NA | NA | 1.15E+07 | 1.36E+07 | 3.15E+05 | 0.00E+00 | NA | NA | NA | NA |
| **1886** | NA | NA | 1.08E+07 | 1.46E+07 | NA | NA | NA | NA | NA | NA |
| **1887** | NA | NA | 1.48E+07 | 1.05E+07 | NA | NA | NA | NA | NA | NA |
| **1888** | NA | NA | 1.04E+07 | 1.46E+07 | NA | NA | NA | NA | NA | NA |
| **1889** | NA | NA | 1.22E+07 | 1.22E+07 | 1.72E+05 | 3.27E+05 | NA | NA | NA | NA |
| **1890** | 7.32E+06 | 0.00E+00 | 5.30E+06 | 1.84E+07 | 4.98E+05 | 6.53E+05 | 1.38E+00 | 1.47E+01 | 0.00E+00 | 0.00E+00 |
| **1891** | NA | NA | 1.87E+07 | 6.12E+06 | NA | NA | NA | NA | NA | NA |
| **1892** | NA | NA | 2.62E+06 | 2.20E+07 | NA | NA | NA | NA | NA | NA |
| **1893** | NA | NA | 1.34E+07 | 1.03E+07 | 5.51E+05 | 0.00E+00 | NA | NA | NA | NA |
| **1894** | NA | NA | 1.99E+07 | 4.42E+06 | 0.00E+00 | 0.00E+00 | NA | NA | NA | NA |
| **1895** | NA | NA | 1.36E+07 | 1.04E+07 | 1.40E+05 | 0.00E+00 | NA | NA | NA | NA |
| **1896** | NA | NA | 2.07E+07 | 3.32E+06 | NA | NA | NA | NA | NA | NA |

| | | | | | | | | | | |
|---|---|---|---|---|---|---|---|---|---|---|
| **1897** | NA | NA | 1.77E+07 | 6.30E+06 | NA | NA | NA | NA | NA | NA |
| **1898** | 0.00E+00 | 5.62E+06 | NA | NA | 1.23E+07 | 1.17E+07 | NA | 0.00E+00 | NA | 4.81E-01 |
| **1899** | NA | NA | 1.78E+07 | 4.94E+06 | 1.00E+06 | 1.51E+05 | NA | NA | NA | NA |
| **1900** | NA | NA | 2.37E+07 | 0.00E+00 | NA | NA | NA | NA | NA | NA |
| **1901** | NA | NA | 1.67E+07 | 6.48E+06 | NA | NA | NA | NA | NA | NA |
| **1902** | NA | NA | 1.36E+07 | 9.32E+06 | NA | NA | NA | NA | NA | NA |
| **1903** | 1.74E+07 | 0.00E+00 | 1.25E+07 | 8.04E+06 | 3.95E+05 | 1.53E+06 | 1.39E+00 | 4.40E+01 | 0.00E+00 | 0.00E+00 |
| **1904** | 1.26E+07 | 0.00E+00 | 1.86E+07 | 3.26E+06 | 0.00E+00 | 5.72E+05 | 6.79E-01 | Inf | 0.00E+00 | 0.00E+00 |
| **1905** | NA | NA | 1.28E+07 | 9.04E+06 | 4.59E+05 | 0.00E+00 | NA | NA | NA | NA |
| **1906** | NA | NA | 1.33E+07 | 8.86E+06 | NA | NA | NA | NA | NA | NA |
| **1907** | NA | NA | 1.25E+07 | 9.60E+06 | NA | NA | NA | NA | NA | NA |
| **1908** | 0.00E+00 | 2.20E+07 | NA | NA | NA | NA | NA | NA | NA | NA |
| **1909** | NA | NA | 2.07E+07 | 1.32E+06 | NA | NA | NA | NA | NA | NA |
| **1910** | NA | NA | 1.22E+07 | 9.82E+06 | NA | NA | NA | NA | NA | NA |
| **1911** | NA | NA | 1.36E+07 | 8.26E+06 | 0.00E+00 | 9.55E+04 | NA | NA | NA | NA |
| **1912** | NA | NA | 8.18E+06 | 1.34E+07 | 1.29E+05 | 1.53E+05 | NA | NA | NA | NA |
| **1913** | NA | NA | 7.38E+06 | 1.38E+07 | 4.73E+05 | 0.00E+00 | NA | NA | NA | NA |
| **1914** | 7.47E+05 | 3.37E+07 | 1.26E+07 | 8.34E+06 | 3.73E+05 | 1.90E+05 | 5.93E-02 | 2.00E+00 | 4.05E+00 | 1.77E+02 |
| **1915** | 2.29E+07 | 1.52E+08 | 2.05E+07 | 0.00E+00 | 0.00E+00 | 9.36E+05 | 1.12E+00 | Inf | Inf | 1.63E+02 |
| **1916** | 7.90E+05 | 0.00E+00 | 9.40E+06 | 1.05E+07 | 1.04E+06 | 3.53E+05 | 8.41E-02 | 7.60E-01 | 0.00E+00 | 0.00E+00 |
| **1917** | 0.00E+00 | 2.46E+06 | 1.05E+07 | 1.07E+07 | 0.00E+00 | 0.00E+00 | 0.00E+00 | NA | 2.29E-01 | Inf |
| **1918** | NA | NA | 2.10E+07 | 0.00E+00 | 0.00E+00 | 8.83E+04 | NA | NA | NA | NA |
| **1919** | NA | NA | 1.75E+07 | 3.10E+06 | NA | NA | NA | NA | NA | NA |
| **1920** | NA | NA | 1.89E+07 | 0.00E+00 | 6.44E+05 | 1.03E+06 | NA | NA | NA | NA |
| **1921** | 0.00E+00 | 1.12E+06 | 1.98E+07 | 0.00E+00 | 3.49E+05 | 2.06E+05 | 0.00E+00 | 0.00E+00 | Inf | 5.43E+00 |
| **1922** | 0.00E+00 | 5.17E+05 | 4.66E+06 | 1.56E+07 | 0.00E+00 | 0.00E+00 | 0.00E+00 | NA | 3.31E-02 | Inf |
| **1923** | NA | NA | 2.02E+07 | 0.00E+00 | 0.00E+00 | 0.00E+00 | NA | NA | NA | NA |
| **1924** | 3.12E+06 | 2.80E+06 | 5.64E+06 | 1.43E+07 | 1.68E+05 | 0.00E+00 | 5.53E-01 | 1.86E+01 | 1.96E-01 | Inf |
| **1925** | NA | NA | 1.07E+07 | 8.86E+06 | 0.00E+00 | 4.45E+05 | NA | NA | NA | NA |
| **1926** | 3.52E+06 | 0.00E+00 | 4.50E+06 | 1.20E+07 | 2.79E+06 | 5.96E+05 | 7.81E-01 | 1.26E+00 | 0.00E+00 | 0.00E+00 |
| **1927** | 0.00E+00 | 1.99E+07 | NA | NA | 0.00E+00 | 0.00E+00 | NA | NA | NA | Inf |
| **1928** | NA | NA | 1.49E+07 | 4.90E+06 | NA | NA | NA | NA | NA | NA |
| **1929** | NA | NA | 6.45E+06 | 1.05E+07 | 2.81E+06 | 0.00E+00 | NA | NA | NA | NA |
| **1930** | NA | NA | 1.69E+07 | 2.92E+06 | NA | NA | NA | NA | NA | NA |
| **1931** | NA | NA | 1.28E+07 | 6.80E+06 | 1.08E+05 | 0.00E+00 | NA | NA | NA | NA |
| **1932** | NA | NA | 0.00E+00 | 1.94E+07 | 1.35E+05 | 0.00E+00 | NA | NA | NA | NA |
| **1933** | 0.00E+00 | 1.94E+07 | NA | NA | 0.00E+00 | 0.00E+00 | NA | NA | NA | Inf |
| **1934** | NA | NA | 5.80E+06 | 1.28E+07 | 2.86E+05 | 3.96E+05 | NA | NA | NA | NA |

| | | | | | | | | | | |
|---|---|---|---|---|---|---|---|---|---|---|
| **1935** | NA | NA | 8.70E+06 | 1.02E+07 | 3.84E+05 | 0.00E+00 | NA | NA | NA | NA |
| **1936** | NA | NA | 1.03E+07 | 8.02E+06 | 8.30E+05 | 0.00E+00 | NA | NA | NA | NA |
| **1937** | 1.83E+07 | 2.93E+07 | 1.45E+07 | 1.90E+06 | 4.28E+04 | 2.54E+06 | 1.26E+00 | 4.28E+02 | 1.54E+01 | 1.15E+01 |
| **1938** | NA | NA | 9.02E+06 | 9.11E+06 | 1.79E+05 | 1.81E+05 | NA | NA | NA | NA |
| **1939** | NA | NA | 1.38E+07 | 4.72E+06 | NA | NA | NA | NA | NA | NA |
| **1940** | NA | NA | 1.18E+07 | 6.72E+06 | 0.00E+00 | 0.00E+00 | NA | NA | NA | NA |
| **1941** | NA | NA | 7.94E+06 | 1.04E+07 | 1.26E+05 | 0.00E+00 | NA | NA | NA | NA |
| **1942** | NA | NA | 4.80E+06 | 1.36E+07 | NA | NA | NA | NA | NA | NA |
| **1943** | NA | NA | 1.12E+07 | 7.08E+06 | NA | NA | NA | NA | NA | NA |
| **1944** | NA | NA | 1.83E+07 | 0.00E+00 | NA | NA | NA | NA | NA | NA |
| **1945** | NA | NA | 1.50E+07 | 3.14E+06 | 0.00E+00 | 0.00E+00 | NA | NA | NA | NA |
| **1946** | 0.00E+00 | 1.80E+07 | NA | NA | NA | NA | NA | NA | NA | NA |
| **1947** | NA | NA | 1.79E+07 | 0.00E+00 | NA | NA | NA | NA | NA | NA |
| **1948** | NA | NA | 1.04E+07 | 7.52E+06 | NA | NA | NA | NA | NA | NA |
| **1949** | NA | NA | 8.76E+06 | 8.94E+06 | 0.00E+00 | 1.82E+05 | NA | NA | NA | NA |
| **1950** | 0.00E+00 | 1.78E+07 | NA | NA | NA | NA | NA | NA | NA | NA |
| **1951** | NA | NA | 1.72E+07 | 0.00E+00 | 1.57E+05 | 2.75E+05 | NA | NA | NA | NA |
| **1952** | NA | NA | 1.73E+07 | 0.00E+00 | NA | NA | NA | NA | NA | NA |
| **1953** | NA | NA | 0.00E+00 | 1.72E+07 | NA | NA | NA | NA | NA | NA |
| **1954** | NA | NA | 6.64E+06 | 1.02E+07 | 3.71E+04 | 2.45E+05 | NA | NA | NA | NA |
| **1955** | NA | NA | 1.85E+06 | 1.50E+07 | 0.00E+00 | 8.30E+04 | NA | NA | NA | NA |
| **1956** | NA | NA | NA | NA | 0.00E+00 | 1.69E+07 | NA | NA | NA | NA |
| **1957** | 2.79E+05 | 0.00E+00 | 0.00E+00 | 1.58E+07 | 0.00E+00 | 8.71E+05 | Inf | Inf | 0.00E+00 | 0.00E+00 |
| **1958** | NA | NA | 1.66E+07 | 0.00E+00 | 0.00E+00 | 0.00E+00 | NA | NA | NA | NA |
| **1959** | 1.33E+07 | 4.28E+05 | 1.23E+07 | 3.54E+06 | 0.00E+00 | 5.36E+05 | 1.08E+00 | Inf | 1.21E-01 | 8.00E-01 |
| **1960** | NA | NA | 1.60E+07 | 0.00E+00 | 0.00E+00 | 4.79E+04 | NA | NA | NA | NA |
| **1961** | NA | NA | 1.59E+07 | 0.00E+00 | NA | NA | NA | NA | NA | NA |
| **1962** | NA | NA | 1.32E+07 | 2.22E+06 | NA | NA | NA | NA | NA | NA |
| **1963** | NA | NA | 8.30E+06 | 6.72E+06 | NA | NA | NA | NA | NA | NA |
| **1964** | NA | NA | 8.58E+06 | 6.32E+06 | NA | NA | NA | NA | NA | NA |
| **1965** | NA | NA | 7.96E+06 | 6.92E+06 | NA | NA | NA | NA | NA | NA |
| **1966** | NA | NA | 1.24E+07 | 2.36E+06 | 0.00E+00 | 0.00E+00 | NA | NA | NA | NA |
| **1967** | 7.49E+05 | 4.27E+06 | 5.28E+06 | 8.46E+06 | 1.47E+05 | 6.04E+05 | 1.42E-01 | 5.10E+00 | 5.05E-01 | 7.07E+00 |
| **1968** | NA | NA | 9.33E+06 | 5.14E+06 | NA | NA | NA | NA | NA | NA |
| **1969** | NA | NA | 1.19E+07 | 2.58E+06 | NA | NA | NA | NA | NA | NA |
| **1970** | 6.14E+06 | 0.00E+00 | 4.80E+06 | 8.30E+06 | 1.44E+05 | 1.04E+06 | 1.28E+00 | 4.25E+01 | 0.00E+00 | 0.00E+00 |
| **1971** | 6.60E+05 | 0.00E+00 | 0.00E+00 | 1.41E+07 | 0.00E+00 | 1.00E+05 | Inf | Inf | 0.00E+00 | 0.00E+00 |
| **1972** | 0.00E+00 | 1.42E+07 | NA | NA | 0.00E+00 | 0.00E+00 | NA | NA | NA | Inf |

| | | | | | | | | | | |
|---|---|---|---|---|---|---|---|---|---|---|
| **1973** | 1.84E+07 | 2.51E+05 | 9.80E+06 | 2.52E+06 | 3.35E+05 | 1.22E+06 | 1.88E+00 | 5.49E+01 | 9.95E-02 | 2.06E-01 |
| **1974** | NA | NA | NA | NA | 0.00E+00 | 1.36E+07 | NA | NA | NA | NA |
| **1975** | 1.03E+07 | 0.00E+00 | NA | NA | 5.48E+05 | 1.30E+07 | NA | 1.88E+01 | NA | 0.00E+00 |
| **1976** | NA | NA | 1.00E+07 | 3.44E+06 | NA | NA | NA | NA | NA | NA |
| **1977** | NA | NA | NA | NA | 6.97E+06 | 6.19E+06 | NA | NA | NA | NA |
| **1978** | 0.00E+00 | 1.28E+07 | NA | NA | 0.00E+00 | 0.00E+00 | NA | NA | NA | Inf |
| **1979** | NA | NA | 0.00E+00 | 1.26E+07 | 1.53E+05 | 0.00E+00 | NA | NA | NA | NA |
| **1980** | NA | NA | 4.78E+06 | 7.38E+06 | 4.73E+05 | 0.00E+00 | NA | NA | NA | NA |
| **1981** | NA | NA | 8.96E+06 | 3.26E+06 | 0.00E+00 | 3.63E+05 | NA | NA | NA | NA |
| **1982** | 0.00E+00 | 1.25E+07 | NA | NA | 0.00E+00 | 0.00E+00 | NA | NA | NA | Inf |
| **1983** | NA | NA | 9.91E+05 | 1.10E+07 | NA | NA | NA | NA | NA | NA |
| **1984** | 0.00E+00 | 1.20E+07 | NA | NA | 0.00E+00 | 0.00E+00 | NA | NA | NA | Inf |
| **1985** | NA | NA | 2.82E+06 | 8.84E+06 | NA | NA | NA | NA | NA | NA |
| **1986** | 8.45E+06 | 0.00E+00 | 5.80E+06 | 4.62E+06 | 6.62E+05 | 3.99E+05 | 1.46E+00 | 1.28E+01 | 0.00E+00 | 0.00E+00 |
| **1987** | 0.00E+00 | 2.38E+06 | 0.00E+00 | 1.03E+07 | 8.57E+05 | 2.97E+05 | NA | 0.00E+00 | 2.31E-01 | 8.04E+00 |
| **1988** | NA | NA | 3.14E+06 | 8.28E+06 | NA | NA | NA | NA | NA | NA |
| **1989** | NA | NA | 1.14E+07 | 0.00E+00 | NA | NA | NA | NA | NA | NA |
| **1990** | 0.00E+00 | 4.01E+06 | 4.24E+06 | 6.94E+06 | NA | NA | 0.00E+00 | NA | 5.78E-01 | NA |
| **1991** | NA | NA | NA | NA | 7.97E+06 | 3.18E+06 | NA | NA | NA | NA |
| **1992** | NA | NA | 1.10E+07 | 0.00E+00 | NA | NA | NA | NA | NA | NA |
| **1993** | 0.00E+00 | 3.00E+06 | 3.42E+06 | 6.08E+06 | 6.80E+05 | 7.66E+05 | 0.00E+00 | 0.00E+00 | 4.94E-01 | 3.92E+00 |
| **1994** | 4.10E+07 | 3.17E+07 | NA | NA | 3.16E+05 | 1.06E+07 | NA | 1.30E+02 | NA | 3.00E+00 |
| **1995** | 0.00E+00 | 1.08E+07 | NA | NA | 0.00E+00 | 0.00E+00 | NA | NA | NA | Inf |
| **1996** | 0.00E+00 | 1.06E+07 | NA | NA | 0.00E+00 | 0.00E+00 | NA | NA | NA | Inf |
| **1997** | NA | NA | 1.05E+07 | 0.00E+00 | NA | NA | NA | NA | NA | NA |
| **1998** | 1.04E+07 | 0.00E+00 | NA | NA | 0.00E+00 | 0.00E+00 | NA | Inf | NA | NA |
| **1999** | NA | NA | 1.00E+07 | 0.00E+00 | 3.56E+05 | 0.00E+00 | NA | NA | NA | NA |
| **2000** | NA | NA | 7.12E+06 | 3.20E+06 | NA | NA | NA | NA | NA | NA |
| **2001** | NA | NA | 0.00E+00 | 1.03E+07 | NA | NA | NA | NA | NA | NA |
| **2002** | 1.02E+07 | 0.00E+00 | NA | NA | NA | NA | NA | NA | NA | NA |
| **2003** | 0.00E+00 | 1.02E+07 | NA | NA | 0.00E+00 | 0.00E+00 | NA | NA | NA | Inf |
| **2004** | NA | NA | 7.04E+06 | 2.76E+06 | 9.49E+04 | 6.42E+04 | NA | NA | NA | NA |
| **2005** | 0.00E+00 | 9.78E+06 | NA | NA | NA | NA | NA | NA | NA | NA |
| **2006** | NA | NA | 9.60E+06 | 0.00E+00 | NA | NA | NA | NA | NA | NA |
| **2007** | NA | NA | 9.16E+06 | 0.00E+00 | 2.75E+05 | 0.00E+00 | NA | NA | NA | NA |
| **2008** | NA | NA | 9.34E+06 | 0.00E+00 | 0.00E+00 | 0.00E+00 | NA | NA | NA | NA |
| **2009** | 0.00E+00 | 4.25E+06 | 4.48E+06 | 4.42E+06 | 3.76E+05 | 0.00E+00 | 0.00E+00 | 0.00E+00 | 9.60E-01 | Inf |
| **2010** | NA | NA | 6.36E+06 | 2.70E+06 | NA | NA | NA | NA | NA | NA |

| | | | | | | | | | | |
|---|---|---|---|---|---|---|---|---|---|---|
| **2011** | NA | NA | 9.02E+06 | 0.00E+00 | NA | NA | NA | NA | NA | NA |
| **2012** | NA | NA | 0.00E+00 | 8.86E+06 | 0.00E+00 | 1.18E+05 | NA | NA | NA | NA |
| **2013** | 0.00E+00 | 8.91E+06 | NA | NA | 0.00E+00 | 0.00E+00 | NA | NA | NA | Inf |
| **2014** | NA | NA | NA | NA | 6.56E+06 | 2.29E+06 | NA | NA | NA | NA |
| **2015** | NA | NA | NA | NA | 3.17E+06 | 5.60E+06 | NA | NA | NA | NA |
| **2016** | 0.00E+00 | 8.68E+06 | NA | NA | 0.00E+00 | 0.00E+00 | NA | NA | NA | Inf |
| **2017** | 0.00E+00 | 1.53E+06 | 0.00E+00 | 7.82E+06 | 4.31E+05 | 4.13E+05 | NA | 0.00E+00 | 1.96E-01 | 3.71E+00 |
| **2018** | 0.00E+00 | 3.98E+07 | 3.90E+06 | 4.62E+06 | 0.00E+00 | 0.00E+00 | 0.00E+00 | NA | 8.63E+00 | Inf |
| **2019** | 0.00E+00 | 8.33E+06 | NA | NA | 0.00E+00 | 0.00E+00 | NA | NA | NA | Inf |
| **2020** | 0.00E+00 | 8.29E+06 | NA | NA | 0.00E+00 | 0.00E+00 | NA | NA | NA | Inf |
| **2021** | 0.00E+00 | 8.29E+06 | NA | NA | 0.00E+00 | 0.00E+00 | NA | NA | NA | Inf |
| **2022** | 8.28E+06 | 0.00E+00 | NA | NA | NA | NA | NA | NA | NA | NA |
| **2023** | 0.00E+00 | 4.43E+05 | 5.80E+06 | 2.26E+06 | NA | NA | 0.00E+00 | NA | 1.96E-01 | NA |
| **2024** | NA | NA | NA | NA | 4.39E+06 | 3.62E+06 | NA | NA | NA | NA |
| **2025** | 0.00E+00 | 7.99E+06 | NA | NA | NA | NA | NA | NA | NA | NA |
| **2026** | NA | NA | 7.92E+06 | 0.00E+00 | NA | NA | NA | NA | NA | NA |
| **2027** | NA | NA | 0.00E+00 | 7.10E+06 | 1.62E+05 | 1.45E+05 | NA | NA | NA | NA |
| **2028** | 2.05E+06 | 2.16E+06 | NA | NA | 3.82E+06 | 3.47E+06 | NA | 5.38E-01 | NA | 6.22E-01 |
| **2029** | NA | NA | 7.16E+06 | 0.00E+00 | NA | NA | NA | NA | NA | NA |
| **2030** | 0.00E+00 | 6.83E+06 | NA | NA | 0.00E+00 | 0.00E+00 | NA | NA | NA | Inf |
| **2031** | 2.62E+05 | 0.00E+00 | NA | NA | 4.32E+06 | 2.49E+06 | NA | 6.06E-02 | NA | 0.00E+00 |
| **2032** | NA | NA | 6.80E+06 | 0.00E+00 | 0.00E+00 | 0.00E+00 | NA | NA | NA | NA |
| **2033** | NA | NA | NA | NA | 9.80E+05 | 5.27E+06 | NA | NA | NA | NA |
| **2034** | NA | NA | NA | NA | 4.27E+06 | 1.85E+06 | NA | NA | NA | NA |
| **2035** | 0.00E+00 | 6.11E+06 | NA | NA | NA | NA | NA | NA | NA | NA |
| **2036** | 0.00E+00 | 6.08E+06 | NA | NA | NA | NA | NA | NA | NA | NA |
| **2037** | 0.00E+00 | 5.98E+06 | NA | NA | NA | NA | NA | NA | NA | NA |
| **2038** | NA | NA | NA | NA | 5.52E+06 | 4.55E+05 | NA | NA | NA | NA |
| **2039** | 7.82E+06 | 0.00E+00 | NA | NA | 4.98E+06 | 8.63E+05 | NA | 1.57E+00 | NA | 0.00E+00 |
| **2040** | 8.26E+05 | 0.00E+00 | NA | NA | 4.33E+06 | 1.50E+06 | NA | 1.91E-01 | NA | 0.00E+00 |
| **2041** | NA | NA | 0.00E+00 | 5.82E+06 | NA | NA | NA | NA | NA | NA |
| **2042** | NA | NA | NA | NA | 3.38E+06 | 2.21E+06 | NA | NA | NA | NA |
| **2043** | 0.00E+00 | 5.53E+06 | NA | NA | 0.00E+00 | 0.00E+00 | NA | NA | NA | Inf |
| **2044** | NA | NA | 0.00E+00 | 5.30E+06 | 0.00E+00 | 0.00E+00 | NA | NA | NA | NA |
| **2045** | 3.21E+05 | 0.00E+00 | NA | NA | 3.37E+05 | 4.96E+06 | NA | 9.55E-01 | NA | 0.00E+00 |
| **2046** | NA | NA | NA | NA | 0.00E+00 | 5.20E+06 | NA | NA | NA | NA |
| **2047** | 0.00E+00 | 5.18E+06 | NA | NA | 0.00E+00 | 0.00E+00 | NA | NA | NA | Inf |
| **2048** | 0.00E+00 | 5.18E+06 | NA | NA | 0.00E+00 | 0.00E+00 | NA | NA | NA | Inf |

| | | | | | | | | | | |
|---|---|---|---|---|---|---|---|---|---|---|
| **2049** | 0.00E+00 | 5.17E+06 | NA | NA | 0.00E+00 | 0.00E+00 | NA | NA | NA | Inf |
| **2050** | 0.00E+00 | 5.10E+06 | NA | NA | 0.00E+00 | 0.00E+00 | NA | NA | NA | Inf |
| **2051** | 5.09E+06 | 0.00E+00 | NA | NA | NA | NA | NA | NA | NA | NA |
| **2052** | 1.33E+06 | 0.00E+00 | 0.00E+00 | 3.68E+06 | 1.01E+06 | 3.28E+05 | Inf | 1.32E+00 | 0.00E+00 | 0.00E+00 |
| **2053** | 0.00E+00 | 4.96E+06 | NA | NA | NA | NA | NA | NA | NA | NA |
| **2054** | NA | NA | 0.00E+00 | 4.92E+06 | NA | NA | NA | NA | NA | NA |
| **2055** | 0.00E+00 | 4.86E+06 | NA | NA | 0.00E+00 | 0.00E+00 | NA | NA | NA | Inf |
| **2056** | 0.00E+00 | 4.81E+06 | NA | NA | 0.00E+00 | 0.00E+00 | NA | NA | NA | Inf |
| **2057** | NA | NA | NA | NA | 1.22E+06 | 3.58E+06 | NA | NA | NA | NA |
| **2058** | 0.00E+00 | 4.77E+06 | NA | NA | 0.00E+00 | 0.00E+00 | NA | NA | NA | Inf |
| **2059** | 0.00E+00 | 4.77E+06 | NA | NA | 0.00E+00 | 0.00E+00 | NA | NA | NA | Inf |
| **2060** | 0.00E+00 | 4.77E+06 | NA | NA | 0.00E+00 | 0.00E+00 | NA | NA | NA | Inf |
| **2061** | 0.00E+00 | 4.73E+06 | NA | NA | NA | NA | NA | NA | NA | NA |
| **2062** | 0.00E+00 | 4.55E+06 | NA | NA | 0.00E+00 | 0.00E+00 | NA | NA | NA | Inf |
| **2063** | 4.52E+06 | 0.00E+00 | NA | NA | 0.00E+00 | 0.00E+00 | NA | Inf | NA | NA |
| **2064** | NA | NA | 4.46E+06 | 0.00E+00 | NA | NA | NA | NA | NA | NA |
| **2065** | NA | NA | NA | NA | 3.34E+06 | 1.10E+06 | NA | NA | NA | NA |
| **2066** | 4.34E+06 | 0.00E+00 | NA | NA | NA | NA | NA | NA | NA | NA |
| **2067** | 0.00E+00 | 4.21E+06 | NA | NA | 0.00E+00 | 0.00E+00 | NA | NA | NA | Inf |
| **2068** | 0.00E+00 | 6.14E+06 | NA | NA | 2.51E+06 | 1.52E+06 | NA | 0.00E+00 | NA | 4.04E+00 |
| **2069** | NA | NA | NA | NA | 3.90E+06 | 9.84E+04 | NA | NA | NA | NA |
| **2070** | 3.95E+06 | 0.00E+00 | NA | NA | NA | NA | NA | NA | NA | NA |
| **2071** | 3.87E+06 | 0.00E+00 | NA | NA | NA | NA | NA | NA | NA | NA |
| **2072** | NA | NA | NA | NA | 2.12E+06 | 1.70E+06 | NA | NA | NA | NA |
| **2073** | NA | NA | 0.00E+00 | 3.82E+06 | NA | NA | NA | NA | NA | NA |
| **2074** | 0.00E+00 | 3.82E+06 | NA | NA | NA | NA | NA | NA | NA | NA |
| **2075** | 0.00E+00 | 3.82E+06 | NA | NA | NA | NA | NA | NA | NA | NA |
| **2076** | 6.25E+07 | 0.00E+00 | NA | NA | 4.06E+05 | 3.40E+06 | NA | 1.54E+02 | NA | 0.00E+00 |
| **2077** | 0.00E+00 | 3.79E+06 | NA | NA | NA | NA | NA | NA | NA | NA |
| **2078** | 0.00E+00 | 3.76E+06 | NA | NA | NA | NA | NA | NA | NA | NA |
| **2079** | 0.00E+00 | 3.74E+06 | NA | NA | NA | NA | NA | NA | NA | NA |
| **2080** | 3.66E+06 | 0.00E+00 | NA | NA | NA | NA | NA | NA | NA | NA |
| **2081** | NA | NA | NA | NA | 1.51E+06 | 2.15E+06 | NA | NA | NA | NA |
| **2082** | NA | NA | NA | NA | 1.55E+06 | 2.02E+06 | NA | NA | NA | NA |
| **2083** | NA | NA | NA | NA | 2.73E+06 | 8.47E+05 | NA | NA | NA | NA |
| **2084** | 0.00E+00 | 3.43E+06 | NA | NA | 0.00E+00 | 0.00E+00 | NA | NA | NA | Inf |
| **2085** | 2.65E+08 | 7.35E+07 | NA | NA | 1.34E+06 | 2.06E+06 | NA | 1.97E+02 | NA | 3.56E+01 |
| **2086** | NA | NA | NA | NA | 1.38E+06 | 1.98E+06 | NA | NA | NA | NA |

| | | | | | | | | | | |
|---|---|---|---|---|---|---|---|---|---|---|
| **2087** | 3.30E+06 | 0.00E+00 | NA | NA | NA | NA | NA | NA | NA | NA |
| **2088** | 0.00E+00 | 0.00E+00 | NA | NA | 1.71E+06 | 1.58E+06 | NA | 0.00E+00 | NA | 0.00E+00 |
| **2089** | 3.28E+06 | 0.00E+00 | NA | NA | NA | NA | NA | NA | NA | NA |
| **2090** | NA | NA | NA | NA | 1.63E+06 | 1.61E+06 | NA | NA | NA | NA |
| **2091** | NA | NA | NA | NA | 8.65E+05 | 2.21E+06 | NA | NA | NA | NA |
| **2092** | 2.82E+07 | 1.50E+06 | NA | NA | 5.25E+05 | 2.50E+06 | NA | 5.37E+01 | NA | 6.01E-01 |
| **2093** | NA | NA | NA | NA | 2.50E+06 | 4.89E+05 | NA | NA | NA | NA |
| **2094** | 1.15E+07 | 0.00E+00 | NA | NA | 1.44E+06 | 1.48E+06 | NA | 7.95E+00 | NA | 0.00E+00 |
| **2095** | 3.90E+07 | 0.00E+00 | NA | NA | 5.43E+05 | 2.37E+06 | NA | 7.19E+01 | NA | 0.00E+00 |
| **2096** | 0.00E+00 | 2.80E+06 | NA | NA | NA | NA | NA | NA | NA | NA |
| **2097** | NA | NA | NA | NA | 2.11E+06 | 6.51E+05 | NA | NA | NA | NA |
| **2098** | 0.00E+00 | 2.76E+06 | NA | NA | NA | NA | NA | NA | NA | NA |
| **2099** | NA | NA | 2.74E+06 | 0.00E+00 | NA | NA | NA | NA | NA | NA |
| **2100** | 3.30E+06 | 3.32E+06 | NA | NA | 1.96E+06 | 7.73E+05 | NA | 1.69E+00 | NA | 4.30E+00 |
| **2101** | 3.30E+06 | 3.32E+06 | NA | NA | 1.96E+06 | 7.73E+05 | NA | 1.69E+00 | NA | 4.30E+00 |
| **2102** | 6.16E+06 | 0.00E+00 | NA | NA | 1.88E+05 | 2.54E+06 | NA | 3.28E+01 | NA | 0.00E+00 |
| **2103** | 0.00E+00 | 2.66E+06 | NA | NA | 0.00E+00 | 0.00E+00 | NA | NA | NA | Inf |
| **2104** | 2.66E+06 | 0.00E+00 | NA | NA | NA | NA | NA | NA | NA | NA |
| **2105** | 2.63E+06 | 0.00E+00 | NA | NA | NA | NA | NA | NA | NA | NA |
| **2106** | 0.00E+00 | 2.60E+06 | NA | NA | NA | NA | NA | NA | NA | NA |
| **2107** | NA | NA | NA | NA | 2.21E+06 | 3.73E+05 | NA | NA | NA | NA |
| **2108** | 2.52E+06 | 0.00E+00 | NA | NA | NA | NA | NA | NA | NA | NA |
| **2109** | 0.00E+00 | 2.49E+06 | NA | NA | NA | NA | NA | NA | NA | NA |
| **2110** | 0.00E+00 | 2.44E+06 | NA | NA | NA | NA | NA | NA | NA | NA |
| **2111** | 0.00E+00 | 2.44E+06 | NA | NA | NA | NA | NA | NA | NA | NA |
| **2112** | 0.00E+00 | 2.42E+06 | NA | NA | 0.00E+00 | 0.00E+00 | NA | NA | NA | Inf |
| **2113** | 2.41E+06 | 0.00E+00 | NA | NA | NA | NA | NA | NA | NA | NA |
| **2114** | 5.63E+06 | 7.25E+07 | NA | NA | 0.00E+00 | 2.39E+06 | NA | Inf | NA | 3.03E+01 |
| **2115** | 1.39E+07 | 0.00E+00 | NA | NA | 4.78E+05 | 1.88E+06 | NA | 2.90E+01 | NA | 0.00E+00 |
| **2116** | NA | NA | 0.00E+00 | 2.34E+06 | NA | NA | NA | NA | NA | NA |
| **2117** | NA | NA | NA | NA | 1.47E+06 | 8.25E+05 | NA | NA | NA | NA |
| **2118** | 0.00E+00 | 2.27E+06 | NA | NA | 0.00E+00 | 0.00E+00 | NA | NA | NA | Inf |
| **2119** | NA | NA | NA | NA | 2.12E+06 | 1.39E+05 | NA | NA | NA | NA |
| **2120** | 0.00E+00 | 2.24E+06 | NA | NA | 0.00E+00 | 0.00E+00 | NA | NA | NA | Inf |
| **2121** | 2.87E+07 | 2.98E+07 | NA | NA | 1.53E+05 | 2.02E+06 | NA | 1.88E+02 | NA | 1.47E+01 |
| **2122** | NA | NA | NA | NA | 1.02E+06 | 1.13E+06 | NA | NA | NA | NA |
| **2123** | 0.00E+00 | 2.11E+06 | NA | NA | NA | NA | NA | NA | NA | NA |
| **2124** | 0.00E+00 | 2.11E+06 | NA | NA | NA | NA | NA | NA | NA | NA |

| | | | | | | | | | | |
|---|---|---|---|---|---|---|---|---|---|---|
| **2125** | 0.00E+00 | 2.10E+06 | NA | NA | 0.00E+00 | 0.00E+00 | NA | NA | NA | Inf |
| **2126** | NA | NA | NA | NA | 2.10E+06 | 0.00E+00 | NA | NA | NA | NA |
| **2127** | 0.00E+00 | 2.07E+06 | NA | NA | NA | NA | NA | NA | NA | NA |
| **2128** | 2.06E+06 | 0.00E+00 | NA | NA | NA | NA | NA | NA | NA | NA |
| **2129** | NA | NA | NA | NA | 1.60E+06 | 4.17E+05 | NA | NA | NA | NA |
| **2130** | NA | NA | NA | NA | 1.78E+06 | 2.32E+05 | NA | NA | NA | NA |
| **2131** | 2.00E+06 | 0.00E+00 | NA | NA | NA | NA | NA | NA | NA | NA |
| **2132** | 0.00E+00 | 1.96E+06 | NA | NA | 0.00E+00 | 0.00E+00 | NA | NA | NA | Inf |
| **2133** | 1.96E+06 | 0.00E+00 | NA | NA | NA | NA | NA | NA | NA | NA |
| **2134** | 1.96E+06 | 0.00E+00 | NA | NA | NA | NA | NA | NA | NA | NA |
| **2135** | 0.00E+00 | 1.95E+06 | NA | NA | NA | NA | NA | NA | NA | NA |
| **2136** | 1.95E+06 | 0.00E+00 | NA | NA | NA | NA | NA | NA | NA | NA |
| **2137** | 0.00E+00 | 1.95E+06 | NA | NA | NA | NA | NA | NA | NA | NA |
| **2138** | NA | NA | NA | NA | 2.89E+05 | 1.63E+06 | NA | NA | NA | NA |
| **2139** | NA | NA | NA | NA | 1.40E+06 | 4.99E+05 | NA | NA | NA | NA |
| **2140** | 1.86E+06 | 0.00E+00 | NA | NA | NA | NA | NA | NA | NA | NA |
| **2141** | NA | NA | NA | NA | 1.71E+06 | 1.36E+05 | NA | NA | NA | NA |
| **2142** | 0.00E+00 | 1.85E+06 | NA | NA | NA | NA | NA | NA | NA | NA |
| **2143** | 0.00E+00 | 1.82E+06 | NA | NA | NA | NA | NA | NA | NA | NA |
| **2144** | 1.81E+06 | 0.00E+00 | NA | NA | NA | NA | NA | NA | NA | NA |
| **2145** | 3.31E+06 | 0.00E+00 | NA | NA | 8.92E+05 | 9.16E+05 | NA | 3.71E+00 | NA | 0.00E+00 |
| **2146** | 0.00E+00 | 1.81E+06 | NA | NA | 0.00E+00 | 0.00E+00 | NA | NA | NA | Inf |
| **2147** | 1.69E+07 | 0.00E+00 | NA | NA | 0.00E+00 | 1.80E+06 | NA | Inf | NA | 0.00E+00 |
| **2148** | NA | NA | NA | NA | 1.61E+06 | 1.92E+05 | NA | NA | NA | NA |
| **2149** | NA | NA | NA | NA | 1.80E+06 | 0.00E+00 | NA | NA | NA | NA |
| **2150** | 0.00E+00 | 1.79E+06 | NA | NA | 0.00E+00 | 0.00E+00 | NA | NA | NA | Inf |
| **2151** | 1.79E+06 | 0.00E+00 | NA | NA | NA | NA | NA | NA | NA | NA |
| **2152** | NA | NA | NA | NA | 6.23E+05 | 1.17E+06 | NA | NA | NA | NA |
| **2153** | NA | NA | NA | NA | 1.78E+06 | 0.00E+00 | NA | NA | NA | NA |
| **2154** | 1.75E+06 | 0.00E+00 | NA | NA | NA | NA | NA | NA | NA | NA |
| **2155** | 1.73E+06 | 0.00E+00 | NA | NA | NA | NA | NA | NA | NA | NA |
| **2156** | NA | NA | NA | NA | 8.61E+05 | 8.24E+05 | NA | NA | NA | NA |
| **2157** | 0.00E+00 | 1.65E+06 | NA | NA | NA | NA | NA | NA | NA | NA |
| **2158** | NA | NA | NA | NA | 1.25E+06 | 3.87E+05 | NA | NA | NA | NA |
| **2159** | 0.00E+00 | 1.64E+06 | NA | NA | NA | NA | NA | NA | NA | NA |
| **2160** | NA | NA | NA | NA | 6.26E+05 | 9.96E+05 | NA | NA | NA | NA |
| **2161** | 0.00E+00 | 1.62E+06 | NA | NA | NA | NA | NA | NA | NA | NA |
| **2162** | NA | NA | NA | NA | 6.05E+05 | 9.84E+05 | NA | NA | NA | NA |

| | | | | | | | | | | |
|---|---|---|---|---|---|---|---|---|---|---|
| **2163** | 1.61E+07 | 5.31E+06 | NA | NA | 2.08E+05 | 1.38E+06 | NA | 7.73E+01 | NA | 3.85E+00 |
| **2164** | NA | NA | NA | NA | 1.58E+06 | 0.00E+00 | NA | NA | NA | NA |
| **2165** | 1.58E+06 | 0.00E+00 | NA | NA | 0.00E+00 | 0.00E+00 | NA | Inf | NA | NA |
| **2166** | NA | NA | NA | NA | 1.44E+06 | 1.12E+05 | NA | NA | NA | NA |
| **2167** | NA | NA | NA | NA | 1.22E+06 | 3.09E+05 | NA | NA | NA | NA |
| **2168** | NA | NA | NA | NA | 1.02E+06 | 4.86E+05 | NA | NA | NA | NA |
| **2169** | 1.06E+07 | 0.00E+00 | NA | NA | 0.00E+00 | 1.50E+06 | NA | Inf | NA | 0.00E+00 |
| **2170** | NA | NA | NA | NA | 1.01E+06 | 4.87E+05 | NA | NA | NA | NA |
| **2171** | 1.49E+06 | 0.00E+00 | NA | NA | NA | NA | NA | NA | NA | NA |
| **2172** | 0.00E+00 | 1.47E+06 | NA | NA | NA | NA | NA | NA | NA | NA |
| **2173** | 0.00E+00 | 1.47E+06 | NA | NA | 0.00E+00 | 0.00E+00 | NA | NA | NA | Inf |
| **2174** | NA | NA | NA | NA | 1.37E+06 | 8.67E+04 | NA | NA | NA | NA |
| **2175** | 1.82E+07 | 0.00E+00 | NA | NA | 7.74E+05 | 6.81E+05 | NA | 2.36E+01 | NA | 0.00E+00 |
| **2176** | NA | NA | NA | NA | 1.45E+06 | 0.00E+00 | NA | NA | NA | NA |
| **2177** | 0.00E+00 | 1.44E+06 | NA | NA | 0.00E+00 | 0.00E+00 | NA | NA | NA | Inf |
| **2178** | NA | NA | NA | NA | 1.20E+06 | 2.32E+05 | NA | NA | NA | NA |
| **2179** | 2.08E+06 | 0.00E+00 | NA | NA | 2.32E+05 | 1.19E+06 | NA | 8.98E+00 | NA | 0.00E+00 |
| **2180** | 1.42E+06 | 0.00E+00 | NA | NA | NA | NA | NA | NA | NA | NA |
| **2181** | 0.00E+00 | 1.42E+06 | NA | NA | 0.00E+00 | 0.00E+00 | NA | NA | NA | Inf |
| **2182** | NA | NA | NA | NA | 1.25E+06 | 1.55E+05 | NA | NA | NA | NA |
| **2183** | 0.00E+00 | 1.40E+06 | NA | NA | NA | NA | NA | NA | NA | NA |
| **2184** | 0.00E+00 | 1.39E+06 | NA | NA | NA | NA | NA | NA | NA | NA |
| **2185** | 2.66E+06 | 0.00E+00 | NA | NA | 7.17E+05 | 6.35E+05 | NA | 3.72E+00 | NA | 0.00E+00 |
| **2186** | 9.81E+05 | 0.00E+00 | NA | NA | 1.33E+06 | 0.00E+00 | NA | 7.36E-01 | NA | NA |
| **2187** | 4.48E+07 | 0.00E+00 | NA | NA | 0.00E+00 | 1.33E+06 | NA | Inf | NA | 0.00E+00 |
| **2188** | NA | NA | NA | NA | 9.32E+05 | 3.99E+05 | NA | NA | NA | NA |
| **2189** | 1.33E+06 | 0.00E+00 | NA | NA | NA | NA | NA | NA | NA | NA |
| **2190** | 0.00E+00 | 1.32E+06 | NA | NA | NA | NA | NA | NA | NA | NA |
| **2191** | 1.80E+07 | 0.00E+00 | NA | NA | 0.00E+00 | 1.31E+06 | NA | Inf | NA | 0.00E+00 |
| **2192** | 7.08E+06 | 0.00E+00 | NA | NA | 0.00E+00 | 1.30E+06 | NA | Inf | NA | 0.00E+00 |
| **2193** | NA | NA | NA | NA | 8.69E+05 | 4.24E+05 | NA | NA | NA | NA |
| **2194** | NA | NA | NA | NA | 1.29E+06 | 0.00E+00 | NA | NA | NA | NA |
| **2195** | NA | NA | NA | NA | 9.41E+05 | 3.25E+05 | NA | NA | NA | NA |
| **2196** | NA | NA | NA | NA | 3.95E+05 | 8.69E+05 | NA | NA | NA | NA |
| **2197** | 1.26E+06 | 0.00E+00 | NA | NA | NA | NA | NA | NA | NA | NA |
| **2198** | 1.26E+06 | 0.00E+00 | NA | NA | NA | NA | NA | NA | NA | NA |
| **2199** | NA | NA | NA | NA | 4.86E+05 | 7.62E+05 | NA | NA | NA | NA |
| **2200** | NA | NA | NA | NA | 7.78E+05 | 4.64E+05 | NA | NA | NA | NA |

| | | | | | | | | | | |
|---|---|---|---|---|---|---|---|---|---|---|
| **2201** | 1.12E+06 | 0.00E+00 | NA | NA | 1.67E+05 | 1.07E+06 | NA | 6.69E+00 | NA | 0.00E+00 |
| **2202** | NA | NA | NA | NA | 1.22E+06 | 0.00E+00 | NA | NA | NA | NA |
| **2203** | 1.21E+06 | 0.00E+00 | NA | NA | NA | NA | NA | NA | NA | NA |
| **2204** | 0.00E+00 | 1.21E+06 | NA | NA | 0.00E+00 | 0.00E+00 | NA | NA | NA | Inf |
| **2205** | 1.94E+07 | 8.07E+05 | NA | NA | 4.64E+05 | 7.37E+05 | NA | 4.19E+01 | NA | 1.10E+00 |
| **2206** | 1.20E+06 | 0.00E+00 | NA | NA | NA | NA | NA | NA | NA | NA |
| **2207** | NA | NA | NA | NA | 8.28E+05 | 3.69E+05 | NA | NA | NA | NA |
| **2208** | 0.00E+00 | 1.18E+06 | NA | NA | NA | NA | NA | NA | NA | NA |
| **2209** | 0.00E+00 | 1.18E+06 | NA | NA | NA | NA | NA | NA | NA | NA |
| **2210** | NA | NA | NA | NA | 3.61E+05 | 8.15E+05 | NA | NA | NA | NA |
| **2211** | NA | NA | NA | NA | 5.89E+05 | 5.87E+05 | NA | NA | NA | NA |
| **2212** | 5.89E+06 | 0.00E+00 | NA | NA | 3.97E+05 | 7.74E+05 | NA | 1.48E+01 | NA | 0.00E+00 |
| **2213** | 1.17E+06 | 0.00E+00 | NA | NA | NA | NA | NA | NA | NA | NA |
| **2214** | 1.16E+06 | 0.00E+00 | NA | NA | NA | NA | NA | NA | NA | NA |
| **2215** | 0.00E+00 | 1.16E+06 | NA | NA | NA | NA | NA | NA | NA | NA |
| **2216** | 0.00E+00 | 1.16E+06 | NA | NA | NA | NA | NA | NA | NA | NA |
| **2217** | NA | NA | NA | NA | 5.05E+05 | 6.42E+05 | NA | NA | NA | NA |
| **2218** | 0.00E+00 | 2.09E+06 | NA | NA | 6.85E+05 | 4.60E+05 | NA | 0.00E+00 | NA | 4.53E+00 |
| **2219** | 0.00E+00 | 1.14E+06 | NA | NA | NA | NA | NA | NA | NA | NA |
| **2220** | 1.12E+06 | 0.00E+00 | NA | NA | 0.00E+00 | 0.00E+00 | NA | Inf | NA | NA |
| **2221** | NA | NA | NA | NA | 8.10E+05 | 3.06E+05 | NA | NA | NA | NA |
| **2222** | 0.00E+00 | 1.11E+06 | NA | NA | 0.00E+00 | 0.00E+00 | NA | NA | NA | Inf |
| **2223** | 1.10E+06 | 0.00E+00 | NA | NA | NA | NA | NA | NA | NA | NA |
| **2224** | 0.00E+00 | 1.10E+06 | NA | NA | NA | NA | NA | NA | NA | NA |
| **2225** | NA | NA | NA | NA | 1.08E+06 | 0.00E+00 | NA | NA | NA | NA |
| **2226** | 1.08E+06 | 0.00E+00 | NA | NA | NA | NA | NA | NA | NA | NA |
| **2227** | NA | NA | NA | NA | 4.77E+05 | 6.02E+05 | NA | NA | NA | NA |
| **2228** | NA | NA | NA | NA | 1.01E+06 | 6.96E+04 | NA | NA | NA | NA |
| **2229** | 5.61E+05 | 0.00E+00 | NA | NA | 1.01E+06 | 5.12E+04 | NA | 5.53E-01 | NA | 0.00E+00 |
| **2230** | 1.06E+06 | 0.00E+00 | NA | NA | NA | NA | NA | NA | NA | NA |
| **2231** | 0.00E+00 | 1.96E+06 | NA | NA | 9.46E+05 | 9.27E+04 | NA | 0.00E+00 | NA | 2.12E+01 |
| **2232** | 1.04E+06 | 0.00E+00 | NA | NA | NA | NA | NA | NA | NA | NA |
| **2233** | 7.97E+06 | 0.00E+00 | NA | NA | 6.11E+05 | 4.25E+05 | NA | 1.30E+01 | NA | 0.00E+00 |
| **2234** | NA | NA | NA | NA | 6.47E+05 | 3.85E+05 | NA | NA | NA | NA |
| **2235** | 1.03E+07 | 4.13E+06 | NA | NA | 0.00E+00 | 1.03E+06 | NA | Inf | NA | 4.03E+00 |
| **2236** | NA | NA | NA | NA | 3.88E+05 | 6.24E+05 | NA | NA | NA | NA |
| **2237** | 1.42E+07 | 0.00E+00 | NA | NA | 2.34E+05 | 7.75E+05 | NA | 6.08E+01 | NA | 0.00E+00 |
| **2238** | NA | NA | NA | NA | 6.32E+05 | 3.70E+05 | NA | NA | NA | NA |

| | | | | | | | | | | |
|---|---|---|---|---|---|---|---|---|---|---|
| **2239** | NA | NA | NA | NA | 9.94E+05 | 0.00E+00 | NA | NA | NA | NA |
| **2240** | 9.92E+05 | 0.00E+00 | NA | NA | NA | NA | NA | NA | NA | NA |
| **2241** | 9.10E+06 | 0.00E+00 | NA | NA | 9.60E+04 | 8.95E+05 | NA | 9.48E+01 | NA | 0.00E+00 |
| **2242** | 2.31E+06 | 0.00E+00 | NA | NA | 0.00E+00 | 9.88E+05 | NA | Inf | NA | 0.00E+00 |
| **2243** | 5.46E+06 | 0.00E+00 | NA | NA | 5.91E+04 | 9.26E+05 | NA | 9.23E+01 | NA | 0.00E+00 |
| **2244** | 5.91E+06 | 0.00E+00 | NA | NA | 0.00E+00 | 9.85E+05 | NA | Inf | NA | 0.00E+00 |
| **2245** | NA | NA | NA | NA | 8.99E+05 | 8.10E+04 | NA | NA | NA | NA |
| **2246** | 8.63E+06 | 0.00E+00 | NA | NA | 5.79E+05 | 3.99E+05 | NA | 1.49E+01 | NA | 0.00E+00 |
| **2247** | 0.00E+00 | 3.17E+07 | NA | NA | 7.45E+05 | 2.25E+05 | NA | 0.00E+00 | NA | 1.41E+02 |
| **2248** | NA | NA | NA | NA | 7.51E+05 | 2.06E+05 | NA | NA | NA | NA |
| **2249** | NA | NA | NA | NA | 9.50E+05 | 0.00E+00 | NA | NA | NA | NA |
| **2250** | NA | NA | NA | NA | 8.99E+05 | 4.34E+04 | NA | NA | NA | NA |
| **2251** | 9.32E+05 | 0.00E+00 | NA | NA | NA | NA | NA | NA | NA | NA |
| **2252** | NA | NA | NA | NA | 4.49E+05 | 4.81E+05 | NA | NA | NA | NA |
| **2253** | NA | NA | NA | NA | 0.00E+00 | 9.20E+05 | NA | NA | NA | NA |
| **2254** | 1.84E+07 | 0.00E+00 | NA | NA | 9.75E+04 | 8.13E+05 | NA | 1.88E+02 | NA | 0.00E+00 |
| **2255** | NA | NA | NA | NA | 9.10E+05 | 0.00E+00 | NA | NA | NA | NA |
| **2256** | 0.00E+00 | 4.05E+06 | NA | NA | 6.80E+05 | 2.20E+05 | NA | 0.00E+00 | NA | 1.84E+01 |
| **2257** | NA | NA | NA | NA | 8.34E+05 | 6.22E+04 | NA | NA | NA | NA |
| **2258** | NA | NA | NA | NA | 8.94E+05 | 0.00E+00 | NA | NA | NA | NA |
| **2259** | NA | NA | NA | NA | 8.92E+05 | 0.00E+00 | NA | NA | NA | NA |
| **2260** | NA | NA | NA | NA | 8.90E+05 | 0.00E+00 | NA | NA | NA | NA |
| **2261** | NA | NA | NA | NA | 0.00E+00 | 8.85E+05 | NA | NA | NA | NA |
| **2262** | 0.00E+00 | 8.80E+05 | NA | NA | NA | NA | NA | NA | NA | NA |
| **2263** | NA | NA | NA | NA | 7.20E+05 | 1.59E+05 | NA | NA | NA | NA |
| **2264** | NA | NA | NA | NA | 8.64E+05 | 0.00E+00 | NA | NA | NA | NA |
| **2265** | NA | NA | NA | NA | 6.56E+05 | 1.96E+05 | NA | NA | NA | NA |
| **2266** | 0.00E+00 | 8.50E+05 | NA | NA | 0.00E+00 | 0.00E+00 | NA | NA | NA | Inf |
| **2267** | 0.00E+00 | 8.38E+05 | NA | NA | 0.00E+00 | 0.00E+00 | NA | NA | NA | Inf |
| **2268** | NA | NA | NA | NA | 8.36E+05 | 0.00E+00 | NA | NA | NA | NA |
| **2269** | 3.62E+05 | 0.00E+00 | NA | NA | 8.32E+05 | 0.00E+00 | NA | 4.35E-01 | NA | NA |
| **2270** | 8.27E+05 | 0.00E+00 | NA | NA | NA | NA | NA | NA | NA | NA |
| **2271** | 0.00E+00 | 8.22E+05 | NA | NA | NA | NA | NA | NA | NA | NA |
| **2272** | 0.00E+00 | 8.19E+05 | NA | NA | 0.00E+00 | 0.00E+00 | NA | NA | NA | Inf |
| **2273** | 8.18E+05 | 0.00E+00 | NA | NA | NA | NA | NA | NA | NA | NA |
| **2274** | NA | NA | NA | NA | 2.23E+05 | 5.88E+05 | NA | NA | NA | NA |
| **2275** | NA | NA | NA | NA | 6.40E+05 | 1.66E+05 | NA | NA | NA | NA |
| **2276** | 0.00E+00 | 8.05E+05 | NA | NA | NA | NA | NA | NA | NA | NA |

| | | | | | | | | | | |
|---|---|---|---|---|---|---|---|---|---|---|
| **2277** | NA | NA | NA | NA | 7.20E+05 | 8.35E+04 | NA | NA | NA | NA |
| **2278** | NA | NA | NA | NA | 4.59E+05 | 3.41E+05 | NA | NA | NA | NA |
| **2279** | NA | NA | NA | NA | 7.92E+05 | 0.00E+00 | NA | NA | NA | NA |
| **2280** | 4.66E+06 | 1.06E+07 | NA | NA | 0.00E+00 | 7.89E+05 | NA | Inf | NA | 1.34E+01 |
| **2281** | NA | NA | NA | NA | 1.55E+05 | 6.27E+05 | NA | NA | NA | NA |
| **2282** | 7.80E+05 | 0.00E+00 | NA | NA | NA | NA | NA | NA | NA | NA |
| **2283** | 0.00E+00 | 7.78E+05 | NA | NA | 0.00E+00 | 0.00E+00 | NA | NA | NA | Inf |
| **2284** | NA | NA | NA | NA | 0.00E+00 | 7.77E+05 | NA | NA | NA | NA |
| **2285** | 2.36E+06 | 3.78E+06 | NA | NA | 7.74E+05 | 0.00E+00 | NA | 3.05E+00 | NA | Inf |
| **2286** | 9.47E+06 | 0.00E+00 | NA | NA | 5.65E+05 | 1.94E+05 | NA | 1.68E+01 | NA | 0.00E+00 |
| **2287** | NA | NA | NA | NA | 7.56E+05 | 0.00E+00 | NA | NA | NA | NA |
| **2288** | 0.00E+00 | 7.56E+05 | NA | NA | NA | NA | NA | NA | NA | NA |
| **2289** | NA | NA | NA | NA | 1.99E+05 | 5.51E+05 | NA | NA | NA | NA |
| **2290** | NA | NA | NA | NA | 0.00E+00 | 7.45E+05 | NA | NA | NA | NA |
| **2291** | NA | NA | NA | NA | 4.17E+05 | 3.24E+05 | NA | NA | NA | NA |
| **2292** | 0.00E+00 | 7.38E+05 | NA | NA | NA | NA | NA | NA | NA | NA |
| **2293** | NA | NA | NA | NA | 7.37E+05 | 0.00E+00 | NA | NA | NA | NA |
| **2294** | 7.36E+05 | 0.00E+00 | NA | NA | NA | NA | NA | NA | NA | NA |
| **2295** | 7.54E+06 | 0.00E+00 | NA | NA | 4.28E+05 | 3.07E+05 | NA | 1.76E+01 | NA | 0.00E+00 |
| **2296** | 0.00E+00 | 7.28E+05 | NA | NA | 0.00E+00 | 0.00E+00 | NA | NA | NA | Inf |
| **2297** | NA | NA | NA | NA | 0.00E+00 | 7.24E+05 | NA | NA | NA | NA |
| **2298** | NA | NA | NA | NA | 7.14E+05 | 0.00E+00 | NA | NA | NA | NA |
| **2299** | NA | NA | NA | NA | 0.00E+00 | 7.12E+05 | NA | NA | NA | NA |
| **2300** | 0.00E+00 | 7.08E+05 | NA | NA | 0.00E+00 | 0.00E+00 | NA | NA | NA | Inf |
| **2301** | 0.00E+00 | 1.14E+06 | NA | NA | 7.02E+05 | 0.00E+00 | NA | 0.00E+00 | NA | Inf |
| **2302** | 6.47E+04 | 0.00E+00 | NA | NA | 0.00E+00 | 6.98E+05 | NA | Inf | NA | 0.00E+00 |
| **2303** | 1.05E+07 | 2.92E+06 | NA | NA | 6.97E+05 | 0.00E+00 | NA | 1.51E+01 | NA | Inf |
| **2304** | 0.00E+00 | 6.95E+05 | NA | NA | NA | NA | NA | NA | NA | NA |
| **2305** | 4.67E+06 | 4.26E+06 | NA | NA | 2.59E+05 | 4.32E+05 | NA | 1.81E+01 | NA | 9.87E+00 |
| **2306** | 6.01E+06 | 0.00E+00 | NA | NA | 0.00E+00 | 6.89E+05 | NA | Inf | NA | 0.00E+00 |
| **2307** | 1.22E+07 | 0.00E+00 | NA | NA | 0.00E+00 | 6.84E+05 | NA | Inf | NA | 0.00E+00 |
| **2308** | NA | NA | NA | NA | 6.84E+05 | 0.00E+00 | NA | NA | NA | NA |
| **2309** | NA | NA | NA | NA | 6.76E+05 | 0.00E+00 | NA | NA | NA | NA |
| **2310** | NA | NA | NA | NA | 6.72E+05 | 0.00E+00 | NA | NA | NA | NA |
| **2311** | NA | NA | NA | NA | 4.06E+05 | 2.62E+05 | NA | NA | NA | NA |
| **2312** | 0.00E+00 | 6.60E+05 | NA | NA | NA | NA | NA | NA | NA | NA |
| **2313** | NA | NA | NA | NA | 6.55E+05 | 0.00E+00 | NA | NA | NA | NA |
| **2314** | NA | NA | NA | NA | 6.53E+05 | 0.00E+00 | NA | NA | NA | NA |

| | | | | | | | | | | |
|---|---|---|---|---|---|---|---|---|---|---|
| **2315** | NA | NA | NA | NA | 6.53E+05 | 0.00E+00 | NA | NA | NA | NA |
| **2316** | NA | NA | NA | NA | 5.59E+05 | 9.14E+04 | NA | NA | NA | NA |
| **2317** | 0.00E+00 | 6.47E+05 | NA | NA | NA | NA | NA | NA | NA | NA |
| **2318** | 1.04E+07 | 0.00E+00 | NA | NA | 3.21E+05 | 3.26E+05 | NA | 3.25E+01 | NA | 0.00E+00 |
| **2319** | 1.50E+07 | 0.00E+00 | NA | NA | 4.42E+05 | 2.02E+05 | NA | 3.41E+01 | NA | 0.00E+00 |
| **2320** | NA | NA | NA | NA | 5.31E+05 | 1.12E+05 | NA | NA | NA | NA |
| **2321** | NA | NA | NA | NA | 6.32E+05 | 0.00E+00 | NA | NA | NA | NA |
| **2322** | 0.00E+00 | 6.31E+05 | NA | NA | NA | NA | NA | NA | NA | NA |
| **2323** | NA | NA | NA | NA | 3.74E+05 | 2.53E+05 | NA | NA | NA | NA |
| **2324** | 0.00E+00 | 1.49E+06 | NA | NA | 6.26E+05 | 0.00E+00 | NA | 0.00E+00 | NA | Inf |
| **2325** | 1.87E+07 | 5.40E+05 | NA | NA | 6.26E+05 | 0.00E+00 | NA | 2.98E+01 | NA | Inf |
| **2326** | NA | NA | NA | NA | 3.32E+05 | 2.87E+05 | NA | NA | NA | NA |
| **2327** | NA | NA | NA | NA | 3.20E+05 | 2.97E+05 | NA | NA | NA | NA |
| **2328** | NA | NA | NA | NA | 3.22E+05 | 2.95E+05 | NA | NA | NA | NA |
| **2329** | 6.14E+05 | 0.00E+00 | NA | NA | NA | NA | NA | NA | NA | NA |
| **2330** | 7.56E+06 | 0.00E+00 | NA | NA | 0.00E+00 | 6.14E+05 | NA | Inf | NA | 0.00E+00 |
| **2331** | NA | NA | NA | NA | 5.90E+04 | 5.55E+05 | NA | NA | NA | NA |
| **2332** | 4.09E+06 | 0.00E+00 | NA | NA | 0.00E+00 | 5.97E+05 | NA | Inf | NA | 0.00E+00 |
| **2333** | NA | NA | NA | NA | 5.94E+05 | 0.00E+00 | NA | NA | NA | NA |
| **2334** | NA | NA | NA | NA | 1.72E+05 | 4.20E+05 | NA | NA | NA | NA |
| **2335** | 5.90E+05 | 0.00E+00 | NA | NA | NA | NA | NA | NA | NA | NA |
| **2336** | 1.31E+05 | 0.00E+00 | NA | NA | 0.00E+00 | 5.89E+05 | NA | Inf | NA | 0.00E+00 |
| **2337** | NA | NA | NA | NA | 5.81E+05 | 0.00E+00 | NA | NA | NA | NA |
| **2338** | NA | NA | NA | NA | 5.09E+04 | 5.26E+05 | NA | NA | NA | NA |
| **2339** | 0.00E+00 | 5.74E+05 | NA | NA | NA | NA | NA | NA | NA | NA |
| **2340** | 0.00E+00 | 3.23E+06 | NA | NA | 4.17E+05 | 1.54E+05 | NA | 0.00E+00 | NA | 2.10E+01 |
| **2341** | 0.00E+00 | 9.04E+06 | NA | NA | 4.32E+05 | 1.39E+05 | NA | 0.00E+00 | NA | 6.53E+01 |
| **2342** | 5.69E+05 | 0.00E+00 | NA | NA | 0.00E+00 | 0.00E+00 | NA | Inf | NA | NA |
| **2343** | NA | NA | NA | NA | 5.67E+05 | 0.00E+00 | NA | NA | NA | NA |
| **2344** | NA | NA | NA | NA | 5.66E+05 | 0.00E+00 | NA | NA | NA | NA |
| **2345** | 0.00E+00 | 5.65E+05 | NA | NA | NA | NA | NA | NA | NA | NA |
| **2346** | 1.91E+06 | 0.00E+00 | NA | NA | 3.10E+05 | 2.53E+05 | NA | 6.16E+00 | NA | 0.00E+00 |
| **2347** | NA | NA | NA | NA | 3.65E+05 | 1.91E+05 | NA | NA | NA | NA |
| **2348** | NA | NA | NA | NA | 5.55E+05 | 0.00E+00 | NA | NA | NA | NA |
| **2349** | 0.00E+00 | 2.53E+06 | NA | NA | 4.78E+05 | 7.41E+04 | NA | 0.00E+00 | NA | 3.41E+01 |
| **2350** | NA | NA | NA | NA | 4.64E+05 | 8.73E+04 | NA | NA | NA | NA |
| **2351** | NA | NA | NA | NA | 2.31E+05 | 3.19E+05 | NA | NA | NA | NA |
| **2352** | 0.00E+00 | 5.46E+05 | NA | NA | NA | NA | NA | NA | NA | NA |

| | | | | | | | | | | |
|---|---|---|---|---|---|---|---|---|---|---|
| **2353** | NA | NA | NA | NA | 5.43E+05 | 0.00E+00 | NA | NA | NA | NA |
| **2354** | 0.00E+00 | 5.42E+05 | NA | NA | NA | NA | NA | NA | NA | NA |
| **2355** | NA | NA | NA | NA | 1.07E+05 | 4.32E+05 | NA | NA | NA | NA |
| **2356** | NA | NA | NA | NA | 3.77E+05 | 1.58E+05 | NA | NA | NA | NA |
| **2357** | NA | NA | NA | NA | 5.34E+05 | 0.00E+00 | NA | NA | NA | NA |
| **2358** | NA | NA | NA | NA | 4.41E+05 | 8.28E+04 | NA | NA | NA | NA |
| **2359** | 1.03E+07 | 0.00E+00 | NA | NA | 5.20E+05 | 0.00E+00 | NA | 1.97E+01 | NA | NA |
| **2360** | NA | NA | NA | NA | 0.00E+00 | 5.18E+05 | NA | NA | NA | NA |
| **2361** | NA | NA | NA | NA | 3.24E+05 | 1.93E+05 | NA | NA | NA | NA |
| **2362** | 0.00E+00 | 2.15E+05 | NA | NA | 3.44E+05 | 1.71E+05 | NA | 0.00E+00 | NA | 1.26E+00 |
| **2363** | NA | NA | NA | NA | 4.07E+05 | 1.01E+05 | NA | NA | NA | NA |
| **2364** | 9.24E+06 | 1.65E+06 | NA | NA | 2.77E+05 | 2.27E+05 | NA | 3.33E+01 | NA | 7.29E+00 |
| **2365** | NA | NA | NA | NA | 5.03E+05 | 0.00E+00 | NA | NA | NA | NA |
| **2366** | NA | NA | NA | NA | 4.81E+04 | 4.52E+05 | NA | NA | NA | NA |
| **2367** | NA | NA | NA | NA | 4.98E+05 | 0.00E+00 | NA | NA | NA | NA |
| **2368** | 5.38E+06 | 0.00E+00 | NA | NA | 4.98E+05 | 0.00E+00 | NA | 1.08E+01 | NA | NA |
| **2369** | NA | NA | NA | NA | 3.78E+05 | 1.20E+05 | NA | NA | NA | NA |
| **2370** | 7.96E+05 | 0.00E+00 | NA | NA | 0.00E+00 | 4.94E+05 | NA | Inf | NA | 0.00E+00 |
| **2371** | 1.80E+05 | 0.00E+00 | NA | NA | 4.19E+05 | 7.10E+04 | NA | 4.30E-01 | NA | 0.00E+00 |
| **2372** | NA | NA | NA | NA | 4.90E+05 | 0.00E+00 | NA | NA | NA | NA |
| **2373** | 0.00E+00 | 4.85E+05 | NA | NA | NA | NA | NA | NA | NA | NA |
| **2374** | 4.83E+05 | 0.00E+00 | NA | NA | NA | NA | NA | NA | NA | NA |
| **2375** | 2.81E+06 | 0.00E+00 | NA | NA | 2.95E+05 | 1.85E+05 | NA | 9.54E+00 | NA | 0.00E+00 |
| **2376** | 0.00E+00 | 4.69E+05 | NA | NA | NA | NA | NA | NA | NA | NA |
| **2377** | NA | NA | NA | NA | 4.68E+05 | 0.00E+00 | NA | NA | NA | NA |
| **2378** | NA | NA | NA | NA | 3.32E+05 | 1.36E+05 | NA | NA | NA | NA |
| **2379** | 0.00E+00 | 4.67E+05 | NA | NA | NA | NA | NA | NA | NA | NA |
| **2380** | NA | NA | NA | NA | 2.89E+05 | 1.76E+05 | NA | NA | NA | NA |
| **2381** | NA | NA | NA | NA | 4.61E+05 | 0.00E+00 | NA | NA | NA | NA |
| **2382** | 2.28E+06 | 0.00E+00 | NA | NA | 4.58E+05 | 0.00E+00 | NA | 4.97E+00 | NA | NA |
| **2383** | 8.93E+06 | 3.88E+06 | NA | NA | 1.59E+05 | 2.97E+05 | NA | 5.62E+01 | NA | 1.31E+01 |
| **2384** | 0.00E+00 | 1.98E+06 | NA | NA | 4.54E+05 | 0.00E+00 | NA | 0.00E+00 | NA | Inf |
| **2385** | 4.54E+05 | 0.00E+00 | NA | NA | NA | NA | NA | NA | NA | NA |
| **2386** | 4.54E+05 | 0.00E+00 | NA | NA | NA | NA | NA | NA | NA | NA |
| **2387** | 0.00E+00 | 4.52E+05 | NA | NA | 0.00E+00 | 0.00E+00 | NA | NA | NA | Inf |
| **2388** | 0.00E+00 | 4.52E+05 | NA | NA | 0.00E+00 | 0.00E+00 | NA | NA | NA | Inf |
| **2389** | NA | NA | NA | NA | 4.44E+05 | 0.00E+00 | NA | NA | NA | NA |
| **2390** | 4.41E+05 | 0.00E+00 | NA | NA | NA | NA | NA | NA | NA | NA |

| | | | | | | | | | | |
|---|---|---|---|---|---|---|---|---|---|---|
| **2391** | 6.07E+06 | 0.00E+00 | NA | NA | 0.00E+00 | 4.40E+05 | NA | Inf | NA | 0.00E+00 |
| **2392** | NA | NA | NA | NA | 3.04E+05 | 1.35E+05 | NA | NA | NA | NA |
| **2393** | NA | NA | NA | NA | 4.38E+05 | 0.00E+00 | NA | NA | NA | NA |
| **2394** | NA | NA | NA | NA | 4.29E+05 | 0.00E+00 | NA | NA | NA | NA |
| **2395** | NA | NA | NA | NA | 3.86E+05 | 4.01E+04 | NA | NA | NA | NA |
| **2396** | 4.26E+05 | 0.00E+00 | NA | NA | NA | NA | NA | NA | NA | NA |
| **2397** | 2.27E+06 | 0.00E+00 | NA | NA | 4.23E+05 | 0.00E+00 | NA | 5.37E+00 | NA | NA |
| **2398** | NA | NA | NA | NA | 0.00E+00 | 4.22E+05 | NA | NA | NA | NA |
| **2399** | 0.00E+00 | 4.22E+05 | NA | NA | NA | NA | NA | NA | NA | NA |
| **2400** | NA | NA | NA | NA | 4.21E+05 | 0.00E+00 | NA | NA | NA | NA |
| **2401** | NA | NA | NA | NA | 4.16E+05 | 0.00E+00 | NA | NA | NA | NA |
| **2402** | NA | NA | NA | NA | 4.13E+05 | 0.00E+00 | NA | NA | NA | NA |
| **2403** | NA | NA | NA | NA | 4.12E+05 | 0.00E+00 | NA | NA | NA | NA |
| **2404** | NA | NA | NA | NA | 4.09E+05 | 0.00E+00 | NA | NA | NA | NA |
| **2405** | 2.26E+06 | 1.01E+08 | NA | NA | 0.00E+00 | 4.09E+05 | NA | Inf | NA | 2.46E+02 |
| **2406** | 2.74E+07 | 6.28E+06 | NA | NA | 0.00E+00 | 4.07E+05 | NA | Inf | NA | 1.54E+01 |
| **2407** | NA | NA | NA | NA | 0.00E+00 | 4.06E+05 | NA | NA | NA | NA |
| **2408** | 0.00E+00 | 4.03E+05 | NA | NA | NA | NA | NA | NA | NA | NA |
| **2409** | 4.59E+05 | 0.00E+00 | NA | NA | 4.02E+05 | 0.00E+00 | NA | 1.14E+00 | NA | NA |
| **2410** | 0.00E+00 | 3.97E+05 | NA | NA | NA | NA | NA | NA | NA | NA |
| **2411** | 1.05E+06 | 0.00E+00 | NA | NA | 3.93E+05 | 0.00E+00 | NA | 2.67E+00 | NA | NA |
| **2412** | 3.90E+05 | 0.00E+00 | NA | NA | NA | NA | NA | NA | NA | NA |
| **2413** | NA | NA | NA | NA | 0.00E+00 | 3.89E+05 | NA | NA | NA | NA |
| **2414** | 4.61E+06 | 1.74E+06 | NA | NA | 3.52E+05 | 3.42E+04 | NA | 1.31E+01 | NA | 5.08E+01 |
| **2415** | 0.00E+00 | 3.84E+05 | NA | NA | NA | NA | NA | NA | NA | NA |
| **2416** | NA | NA | NA | NA | 3.82E+05 | 0.00E+00 | NA | NA | NA | NA |
| **2417** | NA | NA | NA | NA | 3.90E+04 | 3.37E+05 | NA | NA | NA | NA |
| **2418** | 2.15E+06 | 2.38E+06 | NA | NA | 1.80E+05 | 1.94E+05 | NA | 1.19E+01 | NA | 1.22E+01 |
| **2419** | NA | NA | NA | NA | 0.00E+00 | 3.74E+05 | NA | NA | NA | NA |
| **2420** | NA | NA | NA | NA | 3.73E+05 | 0.00E+00 | NA | NA | NA | NA |
| **2421** | 0.00E+00 | 3.70E+05 | NA | NA | NA | NA | NA | NA | NA | NA |
| **2422** | 0.00E+00 | 9.47E+06 | NA | NA | 2.62E+05 | 1.07E+05 | NA | 0.00E+00 | NA | 8.88E+01 |
| **2423** | NA | NA | NA | NA | 3.68E+05 | 0.00E+00 | NA | NA | NA | NA |
| **2424** | 6.85E+06 | 0.00E+00 | NA | NA | 6.24E+04 | 3.04E+05 | NA | 1.10E+02 | NA | 0.00E+00 |
| **2425** | 1.90E+06 | 0.00E+00 | NA | NA | 0.00E+00 | 3.65E+05 | NA | Inf | NA | 0.00E+00 |
| **2426** | NA | NA | NA | NA | 2.46E+05 | 1.18E+05 | NA | NA | NA | NA |
| **2427** | NA | NA | NA | NA | 3.63E+05 | 0.00E+00 | NA | NA | NA | NA |
| **2428** | NA | NA | NA | NA | 3.61E+05 | 0.00E+00 | NA | NA | NA | NA |

| | | | | | | | | | | |
|---|---|---|---|---|---|---|---|---|---|---|
| **2429** | NA | NA | NA | NA | 3.59E+05 | 0.00E+00 | NA | NA | NA | NA |
| **2430** | NA | NA | NA | NA | 3.58E+05 | 0.00E+00 | NA | NA | NA | NA |
| **2431** | NA | NA | NA | NA | 3.57E+05 | 0.00E+00 | NA | NA | NA | NA |
| **2432** | 0.00E+00 | 3.55E+05 | NA | NA | 0.00E+00 | 0.00E+00 | NA | NA | NA | Inf |
| **2433** | NA | NA | NA | NA | 3.54E+05 | 0.00E+00 | NA | NA | NA | NA |
| **2434** | 3.53E+05 | 0.00E+00 | NA | NA | NA | NA | NA | NA | NA | NA |
| **2435** | NA | NA | NA | NA | 0.00E+00 | 3.46E+05 | NA | NA | NA | NA |
| **2436** | NA | NA | NA | NA | 3.45E+05 | 0.00E+00 | NA | NA | NA | NA |
| **2437** | NA | NA | NA | NA | 3.44E+05 | 0.00E+00 | NA | NA | NA | NA |
| **2438** | NA | NA | NA | NA | 3.41E+05 | 0.00E+00 | NA | NA | NA | NA |
| **2439** | NA | NA | NA | NA | 3.40E+05 | 0.00E+00 | NA | NA | NA | NA |
| **2440** | 3.28E+06 | 9.29E+06 | NA | NA | 3.39E+05 | 0.00E+00 | NA | 9.68E+00 | NA | Inf |
| **2441** | 9.72E+05 | 0.00E+00 | NA | NA | 2.89E+05 | 4.43E+04 | NA | 3.36E+00 | NA | 0.00E+00 |
| **2442** | NA | NA | NA | NA | 8.93E+04 | 2.43E+05 | NA | NA | NA | NA |
| **2443** | NA | NA | NA | NA | 3.32E+05 | 0.00E+00 | NA | NA | NA | NA |
| **2444** | 2.70E+06 | 0.00E+00 | NA | NA | 9.61E+04 | 2.36E+05 | NA | 2.81E+01 | NA | 0.00E+00 |
| **2445** | NA | NA | NA | NA | 1.35E+05 | 1.96E+05 | NA | NA | NA | NA |
| **2446** | 8.11E+06 | 0.00E+00 | NA | NA | 0.00E+00 | 3.31E+05 | NA | Inf | NA | 0.00E+00 |
| **2447** | NA | NA | NA | NA | 3.25E+05 | 0.00E+00 | NA | NA | NA | NA |
| **2448** | 7.57E+06 | 9.49E+05 | NA | NA | 0.00E+00 | 3.24E+05 | NA | Inf | NA | 2.93E+00 |
| **2449** | NA | NA | NA | NA | 3.23E+05 | 0.00E+00 | NA | NA | NA | NA |
| **2450** | NA | NA | NA | NA | 0.00E+00 | 3.21E+05 | NA | NA | NA | NA |
| **2451** | 0.00E+00 | 3.20E+05 | NA | NA | NA | NA | NA | NA | NA | NA |
| **2452** | 3.38E+06 | 0.00E+00 | NA | NA | 0.00E+00 | 3.19E+05 | NA | Inf | NA | 0.00E+00 |
| **2453** | 3.71E+06 | 0.00E+00 | NA | NA | 0.00E+00 | 3.19E+05 | NA | Inf | NA | 0.00E+00 |
| **2454** | NA | NA | NA | NA | 3.17E+05 | 0.00E+00 | NA | NA | NA | NA |
| **2455** | NA | NA | NA | NA | 3.16E+05 | 0.00E+00 | NA | NA | NA | NA |
| **2456** | NA | NA | NA | NA | 0.00E+00 | 3.14E+05 | NA | NA | NA | NA |
| **2457** | 9.29E+05 | 0.00E+00 | NA | NA | 2.34E+05 | 7.95E+04 | NA | 3.97E+00 | NA | 0.00E+00 |
| **2458** | NA | NA | NA | NA | 3.13E+05 | 0.00E+00 | NA | NA | NA | NA |
| **2459** | NA | NA | NA | NA | 3.13E+05 | 0.00E+00 | NA | NA | NA | NA |
| **2460** | NA | NA | NA | NA | 3.12E+05 | 0.00E+00 | NA | NA | NA | NA |
| **2461** | NA | NA | NA | NA | 3.06E+05 | 0.00E+00 | NA | NA | NA | NA |
| **2462** | 2.12E+06 | 3.41E+07 | NA | NA | 3.05E+05 | 0.00E+00 | NA | 6.94E+00 | NA | Inf |
| **2463** | 1.27E+06 | 0.00E+00 | NA | NA | 3.05E+05 | 0.00E+00 | NA | 4.16E+00 | NA | NA |
| **2464** | 0.00E+00 | 3.04E+05 | NA | NA | NA | NA | NA | NA | NA | NA |
| **2465** | 0.00E+00 | 1.71E+06 | NA | NA | 0.00E+00 | 3.03E+05 | NA | NA | NA | 5.64E+00 |
| **2466** | NA | NA | NA | NA | 3.02E+05 | 0.00E+00 | NA | NA | NA | NA |

| | | | | | | | | | | |
|---|---|---|---|---|---|---|---|---|---|---|
| **2467** | 0.00E+00 | 3.01E+05 | NA | NA | NA | NA | NA | NA | NA | NA |
| **2468** | 0.00E+00 | 2.97E+05 | NA | NA | NA | NA | NA | NA | NA | NA |
| **2469** | NA | NA | NA | NA | 2.94E+05 | 0.00E+00 | NA | NA | NA | NA |
| **2470** | 0.00E+00 | 5.11E+06 | NA | NA | 6.57E+04 | 2.27E+05 | NA | 0.00E+00 | NA | 2.25E+01 |
| **2471** | NA | NA | NA | NA | 2.92E+05 | 0.00E+00 | NA | NA | NA | NA |
| **2472** | NA | NA | NA | NA | 2.91E+05 | 0.00E+00 | NA | NA | NA | NA |
| **2473** | NA | NA | NA | NA | 5.11E+04 | 2.40E+05 | NA | NA | NA | NA |
| **2474** | 0.00E+00 | 2.90E+05 | NA | NA | NA | NA | NA | NA | NA | NA |
| **2475** | 2.90E+05 | 0.00E+00 | NA | NA | NA | NA | NA | NA | NA | NA |
| **2476** | NA | NA | NA | NA | 2.85E+05 | 0.00E+00 | NA | NA | NA | NA |
| **2477** | NA | NA | NA | NA | 2.83E+05 | 0.00E+00 | NA | NA | NA | NA |
| **2478** | NA | NA | NA | NA | 2.83E+05 | 0.00E+00 | NA | NA | NA | NA |
| **2479** | 1.95E+06 | 6.66E+06 | NA | NA | 2.83E+05 | 0.00E+00 | NA | 6.90E+00 | NA | Inf |
| **2480** | NA | NA | NA | NA | 0.00E+00 | 2.81E+05 | NA | NA | NA | NA |
| **2481** | 2.77E+05 | 0.00E+00 | NA | NA | NA | NA | NA | NA | NA | NA |
| **2482** | NA | NA | NA | NA | 1.84E+05 | 9.21E+04 | NA | NA | NA | NA |
| **2483** | NA | NA | NA | NA | 2.74E+05 | 0.00E+00 | NA | NA | NA | NA |
| **2484** | NA | NA | NA | NA | 0.00E+00 | 2.70E+05 | NA | NA | NA | NA |
| **2485** | 5.78E+06 | 0.00E+00 | NA | NA | 0.00E+00 | 2.69E+05 | NA | Inf | NA | 0.00E+00 |
| **2486** | NA | NA | NA | NA | 1.01E+05 | 1.67E+05 | NA | NA | NA | NA |
| **2487** | NA | NA | NA | NA | 0.00E+00 | 2.68E+05 | NA | NA | NA | NA |
| **2488** | NA | NA | NA | NA | 2.68E+05 | 0.00E+00 | NA | NA | NA | NA |
| **2489** | NA | NA | NA | NA | 2.64E+05 | 0.00E+00 | NA | NA | NA | NA |
| **2490** | 2.61E+05 | 0.00E+00 | NA | NA | NA | NA | NA | NA | NA | NA |
| **2491** | NA | NA | NA | NA | 1.91E+05 | 6.93E+04 | NA | NA | NA | NA |
| **2492** | NA | NA | NA | NA | 2.60E+05 | 0.00E+00 | NA | NA | NA | NA |
| **2493** | 0.00E+00 | 2.59E+05 | NA | NA | NA | NA | NA | NA | NA | NA |
| **2494** | NA | NA | NA | NA | 0.00E+00 | 2.58E+05 | NA | NA | NA | NA |
| **2495** | NA | NA | NA | NA | 2.58E+05 | 0.00E+00 | NA | NA | NA | NA |
| **2496** | 0.00E+00 | 2.58E+05 | NA | NA | NA | NA | NA | NA | NA | NA |
| **2497** | NA | NA | NA | NA | 0.00E+00 | 2.57E+05 | NA | NA | NA | NA |
| **2498** | 3.49E+05 | 8.28E+06 | NA | NA | 0.00E+00 | 2.55E+05 | NA | Inf | NA | 3.25E+01 |
| **2499** | NA | NA | NA | NA | 2.54E+05 | 0.00E+00 | NA | NA | NA | NA |
| **2500** | 0.00E+00 | 2.92E+07 | NA | NA | 2.54E+05 | 0.00E+00 | NA | 0.00E+00 | NA | Inf |
| **2501** | 2.53E+05 | 0.00E+00 | NA | NA | NA | NA | NA | NA | NA | NA |
| **2502** | 2.29E+06 | 0.00E+00 | NA | NA | 0.00E+00 | 2.51E+05 | NA | Inf | NA | 0.00E+00 |
| **2503** | 4.10E+06 | 0.00E+00 | NA | NA | 1.70E+05 | 7.81E+04 | NA | 2.41E+01 | NA | 0.00E+00 |
| **2504** | NA | NA | NA | NA | 2.47E+05 | 0.00E+00 | NA | NA | NA | NA |

| | | | | | | | | | | |
|---|---|---|---|---|---|---|---|---|---|---|
| **2505** | 6.73E+06 | 0.00E+00 | NA | NA | 0.00E+00 | 2.44E+05 | NA | Inf | NA | 0.00E+00 |
| **2506** | NA | NA | NA | NA | 1.97E+05 | 4.36E+04 | NA | NA | NA | NA |
| **2507** | 8.47E+05 | 1.73E+06 | NA | NA | 6.87E+04 | 1.72E+05 | NA | 1.23E+01 | NA | 1.01E+01 |
| **2508** | NA | NA | NA | NA | 1.91E+05 | 4.96E+04 | NA | NA | NA | NA |
| **2509** | 4.57E+06 | 0.00E+00 | NA | NA | 0.00E+00 | 2.40E+05 | NA | Inf | NA | 0.00E+00 |
| **2510** | NA | NA | NA | NA | 2.35E+05 | 0.00E+00 | NA | NA | NA | NA |
| **2511** | 6.05E+05 | 0.00E+00 | NA | NA | 2.34E+05 | 0.00E+00 | NA | 2.58E+00 | NA | NA |
| **2512** | NA | NA | NA | NA | 2.34E+05 | 0.00E+00 | NA | NA | NA | NA |
| **2513** | NA | NA | NA | NA | 2.33E+05 | 0.00E+00 | NA | NA | NA | NA |
| **2514** | 2.32E+05 | 0.00E+00 | NA | NA | NA | NA | NA | NA | NA | NA |
| **2515** | NA | NA | NA | NA | 2.29E+05 | 0.00E+00 | NA | NA | NA | NA |
| **2516** | 2.35E+06 | 0.00E+00 | NA | NA | 1.45E+05 | 8.40E+04 | NA | 1.62E+01 | NA | 0.00E+00 |
| **2517** | 0.00E+00 | 2.29E+05 | NA | NA | NA | NA | NA | NA | NA | NA |
| **2518** | 0.00E+00 | 2.28E+05 | NA | NA | 0.00E+00 | 0.00E+00 | NA | NA | NA | Inf |
| **2519** | NA | NA | NA | NA | 2.27E+05 | 0.00E+00 | NA | NA | NA | NA |
| **2520** | 0.00E+00 | 1.46E+06 | NA | NA | 0.00E+00 | 2.26E+05 | NA | NA | NA | 6.43E+00 |
| **2521** | NA | NA | NA | NA | 2.24E+05 | 0.00E+00 | NA | NA | NA | NA |
| **2522** | NA | NA | NA | NA | 2.24E+05 | 0.00E+00 | NA | NA | NA | NA |
| **2523** | 3.25E+06 | 0.00E+00 | NA | NA | 0.00E+00 | 2.20E+05 | NA | Inf | NA | 0.00E+00 |
| **2524** | 0.00E+00 | 0.00E+00 | NA | NA | 2.18E+05 | 0.00E+00 | NA | 0.00E+00 | NA | NA |
| **2525** | NA | NA | NA | NA | 2.18E+05 | 0.00E+00 | NA | NA | NA | NA |
| **2526** | NA | NA | NA | NA | 1.60E+05 | 5.43E+04 | NA | NA | NA | NA |
| **2527** | 6.06E+05 | 0.00E+00 | NA | NA | 1.69E+05 | 4.42E+04 | NA | 3.59E+00 | NA | 0.00E+00 |
| **2528** | NA | NA | NA | NA | 2.11E+05 | 0.00E+00 | NA | NA | NA | NA |
| **2529** | NA | NA | NA | NA | 2.11E+05 | 0.00E+00 | NA | NA | NA | NA |
| **2530** | NA | NA | NA | NA | 0.00E+00 | 2.11E+05 | NA | NA | NA | NA |
| **2531** | NA | NA | NA | NA | 2.10E+05 | 0.00E+00 | NA | NA | NA | NA |
| **2532** | NA | NA | NA | NA | 2.10E+05 | 0.00E+00 | NA | NA | NA | NA |
| **2533** | NA | NA | NA | NA | 2.09E+05 | 0.00E+00 | NA | NA | NA | NA |
| **2534** | 2.09E+05 | 0.00E+00 | NA | NA | NA | NA | NA | NA | NA | NA |
| **2535** | NA | NA | NA | NA | 2.08E+05 | 0.00E+00 | NA | NA | NA | NA |
| **2536** | 0.00E+00 | 1.64E+06 | NA | NA | 1.02E+05 | 1.06E+05 | NA | 0.00E+00 | NA | 1.55E+01 |
| **2537** | 0.00E+00 | 1.96E+06 | NA | NA | 2.07E+05 | 0.00E+00 | NA | 0.00E+00 | NA | Inf |
| **2538** | NA | NA | NA | NA | 2.07E+05 | 0.00E+00 | NA | NA | NA | NA |
| **2539** | NA | NA | NA | NA | 2.07E+05 | 0.00E+00 | NA | NA | NA | NA |
| **2540** | NA | NA | 0.00E+00 | 0.00E+00 | 2.06E+05 | 0.00E+00 | NA | NA | NA | NA |
| **2541** | NA | NA | NA | NA | 2.06E+05 | 0.00E+00 | NA | NA | NA | NA |
| **2542** | NA | NA | NA | NA | 2.04E+05 | 0.00E+00 | NA | NA | NA | NA |

| | | | | | | | | | | |
|---|---|---|---|---|---|---|---|---|---|---|
| **2543** | NA | NA | NA | NA | 2.04E+05 | 0.00E+00 | NA | NA | NA | NA |
| **2544** | 7.31E+06 | 0.00E+00 | NA | NA | 0.00E+00 | 2.03E+05 | NA | Inf | NA | 0.00E+00 |
| **2545** | NA | NA | NA | NA | 2.02E+05 | 0.00E+00 | NA | NA | NA | NA |
| **2546** | NA | NA | NA | NA | 2.00E+05 | 0.00E+00 | NA | NA | NA | NA |
| **2547** | NA | NA | NA | NA | 4.75E+04 | 1.52E+05 | NA | NA | NA | NA |
| **2548** | NA | NA | NA | NA | 1.99E+05 | 0.00E+00 | NA | NA | NA | NA |
| **2549** | NA | NA | NA | NA | 1.99E+05 | 0.00E+00 | NA | NA | NA | NA |
| **2550** | NA | NA | NA | NA | 0.00E+00 | 1.98E+05 | NA | NA | NA | NA |
| **2551** | NA | NA | NA | NA | 8.25E+04 | 1.15E+05 | NA | NA | NA | NA |
| **2552** | NA | NA | NA | NA | 0.00E+00 | 1.97E+05 | NA | NA | NA | NA |
| **2553** | 0.00E+00 | 1.24E+06 | NA | NA | 1.95E+05 | 0.00E+00 | NA | 0.00E+00 | NA | Inf |
| **2554** | NA | NA | NA | NA | 1.95E+05 | 0.00E+00 | NA | NA | NA | NA |
| **2555** | 3.45E+06 | 0.00E+00 | NA | NA | 1.93E+05 | 0.00E+00 | NA | 1.79E+01 | NA | NA |
| **2556** | NA | NA | NA | NA | 0.00E+00 | 1.93E+05 | NA | NA | NA | NA |
| **2557** | 8.54E+06 | 0.00E+00 | NA | NA | 1.92E+05 | 0.00E+00 | NA | 4.44E+01 | NA | NA |
| **2558** | NA | NA | NA | NA | 0.00E+00 | 1.89E+05 | NA | NA | NA | NA |
| **2559** | NA | NA | NA | NA | 0.00E+00 | 1.89E+05 | NA | NA | NA | NA |
| **2560** | NA | NA | NA | NA | 0.00E+00 | 1.89E+05 | NA | NA | NA | NA |
| **2561** | 1.92E+06 | 0.00E+00 | NA | NA | 0.00E+00 | 1.88E+05 | NA | Inf | NA | 0.00E+00 |
| **2562** | 2.23E+05 | 0.00E+00 | NA | NA | 1.87E+05 | 0.00E+00 | NA | 1.19E+00 | NA | NA |
| **2563** | NA | NA | NA | NA | 1.87E+05 | 0.00E+00 | NA | NA | NA | NA |
| **2564** | NA | NA | NA | NA | 0.00E+00 | 1.84E+05 | NA | NA | NA | NA |
| **2565** | NA | NA | NA | NA | 0.00E+00 | 1.84E+05 | NA | NA | NA | NA |
| **2566** | NA | NA | NA | NA | 0.00E+00 | 1.83E+05 | NA | NA | NA | NA |
| **2567** | NA | NA | NA | NA | 1.81E+05 | 0.00E+00 | NA | NA | NA | NA |
| **2568** | NA | NA | NA | NA | 1.80E+05 | 0.00E+00 | NA | NA | NA | NA |
| **2569** | 2.67E+06 | 0.00E+00 | NA | NA | 1.80E+05 | 0.00E+00 | NA | 1.48E+01 | NA | NA |
| **2570** | NA | NA | NA | NA | 0.00E+00 | 1.80E+05 | NA | NA | NA | NA |
| **2571** | 9.20E+05 | 6.43E+06 | NA | NA | 0.00E+00 | 1.78E+05 | NA | Inf | NA | 3.62E+01 |
| **2572** | NA | NA | NA | NA | 1.15E+05 | 6.15E+04 | NA | NA | NA | NA |
| **2573** | 3.10E+06 | 0.00E+00 | NA | NA | 0.00E+00 | 1.73E+05 | NA | Inf | NA | 0.00E+00 |
| **2574** | 5.88E+06 | 0.00E+00 | NA | NA | 0.00E+00 | 1.73E+05 | NA | Inf | NA | 0.00E+00 |
| **2575** | NA | NA | NA | NA | 1.71E+05 | 0.00E+00 | NA | NA | NA | NA |
| **2576** | NA | NA | NA | NA | 1.71E+05 | 0.00E+00 | NA | NA | NA | NA |
| **2577** | NA | NA | NA | NA | 0.00E+00 | 1.69E+05 | NA | NA | NA | NA |
| **2578** | 6.45E+06 | 0.00E+00 | NA | NA | 0.00E+00 | 1.68E+05 | NA | Inf | NA | 0.00E+00 |
| **2579** | NA | NA | NA | NA | 0.00E+00 | 1.68E+05 | NA | NA | NA | NA |
| **2580** | NA | NA | NA | NA | 1.67E+05 | 0.00E+00 | NA | NA | NA | NA |

| | | | | | | | | | | |
|---|---|---|---|---|---|---|---|---|---|---|
| **2581** | NA | NA | NA | NA | 9.15E+04 | 7.37E+04 | NA | NA | NA | NA |
| **2582** | NA | NA | NA | NA | 1.65E+05 | 0.00E+00 | NA | NA | NA | NA |
| **2583** | NA | NA | NA | NA | 0.00E+00 | 1.64E+05 | NA | NA | NA | NA |
| **2584** | NA | NA | NA | NA | 0.00E+00 | 1.64E+05 | NA | NA | NA | NA |
| **2585** | NA | NA | NA | NA | 1.63E+05 | 0.00E+00 | NA | NA | NA | NA |
| **2586** | NA | NA | NA | NA | 0.00E+00 | 1.63E+05 | NA | NA | NA | NA |
| **2587** | NA | NA | NA | NA | 1.59E+05 | 0.00E+00 | NA | NA | NA | NA |
| **2588** | NA | NA | NA | NA | 0.00E+00 | 1.59E+05 | NA | NA | NA | NA |
| **2589** | 1.11E+06 | 0.00E+00 | NA | NA | 1.21E+05 | 3.61E+04 | NA | 9.17E+00 | NA | 0.00E+00 |
| **2590** | NA | NA | NA | NA | 5.54E+04 | 9.91E+04 | NA | NA | NA | NA |
| **2591** | NA | NA | NA | NA | 0.00E+00 | 1.51E+05 | NA | NA | NA | NA |
| **2592** | NA | NA | NA | NA | 1.48E+05 | 0.00E+00 | NA | NA | NA | NA |
| **2593** | 0.00E+00 | 1.48E+05 | NA | NA | NA | NA | NA | NA | NA | NA |
| **2594** | NA | NA | NA | NA | 1.45E+05 | 0.00E+00 | NA | NA | NA | NA |
| **2595** | 0.00E+00 | 1.44E+05 | NA | NA | NA | NA | NA | NA | NA | NA |
| **2596** | NA | NA | NA | NA | 1.44E+05 | 0.00E+00 | NA | NA | NA | NA |
| **2597** | NA | NA | NA | NA | 1.44E+05 | 0.00E+00 | NA | NA | NA | NA |
| **2598** | NA | NA | NA | NA | 0.00E+00 | 1.44E+05 | NA | NA | NA | NA |
| **2599** | NA | NA | NA | NA | 1.42E+05 | 0.00E+00 | NA | NA | NA | NA |
| **2600** | NA | NA | NA | NA | 1.41E+05 | 0.00E+00 | NA | NA | NA | NA |
| **2601** | 2.25E+05 | 0.00E+00 | NA | NA | 0.00E+00 | 1.41E+05 | NA | Inf | NA | 0.00E+00 |
| **2602** | 0.00E+00 | 0.00E+00 | NA | NA | 7.36E+04 | 6.64E+04 | NA | 0.00E+00 | NA | 0.00E+00 |
| **2603** | NA | NA | NA | NA | 1.39E+05 | 0.00E+00 | NA | NA | NA | NA |
| **2604** | NA | NA | NA | NA | 0.00E+00 | 1.39E+05 | NA | NA | NA | NA |
| **2605** | NA | NA | NA | NA | 1.38E+05 | 0.00E+00 | NA | NA | NA | NA |
| **2606** | 1.01E+07 | 0.00E+00 | NA | NA | 0.00E+00 | 1.36E+05 | NA | Inf | NA | 0.00E+00 |
| **2607** | NA | NA | NA | NA | 1.34E+05 | 0.00E+00 | NA | NA | NA | NA |
| **2608** | NA | NA | NA | NA | 1.32E+05 | 0.00E+00 | NA | NA | NA | NA |
| **2609** | NA | NA | NA | NA | 0.00E+00 | 1.30E+05 | NA | NA | NA | NA |
| **2610** | NA | NA | NA | NA | 1.28E+05 | 0.00E+00 | NA | NA | NA | NA |
| **2611** | 3.36E+05 | 0.00E+00 | NA | NA | 1.28E+05 | 0.00E+00 | NA | 2.62E+00 | NA | NA |
| **2612** | NA | NA | NA | NA | 0.00E+00 | 1.28E+05 | NA | NA | NA | NA |
| **2613** | NA | NA | NA | NA | 5.36E+04 | 7.31E+04 | NA | NA | NA | NA |
| **2614** | 4.38E+06 | 0.00E+00 | NA | NA | 0.00E+00 | 1.23E+05 | NA | Inf | NA | 0.00E+00 |
| **2615** | NA | NA | NA | NA | 0.00E+00 | 1.22E+05 | NA | NA | NA | NA |
| **2616** | NA | NA | NA | NA | 0.00E+00 | 1.20E+05 | NA | NA | NA | NA |
| **2617** | NA | NA | NA | NA | 0.00E+00 | 1.16E+05 | NA | NA | NA | NA |
| **2618** | 0.00E+00 | 6.66E+06 | NA | NA | 1.15E+05 | 0.00E+00 | NA | 0.00E+00 | NA | Inf |

| | | | | | | | | | | |
|---|---|---|---|---|---|---|---|---|---|---|
| **2619** | NA | NA | NA | NA | 1.14E+05 | 0.00E+00 | NA | NA | NA | NA |
| **2620** | NA | NA | NA | NA | 0.00E+00 | 1.14E+05 | NA | NA | NA | NA |
| **2621** | NA | NA | NA | NA | 0.00E+00 | 1.11E+05 | NA | NA | NA | NA |
| **2622** | 2.11E+06 | 0.00E+00 | NA | NA | 0.00E+00 | 1.09E+05 | NA | Inf | NA | 0.00E+00 |
| **2623** | NA | NA | NA | NA | 0.00E+00 | 1.09E+05 | NA | NA | NA | NA |
| **2624** | NA | NA | NA | NA | 1.09E+05 | 0.00E+00 | NA | NA | NA | NA |
| **2625** | NA | NA | NA | NA | 1.08E+05 | 0.00E+00 | NA | NA | NA | NA |
| **2626** | NA | NA | NA | NA | 0.00E+00 | 1.08E+05 | NA | NA | NA | NA |
| **2627** | 0.00E+00 | 1.59E+07 | NA | NA | 0.00E+00 | 1.07E+05 | NA | NA | NA | 1.49E+02 |
| **2628** | NA | NA | NA | NA | 1.07E+05 | 0.00E+00 | NA | NA | NA | NA |
| **2629** | NA | NA | NA | NA | 1.01E+05 | 0.00E+00 | NA | NA | NA | NA |
| **2630** | 0.00E+00 | 9.97E+04 | NA | NA | NA | NA | NA | NA | NA | NA |
| **2631** | NA | NA | NA | NA | 0.00E+00 | 9.83E+04 | NA | NA | NA | NA |
| **2632** | NA | NA | NA | NA | 9.64E+04 | 0.00E+00 | NA | NA | NA | NA |
| **2633** | NA | NA | NA | NA | 0.00E+00 | 9.44E+04 | NA | NA | NA | NA |
| **2634** | NA | NA | NA | NA | 0.00E+00 | 9.27E+04 | NA | NA | NA | NA |
| **2635** | NA | NA | NA | NA | 9.23E+04 | 0.00E+00 | NA | NA | NA | NA |
| **2636** | 0.00E+00 | 2.19E+06 | NA | NA | 9.06E+04 | 0.00E+00 | NA | 0.00E+00 | NA | Inf |
| **2637** | NA | NA | NA | NA | 0.00E+00 | 8.87E+04 | NA | NA | NA | NA |
| **2638** | 0.00E+00 | 0.00E+00 | NA | NA | 0.00E+00 | 8.85E+04 | NA | NA | NA | 0.00E+00 |
| **2639** | NA | NA | NA | NA | 8.84E+04 | 0.00E+00 | NA | NA | NA | NA |
| **2640** | NA | NA | NA | NA | 0.00E+00 | 8.84E+04 | NA | NA | NA | NA |
| **2641** | NA | NA | NA | NA | 8.83E+04 | 0.00E+00 | NA | NA | NA | NA |
| **2642** | NA | NA | NA | NA | 8.64E+04 | 0.00E+00 | NA | NA | NA | NA |
| **2643** | 0.00E+00 | 5.83E+06 | NA | NA | 8.60E+04 | 0.00E+00 | NA | 0.00E+00 | NA | Inf |
| **2644** | 6.91E+05 | 0.00E+00 | NA | NA | 0.00E+00 | 8.47E+04 | NA | Inf | NA | 0.00E+00 |
| **2645** | NA | NA | NA | NA | 0.00E+00 | 8.25E+04 | NA | NA | NA | NA |
| **2646** | NA | NA | NA | NA | 0.00E+00 | 8.11E+04 | NA | NA | NA | NA |
| **2647** | NA | NA | NA | NA | 0.00E+00 | 8.10E+04 | NA | NA | NA | NA |
| **2648** | NA | NA | NA | NA | 0.00E+00 | 8.07E+04 | NA | NA | NA | NA |
| **2649** | NA | NA | NA | NA | 7.93E+04 | 0.00E+00 | NA | NA | NA | NA |
| **2650** | NA | NA | NA | NA | 0.00E+00 | 7.89E+04 | NA | NA | NA | NA |
| **2651** | NA | NA | NA | NA | 0.00E+00 | 7.77E+04 | NA | NA | NA | NA |
| **2652** | NA | NA | NA | NA | 0.00E+00 | 7.61E+04 | NA | NA | NA | NA |
| **2653** | NA | NA | NA | NA | 0.00E+00 | 7.53E+04 | NA | NA | NA | NA |
| **2654** | 0.00E+00 | 0.00E+00 | NA | NA | 0.00E+00 | 7.40E+04 | NA | NA | NA | 0.00E+00 |
| **2655** | NA | NA | NA | NA | 7.39E+04 | 0.00E+00 | NA | NA | NA | NA |
| **2656** | NA | NA | NA | NA | 7.31E+04 | 0.00E+00 | NA | NA | NA | NA |

| | | | | | | | | | | |
|---|---|---|---|---|---|---|---|---|---|---|
| **2657** | NA | NA | NA | NA | 0.00E+00 | 7.16E+04 | NA | NA | NA | NA |
| **2658** | 0.00E+00 | 1.57E+06 | NA | NA | 7.07E+04 | 0.00E+00 | NA | 0.00E+00 | NA | Inf |
| **2659** | NA | NA | NA | NA | 7.03E+04 | 0.00E+00 | NA | NA | NA | NA |
| **2660** | NA | NA | NA | NA | 0.00E+00 | 6.99E+04 | NA | NA | NA | NA |
| **2661** | 1.46E+06 | 0.00E+00 | NA | NA | 0.00E+00 | 6.95E+04 | NA | Inf | NA | 0.00E+00 |
| **2662** | 1.24E+06 | 7.08E+06 | NA | NA | 0.00E+00 | 6.93E+04 | NA | Inf | NA | 1.02E+02 |
| **2663** | NA | NA | NA | NA | 0.00E+00 | 6.90E+04 | NA | NA | NA | NA |
| **2664** | NA | NA | NA | NA | 6.87E+04 | 0.00E+00 | NA | NA | NA | NA |
| **2665** | NA | NA | NA | NA | 0.00E+00 | 6.81E+04 | NA | NA | NA | NA |
| **2666** | NA | NA | NA | NA | 0.00E+00 | 6.69E+04 | NA | NA | NA | NA |
| **2667** | NA | NA | NA | NA | 0.00E+00 | 6.59E+04 | NA | NA | NA | NA |
| **2668** | NA | NA | NA | NA | 0.00E+00 | 6.56E+04 | NA | NA | NA | NA |
| **2669** | NA | NA | NA | NA | 0.00E+00 | 6.51E+04 | NA | NA | NA | NA |
| **2670** | NA | NA | NA | NA | 6.27E+04 | 0.00E+00 | NA | NA | NA | NA |
| **2671** | NA | NA | NA | NA | 6.21E+04 | 0.00E+00 | NA | NA | NA | NA |
| **2672** | NA | NA | NA | NA | 0.00E+00 | 6.16E+04 | NA | NA | NA | NA |
| **2673** | NA | NA | NA | NA | 0.00E+00 | 6.11E+04 | NA | NA | NA | NA |
| **2674** | NA | NA | NA | NA | 6.08E+04 | 0.00E+00 | NA | NA | NA | NA |
| **2675** | 0.00E+00 | 2.54E+06 | NA | NA | 6.06E+04 | 0.00E+00 | NA | 0.00E+00 | NA | Inf |
| **2676** | NA | NA | NA | NA | 0.00E+00 | 6.02E+04 | NA | NA | NA | NA |
| **2677** | NA | NA | NA | NA | 0.00E+00 | 5.88E+04 | NA | NA | NA | NA |
| **2678** | NA | NA | NA | NA | 5.87E+04 | 0.00E+00 | NA | NA | NA | NA |
| **2679** | 3.11E+05 | 2.24E+06 | NA | NA | 0.00E+00 | 5.85E+04 | NA | Inf | NA | 3.84E+01 |
| **2680** | NA | NA | NA | NA | 0.00E+00 | 5.81E+04 | NA | NA | NA | NA |
| **2681** | NA | NA | NA | NA | 0.00E+00 | 5.70E+04 | NA | NA | NA | NA |
| **2682** | 0.00E+00 | 7.03E+06 | NA | NA | 0.00E+00 | 5.66E+04 | NA | NA | NA | 1.24E+02 |
| **2683** | NA | NA | NA | NA | 0.00E+00 | 5.64E+04 | NA | NA | NA | NA |
| **2684** | 3.52E+05 | 0.00E+00 | NA | NA | 0.00E+00 | 5.61E+04 | NA | Inf | NA | 0.00E+00 |
| **2685** | 4.25E+05 | 7.40E+07 | NA | NA | 5.40E+04 | 0.00E+00 | NA | 7.86E+00 | NA | Inf |
| **2686** | 3.19E+06 | 0.00E+00 | NA | NA | 0.00E+00 | 5.40E+04 | NA | Inf | NA | 0.00E+00 |
| **2687** | NA | NA | NA | NA | 0.00E+00 | 5.30E+04 | NA | NA | NA | NA |
| **2688** | NA | NA | NA | NA | 0.00E+00 | 5.20E+04 | NA | NA | NA | NA |
| **2689** | NA | NA | NA | NA | 5.10E+04 | 0.00E+00 | NA | NA | NA | NA |
| **2690** | NA | NA | NA | NA | 0.00E+00 | 4.90E+04 | NA | NA | NA | NA |
| **2691** | NA | NA | NA | NA | 4.82E+04 | 0.00E+00 | NA | NA | NA | NA |
| **2692** | 4.80E+04 | 0.00E+00 | NA | NA | NA | NA | NA | NA | NA | NA |
| **2693** | 2.08E+06 | 0.00E+00 | NA | NA | 0.00E+00 | 4.78E+04 | NA | Inf | NA | 0.00E+00 |
| **2694** | 1.38E+06 | 0.00E+00 | NA | NA | 0.00E+00 | 4.76E+04 | NA | Inf | NA | 0.00E+00 |

| | | | | | | | | | | |
|---|---|---|---|---|---|---|---|---|---|---|
| **2695** | 1.02E+06 | 2.67E+07 | NA | NA | 0.00E+00 | 4.66E+04 | NA | Inf | NA | 5.73E+02 |
| **2696** | NA | NA | NA | NA | 4.55E+04 | 0.00E+00 | NA | NA | NA | NA |
| **2697** | NA | NA | NA | NA | 0.00E+00 | 4.54E+04 | NA | NA | NA | NA |
| **2698** | 1.57E+06 | 0.00E+00 | NA | NA | 0.00E+00 | 4.52E+04 | NA | Inf | NA | 0.00E+00 |
| **2699** | NA | NA | NA | NA | 4.51E+04 | 0.00E+00 | NA | NA | NA | NA |
| **2700** | 0.00E+00 | 5.06E+05 | NA | NA | 0.00E+00 | 4.50E+04 | NA | NA | NA | 1.12E+01 |
| **2701** | NA | NA | NA | NA | 4.08E+04 | 0.00E+00 | NA | NA | NA | NA |
| **2702** | 1.23E+06 | 0.00E+00 | NA | NA | 0.00E+00 | 4.04E+04 | NA | Inf | NA | 0.00E+00 |
| **2703** | NA | NA | NA | NA | 0.00E+00 | 3.85E+04 | NA | NA | NA | NA |
| **2704** | NA | NA | NA | NA | 0.00E+00 | 3.46E+04 | NA | NA | NA | NA |
| **2705** | NA | NA | NA | NA | 0.00E+00 | 2.96E+04 | NA | NA | NA | NA |
| **2706** | NA | NA | NA | NA | 0.00E+00 | 2.82E+04 | NA | NA | NA | NA |
| **2707** | NA | NA | NA | NA | 0.00E+00 | 2.68E+04 | NA | NA | NA | NA |
| **2708** | 4.40E+05 | 0.00E+00 | NA | NA | 2.55E+04 | 0.00E+00 | NA | 1.72E+01 | NA | NA |
| **2709** | 4.08E+05 | 7.89E+07 | NA | NA | 0.00E+00 | 0.00E+00 | NA | Inf | NA | Inf |
| **2710** | 2.44E+05 | 2.53E+07 | NA | NA | NA | NA | NA | NA | NA | NA |
| **2711** | 4.44E+05 | 4.52E+07 | NA | NA | 0.00E+00 | 0.00E+00 | NA | Inf | NA | Inf |
| **2712** | 3.56E+05 | 1.49E+07 | NA | NA | 0.00E+00 | 0.00E+00 | NA | Inf | NA | Inf |
| **2713** | 6.60E+05 | 2.47E+06 | NA | NA | 0.00E+00 | 0.00E+00 | NA | Inf | NA | Inf |
| **2714** | 5.74E+05 | 1.78E+06 | NA | NA | NA | NA | NA | NA | NA | NA |
| **2715** | 4.74E+05 | 1.34E+06 | NA | NA | 0.00E+00 | 0.00E+00 | NA | Inf | NA | Inf |
| **2716** | 2.74E+06 | 7.13E+06 | NA | NA | 0.00E+00 | 0.00E+00 | NA | Inf | NA | Inf |
| **2717** | 1.79E+06 | 3.46E+06 | NA | NA | NA | NA | NA | NA | NA | NA |
| **2718** | 2.18E+06 | 4.07E+06 | NA | NA | NA | NA | NA | NA | NA | NA |
| **2719** | 2.85E+05 | 3.16E+05 | NA | NA | NA | NA | NA | NA | NA | NA |
| **2720** | 4.92E+06 | 5.34E+06 | NA | NA | 0.00E+00 | 0.00E+00 | NA | Inf | NA | Inf |
| **2721** | 1.47E+06 | 7.54E+05 | NA | NA | 0.00E+00 | 0.00E+00 | NA | Inf | NA | Inf |
| **2722** | 1.61E+07 | 8.23E+06 | NA | NA | NA | NA | NA | NA | NA | NA |
| **2723** | 1.15E+07 | 3.48E+05 | NA | NA | NA | NA | NA | NA | NA | NA |

**Fold Changes**

| Row Number | Wash02GRvsWash01GR | Wash02PCFTvsWash01PCFT | ElutePCFTvsEluteGR | Wash01PCFTvsWash01GR | Wash02PCFTvsWash02GR | EluteGRvsWash01GRbins | EluteGRvsWash02GRbins | ElutePCFTvsWash01PCFTbins | ElutePCFTvsWash02PCFTbins |
|---|---|---|---|---|---|---|---|---|---|
| **1** | 5.63E-03 | 6.62E-03 | 1.22E+00 | 9.68E-01 | 1.14E+00 | (4.25e-05,0.00294] | (0,0.101] | (0.000255,0.0184] | (0,0.192] |
| **2** | 5.65E-03 | 6.64E-03 | NA | 9.69E-01 | 1.14E+00 | NA | NA | NA | NA |
| **3** | 2.83E-02 | 3.28E-02 | 3.01E+00 | 1.18E+00 | 1.37E+00 | (0.00294,0.0348] | (0.101,1.34] | (0.0184,Inf] | (0.192,21] |
| **4** | 2.56E-03 | 1.88E-03 | 3.89E-01 | 1.00E+00 | 7.34E-01 | (4.25e-05,0.00294] | (0,0.101] | (0,0.000255] | (0,0.192] |
| **5** | 3.46E-03 | 2.98E-03 | 1.37E-01 | 1.01E+00 | 8.70E-01 | (4.25e-05,0.00294] | (0,0.101] | (0,0.000255] | (0,0.192] |
| **6** | 1.47E-03 | 1.71E-03 | 5.35E-02 | 1.16E+00 | 1.35E+00 | (4.25e-05,0.00294] | (0.101,1.34] | (0,0.000255] | (0,0.192] |
| **7** | 5.50E-03 | 4.02E-03 | 1.21E+00 | 1.06E+00 | 7.73E-01 | (4.25e-05,0.00294] | (0.101,1.34] | (0.000255,0.0184] | (0.192,21] |
| **8** | 9.55E-03 | 1.27E-02 | 1.37E+00 | 1.12E+00 | 1.49E+00 | (4.25e-05,0.00294] | (0.101,1.34] | (0.000255,0.0184] | (0.192,21] |
| **9** | 1.06E-02 | 1.53E-02 | 2.73E-01 | 1.30E+00 | 1.86E+00 | (0.00294,0.0348] | (0.101,1.34] | (0.000255,0.0184] | (0,0.192] |
| **10** | 1.05E-03 | 1.48E-03 | 1.14E+00 | 9.08E-01 | 1.28E+00 | (4.25e-05,0.00294] | (0.101,1.34] | (0,0.000255] | (0,0.192] |
| **11** | 2.03E-03 | 2.47E-03 | 2.97E+00 | 8.98E-01 | 1.10E+00 | (4.25e-05,0.00294] | (0,0.101] | (0.000255,0.0184] | (0,0.192] |
| **12** | 8.55E-03 | 7.31E-03 | 1.28E+00 | 1.10E+00 | 9.36E-01 | (4.25e-05,0.00294] | (0,0.101] | (0.000255,0.0184] | (0,0.192] |
| **13** | 2.52E-03 | 2.45E-03 | 1.20E-01 | 9.99E-01 | 9.73E-01 | (4.25e-05,0.00294] | (0.101,1.34] | (0,0.000255] | (0,0.192] |
| **14** | 2.64E-03 | 3.19E-03 | 2.84E+00 | 9.77E-01 | 1.18E+00 | (4.25e-05,0.00294] | (0,0.101] | (0.000255,0.0184] | (0.192,21] |
| **15** | 3.43E-03 | 2.44E-03 | 3.79E-01 | 9.97E-01 | 7.10E-01 | (4.25e-05,0.00294] | (0,0.101] | (0,0.000255] | (0,0.192] |
| **16** | 3.64E-03 | 2.11E-03 | NA | 9.62E-01 | 5.57E-01 | NA | NA | NA | NA |
| **17** | 2.88E-03 | 3.35E-03 | 7.86E-01 | 1.00E+00 | 1.16E+00 | (4.25e-05,0.00294] | (0.101,1.34] | (0.000255,0.0184] | (0.192,21] |
| **18** | 6.34E-03 | 4.28E-03 | 1.65E-01 | 1.07E+00 | 7.23E-01 | (4.25e-05,0.00294] | (0.101,1.34] | (0,0.000255] | (0,0.192] |
| **19** | 2.58E-03 | 2.42E-03 | 1.20E-01 | 9.82E-01 | 9.19E-01 | (4.25e-05,0.00294] | (0.101,1.34] | (0,0.000255] | (0,0.192] |
| **20** | 1.08E-03 | 1.40E-03 | 3.39E-01 | 1.02E+00 | 1.32E+00 | (0,4.25e-05] | (0,0.101] | (0,0.000255] | (0,0.192] |
| **21** | 6.75E-04 | 6.51E-04 | 1.54E+00 | 1.11E+00 | 1.08E+00 | (0,4.25e-05] | (0,0.101] | (0,0.000255] | (0,0.192] |
| **22** | 1.21E-03 | 1.61E-03 | 2.81E-02 | 9.98E-01 | 1.33E+00 | (4.25e-05,0.00294] | (1.34,14.9] | (0,0.000255] | (0,0.192] |
| **23** | 6.39E-04 | 1.14E-03 | NA | 9.93E-01 | 1.77E+00 | NA | NA | NA | NA |
| **24** | 2.37E-03 | 2.94E-03 | Inf | 1.15E+00 | 1.42E+00 | NA | NA | (0,0.000255] | (0,0.192] |
| **25** | 2.27E-03 | 2.43E-03 | 1.95E-01 | 1.15E+00 | 1.23E+00 | (4.25e-05,0.00294] | (0,0.101] | (0,0.000255] | (0,0.192] |
| **26** | 2.07E-02 | 7.58E-03 | NA | 2.08E+00 | 7.60E-01 | NA | NA | NA | NA |
| **27** | 1.32E-03 | 1.59E-03 | 1.73E-01 | 1.09E+00 | 1.30E+00 | (4.25e-05,0.00294] | (0.101,1.34] | (0,0.000255] | (0,0.192] |
| **28** | 3.70E-03 | 3.65E-03 | NA | 9.96E-01 | 9.83E-01 | NA | NA | NA | NA |
| **29** | 3.92E-03 | 3.86E-03 | NA | 1.07E+00 | 1.06E+00 | NA | NA | NA | NA |
| **30** | 2.84E-03 | 2.71E-03 | 2.39E+00 | 9.38E-01 | 8.95E-01 | (0,4.25e-05] | (0,0.101] | (0,0.000255] | (0,0.192] |
| **31** | 1.73E-03 | 1.65E-03 | 6.27E-01 | 1.04E+00 | 9.91E-01 | (4.25e-05,0.00294] | (0.101,1.34] | (0,0.000255] | (0,0.192] |
| **32** | 4.45E-03 | 2.55E-03 | 8.84E-02 | 1.38E+00 | 7.89E-01 | (4.25e-05,0.00294] | (0.101,1.34] | (0,0.000255] | (0,0.192] |
| **33** | 4.20E-03 | 2.80E-03 | NA | 1.08E+00 | 7.17E-01 | NA | NA | NA | NA |
| **34** | 6.17E-02 | 6.06E-02 | 3.26E-01 | 1.21E+00 | 1.19E+00 | (0.00294,0.0348] | (0.101,1.34] | (0.000255,0.0184] | (0,0.192] |

| | | | | | | | | | | |
|---|---|---|---|---|---|---|---|---|---|---|
| **35** | 3.00E-03 | 3.95E-03 | 1.38E+00 | 1.07E+00 | 1.41E+00 | | (0,4.25e-05] | (0,0.101] | (0,0.000255] | (0,0.192] |
| **36** | 3.59E-03 | 4.53E-03 | 4.26E-01 | 9.60E-01 | 1.21E+00 | | (4.25e-05,0.00294] | (0.101,1.34] | (0.000255,0.0184] | (0,0.192] |
| **37** | 3.07E-03 | 2.14E-03 | 3.75E-01 | 1.06E+00 | 7.41E-01 | | (4.25e-05,0.00294] | (0.101,1.34] | (0.000255,0.0184] | (0,0.192] |
| **38** | 2.45E-03 | 3.65E-03 | 4.04E+00 | 1.02E+00 | 1.52E+00 | | (0,4.25e-05] | (0,0.101] | (0,0.000255] | (0,0.192] |
| **39** | 1.68E-03 | 1.90E-03 | NA | 1.14E+00 | 1.28E+00 | | NA | NA | NA | NA |
| **40** | 3.65E-03 | 2.22E-03 | 1.84E+00 | 9.81E-01 | 5.96E-01 | | (4.25e-05,0.00294] | (0,0.101] | (0.000255,0.0184] | (0.192,21] |
| **41** | 1.28E-03 | 1.54E-03 | NA | 9.73E-01 | 1.16E+00 | | NA | NA | NA | NA |
| **42** | 1.14E-03 | 1.35E-03 | 0.00E+00 | 1.07E+00 | 1.26E+00 | | (4.25e-05,0.00294] | (0.101,1.34] | NA | NA |
| **43** | 3.44E-03 | 4.57E-03 | NA | 1.07E+00 | 1.42E+00 | | NA | NA | NA | NA |
| **44** | 1.87E-03 | 2.44E-03 | NA | 1.11E+00 | 1.45E+00 | | NA | NA | NA | NA |
| **45** | 2.58E-02 | 2.40E-02 | 5.25E-01 | 1.16E+00 | 1.08E+00 | | (0.00294,0.0348] | (0.101,1.34] | (0.000255,0.0184] | (0,0.192] |
| **46** | 3.52E-03 | 4.99E-03 | 9.79E+00 | 1.04E+00 | 1.48E+00 | | (0,4.25e-05] | (0,0.101] | (0,0.000255] | (0,0.192] |
| **47** | 6.01E-04 | 7.35E-04 | NA | 1.09E+00 | 1.34E+00 | | NA | NA | NA | NA |
| **48** | 1.37E-03 | 1.32E-03 | 1.79E-01 | 1.06E+00 | 1.02E+00 | | (4.25e-05,0.00294] | (0.101,1.34] | (0,0.000255] | (0,0.192] |
| **49** | 1.15E-03 | 1.32E-03 | 4.60E-01 | 1.09E+00 | 1.25E+00 | | (4.25e-05,0.00294] | (0.101,1.34] | (0,0.000255] | (0,0.192] |
| **50** | 1.24E-02 | 1.22E-02 | 1.59E+00 | 1.20E+00 | 1.18E+00 | | (4.25e-05,0.00294] | (0,0.101] | (0.000255,0.0184] | (0,0.192] |
| **51** | 4.15E-03 | 3.57E-03 | NA | 1.04E+00 | 8.91E-01 | | NA | NA | NA | NA |
| **52** | 3.57E-03 | 1.51E-03 | 3.93E-01 | 1.16E+00 | 4.90E-01 | | (4.25e-05,0.00294] | (0.101,1.34] | (0,0.000255] | (0,0.192] |
| **53** | 2.39E-03 | 2.83E-03 | NA | 1.02E+00 | 1.21E+00 | | NA | NA | NA | NA |
| **54** | 1.67E-03 | 1.40E-03 | NA | 9.52E-01 | 8.01E-01 | | NA | NA | NA | NA |
| **55** | 7.12E-04 | 1.69E-03 | NA | 9.02E-01 | 2.14E+00 | | NA | NA | NA | NA |
| **56** | 6.40E-03 | 3.61E-03 | NA | 1.17E+00 | 6.61E-01 | | NA | NA | NA | NA |
| **57** | 5.59E-03 | 4.67E-03 | 4.33E-02 | 1.24E+00 | 1.04E+00 | | (4.25e-05,0.00294] | (0.101,1.34] | (0,0.000255] | (0,0.192] |
| **58** | 5.59E-03 | 4.67E-03 | 4.33E-02 | 1.24E+00 | 1.04E+00 | | (4.25e-05,0.00294] | (0.101,1.34] | (0,0.000255] | (0,0.192] |
| **59** | 4.43E-03 | 5.16E-03 | NA | 1.28E+00 | 1.49E+00 | | NA | NA | NA | NA |
| **60** | 4.36E-04 | 2.39E-03 | 3.09E-01 | 1.04E+00 | 5.72E+00 | | (0.00294,0.0348] | (14.9,Inf] | (0.000255,0.0184] | (0.192,21] |
| **61** | 3.22E-03 | 3.17E-03 | NA | 1.04E+00 | 1.02E+00 | | NA | NA | NA | NA |
| **62** | 3.20E-03 | 5.58E-03 | NA | 1.04E+00 | 1.82E+00 | | NA | NA | NA | NA |
| **63** | 2.90E-03 | 4.30E-03 | NA | 9.71E-01 | 1.44E+00 | | NA | NA | NA | NA |
| **64** | 5.41E-03 | 5.55E-03 | NA | 8.84E-01 | 9.07E-01 | | NA | NA | NA | NA |
| **65** | 3.26E-03 | 2.09E-03 | 2.62E+00 | 1.03E+00 | 6.61E-01 | | (0.00294,0.0348] | (1.34,14.9] | (0.000255,0.0184] | (0.192,21] |
| **66** | 1.66E-03 | 1.73E-02 | 4.26E+00 | 8.92E-01 | 9.27E+00 | | (0.0348,Inf] | (14.9,Inf] | (0.0184,Inf] | (21,Inf] |
| **67** | 1.75E-03 | 1.60E-03 | Inf | 1.07E+00 | 9.76E-01 | | NA | NA | (0,0.000255] | (0,0.192] |
| **68** | 3.30E-03 | 2.90E-03 | 1.25E-01 | 1.03E+00 | 9.03E-01 | | (4.25e-05,0.00294] | (0.101,1.34] | (0,0.000255] | (0,0.192] |
| **69** | NA | NA | NA | 1.21E+00 | NA | | NA | NA | NA | NA |
| **70** | 8.09E-03 | 8.82E-03 | 2.34E-01 | 8.61E-01 | 9.38E-01 | | (0.0348,Inf] | (1.34,14.9] | (0.000255,0.0184] | (0.192,21] |
| **71** | 1.59E-03 | 2.60E-03 | Inf | 9.88E-01 | 1.61E+00 | | NA | NA | (0,0.000255] | (0,0.192] |
| **72** | 3.78E-03 | 4.08E-03 | Inf | 1.25E+00 | 1.35E+00 | | NA | NA | (0,0.000255] | (0,0.192] |

| | | | | | | | | | |
|---|---|---|---|---|---|---|---|---|---|
| **73** | 3.30E-03 | 4.88E-03 | NA | 9.27E-01 | 1.37E+00 | NA | NA | NA | NA |
| **74** | 1.33E-03 | 3.69E-03 | 2.13E+01 | 9.22E-01 | 2.55E+00 | (0,4.25e-05] | (0,0.101] | (0.000255,0.0184] | (0.192,21] |
| **75** | 2.61E-03 | 4.71E-03 | 0.00E+00 | 1.05E+00 | 1.89E+00 | (0,4.25e-05] | (0,0.101] | NA | NA |
| **76** | 2.66E-03 | 3.48E-03 | NA | 9.53E-01 | 1.25E+00 | NA | NA | NA | NA |
| **77** | 1.94E-03 | 2.13E-03 | NA | 1.11E+00 | 1.22E+00 | NA | NA | NA | NA |
| **78** | 1.45E-03 | 1.53E-03 | 4.74E-01 | 1.07E+00 | 1.14E+00 | (4.25e-05,0.00294] | (0.101,1.34] | (0.000255,0.0184] | (0,0.192] |
| **79** | 3.57E-03 | 3.92E-03 | Inf | 1.07E+00 | 1.18E+00 | NA | NA | (0,0.000255] | (0,0.192] |
| **80** | 2.47E-03 | 3.10E-03 | 1.09E+00 | 1.09E+00 | 1.36E+00 | (4.25e-05,0.00294] | (0.101,1.34] | (0.000255,0.0184] | (0,0.192] |
| **81** | 3.97E-03 | 1.73E-02 | 4.41E-01 | 1.01E+00 | 4.38E+00 | (0.0348,Inf] | (14.9,Inf] | (0.0184,Inf] | (0.192,21] |
| **82** | 1.85E-03 | 2.45E-03 | 6.49E-01 | 1.12E+00 | 1.48E+00 | (0.00294,0.0348] | (1.34,14.9] | (0.000255,0.0184] | (0.192,21] |
| **83** | 4.47E-03 | 5.12E-03 | Inf | 1.01E+00 | 1.16E+00 | NA | NA | (0.000255,0.0184] | (0,0.192] |
| **84** | 2.79E-03 | 2.50E-03 | 6.21E-01 | 1.11E+00 | 9.97E-01 | (4.25e-05,0.00294] | (0.101,1.34] | (0.000255,0.0184] | (0,0.192] |
| **85** | 4.03E-03 | 5.86E-03 | NA | 1.11E+00 | 1.61E+00 | NA | NA | NA | NA |
| **86** | 3.30E-03 | 3.41E-03 | NA | 1.13E+00 | 1.17E+00 | NA | NA | NA | NA |
| **87** | 2.26E-03 | 2.03E-03 | NA | 1.20E+00 | 1.08E+00 | NA | NA | NA | NA |
| **88** | 2.21E-03 | 2.23E-03 | 9.64E-01 | 1.30E+00 | 1.31E+00 | (4.25e-05,0.00294] | (0.101,1.34] | (0.000255,0.0184] | (0.192,21] |
| **89** | 8.78E-04 | 1.07E-03 | 0.00E+00 | 9.84E-01 | 1.20E+00 | (4.25e-05,0.00294] | (0.101,1.34] | NA | NA |
| **90** | 3.40E-03 | 5.89E-03 | Inf | 8.54E-01 | 1.48E+00 | NA | NA | (0.000255,0.0184] | (0.192,21] |
| **91** | 1.03E-03 | 8.25E-04 | NA | 9.88E-01 | 7.90E-01 | NA | NA | NA | NA |
| **92** | 1.76E-03 | 9.79E-04 | NA | 9.16E-01 | 5.11E-01 | NA | NA | NA | NA |
| **93** | 4.46E-03 | 2.07E-03 | NA | 9.28E-01 | 4.32E-01 | NA | NA | NA | NA |
| **94** | 6.31E-03 | 6.25E-03 | 3.32E-01 | 1.04E+00 | 1.03E+00 | (4.25e-05,0.00294] | (0.101,1.34] | (0.000255,0.0184] | (0,0.192] |
| **95** | 6.03E-04 | 5.49E-04 | NA | 1.09E+00 | 9.93E-01 | NA | NA | NA | NA |
| **96** | 1.09E-03 | 2.05E-03 | NA | 8.80E-01 | 1.65E+00 | NA | NA | NA | NA |
| **97** | 2.22E-03 | 3.88E-03 | NA | 9.88E-01 | 1.73E+00 | NA | NA | NA | NA |
| **98** | 1.96E-03 | 1.44E-03 | NA | 9.88E-01 | 7.24E-01 | NA | NA | NA | NA |
| **99** | 2.46E-03 | 2.23E-03 | Inf | 9.96E-01 | 9.06E-01 | NA | NA | (0,0.000255] | (0,0.192] |
| **100** | 1.22E-03 | 1.17E-03 | NA | 9.06E-01 | 8.70E-01 | NA | NA | NA | NA |
| **101** | 2.54E-03 | 1.58E-03 | NA | 9.32E-01 | 5.80E-01 | NA | NA | NA | NA |
| **102** | 2.47E-03 | 9.47E-04 | 1.83E-01 | 1.08E+00 | 4.13E-01 | (4.25e-05,0.00294] | (0.101,1.34] | (0.000255,0.0184] | (0.192,21] |
| **103** | 4.02E-03 | 1.75E-03 | NA | 8.93E-01 | 3.89E-01 | NA | NA | NA | NA |
| **104** | 1.09E-03 | 1.93E-03 | NA | 9.84E-01 | 1.75E+00 | NA | NA | NA | NA |
| **105** | 2.35E-03 | 1.98E-03 | NA | 9.58E-01 | 8.07E-01 | NA | NA | NA | NA |
| **106** | 4.68E-03 | 2.68E-03 | 0.00E+00 | 1.29E+00 | 7.36E-01 | (0.00294,0.0348] | (1.34,14.9] | NA | NA |
| **107** | 8.21E-04 | 6.77E-04 | 0.00E+00 | 6.93E-01 | 5.71E-01 | (0,4.25e-05] | (0,0.101] | NA | NA |
| **108** | 3.19E-03 | 3.12E-03 | 5.80E-01 | 8.91E-01 | 8.72E-01 | (4.25e-05,0.00294] | (0.101,1.34] | (0,0.000255] | (0,0.192] |
| **109** | 4.15E-03 | 9.00E-03 | NA | 8.73E-01 | 1.89E+00 | NA | NA | NA | NA |
| **110** | 1.65E-03 | 2.07E-03 | NA | 1.05E+00 | 1.32E+00 | NA | NA | NA | NA |

| | | | | | | | | | |
|---|---|---|---|---|---|---|---|---|---|
| **111** | 3.86E-03 | 1.06E-03 | NA | 1.13E+00 | 3.09E-01 | NA | NA | NA | NA |
| **112** | 3.10E-03 | 1.23E-03 | 5.64E+00 | 9.87E-01 | 3.91E-01 | (4.25e-05,0.00294] | (0.101,1.34] | (0.000255,0.0184] | (0.192,21] |
| **113** | 3.85E-03 | 3.41E-03 | NA | 1.34E+00 | 1.19E+00 | NA | NA | NA | NA |
| **114** | 1.86E-03 | 2.06E-03 | 0.00E+00 | 9.74E-01 | 1.08E+00 | (4.25e-05,0.00294] | (0,0.101] | NA | NA |
| **115** | 1.53E-03 | 2.31E-03 | NA | 9.91E-01 | 1.50E+00 | NA | NA | NA | NA |
| **116** | 1.99E-03 | 2.65E-03 | NA | 9.85E-01 | 1.31E+00 | NA | NA | NA | NA |
| **117** | 7.00E-04 | 1.54E-03 | 0.00E+00 | 1.01E+00 | 2.24E+00 | (0.00294,0.0348] | (1.34,14.9] | NA | NA |
| **118** | 3.93E-03 | 3.89E-03 | 7.84E-01 | 9.68E-01 | 9.60E-01 | (0.00294,0.0348] | (0.101,1.34] | (0.000255,0.0184] | (0.192,21] |
| **119** | 1.51E-03 | 1.15E-03 | 0.00E+00 | 9.69E-01 | 7.37E-01 | (4.25e-05,0.00294] | (0,0.101] | NA | NA |
| **120** | 2.43E-03 | 2.25E-03 | 0.00E+00 | 1.02E+00 | 9.48E-01 | (4.25e-05,0.00294] | (0,0.101] | NA | NA |
| **121** | 7.35E-03 | 2.82E-03 | NA | 1.03E+00 | 3.96E-01 | NA | NA | NA | NA |
| **122** | 5.08E-03 | 6.53E-03 | Inf | 1.23E+00 | 1.58E+00 | NA | NA | (0,0.000255] | (0,0.192] |
| **123** | 4.63E-03 | 5.82E-03 | NA | 1.03E+00 | 1.29E+00 | NA | NA | NA | NA |
| **124** | 2.10E-03 | 2.62E-03 | 0.00E+00 | 8.39E-01 | 1.05E+00 | (4.25e-05,0.00294] | (0.101,1.34] | NA | NA |
| **125** | 2.32E-03 | 1.44E-03 | 2.77E+02 | 8.63E-01 | 5.36E-01 | (0,4.25e-05] | (0,0.101] | (0.000255,0.0184] | (0.192,21] |
| **126** | 2.28E-03 | 2.13E-03 | 0.00E+00 | 8.93E-01 | 8.35E-01 | (4.25e-05,0.00294] | (0,0.101] | NA | NA |
| **127** | 3.38E-03 | 2.88E-03 | 0.00E+00 | 9.79E-01 | 8.34E-01 | (4.25e-05,0.00294] | (0.101,1.34] | NA | NA |
| **128** | 2.35E-03 | 1.58E-03 | 2.30E-02 | 1.11E+00 | 7.51E-01 | (0.00294,0.0348] | (0.101,1.34] | (0,0.000255] | (0,0.192] |
| **129** | 2.35E-03 | 1.58E-03 | 2.30E-02 | 1.11E+00 | 7.51E-01 | (0.00294,0.0348] | (0.101,1.34] | (0,0.000255] | (0,0.192] |
| **130** | 1.35E-03 | 1.79E-03 | NA | 9.92E-01 | 1.32E+00 | NA | NA | NA | NA |
| **131** | 4.27E-03 | 3.26E-03 | NA | 1.21E+00 | 9.24E-01 | NA | NA | NA | NA |
| **132** | 3.18E-03 | 3.15E-03 | NA | 9.69E-01 | 9.61E-01 | NA | NA | NA | NA |
| **133** | 3.64E-03 | 5.07E-03 | NA | 9.33E-01 | 1.30E+00 | NA | NA | NA | NA |
| **134** | 2.40E-03 | 2.33E-03 | NA | 1.01E+00 | 9.82E-01 | NA | NA | NA | NA |
| **135** | 1.38E-03 | 8.62E-04 | NA | 1.31E+00 | 8.19E-01 | NA | NA | NA | NA |
| **136** | 2.53E-03 | 2.20E-03 | NA | 7.93E-01 | 6.90E-01 | NA | NA | NA | NA |
| **137** | 2.05E-03 | 3.16E-03 | NA | 1.10E+00 | 1.70E+00 | NA | NA | NA | NA |
| **138** | 7.28E-03 | 4.19E-03 | NA | 9.85E-01 | 5.67E-01 | NA | NA | NA | NA |
| **139** | 4.71E-03 | 4.99E-03 | NA | 1.10E+00 | 1.17E+00 | NA | NA | NA | NA |
| **140** | 3.11E-03 | 3.90E-03 | Inf | 1.00E+00 | 1.26E+00 | NA | NA | (0,0.000255] | (0,0.192] |
| **141** | 1.70E-03 | 2.27E-03 | NA | 9.16E-01 | 1.23E+00 | NA | NA | NA | NA |
| **142** | 5.13E-03 | 4.22E-03 | Inf | 1.27E+00 | 1.05E+00 | NA | NA | (0.000255,0.0184] | (0.192,21] |
| **143** | 4.35E-03 | 8.84E-03 | NA | 9.89E-01 | 2.01E+00 | NA | NA | NA | NA |
| **144** | 2.69E-03 | 1.52E-03 | NA | 1.05E+00 | 5.94E-01 | NA | NA | NA | NA |
| **145** | 6.65E-03 | 3.48E-03 | NA | 8.73E-01 | 4.57E-01 | NA | NA | NA | NA |
| **146** | 1.23E-03 | 1.36E-03 | NA | 8.91E-01 | 9.86E-01 | NA | NA | NA | NA |
| **147** | 2.93E-03 | 2.22E-03 | Inf | 1.13E+00 | 8.55E-01 | NA | NA | (0.000255,0.0184] | (0.192,21] |
| **148** | 8.63E-04 | 1.41E-03 | 0.00E+00 | 9.41E-01 | 1.54E+00 | (4.25e-05,0.00294] | (1.34,14.9] | NA | NA |

| | | | | | | | | | |
|---|---|---|---|---|---|---|---|---|---|
| **149** | 2.01E-03 | 1.58E-03 | 3.56E-02 | 9.95E-01 | 7.81E-01 | (0.00294,0.0348] | (1.34,14.9] | (0,0.000255] | (0,0.192] |
| **150** | 2.89E-03 | 2.30E-03 | 0.00E+00 | 1.04E+00 | 8.30E-01 | (4.25e-05,0.00294] | (0.101,1.34] | NA | NA |
| **151** | 2.74E-03 | 2.25E-03 | 1.54E-01 | 9.81E-01 | 8.03E-01 | (0.00294,0.0348] | (1.34,14.9] | (0.000255,0.0184] | (0.192,21] |
| **152** | 4.02E-03 | 1.69E-03 | NA | 9.58E-01 | 4.03E-01 | NA | NA | NA | NA |
| **153** | 3.96E-03 | 2.51E-03 | 6.51E-02 | 1.06E+00 | 6.70E-01 | (4.25e-05,0.00294] | (0,0.101] | (0,0.000255] | (0,0.192] |
| **154** | 1.51E-03 | 1.50E-03 | 3.83E-02 | 9.53E-01 | 9.50E-01 | (4.25e-05,0.00294] | (0.101,1.34] | (0,0.000255] | (0,0.192] |
| **155** | 1.87E-03 | 1.07E-03 | NA | 8.37E-01 | 4.80E-01 | NA | NA | NA | NA |
| **156** | 3.94E-03 | 5.28E-03 | 0.00E+00 | 1.00E+00 | 1.34E+00 | (0.00294,0.0348] | (1.34,14.9] | NA | NA |
| **157** | 3.15E-03 | 2.37E-03 | Inf | 1.13E+00 | 8.47E-01 | NA | NA | (0.000255,0.0184] | (0.192,21] |
| **158** | 7.10E-03 | 9.16E-03 | 0.00E+00 | 1.04E+00 | 1.35E+00 | (4.25e-05,0.00294] | (0.101,1.34] | NA | NA |
| **159** | 1.68E-03 | 3.13E-03 | 3.16E-01 | 9.59E-01 | 1.78E+00 | (4.25e-05,0.00294] | (1.34,14.9] | (0.000255,0.0184] | (0.192,21] |
| **160** | 8.06E-03 | 5.02E-03 | Inf | 9.18E-01 | 5.72E-01 | NA | NA | (0.000255,0.0184] | (0,0.192] |
| **161** | 9.88E-04 | 1.55E-03 | NA | 1.03E+00 | 1.62E+00 | NA | NA | NA | NA |
| **162** | 5.99E-03 | 4.22E-03 | NA | 1.29E+00 | 9.12E-01 | NA | NA | NA | NA |
| **163** | 3.35E-03 | 4.11E-03 | NA | 8.89E-01 | 1.09E+00 | NA | NA | NA | NA |
| **164** | 1.79E-03 | 2.22E-03 | NA | 8.30E-01 | 1.03E+00 | NA | NA | NA | NA |
| **165** | 1.11E-03 | 1.73E-03 | NA | 9.44E-01 | 1.47E+00 | NA | NA | NA | NA |
| **166** | 3.99E-03 | 1.50E-03 | NA | 1.26E+00 | 4.76E-01 | NA | NA | NA | NA |
| **167** | 1.65E-03 | 1.45E-03 | 0.00E+00 | 9.81E-01 | 8.61E-01 | (4.25e-05,0.00294] | (0.101,1.34] | NA | NA |
| **168** | 8.70E-04 | 6.84E-03 | NA | 6.88E-01 | 5.41E+00 | NA | NA | NA | NA |
| **169** | 7.40E-03 | 8.19E-03 | Inf | 1.07E+00 | 1.18E+00 | NA | NA | (0.000255,0.0184] | (0,0.192] |
| **170** | 4.11E-03 | 5.05E-03 | NA | 9.59E-01 | 1.18E+00 | NA | NA | NA | NA |
| **171** | 6.86E-04 | 6.14E-04 | Inf | 1.30E+00 | 1.17E+00 | NA | NA | (0,0.000255] | (0,0.192] |
| **172** | 2.86E-03 | 2.15E-03 | 0.00E+00 | 1.22E+00 | 9.21E-01 | (4.25e-05,0.00294] | (0.101,1.34] | NA | NA |
| **173** | 3.40E-03 | 2.89E-03 | NA | 9.51E-01 | 8.07E-01 | NA | NA | NA | NA |
| **174** | 3.14E-03 | 4.10E-03 | NA | 8.17E-01 | 1.07E+00 | NA | NA | NA | NA |
| **175** | 2.02E-03 | 1.45E-03 | NA | 8.76E-01 | 6.29E-01 | NA | NA | NA | NA |
| **176** | 1.02E-02 | 6.09E-03 | NA | 9.85E-01 | 5.90E-01 | NA | NA | NA | NA |
| **177** | 5.35E-03 | 4.13E-03 | NA | 8.90E-01 | 6.87E-01 | NA | NA | NA | NA |
| **178** | 1.24E-02 | 1.57E-02 | NA | 8.98E-01 | 1.14E+00 | NA | NA | NA | NA |
| **179** | 6.43E-03 | 2.32E-03 | NA | 1.06E+00 | 3.85E-01 | NA | NA | NA | NA |
| **180** | 2.92E-03 | 2.07E-03 | NA | 1.28E+00 | 9.09E-01 | NA | NA | NA | NA |
| **181** | 1.85E-03 | 2.78E-03 | 2.52E-02 | 9.92E-01 | 1.49E+00 | (0.00294,0.0348] | (1.34,14.9] | (0,0.000255] | (0,0.192] |
| **182** | 4.10E-03 | 2.83E-03 | Inf | 8.53E-01 | 5.88E-01 | NA | NA | (0.000255,0.0184] | (0.192,21] |
| **183** | 1.76E-03 | 2.14E-03 | NA | 9.16E-01 | 1.11E+00 | NA | NA | NA | NA |
| **184** | 4.52E-03 | 3.15E-03 | NA | 1.23E+00 | 8.58E-01 | NA | NA | NA | NA |
| **185** | 3.04E-03 | 2.83E-03 | NA | 8.97E-01 | 8.35E-01 | NA | NA | NA | NA |
| **186** | 1.74E-03 | 6.86E-03 | NA | 8.68E-01 | 3.43E+00 | NA | NA | NA | NA |

| | | | | | | | | | |
|---|---|---|---|---|---|---|---|---|---|
| **187** | 8.51E-04 | 1.07E-03 | NA | 1.02E+00 | 1.28E+00 | NA | NA | NA | NA |
| **188** | 3.63E-03 | 4.01E-03 | NA | 8.84E-01 | 9.77E-01 | NA | NA | NA | NA |
| **189** | 3.84E-03 | 4.68E-03 | NA | 1.21E+00 | 1.48E+00 | NA | NA | NA | NA |
| **190** | 2.74E-03 | 2.59E-03 | 0.00E+00 | 9.70E-01 | 9.18E-01 | (4.25e-05,0.00294] | (0,0.101] | NA | NA |
| **191** | 6.66E-03 | 4.82E-03 | NA | 1.38E+00 | 9.98E-01 | NA | NA | NA | NA |
| **192** | 3.75E-03 | 3.05E-03 | NA | 1.13E+00 | 9.22E-01 | NA | NA | NA | NA |
| **193** | 1.95E-03 | 1.32E-03 | 0.00E+00 | 9.29E-01 | 6.30E-01 | (0.00294,0.0348] | (1.34,14.9] | NA | NA |
| **194** | 9.76E-03 | 5.84E-03 | NA | 9.38E-01 | 5.61E-01 | NA | NA | NA | NA |
| **195** | 5.39E-03 | 5.22E-03 | 0.00E+00 | 8.30E-01 | 8.03E-01 | (4.25e-05,0.00294] | (0.101,1.34] | NA | NA |
| **196** | 1.76E-03 | 6.47E-04 | NA | 1.05E+00 | 3.84E-01 | NA | NA | NA | NA |
| **197** | 3.26E-03 | 4.09E-03 | NA | 1.03E+00 | 1.29E+00 | NA | NA | NA | NA |
| **198** | 1.83E-03 | 2.35E-03 | NA | 9.42E-01 | 1.20E+00 | NA | NA | NA | NA |
| **199** | 4.52E-03 | 2.81E-03 | 1.58E+00 | 9.72E-01 | 6.05E-01 | (0.00294,0.0348] | (0.101,1.34] | (0.000255,0.0184] | (0.192,21] |
| **200** | 9.34E-03 | 7.38E-03 | NA | 9.74E-01 | 7.70E-01 | NA | NA | NA | NA |
| **201** | 2.71E-03 | 6.95E-03 | NA | 1.04E+00 | 2.67E+00 | NA | NA | NA | NA |
| **202** | 4.27E-03 | 1.96E-03 | NA | 8.50E-01 | 3.90E-01 | NA | NA | NA | NA |
| **203** | 3.08E-03 | 5.53E-03 | NA | 8.29E-01 | 1.48E+00 | NA | NA | NA | NA |
| **204** | 2.26E-03 | 4.54E-03 | 0.00E+00 | 7.88E-01 | 1.58E+00 | (4.25e-05,0.00294] | (0.101,1.34] | NA | NA |
| **205** | 4.61E-03 | 2.64E-03 | NA | 1.31E+00 | 7.50E-01 | NA | NA | NA | NA |
| **206** | 3.34E-03 | 3.48E-03 | 0.00E+00 | 1.05E+00 | 1.09E+00 | (4.25e-05,0.00294] | (0.101,1.34] | NA | NA |
| **207** | 3.05E-03 | 3.69E-03 | NA | 1.14E+00 | 1.38E+00 | NA | NA | NA | NA |
| **208** | 1.65E-03 | 1.77E-03 | Inf | 9.05E-01 | 9.70E-01 | NA | NA | (0.000255,0.0184] | (0.192,21] |
| **209** | 2.33E-03 | 1.97E-03 | NA | 9.31E-01 | 7.88E-01 | NA | NA | NA | NA |
| **210** | 4.99E-03 | 3.26E-03 | NA | 7.90E-01 | 5.17E-01 | NA | NA | NA | NA |
| **211** | 1.22E-02 | 6.67E-03 | NA | 2.00E+00 | 1.09E+00 | NA | NA | NA | NA |
| **212** | 1.64E-03 | 1.34E-03 | NA | 1.07E+00 | 8.73E-01 | NA | NA | NA | NA |
| **213** | NA | NA | NA | 8.15E-01 | NA | NA | NA | NA | NA |
| **214** | 7.80E-03 | 6.58E-03 | NA | 1.24E+00 | 1.04E+00 | NA | NA | NA | NA |
| **215** | 2.39E-03 | 2.07E-03 | NA | 8.94E-01 | 7.76E-01 | NA | NA | NA | NA |
| **216** | 4.10E-03 | 5.37E-03 | 5.69E-01 | 8.16E-01 | 1.07E+00 | (4.25e-05,0.00294] | (0.101,1.34] | (0.000255,0.0184] | (0,0.192] |
| **217** | 1.34E-03 | 1.11E-03 | NA | 9.38E-01 | 7.80E-01 | NA | NA | NA | NA |
| **218** | 5.33E-03 | 6.15E-03 | 4.45E-01 | 1.07E+00 | 1.23E+00 | (4.25e-05,0.00294] | (0.101,1.34] | (0.000255,0.0184] | (0.192,21] |
| **219** | 5.58E-03 | 3.70E-03 | NA | 9.41E-01 | 6.24E-01 | NA | NA | NA | NA |
| **220** | 2.53E-03 | 2.29E-03 | 0.00E+00 | 1.07E+00 | 9.67E-01 | (4.25e-05,0.00294] | (0,0.101] | NA | NA |
| **221** | 6.71E-03 | 4.68E-03 | NA | 1.36E+00 | 9.46E-01 | NA | NA | NA | NA |
| **222** | 3.14E-03 | 1.26E-03 | Inf | 9.89E-01 | 3.97E-01 | NA | NA | (0.000255,0.0184] | (0.192,21] |
| **223** | 1.42E-03 | 1.65E-03 | NA | 9.42E-01 | 1.10E+00 | NA | NA | NA | NA |
| **224** | 1.64E-03 | 2.58E-03 | NA | 9.93E-01 | 1.56E+00 | NA | NA | NA | NA |

| | | | | | | | | | |
|---|---|---|---|---|---|---|---|---|---|
| **225** | 3.14E-03 | 1.01E-03 | NA | 9.37E-01 | 3.03E-01 | NA | NA | NA | NA |
| **226** | 1.63E-03 | 2.46E-03 | NA | 8.26E-01 | 1.25E+00 | NA | NA | NA | NA |
| **227** | 2.86E-03 | 2.41E-02 | Inf | 1.06E+00 | 8.91E+00 | NA | NA | (0.000255,0.0184] | (0,0.192] |
| **228** | 5.30E-03 | 2.39E-03 | 0.00E+00 | 9.86E-01 | 4.44E-01 | (4.25e-05,0.00294] | (0.101,1.34] | NA | NA |
| **229** | 1.74E-03 | 1.68E-03 | NA | 8.97E-01 | 8.69E-01 | NA | NA | NA | NA |
| **230** | NA | NA | NA | NA | 1.11E+00 | NA | NA | NA | NA |
| **231** | 2.16E-03 | 7.68E-04 | 2.49E+00 | 8.85E-01 | 3.16E-01 | (4.25e-05,0.00294] | (0,0.101] | (0,0.000255] | (0.192,21] |
| **232** | 1.09E-03 | 1.76E-03 | NA | 9.44E-01 | 1.52E+00 | NA | NA | NA | NA |
| **233** | 3.75E-03 | 1.35E-03 | NA | 8.65E-01 | 3.12E-01 | NA | NA | NA | NA |
| **234** | 1.42E-03 | 2.67E-03 | NA | 9.47E-01 | 1.78E+00 | NA | NA | NA | NA |
| **235** | 2.48E-03 | 5.65E-03 | NA | 7.43E-01 | 1.69E+00 | NA | NA | NA | NA |
| **236** | 1.23E-02 | 6.92E-03 | NA | 9.71E-01 | 5.45E-01 | NA | NA | NA | NA |
| **237** | 2.33E-03 | 4.28E-03 | NA | 8.18E-01 | 1.50E+00 | NA | NA | NA | NA |
| **238** | 3.90E-03 | 2.27E-03 | NA | 1.00E+00 | 5.82E-01 | NA | NA | NA | NA |
| **239** | 3.64E-03 | 3.72E-03 | NA | 1.11E+00 | 1.14E+00 | NA | NA | NA | NA |
| **240** | 4.31E-03 | 1.72E-03 | NA | 1.25E+00 | 4.96E-01 | NA | NA | NA | NA |
| **241** | 1.27E-02 | 7.08E-03 | NA | 9.69E-01 | 5.40E-01 | NA | NA | NA | NA |
| **242** | 1.34E-03 | 2.43E-03 | 0.00E+00 | 8.28E-01 | 1.51E+00 | (4.25e-05,0.00294] | (0.101,1.34] | NA | NA |
| **243** | 3.11E-03 | 6.55E-03 | NA | 7.61E-01 | 1.60E+00 | NA | NA | NA | NA |
| **244** | 8.31E-03 | 3.67E-03 | 0.00E+00 | 1.03E+00 | 4.54E-01 | (4.25e-05,0.00294] | (0.101,1.34] | NA | NA |
| **245** | 2.89E-03 | 1.96E-03 | NA | 8.33E-01 | 5.66E-01 | NA | NA | NA | NA |
| **246** | 2.71E-03 | 1.28E-03 | 2.57E-02 | 9.28E-01 | 4.39E-01 | (4.25e-05,0.00294] | (0.101,1.34] | (0,0.000255] | (0,0.192] |
| **247** | 6.13E-03 | 6.10E-03 | 0.00E+00 | 1.03E+00 | 1.03E+00 | (0.00294,0.0348] | (1.34,14.9] | NA | NA |
| **248** | 1.33E-03 | 1.02E-03 | 0.00E+00 | 1.10E+00 | 8.41E-01 | (0.00294,0.0348] | (1.34,14.9] | NA | NA |
| **249** | 6.12E-03 | 3.17E-03 | NA | 1.02E+00 | 5.27E-01 | NA | NA | NA | NA |
| **250** | 2.43E-03 | 3.08E-03 | 0.00E+00 | 9.54E-01 | 1.21E+00 | (4.25e-05,0.00294] | (0.101,1.34] | NA | NA |
| **251** | 2.26E-03 | 2.13E-03 | 0.00E+00 | 1.10E+00 | 1.04E+00 | (0.00294,0.0348] | (1.34,14.9] | NA | NA |
| **252** | 1.25E-03 | 5.16E-04 | NA | 1.15E+00 | 4.76E-01 | NA | NA | NA | NA |
| **253** | 1.51E-03 | 1.05E-03 | NA | 1.17E+00 | 8.08E-01 | NA | NA | NA | NA |
| **254** | 2.15E-03 | 5.69E-03 | NA | 7.06E-01 | 1.87E+00 | NA | NA | NA | NA |
| **255** | 4.79E-03 | 6.73E-04 | NA | 1.10E+00 | 1.54E-01 | NA | NA | NA | NA |
| **256** | 4.79E-03 | 6.73E-04 | NA | 1.10E+00 | 1.54E-01 | NA | NA | NA | NA |
| **257** | 3.95E-03 | 5.97E-03 | Inf | 9.67E-01 | 1.46E+00 | NA | NA | (0.000255,0.0184] | (0,0.192] |
| **258** | 4.97E-03 | 3.14E-03 | NA | 9.76E-01 | 6.18E-01 | NA | NA | NA | NA |
| **259** | 1.74E-03 | 2.45E-03 | 5.57E-02 | 1.05E+00 | 1.48E+00 | (0.00294,0.0348] | (1.34,14.9] | (0.000255,0.0184] | (0.192,21] |
| **260** | 2.87E-03 | 3.57E-03 | Inf | 8.84E-01 | 1.10E+00 | NA | NA | (0.000255,0.0184] | (0.192,21] |
| **261** | 1.47E-03 | 8.95E-04 | 1.13E+00 | 8.38E-01 | 5.08E-01 | (4.25e-05,0.00294] | (0,0.101] | (0,0.000255] | (0,0.192] |
| **262** | 1.53E-03 | 1.99E-03 | NA | 9.50E-01 | 1.23E+00 | NA | NA | NA | NA |

| | | | | | | | | | | |
|---|---|---|---|---|---|---|---|---|---|---|
| **263** | 1.16E-03 | 1.27E-03 | Inf | 8.61E-01 | 9.47E-01 | NA | NA | (0.000255,0.0184] | (0.192,21] |
| **264** | 3.63E-03 | 1.82E-03 | NA | 9.45E-01 | 4.72E-01 | NA | NA | NA | NA |
| **265** | 1.64E-03 | 1.41E-03 | NA | 9.01E-01 | 7.73E-01 | NA | NA | NA | NA |
| **266** | 9.35E-04 | 1.49E-03 | 0.00E+00 | 9.68E-01 | 1.54E+00 | (4.25e-05,0.00294] | (0.101,1.34] | NA | NA |
| **267** | 2.67E-03 | 2.34E-03 | NA | 6.87E-01 | 6.03E-01 | NA | NA | NA | NA |
| **268** | 3.99E-03 | 3.31E-03 | NA | 1.03E+00 | 8.51E-01 | NA | NA | NA | NA |
| **269** | 2.03E-03 | 1.38E-03 | 0.00E+00 | 9.92E-01 | 6.76E-01 | (4.25e-05,0.00294] | (0.101,1.34] | NA | NA |
| **270** | 1.13E-03 | 1.33E-03 | NA | 9.95E-01 | 1.17E+00 | NA | NA | NA | NA |
| **271** | 1.52E-02 | 1.20E-02 | NA | 1.84E+00 | 1.45E+00 | NA | NA | NA | NA |
| **272** | 4.25E-03 | 1.29E-03 | NA | 1.23E+00 | 3.74E-01 | NA | NA | NA | NA |
| **273** | 7.00E-03 | 1.34E-03 | 0.00E+00 | 1.11E+00 | 2.13E-01 | (0,4.25e-05] | (0,0.101] | NA | NA |
| **274** | 7.22E-03 | 1.09E-02 | 6.92E-02 | 7.50E-01 | 1.13E+00 | (0.00294,0.0348] | (1.34,14.9] | (0.000255,0.0184] | (0,0.192] |
| **275** | 2.30E-03 | 1.85E-03 | NA | 9.23E-01 | 7.44E-01 | NA | NA | NA | NA |
| **276** | 4.53E-03 | 6.55E-03 | NA | 1.03E+00 | 1.48E+00 | NA | NA | NA | NA |
| **277** | 1.26E-03 | 2.24E-03 | NA | 8.85E-01 | 1.58E+00 | NA | NA | NA | NA |
| **278** | 2.63E-03 | 1.17E-03 | NA | 9.81E-01 | 4.38E-01 | NA | NA | NA | NA |
| **279** | 1.06E-03 | 1.44E-03 | NA | 9.30E-01 | 1.27E+00 | NA | NA | NA | NA |
| **280** | 1.27E-03 | 1.70E-03 | NA | 8.64E-01 | 1.15E+00 | NA | NA | NA | NA |
| **281** | 5.51E-03 | 3.51E-03 | 3.07E-02 | 1.05E+00 | 6.71E-01 | (4.25e-05,0.00294] | (0.101,1.34] | (0,0.000255] | (0,0.192] |
| **282** | 3.42E-03 | 4.22E-03 | NA | 1.01E+00 | 1.24E+00 | NA | NA | NA | NA |
| **283** | 2.75E-03 | 4.92E-03 | NA | 7.69E-01 | 1.37E+00 | NA | NA | NA | NA |
| **284** | 1.82E-03 | 1.34E-03 | NA | 9.14E-01 | 6.75E-01 | NA | NA | NA | NA |
| **285** | 5.70E-03 | 3.80E-03 | NA | 1.01E+00 | 6.71E-01 | NA | NA | NA | NA |
| **286** | 3.33E-05 | 0.00E+00 | Inf | 4.86E-01 | 0.00E+00 | NA | NA | (0.0184,Inf] | (21,Inf] |
| **287** | 1.35E-03 | 2.84E-03 | NA | 7.69E-01 | 1.62E+00 | NA | NA | NA | NA |
| **288** | 2.02E-03 | 1.52E-03 | NA | 8.86E-01 | 6.68E-01 | NA | NA | NA | NA |
| **289** | 5.94E-03 | 5.20E-03 | 0.00E+00 | 1.33E+00 | 1.16E+00 | (4.25e-05,0.00294] | (0,0.101] | NA | NA |
| **290** | 4.67E-03 | 2.29E-03 | NA | 1.08E+00 | 5.29E-01 | NA | NA | NA | NA |
| **291** | 9.54E-03 | 4.59E-03 | 1.70E-02 | 1.19E+00 | 5.74E-01 | (0.00294,0.0348] | (1.34,14.9] | (0,0.000255] | (0,0.192] |
| **292** | 2.30E-03 | 2.24E-03 | NA | 9.43E-01 | 9.18E-01 | NA | NA | NA | NA |
| **293** | 2.53E-03 | 1.88E-03 | NA | 8.79E-01 | 6.55E-01 | NA | NA | NA | NA |
| **294** | 1.94E-03 | 1.51E-03 | NA | 1.15E+00 | 8.97E-01 | NA | NA | NA | NA |
| **295** | 1.53E-02 | 1.32E-02 | 6.32E-01 | 1.21E+00 | 1.05E+00 | (0.0348,Inf] | (1.34,14.9] | (0.0184,Inf] | (0.192,21] |
| **296** | 4.15E-03 | 2.39E-03 | 0.00E+00 | 8.21E-01 | 4.72E-01 | (4.25e-05,0.00294] | (0,0.101] | NA | NA |
| **297** | 1.58E-03 | 1.70E-03 | NA | 9.87E-01 | 1.06E+00 | NA | NA | NA | NA |
| **298** | 3.32E-03 | 1.31E-03 | NA | 1.10E+00 | 4.33E-01 | NA | NA | NA | NA |
| **299** | 1.23E-02 | 5.83E-03 | NA | 1.02E+00 | 4.80E-01 | NA | NA | NA | NA |
| **300** | 5.64E-03 | 2.39E-03 | NA | 8.91E-01 | 3.78E-01 | NA | NA | NA | NA |

| | | | | | | | | | |
|---|---|---|---|---|---|---|---|---|---|
| **301** | 1.01E-03 | 1.86E-03 | NA | 8.53E-01 | 1.57E+00 | NA | NA | NA | NA |
| **302** | 1.28E-03 | 1.26E-03 | NA | 9.11E-01 | 8.93E-01 | NA | NA | NA | NA |
| **303** | 2.52E-03 | 3.56E-03 | NA | 7.88E-01 | 1.12E+00 | NA | NA | NA | NA |
| **304** | 2.64E-03 | 2.22E-03 | 0.00E+00 | 8.87E-01 | 7.45E-01 | (4.25e-05,0.00294] | (0.101,1.34] | NA | NA |
| **305** | 9.02E-03 | 1.91E-03 | 1.49E+00 | 9.82E-01 | 2.08E-01 | (4.25e-05,0.00294] | (0,0.101] | (0.000255,0.0184] | (0.192,21] |
| **306** | 2.12E-03 | 2.00E-03 | NA | 8.72E-01 | 8.25E-01 | NA | NA | NA | NA |
| **307** | 3.37E-03 | 3.00E-04 | NA | 1.00E+00 | 8.93E-02 | NA | NA | NA | NA |
| **308** | 1.81E-03 | 1.84E-03 | NA | 1.16E+00 | 1.18E+00 | NA | NA | NA | NA |
| **309** | 3.59E-03 | 2.30E-03 | NA | 1.07E+00 | 6.86E-01 | NA | NA | NA | NA |
| **310** | 2.75E-03 | 2.31E-03 | 0.00E+00 | 1.13E+00 | 9.48E-01 | (4.25e-05,0.00294] | (0.101,1.34] | NA | NA |
| **311** | 1.92E-03 | 6.18E-04 | NA | 1.04E+00 | 3.35E-01 | NA | NA | NA | NA |
| **312** | 2.38E-03 | 1.43E-03 | NA | 8.28E-01 | 4.98E-01 | NA | NA | NA | NA |
| **313** | 1.36E-03 | 8.38E-04 | NA | 1.03E+00 | 6.33E-01 | NA | NA | NA | NA |
| **314** | 3.25E-03 | 4.11E-03 | NA | 8.86E-01 | 1.12E+00 | NA | NA | NA | NA |
| **315** | 3.50E-03 | 1.02E-03 | NA | 1.22E+00 | 3.55E-01 | NA | NA | NA | NA |
| **316** | 1.95E-04 | 3.09E-04 | NA | 9.51E-01 | 1.51E+00 | NA | NA | NA | NA |
| **317** | 3.17E-03 | 1.39E-03 | NA | 1.09E+00 | 4.77E-01 | NA | NA | NA | NA |
| **318** | 5.50E-04 | 1.38E-03 | NA | 7.76E-01 | 1.95E+00 | NA | NA | NA | NA |
| **319** | 3.64E-03 | 3.52E-03 | NA | 9.37E-01 | 9.05E-01 | NA | NA | NA | NA |
| **320** | 7.04E-04 | 1.14E-03 | NA | 1.06E+00 | 1.71E+00 | NA | NA | NA | NA |
| **321** | 2.40E-03 | 1.54E-03 | NA | 9.89E-01 | 6.38E-01 | NA | NA | NA | NA |
| **322** | 1.30E-03 | 1.40E-03 | NA | 7.88E-01 | 8.49E-01 | NA | NA | NA | NA |
| **323** | 2.10E-03 | 4.64E-03 | NA | 8.38E-01 | 1.85E+00 | NA | NA | NA | NA |
| **324** | 4.42E-03 | 4.43E-03 | Inf | 1.04E+00 | 1.04E+00 | NA | NA | (0,0.000255] | (0,0.192] |
| **325** | 3.60E-03 | 4.19E-03 | 0.00E+00 | 1.01E+00 | 1.17E+00 | (4.25e-05,0.00294] | (0,0.101] | NA | NA |
| **326** | 5.69E-03 | 3.95E-03 | NA | 9.64E-01 | 6.70E-01 | NA | NA | NA | NA |
| **327** | 3.16E-03 | 5.70E-04 | Inf | 1.78E+00 | 3.21E-01 | NA | NA | (0,0.000255] | (0,0.192] |
| **328** | 7.13E-03 | 1.92E-03 | 0.00E+00 | 1.05E+00 | 2.83E-01 | (4.25e-05,0.00294] | (0.101,1.34] | NA | NA |
| **329** | 4.33E-03 | 3.04E-03 | NA | 1.17E+00 | 8.19E-01 | NA | NA | NA | NA |
| **330** | 4.81E-03 | 3.04E-03 | NA | 1.09E+00 | 6.92E-01 | NA | NA | NA | NA |
| **331** | 2.03E-03 | 2.04E-03 | NA | 8.51E-01 | 8.54E-01 | NA | NA | NA | NA |
| **332** | 2.10E-03 | 2.52E-03 | NA | 8.33E-01 | 1.00E+00 | NA | NA | NA | NA |
| **333** | 6.02E-03 | 2.61E-03 | Inf | 9.15E-01 | 3.97E-01 | NA | NA | (0.000255,0.0184] | (0,0.192] |
| **334** | 2.58E-03 | 2.73E-03 | NA | 1.06E+00 | 1.12E+00 | NA | NA | NA | NA |
| **335** | 2.21E-03 | 2.35E-03 | 4.54E-02 | 8.94E-01 | 9.47E-01 | (0.00294,0.0348] | (1.34,14.9] | (0.000255,0.0184] | (0,0.192] |
| **336** | 5.55E-03 | 7.16E-03 | NA | 1.09E+00 | 1.40E+00 | NA | NA | NA | NA |
| **337** | 1.81E-03 | 1.57E-03 | NA | 8.69E-01 | 7.52E-01 | NA | NA | NA | NA |
| **338** | 7.01E-03 | 6.14E-03 | Inf | 9.95E-01 | 8.70E-01 | NA | NA | (0.0184,Inf] | (0.192,21] |

| | | | | | | | | | |
|---|---|---|---|---|---|---|---|---|---|
| **339** | 6.14E-03 | 4.88E-03 | NA | 9.03E-01 | 7.18E-01 | NA | NA | NA | NA |
| **340** | 1.27E-03 | 1.39E-03 | NA | 9.17E-01 | 1.00E+00 | NA | NA | NA | NA |
| **341** | 1.40E-03 | 2.68E-03 | NA | 8.81E-01 | 1.69E+00 | NA | NA | NA | NA |
| **342** | 2.52E-03 | 2.74E-03 | 2.87E-02 | 8.37E-01 | 9.09E-01 | (0.00294,0.0348] | (1.34,14.9] | (0.000255,0.0184] | (0,0.192] |
| **343** | 1.42E-03 | 2.39E-03 | NA | 8.68E-01 | 1.47E+00 | NA | NA | NA | NA |
| **344** | 3.57E-03 | 2.73E-03 | 0.00E+00 | 1.06E+00 | 8.13E-01 | (0.00294,0.0348] | (0.101,1.34] | NA | NA |
| **345** | 3.80E-03 | 9.03E-03 | NA | 8.97E-01 | 2.13E+00 | NA | NA | NA | NA |
| **346** | 4.37E-03 | 9.36E-03 | NA | 8.03E-01 | 1.72E+00 | NA | NA | NA | NA |
| **347** | 2.15E-03 | 1.29E-03 | 0.00E+00 | 1.06E+00 | 6.34E-01 | (4.25e-05,0.00294] | (0.101,1.34] | NA | NA |
| **348** | 1.89E-03 | 2.14E-03 | NA | 9.16E-01 | 1.04E+00 | NA | NA | NA | NA |
| **349** | 2.53E-03 | 2.75E-03 | NA | 9.30E-01 | 1.01E+00 | NA | NA | NA | NA |
| **350** | 1.45E-03 | 8.50E-04 | NA | 7.29E-01 | 4.28E-01 | NA | NA | NA | NA |
| **351** | 1.81E-03 | 2.17E-03 | NA | 1.28E+00 | 1.54E+00 | NA | NA | NA | NA |
| **352** | 1.36E-03 | 1.71E-03 | NA | 9.94E-01 | 1.25E+00 | NA | NA | NA | NA |
| **353** | 1.45E-03 | 2.17E-03 | NA | 8.16E-01 | 1.23E+00 | NA | NA | NA | NA |
| **354** | 3.55E-03 | 2.76E-03 | 4.20E-02 | 1.28E+00 | 9.98E-01 | (0.00294,0.0348] | (1.34,14.9] | (0,0.000255] | (0,0.192] |
| **355** | 1.80E-03 | 2.99E-03 | NA | 1.03E+00 | 1.71E+00 | NA | NA | NA | NA |
| **356** | 7.00E-03 | 1.58E-03 | NA | 1.41E+00 | 3.17E-01 | NA | NA | NA | NA |
| **357** | 1.16E-03 | 1.61E-03 | NA | 9.19E-01 | 1.28E+00 | NA | NA | NA | NA |
| **358** | 1.07E-02 | 5.48E-03 | NA | 1.03E+00 | 5.29E-01 | NA | NA | NA | NA |
| **359** | 1.69E-03 | 2.85E-03 | 0.00E+00 | 1.00E+00 | 1.69E+00 | (4.25e-05,0.00294] | (0.101,1.34] | NA | NA |
| **360** | 4.29E-03 | 2.80E-03 | NA | 9.97E-01 | 6.50E-01 | NA | NA | NA | NA |
| **361** | 4.48E-03 | 5.12E-03 | NA | 1.17E+00 | 1.34E+00 | NA | NA | NA | NA |
| **362** | 4.72E-03 | 3.94E-03 | NA | 1.11E+00 | 9.22E-01 | NA | NA | NA | NA |
| **363** | 9.85E-04 | 2.09E-03 | 1.65E+00 | 6.12E-01 | 1.30E+00 | (0.00294,0.0348] | (1.34,14.9] | (0.000255,0.0184] | (0.192,21] |
| **364** | 9.85E-04 | 2.09E-03 | 1.65E+00 | 6.12E-01 | 1.30E+00 | (0.00294,0.0348] | (1.34,14.9] | (0.000255,0.0184] | (0.192,21] |
| **365** | 4.45E-03 | 5.65E-03 | Inf | 9.02E-01 | 1.14E+00 | NA | NA | (0.000255,0.0184] | (0.192,21] |
| **366** | 7.12E-03 | 2.50E-03 | NA | 1.15E+00 | 4.02E-01 | NA | NA | NA | NA |
| **367** | 4.02E-03 | 1.91E-03 | NA | 9.65E-01 | 4.57E-01 | NA | NA | NA | NA |
| **368** | 1.28E-03 | 1.89E-03 | NA | 1.07E+00 | 1.58E+00 | NA | NA | NA | NA |
| **369** | 1.82E-03 | 1.35E-03 | NA | 1.25E+00 | 9.26E-01 | NA | NA | NA | NA |
| **370** | 1.97E-03 | 2.00E-03 | NA | 8.96E-01 | 9.09E-01 | NA | NA | NA | NA |
| **371** | 2.83E-03 | 8.86E-04 | NA | 8.42E-01 | 2.64E-01 | NA | NA | NA | NA |
| **372** | 1.00E-03 | 1.85E-03 | NA | 1.24E+00 | 2.28E+00 | NA | NA | NA | NA |
| **373** | 4.47E-03 | 3.35E-03 | NA | 1.36E+00 | 1.02E+00 | NA | NA | NA | NA |
| **374** | 5.82E-04 | 1.80E-03 | NA | 9.44E-01 | 2.93E+00 | NA | NA | NA | NA |
| **375** | 2.07E-03 | 1.87E-03 | Inf | 1.09E+00 | 9.84E-01 | NA | NA | (0.000255,0.0184] | (0.192,21] |
| **376** | 2.42E-03 | 3.26E-03 | NA | 9.82E-01 | 1.32E+00 | NA | NA | NA | NA |

| | | | | | | | | | |
|---|---|---|---|---|---|---|---|---|---|
| **377** | 2.99E-03 | 2.78E-03 | NA | 8.71E-01 | 8.09E-01 | NA | NA | NA | NA |
| **378** | 4.60E-03 | 3.35E-03 | NA | 1.11E+00 | 8.09E-01 | NA | NA | NA | NA |
| **379** | 3.51E-03 | 3.68E-03 | 0.00E+00 | 1.15E+00 | 1.21E+00 | (4.25e-05,0.00294] | (0.101,1.34] | NA | NA |
| **380** | 1.88E-03 | 1.52E-03 | NA | 1.17E+00 | 9.41E-01 | NA | NA | NA | NA |
| **381** | 3.22E-03 | 1.44E-03 | NA | 9.49E-01 | 4.24E-01 | NA | NA | NA | NA |
| **382** | 2.64E-03 | 2.80E-03 | NA | 9.49E-01 | 1.01E+00 | NA | NA | NA | NA |
| **383** | 7.78E-03 | 6.66E-03 | NA | 1.46E+00 | 1.25E+00 | NA | NA | NA | NA |
| **384** | 1.79E-03 | 2.53E-03 | NA | 1.02E+00 | 1.44E+00 | NA | NA | NA | NA |
| **385** | 5.70E-03 | 5.33E-03 | 1.28E-01 | 1.27E+00 | 1.19E+00 | (0.0348,Inf] | (14.9,Inf] | (0.000255,0.0184] | (0.192,21] |
| **386** | 2.07E-03 | 2.25E-03 | 3.20E+00 | 9.01E-01 | 9.81E-01 | (4.25e-05,0.00294] | (0.101,1.34] | (0.000255,0.0184] | (0.192,21] |
| **387** | 6.28E-03 | 9.67E-03 | NA | 1.04E+00 | 1.61E+00 | NA | NA | NA | NA |
| **388** | NA | NA | NA | 8.94E-01 | NA | NA | NA | NA | NA |
| **389** | 5.84E-03 | 1.96E-03 | NA | 1.03E+00 | 3.46E-01 | NA | NA | NA | NA |
| **390** | 6.41E-04 | 4.12E-03 | NA | 8.09E-01 | 5.20E+00 | NA | NA | NA | NA |
| **391** | NA | NA | NA | 8.00E-01 | NA | NA | NA | NA | NA |
| **392** | 2.04E-03 | 2.29E-03 | NA | 8.97E-01 | 1.01E+00 | NA | NA | NA | NA |
| **393** | 3.04E-03 | 3.62E-03 | NA | 7.93E-01 | 9.44E-01 | NA | NA | NA | NA |
| **394** | 2.73E-02 | 9.93E-03 | NA | 1.33E+00 | 4.82E-01 | NA | NA | NA | NA |
| **395** | 2.50E-03 | 1.53E-03 | NA | 9.18E-01 | 5.62E-01 | NA | NA | NA | NA |
| **396** | 1.24E-03 | 1.49E-03 | NA | 9.62E-01 | 1.15E+00 | NA | NA | NA | NA |
| **397** | 2.53E-03 | 1.72E-03 | NA | 1.07E+00 | 7.29E-01 | NA | NA | NA | NA |
| **398** | 1.61E-03 | 1.18E-03 | NA | 1.20E+00 | 8.80E-01 | NA | NA | NA | NA |
| **399** | 3.82E-03 | 1.76E-03 | 2.25E+00 | 7.36E-01 | 3.38E-01 | (4.25e-05,0.00294] | (0.101,1.34] | (0.000255,0.0184] | (0.192,21] |
| **400** | 1.44E-02 | 2.90E-02 | NA | 4.58E-01 | 9.23E-01 | NA | NA | NA | NA |
| **401** | 1.71E-03 | 1.34E-03 | NA | 9.39E-01 | 7.40E-01 | NA | NA | NA | NA |
| **402** | 3.51E-03 | 3.28E-03 | NA | 8.16E-01 | 7.62E-01 | NA | NA | NA | NA |
| **403** | 1.18E-03 | 8.06E-04 | NA | 9.33E-01 | 6.35E-01 | NA | NA | NA | NA |
| **404** | 3.06E-03 | 3.71E-03 | 3.79E+00 | 1.06E+00 | 1.29E+00 | (0.00294,0.0348] | (1.34,14.9] | (0.0184,Inf] | (0.192,21] |
| **405** | 1.29E-02 | 9.77E-03 | 0.00E+00 | 1.10E+00 | 8.37E-01 | (4.25e-05,0.00294] | (0.101,1.34] | NA | NA |
| **406** | NA | NA | NA | 1.19E+00 | NA | NA | NA | NA | NA |
| **407** | 3.56E-03 | 1.93E-03 | 0.00E+00 | 1.13E+00 | 6.10E-01 | (0.00294,0.0348] | (1.34,14.9] | NA | NA |
| **408** | 2.32E-03 | 3.80E-03 | NA | 9.79E-01 | 1.60E+00 | NA | NA | NA | NA |
| **409** | 4.40E-03 | 2.07E-03 | Inf | 9.06E-01 | 4.26E-01 | NA | NA | (0.0184,Inf] | (0.192,21] |
| **410** | 1.68E-03 | 1.56E-03 | NA | 1.07E+00 | 9.97E-01 | NA | NA | NA | NA |
| **411** | 1.31E-02 | 1.25E-02 | 0.00E+00 | 9.00E-01 | 8.59E-01 | (4.25e-05,0.00294] | (0,0.101] | NA | NA |
| **412** | 1.02E-02 | 4.94E-03 | NA | 1.18E+00 | 5.71E-01 | NA | NA | NA | NA |
| **413** | 7.90E-04 | 6.48E-04 | NA | 7.37E-01 | 6.04E-01 | NA | NA | NA | NA |
| **414** | 7.27E-03 | 8.82E-03 | NA | 8.84E-01 | 1.07E+00 | NA | NA | NA | NA |

| | | | | | | | | | |
|---|---|---|---|---|---|---|---|---|---|
| **415** | 1.73E-03 | 1.40E-03 | NA | 1.10E+00 | 8.85E-01 | NA | NA | NA | NA |
| **416** | 2.25E-03 | 3.96E-03 | NA | 9.50E-01 | 1.67E+00 | NA | NA | NA | NA |
| **417** | 4.72E-03 | 6.09E-03 | NA | 6.12E-01 | 7.89E-01 | NA | NA | NA | NA |
| **418** | 2.49E-03 | 2.98E-03 | 2.37E+00 | 1.01E+00 | 1.20E+00 | (4.25e-05,0.00294] | (0.101,1.34] | (0.000255,0.0184] | (0.192,21] |
| **419** | 3.22E-03 | 4.42E-03 | NA | 8.89E-01 | 1.22E+00 | NA | NA | NA | NA |
| **420** | 2.96E-03 | 2.06E-03 | 0.00E+00 | 1.25E+00 | 8.68E-01 | (4.25e-05,0.00294] | (0,0.101] | NA | NA |
| **421** | 2.39E-03 | 1.27E-03 | NA | 8.13E-01 | 4.30E-01 | NA | NA | NA | NA |
| **422** | 3.83E-03 | 4.35E-04 | NA | 1.14E+00 | 1.29E-01 | NA | NA | NA | NA |
| **423** | 4.43E-03 | 4.77E-03 | NA | 9.89E-01 | 1.06E+00 | NA | NA | NA | NA |
| **424** | 1.79E-03 | 1.00E-03 | NA | 9.13E-01 | 5.12E-01 | NA | NA | NA | NA |
| **425** | 2.18E-03 | 1.32E-03 | NA | 1.24E+00 | 7.53E-01 | NA | NA | NA | NA |
| **426** | 7.44E-03 | 5.89E-03 | NA | 1.06E+00 | 8.37E-01 | NA | NA | NA | NA |
| **427** | 4.58E-03 | 2.57E-03 | 0.00E+00 | 1.04E+00 | 5.83E-01 | (0.00294,0.0348] | (1.34,14.9] | NA | NA |
| **428** | 7.11E-03 | 6.24E-03 | NA | 1.37E+00 | 1.20E+00 | NA | NA | NA | NA |
| **429** | 7.40E-03 | 6.45E-03 | NA | 1.13E+00 | 9.87E-01 | NA | NA | NA | NA |
| **430** | 1.07E-02 | 5.00E-03 | 1.16E-01 | 8.97E-01 | 4.19E-01 | (0.00294,0.0348] | (0.101,1.34] | (0.000255,0.0184] | (0.192,21] |
| **431** | 6.26E-03 | 2.74E-03 | NA | 9.22E-01 | 4.03E-01 | NA | NA | NA | NA |
| **432** | 6.51E-03 | 3.75E-03 | NA | 8.49E-01 | 4.88E-01 | NA | NA | NA | NA |
| **433** | 4.73E-04 | 1.03E-03 | NA | 7.70E-01 | 1.67E+00 | NA | NA | NA | NA |
| **434** | 2.18E-03 | 2.20E-03 | 5.63E-01 | 7.61E-01 | 7.71E-01 | (0.00294,0.0348] | (1.34,14.9] | (0.000255,0.0184] | (0.192,21] |
| **435** | 2.93E-02 | 1.01E-02 | 1.90E+00 | 1.41E+00 | 4.86E-01 | (0.00294,0.0348] | (0.101,1.34] | (0.000255,0.0184] | (0.192,21] |
| **436** | 4.67E-03 | 5.65E-03 | Inf | 8.75E-01 | 1.06E+00 | NA | NA | (0.000255,0.0184] | (0.192,21] |
| **437** | 6.33E-03 | 4.19E-03 | NA | 9.43E-01 | 6.24E-01 | NA | NA | NA | NA |
| **438** | 3.83E-03 | 2.20E-03 | NA | 1.01E+00 | 5.79E-01 | NA | NA | NA | NA |
| **439** | 5.59E-03 | 3.31E-03 | NA | 8.81E-01 | 5.22E-01 | NA | NA | NA | NA |
| **440** | 4.53E-03 | 9.64E-04 | NA | 4.46E+00 | 9.48E-01 | NA | NA | NA | NA |
| **441** | 2.09E-03 | 5.37E-04 | NA | 6.53E-01 | 1.68E-01 | NA | NA | NA | NA |
| **442** | 6.32E-03 | 1.98E-03 | 0.00E+00 | 1.17E+00 | 3.67E-01 | (0.00294,0.0348] | (0.101,1.34] | NA | NA |
| **443** | 6.32E-03 | 1.98E-03 | 0.00E+00 | 1.17E+00 | 3.67E-01 | (0.00294,0.0348] | (0.101,1.34] | NA | NA |
| **444** | 4.59E-03 | 6.62E-03 | NA | 8.85E-01 | 1.28E+00 | NA | NA | NA | NA |
| **445** | 5.44E-03 | 2.34E-03 | NA | 8.20E-01 | 3.53E-01 | NA | NA | NA | NA |
| **446** | 2.89E-03 | 4.98E-03 | 2.92E-01 | 9.48E-01 | 1.63E+00 | (0.00294,0.0348] | (1.34,14.9] | (0.000255,0.0184] | (0.192,21] |
| **447** | 3.29E-03 | 1.79E-03 | NA | 8.95E-01 | 4.89E-01 | NA | NA | NA | NA |
| **448** | 6.00E-03 | 3.02E-03 | 0.00E+00 | 1.14E+00 | 5.73E-01 | (4.25e-05,0.00294] | (0.101,1.34] | NA | NA |
| **449** | 7.70E-03 | 2.39E-03 | NA | 1.08E+00 | 3.36E-01 | NA | NA | NA | NA |
| **450** | 7.66E-03 | 2.64E-03 | 0.00E+00 | 1.11E+00 | 3.82E-01 | (4.25e-05,0.00294] | (0.101,1.34] | NA | NA |
| **451** | 1.64E-03 | 1.14E-03 | Inf | 8.99E-01 | 6.25E-01 | NA | NA | (0.000255,0.0184] | (0.192,21] |
| **452** | 1.64E-03 | 1.14E-03 | Inf | 8.99E-01 | 6.25E-01 | NA | NA | (0.000255,0.0184] | (0.192,21] |

| | | | | | | | | | | |
|---|---|---|---|---|---|---|---|---|---|---|
| **453** | 3.52E-03 | 3.76E-03 | NA | 7.55E-01 | 8.07E-01 | NA | NA | NA | NA |
| **454** | 1.63E-03 | 2.00E-03 | NA | 9.67E-01 | 1.18E+00 | NA | NA | NA | NA |
| **455** | 2.26E-03 | 2.21E-03 | NA | 8.10E-01 | 7.92E-01 | NA | NA | NA | NA |
| **456** | 1.89E-03 | 1.65E-03 | NA | 8.50E-01 | 7.42E-01 | NA | NA | NA | NA |
| **457** | 2.41E-03 | 1.82E-03 | NA | 8.86E-01 | 6.71E-01 | NA | NA | NA | NA |
| **458** | 6.20E-04 | 1.85E-03 | NA | 1.00E+00 | 2.98E+00 | NA | NA | NA | NA |
| **459** | 3.23E-03 | 2.36E-03 | 0.00E+00 | 9.02E-01 | 6.60E-01 | (4.25e-05,0.00294] | (0,0.101] | NA | NA |
| **460** | 1.66E-03 | 0.00E+00 | 2.01E+00 | 8.15E-01 | 0.00E+00 | (0.00294,0.0348] | (1.34,14.9] | (0.000255,0.0184] | (21,Inf] |
| **461** | 1.71E-03 | 1.07E-03 | 0.00E+00 | 1.04E+00 | 6.50E-01 | (4.25e-05,0.00294] | (0.101,1.34] | NA | NA |
| **462** | 2.63E-03 | 7.31E-04 | NA | 9.30E-01 | 2.59E-01 | NA | NA | NA | NA |
| **463** | 4.88E-03 | 2.13E-03 | NA | 1.47E+00 | 6.39E-01 | NA | NA | NA | NA |
| **464** | 1.59E-03 | 1.67E-03 | 2.30E+02 | 6.80E-01 | 7.16E-01 | (4.25e-05,0.00294] | (0.101,1.34] | (0.0184,Inf] | (21,Inf] |
| **465** | 1.59E-03 | 1.67E-03 | 2.30E+02 | 6.80E-01 | 7.16E-01 | (4.25e-05,0.00294] | (0.101,1.34] | (0.0184,Inf] | (21,Inf] |
| **466** | 7.85E-03 | 2.26E-03 | NA | 9.94E-01 | 2.87E-01 | NA | NA | NA | NA |
| **467** | 2.14E-03 | 3.28E-03 | NA | 6.61E-01 | 1.01E+00 | NA | NA | NA | NA |
| **468** | 1.21E-03 | 1.93E-03 | Inf | 6.79E-01 | 1.08E+00 | NA | NA | (0.000255,0.0184] | (0.192,21] |
| **469** | 2.44E-03 | 6.60E-03 | 0.00E+00 | 1.10E+00 | 2.98E+00 | (4.25e-05,0.00294] | (0.101,1.34] | NA | NA |
| **470** | 3.94E-03 | 8.01E-03 | NA | 6.48E-01 | 1.32E+00 | NA | NA | NA | NA |
| **471** | 1.17E-03 | 1.93E-03 | NA | 6.28E-01 | 1.04E+00 | NA | NA | NA | NA |
| **472** | 1.43E-03 | 9.78E-04 | NA | 8.99E-01 | 6.13E-01 | NA | NA | NA | NA |
| **473** | 2.40E-03 | 2.67E-03 | NA | 8.62E-01 | 9.57E-01 | NA | NA | NA | NA |
| **474** | 2.68E-03 | 5.18E-03 | NA | 8.16E-01 | 1.58E+00 | NA | NA | NA | NA |
| **475** | 5.24E-03 | 5.84E-03 | 0.00E+00 | 9.53E-01 | 1.06E+00 | (0.00294,0.0348] | (0.101,1.34] | NA | NA |
| **476** | 5.96E-03 | 3.92E-03 | NA | 1.18E+00 | 7.81E-01 | NA | NA | NA | NA |
| **477** | 3.49E-03 | 2.43E-03 | 0.00E+00 | 1.03E+00 | 7.16E-01 | (4.25e-05,0.00294] | (0.101,1.34] | NA | NA |
| **478** | 3.85E-03 | 2.62E-03 | NA | 1.08E+00 | 7.37E-01 | NA | NA | NA | NA |
| **479** | 1.85E-03 | 3.44E-04 | NA | 8.91E-01 | 1.66E-01 | NA | NA | NA | NA |
| **480** | 4.77E-03 | 1.39E-03 | NA | 8.73E-01 | 2.55E-01 | NA | NA | NA | NA |
| **481** | 6.25E-03 | 2.72E-03 | NA | 9.88E-01 | 4.29E-01 | NA | NA | NA | NA |
| **482** | 4.93E-03 | 6.37E-03 | NA | 1.04E+00 | 1.35E+00 | NA | NA | NA | NA |
| **483** | 4.90E-03 | 2.66E-03 | NA | 1.12E+00 | 6.07E-01 | NA | NA | NA | NA |
| **484** | 4.28E-03 | 6.74E-03 | 4.44E-01 | 1.26E+00 | 1.98E+00 | (0.00294,0.0348] | (1.34,14.9] | (0.000255,0.0184] | (0.192,21] |
| **485** | 1.54E-03 | 1.06E-03 | NA | 8.17E-01 | 5.63E-01 | NA | NA | NA | NA |
| **486** | 4.81E-03 | 8.66E-03 | NA | 9.50E-01 | 1.71E+00 | NA | NA | NA | NA |
| **487** | 1.90E-03 | 1.04E-03 | NA | 1.05E+00 | 5.75E-01 | NA | NA | NA | NA |
| **488** | 4.16E-03 | 5.05E-03 | NA | 9.34E-01 | 1.13E+00 | NA | NA | NA | NA |
| **489** | 7.41E-03 | 4.30E-03 | 2.42E+00 | 1.06E+00 | 6.16E-01 | (0.00294,0.0348] | (0.101,1.34] | (0.000255,0.0184] | (0.192,21] |
| **490** | 3.26E-03 | 2.29E-03 | NA | 8.52E-01 | 5.99E-01 | NA | NA | NA | NA |

| | | | | | | | | | |
|---|---|---|---|---|---|---|---|---|---|
| **491** | 3.36E-03 | 3.67E-03 | NA | 1.05E+00 | 1.14E+00 | NA | NA | NA | NA |
| **492** | 4.09E-03 | 2.98E-03 | NA | 9.87E-01 | 7.18E-01 | NA | NA | NA | NA |
| **493** | 1.50E-03 | 1.19E-03 | NA | 1.05E+00 | 8.28E-01 | NA | NA | NA | NA |
| **494** | 4.33E-03 | 6.47E-03 | 7.95E-01 | 9.54E-01 | 1.43E+00 | (0.00294,0.0348] | (1.34,14.9] | (0.0184,Inf] | (0.192,21] |
| **495** | 3.15E-03 | 2.38E-03 | 6.83E+00 | 9.90E-01 | 7.49E-01 | (0.00294,0.0348] | (1.34,14.9] | (0.0184,Inf] | (0.192,21] |
| **496** | 1.26E-03 | 1.85E-03 | NA | 8.38E-01 | 1.23E+00 | NA | NA | NA | NA |
| **497** | 4.09E-03 | 3.26E-03 | NA | 7.33E-01 | 5.85E-01 | NA | NA | NA | NA |
| **498** | 2.46E-03 | 2.96E-03 | 0.00E+00 | 8.52E-01 | 1.02E+00 | (4.25e-05,0.00294] | (0.101,1.34] | NA | NA |
| **499** | 1.02E-02 | 5.45E-03 | NA | 1.40E+00 | 7.50E-01 | NA | NA | NA | NA |
| **500** | 3.44E-03 | 1.60E-03 | NA | 1.46E+00 | 6.79E-01 | NA | NA | NA | NA |
| **501** | 2.38E-03 | 2.54E-03 | NA | 1.09E+00 | 1.16E+00 | NA | NA | NA | NA |
| **502** | 3.01E-03 | 1.90E-03 | NA | 1.03E+00 | 6.49E-01 | NA | NA | NA | NA |
| **503** | 3.35E-03 | 3.10E-03 | NA | 7.94E-01 | 7.34E-01 | NA | NA | NA | NA |
| **504** | 2.31E-03 | 3.95E-03 | NA | 3.21E-01 | 5.49E-01 | NA | NA | NA | NA |
| **505** | 4.18E-03 | 4.01E-03 | NA | 1.04E+00 | 9.97E-01 | NA | NA | NA | NA |
| **506** | 2.44E-03 | 2.03E-03 | NA | 9.24E-01 | 7.71E-01 | NA | NA | NA | NA |
| **507** | 1.94E-02 | 3.62E-02 | NA | 4.05E-01 | 7.55E-01 | NA | NA | NA | NA |
| **508** | 2.81E-03 | 3.49E-03 | NA | 7.60E-01 | 9.44E-01 | NA | NA | NA | NA |
| **509** | 5.40E-03 | 1.13E-02 | 0.00E+00 | 1.16E+00 | 2.41E+00 | (4.25e-05,0.00294] | (0.101,1.34] | NA | NA |
| **510** | 1.87E-03 | 1.65E-03 | NA | 8.67E-01 | 7.68E-01 | NA | NA | NA | NA |
| **511** | 1.40E-03 | 2.19E-03 | NA | 6.79E-01 | 1.06E+00 | NA | NA | NA | NA |
| **512** | 3.38E-03 | 5.60E-03 | NA | 1.51E+00 | 2.50E+00 | NA | NA | NA | NA |
| **513** | 2.52E-03 | 2.99E-03 | NA | 8.97E-01 | 1.06E+00 | NA | NA | NA | NA |
| **514** | 1.84E-02 | 1.66E-02 | NA | 1.12E+00 | 1.01E+00 | NA | NA | NA | NA |
| **515** | 1.39E-03 | 2.32E-03 | 0.00E+00 | 1.00E+00 | 1.67E+00 | (4.25e-05,0.00294] | (0.101,1.34] | NA | NA |
| **516** | 5.08E-03 | 1.42E-03 | NA | 1.08E+00 | 3.02E-01 | NA | NA | NA | NA |
| **517** | 9.70E-03 | 8.03E-03 | NA | 1.03E+00 | 8.52E-01 | NA | NA | NA | NA |
| **518** | 3.20E-03 | 3.20E-03 | NA | 1.05E+00 | 1.05E+00 | NA | NA | NA | NA |
| **519** | 7.92E-04 | 0.00E+00 | NA | 1.13E+00 | 0.00E+00 | NA | NA | NA | NA |
| **520** | 2.87E-03 | 1.15E-03 | NA | 1.26E+00 | 5.07E-01 | NA | NA | NA | NA |
| **521** | 2.44E-03 | 3.93E-03 | NA | 7.87E-01 | 1.27E+00 | NA | NA | NA | NA |
| **522** | 4.88E-03 | 1.57E-03 | NA | 1.07E+00 | 3.46E-01 | NA | NA | NA | NA |
| **523** | 3.62E-03 | 2.18E-03 | 3.65E-01 | 9.00E-01 | 5.41E-01 | (4.25e-05,0.00294] | (0.101,1.34] | (0.000255,0.0184] | (0,0.192] |
| **524** | 7.49E-04 | 1.66E-03 | NA | 9.41E-01 | 2.09E+00 | NA | NA | NA | NA |
| **525** | 2.28E-03 | 2.79E-03 | NA | 8.23E-01 | 1.01E+00 | NA | NA | NA | NA |
| **526** | 7.25E-03 | 8.46E-03 | 2.95E-02 | 9.44E-01 | 1.10E+00 | (0.00294,0.0348] | (1.34,14.9] | (0.000255,0.0184] | (0,0.192] |
| **527** | 2.36E-03 | 6.81E-03 | NA | 8.54E-01 | 2.47E+00 | NA | NA | NA | NA |
| **528** | 2.97E-03 | 1.29E-03 | NA | 9.16E-01 | 3.98E-01 | NA | NA | NA | NA |

| | | | | | | | | | |
|---|---|---|---|---|---|---|---|---|---|
| **529** | 1.04E-02 | 1.19E-02 | 0.00E+00 | 9.11E-01 | 1.04E+00 | (4.25e-05,0.00294] | (0,0.101] | NA | NA |
| **530** | 5.81E-03 | 4.82E-03 | NA | 8.75E-01 | 7.26E-01 | NA | NA | NA | NA |
| **531** | 4.49E-03 | 4.17E-03 | NA | 1.17E+00 | 1.09E+00 | NA | NA | NA | NA |
| **532** | 1.77E-03 | 1.32E-03 | NA | 8.39E-01 | 6.24E-01 | NA | NA | NA | NA |
| **533** | 4.25E-03 | 2.93E-03 | NA | 1.10E+00 | 7.57E-01 | NA | NA | NA | NA |
| **534** | 3.12E-03 | 2.64E-03 | NA | 9.50E-01 | 8.06E-01 | NA | NA | NA | NA |
| **535** | 3.03E-03 | 2.66E-03 | Inf | 1.16E+00 | 1.01E+00 | NA | NA | (0,0.000255] | (0,0.192] |
| **536** | 6.67E-03 | 1.78E-03 | NA | 1.01E+00 | 2.70E-01 | NA | NA | NA | NA |
| **537** | 1.32E-03 | 4.60E-04 | NA | 9.61E-01 | 3.34E-01 | NA | NA | NA | NA |
| **538** | 2.60E-03 | 4.17E-04 | NA | 1.05E+00 | 1.68E-01 | NA | NA | NA | NA |
| **539** | 2.32E-03 | 1.57E-03 | NA | 8.33E-01 | 5.64E-01 | NA | NA | NA | NA |
| **540** | 1.94E-03 | 2.65E-03 | NA | 1.03E+00 | 1.42E+00 | NA | NA | NA | NA |
| **541** | 0.00E+00 | 2.81E-04 | NA | 5.06E-01 | Inf | NA | NA | NA | NA |
| **542** | 3.03E-03 | 2.88E-03 | NA | 8.54E-01 | 8.12E-01 | NA | NA | NA | NA |
| **543** | 1.60E-03 | 3.13E-03 | 0.00E+00 | 1.04E+00 | 2.04E+00 | (0.00294,0.0348] | (1.34,14.9] | NA | NA |
| **544** | 4.73E-03 | 2.11E-03 | 0.00E+00 | 9.58E-01 | 4.29E-01 | (0.00294,0.0348] | (1.34,14.9] | NA | NA |
| **545** | NA | NA | NA | 1.43E+00 | NA | NA | NA | NA | NA |
| **546** | 3.40E-03 | 9.17E-04 | NA | 1.16E+00 | 3.13E-01 | NA | NA | NA | NA |
| **547** | 5.76E-03 | 3.69E-03 | NA | 7.88E-01 | 5.05E-01 | NA | NA | NA | NA |
| **548** | 5.06E-03 | 3.56E-03 | NA | 1.06E+00 | 7.43E-01 | NA | NA | NA | NA |
| **549** | 4.45E-03 | 1.48E-02 | NA | 3.53E-01 | 1.18E+00 | NA | NA | NA | NA |
| **550** | 1.94E-03 | 2.25E-03 | NA | 1.20E+00 | 1.39E+00 | NA | NA | NA | NA |
| **551** | 2.92E-03 | 3.44E-03 | 0.00E+00 | 9.05E-01 | 1.06E+00 | (4.25e-05,0.00294] | (0.101,1.34] | NA | NA |
| **552** | 2.16E-03 | 8.43E-04 | NA | 1.25E+00 | 4.88E-01 | NA | NA | NA | NA |
| **553** | 1.13E-03 | 2.38E-03 | 0.00E+00 | 1.06E+00 | 2.24E+00 | (4.25e-05,0.00294] | (0.101,1.34] | NA | NA |
| **554** | 3.51E-03 | 1.14E-03 | NA | 7.76E-01 | 2.52E-01 | NA | NA | NA | NA |
| **555** | 1.58E-03 | 1.85E-03 | NA | 7.22E-01 | 8.47E-01 | NA | NA | NA | NA |
| **556** | 2.00E-03 | 0.00E+00 | NA | 7.13E-01 | 0.00E+00 | NA | NA | NA | NA |
| **557** | 3.24E-03 | 9.84E-04 | NA | 1.11E+00 | 3.38E-01 | NA | NA | NA | NA |
| **558** | 3.95E-03 | 2.37E-03 | NA | 1.11E+00 | 6.67E-01 | NA | NA | NA | NA |
| **559** | 1.65E-03 | 3.42E-03 | NA | 9.19E-01 | 1.90E+00 | NA | NA | NA | NA |
| **560** | 4.07E-03 | 1.52E-03 | NA | 9.21E-01 | 3.44E-01 | NA | NA | NA | NA |
| **561** | 4.47E-03 | 1.49E-03 | NA | 9.37E-01 | 3.12E-01 | NA | NA | NA | NA |
| **562** | 5.46E-03 | 4.16E-04 | NA | 1.16E+00 | 8.88E-02 | NA | NA | NA | NA |
| **563** | 1.51E-03 | 1.24E-03 | NA | 1.22E+00 | 9.93E-01 | NA | NA | NA | NA |
| **564** | 3.36E-03 | 8.15E-04 | NA | 8.82E-01 | 2.14E-01 | NA | NA | NA | NA |
| **565** | 1.84E-03 | 3.44E-03 | NA | 9.99E-01 | 1.87E+00 | NA | NA | NA | NA |
| **566** | 2.07E-03 | 9.55E-04 | NA | 1.13E+00 | 5.22E-01 | NA | NA | NA | NA |

| | | | | | | | | | |
|---|---|---|---|---|---|---|---|---|---|
| **567** | 1.08E-02 | 9.93E-03 | NA | 1.14E+00 | 1.05E+00 | NA | NA | NA | NA |
| **568** | 9.75E-03 | 4.54E-03 | NA | 9.47E-01 | 4.41E-01 | NA | NA | NA | NA |
| **569** | 2.57E-03 | 2.32E-03 | NA | 9.36E-01 | 8.47E-01 | NA | NA | NA | NA |
| **570** | 7.26E-03 | 3.35E-03 | 0.00E+00 | 9.01E-01 | 4.15E-01 | (4.25e-05,0.00294] | (0.101,1.34] | NA | NA |
| **571** | 1.25E-03 | 1.56E-03 | NA | 7.64E-01 | 9.59E-01 | NA | NA | NA | NA |
| **572** | 1.42E-02 | 8.78E-03 | NA | 9.63E-01 | 5.97E-01 | NA | NA | NA | NA |
| **573** | 6.83E-03 | 5.50E-03 | NA | 1.59E+00 | 1.28E+00 | NA | NA | NA | NA |
| **574** | 5.55E-03 | 1.91E-03 | NA | 1.24E+00 | 4.26E-01 | NA | NA | NA | NA |
| **575** | 7.02E-03 | 2.85E-03 | NA | 1.16E+00 | 4.70E-01 | NA | NA | NA | NA |
| **576** | 1.99E-03 | 3.25E-03 | NA | 1.20E+00 | 1.96E+00 | NA | NA | NA | NA |
| **577** | 1.96E-03 | 2.72E-03 | NA | 1.02E+00 | 1.41E+00 | NA | NA | NA | NA |
| **578** | 5.11E-03 | 5.15E-03 | NA | 1.03E+00 | 1.04E+00 | NA | NA | NA | NA |
| **579** | 2.55E-03 | 1.82E-03 | NA | 1.09E+00 | 7.74E-01 | NA | NA | NA | NA |
| **580** | 4.48E-03 | 3.52E-03 | NA | 1.09E+00 | 8.57E-01 | NA | NA | NA | NA |
| **581** | 2.05E-03 | 3.33E-03 | NA | 9.79E-01 | 1.59E+00 | NA | NA | NA | NA |
| **582** | 2.16E-03 | 1.35E-03 | NA | 1.36E+00 | 8.46E-01 | NA | NA | NA | NA |
| **583** | 1.19E-02 | 7.86E-03 | 2.14E-02 | 1.26E+00 | 8.34E-01 | (0.0348,Inf] | (1.34,14.9] | (0.000255,0.0184] | (0,0.192] |
| **584** | 3.62E-03 | 3.90E-03 | NA | 6.46E-01 | 6.97E-01 | NA | NA | NA | NA |
| **585** | 2.33E-03 | 3.09E-03 | NA | 5.53E-01 | 7.31E-01 | NA | NA | NA | NA |
| **586** | 3.65E-03 | 1.73E-03 | NA | 9.59E-01 | 4.54E-01 | NA | NA | NA | NA |
| **587** | 1.43E-03 | 1.44E-03 | NA | 8.90E-01 | 8.90E-01 | NA | NA | NA | NA |
| **588** | 3.89E-04 | 4.62E-04 | NA | 7.73E-01 | 9.18E-01 | NA | NA | NA | NA |
| **589** | 2.72E-03 | 2.70E-03 | NA | 9.65E-01 | 9.59E-01 | NA | NA | NA | NA |
| **590** | 8.97E-04 | 7.03E-04 | NA | 9.07E-01 | 7.11E-01 | NA | NA | NA | NA |
| **591** | 1.22E-03 | 2.89E-03 | NA | 8.25E-01 | 1.96E+00 | NA | NA | NA | NA |
| **592** | 2.16E-03 | 3.13E-03 | NA | 1.03E+00 | 1.50E+00 | NA | NA | NA | NA |
| **593** | 1.57E-03 | 5.79E-03 | NA | 8.03E-01 | 2.97E+00 | NA | NA | NA | NA |
| **594** | 1.85E-03 | 1.61E-03 | NA | 8.28E-01 | 7.23E-01 | NA | NA | NA | NA |
| **595** | 5.68E-03 | 2.74E-03 | NA | 1.13E+00 | 5.45E-01 | NA | NA | NA | NA |
| **596** | 4.23E-03 | 0.00E+00 | NA | 1.21E+00 | 0.00E+00 | NA | NA | NA | NA |
| **597** | 2.91E-03 | 1.21E-03 | NA | 9.68E-01 | 4.01E-01 | NA | NA | NA | NA |
| **598** | 6.10E-03 | 3.84E-03 | 7.05E-01 | 1.13E+00 | 7.11E-01 | (0.0348,Inf] | (1.34,14.9] | (0.0184,Inf] | (0.192,21] |
| **599** | 2.14E-03 | 5.62E-03 | NA | 8.79E-01 | 2.31E+00 | NA | NA | NA | NA |
| **600** | 3.17E-03 | 7.37E-04 | NA | 8.38E-01 | 1.95E-01 | NA | NA | NA | NA |
| **601** | 6.26E-03 | 6.39E-03 | NA | 6.87E-01 | 7.01E-01 | NA | NA | NA | NA |
| **602** | 9.08E-03 | 2.49E-03 | NA | 1.13E+00 | 3.10E-01 | NA | NA | NA | NA |
| **603** | 3.80E-03 | 6.77E-03 | 0.00E+00 | 8.53E-01 | 1.52E+00 | (0.00294,0.0348] | (1.34,14.9] | NA | NA |
| **604** | 2.32E-03 | 4.90E-03 | NA | 1.13E+00 | 2.39E+00 | NA | NA | NA | NA |

| | | | | | | | | | |
|---|---|---|---|---|---|---|---|---|---|
| **605** | 2.58E-03 | 5.52E-04 | 1.30E+01 | 7.38E-01 | 1.58E-01 | (0.00294,0.0348] | (1.34,14.9] | (0.0184,Inf] | (21,Inf] |
| **606** | 1.90E-03 | 6.07E-03 | NA | 6.36E-01 | 2.03E+00 | NA | NA | NA | NA |
| **607** | 1.73E-02 | 1.10E-02 | NA | 8.75E-01 | 5.57E-01 | NA | NA | NA | NA |
| **608** | 4.25E-03 | 1.39E-03 | NA | 9.03E-01 | 2.95E-01 | NA | NA | NA | NA |
| **609** | 6.04E-03 | 1.53E-03 | NA | 1.03E+00 | 2.61E-01 | NA | NA | NA | NA |
| **610** | 1.74E-03 | 1.61E-03 | NA | 1.03E+00 | 9.60E-01 | NA | NA | NA | NA |
| **611** | 2.32E-03 | 2.84E-03 | NA | 1.12E+00 | 1.37E+00 | NA | NA | NA | NA |
| **612** | NA | NA | NA | 1.10E+00 | NA | NA | NA | NA | NA |
| **613** | 3.15E-03 | 3.41E-03 | NA | 1.04E+00 | 1.13E+00 | NA | NA | NA | NA |
| **614** | 2.17E-03 | 2.40E-03 | NA | 7.21E-01 | 7.97E-01 | NA | NA | NA | NA |
| **615** | 3.54E-03 | 2.04E-03 | NA | 6.12E-01 | 3.54E-01 | NA | NA | NA | NA |
| **616** | 2.52E-03 | 3.10E-03 | NA | 1.16E+00 | 1.43E+00 | NA | NA | NA | NA |
| **617** | 2.31E-03 | 1.81E-03 | 0.00E+00 | 8.20E-01 | 6.43E-01 | (4.25e-05,0.00294] | (0.101,1.34] | NA | NA |
| **618** | 2.16E-03 | 1.97E-02 | 4.00E-02 | 1.47E+00 | 1.34E+01 | (0.0348,Inf] | (14.9,Inf] | (0.000255,0.0184] | (0.192,21] |
| **619** | 5.09E-03 | 3.33E-03 | NA | 1.05E+00 | 6.88E-01 | NA | NA | NA | NA |
| **620** | 3.02E-03 | 3.11E-03 | NA | 7.53E-01 | 7.75E-01 | NA | NA | NA | NA |
| **621** | 2.03E-03 | 7.67E-04 | NA | 9.33E-01 | 3.53E-01 | NA | NA | NA | NA |
| **622** | 1.26E-03 | 2.72E-03 | NA | 6.64E-01 | 1.43E+00 | NA | NA | NA | NA |
| **623** | 4.51E-03 | 3.06E-03 | NA | 9.85E-01 | 6.69E-01 | NA | NA | NA | NA |
| **624** | 5.77E-04 | 1.73E-03 | NA | 1.42E+00 | 4.24E+00 | NA | NA | NA | NA |
| **625** | 1.74E-03 | 7.27E-04 | NA | 8.08E-01 | 3.38E-01 | NA | NA | NA | NA |
| **626** | 3.58E-03 | 5.95E-03 | NA | 1.05E+00 | 1.75E+00 | NA | NA | NA | NA |
| **627** | 9.96E-03 | 6.54E-03 | NA | 1.39E+00 | 9.12E-01 | NA | NA | NA | NA |
| **628** | 2.82E-02 | 4.40E-04 | NA | 1.31E+00 | 2.05E-02 | NA | NA | NA | NA |
| **629** | 4.41E-03 | 6.76E-03 | 8.06E-01 | 8.76E-01 | 1.34E+00 | (4.25e-05,0.00294] | (0.101,1.34] | (0.000255,0.0184] | (0,0.192] |
| **630** | 3.27E-03 | 1.39E-02 | NA | 8.98E-01 | 3.83E+00 | NA | NA | NA | NA |
| **631** | 1.96E-03 | 1.58E-03 | NA | 7.33E-01 | 5.94E-01 | NA | NA | NA | NA |
| **632** | 2.05E-03 | 2.93E-03 | NA | 9.98E-01 | 1.43E+00 | NA | NA | NA | NA |
| **633** | 1.12E-03 | 6.63E-04 | NA | 8.82E-01 | 5.20E-01 | NA | NA | NA | NA |
| **634** | 2.23E-03 | 2.52E-03 | NA | 7.16E-01 | 8.08E-01 | NA | NA | NA | NA |
| **635** | 2.39E-03 | 2.12E-03 | NA | 1.11E+00 | 9.90E-01 | NA | NA | NA | NA |
| **636** | 2.18E-03 | 2.79E-03 | NA | 6.61E-01 | 8.48E-01 | NA | NA | NA | NA |
| **637** | 1.33E-03 | 2.09E-03 | NA | 1.22E+00 | 1.92E+00 | NA | NA | NA | NA |
| **638** | 4.38E-04 | 1.84E-03 | NA | 7.36E-01 | 3.09E+00 | NA | NA | NA | NA |
| **639** | 4.40E-03 | 5.25E-03 | NA | 9.15E-01 | 1.09E+00 | NA | NA | NA | NA |
| **640** | 1.10E-03 | 1.40E-03 | NA | 7.72E-01 | 9.85E-01 | NA | NA | NA | NA |
| **641** | 4.61E-03 | 2.40E-03 | NA | 1.27E+00 | 6.63E-01 | NA | NA | NA | NA |
| **642** | 5.73E-03 | 2.19E-03 | NA | 8.97E-01 | 3.43E-01 | NA | NA | NA | NA |

| | | | | | | | | | |
|---|---|---|---|---|---|---|---|---|---|
| **643** | 4.77E-03 | 4.95E-03 | NA | 8.29E-01 | 8.60E-01 | NA | NA | NA | NA |
| **644** | 8.27E-03 | 5.17E-03 | NA | 6.64E-01 | 4.15E-01 | NA | NA | NA | NA |
| **645** | 3.68E-03 | 2.02E-03 | NA | 4.48E-01 | 2.45E-01 | NA | NA | NA | NA |
| **646** | 5.90E-03 | 6.83E-03 | NA | 8.29E-01 | 9.60E-01 | NA | NA | NA | NA |
| **647** | 2.29E-03 | 1.74E-03 | NA | 8.75E-01 | 6.66E-01 | NA | NA | NA | NA |
| **648** | 1.71E-03 | 3.07E-03 | 0.00E+00 | 7.81E-01 | 1.40E+00 | (4.25e-05,0.00294] | (0.101,1.34] | NA | NA |
| **649** | 3.79E-03 | 4.54E-03 | NA | 1.00E+00 | 1.21E+00 | NA | NA | NA | NA |
| **650** | NA | NA | NA | 2.12E+00 | NA | NA | NA | NA | NA |
| **651** | 4.34E-03 | 4.38E-03 | 0.00E+00 | 9.58E-01 | 9.65E-01 | (4.25e-05,0.00294] | (0.101,1.34] | NA | NA |
| **652** | 3.22E-03 | 1.70E-03 | NA | 1.08E+00 | 5.67E-01 | NA | NA | NA | NA |
| **653** | 4.37E-03 | 7.74E-03 | NA | 9.53E-01 | 1.69E+00 | NA | NA | NA | NA |
| **654** | 2.93E-03 | 2.57E-03 | 0.00E+00 | 1.00E+00 | 8.76E-01 | (4.25e-05,0.00294] | (0.101,1.34] | NA | NA |
| **655** | 1.54E-02 | 3.96E-02 | NA | 1.02E+00 | 2.64E+00 | NA | NA | NA | NA |
| **656** | 5.70E-03 | 1.24E-03 | NA | 9.13E-01 | 1.99E-01 | NA | NA | NA | NA |
| **657** | 7.56E-03 | 2.44E-03 | NA | 1.07E+00 | 3.45E-01 | NA | NA | NA | NA |
| **658** | 6.80E-03 | 4.32E-03 | 5.73E-01 | 1.08E+00 | 6.87E-01 | (0.0348,Inf] | (14.9,Inf] | (0.0184,Inf] | (0.192,21] |
| **659** | 7.12E-03 | 2.69E-03 | NA | 7.99E-01 | 3.02E-01 | NA | NA | NA | NA |
| **660** | 3.53E-03 | 3.14E-03 | NA | 1.02E+00 | 9.10E-01 | NA | NA | NA | NA |
| **661** | NA | NA | NA | 6.73E-01 | NA | NA | NA | NA | NA |
| **662** | 5.61E-03 | 1.90E-03 | 0.00E+00 | 9.60E-01 | 3.25E-01 | (0.00294,0.0348] | (1.34,14.9] | NA | NA |
| **663** | 3.01E-03 | 3.93E-03 | NA | 9.51E-01 | 1.24E+00 | NA | NA | NA | NA |
| **664** | 1.54E-03 | 1.11E-03 | NA | 6.85E-01 | 4.92E-01 | NA | NA | NA | NA |
| **665** | 0.00E+00 | 3.34E-03 | NA | 1.43E+00 | Inf | NA | NA | NA | NA |
| **666** | 7.81E-03 | 3.49E-03 | NA | 9.82E-01 | 4.38E-01 | NA | NA | NA | NA |
| **667** | 4.90E-03 | 5.86E-03 | NA | 1.09E+00 | 1.31E+00 | NA | NA | NA | NA |
| **668** | 4.05E-03 | 4.92E-03 | 0.00E+00 | 8.70E-01 | 1.06E+00 | (0.0348,Inf] | (1.34,14.9] | NA | NA |
| **669** | 3.36E-03 | 1.12E-03 | NA | 1.22E+00 | 4.07E-01 | NA | NA | NA | NA |
| **670** | 6.24E-04 | 0.00E+00 | NA | 8.46E-01 | 0.00E+00 | NA | NA | NA | NA |
| **671** | 1.50E-03 | 1.60E-03 | NA | 7.84E-01 | 8.36E-01 | NA | NA | NA | NA |
| **672** | NA | NA | NA | 7.07E-01 | NA | NA | NA | NA | NA |
| **673** | 1.27E-02 | 8.57E-03 | 9.80E-01 | 9.02E-01 | 6.08E-01 | (0.0348,Inf] | (1.34,14.9] | (0.0184,Inf] | (0.192,21] |
| **674** | 7.34E-03 | 4.42E-03 | NA | 8.73E-01 | 5.26E-01 | NA | NA | NA | NA |
| **675** | 3.41E-03 | 2.87E-03 | NA | 6.39E-01 | 5.37E-01 | NA | NA | NA | NA |
| **676** | 2.99E-03 | 2.61E-03 | 0.00E+00 | 1.02E+00 | 8.94E-01 | (4.25e-05,0.00294] | (0,0.101] | NA | NA |
| **677** | 1.94E-03 | 1.10E-03 | NA | 8.88E-01 | 5.03E-01 | NA | NA | NA | NA |
| **678** | NA | NA | NA | 9.77E-01 | NA | NA | NA | NA | NA |
| **679** | 1.68E-03 | 4.27E-03 | NA | 7.21E-01 | 1.83E+00 | NA | NA | NA | NA |
| **680** | 5.18E-03 | 2.85E-03 | NA | 6.21E-01 | 3.42E-01 | NA | NA | NA | NA |

| | | | | | | | | | |
|---|---|---|---|---|---|---|---|---|---|
| **681** | 1.84E-03 | 0.00E+00 | NA | 1.06E+00 | 0.00E+00 | NA | NA | NA | NA |
| **682** | 1.16E-03 | 9.57E-04 | NA | 9.46E-01 | 7.84E-01 | NA | NA | NA | NA |
| **683** | 2.18E-03 | 5.98E-03 | NA | 9.00E-01 | 2.47E+00 | NA | NA | NA | NA |
| **684** | 5.89E-03 | 5.74E-03 | NA | 7.88E-01 | 7.67E-01 | NA | NA | NA | NA |
| **685** | 2.18E-03 | 2.39E-03 | NA | 8.69E-01 | 9.51E-01 | NA | NA | NA | NA |
| **686** | 8.70E-03 | 2.47E-03 | 0.00E+00 | 1.08E+00 | 3.09E-01 | (0.00294,0.0348] | (0.101,1.34] | NA | NA |
| **687** | 1.19E-02 | 7.17E-03 | NA | 9.65E-01 | 5.80E-01 | NA | NA | NA | NA |
| **688** | 3.88E-03 | 1.10E-02 | NA | 7.05E-01 | 2.00E+00 | NA | NA | NA | NA |
| **689** | 2.95E-03 | 3.77E-04 | NA | 1.24E+00 | 1.58E-01 | NA | NA | NA | NA |
| **690** | 4.32E-03 | 1.49E-03 | NA | 8.58E-01 | 2.96E-01 | NA | NA | NA | NA |
| **691** | 2.60E-03 | 5.17E-03 | 0.00E+00 | 1.33E+00 | 2.63E+00 | (0.00294,0.0348] | (1.34,14.9] | NA | NA |
| **692** | 1.13E-03 | 3.00E-03 | Inf | 9.27E-01 | 2.46E+00 | NA | NA | (0.000255,0.0184] | (0.192,21] |
| **693** | 1.43E-02 | 5.08E-03 | NA | 1.18E+00 | 4.21E-01 | NA | NA | NA | NA |
| **694** | 6.21E-03 | 6.12E-03 | 0.00E+00 | 1.15E+00 | 1.14E+00 | (0.00294,0.0348] | (0.101,1.34] | NA | NA |
| **695** | 5.32E-03 | 5.70E-03 | 0.00E+00 | 1.26E+00 | 1.35E+00 | (0.00294,0.0348] | (1.34,14.9] | NA | NA |
| **696** | 1.69E-03 | 3.10E-03 | NA | 6.58E-01 | 1.21E+00 | NA | NA | NA | NA |
| **697** | 4.24E-03 | 0.00E+00 | NA | 8.88E-01 | 0.00E+00 | NA | NA | NA | NA |
| **698** | 1.75E-03 | 1.42E-02 | 0.00E+00 | 1.23E+00 | 9.98E+00 | (0.0348,Inf] | (14.9,Inf] | NA | NA |
| **699** | 3.90E-03 | 4.20E-03 | NA | 9.95E-01 | 1.07E+00 | NA | NA | NA | NA |
| **700** | 1.70E-03 | 1.32E-03 | NA | 1.13E+00 | 8.78E-01 | NA | NA | NA | NA |
| **701** | 1.25E-03 | 2.33E-03 | NA | 1.07E+00 | 2.00E+00 | NA | NA | NA | NA |
| **702** | 5.85E-03 | 1.07E-03 | NA | 1.14E+00 | 2.07E-01 | NA | NA | NA | NA |
| **703** | 9.74E-04 | 8.86E-04 | NA | 7.93E-01 | 7.21E-01 | NA | NA | NA | NA |
| **704** | 1.33E-02 | 4.05E-03 | NA | 1.30E+00 | 3.93E-01 | NA | NA | NA | NA |
| **705** | 2.38E-03 | 1.17E-03 | 0.00E+00 | 8.74E-01 | 4.30E-01 | (4.25e-05,0.00294] | (0.101,1.34] | NA | NA |
| **706** | 5.28E-03 | 3.99E-03 | NA | 8.52E-01 | 6.43E-01 | NA | NA | NA | NA |
| **707** | 6.06E-03 | 4.63E-03 | NA | 1.08E+00 | 8.29E-01 | NA | NA | NA | NA |
| **708** | 2.02E-03 | 1.84E-03 | 0.00E+00 | 6.95E-01 | 6.34E-01 | (4.25e-05,0.00294] | (0.101,1.34] | NA | NA |
| **709** | 6.92E-03 | 4.45E-03 | 8.49E-02 | 1.15E+00 | 7.40E-01 | (0.00294,0.0348] | (0.101,1.34] | (0.000255,0.0184] | (0,0.192] |
| **710** | 6.99E-04 | 2.18E-03 | NA | 8.66E-01 | 2.70E+00 | NA | NA | NA | NA |
| **711** | 7.37E-03 | 3.46E-03 | NA | 1.11E+00 | 5.20E-01 | NA | NA | NA | NA |
| **712** | 6.63E-04 | 0.00E+00 | NA | 6.69E-01 | 0.00E+00 | NA | NA | NA | NA |
| **713** | 5.10E-03 | 6.43E-03 | NA | 7.61E-01 | 9.59E-01 | NA | NA | NA | NA |
| **714** | 1.44E-02 | 1.04E-02 | NA | 1.06E+00 | 7.62E-01 | NA | NA | NA | NA |
| **715** | 4.35E-03 | 1.92E-03 | NA | 1.05E+00 | 4.62E-01 | NA | NA | NA | NA |
| **716** | 3.98E-03 | 5.96E-03 | NA | 9.03E-01 | 1.35E+00 | NA | NA | NA | NA |
| **717** | 4.94E-03 | 1.10E-02 | 6.09E-02 | 9.96E-01 | 2.22E+00 | (0.0348,Inf] | (14.9,Inf] | (0.000255,0.0184] | (0.192,21] |
| **718** | 4.94E-03 | 1.10E-02 | 6.09E-02 | 9.96E-01 | 2.22E+00 | (0.0348,Inf] | (14.9,Inf] | (0.000255,0.0184] | (0.192,21] |

| | | | | | | | | | |
|---|---|---|---|---|---|---|---|---|---|
| **719** | 2.72E-03 | 2.29E-03 | 4.58E+01 | 9.08E-01 | 7.62E-01 | (0.00294,0.0348] | (1.34,14.9] | (0.0184,Inf] | (21,Inf] |
| **720** | 2.72E-03 | 2.29E-03 | 4.58E+01 | 9.08E-01 | 7.62E-01 | (0.00294,0.0348] | (1.34,14.9] | (0.0184,Inf] | (21,Inf] |
| **721** | 6.73E-03 | 6.43E-03 | NA | 8.30E-01 | 7.93E-01 | NA | NA | NA | NA |
| **722** | 4.77E-03 | 1.30E-03 | NA | 1.07E+00 | 2.91E-01 | NA | NA | NA | NA |
| **723** | 8.54E-03 | 2.90E-03 | 0.00E+00 | 1.18E+00 | 4.00E-01 | (0.00294,0.0348] | (0.101,1.34] | NA | NA |
| **724** | 1.17E-03 | 8.96E-04 | NA | 1.16E+00 | 8.88E-01 | NA | NA | NA | NA |
| **725** | 4.30E-03 | 1.36E-02 | 0.00E+00 | 1.65E+00 | 5.21E+00 | (0.00294,0.0348] | (1.34,14.9] | NA | NA |
| **726** | 5.87E-03 | 2.67E-03 | NA | 1.00E+00 | 4.56E-01 | NA | NA | NA | NA |
| **727** | 4.21E-03 | 3.36E-03 | NA | 1.07E+00 | 8.54E-01 | NA | NA | NA | NA |
| **728** | 3.52E-03 | 1.94E-03 | 0.00E+00 | 1.10E+00 | 6.04E-01 | (0.00294,0.0348] | (0.101,1.34] | NA | NA |
| **729** | 3.56E-03 | 1.85E-03 | NA | 8.85E-01 | 4.61E-01 | NA | NA | NA | NA |
| **730** | 4.13E-03 | 2.79E-03 | NA | 1.29E+00 | 8.69E-01 | NA | NA | NA | NA |
| **731** | 1.01E-02 | 4.43E-03 | 0.00E+00 | 1.28E+00 | 5.61E-01 | (0.00294,0.0348] | (1.34,14.9] | NA | NA |
| **732** | 3.18E-03 | 1.56E-03 | NA | 6.49E-01 | 3.20E-01 | NA | NA | NA | NA |
| **733** | NA | NA | NA | 5.43E-01 | NA | NA | NA | NA | NA |
| **734** | 1.67E-03 | 6.12E-03 | NA | 5.84E-01 | 2.14E+00 | NA | NA | NA | NA |
| **735** | 3.70E-03 | 3.99E-03 | Inf | 8.29E-01 | 8.92E-01 | NA | NA | (0.0184,Inf] | (0.192,21] |
| **736** | 8.03E-03 | 7.54E-03 | 0.00E+00 | 1.15E+00 | 1.08E+00 | (0.00294,0.0348] | (0.101,1.34] | NA | NA |
| **737** | 2.85E-03 | 1.01E-03 | NA | 1.27E+00 | 4.51E-01 | NA | NA | NA | NA |
| **738** | 3.08E-03 | 3.88E-03 | NA | 1.17E+00 | 1.48E+00 | NA | NA | NA | NA |
| **739** | 8.09E-03 | 2.88E-03 | NA | 1.07E+00 | 3.83E-01 | NA | NA | NA | NA |
| **740** | 1.69E-02 | 1.36E-03 | 0.00E+00 | 9.58E+00 | 7.72E-01 | (0.00294,0.0348] | (0.101,1.34] | NA | NA |
| **741** | 6.16E-03 | 5.43E-04 | 1.56E+00 | 1.11E+00 | 9.81E-02 | (0.00294,0.0348] | (0.101,1.34] | (0.000255,0.0184] | (0.192,21] |
| **742** | 2.90E-03 | 4.99E-03 | 0.00E+00 | 1.53E+00 | 2.63E+00 | (0.00294,0.0348] | (1.34,14.9] | NA | NA |
| **743** | NA | NA | 5.48E-01 | NA | 1.07E+00 | NA | (0.101,1.34] | NA | (0,0.192] |
| **744** | 2.23E-02 | 8.48E-03 | 6.17E-01 | 1.24E+00 | 4.73E-01 | (0.0348,Inf] | (1.34,14.9] | (0.0184,Inf] | (0.192,21] |
| **745** | 3.80E-03 | 0.00E+00 | NA | 1.21E+00 | 0.00E+00 | NA | NA | NA | NA |
| **746** | 4.06E-04 | 3.36E-03 | NA | 7.50E-01 | 6.22E+00 | NA | NA | NA | NA |
| **747** | 4.60E-03 | 9.60E-03 | 0.00E+00 | 1.41E+00 | 2.95E+00 | (0.0348,Inf] | (14.9,Inf] | NA | NA |
| **748** | 2.58E-03 | 9.71E-04 | NA | 1.21E+00 | 4.57E-01 | NA | NA | NA | NA |
| **749** | 0.00E+00 | 8.76E-04 | NA | 7.24E-01 | Inf | NA | NA | NA | NA |
| **750** | 2.40E-02 | 3.12E-02 | 0.00E+00 | 7.77E-01 | 1.01E+00 | (0.00294,0.0348] | (0.101,1.34] | NA | NA |
| **751** | 1.98E-03 | 5.47E-03 | NA | 5.42E-01 | 1.50E+00 | NA | NA | NA | NA |
| **752** | 1.42E-02 | 6.15E-03 | NA | 1.04E+00 | 4.52E-01 | NA | NA | NA | NA |
| **753** | 4.84E-03 | 2.88E-03 | NA | 9.98E-01 | 5.94E-01 | NA | NA | NA | NA |
| **754** | 7.27E-03 | 4.13E-03 | NA | 7.59E-01 | 4.31E-01 | NA | NA | NA | NA |
| **755** | 3.44E-03 | 3.36E-03 | NA | 8.87E-01 | 8.67E-01 | NA | NA | NA | NA |
| **756** | 4.10E-03 | 2.02E-03 | 1.65E+01 | 1.29E+00 | 6.35E-01 | (0.00294,0.0348] | (0.101,1.34] | (0.0184,Inf] | (21,Inf] |

| | | | | | | | | | |
|---|---|---|---|---|---|---|---|---|---|
| **757** | 3.48E-03 | 2.45E-03 | NA | 1.30E+00 | 9.17E-01 | NA | NA | NA | NA |
| **758** | 2.26E-03 | 6.56E-03 | NA | 1.00E+00 | 2.90E+00 | NA | NA | NA | NA |
| **759** | 2.46E-03 | 1.25E-03 | NA | 9.58E-01 | 4.84E-01 | NA | NA | NA | NA |
| **760** | NA | NA | NA | NA | 6.42E-01 | NA | NA | NA | NA |
| **761** | 3.06E-03 | 8.30E-03 | NA | 6.25E-01 | 1.69E+00 | NA | NA | NA | NA |
| **762** | 3.42E-03 | 7.07E-03 | 0.00E+00 | 1.28E+00 | 2.65E+00 | (0.0348,Inf] | (14.9,Inf] | NA | NA |
| **763** | 2.26E-03 | 4.36E-03 | NA | 7.27E-01 | 1.40E+00 | NA | NA | NA | NA |
| **764** | 0.00E+00 | 6.37E-04 | NA | 9.65E-01 | Inf | NA | NA | NA | NA |
| **765** | 0.00E+00 | 0.00E+00 | NA | 9.99E-01 | NA | NA | NA | NA | NA |
| **766** | 2.72E-03 | 0.00E+00 | NA | 9.72E-01 | 0.00E+00 | NA | NA | NA | NA |
| **767** | 7.51E-03 | 3.85E-03 | 7.78E-01 | 1.26E+00 | 6.47E-01 | (0.00294,0.0348] | (1.34,14.9] | (0.000255,0.0184] | (0.192,21] |
| **768** | 2.35E-03 | 5.89E-04 | NA | 9.90E-01 | 2.48E-01 | NA | NA | NA | NA |
| **769** | 8.76E-03 | 1.02E-03 | NA | 9.30E-01 | 1.08E-01 | NA | NA | NA | NA |
| **770** | 8.32E-03 | 0.00E+00 | NA | 9.29E-01 | 0.00E+00 | NA | NA | NA | NA |
| **771** | 6.08E-03 | 4.51E-03 | 1.25E+02 | 8.46E-01 | 6.28E-01 | (4.25e-05,0.00294] | (0.101,1.34] | (0.0184,Inf] | (21,Inf] |
| **772** | NA | NA | NA | 7.54E-01 | NA | NA | NA | NA | NA |
| **773** | 3.75E-03 | 4.10E-03 | NA | 8.78E-01 | 9.61E-01 | NA | NA | NA | NA |
| **774** | NA | NA | NA | 1.18E+00 | NA | NA | NA | NA | NA |
| **775** | 1.33E-02 | 4.07E-03 | NA | 9.65E-01 | 2.95E-01 | NA | NA | NA | NA |
| **776** | 5.14E-04 | 2.45E-03 | 0.00E+00 | 8.32E-01 | 3.97E+00 | (0.00294,0.0348] | (1.34,14.9] | NA | NA |
| **777** | 3.73E-03 | 9.76E-03 | 0.00E+00 | 7.71E-01 | 2.02E+00 | (0.00294,0.0348] | (1.34,14.9] | NA | NA |
| **778** | 6.22E-03 | 8.08E-03 | NA | 1.01E+00 | 1.31E+00 | NA | NA | NA | NA |
| **779** | 3.16E-03 | 3.13E-03 | NA | 6.07E-01 | 6.01E-01 | NA | NA | NA | NA |
| **780** | 2.10E-02 | 3.91E-03 | NA | 8.72E-01 | 1.62E-01 | NA | NA | NA | NA |
| **781** | 1.42E-03 | 8.37E-04 | NA | 1.20E+00 | 7.07E-01 | NA | NA | NA | NA |
| **782** | 1.43E-02 | 1.59E-02 | NA | 1.51E+00 | 1.67E+00 | NA | NA | NA | NA |
| **783** | 6.93E-04 | 2.64E-03 | 2.19E+00 | 9.64E-01 | 3.68E+00 | (0.00294,0.0348] | (1.34,14.9] | (0.000255,0.0184] | (0.192,21] |
| **784** | 3.02E-03 | 2.07E-03 | NA | 1.03E+00 | 7.03E-01 | NA | NA | NA | NA |
| **785** | 5.42E-03 | 7.41E-03 | NA | 8.44E-01 | 1.15E+00 | NA | NA | NA | NA |
| **786** | 1.14E-03 | 2.61E-03 | NA | 1.03E+00 | 2.37E+00 | NA | NA | NA | NA |
| **787** | 7.72E-03 | 7.41E-03 | NA | 7.81E-01 | 7.50E-01 | NA | NA | NA | NA |
| **788** | 4.74E-03 | 4.78E-03 | 1.13E-02 | 1.07E+00 | 1.08E+00 | (0.0348,Inf] | (1.34,14.9] | (0.000255,0.0184] | (0,0.192] |
| **789** | NA | NA | NA | 6.76E-01 | NA | NA | NA | NA | NA |
| **790** | 2.39E-03 | 2.09E-03 | NA | 7.60E-01 | 6.66E-01 | NA | NA | NA | NA |
| **791** | 2.69E-03 | 3.88E-03 | 0.00E+00 | 9.36E-01 | 1.35E+00 | (0.00294,0.0348] | (1.34,14.9] | NA | NA |
| **792** | 7.77E-03 | 3.27E-03 | NA | 9.17E-01 | 3.86E-01 | NA | NA | NA | NA |
| **793** | 6.60E-03 | 4.92E-03 | NA | 8.41E-01 | 6.27E-01 | NA | NA | NA | NA |
| **794** | 3.61E-03 | 5.11E-04 | 0.00E+00 | 8.51E-01 | 1.20E-01 | (4.25e-05,0.00294] | (0.101,1.34] | NA | NA |

| | | | | | | | | | |
|---|---|---|---|---|---|---|---|---|---|
| **795** | 1.97E-02 | 4.69E-03 | NA | 2.66E+00 | 6.32E-01 | NA | NA | NA | NA |
| **796** | 1.45E-03 | 3.02E-03 | NA | 1.14E+00 | 2.37E+00 | NA | NA | NA | NA |
| **797** | 2.61E-03 | 0.00E+00 | NA | 8.11E-01 | 0.00E+00 | NA | NA | NA | NA |
| **798** | 0.00E+00 | 0.00E+00 | NA | 9.48E-01 | NA | NA | NA | NA | NA |
| **799** | 3.07E-02 | 1.07E-02 | 0.00E+00 | 2.47E+00 | 8.61E-01 | (0.0348,Inf] | (0.101,1.34] | NA | NA |
| **800** | 3.98E-03 | 5.37E-03 | NA | 9.15E-01 | 1.24E+00 | NA | NA | NA | NA |
| **801** | 0.00E+00 | 2.05E-03 | NA | 6.46E-01 | Inf | NA | NA | NA | NA |
| **802** | NA | NA | NA | 1.43E+00 | NA | NA | NA | NA | NA |
| **803** | 3.06E-03 | 4.88E-03 | NA | 9.85E-01 | 1.57E+00 | NA | NA | NA | NA |
| **804** | 2.27E-04 | 0.00E+00 | NA | 9.18E-01 | 0.00E+00 | NA | NA | NA | NA |
| **805** | 1.27E-03 | 9.68E-03 | Inf | 7.73E-01 | 5.88E+00 | NA | NA | (0.000255,0.0184] | (0.192,21] |
| **806** | 1.76E-03 | 1.35E-03 | NA | 7.47E-01 | 5.73E-01 | NA | NA | NA | NA |
| **807** | 6.22E-04 | 1.52E-03 | NA | 9.42E-01 | 2.30E+00 | NA | NA | NA | NA |
| **808** | 3.18E-03 | 4.13E-03 | 0.00E+00 | 8.21E-01 | 1.07E+00 | (0.00294,0.0348] | (1.34,14.9] | NA | NA |
| **809** | 3.01E-03 | 0.00E+00 | NA | 7.40E-01 | 0.00E+00 | NA | NA | NA | NA |
| **810** | 2.85E-03 | 4.58E-04 | Inf | 6.58E-01 | 1.06E-01 | NA | NA | (0.0184,Inf] | (21,Inf] |
| **811** | 5.85E-03 | 8.19E-03 | NA | 8.89E-01 | 1.24E+00 | NA | NA | NA | NA |
| **812** | 4.21E-03 | 2.11E-03 | NA | 9.21E-01 | 4.62E-01 | NA | NA | NA | NA |
| **813** | 3.26E-03 | 1.14E-03 | NA | 1.48E+00 | 5.17E-01 | NA | NA | NA | NA |
| **814** | 1.71E-03 | 1.99E-03 | NA | 8.58E-01 | 9.98E-01 | NA | NA | NA | NA |
| **815** | 4.47E-03 | 2.52E-03 | 0.00E+00 | 6.32E-01 | 3.57E-01 | (0.00294,0.0348] | (1.34,14.9] | NA | NA |
| **816** | 3.39E-03 | 4.23E-03 | NA | 5.30E-01 | 6.63E-01 | NA | NA | NA | NA |
| **817** | 3.64E-03 | 3.06E-04 | NA | 1.59E+00 | 1.34E-01 | NA | NA | NA | NA |
| **818** | 1.85E-03 | 0.00E+00 | NA | 6.49E-01 | 0.00E+00 | NA | NA | NA | NA |
| **819** | 0.00E+00 | 1.01E-03 | NA | 7.11E-01 | Inf | NA | NA | NA | NA |
| **820** | 2.41E-03 | 4.37E-03 | NA | 6.84E-01 | 1.24E+00 | NA | NA | NA | NA |
| **821** | 1.71E-02 | 3.25E-02 | 0.00E+00 | 8.55E-01 | 1.63E+00 | (0.00294,0.0348] | (0.101,1.34] | NA | NA |
| **822** | 2.64E-03 | 1.48E-03 | NA | 1.04E+00 | 5.85E-01 | NA | NA | NA | NA |
| **823** | 1.79E-03 | 3.30E-03 | NA | 6.22E-01 | 1.14E+00 | NA | NA | NA | NA |
| **824** | 3.46E-04 | 0.00E+00 | NA | 6.52E-01 | 0.00E+00 | NA | NA | NA | NA |
| **825** | 0.00E+00 | 2.24E-03 | NA | 7.42E-01 | Inf | NA | NA | NA | NA |
| **826** | 1.75E-03 | 4.63E-03 | 0.00E+00 | 1.12E+00 | 2.97E+00 | (0.00294,0.0348] | (1.34,14.9] | NA | NA |
| **827** | 2.85E-03 | 3.63E-03 | NA | 8.81E-01 | 1.12E+00 | NA | NA | NA | NA |
| **828** | 4.04E-03 | 1.14E-02 | NA | 7.09E-01 | 2.00E+00 | NA | NA | NA | NA |
| **829** | 1.13E-03 | 1.96E-03 | NA | 1.06E+00 | 1.83E+00 | NA | NA | NA | NA |
| **830** | 1.55E-03 | 3.33E-03 | NA | 1.53E+00 | 3.29E+00 | NA | NA | NA | NA |
| **831** | 9.92E-03 | 7.84E-03 | 0.00E+00 | 7.94E-01 | 6.28E-01 | (0.00294,0.0348] | (0.101,1.34] | NA | NA |
| **832** | 5.01E-03 | 6.59E-03 | NA | 9.23E-01 | 1.21E+00 | NA | NA | NA | NA |

| | | | | | | | | | |
|---|---|---|---|---|---|---|---|---|---|
| **833** | 1.94E-02 | 2.77E-02 | NA | 1.07E+00 | 1.53E+00 | NA | NA | NA | NA |
| **834** | 1.94E-02 | 2.77E-02 | NA | 1.07E+00 | 1.53E+00 | NA | NA | NA | NA |
| **835** | 7.97E-03 | 1.35E-02 | NA | 6.77E-01 | 1.15E+00 | NA | NA | NA | NA |
| **836** | 3.21E-03 | 2.33E-03 | NA | 8.20E-01 | 5.94E-01 | NA | NA | NA | NA |
| **837** | 2.08E-03 | 5.24E-03 | 0.00E+00 | 7.24E-01 | 1.82E+00 | (0.00294,0.0348] | (1.34,14.9] | NA | NA |
| **838** | 3.43E-03 | 3.19E-04 | NA | 2.22E+00 | 2.06E-01 | NA | NA | NA | NA |
| **839** | NA | NA | NA | 6.36E-01 | NA | NA | NA | NA | NA |
| **840** | 7.56E-04 | 1.38E-03 | NA | 8.74E-01 | 1.59E+00 | NA | NA | NA | NA |
| **841** | 3.80E-03 | 0.00E+00 | NA | 7.88E-01 | 0.00E+00 | NA | NA | NA | NA |
| **842** | 2.89E-03 | 7.79E-03 | NA | 1.25E+00 | 3.37E+00 | NA | NA | NA | NA |
| **843** | NA | NA | NA | 9.77E-01 | NA | NA | NA | NA | NA |
| **844** | 0.00E+00 | 0.00E+00 | NA | 9.44E-01 | NA | NA | NA | NA | NA |
| **845** | 9.84E-05 | 1.66E-03 | NA | 1.24E+00 | 2.08E+01 | NA | NA | NA | NA |
| **846** | 2.48E-03 | 1.96E-03 | NA | 9.34E-01 | 7.39E-01 | NA | NA | NA | NA |
| **847** | 6.77E-03 | 4.74E-03 | NA | 9.14E-01 | 6.39E-01 | NA | NA | NA | NA |
| **848** | 2.62E-03 | 2.33E-03 | NA | 6.25E-01 | 5.56E-01 | NA | NA | NA | NA |
| **849** | 1.08E-03 | 2.29E-03 | NA | 6.21E-01 | 1.31E+00 | NA | NA | NA | NA |
| **850** | 1.08E-03 | 2.29E-03 | NA | 6.21E-01 | 1.31E+00 | NA | NA | NA | NA |
| **851** | 3.36E-03 | 6.06E-04 | NA | 7.71E-01 | 1.39E-01 | NA | NA | NA | NA |
| **852** | 2.94E-03 | 0.00E+00 | Inf | 8.50E-01 | 0.00E+00 | NA | NA | (0.000255,0.0184] | (21,Inf] |
| **853** | 2.83E-03 | 2.93E-03 | NA | 8.71E-01 | 9.05E-01 | NA | NA | NA | NA |
| **854** | 5.37E-03 | 4.93E-03 | NA | 6.40E-01 | 5.87E-01 | NA | NA | NA | NA |
| **855** | 1.69E-03 | 1.24E-03 | 0.00E+00 | 1.08E+00 | 7.90E-01 | (4.25e-05,0.00294] | (0.101,1.34] | NA | NA |
| **856** | 2.71E-03 | 3.32E-03 | NA | 6.33E-01 | 7.74E-01 | NA | NA | NA | NA |
| **857** | 7.11E-03 | 6.03E-03 | Inf | 1.12E+00 | 9.51E-01 | NA | NA | (0.000255,0.0184] | (0.192,21] |
| **858** | 1.18E-03 | 0.00E+00 | NA | 9.89E-01 | 0.00E+00 | NA | NA | NA | NA |
| **859** | 5.79E-03 | 0.00E+00 | NA | 9.64E-01 | 0.00E+00 | NA | NA | NA | NA |
| **860** | 4.84E-03 | 0.00E+00 | NA | 9.34E-01 | 0.00E+00 | NA | NA | NA | NA |
| **861** | 1.10E-02 | 4.80E-03 | NA | 9.68E-01 | 4.21E-01 | NA | NA | NA | NA |
| **862** | 4.43E-03 | 4.66E-03 | NA | 8.71E-01 | 9.17E-01 | NA | NA | NA | NA |
| **863** | 3.43E-03 | 5.54E-03 | NA | 7.06E-01 | 1.14E+00 | NA | NA | NA | NA |
| **864** | 3.59E-03 | 2.14E-03 | NA | 1.06E+00 | 6.29E-01 | NA | NA | NA | NA |
| **865** | 3.93E-03 | 1.30E-03 | Inf | 8.29E-01 | 2.75E-01 | NA | NA | (0.0184,Inf] | (21,Inf] |
| **866** | 3.43E-03 | 0.00E+00 | NA | 5.88E-01 | 0.00E+00 | NA | NA | NA | NA |
| **867** | 2.74E-03 | 0.00E+00 | NA | 1.10E+00 | 0.00E+00 | NA | NA | NA | NA |
| **868** | 4.33E-03 | 0.00E+00 | NA | 1.06E+00 | 0.00E+00 | NA | NA | NA | NA |
| **869** | 3.71E-03 | 2.97E-03 | NA | 1.09E+00 | 8.70E-01 | NA | NA | NA | NA |
| **870** | 5.04E-03 | 3.99E-03 | Inf | 7.20E-01 | 5.71E-01 | NA | NA | (0.000255,0.0184] | (0.192,21] |

| | | | | | | | | | | |
|---|---|---|---|---|---|---|---|---|---|---|
| **871** | 1.94E-03 | 4.81E-03 | NA | 8.44E-01 | 2.09E+00 | NA | NA | NA | NA |
| **872** | 1.10E-02 | 6.57E-03 | 0.00E+00 | 9.29E-01 | 5.58E-01 | (0.00294,0.0348] | (0.101,1.34] | NA | NA |
| **873** | 9.84E-03 | 4.56E-03 | 6.47E-01 | 1.60E+00 | 7.41E-01 | (0.00294,0.0348] | (1.34,14.9] | (0.000255,0.0184] | (0.192,21] |
| **874** | 1.45E-02 | 1.24E-02 | NA | 1.03E+00 | 8.78E-01 | NA | NA | NA | NA |
| **875** | 1.45E-03 | 0.00E+00 | NA | 8.71E-01 | 0.00E+00 | NA | NA | NA | NA |
| **876** | 2.89E-03 | 7.73E-03 | NA | 8.30E-01 | 2.22E+00 | NA | NA | NA | NA |
| **877** | 2.44E-03 | 2.18E-03 | NA | 5.99E-01 | 5.36E-01 | NA | NA | NA | NA |
| **878** | 1.44E-03 | 0.00E+00 | NA | 7.60E-01 | 0.00E+00 | NA | NA | NA | NA |
| **879** | 4.63E-03 | 6.56E-03 | 4.18E-01 | 1.20E+00 | 1.70E+00 | (0.00294,0.0348] | (1.34,14.9] | (0.000255,0.0184] | (0.192,21] |
| **880** | 3.08E-03 | 6.45E-03 | NA | 6.28E-01 | 1.32E+00 | NA | NA | NA | NA |
| **881** | 2.73E-03 | 1.92E-03 | NA | 1.04E+00 | 7.31E-01 | NA | NA | NA | NA |
| **882** | 6.13E-03 | 1.63E-03 | Inf | 6.99E-01 | 1.86E-01 | NA | NA | (0.000255,0.0184] | (0.192,21] |
| **883** | 0.00E+00 | 1.08E-03 | NA | 5.26E-01 | Inf | NA | NA | NA | NA |
| **884** | 4.98E-03 | 1.57E-03 | NA | 8.80E-01 | 2.77E-01 | NA | NA | NA | NA |
| **885** | 4.24E-03 | 5.22E-03 | NA | 9.26E-01 | 1.14E+00 | NA | NA | NA | NA |
| **886** | 1.77E-02 | 6.28E-03 | NA | 1.31E+00 | 4.64E-01 | NA | NA | NA | NA |
| **887** | 2.69E-04 | 3.92E-04 | NA | 1.01E+00 | 1.47E+00 | NA | NA | NA | NA |
| **888** | 3.74E-03 | 5.57E-03 | NA | 5.39E-01 | 8.03E-01 | NA | NA | NA | NA |
| **889** | 4.15E-03 | 4.24E-03 | 3.06E+02 | 9.00E-01 | 9.19E-01 | (4.25e-05,0.00294] | (0,0.101] | (0.0184,Inf] | (21,Inf] |
| **890** | NA | NA | NA | 9.03E-01 | NA | NA | NA | NA | NA |
| **891** | 4.58E-03 | 3.54E-03 | NA | 8.63E-01 | 6.68E-01 | NA | NA | NA | NA |
| **892** | NA | NA | NA | 1.32E+00 | NA | NA | NA | NA | NA |
| **893** | NA | NA | NA | 1.74E+00 | NA | NA | NA | NA | NA |
| **894** | 1.77E-02 | 1.12E-02 | NA | 1.42E+00 | 8.94E-01 | NA | NA | NA | NA |
| **895** | 6.94E-03 | 2.47E-03 | NA | 9.47E-01 | 3.37E-01 | NA | NA | NA | NA |
| **896** | 5.78E-03 | 2.96E-03 | 0.00E+00 | 8.70E-01 | 4.47E-01 | (0.00294,0.0348] | (1.34,14.9] | NA | NA |
| **897** | 8.49E-03 | 0.00E+00 | NA | 1.12E+00 | 0.00E+00 | NA | NA | NA | NA |
| **898** | 2.26E-03 | 1.83E-03 | NA | 9.25E-01 | 7.49E-01 | NA | NA | NA | NA |
| **899** | 1.41E-03 | 1.20E-03 | NA | 7.18E-01 | 6.12E-01 | NA | NA | NA | NA |
| **900** | 6.97E-04 | 1.72E-03 | NA | 9.55E-01 | 2.36E+00 | NA | NA | NA | NA |
| **901** | 1.84E-03 | 5.33E-04 | NA | 5.91E-01 | 1.72E-01 | NA | NA | NA | NA |
| **902** | 2.70E-03 | 0.00E+00 | NA | 1.06E+00 | 0.00E+00 | NA | NA | NA | NA |
| **903** | 5.77E-03 | 7.56E-03 | 0.00E+00 | 9.97E-01 | 1.31E+00 | (4.25e-05,0.00294] | (0.101,1.34] | NA | NA |
| **904** | 5.26E-04 | 5.36E-04 | NA | 8.67E-01 | 8.84E-01 | NA | NA | NA | NA |
| **905** | 8.48E-03 | 1.70E-02 | 3.03E-02 | 1.29E+00 | 2.59E+00 | (0.0348,Inf] | (14.9,Inf] | (0.000255,0.0184] | (0.192,21] |
| **906** | 7.74E-03 | 1.80E-03 | NA | 1.36E+00 | 3.16E-01 | NA | NA | NA | NA |
| **907** | 2.93E-03 | 4.53E-03 | NA | 1.32E+00 | 2.04E+00 | NA | NA | NA | NA |
| **908** | 5.17E-04 | 7.19E-04 | NA | 9.84E-01 | 1.37E+00 | NA | NA | NA | NA |

| | | | | | | | | | |
|---|---|---|---|---|---|---|---|---|---|
| **909** | 3.05E-03 | 3.11E-03 | NA | 7.32E-01 | 7.48E-01 | NA | NA | NA | NA |
| **910** | 5.63E-04 | 5.65E-03 | NA | 7.25E-01 | 7.28E+00 | NA | NA | NA | NA |
| **911** | 6.94E-03 | 8.48E-03 | NA | 7.55E-01 | 9.23E-01 | NA | NA | NA | NA |
| **912** | 9.09E-04 | 0.00E+00 | NA | 1.06E+00 | 0.00E+00 | NA | NA | NA | NA |
| **913** | 0.00E+00 | 2.81E-04 | 0.00E+00 | 1.11E+01 | Inf | (0.0348,Inf] | (14.9,Inf] | NA | NA |
| **914** | 2.23E-03 | 2.09E-03 | NA | 1.09E+00 | 1.02E+00 | NA | NA | NA | NA |
| **915** | NA | NA | 3.28E-01 | NA | 1.04E+00 | NA | (0.101,1.34] | NA | (0,0.192] |
| **916** | 1.36E-03 | 0.00E+00 | NA | 1.48E+00 | 0.00E+00 | NA | NA | NA | NA |
| **917** | 4.17E-03 | 3.29E-03 | NA | 9.13E-01 | 7.20E-01 | NA | NA | NA | NA |
| **918** | 5.03E-03 | 4.01E-03 | NA | 7.78E-01 | 6.21E-01 | NA | NA | NA | NA |
| **919** | 1.36E-03 | 0.00E+00 | 0.00E+00 | 1.93E-01 | 0.00E+00 | (4.25e-05,0.00294] | (0.101,1.34] | NA | NA |
| **920** | 2.80E-03 | 0.00E+00 | Inf | 8.35E-01 | 0.00E+00 | NA | NA | (0.0184,Inf] | (21,Inf] |
| **921** | 9.52E-03 | 8.17E-03 | NA | 8.36E-01 | 7.17E-01 | NA | NA | NA | NA |
| **922** | 9.49E-03 | 2.59E-02 | 0.00E+00 | 6.18E-01 | 1.69E+00 | (0.0348,Inf] | (1.34,14.9] | NA | NA |
| **923** | 2.89E-03 | 4.45E-03 | NA | 9.69E-01 | 1.49E+00 | NA | NA | NA | NA |
| **924** | 2.34E-03 | 1.90E-03 | NA | 1.03E+00 | 8.36E-01 | NA | NA | NA | NA |
| **925** | 1.58E-02 | 7.82E-03 | NA | 9.96E-01 | 4.94E-01 | NA | NA | NA | NA |
| **926** | 5.70E-04 | 1.37E-03 | NA | 7.38E-01 | 1.78E+00 | NA | NA | NA | NA |
| **927** | 1.50E-02 | 5.40E-03 | NA | 1.80E+00 | 6.48E-01 | NA | NA | NA | NA |
| **928** | NA | NA | NA | 8.17E-01 | NA | NA | NA | NA | NA |
| **929** | 1.65E-03 | 7.96E-03 | NA | 8.28E-01 | 3.99E+00 | NA | NA | NA | NA |
| **930** | 9.80E-03 | 6.20E-03 | NA | 8.78E-01 | 5.56E-01 | NA | NA | NA | NA |
| **931** | 4.44E-03 | 0.00E+00 | NA | 9.60E-01 | 0.00E+00 | NA | NA | NA | NA |
| **932** | 1.01E-02 | 5.20E-03 | Inf | 8.69E-01 | 4.49E-01 | NA | NA | (0.0184,Inf] | (21,Inf] |
| **933** | 1.32E-02 | 5.51E-03 | 1.47E+00 | 1.19E+00 | 4.99E-01 | (0.00294,0.0348] | (1.34,14.9] | (0.0184,Inf] | (0.192,21] |
| **934** | 5.69E-03 | 0.00E+00 | NA | 1.05E+00 | 0.00E+00 | NA | NA | NA | NA |
| **935** | 2.35E-03 | 3.75E-04 | NA | 7.15E-01 | 1.14E-01 | NA | NA | NA | NA |
| **936** | 5.47E-03 | 1.08E-03 | NA | 8.22E-01 | 1.63E-01 | NA | NA | NA | NA |
| **937** | 5.42E-03 | 4.39E-04 | 0.00E+00 | 8.88E-01 | 7.19E-02 | (0.00294,0.0348] | (1.34,14.9] | NA | NA |
| **938** | 1.52E-02 | 2.49E-03 | NA | 1.44E+00 | 2.36E-01 | NA | NA | NA | NA |
| **939** | 7.53E-03 | 0.00E+00 | NA | 8.93E-01 | 0.00E+00 | NA | NA | NA | NA |
| **940** | 5.53E-03 | 9.97E-03 | NA | 8.70E-01 | 1.57E+00 | NA | NA | NA | NA |
| **941** | 2.07E-02 | 3.38E-02 | NA | 5.25E-01 | 8.58E-01 | NA | NA | NA | NA |
| **942** | 1.15E-03 | 5.00E-03 | Inf | 6.32E-01 | 2.75E+00 | NA | NA | (0.000255,0.0184] | (0.192,21] |
| **943** | 1.33E-02 | 4.67E-03 | 5.59E-01 | 1.60E+00 | 5.64E-01 | (0.0348,Inf] | (1.34,14.9] | (0.0184,Inf] | (0.192,21] |
| **944** | 7.92E-03 | 1.01E-02 | 3.63E+00 | 1.03E+00 | 1.32E+00 | (4.25e-05,0.00294] | (0.101,1.34] | (0.000255,0.0184] | (0.192,21] |
| **945** | 3.58E-03 | 0.00E+00 | NA | 1.11E+00 | 0.00E+00 | NA | NA | NA | NA |
| **946** | 5.13E-03 | 8.36E-04 | NA | 9.12E-01 | 1.48E-01 | NA | NA | NA | NA |

| | | | | | | | | | |
|---|---|---|---|---|---|---|---|---|---|
| **947** | 5.16E-03 | 5.75E-03 | 4.53E-01 | 6.63E-01 | 7.40E-01 | (0.0348,Inf] | (1.34,14.9] | (0.0184,Inf] | (0.192,21] |
| **948** | 5.86E-03 | 3.46E-03 | NA | 6.26E-01 | 3.69E-01 | NA | NA | NA | NA |
| **949** | 1.36E-03 | 9.53E-04 | NA | 1.29E+00 | 9.07E-01 | NA | NA | NA | NA |
| **950** | 4.04E-03 | 2.33E-03 | NA | 1.46E+00 | 8.47E-01 | NA | NA | NA | NA |
| **951** | 2.58E-03 | 0.00E+00 | Inf | 8.00E-01 | 0.00E+00 | NA | NA | (0.0184,Inf] | (21,Inf] |
| **952** | 7.46E-03 | 5.05E-03 | 3.95E-02 | 8.86E-01 | 6.00E-01 | (0.0348,Inf] | (1.34,14.9] | (0.000255,0.0184] | (0.192,21] |
| **953** | 7.74E-03 | 8.24E-04 | 0.00E+00 | 1.06E+00 | 1.13E-01 | (0.00294,0.0348] | (0.101,1.34] | NA | NA |
| **954** | NA | NA | NA | 9.36E-01 | NA | NA | NA | NA | NA |
| **955** | 2.83E-03 | 8.07E-03 | 0.00E+00 | 1.04E+00 | 2.96E+00 | (0.00294,0.0348] | (1.34,14.9] | NA | NA |
| **956** | 1.23E-02 | 1.45E-02 | NA | 1.03E+00 | 1.21E+00 | NA | NA | NA | NA |
| **957** | 3.74E-03 | 1.56E-03 | NA | 1.28E+00 | 5.35E-01 | NA | NA | NA | NA |
| **958** | 2.46E-02 | 1.02E-02 | NA | 9.79E-01 | 4.04E-01 | NA | NA | NA | NA |
| **959** | 0.00E+00 | 0.00E+00 | NA | 8.68E-01 | NA | NA | NA | NA | NA |
| **960** | 1.61E-02 | 8.72E-03 | 9.14E-02 | 7.67E-01 | 4.16E-01 | (0.00294,0.0348] | (0.101,1.34] | (0.000255,0.0184] | (0,0.192] |
| **961** | 1.65E-03 | 1.66E-03 | NA | 8.32E-01 | 8.35E-01 | NA | NA | NA | NA |
| **962** | 1.40E-03 | 6.75E-03 | 0.00E+00 | 8.39E-01 | 4.05E+00 | (4.25e-05,0.00294] | (0.101,1.34] | NA | NA |
| **963** | 5.15E-03 | 1.33E-02 | NA | 9.24E-01 | 2.39E+00 | NA | NA | NA | NA |
| **964** | 1.34E-02 | 0.00E+00 | 0.00E+00 | 9.72E-01 | 0.00E+00 | (0.00294,0.0348] | (1.34,14.9] | NA | NA |
| **965** | 1.44E-02 | 2.01E-02 | NA | 7.30E-01 | 1.02E+00 | NA | NA | NA | NA |
| **966** | 1.96E-03 | 1.97E-03 | NA | 9.51E-01 | 9.52E-01 | NA | NA | NA | NA |
| **967** | 8.90E-03 | 1.10E-02 | NA | 6.43E-01 | 7.98E-01 | NA | NA | NA | NA |
| **968** | 1.25E-03 | 4.46E-03 | NA | 8.48E-01 | 3.04E+00 | NA | NA | NA | NA |
| **969** | 5.64E-03 | 3.29E-04 | NA | 9.44E-01 | 5.50E-02 | NA | NA | NA | NA |
| **970** | 4.64E-03 | 4.35E-03 | 1.34E+00 | 8.75E-01 | 8.20E-01 | (0.00294,0.0348] | (0.101,1.34] | (0.000255,0.0184] | (0.192,21] |
| **971** | NA | NA | NA | 7.77E-01 | NA | NA | NA | NA | NA |
| **972** | 5.30E-03 | 0.00E+00 | NA | 8.45E-01 | 0.00E+00 | NA | NA | NA | NA |
| **973** | 1.69E-02 | 2.80E-02 | NA | 8.16E-01 | 1.35E+00 | NA | NA | NA | NA |
| **974** | 1.69E-02 | 8.65E-03 | 8.11E-01 | 9.48E-01 | 4.84E-01 | (0.00294,0.0348] | (0.101,1.34] | (0.000255,0.0184] | (0.192,21] |
| **975** | 0.00E+00 | 7.82E-03 | NA | 7.86E-01 | Inf | NA | NA | NA | NA |
| **976** | 3.41E-03 | 5.77E-03 | NA | 8.58E-01 | 1.45E+00 | NA | NA | NA | NA |
| **977** | 2.08E-02 | 4.19E-03 | 1.41E+00 | 2.07E+00 | 4.18E-01 | (0.0348,Inf] | (1.34,14.9] | (0.0184,Inf] | (0.192,21] |
| **978** | 6.21E-03 | 4.37E-03 | NA | 7.38E-01 | 5.19E-01 | NA | NA | NA | NA |
| **979** | 1.85E-02 | 1.42E-02 | NA | 9.08E-01 | 6.97E-01 | NA | NA | NA | NA |
| **980** | 4.04E-03 | 2.78E-03 | NA | 1.23E+00 | 8.42E-01 | NA | NA | NA | NA |
| **981** | NA | NA | NA | 9.72E-01 | NA | NA | NA | NA | NA |
| **982** | 0.00E+00 | 0.00E+00 | NA | 8.54E-01 | NA | NA | NA | NA | NA |
| **983** | 5.13E-03 | 3.99E-03 | NA | 1.16E+00 | 9.05E-01 | NA | NA | NA | NA |
| **984** | 0.00E+00 | 0.00E+00 | NA | 9.32E-01 | NA | NA | NA | NA | NA |

| | | | | | | | | | |
|---|---|---|---|---|---|---|---|---|---|
| **985** | 3.31E-03 | 0.00E+00 | NA | 8.23E-01 | 0.00E+00 | NA | NA | NA | NA |
| **986** | 3.94E-03 | 3.69E-04 | 7.20E-01 | 1.02E+00 | 9.60E-02 | (0.0348,Inf] | (1.34,14.9] | (0.0184,Inf] | (21,Inf] |
| **987** | 8.47E-03 | 1.19E-03 | 0.00E+00 | 9.69E-01 | 1.36E-01 | (4.25e-05,0.00294] | (0.101,1.34] | NA | NA |
| **988** | 7.85E-04 | 1.94E-03 | NA | 6.01E-01 | 1.49E+00 | NA | NA | NA | NA |
| **989** | 3.63E-03 | 9.41E-03 | NA | 6.96E-01 | 1.80E+00 | NA | NA | NA | NA |
| **990** | 1.34E-02 | 0.00E+00 | NA | 1.59E+00 | 0.00E+00 | NA | NA | NA | NA |
| **991** | 1.57E-03 | 2.03E-02 | 5.83E-02 | 1.04E+00 | 1.35E+01 | (0.0348,Inf] | (14.9,Inf] | (0.0184,Inf] | (0.192,21] |
| **992** | 5.28E-03 | 1.05E-03 | NA | 8.44E-01 | 1.67E-01 | NA | NA | NA | NA |
| **993** | 1.18E-02 | 4.00E-03 | NA | 1.16E+00 | 3.92E-01 | NA | NA | NA | NA |
| **994** | 5.88E-04 | 4.69E-04 | NA | 7.92E-01 | 6.31E-01 | NA | NA | NA | NA |
| **995** | 0.00E+00 | 1.63E-02 | NA | 7.06E-01 | Inf | NA | NA | NA | NA |
| **996** | NA | NA | NA | 7.87E-01 | NA | NA | NA | NA | NA |
| **997** | 0.00E+00 | 3.09E-03 | NA | 1.10E+00 | Inf | NA | NA | NA | NA |
| **998** | 6.33E-03 | 4.32E-03 | NA | 8.30E-01 | 5.65E-01 | NA | NA | NA | NA |
| **999** | 4.14E-03 | 4.95E-03 | NA | 9.24E-01 | 1.10E+00 | NA | NA | NA | NA |
| **1000** | 2.40E-03 | 3.47E-03 | NA | 6.68E-01 | 9.65E-01 | NA | NA | NA | NA |
| **1001** | 0.00E+00 | 6.43E-04 | NA | 9.09E-01 | Inf | NA | NA | NA | NA |
| **1002** | 8.40E-03 | 6.50E-03 | NA | 7.95E-01 | 6.15E-01 | NA | NA | NA | NA |
| **1003** | 0.00E+00 | 3.36E-03 | NA | 7.34E-01 | Inf | NA | NA | NA | NA |
| **1004** | 5.97E-03 | 2.37E-03 | NA | 1.16E+00 | 4.61E-01 | NA | NA | NA | NA |
| **1005** | NA | NA | NA | 5.41E-01 | NA | NA | NA | NA | NA |
| **1006** | 3.04E-03 | 0.00E+00 | 0.00E+00 | 7.58E-01 | 0.00E+00 | (0.00294,0.0348] | (1.34,14.9] | NA | NA |
| **1007** | 1.19E-02 | 4.13E-03 | 3.78E+00 | 1.06E+00 | 3.66E-01 | (4.25e-05,0.00294] | (0.101,1.34] | (0.000255,0.0184] | (0.192,21] |
| **1008** | 4.37E-03 | 1.11E-03 | NA | 1.48E+00 | 3.75E-01 | NA | NA | NA | NA |
| **1009** | 2.04E-03 | 0.00E+00 | NA | 1.38E+00 | 0.00E+00 | NA | NA | NA | NA |
| **1010** | 4.46E-03 | 6.93E-03 | NA | 8.49E-01 | 1.32E+00 | NA | NA | NA | NA |
| **1011** | 8.36E-03 | 0.00E+00 | NA | 1.08E+00 | 0.00E+00 | NA | NA | NA | NA |
| **1012** | NA | NA | NA | 5.99E-01 | NA | NA | NA | NA | NA |
| **1013** | 1.21E-03 | 2.02E-03 | NA | 1.24E+00 | 2.08E+00 | NA | NA | NA | NA |
| **1014** | NA | NA | NA | 1.07E+00 | NA | NA | NA | NA | NA |
| **1015** | 1.71E-03 | 1.80E-03 | NA | 9.90E-01 | 1.04E+00 | NA | NA | NA | NA |
| **1016** | 0.00E+00 | 9.94E-04 | NA | 7.54E-01 | Inf | NA | NA | NA | NA |
| **1017** | 2.00E-03 | 0.00E+00 | NA | 6.46E-01 | 0.00E+00 | NA | NA | NA | NA |
| **1018** | 5.16E-03 | 1.67E-03 | NA | 9.98E-01 | 3.23E-01 | NA | NA | NA | NA |
| **1019** | 2.19E-03 | 7.83E-03 | 0.00E+00 | 5.17E-01 | 1.85E+00 | (0.00294,0.0348] | (1.34,14.9] | NA | NA |
| **1020** | NA | NA | NA | 5.78E-01 | NA | NA | NA | NA | NA |
| **1021** | NA | NA | NA | 6.49E-01 | NA | NA | NA | NA | NA |
| **1022** | 2.39E-03 | 2.82E-03 | NA | 7.85E-01 | 9.27E-01 | NA | NA | NA | NA |

| | | | | | | | | | |
|---|---|---|---|---|---|---|---|---|---|
| **1023** | 9.76E-03 | 1.89E-03 | NA | 9.56E-01 | 1.85E-01 | NA | NA | NA | NA |
| **1024** | 0.00E+00 | 0.00E+00 | NA | 8.19E-01 | NA | NA | NA | NA | NA |
| **1025** | 7.83E-03 | 7.02E-03 | NA | 9.56E-01 | 8.58E-01 | NA | NA | NA | NA |
| **1026** | 1.28E-02 | 3.82E-03 | NA | 8.37E-01 | 2.50E-01 | NA | NA | NA | NA |
| **1027** | 0.00E+00 | 5.20E-04 | NA | 1.05E+00 | Inf | NA | NA | NA | NA |
| **1028** | 0.00E+00 | 1.50E-03 | NA | 7.93E-01 | Inf | NA | NA | NA | NA |
| **1029** | 0.00E+00 | 0.00E+00 | NA | 1.14E+00 | NA | NA | NA | NA | NA |
| **1030** | 6.65E-03 | 4.53E-03 | 1.53E-01 | 9.92E-01 | 6.76E-01 | (0.00294,0.0348] | (1.34,14.9] | (0.000255,0.0184] | (0.192,21] |
| **1031** | 3.91E-03 | 0.00E+00 | NA | 9.90E-01 | 0.00E+00 | NA | NA | NA | NA |
| **1032** | 1.82E-02 | 7.02E-03 | NA | 1.10E+00 | 4.25E-01 | NA | NA | NA | NA |
| **1033** | 4.01E-03 | 4.75E-04 | NA | 9.46E-01 | 1.12E-01 | NA | NA | NA | NA |
| **1034** | 3.87E-03 | 1.58E-03 | NA | 8.03E-01 | 3.28E-01 | NA | NA | NA | NA |
| **1035** | 0.00E+00 | 0.00E+00 | Inf | 5.00E-01 | NA | NA | NA | (0.0184,Inf] | (21,Inf] |
| **1036** | 6.53E-03 | 4.75E-03 | 3.21E+00 | 9.63E-01 | 7.00E-01 | (0.00294,0.0348] | (1.34,14.9] | (0.0184,Inf] | (0.192,21] |
| **1037** | 2.21E-03 | 0.00E+00 | NA | 7.38E-01 | 0.00E+00 | NA | NA | NA | NA |
| **1038** | 5.46E-03 | 1.53E-02 | NA | 1.15E+00 | 3.22E+00 | NA | NA | NA | NA |
| **1039** | 4.15E-03 | 1.24E-02 | 0.00E+00 | 7.13E-01 | 2.13E+00 | (0.00294,0.0348] | (1.34,14.9] | NA | NA |
| **1040** | 0.00E+00 | 4.17E-03 | NA | 9.15E-01 | Inf | NA | NA | NA | NA |
| **1041** | 1.28E-02 | 1.82E-02 | 1.44E+00 | 9.71E-01 | 1.38E+00 | (0.0348,Inf] | (14.9,Inf] | (0.0184,Inf] | (21,Inf] |
| **1042** | 8.23E-03 | 4.55E-03 | NA | 1.28E+00 | 7.07E-01 | NA | NA | NA | NA |
| **1043** | 0.00E+00 | 1.16E-03 | NA | 8.94E-01 | Inf | NA | NA | NA | NA |
| **1044** | 1.52E-02 | 1.36E-02 | 0.00E+00 | 1.26E+00 | 1.13E+00 | (0.0348,Inf] | (1.34,14.9] | NA | NA |
| **1045** | 4.73E-03 | 4.23E-03 | NA | 9.69E-01 | 8.67E-01 | NA | NA | NA | NA |
| **1046** | 5.15E-03 | 2.02E-03 | Inf | 1.39E+00 | 5.47E-01 | NA | NA | (0.000255,0.0184] | (0.192,21] |
| **1047** | 4.31E-03 | 0.00E+00 | NA | 4.73E-01 | 0.00E+00 | NA | NA | NA | NA |
| **1048** | 0.00E+00 | 0.00E+00 | NA | 9.60E-01 | NA | NA | NA | NA | NA |
| **1049** | 1.10E-02 | 6.17E-03 | NA | 1.31E+00 | 7.34E-01 | NA | NA | NA | NA |
| **1050** | 8.69E-03 | 1.03E-03 | NA | 1.09E+00 | 1.30E-01 | NA | NA | NA | NA |
| **1051** | 2.89E-03 | 2.04E-03 | NA | 1.16E+00 | 8.22E-01 | NA | NA | NA | NA |
| **1052** | 9.85E-03 | 3.17E-02 | NA | 1.24E+00 | 3.99E+00 | NA | NA | NA | NA |
| **1053** | 1.81E-02 | 8.52E-03 | NA | 1.07E+00 | 5.06E-01 | NA | NA | NA | NA |
| **1054** | 1.38E-02 | 6.43E-04 | NA | 1.25E+00 | 5.80E-02 | NA | NA | NA | NA |
| **1055** | 1.12E-02 | 1.55E-02 | NA | 5.99E-01 | 8.28E-01 | NA | NA | NA | NA |
| **1056** | 0.00E+00 | 0.00E+00 | NA | 5.44E-01 | NA | NA | NA | NA | NA |
| **1057** | 1.44E-02 | 1.78E-03 | NA | 1.30E+00 | 1.60E-01 | NA | NA | NA | NA |
| **1058** | 0.00E+00 | 1.25E-03 | NA | 1.14E+00 | Inf | NA | NA | NA | NA |
| **1059** | 9.12E-04 | 5.94E-03 | 0.00E+00 | 1.35E+00 | 8.80E+00 | (4.25e-05,0.00294] | (1.34,14.9] | NA | NA |
| **1060** | 5.05E-03 | 2.46E-03 | NA | 8.62E-01 | 4.20E-01 | NA | NA | NA | NA |

| | | | | | | | | | |
|---|---|---|---|---|---|---|---|---|---|
| **1061** | 0.00E+00 | 0.00E+00 | 0.00E+00 | 7.33E-01 | NA | (0.00294,0.0348] | (14.9,Inf] | NA | NA |
| **1062** | 4.44E-03 | 6.31E-03 | 0.00E+00 | 5.01E-01 | 7.12E-01 | (4.25e-05,0.00294] | (0.101,1.34] | NA | NA |
| **1063** | 2.98E-03 | 1.33E-03 | NA | 8.90E-01 | 3.96E-01 | NA | NA | NA | NA |
| **1064** | 1.40E-02 | 1.84E-03 | 2.85E+02 | 1.39E+00 | 1.84E-01 | (0.00294,0.0348] | (0.101,1.34] | (0.0184,Inf] | (21,Inf] |
| **1065** | NA | NA | NA | 6.47E-01 | NA | NA | NA | NA | NA |
| **1066** | 6.65E-03 | 5.32E-03 | NA | 7.66E-01 | 6.13E-01 | NA | NA | NA | NA |
| **1067** | 1.12E-02 | 1.05E-02 | NA | 1.94E+00 | 1.81E+00 | NA | NA | NA | NA |
| **1068** | 1.14E-02 | 6.65E-03 | NA | 8.77E-01 | 5.13E-01 | NA | NA | NA | NA |
| **1069** | 4.32E-03 | 0.00E+00 | NA | 7.42E-01 | 0.00E+00 | NA | NA | NA | NA |
| **1070** | 8.35E-03 | 7.72E-03 | NA | 9.78E-01 | 9.04E-01 | NA | NA | NA | NA |
| **1071** | NA | NA | NA | 7.65E-01 | NA | NA | NA | NA | NA |
| **1072** | 0.00E+00 | 0.00E+00 | NA | 1.11E+00 | NA | NA | NA | NA | NA |
| **1073** | NA | NA | NA | 6.28E-01 | NA | NA | NA | NA | NA |
| **1074** | 2.86E-03 | 1.06E-03 | NA | 7.40E-01 | 2.75E-01 | NA | NA | NA | NA |
| **1075** | 1.33E-02 | 2.05E-02 | NA | 7.11E-01 | 1.10E+00 | NA | NA | NA | NA |
| **1076** | 6.58E-03 | 1.49E-03 | NA | 8.92E-01 | 2.03E-01 | NA | NA | NA | NA |
| **1077** | NA | NA | NA | 1.54E+00 | NA | NA | NA | NA | NA |
| **1078** | 0.00E+00 | 6.32E-03 | NA | 1.01E+00 | Inf | NA | NA | NA | NA |
| **1079** | NA | NA | NA | 7.46E-01 | NA | NA | NA | NA | NA |
| **1080** | 8.02E-03 | 0.00E+00 | NA | 1.73E+00 | 0.00E+00 | NA | NA | NA | NA |
| **1081** | 1.92E-03 | 0.00E+00 | NA | 6.92E-01 | 0.00E+00 | NA | NA | NA | NA |
| **1082** | 1.94E-03 | 7.78E-03 | NA | 4.24E-01 | 1.70E+00 | NA | NA | NA | NA |
| **1083** | 3.64E-03 | 0.00E+00 | NA | 6.19E-01 | 0.00E+00 | NA | NA | NA | NA |
| **1084** | NA | NA | NA | 1.18E+00 | NA | NA | NA | NA | NA |
| **1085** | 3.08E-03 | 7.60E-03 | 0.00E+00 | 9.59E-01 | 2.36E+00 | (0.0348,Inf] | (14.9,Inf] | NA | NA |
| **1086** | 1.30E-02 | 1.67E-02 | 2.21E-01 | 9.39E-01 | 1.21E+00 | (0.00294,0.0348] | (1.34,14.9] | (0.000255,0.0184] | (0.192,21] |
| **1087** | 9.14E-04 | 1.90E-03 | NA | 1.19E+00 | 2.46E+00 | NA | NA | NA | NA |
| **1088** | 1.36E-02 | 4.20E-03 | NA | 9.44E-01 | 2.93E-01 | NA | NA | NA | NA |
| **1089** | 1.37E-02 | 1.40E-02 | 1.83E-01 | 1.42E+00 | 1.45E+00 | (0.0348,Inf] | (1.34,14.9] | (0.0184,Inf] | (0.192,21] |
| **1090** | 2.90E-03 | 1.16E-02 | 0.00E+00 | 9.75E-01 | 3.89E+00 | (0.00294,0.0348] | (1.34,14.9] | NA | NA |
| **1091** | 1.15E-02 | 8.89E-03 | NA | 7.44E-01 | 5.74E-01 | NA | NA | NA | NA |
| **1092** | 6.73E-04 | 0.00E+00 | NA | 4.56E-01 | 0.00E+00 | NA | NA | NA | NA |
| **1093** | 1.26E-02 | 1.06E-02 | NA | 9.31E-01 | 7.81E-01 | NA | NA | NA | NA |
| **1094** | 8.71E-03 | 0.00E+00 | NA | 1.05E+00 | 0.00E+00 | NA | NA | NA | NA |
| **1095** | 0.00E+00 | 3.10E-03 | NA | 1.13E+00 | Inf | NA | NA | NA | NA |
| **1096** | 1.05E-03 | 0.00E+00 | NA | 6.50E-01 | 0.00E+00 | NA | NA | NA | NA |
| **1097** | 1.21E-02 | 1.48E-03 | Inf | 7.00E-01 | 8.56E-02 | NA | NA | (0.0184,Inf] | (21,Inf] |
| **1098** | 6.22E-03 | 1.08E-03 | NA | 8.39E-01 | 1.46E-01 | NA | NA | NA | NA |

| | | | | | | | | | |
|---|---|---|---|---|---|---|---|---|---|
| **1099** | 1.69E-02 | 6.77E-03 | Inf | 2.49E+00 | 9.99E-01 | NA | NA | (0.0184,Inf] | (21,Inf] |
| **1100** | 0.00E+00 | 1.16E-02 | NA | 6.45E-01 | Inf | NA | NA | NA | NA |
| **1101** | 1.35E-02 | 1.09E-02 | 0.00E+00 | 9.98E-01 | 8.07E-01 | (0.0348,Inf] | (1.34,14.9] | NA | NA |
| **1102** | 8.77E-03 | 5.07E-03 | NA | 6.21E-01 | 3.59E-01 | NA | NA | NA | NA |
| **1103** | 1.12E-02 | 0.00E+00 | NA | 1.18E+00 | 0.00E+00 | NA | NA | NA | NA |
| **1104** | 1.38E-02 | 2.76E-03 | NA | 8.48E-01 | 1.69E-01 | NA | NA | NA | NA |
| **1105** | 6.16E-03 | 2.44E-03 | NA | 1.03E+00 | 4.10E-01 | NA | NA | NA | NA |
| **1106** | 5.04E-03 | 4.27E-03 | NA | 9.69E-01 | 8.22E-01 | NA | NA | NA | NA |
| **1107** | 5.39E-03 | 1.02E-02 | NA | 9.20E-01 | 1.74E+00 | NA | NA | NA | NA |
| **1108** | 9.16E-02 | 1.44E-02 | NA | 3.32E+00 | 5.23E-01 | NA | NA | NA | NA |
| **1109** | 6.53E-03 | 0.00E+00 | NA | 9.71E-01 | 0.00E+00 | NA | NA | NA | NA |
| **1110** | 4.84E-02 | 1.85E-02 | NA | 1.70E+00 | 6.51E-01 | NA | NA | NA | NA |
| **1111** | 1.58E-02 | 0.00E+00 | NA | 9.44E-01 | 0.00E+00 | NA | NA | NA | NA |
| **1112** | 6.06E-04 | 1.24E-03 | NA | 1.27E+00 | 2.60E+00 | NA | NA | NA | NA |
| **1113** | 0.00E+00 | 4.04E-04 | NA | 9.95E-01 | Inf | NA | NA | NA | NA |
| **1114** | 1.82E-03 | 9.62E-03 | NA | 7.84E-01 | 4.14E+00 | NA | NA | NA | NA |
| **1115** | 0.00E+00 | 4.69E-03 | NA | 4.98E-01 | Inf | NA | NA | NA | NA |
| **1116** | 1.51E-03 | 8.76E-04 | NA | 6.64E-01 | 3.84E-01 | NA | NA | NA | NA |
| **1117** | 3.24E-02 | 0.00E+00 | NA | 8.14E-01 | 0.00E+00 | NA | NA | NA | NA |
| **1118** | 0.00E+00 | 6.68E-04 | NA | 7.59E-01 | Inf | NA | NA | NA | NA |
| **1119** | 9.44E-03 | 6.90E-04 | NA | 8.04E-01 | 5.88E-02 | NA | NA | NA | NA |
| **1120** | 0.00E+00 | 3.76E-02 | 0.00E+00 | 1.22E+00 | Inf | (0.0348,Inf] | (14.9,Inf] | NA | NA |
| **1121** | 1.55E-02 | 9.52E-03 | 9.43E-01 | 9.15E-01 | 5.62E-01 | (0.0348,Inf] | (1.34,14.9] | (0.0184,Inf] | (0.192,21] |
| **1122** | 1.69E-03 | 3.78E-03 | NA | 7.08E-01 | 1.58E+00 | NA | NA | NA | NA |
| **1123** | 9.57E-03 | 3.54E-03 | NA | 1.99E-01 | 7.36E-02 | NA | NA | NA | NA |
| **1124** | NA | NA | NA | 8.71E-01 | NA | NA | NA | NA | NA |
| **1125** | 1.99E-02 | 7.03E-03 | NA | 1.19E+00 | 4.21E-01 | NA | NA | NA | NA |
| **1126** | 1.42E-02 | 1.06E-02 | NA | 1.78E+00 | 1.33E+00 | NA | NA | NA | NA |
| **1127** | NA | NA | 3.57E-01 | NA | 9.01E-01 | NA | (0.101,1.34] | NA | (0,0.192] |
| **1128** | 2.40E-03 | 0.00E+00 | NA | 3.86E-01 | 0.00E+00 | NA | NA | NA | NA |
| **1129** | 1.42E-02 | 1.88E-03 | 1.24E+00 | 1.42E+00 | 1.88E-01 | (0.00294,0.0348] | (1.34,14.9] | (0.0184,Inf] | (0.192,21] |
| **1130** | NA | NA | NA | 9.24E-01 | NA | NA | NA | NA | NA |
| **1131** | 6.22E-03 | 7.73E-03 | 0.00E+00 | 1.40E+00 | 1.75E+00 | (0.0348,Inf] | (1.34,14.9] | NA | NA |
| **1132** | 9.85E-03 | 4.20E-03 | NA | 1.13E+00 | 4.84E-01 | NA | NA | NA | NA |
| **1133** | 3.37E-03 | 0.00E+00 | NA | 9.86E-01 | 0.00E+00 | NA | NA | NA | NA |
| **1134** | NA | NA | NA | 9.02E-01 | NA | NA | NA | NA | NA |
| **1135** | 1.64E-02 | 1.24E-02 | NA | 8.32E-01 | 6.31E-01 | NA | NA | NA | NA |
| **1136** | 7.48E-04 | 5.16E-03 | NA | 1.13E+00 | 7.78E+00 | NA | NA | NA | NA |

| | | | | | | | | | |
|---|---|---|---|---|---|---|---|---|---|
| **1137** | 0.00E+00 | 1.78E-03 | NA | 9.56E-01 | Inf | NA | NA | NA | NA |
| **1138** | 7.62E-03 | 1.52E-03 | NA | 5.95E-01 | 1.19E-01 | NA | NA | NA | NA |
| **1139** | 3.85E-03 | 4.54E-03 | NA | 1.43E+00 | 1.68E+00 | NA | NA | NA | NA |
| **1140** | 0.00E+00 | 5.59E-03 | NA | 4.55E-01 | Inf | NA | NA | NA | NA |
| **1141** | 9.58E-03 | 7.17E-03 | NA | 6.26E-01 | 4.68E-01 | NA | NA | NA | NA |
| **1142** | 3.50E-03 | 0.00E+00 | NA | 7.05E-01 | 0.00E+00 | NA | NA | NA | NA |
| **1143** | 1.04E-02 | 1.05E-02 | NA | 1.95E+00 | 1.98E+00 | NA | NA | NA | NA |
| **1144** | NA | NA | NA | 1.44E+00 | NA | NA | NA | NA | NA |
| **1145** | 1.54E-02 | 1.40E-02 | 1.11E-01 | 9.00E-01 | 8.19E-01 | (0.0348,Inf] | (14.9,Inf] | (0.0184,Inf] | (0.192,21] |
| **1146** | 8.53E-03 | 2.80E-03 | NA | 9.60E-01 | 3.15E-01 | NA | NA | NA | NA |
| **1147** | 7.53E-03 | 7.52E-03 | NA | 8.35E-01 | 8.34E-01 | NA | NA | NA | NA |
| **1148** | 2.25E-02 | 1.11E-02 | 8.24E-01 | 1.11E+00 | 5.47E-01 | (0.0348,Inf] | (1.34,14.9] | (0.0184,Inf] | (0.192,21] |
| **1149** | 0.00E+00 | 2.32E-03 | NA | 7.51E-01 | Inf | NA | NA | NA | NA |
| **1150** | 6.37E-03 | 1.73E-03 | NA | 6.20E-01 | 1.69E-01 | NA | NA | NA | NA |
| **1151** | 2.50E-04 | 9.79E-04 | NA | 4.44E-01 | 1.74E+00 | NA | NA | NA | NA |
| **1152** | 6.08E-03 | 4.36E-03 | NA | 6.79E-01 | 4.88E-01 | NA | NA | NA | NA |
| **1153** | 8.59E-03 | 0.00E+00 | NA | 8.31E-01 | 0.00E+00 | NA | NA | NA | NA |
| **1154** | NA | NA | NA | 9.59E-01 | NA | NA | NA | NA | NA |
| **1155** | 3.78E-03 | 8.16E-03 | NA | 7.41E-01 | 1.60E+00 | NA | NA | NA | NA |
| **1156** | 0.00E+00 | 8.25E-03 | 0.00E+00 | 3.83E-01 | Inf | (4.25e-05,0.00294] | (14.9,Inf] | NA | NA |
| **1157** | 6.16E-03 | 0.00E+00 | NA | 8.55E-01 | 0.00E+00 | NA | NA | NA | NA |
| **1158** | NA | NA | NA | 9.59E-01 | NA | NA | NA | NA | NA |
| **1159** | 0.00E+00 | 6.56E-03 | NA | 9.82E-01 | Inf | NA | NA | NA | NA |
| **1160** | 1.13E-02 | 0.00E+00 | NA | 1.11E+00 | 0.00E+00 | NA | NA | NA | NA |
| **1161** | 0.00E+00 | 1.53E-02 | NA | 3.28E-01 | Inf | NA | NA | NA | NA |
| **1162** | NA | NA | NA | 1.05E+00 | NA | NA | NA | NA | NA |
| **1163** | 5.20E-03 | 4.85E-03 | NA | 6.94E-01 | 6.47E-01 | NA | NA | NA | NA |
| **1164** | 2.93E-03 | 0.00E+00 | NA | 6.56E-01 | 0.00E+00 | NA | NA | NA | NA |
| **1165** | 8.50E-03 | 8.35E-03 | Inf | 8.61E-01 | 8.46E-01 | NA | NA | (0.0184,Inf] | (0.192,21] |
| **1166** | 3.57E-03 | 0.00E+00 | NA | 7.86E-01 | 0.00E+00 | NA | NA | NA | NA |
| **1167** | 0.00E+00 | 0.00E+00 | NA | 7.18E-01 | NA | NA | NA | NA | NA |
| **1168** | 2.48E-02 | 0.00E+00 | NA | 1.23E+00 | 0.00E+00 | NA | NA | NA | NA |
| **1169** | 9.66E-04 | 1.21E-03 | NA | 7.73E-01 | 9.66E-01 | NA | NA | NA | NA |
| **1170** | 6.60E-03 | 9.31E-03 | 2.32E+02 | 7.19E-01 | 1.01E+00 | (4.25e-05,0.00294] | (0.101,1.34] | (0.0184,Inf] | (21,Inf] |
| **1171** | 0.00E+00 | 0.00E+00 | Inf | 9.65E-01 | NA | NA | NA | (0.0184,Inf] | (21,Inf] |
| **1172** | 1.08E-02 | 1.26E-02 | 0.00E+00 | 7.46E-01 | 8.75E-01 | (0.0348,Inf] | (1.34,14.9] | NA | NA |
| **1173** | 3.71E-03 | 2.15E-03 | NA | 5.75E-01 | 3.33E-01 | NA | NA | NA | NA |
| **1174** | 8.43E-03 | 5.24E-03 | NA | 1.20E+00 | 7.48E-01 | NA | NA | NA | NA |

| | | | | | | | | | |
|---|---|---|---|---|---|---|---|---|---|
| **1175** | NA | NA | NA | 4.42E-01 | NA | NA | NA | NA | NA |
| **1176** | 2.91E-03 | 0.00E+00 | NA | 5.09E-01 | 0.00E+00 | NA | NA | NA | NA |
| **1177** | 1.17E-02 | 0.00E+00 | NA | 1.32E+00 | 0.00E+00 | NA | NA | NA | NA |
| **1178** | 3.85E-03 | 2.77E-03 | 0.00E+00 | 9.70E-01 | 6.98E-01 | (0.00294,0.0348] | (1.34,14.9] | NA | NA |
| **1179** | NA | NA | NA | 9.62E-01 | NA | NA | NA | NA | NA |
| **1180** | 2.77E-03 | 2.89E-02 | NA | 5.97E-01 | 6.23E+00 | NA | NA | NA | NA |
| **1181** | 1.23E-03 | 0.00E+00 | NA | 7.46E-01 | 0.00E+00 | NA | NA | NA | NA |
| **1182** | 8.56E-03 | 9.67E-03 | NA | 1.15E+00 | 1.30E+00 | NA | NA | NA | NA |
| **1183** | 0.00E+00 | 0.00E+00 | NA | 1.04E+00 | NA | NA | NA | NA | NA |
| **1184** | 7.45E-03 | 1.62E-03 | 1.85E+00 | 5.99E-01 | 1.30E-01 | (0.0348,Inf] | (1.34,14.9] | (0.0184,Inf] | (21,Inf] |
| **1185** | NA | NA | NA | 7.03E-01 | NA | NA | NA | NA | NA |
| **1186** | 5.31E-03 | 5.98E-03 | NA | 1.05E+00 | 1.18E+00 | NA | NA | NA | NA |
| **1187** | 2.11E-02 | 1.31E-02 | NA | 6.93E-01 | 4.30E-01 | NA | NA | NA | NA |
| **1188** | 2.54E-03 | 5.07E-03 | NA | 5.74E-01 | 1.15E+00 | NA | NA | NA | NA |
| **1189** | 0.00E+00 | 0.00E+00 | NA | 7.11E-01 | NA | NA | NA | NA | NA |
| **1190** | 1.55E-02 | 0.00E+00 | NA | 6.90E-01 | 0.00E+00 | NA | NA | NA | NA |
| **1191** | 0.00E+00 | 1.33E-03 | NA | 8.07E-01 | Inf | NA | NA | NA | NA |
| **1192** | NA | NA | NA | 5.92E-01 | NA | NA | NA | NA | NA |
| **1193** | 1.65E-02 | 1.42E-03 | NA | 6.98E-01 | 6.02E-02 | NA | NA | NA | NA |
| **1194** | 4.83E-03 | 1.35E-02 | NA | 6.66E-01 | 1.87E+00 | NA | NA | NA | NA |
| **1195** | 0.00E+00 | 1.82E-03 | NA | 9.46E-01 | Inf | NA | NA | NA | NA |
| **1196** | 0.00E+00 | 0.00E+00 | NA | 1.10E+00 | NA | NA | NA | NA | NA |
| **1197** | 1.12E-02 | 9.70E-04 | NA | 9.54E-01 | 8.25E-02 | NA | NA | NA | NA |
| **1198** | NA | NA | NA | NA | 5.80E-01 | NA | NA | NA | NA |
| **1199** | 1.82E-03 | 7.68E-03 | NA | 8.87E-01 | 3.74E+00 | NA | NA | NA | NA |
| **1200** | 3.90E-02 | 8.55E-03 | NA | 1.17E+00 | 2.56E-01 | NA | NA | NA | NA |
| **1201** | 0.00E+00 | 2.32E-02 | NA | 7.76E-01 | Inf | NA | NA | NA | NA |
| **1202** | 3.39E-02 | 3.85E-03 | NA | 3.04E+00 | 3.46E-01 | NA | NA | NA | NA |
| **1203** | NA | NA | NA | 1.64E+00 | NA | NA | NA | NA | NA |
| **1204** | 1.02E-02 | 6.23E-03 | Inf | 6.69E-01 | 4.07E-01 | NA | NA | (0.000255,0.0184] | (0.192,21] |
| **1205** | NA | NA | NA | 8.35E-01 | NA | NA | NA | NA | NA |
| **1206** | 1.11E-02 | 1.45E-02 | 5.18E-02 | 1.62E+00 | 2.12E+00 | (0.0348,Inf] | (14.9,Inf] | (0.0184,Inf] | (0.192,21] |
| **1207** | 1.38E-02 | 1.32E-02 | NA | 5.06E-01 | 4.84E-01 | NA | NA | NA | NA |
| **1208** | 1.35E-02 | 1.42E-02 | NA | 1.26E+00 | 1.32E+00 | NA | NA | NA | NA |
| **1209** | 6.13E-02 | 1.10E-02 | NA | 1.32E+00 | 2.37E-01 | NA | NA | NA | NA |
| **1210** | NA | NA | NA | 7.85E-01 | NA | NA | NA | NA | NA |
| **1211** | NA | NA | NA | 8.38E-01 | NA | NA | NA | NA | NA |
| **1212** | 1.90E-03 | 1.36E-03 | NA | 6.90E-01 | 4.92E-01 | NA | NA | NA | NA |

| | | | | | | | | | |
|---|---|---|---|---|---|---|---|---|---|
| **1213** | 9.11E-03 | 1.72E-02 | 0.00E+00 | 9.81E-01 | 1.85E+00 | (0.0348,Inf] | (14.9,Inf] | NA | NA |
| **1214** | 8.48E-04 | 0.00E+00 | NA | 2.54E-01 | 0.00E+00 | NA | NA | NA | NA |
| **1215** | 1.94E-02 | 1.01E-02 | NA | 4.74E-01 | 2.48E-01 | NA | NA | NA | NA |
| **1216** | 9.40E-03 | 0.00E+00 | NA | 6.42E-01 | 0.00E+00 | NA | NA | NA | NA |
| **1217** | NA | NA | NA | 6.66E-01 | NA | NA | NA | NA | NA |
| **1218** | 4.42E-02 | 6.14E-03 | NA | 1.50E+00 | 2.09E-01 | NA | NA | NA | NA |
| **1219** | 2.68E-03 | 3.43E-03 | NA | 9.47E-01 | 1.21E+00 | NA | NA | NA | NA |
| **1220** | 9.29E-03 | 0.00E+00 | NA | 8.16E-01 | 0.00E+00 | NA | NA | NA | NA |
| **1221** | 8.43E-03 | 1.68E-02 | NA | 3.97E-01 | 7.90E-01 | NA | NA | NA | NA |
| **1222** | 4.77E-03 | 3.36E-03 | 0.00E+00 | 1.12E+00 | 7.89E-01 | (0.00294,0.0348] | (1.34,14.9] | NA | NA |
| **1223** | 7.46E-03 | 3.28E-02 | NA | 7.19E-01 | 3.16E+00 | NA | NA | NA | NA |
| **1224** | 1.70E-02 | 9.67E-03 | NA | 1.11E+00 | 6.32E-01 | NA | NA | NA | NA |
| **1225** | 5.64E-03 | 0.00E+00 | NA | 9.38E-01 | 0.00E+00 | NA | NA | NA | NA |
| **1226** | 8.53E-03 | 0.00E+00 | NA | 2.28E+00 | 0.00E+00 | NA | NA | NA | NA |
| **1227** | NA | NA | NA | NA | 9.37E-01 | NA | NA | NA | NA |
| **1228** | NA | NA | NA | 5.64E-01 | NA | NA | NA | NA | NA |
| **1229** | 2.44E-03 | 0.00E+00 | NA | 1.05E+00 | 0.00E+00 | NA | NA | NA | NA |
| **1230** | 0.00E+00 | 1.42E-03 | NA | 1.43E+00 | Inf | NA | NA | NA | NA |
| **1231** | 8.42E-03 | 2.18E-03 | NA | 1.01E+00 | 2.60E-01 | NA | NA | NA | NA |
| **1232** | NA | NA | NA | 7.90E-01 | NA | NA | NA | NA | NA |
| **1233** | NA | NA | NA | 8.87E-01 | NA | NA | NA | NA | NA |
| **1234** | NA | NA | NA | 1.25E+00 | NA | NA | NA | NA | NA |
| **1235** | 7.77E-03 | 0.00E+00 | NA | 8.06E-01 | 0.00E+00 | NA | NA | NA | NA |
| **1236** | 0.00E+00 | 2.24E-03 | NA | 6.33E-01 | Inf | NA | NA | NA | NA |
| **1237** | 1.83E-02 | 0.00E+00 | NA | 1.24E+00 | 0.00E+00 | NA | NA | NA | NA |
| **1238** | 5.92E-03 | 3.45E-03 | NA | 1.31E+00 | 7.66E-01 | NA | NA | NA | NA |
| **1239** | 6.47E-03 | 0.00E+00 | NA | 7.59E-01 | 0.00E+00 | NA | NA | NA | NA |
| **1240** | 2.48E-03 | 2.61E-03 | 0.00E+00 | 5.47E-01 | 5.74E-01 | (0.00294,0.0348] | (1.34,14.9] | NA | NA |
| **1241** | 1.14E-02 | 1.38E-02 | 0.00E+00 | 9.42E-01 | 1.14E+00 | (0.00294,0.0348] | (0.101,1.34] | NA | NA |
| **1242** | 1.09E-02 | 8.54E-02 | NA | 9.51E-01 | 7.44E+00 | NA | NA | NA | NA |
| **1243** | 8.71E-03 | 5.77E-03 | NA | 1.09E+00 | 7.21E-01 | NA | NA | NA | NA |
| **1244** | 4.50E-03 | 0.00E+00 | NA | 8.56E-01 | 0.00E+00 | NA | NA | NA | NA |
| **1245** | 6.21E-03 | 4.22E-03 | NA | 6.67E-01 | 4.53E-01 | NA | NA | NA | NA |
| **1246** | 4.91E-03 | 4.14E-03 | NA | 1.20E+00 | 1.01E+00 | NA | NA | NA | NA |
| **1247** | 1.41E-03 | 0.00E+00 | NA | 1.09E+00 | 0.00E+00 | NA | NA | NA | NA |
| **1248** | 4.08E-04 | 5.70E-03 | NA | 2.07E-01 | 2.90E+00 | NA | NA | NA | NA |
| **1249** | NA | NA | NA | 6.74E-01 | NA | NA | NA | NA | NA |
| **1250** | NA | NA | NA | 3.27E-01 | NA | NA | NA | NA | NA |

| | | | | | | | | | | |
|---|---|---|---|---|---|---|---|---|---|---|
| **1251** | 3.30E-03 | 1.43E-03 | NA | 6.06E-01 | 2.62E-01 | NA | NA | NA | NA |
| **1252** | NA | NA | NA | 8.58E-01 | NA | NA | NA | NA | NA |
| **1253** | 8.31E-03 | 2.68E-03 | 0.00E+00 | 8.93E-01 | 2.88E-01 | (0.00294,0.0348] | (1.34,14.9] | NA | NA |
| **1254** | 3.41E-03 | 9.25E-04 | 3.46E+01 | 9.70E-01 | 2.63E-01 | (4.25e-05,0.00294] | (0.101,1.34] | (0.0184,Inf] | (21,Inf] |
| **1255** | 2.77E-03 | 2.02E-03 | Inf | 1.30E+00 | 9.49E-01 | NA | NA | (0.000255,0.0184] | (0.192,21] |
| **1256** | 2.95E-03 | 1.48E-02 | Inf | 5.48E-01 | 2.75E+00 | NA | NA | (0.0184,Inf] | (0.192,21] |
| **1257** | 5.57E-03 | 1.02E-02 | NA | 1.21E+00 | 2.21E+00 | NA | NA | NA | NA |
| **1258** | NA | NA | NA | 9.29E-01 | NA | NA | NA | NA | NA |
| **1259** | 4.60E-03 | 0.00E+00 | NA | 1.70E-01 | 0.00E+00 | NA | NA | NA | NA |
| **1260** | 4.11E-03 | 3.44E-03 | NA | 6.77E-01 | 5.67E-01 | NA | NA | NA | NA |
| **1261** | 6.48E-03 | 2.95E-02 | 0.00E+00 | 6.02E-01 | 2.74E+00 | (0.0348,Inf] | (14.9,Inf] | NA | NA |
| **1262** | 1.17E-02 | 1.66E-03 | NA | 1.46E+00 | 2.08E-01 | NA | NA | NA | NA |
| **1263** | 7.01E-03 | 0.00E+00 | NA | 8.36E-01 | 0.00E+00 | NA | NA | NA | NA |
| **1264** | NA | NA | NA | 8.29E-01 | NA | NA | NA | NA | NA |
| **1265** | 2.48E-03 | 7.59E-03 | NA | 5.18E-01 | 1.59E+00 | NA | NA | NA | NA |
| **1266** | 3.62E-03 | 2.48E-03 | NA | 8.78E-01 | 6.02E-01 | NA | NA | NA | NA |
| **1267** | 4.97E-04 | 1.12E-03 | NA | 4.33E-01 | 9.80E-01 | NA | NA | NA | NA |
| **1268** | 6.21E-03 | 8.89E-03 | NA | 1.12E-01 | 1.60E-01 | NA | NA | NA | NA |
| **1269** | 4.33E-03 | 3.02E-03 | NA | 4.01E-01 | 2.79E-01 | NA | NA | NA | NA |
| **1270** | 1.35E-02 | 0.00E+00 | NA | 1.32E+00 | 0.00E+00 | NA | NA | NA | NA |
| **1271** | 3.78E-03 | 4.39E-03 | NA | 6.01E-01 | 6.97E-01 | NA | NA | NA | NA |
| **1272** | 3.16E-02 | 7.99E-03 | NA | 1.02E+00 | 2.57E-01 | NA | NA | NA | NA |
| **1273** | 6.31E-03 | 1.90E-02 | NA | 4.31E-01 | 1.30E+00 | NA | NA | NA | NA |
| **1274** | 6.25E-03 | 1.01E-02 | 7.18E+00 | 4.45E-01 | 7.22E-01 | (0.00294,0.0348] | (1.34,14.9] | (0.0184,Inf] | (21,Inf] |
| **1275** | NA | NA | NA | 8.39E-01 | NA | NA | NA | NA | NA |
| **1276** | 2.74E-02 | 2.28E-02 | NA | 8.72E-01 | 7.25E-01 | NA | NA | NA | NA |
| **1277** | 1.89E-02 | 0.00E+00 | NA | 5.59E-01 | 0.00E+00 | NA | NA | NA | NA |
| **1278** | NA | NA | 0.00E+00 | 2.83E+00 | NA | (0.0348,Inf] | NA | NA | NA |
| **1279** | 4.16E-03 | 4.91E-03 | NA | 3.84E-01 | 4.54E-01 | NA | NA | NA | NA |
| **1280** | 9.20E-04 | 1.16E-02 | NA | 1.24E+00 | 1.56E+01 | NA | NA | NA | NA |
| **1281** | 6.57E-03 | 0.00E+00 | NA | 8.64E-01 | 0.00E+00 | NA | NA | NA | NA |
| **1282** | 0.00E+00 | 6.55E-04 | NA | 9.46E-01 | Inf | NA | NA | NA | NA |
| **1283** | 9.85E-03 | 7.39E-04 | NA | 1.37E+00 | 1.03E-01 | NA | NA | NA | NA |
| **1284** | NA | NA | NA | 1.42E+00 | NA | NA | NA | NA | NA |
| **1285** | 8.36E-03 | 0.00E+00 | NA | 1.12E+00 | 0.00E+00 | NA | NA | NA | NA |
| **1286** | 0.00E+00 | 1.97E-03 | NA | 1.38E+00 | Inf | NA | NA | NA | NA |
| **1287** | 9.50E-03 | 6.67E-03 | NA | 9.00E-01 | 6.32E-01 | NA | NA | NA | NA |
| **1288** | 4.87E-03 | 6.69E-04 | NA | 6.82E-01 | 9.36E-02 | NA | NA | NA | NA |

| | | | | | | | | | |
|---|---|---|---|---|---|---|---|---|---|
| **1289** | 6.81E-03 | 1.31E-02 | NA | 6.24E-01 | 1.20E+00 | NA | NA | NA | NA |
| **1290** | 2.07E-02 | 3.25E-03 | NA | 8.32E-01 | 1.30E-01 | NA | NA | NA | NA |
| **1291** | 5.61E-03 | 0.00E+00 | 1.44E+00 | 4.34E-01 | 0.00E+00 | (0.0348,Inf] | (14.9,Inf] | (0.0184,Inf] | (21,Inf] |
| **1292** | 1.85E-03 | 1.56E-02 | NA | 7.89E-01 | 6.64E+00 | NA | NA | NA | NA |
| **1293** | 7.95E-03 | 0.00E+00 | NA | 6.56E-01 | 0.00E+00 | NA | NA | NA | NA |
| **1294** | 1.63E-02 | 2.00E-03 | NA | 1.61E+00 | 1.96E-01 | NA | NA | NA | NA |
| **1295** | 1.22E-02 | 2.94E-03 | NA | 9.53E-01 | 2.29E-01 | NA | NA | NA | NA |
| **1296** | NA | NA | NA | 6.45E-01 | NA | NA | NA | NA | NA |
| **1297** | 0.00E+00 | 1.74E-03 | NA | 9.51E-01 | Inf | NA | NA | NA | NA |
| **1298** | 8.89E-03 | 0.00E+00 | NA | 1.33E+00 | 0.00E+00 | NA | NA | NA | NA |
| **1299** | 8.59E-03 | 7.47E-03 | NA | 5.31E-01 | 4.62E-01 | NA | NA | NA | NA |
| **1300** | 1.80E-02 | 0.00E+00 | NA | 6.23E-01 | 0.00E+00 | NA | NA | NA | NA |
| **1301** | NA | NA | NA | 8.33E-01 | NA | NA | NA | NA | NA |
| **1302** | 8.32E-03 | 5.47E-03 | NA | 7.56E-01 | 4.97E-01 | NA | NA | NA | NA |
| **1303** | 3.94E-02 | 3.46E-02 | 2.49E+00 | 1.64E+00 | 1.44E+00 | (0.00294,0.0348] | (0.101,1.34] | (0.0184,Inf] | (0.192,21] |
| **1304** | 0.00E+00 | 0.00E+00 | NA | 6.21E-01 | NA | NA | NA | NA | NA |
| **1305** | 0.00E+00 | 1.51E-03 | NA | 4.70E-01 | Inf | NA | NA | NA | NA |
| **1306** | 1.01E-03 | 0.00E+00 | NA | 9.37E-01 | 0.00E+00 | NA | NA | NA | NA |
| **1307** | NA | NA | NA | 1.11E+00 | NA | NA | NA | NA | NA |
| **1308** | 8.56E-03 | 6.04E-03 | 0.00E+00 | 1.68E+00 | 1.18E+00 | (0.00294,0.0348] | (0.101,1.34] | NA | NA |
| **1309** | NA | NA | NA | 7.28E-01 | NA | NA | NA | NA | NA |
| **1310** | 8.61E-03 | 4.56E-03 | NA | 2.37E+00 | 1.26E+00 | NA | NA | NA | NA |
| **1311** | 1.29E-02 | 3.00E-02 | NA | 8.40E-01 | 1.95E+00 | NA | NA | NA | NA |
| **1312** | 1.24E-02 | 1.92E-02 | 0.00E+00 | 8.50E-01 | 1.32E+00 | (0.0348,Inf] | (1.34,14.9] | NA | NA |
| **1313** | 9.97E-03 | 7.53E-03 | NA | 8.18E-01 | 6.18E-01 | NA | NA | NA | NA |
| **1314** | 4.21E-03 | 3.60E-03 | NA | 1.53E+00 | 1.31E+00 | NA | NA | NA | NA |
| **1315** | 0.00E+00 | 0.00E+00 | NA | 1.18E+00 | NA | NA | NA | NA | NA |
| **1316** | 1.46E-02 | 1.64E-02 | NA | 5.11E-01 | 5.75E-01 | NA | NA | NA | NA |
| **1317** | 7.97E-03 | 1.43E-03 | NA | 8.46E-01 | 1.51E-01 | NA | NA | NA | NA |
| **1318** | NA | NA | NA | 6.99E-01 | NA | NA | NA | NA | NA |
| **1319** | 6.35E-03 | 0.00E+00 | NA | 6.14E-01 | 0.00E+00 | NA | NA | NA | NA |
| **1320** | NA | NA | NA | 9.66E-01 | NA | NA | NA | NA | NA |
| **1321** | 0.00E+00 | 0.00E+00 | NA | 5.46E-01 | NA | NA | NA | NA | NA |
| **1322** | NA | NA | NA | 6.23E-01 | NA | NA | NA | NA | NA |
| **1323** | NA | NA | NA | 7.27E-01 | NA | NA | NA | NA | NA |
| **1324** | NA | NA | NA | 1.02E+00 | NA | NA | NA | NA | NA |
| **1325** | 1.46E-02 | 0.00E+00 | NA | 1.52E+00 | 0.00E+00 | NA | NA | NA | NA |
| **1326** | 0.00E+00 | 1.74E-03 | NA | 7.23E-01 | Inf | NA | NA | NA | NA |

| | | | | | | | | | |
|---|---|---|---|---|---|---|---|---|---|
| **1327** | 1.12E-02 | 0.00E+00 | NA | 1.33E+00 | 0.00E+00 | NA | NA | NA | NA |
| **1328** | 5.15E-03 | 0.00E+00 | NA | 8.74E-01 | 0.00E+00 | NA | NA | NA | NA |
| **1329** | NA | NA | 9.76E-01 | 1.25E+00 | NA | (0.0348,Inf] | NA | (0.0184,Inf] | NA |
| **1330** | NA | NA | Inf | NA | NA | NA | NA | NA | (21,Inf] |
| **1331** | NA | NA | NA | 7.75E-01 | NA | NA | NA | NA | NA |
| **1332** | NA | NA | NA | 7.61E-01 | NA | NA | NA | NA | NA |
| **1333** | 7.25E-03 | 1.53E-02 | NA | 5.60E-01 | 1.18E+00 | NA | NA | NA | NA |
| **1334** | NA | NA | NA | 4.82E-01 | NA | NA | NA | NA | NA |
| **1335** | 0.00E+00 | 9.11E-03 | NA | 1.19E+00 | Inf | NA | NA | NA | NA |
| **1336** | 1.29E-02 | 1.38E-02 | NA | 6.70E-01 | 7.20E-01 | NA | NA | NA | NA |
| **1337** | 1.06E-02 | 0.00E+00 | NA | 8.51E-01 | 0.00E+00 | NA | NA | NA | NA |
| **1338** | 1.93E-02 | 1.37E-02 | NA | 1.76E+00 | 1.25E+00 | NA | NA | NA | NA |
| **1339** | 8.55E-04 | 1.16E-03 | NA | 6.76E-01 | 9.19E-01 | NA | NA | NA | NA |
| **1340** | 3.73E-03 | 1.11E-02 | 6.95E+00 | 5.54E-01 | 1.64E+00 | (0.0348,Inf] | (14.9,Inf] | (0.0184,Inf] | (21,Inf] |
| **1341** | NA | NA | NA | 1.51E+00 | NA | NA | NA | NA | NA |
| **1342** | 3.51E-03 | 0.00E+00 | NA | 1.35E+00 | 0.00E+00 | NA | NA | NA | NA |
| **1343** | NA | NA | NA | 8.30E-01 | NA | NA | NA | NA | NA |
| **1344** | 6.95E-03 | 0.00E+00 | NA | 6.18E-01 | 0.00E+00 | NA | NA | NA | NA |
| **1345** | 1.68E-03 | 1.60E-03 | 0.00E+00 | 8.00E-01 | 7.65E-01 | (0.00294,0.0348] | (14.9,Inf] | NA | NA |
| **1346** | NA | NA | NA | 5.60E-01 | NA | NA | NA | NA | NA |
| **1347** | 0.00E+00 | 0.00E+00 | NA | 2.14E+01 | NA | NA | NA | NA | NA |
| **1348** | 1.20E-03 | 1.12E-02 | NA | 6.68E-01 | 6.22E+00 | NA | NA | NA | NA |
| **1349** | 2.95E-02 | 2.38E-02 | 0.00E+00 | 1.46E+00 | 1.18E+00 | (0.0348,Inf] | (14.9,Inf] | NA | NA |
| **1350** | 0.00E+00 | 1.09E-02 | NA | 1.79E+00 | Inf | NA | NA | NA | NA |
| **1351** | 8.44E-03 | 3.57E-03 | Inf | 5.17E-01 | 2.19E-01 | NA | NA | (0.0184,Inf] | (21,Inf] |
| **1352** | 1.59E-02 | 8.99E-03 | NA | 1.66E+00 | 9.38E-01 | NA | NA | NA | NA |
| **1353** | 4.50E-03 | 6.27E-03 | NA | 4.90E-01 | 6.83E-01 | NA | NA | NA | NA |
| **1354** | 1.34E-02 | 1.11E-02 | NA | 1.03E+00 | 8.57E-01 | NA | NA | NA | NA |
| **1355** | NA | NA | 7.49E-01 | 9.78E-01 | NA | (0.00294,0.0348] | NA | (0.000255,0.0184] | NA |
| **1356** | 1.49E-02 | 0.00E+00 | NA | 1.40E+00 | 0.00E+00 | NA | NA | NA | NA |
| **1357** | 1.03E-02 | 2.29E-03 | NA | 9.31E-01 | 2.08E-01 | NA | NA | NA | NA |
| **1358** | 0.00E+00 | 0.00E+00 | NA | 7.23E-01 | NA | NA | NA | NA | NA |
| **1359** | 7.72E-03 | 0.00E+00 | NA | 1.47E+00 | 0.00E+00 | NA | NA | NA | NA |
| **1360** | 5.06E-03 | 1.22E-03 | NA | 8.91E-01 | 2.15E-01 | NA | NA | NA | NA |
| **1361** | 3.29E-03 | 0.00E+00 | NA | 1.18E+00 | 0.00E+00 | NA | NA | NA | NA |
| **1362** | 7.48E-02 | 2.52E-03 | Inf | 7.96E-01 | 2.68E-02 | NA | NA | (0.0184,Inf] | (21,Inf] |
| **1363** | 7.13E-03 | 3.89E-03 | NA | 9.82E-01 | 5.36E-01 | NA | NA | NA | NA |
| **1364** | 1.04E-02 | 3.53E-03 | NA | 8.38E-01 | 2.84E-01 | NA | NA | NA | NA |

| | | | | | | | | | |
|---|---|---|---|---|---|---|---|---|---|
| **1365** | NA | NA | NA | 5.26E-01 | NA | NA | NA | NA | NA |
| **1366** | NA | NA | NA | 5.66E-01 | NA | NA | NA | NA | NA |
| **1367** | 1.38E-02 | 4.47E-03 | NA | 1.17E+00 | 3.80E-01 | NA | NA | NA | NA |
| **1368** | 1.09E-02 | 1.28E-02 | NA | 9.96E-01 | 1.17E+00 | NA | NA | NA | NA |
| **1369** | 1.62E-02 | 1.40E-02 | 0.00E+00 | 2.23E+00 | 1.92E+00 | (0.0348,Inf] | (14.9,Inf] | NA | NA |
| **1370** | 1.12E-02 | 2.87E-03 | NA | 1.16E+00 | 2.98E-01 | NA | NA | NA | NA |
| **1371** | 1.61E-02 | 9.64E-03 | 0.00E+00 | 4.77E-01 | 2.86E-01 | (0.0348,Inf] | (1.34,14.9] | NA | NA |
| **1372** | 2.66E-02 | 2.42E-02 | NA | 6.64E-01 | 6.03E-01 | NA | NA | NA | NA |
| **1373** | 0.00E+00 | 0.00E+00 | NA | 9.18E-01 | NA | NA | NA | NA | NA |
| **1374** | 0.00E+00 | 0.00E+00 | NA | 1.77E+00 | NA | NA | NA | NA | NA |
| **1375** | 9.21E-03 | 0.00E+00 | NA | 5.05E-01 | 0.00E+00 | NA | NA | NA | NA |
| **1376** | 8.52E-03 | 7.79E-03 | 9.48E-02 | 1.20E+00 | 1.10E+00 | (0.0348,Inf] | (1.34,14.9] | (0.000255,0.0184] | (0.192,21] |
| **1377** | 0.00E+00 | 0.00E+00 | NA | 1.36E-01 | NA | NA | NA | NA | NA |
| **1378** | NA | NA | NA | 4.98E-01 | NA | NA | NA | NA | NA |
| **1379** | 2.72E-02 | 0.00E+00 | Inf | 4.91E-01 | 0.00E+00 | NA | NA | (0.000255,0.0184] | (21,Inf] |
| **1380** | 4.82E-03 | 1.74E-03 | 5.56E+01 | 3.87E-01 | 1.40E-01 | (0.0348,Inf] | (1.34,14.9] | (0.0184,Inf] | (21,Inf] |
| **1381** | 4.87E-03 | 0.00E+00 | 0.00E+00 | 2.06E+00 | 0.00E+00 | (0.00294,0.0348] | (1.34,14.9] | NA | NA |
| **1382** | 4.47E-03 | 1.87E-02 | 0.00E+00 | 1.00E+00 | 4.19E+00 | (0.0348,Inf] | (14.9,Inf] | NA | NA |
| **1383** | 0.00E+00 | 0.00E+00 | Inf | 4.66E-01 | NA | NA | NA | (0.0184,Inf] | (21,Inf] |
| **1384** | 5.23E-03 | 0.00E+00 | Inf | 6.14E-01 | 0.00E+00 | NA | NA | (0.000255,0.0184] | (21,Inf] |
| **1385** | 7.38E-03 | 1.01E-03 | NA | 1.01E+00 | 1.38E-01 | NA | NA | NA | NA |
| **1386** | 1.01E-02 | 0.00E+00 | NA | 6.36E-01 | 0.00E+00 | NA | NA | NA | NA |
| **1387** | 1.28E-02 | 3.20E-03 | NA | 8.29E-01 | 2.07E-01 | NA | NA | NA | NA |
| **1388** | 0.00E+00 | 6.50E-03 | NA | 3.98E-01 | Inf | NA | NA | NA | NA |
| **1389** | NA | NA | NA | 8.95E-01 | NA | NA | NA | NA | NA |
| **1390** | 8.56E-03 | 3.76E-03 | NA | 1.01E+00 | 4.46E-01 | NA | NA | NA | NA |
| **1391** | 2.94E-03 | 4.96E-03 | NA | 6.08E-01 | 1.03E+00 | NA | NA | NA | NA |
| **1392** | 1.35E-02 | 0.00E+00 | Inf | 1.31E+00 | 0.00E+00 | NA | NA | (0.0184,Inf] | (21,Inf] |
| **1393** | 6.50E-03 | 8.60E-03 | NA | 1.15E+00 | 1.53E+00 | NA | NA | NA | NA |
| **1394** | 5.32E-03 | 7.53E-03 | Inf | 6.52E-01 | 9.24E-01 | NA | NA | (0.000255,0.0184] | (0.192,21] |
| **1395** | 1.71E-03 | 0.00E+00 | 2.04E+00 | 1.03E+00 | 0.00E+00 | (0.0348,Inf] | (14.9,Inf] | (0.0184,Inf] | (21,Inf] |
| **1396** | 1.22E-02 | 2.83E-03 | 0.00E+00 | 1.54E+00 | 3.56E-01 | (0.0348,Inf] | (14.9,Inf] | NA | NA |
| **1397** | 1.04E-02 | 1.23E-02 | 0.00E+00 | 5.36E-01 | 6.31E-01 | (0.00294,0.0348] | (1.34,14.9] | NA | NA |
| **1398** | NA | NA | NA | 7.14E-01 | NA | NA | NA | NA | NA |
| **1399** | 6.59E-03 | 5.59E-03 | NA | 9.67E-01 | 8.20E-01 | NA | NA | NA | NA |
| **1400** | 5.00E-02 | 2.00E-02 | Inf | 6.50E-01 | 2.60E-01 | NA | NA | (0.0184,Inf] | (0.192,21] |
| **1401** | 3.51E-04 | 2.53E-03 | Inf | 7.08E-01 | 5.10E+00 | NA | NA | (0.0184,Inf] | (0.192,21] |
| **1402** | 0.00E+00 | 8.71E-03 | NA | 9.26E-01 | Inf | NA | NA | NA | NA |

| | | | | | | | | | |
|---|---|---|---|---|---|---|---|---|---|
| **1403** | 8.27E-03 | 7.06E-03 | NA | 7.16E-01 | 6.11E-01 | NA | NA | NA | NA |
| **1404** | 1.18E-02 | 6.65E-03 | NA | 9.52E-01 | 5.37E-01 | NA | NA | NA | NA |
| **1405** | 7.92E-03 | 3.82E-03 | NA | 8.33E-01 | 4.02E-01 | NA | NA | NA | NA |
| **1406** | 3.62E-03 | 3.56E-03 | NA | 8.89E-01 | 8.73E-01 | NA | NA | NA | NA |
| **1407** | 1.35E-02 | 1.44E-03 | 0.00E+00 | 1.38E+00 | 1.48E-01 | (0.0348,Inf] | (1.34,14.9] | NA | NA |
| **1408** | 3.19E-02 | 0.00E+00 | NA | 1.49E+00 | 0.00E+00 | NA | NA | NA | NA |
| **1409** | 2.85E-03 | 2.10E-02 | NA | 3.18E-01 | 2.34E+00 | NA | NA | NA | NA |
| **1410** | 5.66E-03 | 1.30E-02 | NA | 6.52E-01 | 1.49E+00 | NA | NA | NA | NA |
| **1411** | NA | NA | NA | 3.78E-01 | NA | NA | NA | NA | NA |
| **1412** | NA | NA | NA | 8.04E-01 | NA | NA | NA | NA | NA |
| **1413** | 2.20E-02 | 6.32E-03 | 3.06E-01 | 2.59E+00 | 7.44E-01 | (0.0348,Inf] | (1.34,14.9] | (0.000255,0.0184] | (0.192,21] |
| **1414** | NA | NA | NA | 6.01E-01 | NA | NA | NA | NA | NA |
| **1415** | 2.83E-02 | 1.04E-02 | NA | 1.39E+00 | 5.14E-01 | NA | NA | NA | NA |
| **1416** | 1.34E-02 | 0.00E+00 | NA | 1.63E+00 | 0.00E+00 | NA | NA | NA | NA |
| **1417** | 2.42E-02 | 7.83E-03 | 0.00E+00 | 1.25E+00 | 4.06E-01 | (0.0348,Inf] | (1.34,14.9] | NA | NA |
| **1418** | 8.90E-03 | 2.92E-03 | NA | 6.60E-01 | 2.17E-01 | NA | NA | NA | NA |
| **1419** | NA | NA | NA | 6.45E-01 | NA | NA | NA | NA | NA |
| **1420** | 3.01E-03 | 1.39E-02 | NA | 8.97E-01 | 4.14E+00 | NA | NA | NA | NA |
| **1421** | 1.40E-02 | 0.00E+00 | NA | 1.50E+00 | 0.00E+00 | NA | NA | NA | NA |
| **1422** | 0.00E+00 | 8.39E-04 | NA | 1.05E+00 | Inf | NA | NA | NA | NA |
| **1423** | 9.10E-03 | 0.00E+00 | NA | 1.07E+00 | 0.00E+00 | NA | NA | NA | NA |
| **1424** | NA | NA | 0.00E+00 | NA | 3.34E-01 | NA | (1.34,14.9] | NA | NA |
| **1425** | 2.12E-03 | 1.27E-03 | NA | 1.11E+00 | 6.64E-01 | NA | NA | NA | NA |
| **1426** | 0.00E+00 | 0.00E+00 | NA | 8.12E-01 | NA | NA | NA | NA | NA |
| **1427** | NA | NA | NA | 7.08E-01 | NA | NA | NA | NA | NA |
| **1428** | NA | NA | Inf | 1.03E+00 | NA | NA | NA | (0.0184,Inf] | NA |
| **1429** | NA | NA | NA | 1.38E+00 | NA | NA | NA | NA | NA |
| **1430** | 0.00E+00 | 0.00E+00 | NA | 8.35E-01 | NA | NA | NA | NA | NA |
| **1431** | 1.07E-02 | 0.00E+00 | NA | 1.03E+00 | 0.00E+00 | NA | NA | NA | NA |
| **1432** | 9.50E-03 | 0.00E+00 | NA | 7.31E-01 | 0.00E+00 | NA | NA | NA | NA |
| **1433** | NA | NA | NA | 1.77E+00 | NA | NA | NA | NA | NA |
| **1434** | 0.00E+00 | 1.29E-03 | NA | 1.30E+00 | Inf | NA | NA | NA | NA |
| **1435** | NA | NA | NA | 1.80E-01 | NA | NA | NA | NA | NA |
| **1436** | 4.51E-03 | 4.10E-03 | NA | 6.10E-01 | 5.56E-01 | NA | NA | NA | NA |
| **1437** | 1.02E-03 | 6.09E-03 | NA | 5.33E-01 | 3.17E+00 | NA | NA | NA | NA |
| **1438** | 3.55E-03 | 2.15E-02 | NA | 3.06E-01 | 1.85E+00 | NA | NA | NA | NA |
| **1439** | 0.00E+00 | 0.00E+00 | NA | 4.90E-01 | NA | NA | NA | NA | NA |
| **1440** | 0.00E+00 | 1.03E-02 | NA | 8.35E-01 | Inf | NA | NA | NA | NA |

| | | | | | | | | | |
|---|---|---|---|---|---|---|---|---|---|
| **1441** | 1.88E-02 | 0.00E+00 | NA | 9.11E-01 | 0.00E+00 | NA | NA | NA | NA |
| **1442** | 3.15E-02 | 3.70E-03 | NA | 1.32E+00 | 1.55E-01 | NA | NA | NA | NA |
| **1443** | 0.00E+00 | 5.37E-03 | NA | 5.09E-01 | Inf | NA | NA | NA | NA |
| **1444** | 1.62E-02 | 1.34E-03 | NA | 7.67E-01 | 6.34E-02 | NA | NA | NA | NA |
| **1445** | 0.00E+00 | 0.00E+00 | NA | 6.36E-01 | NA | NA | NA | NA | NA |
| **1446** | 6.68E-03 | 0.00E+00 | NA | 4.66E-01 | 0.00E+00 | NA | NA | NA | NA |
| **1447** | 1.75E-02 | 0.00E+00 | Inf | 6.97E-01 | 0.00E+00 | NA | NA | (0.0184,Inf] | (21,Inf] |
| **1448** | 2.16E-02 | 1.01E-02 | 1.85E-01 | 8.46E-01 | 3.95E-01 | (0.0348,Inf] | (1.34,14.9] | (0.0184,Inf] | (0.192,21] |
| **1449** | 2.74E-03 | 0.00E+00 | NA | 8.97E-01 | 0.00E+00 | NA | NA | NA | NA |
| **1450** | NA | NA | NA | 9.11E-01 | NA | NA | NA | NA | NA |
| **1451** | 0.00E+00 | 0.00E+00 | NA | 8.80E-01 | NA | NA | NA | NA | NA |
| **1452** | 6.86E-03 | 5.60E-04 | NA | 1.08E+00 | 8.81E-02 | NA | NA | NA | NA |
| **1453** | 1.14E-02 | 0.00E+00 | NA | 4.30E-01 | 0.00E+00 | NA | NA | NA | NA |
| **1454** | 5.23E-02 | 5.85E-03 | NA | 1.07E+00 | 1.19E-01 | NA | NA | NA | NA |
| **1455** | 0.00E+00 | 5.98E-03 | NA | 9.37E-01 | Inf | NA | NA | NA | NA |
| **1456** | 0.00E+00 | 4.76E-03 | NA | 4.68E-01 | Inf | NA | NA | NA | NA |
| **1457** | NA | NA | NA | 3.25E-01 | NA | NA | NA | NA | NA |
| **1458** | NA | NA | NA | 1.32E+00 | NA | NA | NA | NA | NA |
| **1459** | 8.63E-03 | 1.06E-03 | NA | 1.54E+00 | 1.90E-01 | NA | NA | NA | NA |
| **1460** | 3.29E-02 | 3.36E-02 | NA | 7.49E-01 | 7.65E-01 | NA | NA | NA | NA |
| **1461** | 3.22E-02 | 1.05E-02 | NA | 5.84E-01 | 1.91E-01 | NA | NA | NA | NA |
| **1462** | NA | NA | NA | 6.89E-01 | NA | NA | NA | NA | NA |
| **1463** | 2.76E-02 | 6.56E-03 | NA | 1.25E+00 | 2.98E-01 | NA | NA | NA | NA |
| **1464** | 2.04E-02 | 1.76E-02 | NA | 8.85E-01 | 7.63E-01 | NA | NA | NA | NA |
| **1465** | 3.85E-02 | 0.00E+00 | NA | 2.66E+00 | 0.00E+00 | NA | NA | NA | NA |
| **1466** | 3.06E-03 | 2.13E-02 | NA | 2.29E-01 | 1.59E+00 | NA | NA | NA | NA |
| **1467** | 1.78E-02 | 6.60E-03 | 0.00E+00 | 9.38E-01 | 3.48E-01 | (0.0348,Inf] | (1.34,14.9] | NA | NA |
| **1468** | NA | NA | NA | 7.57E-01 | NA | NA | NA | NA | NA |
| **1469** | 3.36E-02 | 1.92E-02 | 9.24E-01 | 1.84E+00 | 1.05E+00 | (0.00294,0.0348] | (0.101,1.34] | (0.000255,0.0184] | (0.192,21] |
| **1470** | 8.50E-03 | 3.94E-03 | NA | 8.61E-01 | 4.00E-01 | NA | NA | NA | NA |
| **1471** | 8.26E-03 | 2.39E-02 | 0.00E+00 | 5.31E-01 | 1.54E+00 | (0.00294,0.0348] | (0.101,1.34] | NA | NA |
| **1472** | NA | NA | NA | 1.24E+00 | NA | NA | NA | NA | NA |
| **1473** | NA | NA | Inf | NA | NA | NA | NA | NA | (21,Inf] |
| **1474** | NA | NA | NA | 7.42E-01 | NA | NA | NA | NA | NA |
| **1475** | 5.19E-04 | 7.34E-03 | NA | 4.48E-01 | 6.34E+00 | NA | NA | NA | NA |
| **1476** | 0.00E+00 | 3.00E-03 | NA | 1.03E+00 | Inf | NA | NA | NA | NA |
| **1477** | NA | NA | NA | 6.63E-01 | NA | NA | NA | NA | NA |
| **1478** | 1.56E-02 | 0.00E+00 | NA | 1.34E+00 | 0.00E+00 | NA | NA | NA | NA |

| | | | | | | | | | |
|---|---|---|---|---|---|---|---|---|---|
| **1479** | 8.05E-03 | 3.27E-02 | NA | 5.84E-01 | 2.37E+00 | NA | NA | NA | NA |
| **1480** | 2.06E-02 | 1.21E-02 | NA | 8.96E-01 | 5.28E-01 | NA | NA | NA | NA |
| **1481** | 1.14E-02 | 3.74E-02 | NA | 5.17E-01 | 1.69E+00 | NA | NA | NA | NA |
| **1482** | NA | NA | NA | 9.94E-01 | NA | NA | NA | NA | NA |
| **1483** | 0.00E+00 | 3.85E-03 | NA | 7.19E-01 | Inf | NA | NA | NA | NA |
| **1484** | 6.51E-03 | 8.85E-03 | NA | 1.12E+00 | 1.52E+00 | NA | NA | NA | NA |
| **1485** | 1.64E-02 | 0.00E+00 | NA | 1.06E+00 | 0.00E+00 | NA | NA | NA | NA |
| **1486** | 1.96E-02 | 2.12E-02 | NA | 1.48E+00 | 1.60E+00 | NA | NA | NA | NA |
| **1487** | NA | NA | NA | 9.12E-01 | NA | NA | NA | NA | NA |
| **1488** | 9.96E-03 | 1.11E-02 | NA | 1.02E+00 | 1.14E+00 | NA | NA | NA | NA |
| **1489** | 2.25E-02 | 1.28E-02 | NA | 1.81E+00 | 1.03E+00 | NA | NA | NA | NA |
| **1490** | 3.42E-01 | 7.14E-01 | 3.12E-01 | 6.36E-01 | 1.33E+00 | (0.0348,Inf] | (0.101,1.34] | (0.0184,Inf] | (0,0.192] |
| **1491** | 2.68E-02 | 1.43E-02 | 0.00E+00 | 2.35E+00 | 1.26E+00 | (0.0348,Inf] | (1.34,14.9] | NA | NA |
| **1492** | 0.00E+00 | 3.18E-03 | NA | 1.96E+00 | Inf | NA | NA | NA | NA |
| **1493** | 3.65E-03 | 5.66E-03 | NA | 1.06E+00 | 1.65E+00 | NA | NA | NA | NA |
| **1494** | NA | NA | NA | 1.43E+00 | NA | NA | NA | NA | NA |
| **1495** | 1.42E-03 | 5.56E-03 | NA | 1.59E+00 | 6.22E+00 | NA | NA | NA | NA |
| **1496** | 3.64E-03 | 7.54E-03 | 0.00E+00 | 8.34E-01 | 1.73E+00 | (0.0348,Inf] | (1.34,14.9] | NA | NA |
| **1497** | 0.00E+00 | 1.51E-02 | NA | 3.54E-01 | Inf | NA | NA | NA | NA |
| **1498** | 1.25E-02 | 3.95E-03 | 0.00E+00 | 1.23E+00 | 3.92E-01 | (0.0348,Inf] | (14.9,Inf] | NA | NA |
| **1499** | 4.39E-02 | 9.91E-03 | 0.00E+00 | 1.84E+00 | 4.14E-01 | (0.00294,0.0348] | (0.101,1.34] | NA | NA |
| **1500** | 2.58E-02 | 9.95E-03 | NA | 6.42E-01 | 2.48E-01 | NA | NA | NA | NA |
| **1501** | 1.23E-02 | 8.09E-02 | 1.09E+00 | 4.37E-01 | 2.86E+00 | (0.0348,Inf] | (1.34,14.9] | (0.0184,Inf] | (0.192,21] |
| **1502** | 1.20E-02 | 2.13E-03 | NA | 1.37E+00 | 2.43E-01 | NA | NA | NA | NA |
| **1503** | 5.23E-02 | 1.44E-02 | Inf | 1.99E+00 | 5.47E-01 | NA | NA | (0.000255,0.0184] | (0.192,21] |
| **1504** | 7.22E-03 | 2.27E-03 | NA | 1.09E+00 | 3.42E-01 | NA | NA | NA | NA |
| **1505** | 9.60E-03 | 0.00E+00 | NA | 1.10E+00 | 0.00E+00 | NA | NA | NA | NA |
| **1506** | NA | NA | NA | 1.15E+00 | NA | NA | NA | NA | NA |
| **1507** | 2.12E-02 | 2.67E-03 | NA | 1.70E+00 | 2.14E-01 | NA | NA | NA | NA |
| **1508** | 4.21E-03 | 1.52E-02 | NA | 8.47E-01 | 3.05E+00 | NA | NA | NA | NA |
| **1509** | NA | NA | NA | 6.85E-01 | NA | NA | NA | NA | NA |
| **1510** | 2.76E-03 | 1.26E-03 | NA | 3.14E+00 | 1.43E+00 | NA | NA | NA | NA |
| **1511** | 1.01E-02 | 0.00E+00 | NA | 9.62E-01 | 0.00E+00 | NA | NA | NA | NA |
| **1512** | 1.50E-02 | 1.56E-02 | NA | 1.45E+00 | 1.50E+00 | NA | NA | NA | NA |
| **1513** | 3.44E-03 | 3.62E-03 | NA | 1.21E+00 | 1.27E+00 | NA | NA | NA | NA |
| **1514** | 0.00E+00 | 5.82E-03 | NA | 7.44E-01 | Inf | NA | NA | NA | NA |
| **1515** | NA | NA | NA | 9.28E-01 | NA | NA | NA | NA | NA |
| **1516** | 1.78E-02 | 9.02E-04 | NA | 2.28E+00 | 1.15E-01 | NA | NA | NA | NA |

| | | | | | | | | | |
|---|---|---|---|---|---|---|---|---|---|
| **1517** | 8.47E-03 | 1.53E-02 | NA | 4.08E-01 | 7.37E-01 | NA | NA | NA | NA |
| **1518** | 0.00E+00 | 7.83E-03 | NA | 6.43E-01 | Inf | NA | NA | NA | NA |
| **1519** | NA | NA | NA | 9.13E-01 | NA | NA | NA | NA | NA |
| **1520** | 5.97E-03 | 0.00E+00 | NA | 1.14E+00 | 0.00E+00 | NA | NA | NA | NA |
| **1521** | 2.70E-03 | 4.82E-03 | Inf | 7.34E-01 | 1.31E+00 | NA | NA | (0.0184,Inf] | (0.192,21] |
| **1522** | 9.16E-03 | 0.00E+00 | NA | 5.45E-01 | 0.00E+00 | NA | NA | NA | NA |
| **1523** | 1.03E-02 | 0.00E+00 | NA | 1.18E+00 | 0.00E+00 | NA | NA | NA | NA |
| **1524** | NA | NA | NA | 1.00E+00 | NA | NA | NA | NA | NA |
| **1525** | 0.00E+00 | 1.05E-02 | NA | 1.27E+00 | Inf | NA | NA | NA | NA |
| **1526** | 9.59E-02 | 5.01E-03 | NA | 1.21E+00 | 6.33E-02 | NA | NA | NA | NA |
| **1527** | NA | NA | NA | 7.86E-01 | NA | NA | NA | NA | NA |
| **1528** | 1.91E-02 | 1.31E-02 | NA | 9.98E-01 | 6.85E-01 | NA | NA | NA | NA |
| **1529** | 2.70E-02 | 5.95E-03 | NA | 7.20E-01 | 1.59E-01 | NA | NA | NA | NA |
| **1530** | NA | NA | NA | 0.00E+00 | NA | NA | NA | NA | NA |
| **1531** | NA | NA | NA | 7.80E-01 | NA | NA | NA | NA | NA |
| **1532** | NA | NA | NA | 7.89E-01 | NA | NA | NA | NA | NA |
| **1533** | NA | NA | NA | 6.90E-01 | NA | NA | NA | NA | NA |
| **1534** | 5.53E-03 | 5.52E-03 | NA | 4.99E-01 | 4.97E-01 | NA | NA | NA | NA |
| **1535** | NA | NA | NA | 1.00E+00 | NA | NA | NA | NA | NA |
| **1536** | 2.21E-01 | 5.03E-01 | NA | 4.90E-01 | 1.11E+00 | NA | NA | NA | NA |
| **1537** | 1.79E-02 | 1.02E-02 | NA | 1.09E+00 | 6.24E-01 | NA | NA | NA | NA |
| **1538** | 0.00E+00 | 0.00E+00 | NA | 2.04E+00 | NA | NA | NA | NA | NA |
| **1539** | NA | NA | NA | 1.51E+00 | NA | NA | NA | NA | NA |
| **1540** | 2.42E-03 | 1.66E-02 | NA | 9.29E-01 | 6.38E+00 | NA | NA | NA | NA |
| **1541** | 0.00E+00 | 5.79E-03 | NA | 5.48E-01 | Inf | NA | NA | NA | NA |
| **1542** | NA | NA | NA | 4.29E-01 | NA | NA | NA | NA | NA |
| **1543** | 1.16E-02 | 0.00E+00 | NA | 8.39E-01 | 0.00E+00 | NA | NA | NA | NA |
| **1544** | 1.89E-02 | 1.15E-02 | NA | 7.51E-01 | 4.55E-01 | NA | NA | NA | NA |
| **1545** | 3.53E-03 | 1.71E-02 | NA | 6.26E-01 | 3.04E+00 | NA | NA | NA | NA |
| **1546** | NA | NA | Inf | 6.31E-01 | NA | NA | NA | (0.000255,0.0184] | NA |
| **1547** | 7.66E-03 | 2.51E-02 | 7.17E-01 | 7.27E-01 | 2.38E+00 | (0.0348,Inf] | (14.9,Inf] | (0.0184,Inf] | (0.192,21] |
| **1548** | 8.16E-03 | 4.49E-03 | NA | 6.97E-01 | 3.84E-01 | NA | NA | NA | NA |
| **1549** | 0.00E+00 | 1.96E-02 | NA | 1.79E+00 | Inf | NA | NA | NA | NA |
| **1550** | NA | NA | NA | 9.34E-01 | NA | NA | NA | NA | NA |
| **1551** | NA | NA | NA | 3.06E+00 | NA | NA | NA | NA | NA |
| **1552** | 1.21E-02 | 1.32E-02 | NA | 1.75E+00 | 1.90E+00 | NA | NA | NA | NA |
| **1553** | NA | NA | NA | 7.87E-01 | NA | NA | NA | NA | NA |
| **1554** | 1.47E-02 | 2.77E-02 | NA | 1.70E-01 | 3.19E-01 | NA | NA | NA | NA |

| | | | | | | | | | | |
|---|---|---|---|---|---|---|---|---|---|---|
| **1555** | 2.25E-03 | 0.00E+00 | NA | 4.98E-01 | 0.00E+00 | | NA | NA | NA | NA |
| **1556** | 1.30E-02 | 1.07E-02 | NA | 6.35E-01 | 5.20E-01 | | NA | NA | NA | NA |
| **1557** | NA | NA | NA | NA | 2.19E+00 | | NA | NA | NA | NA |
| **1558** | 5.81E-03 | 1.48E-02 | NA | 3.16E-01 | 8.07E-01 | | NA | NA | NA | NA |
| **1559** | NA | NA | NA | 1.50E+00 | NA | | NA | NA | NA | NA |
| **1560** | NA | NA | NA | 8.06E-01 | NA | | NA | NA | NA | NA |
| **1561** | NA | NA | NA | 8.58E-01 | NA | | NA | NA | NA | NA |
| **1562** | NA | NA | NA | 5.41E-01 | NA | | NA | NA | NA | NA |
| **1563** | 0.00E+00 | 0.00E+00 | NA | 9.67E-01 | NA | | NA | NA | NA | NA |
| **1564** | NA | NA | NA | 2.57E-01 | NA | | NA | NA | NA | NA |
| **1565** | 0.00E+00 | 0.00E+00 | NA | 8.13E-01 | NA | | NA | NA | NA | NA |
| **1566** | NA | NA | 1.25E+00 | NA | 7.03E-01 | | NA | (1.34,14.9] | NA | (0.192,21] |
| **1567** | 1.67E-03 | 0.00E+00 | NA | 7.05E-01 | 0.00E+00 | | NA | NA | NA | NA |
| **1568** | 0.00E+00 | 1.19E-02 | NA | 6.32E-01 | Inf | | NA | NA | NA | NA |
| **1569** | 2.84E-02 | 1.90E-02 | NA | 8.44E-01 | 5.67E-01 | | NA | NA | NA | NA |
| **1570** | NA | NA | NA | 7.17E-01 | NA | | NA | NA | NA | NA |
| **1571** | NA | NA | NA | 9.30E-01 | NA | | NA | NA | NA | NA |
| **1572** | 1.07E-03 | 0.00E+00 | NA | 6.47E-01 | 0.00E+00 | | NA | NA | NA | NA |
| **1573** | NA | NA | NA | 4.73E-01 | NA | | NA | NA | NA | NA |
| **1574** | NA | NA | NA | 6.82E-01 | NA | | NA | NA | NA | NA |
| **1575** | NA | NA | NA | 9.78E-01 | NA | | NA | NA | NA | NA |
| **1576** | NA | NA | NA | 2.54E+00 | NA | | NA | NA | NA | NA |
| **1577** | 1.00E-02 | 0.00E+00 | NA | 7.45E-01 | 0.00E+00 | | NA | NA | NA | NA |
| **1578** | 0.00E+00 | 0.00E+00 | Inf | 5.06E+00 | NA | | NA | NA | (0.000255,0.0184] | (21,Inf] |
| **1579** | 3.45E-02 | 2.03E-03 | Inf | 9.99E-01 | 5.89E-02 | | NA | NA | (0.0184,Inf] | (21,Inf] |
| **1580** | 0.00E+00 | 0.00E+00 | NA | 4.82E-01 | NA | | NA | NA | NA | NA |
| **1581** | NA | NA | NA | 3.63E-01 | NA | | NA | NA | NA | NA |
| **1582** | 1.54E-02 | 1.57E-02 | Inf | 5.00E-01 | 5.11E-01 | | NA | NA | (0.0184,Inf] | (0.192,21] |
| **1583** | 9.63E-03 | 6.76E-03 | NA | 9.44E-01 | 6.63E-01 | | NA | NA | NA | NA |
| **1584** | 1.38E-02 | 5.64E-03 | NA | 6.06E-01 | 2.47E-01 | | NA | NA | NA | NA |
| **1585** | 2.05E-03 | 4.53E-03 | NA | 7.28E-01 | 1.61E+00 | | NA | NA | NA | NA |
| **1586** | 2.74E-03 | 1.50E-02 | NA | 9.84E-01 | 5.38E+00 | | NA | NA | NA | NA |
| **1587** | 0.00E+00 | 0.00E+00 | NA | 2.73E-01 | NA | | NA | NA | NA | NA |
| **1588** | NA | NA | NA | 6.64E-01 | NA | | NA | NA | NA | NA |
| **1589** | 9.24E-03 | 0.00E+00 | NA | 1.19E+00 | 0.00E+00 | | NA | NA | NA | NA |
| **1590** | NA | NA | NA | 7.71E-01 | NA | | NA | NA | NA | NA |
| **1591** | 0.00E+00 | 0.00E+00 | NA | 9.02E-01 | NA | | NA | NA | NA | NA |
| **1592** | 0.00E+00 | 1.66E-03 | NA | 1.33E+00 | Inf | | NA | NA | NA | NA |

| | | | | | | | | | |
|---|---|---|---|---|---|---|---|---|---|
| **1593** | NA | NA | NA | 1.56E+00 | NA | NA | NA | NA | NA |
| **1594** | 1.63E-02 | 0.00E+00 | Inf | 9.25E-01 | 0.00E+00 | NA | NA | (0.0184,Inf] | (21,Inf] |
| **1595** | 1.22E-02 | 2.85E-02 | NA | 7.24E-01 | 1.70E+00 | NA | NA | NA | NA |
| **1596** | 0.00E+00 | 2.30E-03 | NA | 1.33E+00 | Inf | NA | NA | NA | NA |
| **1597** | 1.13E-02 | 2.89E-02 | 0.00E+00 | 3.08E-01 | 7.87E-01 | (0.0348,Inf] | (1.34,14.9] | NA | NA |
| **1598** | 9.47E-03 | 2.80E-02 | NA | 5.03E-01 | 1.49E+00 | NA | NA | NA | NA |
| **1599** | 3.13E-02 | 3.50E-02 | 0.00E+00 | 1.60E+00 | 1.79E+00 | (0.0348,Inf] | (1.34,14.9] | NA | NA |
| **1600** | NA | NA | NA | 5.92E-02 | NA | NA | NA | NA | NA |
| **1601** | NA | NA | NA | 5.07E-01 | NA | NA | NA | NA | NA |
| **1602** | 2.79E-02 | 1.35E-02 | 2.45E+00 | 1.06E+00 | 5.15E-01 | (0.0348,Inf] | (1.34,14.9] | (0.0184,Inf] | (21,Inf] |
| **1603** | 1.23E-02 | 0.00E+00 | NA | 6.02E-01 | 0.00E+00 | NA | NA | NA | NA |
| **1604** | 0.00E+00 | 2.54E-03 | NA | 1.56E+00 | Inf | NA | NA | NA | NA |
| **1605** | 5.73E-03 | 0.00E+00 | NA | 2.88E+00 | 0.00E+00 | NA | NA | NA | NA |
| **1606** | NA | NA | NA | 7.30E-01 | NA | NA | NA | NA | NA |
| **1607** | 0.00E+00 | 3.35E-03 | NA | 2.78E-01 | Inf | NA | NA | NA | NA |
| **1608** | NA | NA | NA | 1.19E-01 | NA | NA | NA | NA | NA |
| **1609** | NA | NA | NA | 1.54E+00 | NA | NA | NA | NA | NA |
| **1610** | 1.74E-01 | 5.05E-01 | NA | 5.65E-01 | 1.64E+00 | NA | NA | NA | NA |
| **1611** | 0.00E+00 | 0.00E+00 | NA | 1.07E+00 | NA | NA | NA | NA | NA |
| **1612** | 6.34E-03 | 7.26E-02 | NA | 2.50E-01 | 2.87E+00 | NA | NA | NA | NA |
| **1613** | 0.00E+00 | 2.12E-02 | NA | 1.69E-01 | Inf | NA | NA | NA | NA |
| **1614** | NA | NA | NA | 4.69E-01 | NA | NA | NA | NA | NA |
| **1615** | 1.45E-02 | 0.00E+00 | NA | 6.39E-01 | 0.00E+00 | NA | NA | NA | NA |
| **1616** | 0.00E+00 | 7.76E-03 | NA | 7.67E-01 | Inf | NA | NA | NA | NA |
| **1617** | 4.18E-01 | 5.52E-01 | NA | 7.15E-01 | 9.44E-01 | NA | NA | NA | NA |
| **1618** | 5.30E-03 | 1.78E-03 | NA | 1.47E+00 | 4.94E-01 | NA | NA | NA | NA |
| **1619** | 0.00E+00 | 0.00E+00 | NA | 1.09E+00 | NA | NA | NA | NA | NA |
| **1620** | 4.27E-03 | 1.40E-02 | NA | 1.21E+00 | 3.96E+00 | NA | NA | NA | NA |
| **1621** | 1.62E-02 | 2.84E-03 | NA | 3.23E+00 | 5.68E-01 | NA | NA | NA | NA |
| **1622** | 1.78E-02 | 4.83E-02 | 9.19E-01 | 2.86E-01 | 7.76E-01 | (0.0348,Inf] | (1.34,14.9] | (0.0184,Inf] | (0.192,21] |
| **1623** | 0.00E+00 | 2.91E-02 | NA | 9.70E-01 | Inf | NA | NA | NA | NA |
| **1624** | NA | NA | Inf | 1.29E+00 | NA | NA | NA | (0.0184,Inf] | NA |
| **1625** | NA | NA | NA | 1.12E+00 | NA | NA | NA | NA | NA |
| **1626** | NA | NA | NA | 1.17E+00 | NA | NA | NA | NA | NA |
| **1627** | NA | NA | NA | 3.76E-01 | NA | NA | NA | NA | NA |
| **1628** | NA | NA | NA | 1.05E+00 | NA | NA | NA | NA | NA |
| **1629** | 3.63E-02 | 4.54E-02 | 0.00E+00 | 6.97E-01 | 8.73E-01 | (0.00294,0.0348] | (0.101,1.34] | NA | NA |
| **1630** | NA | NA | NA | 1.03E+00 | NA | NA | NA | NA | NA |

| | | | | | | | | | |
|---|---|---|---|---|---|---|---|---|---|
| **1631** | 1.39E-02 | 2.06E-02 | NA | 4.54E-01 | 6.69E-01 | NA | NA | NA | NA |
| **1632** | 1.29E-02 | 0.00E+00 | NA | 3.45E-01 | 0.00E+00 | NA | NA | NA | NA |
| **1633** | 1.22E-02 | 0.00E+00 | NA | 6.92E-01 | 0.00E+00 | NA | NA | NA | NA |
| **1634** | NA | NA | NA | 5.33E-01 | NA | NA | NA | NA | NA |
| **1635** | 1.23E-02 | 0.00E+00 | Inf | 1.51E+00 | 0.00E+00 | NA | NA | (0.0184,Inf] | (21,Inf] |
| **1636** | 0.00E+00 | 0.00E+00 | NA | 1.49E+00 | NA | NA | NA | NA | NA |
| **1637** | 0.00E+00 | 6.32E-02 | NA | 2.04E-01 | Inf | NA | NA | NA | NA |
| **1638** | NA | NA | NA | 1.85E+00 | NA | NA | NA | NA | NA |
| **1639** | 2.59E-03 | 1.40E-01 | NA | 8.46E-01 | 4.57E+01 | NA | NA | NA | NA |
| **1640** | NA | NA | NA | 1.68E+00 | NA | NA | NA | NA | NA |
| **1641** | 5.88E-03 | 8.31E-03 | NA | 3.17E-01 | 4.47E-01 | NA | NA | NA | NA |
| **1642** | 0.00E+00 | 1.43E-02 | NA | 5.65E-01 | Inf | NA | NA | NA | NA |
| **1643** | 1.10E-02 | 0.00E+00 | NA | 6.65E-01 | 0.00E+00 | NA | NA | NA | NA |
| **1644** | 4.45E-02 | 0.00E+00 | 2.22E+01 | 2.93E+00 | 0.00E+00 | (0.0348,Inf] | (1.34,14.9] | (0.0184,Inf] | (21,Inf] |
| **1645** | 1.57E-02 | 2.06E-03 | 0.00E+00 | 5.43E-01 | 7.13E-02 | (0.0348,Inf] | (1.34,14.9] | NA | NA |
| **1646** | Inf | 3.08E+00 | 2.32E+01 | Inf | 1.59E+02 | (0.0348,Inf] | (0.101,1.34] | (0.0184,Inf] | (0,0.192] |
| **1647** | 0.00E+00 | 0.00E+00 | NA | 3.09E-01 | NA | NA | NA | NA | NA |
| **1648** | NA | NA | NA | 1.27E-01 | NA | NA | NA | NA | NA |
| **1649** | 5.03E-02 | 1.32E-02 | NA | 1.48E+00 | 3.88E-01 | NA | NA | NA | NA |
| **1650** | NA | NA | NA | 8.13E-01 | NA | NA | NA | NA | NA |
| **1651** | 4.60E-03 | 6.52E-03 | NA | 5.27E-01 | 7.46E-01 | NA | NA | NA | NA |
| **1652** | 1.81E-02 | 2.70E-02 | NA | 4.77E-01 | 7.12E-01 | NA | NA | NA | NA |
| **1653** | NA | NA | 0.00E+00 | 8.78E-01 | NA | (0.0348,Inf] | NA | NA | NA |
| **1654** | 6.34E-03 | 0.00E+00 | NA | 1.03E+00 | 0.00E+00 | NA | NA | NA | NA |
| **1655** | NA | NA | NA | 1.32E+00 | NA | NA | NA | NA | NA |
| **1656** | 4.66E-02 | 4.44E-02 | 0.00E+00 | 1.69E+00 | 1.61E+00 | (0.0348,Inf] | (14.9,Inf] | NA | NA |
| **1657** | NA | NA | NA | 4.23E-01 | NA | NA | NA | NA | NA |
| **1658** | 7.19E-03 | 0.00E+00 | NA | 4.39E+00 | 0.00E+00 | NA | NA | NA | NA |
| **1659** | 2.02E-02 | 1.86E-02 | NA | 8.05E-01 | 7.42E-01 | NA | NA | NA | NA |
| **1660** | 1.54E-02 | 4.91E-03 | Inf | 9.48E-01 | 3.02E-01 | NA | NA | (0.0184,Inf] | (0.192,21] |
| **1661** | 0.00E+00 | 7.35E-03 | NA | 1.74E+00 | Inf | NA | NA | NA | NA |
| **1662** | 1.86E-02 | 0.00E+00 | NA | 4.56E-01 | 0.00E+00 | NA | NA | NA | NA |
| **1663** | 0.00E+00 | 1.32E-03 | NA | 8.46E-01 | Inf | NA | NA | NA | NA |
| **1664** | 1.67E-02 | 4.68E-02 | 0.00E+00 | 4.41E-01 | 1.24E+00 | (0.0348,Inf] | (1.34,14.9] | NA | NA |
| **1665** | 2.00E-02 | 0.00E+00 | NA | 1.89E+00 | 0.00E+00 | NA | NA | NA | NA |
| **1666** | NA | NA | NA | 9.50E-02 | NA | NA | NA | NA | NA |
| **1667** | 6.19E-03 | 3.26E-02 | 0.00E+00 | 1.90E+00 | 1.00E+01 | (0.0348,Inf] | (14.9,Inf] | NA | NA |
| **1668** | 2.35E-03 | 1.06E-02 | NA | 1.11E+00 | 4.99E+00 | NA | NA | NA | NA |

| | | | | | | | | | |
|---|---|---|---|---|---|---|---|---|---|
| **1669** | NA | NA | NA | 1.96E+00 | NA | NA | NA | NA | NA |
| **1670** | NA | NA | NA | 1.76E-01 | NA | NA | NA | NA | NA |
| **1671** | 6.50E-03 | 0.00E+00 | 0.00E+00 | 2.72E-01 | 0.00E+00 | (0.0348,Inf] | (1.34,14.9] | NA | NA |
| **1672** | 2.58E-02 | 6.74E-03 | NA | 8.10E-01 | 2.12E-01 | NA | NA | NA | NA |
| **1673** | 1.63E-02 | 2.26E-02 | 0.00E+00 | 1.12E+00 | 1.55E+00 | (0.0348,Inf] | (1.34,14.9] | NA | NA |
| **1674** | NA | NA | NA | 3.69E-01 | NA | NA | NA | NA | NA |
| **1675** | 0.00E+00 | 8.00E-03 | NA | 7.37E-01 | Inf | NA | NA | NA | NA |
| **1676** | 0.00E+00 | 0.00E+00 | NA | 1.55E+00 | NA | NA | NA | NA | NA |
| **1677** | 8.15E-03 | 6.51E-03 | NA | 9.42E-01 | 7.53E-01 | NA | NA | NA | NA |
| **1678** | NA | NA | NA | 3.55E-01 | NA | NA | NA | NA | NA |
| **1679** | 3.57E-02 | 0.00E+00 | NA | 8.95E-01 | 0.00E+00 | NA | NA | NA | NA |
| **1680** | NA | NA | NA | 4.54E-01 | NA | NA | NA | NA | NA |
| **1681** | 0.00E+00 | 0.00E+00 | NA | 1.75E-01 | NA | NA | NA | NA | NA |
| **1682** | 0.00E+00 | 2.15E-03 | NA | 1.53E+00 | Inf | NA | NA | NA | NA |
| **1683** | 1.59E-02 | 0.00E+00 | NA | 1.01E+00 | 0.00E+00 | NA | NA | NA | NA |
| **1684** | 1.40E-03 | 2.09E-02 | NA | 8.61E-01 | 1.28E+01 | NA | NA | NA | NA |
| **1685** | 1.09E-02 | 1.46E-02 | NA | 4.77E-01 | 6.37E-01 | NA | NA | NA | NA |
| **1686** | 0.00E+00 | 9.91E-03 | NA | 6.47E-01 | Inf | NA | NA | NA | NA |
| **1687** | 0.00E+00 | 0.00E+00 | NA | 4.22E-01 | NA | NA | NA | NA | NA |
| **1688** | 0.00E+00 | 0.00E+00 | Inf | 4.79E-01 | NA | NA | NA | (0.0184,Inf] | (21,Inf] |
| **1689** | NA | NA | 0.00E+00 | 2.01E-01 | NA | (0.0348,Inf] | NA | NA | NA |
| **1690** | 1.99E-02 | 0.00E+00 | NA | 1.27E+00 | 0.00E+00 | NA | NA | NA | NA |
| **1691** | 1.54E-02 | 3.92E-02 | 0.00E+00 | 4.04E-01 | 1.03E+00 | (0.00294,0.0348] | (0.101,1.34] | NA | NA |
| **1692** | 8.14E-03 | 4.21E-03 | NA | 6.00E-01 | 3.11E-01 | NA | NA | NA | NA |
| **1693** | 0.00E+00 | 1.03E-02 | NA | 7.51E-01 | Inf | NA | NA | NA | NA |
| **1694** | 1.04E-02 | 5.42E-02 | 6.49E-01 | 3.73E-01 | 1.95E+00 | (0.0348,Inf] | (14.9,Inf] | (0.0184,Inf] | (0.192,21] |
| **1695** | 4.36E-02 | 3.15E-02 | 1.52E+01 | 3.86E+00 | 2.79E+00 | (0.0348,Inf] | (14.9,Inf] | (0.0184,Inf] | (21,Inf] |
| **1696** | 0.00E+00 | 5.80E-03 | Inf | 8.48E-01 | Inf | NA | NA | (0.0184,Inf] | (0.192,21] |
| **1697** | 0.00E+00 | 0.00E+00 | NA | 1.16E+00 | NA | NA | NA | NA | NA |
| **1698** | 0.00E+00 | 3.91E-02 | NA | 5.10E-01 | Inf | NA | NA | NA | NA |
| **1699** | 3.85E-02 | 1.80E-01 | NA | 1.16E-01 | 5.44E-01 | NA | NA | NA | NA |
| **1700** | 2.45E-03 | 4.40E-03 | NA | 1.35E+00 | 2.42E+00 | NA | NA | NA | NA |
| **1701** | 1.02E-02 | 0.00E+00 | NA | 5.77E-01 | 0.00E+00 | NA | NA | NA | NA |
| **1702** | NA | NA | NA | 2.40E-01 | NA | NA | NA | NA | NA |
| **1703** | NA | NA | NA | 7.53E-01 | NA | NA | NA | NA | NA |
| **1704** | 6.45E-03 | 7.90E-02 | NA | 2.58E-01 | 3.16E+00 | NA | NA | NA | NA |
| **1705** | 4.01E-02 | 1.03E-02 | 0.00E+00 | 1.23E+00 | 3.19E-01 | (0.0348,Inf] | (1.34,14.9] | NA | NA |
| **1706** | 0.00E+00 | 2.80E-03 | NA | 1.78E+00 | Inf | NA | NA | NA | NA |

| | | | | | | | | | |
|---|---|---|---|---|---|---|---|---|---|
| **1707** | 2.19E-02 | 4.98E-03 | NA | 1.95E+00 | 4.43E-01 | NA | NA | NA | NA |
| **1708** | Inf | 2.94E-03 | NA | Inf | 1.75E-01 | NA | NA | NA | NA |
| **1709** | 4.17E-03 | 0.00E+00 | NA | 5.22E-01 | 0.00E+00 | NA | NA | NA | NA |
| **1710** | 1.20E-01 | 1.61E-02 | 1.24E+00 | 2.53E+00 | 3.41E-01 | (0.0348,Inf] | (1.34,14.9] | (0.0184,Inf] | (21,Inf] |
| **1711** | 0.00E+00 | 0.00E+00 | 1.31E+01 | 1.98E-01 | NA | (0.00294,0.0348] | (14.9,Inf] | (0.0184,Inf] | (21,Inf] |
| **1712** | 3.22E-02 | 5.24E-02 | NA | 3.61E-01 | 5.87E-01 | NA | NA | NA | NA |
| **1713** | 3.04E-02 | 1.36E-02 | 0.00E+00 | 2.62E+00 | 1.17E+00 | (0.0348,Inf] | (14.9,Inf] | NA | NA |
| **1714** | NA | NA | NA | 1.03E+00 | NA | NA | NA | NA | NA |
| **1715** | 0.00E+00 | 0.00E+00 | 0.00E+00 | 1.73E+00 | NA | (0.0348,Inf] | (14.9,Inf] | NA | NA |
| **1716** | 2.61E-02 | 0.00E+00 | NA | 3.83E-01 | 0.00E+00 | NA | NA | NA | NA |
| **1717** | 0.00E+00 | 4.18E-02 | NA | 4.06E-01 | Inf | NA | NA | NA | NA |
| **1718** | 1.84E-02 | 0.00E+00 | NA | 2.28E+00 | 0.00E+00 | NA | NA | NA | NA |
| **1719** | NA | NA | NA | 2.24E-01 | NA | NA | NA | NA | NA |
| **1720** | NA | NA | NA | 1.23E+00 | NA | NA | NA | NA | NA |
| **1721** | NA | NA | 3.75E-01 | NA | 1.64E+00 | NA | (0,0.101] | NA | (0,0.192] |
| **1722** | 3.11E-02 | 2.16E-02 | NA | 6.24E-01 | 4.33E-01 | NA | NA | NA | NA |
| **1723** | 7.90E-02 | 3.39E-02 | NA | 1.51E+00 | 6.45E-01 | NA | NA | NA | NA |
| **1724** | 7.53E-03 | 0.00E+00 | NA | 2.10E-01 | 0.00E+00 | NA | NA | NA | NA |
| **1725** | 5.07E-02 | 1.67E-03 | NA | 1.94E+00 | 6.37E-02 | NA | NA | NA | NA |
| **1726** | NA | NA | 0.00E+00 | 2.62E+00 | NA | (0.0348,Inf] | NA | NA | NA |
| **1727** | 3.24E-03 | 1.61E-02 | Inf | 9.69E-01 | 4.82E+00 | NA | NA | (0.000255,0.0184] | (0.192,21] |
| **1728** | NA | NA | 0.00E+00 | NA | 5.40E-01 | NA | (0.101,1.34] | NA | NA |
| **1729** | NA | NA | NA | 3.30E-01 | NA | NA | NA | NA | NA |
| **1730** | 7.69E-03 | 0.00E+00 | NA | 3.70E-01 | 0.00E+00 | NA | NA | NA | NA |
| **1731** | NA | NA | Inf | NA | NA | NA | NA | NA | (21,Inf] |
| **1732** | NA | NA | Inf | NA | NA | NA | NA | NA | (21,Inf] |
| **1733** | NA | NA | NA | 1.49E+00 | NA | NA | NA | NA | NA |
| **1734** | 8.61E-03 | 4.97E-03 | 0.00E+00 | 7.75E-01 | 4.48E-01 | (0.0348,Inf] | (1.34,14.9] | NA | NA |
| **1735** | 1.00E-02 | 1.20E-02 | 0.00E+00 | 8.17E-01 | 9.79E-01 | (0.0348,Inf] | (1.34,14.9] | NA | NA |
| **1736** | 0.00E+00 | 0.00E+00 | Inf | 1.57E+00 | NA | NA | NA | (0.0184,Inf] | (21,Inf] |
| **1737** | NA | NA | NA | 1.04E+00 | NA | NA | NA | NA | NA |
| **1738** | 9.83E-02 | 1.97E-02 | NA | 2.75E+00 | 5.51E-01 | NA | NA | NA | NA |
| **1739** | 3.16E+00 | 0.00E+00 | NA | 4.00E-01 | 0.00E+00 | NA | NA | NA | NA |
| **1740** | 3.12E-01 | 7.61E-02 | 0.00E+00 | 3.08E+00 | 7.52E-01 | (0.0348,Inf] | (1.34,14.9] | NA | NA |
| **1741** | 4.00E-02 | 0.00E+00 | NA | 3.40E+00 | 0.00E+00 | NA | NA | NA | NA |
| **1742** | 1.41E-01 | 4.75E-02 | 0.00E+00 | 5.01E-01 | 1.68E-01 | (0.0348,Inf] | (1.34,14.9] | NA | NA |
| **1743** | NA | NA | 0.00E+00 | 1.41E-01 | NA | (0.0348,Inf] | NA | NA | NA |
| **1744** | 5.87E-02 | 0.00E+00 | NA | 1.70E+00 | 0.00E+00 | NA | NA | NA | NA |

| | | | | | | | | | |
|---|---|---|---|---|---|---|---|---|---|
| **1745** | NA | NA | NA | 2.23E-01 | NA | NA | NA | NA | NA |
| **1746** | NA | NA | NA | 7.35E-01 | NA | NA | NA | NA | NA |
| **1747** | NA | NA | NA | 6.32E-01 | NA | NA | NA | NA | NA |
| **1748** | 0.00E+00 | 0.00E+00 | NA | 4.37E-01 | NA | NA | NA | NA | NA |
| **1749** | Inf | 2.01E-03 | NA | Inf | 4.06E-01 | NA | NA | NA | NA |
| **1750** | 1.22E+00 | 0.00E+00 | NA | 8.83E+00 | 0.00E+00 | NA | NA | NA | NA |
| **1751** | 0.00E+00 | 1.58E-02 | NA | 9.32E-01 | Inf | NA | NA | NA | NA |
| **1752** | 0.00E+00 | 2.24E-02 | NA | 6.71E-01 | Inf | NA | NA | NA | NA |
| **1753** | 0.00E+00 | 1.96E-02 | NA | 1.59E-01 | Inf | NA | NA | NA | NA |
| **1754** | 1.09E-01 | 0.00E+00 | NA | 4.31E+00 | 0.00E+00 | NA | NA | NA | NA |
| **1755** | NA | NA | NA | 3.89E-01 | NA | NA | NA | NA | NA |
| **1756** | 3.79E-02 | 2.13E-03 | NA | 1.33E+00 | 7.47E-02 | NA | NA | NA | NA |
| **1757** | NA | NA | NA | 9.50E-01 | NA | NA | NA | NA | NA |
| **1758** | NA | NA | NA | 2.94E-01 | NA | NA | NA | NA | NA |
| **1759** | 0.00E+00 | 0.00E+00 | NA | 5.25E-01 | NA | NA | NA | NA | NA |
| **1760** | 3.04E-03 | 5.88E-02 | 4.50E-01 | 8.02E-01 | 1.55E+01 | (0.0348,Inf] | (14.9,Inf] | (0.0184,Inf] | (0.192,21] |
| **1761** | 0.00E+00 | 0.00E+00 | NA | 4.09E-01 | NA | NA | NA | NA | NA |
| **1762** | NA | NA | NA | 1.07E+00 | NA | NA | NA | NA | NA |
| **1763** | 1.43E-02 | 0.00E+00 | NA | 5.00E-01 | 0.00E+00 | NA | NA | NA | NA |
| **1764** | NA | NA | NA | 1.08E+00 | NA | NA | NA | NA | NA |
| **1765** | 1.28E-02 | 0.00E+00 | 7.92E-01 | 2.59E-01 | 0.00E+00 | (0.0348,Inf] | (14.9,Inf] | (0.0184,Inf] | (21,Inf] |
| **1766** | 1.76E-02 | 2.58E-03 | NA | 1.12E+00 | 1.64E-01 | NA | NA | NA | NA |
| **1767** | NA | NA | NA | 1.81E+00 | NA | NA | NA | NA | NA |
| **1768** | NA | NA | NA | 1.01E+00 | NA | NA | NA | NA | NA |
| **1769** | 0.00E+00 | 0.00E+00 | NA | 4.54E-01 | NA | NA | NA | NA | NA |
| **1770** | 5.23E-02 | 0.00E+00 | NA | 1.16E+00 | 0.00E+00 | NA | NA | NA | NA |
| **1771** | NA | NA | NA | 3.06E-01 | NA | NA | NA | NA | NA |
| **1772** | 5.83E-02 | 8.26E-03 | Inf | 3.94E+00 | 5.58E-01 | NA | NA | (0.0184,Inf] | (0.192,21] |
| **1773** | 2.65E-02 | 1.20E-02 | 8.17E-02 | 1.75E+00 | 7.95E-01 | (0.0348,Inf] | (1.34,14.9] | (0.000255,0.0184] | (0.192,21] |
| **1774** | NA | NA | NA | 5.00E-01 | NA | NA | NA | NA | NA |
| **1775** | NA | NA | NA | 7.70E-01 | NA | NA | NA | NA | NA |
| **1776** | NA | NA | NA | 3.56E+00 | NA | NA | NA | NA | NA |
| **1777** | 6.12E-03 | 0.00E+00 | NA | 2.28E-01 | 0.00E+00 | NA | NA | NA | NA |
| **1778** | 0.00E+00 | 0.00E+00 | NA | 3.12E-01 | NA | NA | NA | NA | NA |
| **1779** | 1.63E-02 | 0.00E+00 | NA | 7.28E-01 | 0.00E+00 | NA | NA | NA | NA |
| **1780** | 3.64E-02 | 2.65E-02 | NA | 9.99E-01 | 7.28E-01 | NA | NA | NA | NA |
| **1781** | NA | NA | NA | NA | 1.26E+00 | NA | NA | NA | NA |
| **1782** | 0.00E+00 | 0.00E+00 | NA | 2.44E-01 | NA | NA | NA | NA | NA |

| | | | | | | | | | |
|---|---|---|---|---|---|---|---|---|---|
| **1783** | NA | NA | NA | 4.29E-01 | NA | NA | NA | NA | NA |
| **1784** | 1.21E-01 | 2.13E-03 | NA | 4.02E+00 | 7.07E-02 | NA | NA | NA | NA |
| **1785** | 0.00E+00 | 1.67E-02 | NA | 9.99E-01 | Inf | NA | NA | NA | NA |
| **1786** | 1.45E-02 | 3.18E-02 | 2.98E+00 | 2.84E-01 | 6.24E-01 | (0.0348,Inf] | (14.9,Inf] | (0.0184,Inf] | (21,Inf] |
| **1787** | 0.00E+00 | 0.00E+00 | 1.93E+00 | 1.49E-01 | NA | (0.0348,Inf] | (14.9,Inf] | (0.0184,Inf] | (21,Inf] |
| **1788** | 6.95E-03 | 5.80E-02 | 3.89E-01 | 2.93E+00 | 2.45E+01 | (0.0348,Inf] | (14.9,Inf] | (0.0184,Inf] | (0.192,21] |
| **1789** | 1.96E-02 | 7.87E-02 | NA | 7.29E-01 | 2.93E+00 | NA | NA | NA | NA |
| **1790** | NA | NA | NA | 3.53E-01 | NA | NA | NA | NA | NA |
| **1791** | 1.38E-02 | 2.49E-02 | NA | 1.25E+00 | 2.26E+00 | NA | NA | NA | NA |
| **1792** | NA | NA | NA | 2.54E+00 | NA | NA | NA | NA | NA |
| **1793** | NA | NA | Inf | NA | NA | NA | NA | NA | (21,Inf] |
| **1794** | NA | NA | Inf | NA | NA | NA | NA | NA | (21,Inf] |
| **1795** | 0.00E+00 | 1.26E-01 | NA | 1.46E+00 | Inf | NA | NA | NA | NA |
| **1796** | NA | NA | NA | 1.79E+00 | NA | NA | NA | NA | NA |
| **1797** | NA | NA | NA | 8.73E-01 | NA | NA | NA | NA | NA |
| **1798** | 4.67E-02 | 1.42E-02 | NA | 1.26E+00 | 3.83E-01 | NA | NA | NA | NA |
| **1799** | 8.42E-02 | 0.00E+00 | NA | 3.24E+00 | 0.00E+00 | NA | NA | NA | NA |
| **1800** | NA | NA | NA | 3.09E-01 | NA | NA | NA | NA | NA |
| **1801** | NA | NA | NA | 2.49E+00 | NA | NA | NA | NA | NA |
| **1802** | 0.00E+00 | 1.00E-02 | NA | 9.32E-01 | Inf | NA | NA | NA | NA |
| **1803** | NA | NA | NA | 2.07E+00 | NA | NA | NA | NA | NA |
| **1804** | 0.00E+00 | 2.46E-03 | NA | 1.57E+00 | Inf | NA | NA | NA | NA |
| **1805** | 2.46E-02 | 9.80E-02 | 4.23E-02 | 2.59E-01 | 1.03E+00 | (0.0348,Inf] | (1.34,14.9] | (0.0184,Inf] | (0.192,21] |
| **1806** | 1.71E-03 | 0.00E+00 | NA | 1.19E+00 | 0.00E+00 | NA | NA | NA | NA |
| **1807** | NA | NA | NA | 8.92E-01 | NA | NA | NA | NA | NA |
| **1808** | 1.41E-03 | 0.00E+00 | 7.73E+01 | 3.48E-01 | 0.00E+00 | (0.0348,Inf] | (14.9,Inf] | (0.0184,Inf] | (21,Inf] |
| **1809** | NA | NA | NA | 1.45E+00 | NA | NA | NA | NA | NA |
| **1810** | 2.98E-02 | 0.00E+00 | NA | 1.01E+00 | 0.00E+00 | NA | NA | NA | NA |
| **1811** | 3.82E-02 | 1.05E-02 | NA | 4.14E-01 | 1.13E-01 | NA | NA | NA | NA |
| **1812** | NA | NA | NA | 1.03E+00 | NA | NA | NA | NA | NA |
| **1813** | NA | NA | NA | 0.00E+00 | NA | NA | NA | NA | NA |
| **1814** | 6.57E-03 | 0.00E+00 | NA | 3.30E-01 | 0.00E+00 | NA | NA | NA | NA |
| **1815** | NA | NA | Inf | NA | NA | NA | NA | NA | NA |
| **1816** | NA | NA | NA | 8.39E-01 | NA | NA | NA | NA | NA |
| **1817** | NA | NA | NA | 3.99E-01 | NA | NA | NA | NA | NA |
| **1818** | 0.00E+00 | 0.00E+00 | NA | 8.52E-02 | NA | NA | NA | NA | NA |
| **1819** | NA | NA | NA | 4.65E-01 | NA | NA | NA | NA | NA |
| **1820** | 0.00E+00 | 3.56E-02 | NA | 2.63E-01 | Inf | NA | NA | NA | NA |

| | | | | | | | | | | |
|---|---|---|---|---|---|---|---|---|---|---|
| **1821** | NA | NA | NA | 3.64E-01 | NA | NA | NA | NA | NA |
| **1822** | NA | NA | NA | 1.70E+00 | NA | NA | NA | NA | NA |
| **1823** | 3.57E-02 | 0.00E+00 | NA | 1.77E+00 | 0.00E+00 | NA | NA | NA | NA |
| **1824** | 8.90E-03 | 6.28E-03 | NA | 1.63E+00 | 1.15E+00 | NA | NA | NA | NA |
| **1825** | NA | NA | NA | 1.47E+00 | NA | NA | NA | NA | NA |
| **1826** | 1.84E-02 | 1.76E-02 | NA | 9.94E-01 | 9.52E-01 | NA | NA | NA | NA |
| **1827** | 0.00E+00 | 1.24E-01 | NA | 1.97E-01 | Inf | NA | NA | NA | NA |
| **1828** | 1.44E-02 | 0.00E+00 | NA | 2.28E-01 | 0.00E+00 | NA | NA | NA | NA |
| **1829** | NA | NA | NA | 1.12E+00 | NA | NA | NA | NA | NA |
| **1830** | 3.73E-02 | 9.83E-02 | NA | 3.48E-01 | 9.18E-01 | NA | NA | NA | NA |
| **1831** | NA | NA | NA | 3.51E-01 | NA | NA | NA | NA | NA |
| **1832** | NA | NA | NA | 1.10E+00 | NA | NA | NA | NA | NA |
| **1833** | NA | NA | NA | 6.11E-01 | NA | NA | NA | NA | NA |
| **1834** | 1.37E-02 | 2.27E-02 | NA | 1.57E-01 | 2.60E-01 | NA | NA | NA | NA |
| **1835** | 6.70E-02 | 7.69E-03 | NA | 2.25E+00 | 2.58E-01 | NA | NA | NA | NA |
| **1836** | NA | NA | NA | 7.45E-01 | NA | NA | NA | NA | NA |
| **1837** | 0.00E+00 | 1.43E-02 | NA | 7.39E-01 | Inf | NA | NA | NA | NA |
| **1838** | NA | NA | NA | 4.05E-01 | NA | NA | NA | NA | NA |
| **1839** | NA | NA | NA | 1.87E+00 | NA | NA | NA | NA | NA |
| **1840** | 1.64E-02 | 8.42E-02 | NA | 2.27E-01 | 1.17E+00 | NA | NA | NA | NA |
| **1841** | 0.00E+00 | 2.12E-02 | Inf | 2.56E-01 | Inf | NA | NA | (0.0184,Inf] | (0.192,21] |
| **1842** | 2.01E-02 | 0.00E+00 | NA | 4.56E-01 | 0.00E+00 | NA | NA | NA | NA |
| **1843** | NA | NA | Inf | NA | NA | NA | NA | NA | NA |
| **1844** | NA | NA | NA | 4.25E-01 | NA | NA | NA | NA | NA |
| **1845** | 0.00E+00 | 0.00E+00 | NA | 4.86E-01 | NA | NA | NA | NA | NA |
| **1846** | 4.95E-03 | Inf | NA | 0.00E+00 | 3.92E-01 | NA | NA | NA | NA |
| **1847** | NA | NA | NA | 1.44E+00 | NA | NA | NA | NA | NA |
| **1848** | NA | NA | NA | 0.00E+00 | NA | NA | NA | NA | NA |
| **1849** | 0.00E+00 | 3.90E-02 | 0.00E+00 | 2.39E-01 | Inf | (0.0348,Inf] | (14.9,Inf] | NA | NA |
| **1850** | 0.00E+00 | 0.00E+00 | NA | 8.14E-01 | NA | NA | NA | NA | NA |
| **1851** | NA | NA | NA | 1.37E+00 | NA | NA | NA | NA | NA |
| **1852** | 0.00E+00 | 0.00E+00 | NA | 3.47E+00 | NA | NA | NA | NA | NA |
| **1853** | 7.16E-03 | 2.58E-02 | NA | 6.35E-01 | 2.29E+00 | NA | NA | NA | NA |
| **1854** | Inf | 0.00E+00 | NA | Inf | 0.00E+00 | NA | NA | NA | NA |
| **1855** | 0.00E+00 | 0.00E+00 | Inf | 5.72E-01 | NA | NA | NA | (0.0184,Inf] | (21,Inf] |
| **1856** | NA | NA | NA | 2.51E-01 | NA | NA | NA | NA | NA |
| **1857** | 0.00E+00 | 7.34E-03 | 0.00E+00 | 6.26E-01 | Inf | (0.0348,Inf] | (14.9,Inf] | NA | NA |
| **1858** | 5.86E-02 | 8.46E-02 | NA | 1.29E+00 | 1.86E+00 | NA | NA | NA | NA |

| | | | | | | | | | |
|---|---|---|---|---|---|---|---|---|---|
| **1859** | 7.08E-03 | 1.14E-02 | NA | 5.78E-01 | 9.30E-01 | NA | NA | NA | NA |
| **1860** | NA | NA | NA | 5.80E-01 | NA | NA | NA | NA | NA |
| **1861** | NA | NA | NA | 2.41E+00 | NA | NA | NA | NA | NA |
| **1862** | NA | NA | NA | 0.00E+00 | NA | NA | NA | NA | NA |
| **1863** | NA | NA | NA | Inf | NA | NA | NA | NA | NA |
| **1864** | 9.56E-03 | Inf | NA | 0.00E+00 | 2.46E+00 | NA | NA | NA | NA |
| **1865** | 2.42E-02 | 0.00E+00 | Inf | 1.72E-01 | 0.00E+00 | NA | NA | (0.0184,Inf] | (21,Inf] |
| **1866** | 4.29E-02 | 0.00E+00 | NA | 4.54E-01 | 0.00E+00 | NA | NA | NA | NA |
| **1867** | NA | NA | 0.00E+00 | NA | 1.18E+00 | NA | (0.101,1.34] | NA | NA |
| **1868** | 6.70E-03 | 7.74E-03 | NA | 2.55E+00 | 2.94E+00 | NA | NA | NA | NA |
| **1869** | 0.00E+00 | 1.24E-02 | NA | 6.00E-01 | Inf | NA | NA | NA | NA |
| **1870** | 9.73E-03 | 0.00E+00 | NA | 4.40E-01 | 0.00E+00 | NA | NA | NA | NA |
| **1871** | NA | NA | NA | 5.86E-01 | NA | NA | NA | NA | NA |
| **1872** | 4.61E-02 | 3.29E-02 | 0.00E+00 | 1.01E+00 | 7.23E-01 | (0.0348,Inf] | (1.34,14.9] | NA | NA |
| **1873** | 3.32E-02 | 0.00E+00 | NA | 4.23E-01 | 0.00E+00 | NA | NA | NA | NA |
| **1874** | NA | 7.51E-03 | 0.00E+00 | Inf | Inf | (0.0348,Inf] | (14.9,Inf] | NA | NA |
| **1875** | 1.90E-02 | 2.13E-02 | NA | 2.53E+00 | 2.83E+00 | NA | NA | NA | NA |
| **1876** | NA | NA | NA | 7.55E-01 | NA | NA | NA | NA | NA |
| **1877** | 0.00E+00 | 0.00E+00 | NA | 1.30E+00 | NA | NA | NA | NA | NA |
| **1878** | NA | NA | NA | 7.79E-01 | NA | NA | NA | NA | NA |
| **1879** | 7.29E-03 | 0.00E+00 | NA | 1.44E+00 | 0.00E+00 | NA | NA | NA | NA |
| **1880** | NA | NA | NA | 2.56E-01 | NA | NA | NA | NA | NA |
| **1881** | NA | NA | NA | 0.00E+00 | NA | NA | NA | NA | NA |
| **1882** | 0.00E+00 | 8.99E-02 | NA | 1.48E-01 | Inf | NA | NA | NA | NA |
| **1883** | NA | NA | Inf | 7.19E-01 | NA | NA | NA | (0.0184,Inf] | NA |
| **1884** | 1.22E-01 | 3.00E-02 | NA | 2.06E+00 | 5.10E-01 | NA | NA | NA | NA |
| **1885** | 2.75E-02 | 0.00E+00 | NA | 1.19E+00 | 0.00E+00 | NA | NA | NA | NA |
| **1886** | NA | NA | NA | 1.35E+00 | NA | NA | NA | NA | NA |
| **1887** | NA | NA | NA | 7.07E-01 | NA | NA | NA | NA | NA |
| **1888** | NA | NA | NA | 1.39E+00 | NA | NA | NA | NA | NA |
| **1889** | 1.41E-02 | 2.69E-02 | NA | 9.93E-01 | 1.90E+00 | NA | NA | NA | NA |
| **1890** | 9.40E-02 | 3.56E-02 | 0.00E+00 | 3.46E+00 | 1.31E+00 | (0.0348,Inf] | (1.34,14.9] | NA | NA |
| **1891** | NA | NA | NA | 3.28E-01 | NA | NA | NA | NA | NA |
| **1892** | NA | NA | NA | 8.39E+00 | NA | NA | NA | NA | NA |
| **1893** | 4.10E-02 | 0.00E+00 | NA | 7.69E-01 | 0.00E+00 | NA | NA | NA | NA |
| **1894** | 0.00E+00 | 0.00E+00 | NA | 2.23E-01 | NA | NA | NA | NA | NA |
| **1895** | 1.02E-02 | 0.00E+00 | NA | 7.65E-01 | 0.00E+00 | NA | NA | NA | NA |
| **1896** | NA | NA | NA | 1.61E-01 | NA | NA | NA | NA | NA |

| | | | | | | | | | |
|---|---|---|---|---|---|---|---|---|---|
| **1897** | NA | NA | NA | 3.57E-01 | NA | NA | NA | NA | NA |
| **1898** | NA | NA | Inf | NA | 9.55E-01 | NA | NA | NA | (0.192,21] |
| **1899** | 5.64E-02 | 3.05E-02 | NA | 2.78E-01 | 1.50E-01 | NA | NA | NA | NA |
| **1900** | NA | NA | NA | 0.00E+00 | NA | NA | NA | NA | NA |
| **1901** | NA | NA | NA | 3.87E-01 | NA | NA | NA | NA | NA |
| **1902** | NA | NA | NA | 6.85E-01 | NA | NA | NA | NA | NA |
| **1903** | 3.17E-02 | 1.91E-01 | 0.00E+00 | 6.45E-01 | 3.89E+00 | (0.0348,Inf] | (14.9,Inf] | NA | NA |
| **1904** | 0.00E+00 | 1.75E-01 | 0.00E+00 | 1.75E-01 | Inf | (0.0348,Inf] | (14.9,Inf] | NA | NA |
| **1905** | 3.59E-02 | 0.00E+00 | NA | 7.07E-01 | 0.00E+00 | NA | NA | NA | NA |
| **1906** | NA | NA | NA | 6.67E-01 | NA | NA | NA | NA | NA |
| **1907** | NA | NA | NA | 7.69E-01 | NA | NA | NA | NA | NA |
| **1908** | NA | NA | Inf | NA | NA | NA | NA | NA | NA |
| **1909** | NA | NA | NA | 6.40E-02 | NA | NA | NA | NA | NA |
| **1910** | NA | NA | NA | 8.05E-01 | NA | NA | NA | NA | NA |
| **1911** | 0.00E+00 | 1.16E-02 | NA | 6.08E-01 | Inf | NA | NA | NA | NA |
| **1912** | 1.57E-02 | 1.14E-02 | NA | 1.63E+00 | 1.19E+00 | NA | NA | NA | NA |
| **1913** | 6.41E-02 | 0.00E+00 | NA | 1.86E+00 | 0.00E+00 | NA | NA | NA | NA |
| **1914** | 2.96E-02 | 2.28E-02 | 4.52E+01 | 6.62E-01 | 5.09E-01 | (0.0348,Inf] | (1.34,14.9] | (0.0184,Inf] | (21,Inf] |
| **1915** | 0.00E+00 | Inf | 6.65E+00 | 0.00E+00 | Inf | (0.0348,Inf] | (14.9,Inf] | (0.0184,Inf] | (21,Inf] |
| **1916** | 1.11E-01 | 3.37E-02 | 0.00E+00 | 1.11E+00 | 3.40E-01 | (0.0348,Inf] | (0.101,1.34] | NA | NA |
| **1917** | 0.00E+00 | 0.00E+00 | Inf | 1.02E+00 | NA | NA | NA | (0.0184,Inf] | (21,Inf] |
| **1918** | 0.00E+00 | Inf | NA | 0.00E+00 | Inf | NA | NA | NA | NA |
| **1919** | NA | NA | NA | 1.78E-01 | NA | NA | NA | NA | NA |
| **1920** | 3.42E-02 | Inf | NA | 0.00E+00 | 1.59E+00 | NA | NA | NA | NA |
| **1921** | 1.77E-02 | Inf | Inf | 0.00E+00 | 5.89E-01 | NA | NA | (0.0184,Inf] | (0.192,21] |
| **1922** | 0.00E+00 | 0.00E+00 | Inf | 3.35E+00 | NA | NA | NA | (0.0184,Inf] | (21,Inf] |
| **1923** | 0.00E+00 | NA | NA | 0.00E+00 | NA | NA | NA | NA | NA |
| **1924** | 2.98E-02 | 0.00E+00 | 8.98E-01 | 2.54E+00 | 0.00E+00 | (0.0348,Inf] | (14.9,Inf] | (0.0184,Inf] | (21,Inf] |
| **1925** | 0.00E+00 | 5.02E-02 | NA | 8.31E-01 | Inf | NA | NA | NA | NA |
| **1926** | 6.21E-01 | 4.96E-02 | 0.00E+00 | 2.67E+00 | 2.13E-01 | (0.0348,Inf] | (0.101,1.34] | NA | NA |
| **1927** | NA | NA | Inf | NA | NA | NA | NA | NA | (21,Inf] |
| **1928** | NA | NA | NA | 3.28E-01 | NA | NA | NA | NA | NA |
| **1929** | 4.37E-01 | 0.00E+00 | NA | 1.63E+00 | 0.00E+00 | NA | NA | NA | NA |
| **1930** | NA | NA | NA | 1.73E-01 | NA | NA | NA | NA | NA |
| **1931** | 8.38E-03 | 0.00E+00 | NA | 5.30E-01 | 0.00E+00 | NA | NA | NA | NA |
| **1932** | Inf | 0.00E+00 | NA | Inf | 0.00E+00 | NA | NA | NA | NA |
| **1933** | NA | NA | Inf | NA | NA | NA | NA | NA | (21,Inf] |
| **1934** | 4.93E-02 | 3.09E-02 | NA | 2.21E+00 | 1.38E+00 | NA | NA | NA | NA |

| | | | | | | | | | |
|---|---|---|---|---|---|---|---|---|---|
| **1935** | 4.42E-02 | 0.00E+00 | NA | 1.17E+00 | 0.00E+00 | NA | NA | NA | NA |
| **1936** | 8.09E-02 | 0.00E+00 | NA | 7.82E-01 | 0.00E+00 | NA | NA | NA | NA |
| **1937** | 2.95E-03 | 1.34E+00 | 1.60E+00 | 1.31E-01 | 5.94E+01 | (0.0348,Inf] | (14.9,Inf] | (0.0184,Inf] | (0.192,21] |
| **1938** | 1.99E-02 | 1.99E-02 | NA | 1.01E+00 | 1.01E+00 | NA | NA | NA | NA |
| **1939** | NA | NA | NA | 3.43E-01 | NA | NA | NA | NA | NA |
| **1940** | 0.00E+00 | 0.00E+00 | NA | 5.71E-01 | NA | NA | NA | NA | NA |
| **1941** | 1.59E-02 | 0.00E+00 | NA | 1.30E+00 | 0.00E+00 | NA | NA | NA | NA |
| **1942** | NA | NA | NA | 2.84E+00 | NA | NA | NA | NA | NA |
| **1943** | NA | NA | NA | 6.31E-01 | NA | NA | NA | NA | NA |
| **1944** | NA | NA | NA | 0.00E+00 | NA | NA | NA | NA | NA |
| **1945** | 0.00E+00 | 0.00E+00 | NA | 2.09E-01 | NA | NA | NA | NA | NA |
| **1946** | NA | NA | Inf | NA | NA | NA | NA | NA | NA |
| **1947** | NA | NA | NA | 0.00E+00 | NA | NA | NA | NA | NA |
| **1948** | NA | NA | NA | 7.23E-01 | NA | NA | NA | NA | NA |
| **1949** | 0.00E+00 | 2.03E-02 | NA | 1.02E+00 | Inf | NA | NA | NA | NA |
| **1950** | NA | NA | Inf | NA | NA | NA | NA | NA | NA |
| **1951** | 9.14E-03 | Inf | NA | 0.00E+00 | 1.75E+00 | NA | NA | NA | NA |
| **1952** | NA | NA | NA | 0.00E+00 | NA | NA | NA | NA | NA |
| **1953** | NA | NA | NA | Inf | NA | NA | NA | NA | NA |
| **1954** | 5.58E-03 | 2.41E-02 | NA | 1.54E+00 | 6.62E+00 | NA | NA | NA | NA |
| **1955** | 0.00E+00 | 5.52E-03 | NA | 8.12E+00 | Inf | NA | NA | NA | NA |
| **1956** | NA | NA | NA | NA | Inf | NA | NA | NA | NA |
| **1957** | NA | 5.53E-02 | 0.00E+00 | Inf | Inf | (0.0348,Inf] | (14.9,Inf] | NA | NA |
| **1958** | 0.00E+00 | NA | NA | 0.00E+00 | NA | NA | NA | NA | NA |
| **1959** | 0.00E+00 | 1.51E-01 | 3.22E-02 | 2.87E-01 | Inf | (0.0348,Inf] | (14.9,Inf] | (0.0184,Inf] | (0.192,21] |
| **1960** | 0.00E+00 | Inf | NA | 0.00E+00 | Inf | NA | NA | NA | NA |
| **1961** | NA | NA | NA | 0.00E+00 | NA | NA | NA | NA | NA |
| **1962** | NA | NA | NA | 1.68E-01 | NA | NA | NA | NA | NA |
| **1963** | NA | NA | NA | 8.10E-01 | NA | NA | NA | NA | NA |
| **1964** | NA | NA | NA | 7.37E-01 | NA | NA | NA | NA | NA |
| **1965** | NA | NA | NA | 8.69E-01 | NA | NA | NA | NA | NA |
| **1966** | 0.00E+00 | 0.00E+00 | NA | 1.91E-01 | NA | NA | NA | NA | NA |
| **1967** | 2.78E-02 | 7.14E-02 | 5.70E+00 | 1.60E+00 | 4.11E+00 | (0.0348,Inf] | (1.34,14.9] | (0.0184,Inf] | (0.192,21] |
| **1968** | NA | NA | NA | 5.51E-01 | NA | NA | NA | NA | NA |
| **1969** | NA | NA | NA | 2.17E-01 | NA | NA | NA | NA | NA |
| **1970** | 3.01E-02 | 1.25E-01 | 0.00E+00 | 1.73E+00 | 7.17E+00 | (0.0348,Inf] | (14.9,Inf] | NA | NA |
| **1971** | NA | 7.12E-03 | 0.00E+00 | Inf | Inf | (0.0348,Inf] | (14.9,Inf] | NA | NA |
| **1972** | NA | NA | Inf | NA | NA | NA | NA | NA | (21,Inf] |

| | | | | | | | | | |
|---|---|---|---|---|---|---|---|---|---|
| **1973** | 3.42E-02 | 4.83E-01 | 1.36E-02 | 2.57E-01 | 3.63E+00 | (0.0348,Inf] | (14.9,Inf] | (0.0184,Inf] | (0.192,21] |
| **1974** | NA | NA | NA | NA | Inf | NA | NA | NA | NA |
| **1975** | NA | NA | 0.00E+00 | NA | 2.37E+01 | NA | (14.9,Inf] | NA | NA |
| **1976** | NA | NA | NA | 3.43E-01 | NA | NA | NA | NA | NA |
| **1977** | NA | NA | NA | NA | 8.88E-01 | NA | NA | NA | NA |
| **1978** | NA | NA | Inf | NA | NA | NA | NA | NA | (21,Inf] |
| **1979** | Inf | 0.00E+00 | NA | Inf | 0.00E+00 | NA | NA | NA | NA |
| **1980** | 9.91E-02 | 0.00E+00 | NA | 1.54E+00 | 0.00E+00 | NA | NA | NA | NA |
| **1981** | 0.00E+00 | 1.11E-01 | NA | 3.64E-01 | Inf | NA | NA | NA | NA |
| **1982** | NA | NA | Inf | NA | NA | NA | NA | NA | (21,Inf] |
| **1983** | NA | NA | NA | 1.11E+01 | NA | NA | NA | NA | NA |
| **1984** | NA | NA | Inf | NA | NA | NA | NA | NA | (21,Inf] |
| **1985** | NA | NA | NA | 3.13E+00 | NA | NA | NA | NA | NA |
| **1986** | 1.14E-01 | 8.64E-02 | 0.00E+00 | 7.97E-01 | 6.03E-01 | (0.0348,Inf] | (1.34,14.9] | NA | NA |
| **1987** | Inf | 2.88E-02 | Inf | Inf | 3.46E-01 | NA | NA | (0.0184,Inf] | (0.192,21] |
| **1988** | NA | NA | NA | 2.64E+00 | NA | NA | NA | NA | NA |
| **1989** | NA | NA | NA | 0.00E+00 | NA | NA | NA | NA | NA |
| **1990** | NA | NA | Inf | 1.64E+00 | NA | NA | NA | (0.0184,Inf] | NA |
| **1991** | NA | NA | NA | NA | 3.98E-01 | NA | NA | NA | NA |
| **1992** | NA | NA | NA | 0.00E+00 | NA | NA | NA | NA | NA |
| **1993** | 1.99E-01 | 1.26E-01 | Inf | 1.78E+00 | 1.13E+00 | NA | NA | (0.0184,Inf] | (0.192,21] |
| **1994** | NA | NA | 7.73E-01 | NA | 3.34E+01 | NA | (14.9,Inf] | NA | (0.192,21] |
| **1995** | NA | NA | Inf | NA | NA | NA | NA | NA | (21,Inf] |
| **1996** | NA | NA | Inf | NA | NA | NA | NA | NA | (21,Inf] |
| **1997** | NA | NA | NA | 0.00E+00 | NA | NA | NA | NA | NA |
| **1998** | NA | NA | 0.00E+00 | NA | NA | NA | (14.9,Inf] | NA | NA |
| **1999** | 3.56E-02 | NA | NA | 0.00E+00 | 0.00E+00 | NA | NA | NA | NA |
| **2000** | NA | NA | NA | 4.49E-01 | NA | NA | NA | NA | NA |
| **2001** | NA | NA | NA | Inf | NA | NA | NA | NA | NA |
| **2002** | NA | NA | 0.00E+00 | NA | NA | NA | NA | NA | NA |
| **2003** | NA | NA | Inf | NA | NA | NA | NA | NA | (21,Inf] |
| **2004** | 1.35E-02 | 2.33E-02 | NA | 3.92E-01 | 6.76E-01 | NA | NA | NA | NA |
| **2005** | NA | NA | Inf | NA | NA | NA | NA | NA | NA |
| **2006** | NA | NA | NA | 0.00E+00 | NA | NA | NA | NA | NA |
| **2007** | 3.00E-02 | NA | NA | 0.00E+00 | 0.00E+00 | NA | NA | NA | NA |
| **2008** | 0.00E+00 | NA | NA | 0.00E+00 | NA | NA | NA | NA | NA |
| **2009** | 8.38E-02 | 0.00E+00 | Inf | 9.87E-01 | 0.00E+00 | NA | NA | (0.0184,Inf] | (21,Inf] |
| **2010** | NA | NA | NA | 4.25E-01 | NA | NA | NA | NA | NA |

| | | | | | | | | | |
|---|---|---|---|---|---|---|---|---|---|
| **2011** | NA | NA | NA | 0.00E+00 | NA | NA | NA | NA | NA |
| **2012** | NA | 1.33E-02 | NA | Inf | Inf | NA | NA | NA | NA |
| **2013** | NA | NA | Inf | NA | NA | NA | NA | NA | (21,Inf] |
| **2014** | NA | NA | NA | NA | 3.49E-01 | NA | NA | NA | NA |
| **2015** | NA | NA | NA | NA | 1.77E+00 | NA | NA | NA | NA |
| **2016** | NA | NA | Inf | NA | NA | NA | NA | NA | (21,Inf] |
| **2017** | Inf | 5.28E-02 | Inf | Inf | 9.59E-01 | NA | NA | (0.0184,Inf] | (0.192,21] |
| **2018** | 0.00E+00 | 0.00E+00 | Inf | 1.18E+00 | NA | NA | NA | (0.0184,Inf] | (21,Inf] |
| **2019** | NA | NA | Inf | NA | NA | NA | NA | NA | (21,Inf] |
| **2020** | NA | NA | Inf | NA | NA | NA | NA | NA | (21,Inf] |
| **2021** | NA | NA | Inf | NA | NA | NA | NA | NA | (21,Inf] |
| **2022** | NA | NA | 0.00E+00 | NA | NA | NA | NA | NA | NA |
| **2023** | NA | NA | Inf | 3.90E-01 | NA | NA | NA | (0.0184,Inf] | NA |
| **2024** | NA | NA | NA | NA | 8.24E-01 | NA | NA | NA | NA |
| **2025** | NA | NA | Inf | NA | NA | NA | NA | NA | NA |
| **2026** | NA | NA | NA | 0.00E+00 | NA | NA | NA | NA | NA |
| **2027** | Inf | 2.04E-02 | NA | Inf | 8.92E-01 | NA | NA | NA | NA |
| **2028** | NA | NA | 1.05E+00 | NA | 9.08E-01 | NA | (0.101,1.34] | NA | (0.192,21] |
| **2029** | NA | NA | NA | 0.00E+00 | NA | NA | NA | NA | NA |
| **2030** | NA | NA | Inf | NA | NA | NA | NA | NA | (21,Inf] |
| **2031** | NA | NA | 0.00E+00 | NA | 5.75E-01 | NA | (0,0.101] | NA | NA |
| **2032** | 0.00E+00 | NA | NA | 0.00E+00 | NA | NA | NA | NA | NA |
| **2033** | NA | NA | NA | NA | 5.37E+00 | NA | NA | NA | NA |
| **2034** | NA | NA | NA | NA | 4.32E-01 | NA | NA | NA | NA |
| **2035** | NA | NA | Inf | NA | NA | NA | NA | NA | NA |
| **2036** | NA | NA | Inf | NA | NA | NA | NA | NA | NA |
| **2037** | NA | NA | Inf | NA | NA | NA | NA | NA | NA |
| **2038** | NA | NA | NA | NA | 8.24E-02 | NA | NA | NA | NA |
| **2039** | NA | NA | 0.00E+00 | NA | 1.73E-01 | NA | (1.34,14.9] | NA | NA |
| **2040** | NA | NA | 0.00E+00 | NA | 3.47E-01 | NA | (0.101,1.34] | NA | NA |
| **2041** | NA | NA | NA | Inf | NA | NA | NA | NA | NA |
| **2042** | NA | NA | NA | NA | 6.54E-01 | NA | NA | NA | NA |
| **2043** | NA | NA | Inf | NA | NA | NA | NA | NA | (21,Inf] |
| **2044** | NA | 0.00E+00 | NA | Inf | NA | NA | NA | NA | NA |
| **2045** | NA | NA | 0.00E+00 | NA | 1.47E+01 | NA | (0.101,1.34] | NA | NA |
| **2046** | NA | NA | NA | NA | Inf | NA | NA | NA | NA |
| **2047** | NA | NA | Inf | NA | NA | NA | NA | NA | (21,Inf] |
| **2048** | NA | NA | Inf | NA | NA | NA | NA | NA | (21,Inf] |

| | | | | | | | | | |
|---|---|---|---|---|---|---|---|---|---|
| **2049** | NA | NA | Inf | NA | NA | NA | NA | NA | (21,Inf] |
| **2050** | NA | NA | Inf | NA | NA | NA | NA | NA | (21,Inf] |
| **2051** | NA | NA | 0.00E+00 | NA | NA | NA | NA | NA | NA |
| **2052** | Inf | 8.92E-02 | 0.00E+00 | Inf | 3.25E-01 | (0.0348,Inf] | (0.101,1.34] | NA | NA |
| **2053** | NA | NA | Inf | NA | NA | NA | NA | NA | NA |
| **2054** | NA | NA | NA | Inf | NA | NA | NA | NA | NA |
| **2055** | NA | NA | Inf | NA | NA | NA | NA | NA | (21,Inf] |
| **2056** | NA | NA | Inf | NA | NA | NA | NA | NA | (21,Inf] |
| **2057** | NA | NA | NA | NA | 2.94E+00 | NA | NA | NA | NA |
| **2058** | NA | NA | Inf | NA | NA | NA | NA | NA | (21,Inf] |
| **2059** | NA | NA | Inf | NA | NA | NA | NA | NA | (21,Inf] |
| **2060** | NA | NA | Inf | NA | NA | NA | NA | NA | (21,Inf] |
| **2061** | NA | NA | Inf | NA | NA | NA | NA | NA | NA |
| **2062** | NA | NA | Inf | NA | NA | NA | NA | NA | (21,Inf] |
| **2063** | NA | NA | 0.00E+00 | NA | NA | NA | (14.9,Inf] | NA | NA |
| **2064** | NA | NA | NA | 0.00E+00 | NA | NA | NA | NA | NA |
| **2065** | NA | NA | NA | NA | 3.29E-01 | NA | NA | NA | NA |
| **2066** | NA | NA | 0.00E+00 | NA | NA | NA | NA | NA | NA |
| **2067** | NA | NA | Inf | NA | NA | NA | NA | NA | (21,Inf] |
| **2068** | NA | NA | Inf | NA | 6.06E-01 | NA | NA | NA | (0.192,21] |
| **2069** | NA | NA | NA | NA | 2.52E-02 | NA | NA | NA | NA |
| **2070** | NA | NA | 0.00E+00 | NA | NA | NA | NA | NA | NA |
| **2071** | NA | NA | 0.00E+00 | NA | NA | NA | NA | NA | NA |
| **2072** | NA | NA | NA | NA | 8.06E-01 | NA | NA | NA | NA |
| **2073** | NA | NA | NA | Inf | NA | NA | NA | NA | NA |
| **2074** | NA | NA | Inf | NA | NA | NA | NA | NA | NA |
| **2075** | NA | NA | Inf | NA | NA | NA | NA | NA | NA |
| **2076** | NA | NA | 0.00E+00 | NA | 8.38E+00 | NA | (14.9,Inf] | NA | NA |
| **2077** | NA | NA | Inf | NA | NA | NA | NA | NA | NA |
| **2078** | NA | NA | Inf | NA | NA | NA | NA | NA | NA |
| **2079** | NA | NA | Inf | NA | NA | NA | NA | NA | NA |
| **2080** | NA | NA | 0.00E+00 | NA | NA | NA | NA | NA | NA |
| **2081** | NA | NA | NA | NA | 1.42E+00 | NA | NA | NA | NA |
| **2082** | NA | NA | NA | NA | 1.30E+00 | NA | NA | NA | NA |
| **2083** | NA | NA | NA | NA | 3.11E-01 | NA | NA | NA | NA |
| **2084** | NA | NA | Inf | NA | NA | NA | NA | NA | (21,Inf] |
| **2085** | NA | NA | 2.77E-01 | NA | 1.54E+00 | NA | (14.9,Inf] | NA | (21,Inf] |
| **2086** | NA | NA | NA | NA | 1.43E+00 | NA | NA | NA | NA |

| | | | | | | | | | |
|---|---|---|---|---|---|---|---|---|---|
| **2087** | NA | NA | 0.00E+00 | NA | NA | NA | NA | NA | NA |
| **2088** | NA | NA | NA | NA | 9.19E-01 | NA | NA | NA | NA |
| **2089** | NA | NA | 0.00E+00 | NA | NA | NA | NA | NA | NA |
| **2090** | NA | NA | NA | NA | 9.84E-01 | NA | NA | NA | NA |
| **2091** | NA | NA | NA | NA | 2.56E+00 | NA | NA | NA | NA |
| **2092** | NA | NA | 5.34E-02 | NA | 4.77E+00 | NA | (14.9,Inf] | NA | (0.192,21] |
| **2093** | NA | NA | NA | NA | 1.96E-01 | NA | NA | NA | NA |
| **2094** | NA | NA | 0.00E+00 | NA | 1.03E+00 | NA | (1.34,14.9] | NA | NA |
| **2095** | NA | NA | 0.00E+00 | NA | 4.37E+00 | NA | (14.9,Inf] | NA | NA |
| **2096** | NA | NA | Inf | NA | NA | NA | NA | NA | NA |
| **2097** | NA | NA | NA | NA | 3.08E-01 | NA | NA | NA | NA |
| **2098** | NA | NA | Inf | NA | NA | NA | NA | NA | NA |
| **2099** | NA | NA | NA | 0.00E+00 | NA | NA | NA | NA | NA |
| **2100** | NA | NA | 1.01E+00 | NA | 3.95E-01 | NA | (1.34,14.9] | NA | (0.192,21] |
| **2101** | NA | NA | 1.01E+00 | NA | 3.95E-01 | NA | (1.34,14.9] | NA | (0.192,21] |
| **2102** | NA | NA | 0.00E+00 | NA | 1.35E+01 | NA | (14.9,Inf] | NA | NA |
| **2103** | NA | NA | Inf | NA | NA | NA | NA | NA | (21,Inf] |
| **2104** | NA | NA | 0.00E+00 | NA | NA | NA | NA | NA | NA |
| **2105** | NA | NA | 0.00E+00 | NA | NA | NA | NA | NA | NA |
| **2106** | NA | NA | Inf | NA | NA | NA | NA | NA | NA |
| **2107** | NA | NA | NA | NA | 1.69E-01 | NA | NA | NA | NA |
| **2108** | NA | NA | 0.00E+00 | NA | NA | NA | NA | NA | NA |
| **2109** | NA | NA | Inf | NA | NA | NA | NA | NA | NA |
| **2110** | NA | NA | Inf | NA | NA | NA | NA | NA | NA |
| **2111** | NA | NA | Inf | NA | NA | NA | NA | NA | NA |
| **2112** | NA | NA | Inf | NA | NA | NA | NA | NA | (21,Inf] |
| **2113** | NA | NA | 0.00E+00 | NA | NA | NA | NA | NA | NA |
| **2114** | NA | NA | 1.29E+01 | NA | Inf | NA | (14.9,Inf] | NA | (21,Inf] |
| **2115** | NA | NA | 0.00E+00 | NA | 3.94E+00 | NA | (14.9,Inf] | NA | NA |
| **2116** | NA | NA | NA | Inf | NA | NA | NA | NA | NA |
| **2117** | NA | NA | NA | NA | 5.61E-01 | NA | NA | NA | NA |
| **2118** | NA | NA | Inf | NA | NA | NA | NA | NA | (21,Inf] |
| **2119** | NA | NA | NA | NA | 6.54E-02 | NA | NA | NA | NA |
| **2120** | NA | NA | Inf | NA | NA | NA | NA | NA | (21,Inf] |
| **2121** | NA | NA | 1.04E+00 | NA | 1.32E+01 | NA | (14.9,Inf] | NA | (0.192,21] |
| **2122** | NA | NA | NA | NA | 1.12E+00 | NA | NA | NA | NA |
| **2123** | NA | NA | Inf | NA | NA | NA | NA | NA | NA |
| **2124** | NA | NA | Inf | NA | NA | NA | NA | NA | NA |

| | | | | | | | | | |
|---|---|---|---|---|---|---|---|---|---|
| **2125** | NA | NA | Inf | NA | NA | NA | NA | NA | (21,Inf] |
| **2126** | NA | NA | NA | NA | 0.00E+00 | NA | NA | NA | NA |
| **2127** | NA | NA | Inf | NA | NA | NA | NA | NA | NA |
| **2128** | NA | NA | 0.00E+00 | NA | NA | NA | NA | NA | NA |
| **2129** | NA | NA | NA | NA | 2.61E-01 | NA | NA | NA | NA |
| **2130** | NA | NA | NA | NA | 1.30E-01 | NA | NA | NA | NA |
| **2131** | NA | NA | 0.00E+00 | NA | NA | NA | NA | NA | NA |
| **2132** | NA | NA | Inf | NA | NA | NA | NA | NA | (21,Inf] |
| **2133** | NA | NA | 0.00E+00 | NA | NA | NA | NA | NA | NA |
| **2134** | NA | NA | 0.00E+00 | NA | NA | NA | NA | NA | NA |
| **2135** | NA | NA | Inf | NA | NA | NA | NA | NA | NA |
| **2136** | NA | NA | 0.00E+00 | NA | NA | NA | NA | NA | NA |
| **2137** | NA | NA | Inf | NA | NA | NA | NA | NA | NA |
| **2138** | NA | NA | NA | NA | 5.64E+00 | NA | NA | NA | NA |
| **2139** | NA | NA | NA | NA | 3.57E-01 | NA | NA | NA | NA |
| **2140** | NA | NA | 0.00E+00 | NA | NA | NA | NA | NA | NA |
| **2141** | NA | NA | NA | NA | 7.95E-02 | NA | NA | NA | NA |
| **2142** | NA | NA | Inf | NA | NA | NA | NA | NA | NA |
| **2143** | NA | NA | Inf | NA | NA | NA | NA | NA | NA |
| **2144** | NA | NA | 0.00E+00 | NA | NA | NA | NA | NA | NA |
| **2145** | NA | NA | 0.00E+00 | NA | 1.03E+00 | NA | (1.34,14.9] | NA | NA |
| **2146** | NA | NA | Inf | NA | NA | NA | NA | NA | (21,Inf] |
| **2147** | NA | NA | 0.00E+00 | NA | Inf | NA | (14.9,Inf] | NA | NA |
| **2148** | NA | NA | NA | NA | 1.19E-01 | NA | NA | NA | NA |
| **2149** | NA | NA | NA | NA | 0.00E+00 | NA | NA | NA | NA |
| **2150** | NA | NA | Inf | NA | NA | NA | NA | NA | (21,Inf] |
| **2151** | NA | NA | 0.00E+00 | NA | NA | NA | NA | NA | NA |
| **2152** | NA | NA | NA | NA | 1.87E+00 | NA | NA | NA | NA |
| **2153** | NA | NA | NA | NA | 0.00E+00 | NA | NA | NA | NA |
| **2154** | NA | NA | 0.00E+00 | NA | NA | NA | NA | NA | NA |
| **2155** | NA | NA | 0.00E+00 | NA | NA | NA | NA | NA | NA |
| **2156** | NA | NA | NA | NA | 9.57E-01 | NA | NA | NA | NA |
| **2157** | NA | NA | Inf | NA | NA | NA | NA | NA | NA |
| **2158** | NA | NA | NA | NA | 3.09E-01 | NA | NA | NA | NA |
| **2159** | NA | NA | Inf | NA | NA | NA | NA | NA | NA |
| **2160** | NA | NA | NA | NA | 1.59E+00 | NA | NA | NA | NA |
| **2161** | NA | NA | Inf | NA | NA | NA | NA | NA | NA |
| **2162** | NA | NA | NA | NA | 1.63E+00 | NA | NA | NA | NA |

| | | | | | | | | | |
|---|---|---|---|---|---|---|---|---|---|
| **2163** | NA | NA | 3.30E-01 | NA | 6.64E+00 | NA | (14.9,Inf] | NA | (0.192,21] |
| **2164** | NA | NA | NA | NA | 0.00E+00 | NA | NA | NA | NA |
| **2165** | NA | NA | 0.00E+00 | NA | NA | NA | (14.9,Inf] | NA | NA |
| **2166** | NA | NA | NA | NA | 7.75E-02 | NA | NA | NA | NA |
| **2167** | NA | NA | NA | NA | 2.55E-01 | NA | NA | NA | NA |
| **2168** | NA | NA | NA | NA | 4.75E-01 | NA | NA | NA | NA |
| **2169** | NA | NA | 0.00E+00 | NA | Inf | NA | (14.9,Inf] | NA | NA |
| **2170** | NA | NA | NA | NA | 4.82E-01 | NA | NA | NA | NA |
| **2171** | NA | NA | 0.00E+00 | NA | NA | NA | NA | NA | NA |
| **2172** | NA | NA | Inf | NA | NA | NA | NA | NA | NA |
| **2173** | NA | NA | Inf | NA | NA | NA | NA | NA | (21,Inf] |
| **2174** | NA | NA | NA | NA | 6.32E-02 | NA | NA | NA | NA |
| **2175** | NA | NA | 0.00E+00 | NA | 8.81E-01 | NA | (14.9,Inf] | NA | NA |
| **2176** | NA | NA | NA | NA | 0.00E+00 | NA | NA | NA | NA |
| **2177** | NA | NA | Inf | NA | NA | NA | NA | NA | (21,Inf] |
| **2178** | NA | NA | NA | NA | 1.93E-01 | NA | NA | NA | NA |
| **2179** | NA | NA | 0.00E+00 | NA | 5.15E+00 | NA | (1.34,14.9] | NA | NA |
| **2180** | NA | NA | 0.00E+00 | NA | NA | NA | NA | NA | NA |
| **2181** | NA | NA | Inf | NA | NA | NA | NA | NA | (21,Inf] |
| **2182** | NA | NA | NA | NA | 1.25E-01 | NA | NA | NA | NA |
| **2183** | NA | NA | Inf | NA | NA | NA | NA | NA | NA |
| **2184** | NA | NA | Inf | NA | NA | NA | NA | NA | NA |
| **2185** | NA | NA | 0.00E+00 | NA | 8.86E-01 | NA | (1.34,14.9] | NA | NA |
| **2186** | NA | NA | 0.00E+00 | NA | 0.00E+00 | NA | (0.101,1.34] | NA | NA |
| **2187** | NA | NA | 0.00E+00 | NA | Inf | NA | (14.9,Inf] | NA | NA |
| **2188** | NA | NA | NA | NA | 4.27E-01 | NA | NA | NA | NA |
| **2189** | NA | NA | 0.00E+00 | NA | NA | NA | NA | NA | NA |
| **2190** | NA | NA | Inf | NA | NA | NA | NA | NA | NA |
| **2191** | NA | NA | 0.00E+00 | NA | Inf | NA | (14.9,Inf] | NA | NA |
| **2192** | NA | NA | 0.00E+00 | NA | Inf | NA | (14.9,Inf] | NA | NA |
| **2193** | NA | NA | NA | NA | 4.88E-01 | NA | NA | NA | NA |
| **2194** | NA | NA | NA | NA | 0.00E+00 | NA | NA | NA | NA |
| **2195** | NA | NA | NA | NA | 3.45E-01 | NA | NA | NA | NA |
| **2196** | NA | NA | NA | NA | 2.20E+00 | NA | NA | NA | NA |
| **2197** | NA | NA | 0.00E+00 | NA | NA | NA | NA | NA | NA |
| **2198** | NA | NA | 0.00E+00 | NA | NA | NA | NA | NA | NA |
| **2199** | NA | NA | NA | NA | 1.57E+00 | NA | NA | NA | NA |
| **2200** | NA | NA | NA | NA | 5.96E-01 | NA | NA | NA | NA |

| | | | | | | | | | | |
|---|---|---|---|---|---|---|---|---|---|---|
| **2201** | NA | NA | 0.00E+00 | NA | 6.42E+00 | | NA | (1.34,14.9] | NA | NA |
| **2202** | NA | NA | NA | NA | 0.00E+00 | | NA | NA | NA | NA |
| **2203** | NA | NA | 0.00E+00 | NA | NA | | NA | NA | NA | NA |
| **2204** | NA | NA | Inf | NA | NA | | NA | NA | NA | (21,Inf] |
| **2205** | NA | NA | 4.16E-02 | NA | 1.59E+00 | | NA | (14.9,Inf] | NA | (0.192,21] |
| **2206** | NA | NA | 0.00E+00 | NA | NA | | NA | NA | NA | NA |
| **2207** | NA | NA | NA | NA | 4.46E-01 | | NA | NA | NA | NA |
| **2208** | NA | NA | Inf | NA | NA | | NA | NA | NA | NA |
| **2209** | NA | NA | Inf | NA | NA | | NA | NA | NA | NA |
| **2210** | NA | NA | NA | NA | 2.26E+00 | | NA | NA | NA | NA |
| **2211** | NA | NA | NA | NA | 9.97E-01 | | NA | NA | NA | NA |
| **2212** | NA | NA | 0.00E+00 | NA | 1.95E+00 | | NA | (1.34,14.9] | NA | NA |
| **2213** | NA | NA | 0.00E+00 | NA | NA | | NA | NA | NA | NA |
| **2214** | NA | NA | 0.00E+00 | NA | NA | | NA | NA | NA | NA |
| **2215** | NA | NA | Inf | NA | NA | | NA | NA | NA | NA |
| **2216** | NA | NA | Inf | NA | NA | | NA | NA | NA | NA |
| **2217** | NA | NA | NA | NA | 1.27E+00 | | NA | NA | NA | NA |
| **2218** | NA | NA | Inf | NA | 6.73E-01 | | NA | NA | NA | (0.192,21] |
| **2219** | NA | NA | Inf | NA | NA | | NA | NA | NA | NA |
| **2220** | NA | NA | 0.00E+00 | NA | NA | | NA | (14.9,Inf] | NA | NA |
| **2221** | NA | NA | NA | NA | 3.77E-01 | | NA | NA | NA | NA |
| **2222** | NA | NA | Inf | NA | NA | | NA | NA | NA | (21,Inf] |
| **2223** | NA | NA | 0.00E+00 | NA | NA | | NA | NA | NA | NA |
| **2224** | NA | NA | Inf | NA | NA | | NA | NA | NA | NA |
| **2225** | NA | NA | NA | NA | 0.00E+00 | | NA | NA | NA | NA |
| **2226** | NA | NA | 0.00E+00 | NA | NA | | NA | NA | NA | NA |
| **2227** | NA | NA | NA | NA | 1.26E+00 | | NA | NA | NA | NA |
| **2228** | NA | NA | NA | NA | 6.92E-02 | | NA | NA | NA | NA |
| **2229** | NA | NA | 0.00E+00 | NA | 5.05E-02 | | NA | (0.101,1.34] | NA | NA |
| **2230** | NA | NA | 0.00E+00 | NA | NA | | NA | NA | NA | NA |
| **2231** | NA | NA | Inf | NA | 9.79E-02 | | NA | NA | NA | (21,Inf] |
| **2232** | NA | NA | 0.00E+00 | NA | NA | | NA | NA | NA | NA |
| **2233** | NA | NA | 0.00E+00 | NA | 6.95E-01 | | NA | (1.34,14.9] | NA | NA |
| **2234** | NA | NA | NA | NA | 5.96E-01 | | NA | NA | NA | NA |
| **2235** | NA | NA | 4.03E-01 | NA | Inf | | NA | (14.9,Inf] | NA | (0.192,21] |
| **2236** | NA | NA | NA | NA | 1.61E+00 | | NA | NA | NA | NA |
| **2237** | NA | NA | 0.00E+00 | NA | 3.31E+00 | | NA | (14.9,Inf] | NA | NA |
| **2238** | NA | NA | NA | NA | 5.85E-01 | | NA | NA | NA | NA |

| | | | | | | | | | | |
|---|---|---|---|---|---|---|---|---|---|---|
| **2239** | NA | NA | NA | NA | 0.00E+00 | | NA | NA | NA | NA |
| **2240** | NA | NA | 0.00E+00 | NA | NA | | NA | NA | NA | NA |
| **2241** | NA | NA | 0.00E+00 | NA | 9.32E+00 | | NA | (14.9,Inf] | NA | NA |
| **2242** | NA | NA | 0.00E+00 | NA | Inf | | NA | (14.9,Inf] | NA | NA |
| **2243** | NA | NA | 0.00E+00 | NA | 1.57E+01 | | NA | (14.9,Inf] | NA | NA |
| **2244** | NA | NA | 0.00E+00 | NA | Inf | | NA | (14.9,Inf] | NA | NA |
| **2245** | NA | NA | NA | NA | 9.01E-02 | | NA | NA | NA | NA |
| **2246** | NA | NA | 0.00E+00 | NA | 6.89E-01 | | NA | (1.34,14.9] | NA | NA |
| **2247** | NA | NA | Inf | NA | 3.02E-01 | | NA | NA | NA | (21,Inf] |
| **2248** | NA | NA | NA | NA | 2.74E-01 | | NA | NA | NA | NA |
| **2249** | NA | NA | NA | NA | 0.00E+00 | | NA | NA | NA | NA |
| **2250** | NA | NA | NA | NA | 4.83E-02 | | NA | NA | NA | NA |
| **2251** | NA | NA | 0.00E+00 | NA | NA | | NA | NA | NA | NA |
| **2252** | NA | NA | NA | NA | 1.07E+00 | | NA | NA | NA | NA |
| **2253** | NA | NA | NA | NA | Inf | | NA | NA | NA | NA |
| **2254** | NA | NA | 0.00E+00 | NA | 8.33E+00 | | NA | (14.9,Inf] | NA | NA |
| **2255** | NA | NA | NA | NA | 0.00E+00 | | NA | NA | NA | NA |
| **2256** | NA | NA | Inf | NA | 3.24E-01 | | NA | NA | NA | (0.192,21] |
| **2257** | NA | NA | NA | NA | 7.46E-02 | | NA | NA | NA | NA |
| **2258** | NA | NA | NA | NA | 0.00E+00 | | NA | NA | NA | NA |
| **2259** | NA | NA | NA | NA | 0.00E+00 | | NA | NA | NA | NA |
| **2260** | NA | NA | NA | NA | 0.00E+00 | | NA | NA | NA | NA |
| **2261** | NA | NA | NA | NA | Inf | | NA | NA | NA | NA |
| **2262** | NA | NA | Inf | NA | NA | | NA | NA | NA | NA |
| **2263** | NA | NA | NA | NA | 2.20E-01 | | NA | NA | NA | NA |
| **2264** | NA | NA | NA | NA | 0.00E+00 | | NA | NA | NA | NA |
| **2265** | NA | NA | NA | NA | 2.98E-01 | | NA | NA | NA | NA |
| **2266** | NA | NA | Inf | NA | NA | | NA | NA | NA | (21,Inf] |
| **2267** | NA | NA | Inf | NA | NA | | NA | NA | NA | (21,Inf] |
| **2268** | NA | NA | NA | NA | 0.00E+00 | | NA | NA | NA | NA |
| **2269** | NA | NA | 0.00E+00 | NA | 0.00E+00 | | NA | (0.101,1.34] | NA | NA |
| **2270** | NA | NA | 0.00E+00 | NA | NA | | NA | NA | NA | NA |
| **2271** | NA | NA | Inf | NA | NA | | NA | NA | NA | NA |
| **2272** | NA | NA | Inf | NA | NA | | NA | NA | NA | (21,Inf] |
| **2273** | NA | NA | 0.00E+00 | NA | NA | | NA | NA | NA | NA |
| **2274** | NA | NA | NA | NA | 2.64E+00 | | NA | NA | NA | NA |
| **2275** | NA | NA | NA | NA | 2.59E-01 | | NA | NA | NA | NA |
| **2276** | NA | NA | Inf | NA | NA | | NA | NA | NA | NA |

| | | | | | | | | | |
|---|---|---|---|---|---|---|---|---|---|
| **2277** | NA | NA | NA | NA | 1.16E-01 | NA | NA | NA | NA |
| **2278** | NA | NA | NA | NA | 7.44E-01 | NA | NA | NA | NA |
| **2279** | NA | NA | NA | NA | 0.00E+00 | NA | NA | NA | NA |
| **2280** | NA | NA | 2.27E+00 | NA | Inf | NA | (14.9,Inf] | NA | (0.192,21] |
| **2281** | NA | NA | NA | NA | 4.06E+00 | NA | NA | NA | NA |
| **2282** | NA | NA | 0.00E+00 | NA | NA | NA | NA | NA | NA |
| **2283** | NA | NA | Inf | NA | NA | NA | NA | NA | (21,Inf] |
| **2284** | NA | NA | NA | NA | Inf | NA | NA | NA | NA |
| **2285** | NA | NA | 1.60E+00 | NA | 0.00E+00 | NA | (1.34,14.9] | NA | (21,Inf] |
| **2286** | NA | NA | 0.00E+00 | NA | 3.43E-01 | NA | (14.9,Inf] | NA | NA |
| **2287** | NA | NA | NA | NA | 0.00E+00 | NA | NA | NA | NA |
| **2288** | NA | NA | Inf | NA | NA | NA | NA | NA | NA |
| **2289** | NA | NA | NA | NA | 2.77E+00 | NA | NA | NA | NA |
| **2290** | NA | NA | NA | NA | Inf | NA | NA | NA | NA |
| **2291** | NA | NA | NA | NA | 7.77E-01 | NA | NA | NA | NA |
| **2292** | NA | NA | Inf | NA | NA | NA | NA | NA | NA |
| **2293** | NA | NA | NA | NA | 0.00E+00 | NA | NA | NA | NA |
| **2294** | NA | NA | 0.00E+00 | NA | NA | NA | NA | NA | NA |
| **2295** | NA | NA | 0.00E+00 | NA | 7.16E-01 | NA | (14.9,Inf] | NA | NA |
| **2296** | NA | NA | Inf | NA | NA | NA | NA | NA | (21,Inf] |
| **2297** | NA | NA | NA | NA | Inf | NA | NA | NA | NA |
| **2298** | NA | NA | NA | NA | 0.00E+00 | NA | NA | NA | NA |
| **2299** | NA | NA | NA | NA | Inf | NA | NA | NA | NA |
| **2300** | NA | NA | Inf | NA | NA | NA | NA | NA | (21,Inf] |
| **2301** | NA | NA | Inf | NA | 0.00E+00 | NA | NA | NA | (21,Inf] |
| **2302** | NA | NA | 0.00E+00 | NA | Inf | NA | (14.9,Inf] | NA | NA |
| **2303** | NA | NA | 2.78E-01 | NA | 0.00E+00 | NA | (14.9,Inf] | NA | (21,Inf] |
| **2304** | NA | NA | Inf | NA | NA | NA | NA | NA | NA |
| **2305** | NA | NA | 9.11E-01 | NA | 1.67E+00 | NA | (14.9,Inf] | NA | (0.192,21] |
| **2306** | NA | NA | 0.00E+00 | NA | Inf | NA | (14.9,Inf] | NA | NA |
| **2307** | NA | NA | 0.00E+00 | NA | Inf | NA | (14.9,Inf] | NA | NA |
| **2308** | NA | NA | NA | NA | 0.00E+00 | NA | NA | NA | NA |
| **2309** | NA | NA | NA | NA | 0.00E+00 | NA | NA | NA | NA |
| **2310** | NA | NA | NA | NA | 0.00E+00 | NA | NA | NA | NA |
| **2311** | NA | NA | NA | NA | 6.44E-01 | NA | NA | NA | NA |
| **2312** | NA | NA | Inf | NA | NA | NA | NA | NA | NA |
| **2313** | NA | NA | NA | NA | 0.00E+00 | NA | NA | NA | NA |
| **2314** | NA | NA | NA | NA | 0.00E+00 | NA | NA | NA | NA |

| | | | | | | | | | |
|---|---|---|---|---|---|---|---|---|---|
| **2315** | NA | NA | NA | NA | 0.00E+00 | NA | NA | NA | NA |
| **2316** | NA | NA | NA | NA | 1.64E-01 | NA | NA | NA | NA |
| **2317** | NA | NA | Inf | NA | NA | NA | NA | NA | NA |
| **2318** | NA | NA | 0.00E+00 | NA | 1.02E+00 | NA | (14.9,Inf] | NA | NA |
| **2319** | NA | NA | 0.00E+00 | NA | 4.57E-01 | NA | (14.9,Inf] | NA | NA |
| **2320** | NA | NA | NA | NA | 2.11E-01 | NA | NA | NA | NA |
| **2321** | NA | NA | NA | NA | 0.00E+00 | NA | NA | NA | NA |
| **2322** | NA | NA | Inf | NA | NA | NA | NA | NA | NA |
| **2323** | NA | NA | NA | NA | 6.75E-01 | NA | NA | NA | NA |
| **2324** | NA | NA | Inf | NA | 0.00E+00 | NA | NA | NA | (21,Inf] |
| **2325** | NA | NA | 2.89E-02 | NA | 0.00E+00 | NA | (14.9,Inf] | NA | (21,Inf] |
| **2326** | NA | NA | NA | NA | 8.63E-01 | NA | NA | NA | NA |
| **2327** | NA | NA | NA | NA | 9.28E-01 | NA | NA | NA | NA |
| **2328** | NA | NA | NA | NA | 9.17E-01 | NA | NA | NA | NA |
| **2329** | NA | NA | 0.00E+00 | NA | NA | NA | NA | NA | NA |
| **2330** | NA | NA | 0.00E+00 | NA | Inf | NA | (14.9,Inf] | NA | NA |
| **2331** | NA | NA | NA | NA | 9.40E+00 | NA | NA | NA | NA |
| **2332** | NA | NA | 0.00E+00 | NA | Inf | NA | (14.9,Inf] | NA | NA |
| **2333** | NA | NA | NA | NA | 0.00E+00 | NA | NA | NA | NA |
| **2334** | NA | NA | NA | NA | 2.43E+00 | NA | NA | NA | NA |
| **2335** | NA | NA | 0.00E+00 | NA | NA | NA | NA | NA | NA |
| **2336** | NA | NA | 0.00E+00 | NA | Inf | NA | (14.9,Inf] | NA | NA |
| **2337** | NA | NA | NA | NA | 0.00E+00 | NA | NA | NA | NA |
| **2338** | NA | NA | NA | NA | 1.03E+01 | NA | NA | NA | NA |
| **2339** | NA | NA | Inf | NA | NA | NA | NA | NA | NA |
| **2340** | NA | NA | Inf | NA | 3.70E-01 | NA | NA | NA | (0.192,21] |
| **2341** | NA | NA | Inf | NA | 3.21E-01 | NA | NA | NA | (21,Inf] |
| **2342** | NA | NA | 0.00E+00 | NA | NA | NA | (14.9,Inf] | NA | NA |
| **2343** | NA | NA | NA | NA | 0.00E+00 | NA | NA | NA | NA |
| **2344** | NA | NA | NA | NA | 0.00E+00 | NA | NA | NA | NA |
| **2345** | NA | NA | Inf | NA | NA | NA | NA | NA | NA |
| **2346** | NA | NA | 0.00E+00 | NA | 8.16E-01 | NA | (1.34,14.9] | NA | NA |
| **2347** | NA | NA | NA | NA | 5.24E-01 | NA | NA | NA | NA |
| **2348** | NA | NA | NA | NA | 0.00E+00 | NA | NA | NA | NA |
| **2349** | NA | NA | Inf | NA | 1.55E-01 | NA | NA | NA | (21,Inf] |
| **2350** | NA | NA | NA | NA | 1.88E-01 | NA | NA | NA | NA |
| **2351** | NA | NA | NA | NA | 1.38E+00 | NA | NA | NA | NA |
| **2352** | NA | NA | Inf | NA | NA | NA | NA | NA | NA |

| | | | | | | | | | |
|---|---|---|---|---|---|---|---|---|---|
| **2353** | NA | NA | NA | NA | 0.00E+00 | NA | NA | NA | NA |
| **2354** | NA | NA | Inf | NA | NA | NA | NA | NA | NA |
| **2355** | NA | NA | NA | NA | 4.04E+00 | NA | NA | NA | NA |
| **2356** | NA | NA | NA | NA | 4.19E-01 | NA | NA | NA | NA |
| **2357** | NA | NA | NA | NA | 0.00E+00 | NA | NA | NA | NA |
| **2358** | NA | NA | NA | NA | 1.88E-01 | NA | NA | NA | NA |
| **2359** | NA | NA | 0.00E+00 | NA | 0.00E+00 | NA | (14.9,Inf] | NA | NA |
| **2360** | NA | NA | NA | NA | Inf | NA | NA | NA | NA |
| **2361** | NA | NA | NA | NA | 5.96E-01 | NA | NA | NA | NA |
| **2362** | NA | NA | Inf | NA | 4.97E-01 | NA | NA | NA | (0.192,21] |
| **2363** | NA | NA | NA | NA | 2.48E-01 | NA | NA | NA | NA |
| **2364** | NA | NA | 1.79E-01 | NA | 8.19E-01 | NA | (14.9,Inf] | NA | (0.192,21] |
| **2365** | NA | NA | NA | NA | 0.00E+00 | NA | NA | NA | NA |
| **2366** | NA | NA | NA | NA | 9.41E+00 | NA | NA | NA | NA |
| **2367** | NA | NA | NA | NA | 0.00E+00 | NA | NA | NA | NA |
| **2368** | NA | NA | 0.00E+00 | NA | 0.00E+00 | NA | (1.34,14.9] | NA | NA |
| **2369** | NA | NA | NA | NA | 3.16E-01 | NA | NA | NA | NA |
| **2370** | NA | NA | 0.00E+00 | NA | Inf | NA | (14.9,Inf] | NA | NA |
| **2371** | NA | NA | 0.00E+00 | NA | 1.69E-01 | NA | (0.101,1.34] | NA | NA |
| **2372** | NA | NA | NA | NA | 0.00E+00 | NA | NA | NA | NA |
| **2373** | NA | NA | Inf | NA | NA | NA | NA | NA | NA |
| **2374** | NA | NA | 0.00E+00 | NA | NA | NA | NA | NA | NA |
| **2375** | NA | NA | 0.00E+00 | NA | 6.26E-01 | NA | (1.34,14.9] | NA | NA |
| **2376** | NA | NA | Inf | NA | NA | NA | NA | NA | NA |
| **2377** | NA | NA | NA | NA | 0.00E+00 | NA | NA | NA | NA |
| **2378** | NA | NA | NA | NA | 4.08E-01 | NA | NA | NA | NA |
| **2379** | NA | NA | Inf | NA | NA | NA | NA | NA | NA |
| **2380** | NA | NA | NA | NA | 6.08E-01 | NA | NA | NA | NA |
| **2381** | NA | NA | NA | NA | 0.00E+00 | NA | NA | NA | NA |
| **2382** | NA | NA | 0.00E+00 | NA | 0.00E+00 | NA | (1.34,14.9] | NA | NA |
| **2383** | NA | NA | 4.35E-01 | NA | 1.87E+00 | NA | (14.9,Inf] | NA | (0.192,21] |
| **2384** | NA | NA | Inf | NA | 0.00E+00 | NA | NA | NA | (21,Inf] |
| **2385** | NA | NA | 0.00E+00 | NA | NA | NA | NA | NA | NA |
| **2386** | NA | NA | 0.00E+00 | NA | NA | NA | NA | NA | NA |
| **2387** | NA | NA | Inf | NA | NA | NA | NA | NA | (21,Inf] |
| **2388** | NA | NA | Inf | NA | NA | NA | NA | NA | (21,Inf] |
| **2389** | NA | NA | NA | NA | 0.00E+00 | NA | NA | NA | NA |
| **2390** | NA | NA | 0.00E+00 | NA | NA | NA | NA | NA | NA |

| | | | | | | | | | |
|---|---|---|---|---|---|---|---|---|---|
| **2391** | NA | NA | 0.00E+00 | NA | Inf | NA | (14.9,Inf] | NA | NA |
| **2392** | NA | NA | NA | NA | 4.45E-01 | NA | NA | NA | NA |
| **2393** | NA | NA | NA | NA | 0.00E+00 | NA | NA | NA | NA |
| **2394** | NA | NA | NA | NA | 0.00E+00 | NA | NA | NA | NA |
| **2395** | NA | NA | NA | NA | 1.04E-01 | NA | NA | NA | NA |
| **2396** | NA | NA | 0.00E+00 | NA | NA | NA | NA | NA | NA |
| **2397** | NA | NA | 0.00E+00 | NA | 0.00E+00 | NA | (1.34,14.9] | NA | NA |
| **2398** | NA | NA | NA | NA | Inf | NA | NA | NA | NA |
| **2399** | NA | NA | Inf | NA | NA | NA | NA | NA | NA |
| **2400** | NA | NA | NA | NA | 0.00E+00 | NA | NA | NA | NA |
| **2401** | NA | NA | NA | NA | 0.00E+00 | NA | NA | NA | NA |
| **2402** | NA | NA | NA | NA | 0.00E+00 | NA | NA | NA | NA |
| **2403** | NA | NA | NA | NA | 0.00E+00 | NA | NA | NA | NA |
| **2404** | NA | NA | NA | NA | 0.00E+00 | NA | NA | NA | NA |
| **2405** | NA | NA | 4.46E+01 | NA | Inf | NA | (14.9,Inf] | NA | (21,Inf] |
| **2406** | NA | NA | 2.29E-01 | NA | Inf | NA | (14.9,Inf] | NA | (0.192,21] |
| **2407** | NA | NA | NA | NA | Inf | NA | NA | NA | NA |
| **2408** | NA | NA | Inf | NA | NA | NA | NA | NA | NA |
| **2409** | NA | NA | 0.00E+00 | NA | 0.00E+00 | NA | (0.101,1.34] | NA | NA |
| **2410** | NA | NA | Inf | NA | NA | NA | NA | NA | NA |
| **2411** | NA | NA | 0.00E+00 | NA | 0.00E+00 | NA | (1.34,14.9] | NA | NA |
| **2412** | NA | NA | 0.00E+00 | NA | NA | NA | NA | NA | NA |
| **2413** | NA | NA | NA | NA | Inf | NA | NA | NA | NA |
| **2414** | NA | NA | 3.77E-01 | NA | 9.73E-02 | NA | (1.34,14.9] | NA | (21,Inf] |
| **2415** | NA | NA | Inf | NA | NA | NA | NA | NA | NA |
| **2416** | NA | NA | NA | NA | 0.00E+00 | NA | NA | NA | NA |
| **2417** | NA | NA | NA | NA | 8.66E+00 | NA | NA | NA | NA |
| **2418** | NA | NA | 1.11E+00 | NA | 1.08E+00 | NA | (1.34,14.9] | NA | (0.192,21] |
| **2419** | NA | NA | NA | NA | Inf | NA | NA | NA | NA |
| **2420** | NA | NA | NA | NA | 0.00E+00 | NA | NA | NA | NA |
| **2421** | NA | NA | Inf | NA | NA | NA | NA | NA | NA |
| **2422** | NA | NA | Inf | NA | 4.07E-01 | NA | NA | NA | (21,Inf] |
| **2423** | NA | NA | NA | NA | 0.00E+00 | NA | NA | NA | NA |
| **2424** | NA | NA | 0.00E+00 | NA | 4.87E+00 | NA | (14.9,Inf] | NA | NA |
| **2425** | NA | NA | 0.00E+00 | NA | Inf | NA | (14.9,Inf] | NA | NA |
| **2426** | NA | NA | NA | NA | 4.79E-01 | NA | NA | NA | NA |
| **2427** | NA | NA | NA | NA | 0.00E+00 | NA | NA | NA | NA |
| **2428** | NA | NA | NA | NA | 0.00E+00 | NA | NA | NA | NA |

| | | | | | | | | | |
|---|---|---|---|---|---|---|---|---|---|
| **2429** | NA | NA | NA | NA | 0.00E+00 | NA | NA | NA | NA |
| **2430** | NA | NA | NA | NA | 0.00E+00 | NA | NA | NA | NA |
| **2431** | NA | NA | NA | NA | 0.00E+00 | NA | NA | NA | NA |
| **2432** | NA | NA | Inf | NA | NA | NA | NA | NA | (21,Inf] |
| **2433** | NA | NA | NA | NA | 0.00E+00 | NA | NA | NA | NA |
| **2434** | NA | NA | 0.00E+00 | NA | NA | NA | NA | NA | NA |
| **2435** | NA | NA | NA | NA | Inf | NA | NA | NA | NA |
| **2436** | NA | NA | NA | NA | 0.00E+00 | NA | NA | NA | NA |
| **2437** | NA | NA | NA | NA | 0.00E+00 | NA | NA | NA | NA |
| **2438** | NA | NA | NA | NA | 0.00E+00 | NA | NA | NA | NA |
| **2439** | NA | NA | NA | NA | 0.00E+00 | NA | NA | NA | NA |
| **2440** | NA | NA | 2.83E+00 | NA | 0.00E+00 | NA | (1.34,14.9] | NA | (21,Inf] |
| **2441** | NA | NA | 0.00E+00 | NA | 1.53E-01 | NA | (1.34,14.9] | NA | NA |
| **2442** | NA | NA | NA | NA | 2.73E+00 | NA | NA | NA | NA |
| **2443** | NA | NA | NA | NA | 0.00E+00 | NA | NA | NA | NA |
| **2444** | NA | NA | 0.00E+00 | NA | 2.45E+00 | NA | (14.9,Inf] | NA | NA |
| **2445** | NA | NA | NA | NA | 1.46E+00 | NA | NA | NA | NA |
| **2446** | NA | NA | 0.00E+00 | NA | Inf | NA | (14.9,Inf] | NA | NA |
| **2447** | NA | NA | NA | NA | 0.00E+00 | NA | NA | NA | NA |
| **2448** | NA | NA | 1.25E-01 | NA | Inf | NA | (14.9,Inf] | NA | (0.192,21] |
| **2449** | NA | NA | NA | NA | 0.00E+00 | NA | NA | NA | NA |
| **2450** | NA | NA | NA | NA | Inf | NA | NA | NA | NA |
| **2451** | NA | NA | Inf | NA | NA | NA | NA | NA | NA |
| **2452** | NA | NA | 0.00E+00 | NA | Inf | NA | (14.9,Inf] | NA | NA |
| **2453** | NA | NA | 0.00E+00 | NA | Inf | NA | (14.9,Inf] | NA | NA |
| **2454** | NA | NA | NA | NA | 0.00E+00 | NA | NA | NA | NA |
| **2455** | NA | NA | NA | NA | 0.00E+00 | NA | NA | NA | NA |
| **2456** | NA | NA | NA | NA | Inf | NA | NA | NA | NA |
| **2457** | NA | NA | 0.00E+00 | NA | 3.40E-01 | NA | (1.34,14.9] | NA | NA |
| **2458** | NA | NA | NA | NA | 0.00E+00 | NA | NA | NA | NA |
| **2459** | NA | NA | NA | NA | 0.00E+00 | NA | NA | NA | NA |
| **2460** | NA | NA | NA | NA | 0.00E+00 | NA | NA | NA | NA |
| **2461** | NA | NA | NA | NA | 0.00E+00 | NA | NA | NA | NA |
| **2462** | NA | NA | 1.61E+01 | NA | 0.00E+00 | NA | (1.34,14.9] | NA | (21,Inf] |
| **2463** | NA | NA | 0.00E+00 | NA | 0.00E+00 | NA | (1.34,14.9] | NA | NA |
| **2464** | NA | NA | Inf | NA | NA | NA | NA | NA | NA |
| **2465** | NA | NA | Inf | NA | Inf | NA | NA | NA | (0.192,21] |
| **2466** | NA | NA | NA | NA | 0.00E+00 | NA | NA | NA | NA |

| | | | | | | | | | |
|---|---|---|---|---|---|---|---|---|---|
| **2467** | NA | NA | Inf | NA | NA | NA | NA | NA | NA |
| **2468** | NA | NA | Inf | NA | NA | NA | NA | NA | NA |
| **2469** | NA | NA | NA | NA | 0.00E+00 | NA | NA | NA | NA |
| **2470** | NA | NA | Inf | NA | 3.45E+00 | NA | NA | NA | (21,Inf] |
| **2471** | NA | NA | NA | NA | 0.00E+00 | NA | NA | NA | NA |
| **2472** | NA | NA | NA | NA | 0.00E+00 | NA | NA | NA | NA |
| **2473** | NA | NA | NA | NA | 4.70E+00 | NA | NA | NA | NA |
| **2474** | NA | NA | Inf | NA | NA | NA | NA | NA | NA |
| **2475** | NA | NA | 0.00E+00 | NA | NA | NA | NA | NA | NA |
| **2476** | NA | NA | NA | NA | 0.00E+00 | NA | NA | NA | NA |
| **2477** | NA | NA | NA | NA | 0.00E+00 | NA | NA | NA | NA |
| **2478** | NA | NA | NA | NA | 0.00E+00 | NA | NA | NA | NA |
| **2479** | NA | NA | 3.41E+00 | NA | 0.00E+00 | NA | (1.34,14.9] | NA | (21,Inf] |
| **2480** | NA | NA | NA | NA | Inf | NA | NA | NA | NA |
| **2481** | NA | NA | 0.00E+00 | NA | NA | NA | NA | NA | NA |
| **2482** | NA | NA | NA | NA | 5.01E-01 | NA | NA | NA | NA |
| **2483** | NA | NA | NA | NA | 0.00E+00 | NA | NA | NA | NA |
| **2484** | NA | NA | NA | NA | Inf | NA | NA | NA | NA |
| **2485** | NA | NA | 0.00E+00 | NA | Inf | NA | (14.9,Inf] | NA | NA |
| **2486** | NA | NA | NA | NA | 1.65E+00 | NA | NA | NA | NA |
| **2487** | NA | NA | NA | NA | Inf | NA | NA | NA | NA |
| **2488** | NA | NA | NA | NA | 0.00E+00 | NA | NA | NA | NA |
| **2489** | NA | NA | NA | NA | 0.00E+00 | NA | NA | NA | NA |
| **2490** | NA | NA | 0.00E+00 | NA | NA | NA | NA | NA | NA |
| **2491** | NA | NA | NA | NA | 3.62E-01 | NA | NA | NA | NA |
| **2492** | NA | NA | NA | NA | 0.00E+00 | NA | NA | NA | NA |
| **2493** | NA | NA | Inf | NA | NA | NA | NA | NA | NA |
| **2494** | NA | NA | NA | NA | Inf | NA | NA | NA | NA |
| **2495** | NA | NA | NA | NA | 0.00E+00 | NA | NA | NA | NA |
| **2496** | NA | NA | Inf | NA | NA | NA | NA | NA | NA |
| **2497** | NA | NA | NA | NA | Inf | NA | NA | NA | NA |
| **2498** | NA | NA | 2.37E+01 | NA | Inf | NA | (14.9,Inf] | NA | (21,Inf] |
| **2499** | NA | NA | NA | NA | 0.00E+00 | NA | NA | NA | NA |
| **2500** | NA | NA | Inf | NA | 0.00E+00 | NA | NA | NA | (21,Inf] |
| **2501** | NA | NA | 0.00E+00 | NA | NA | NA | NA | NA | NA |
| **2502** | NA | NA | 0.00E+00 | NA | Inf | NA | (14.9,Inf] | NA | NA |
| **2503** | NA | NA | 0.00E+00 | NA | 4.59E-01 | NA | (14.9,Inf] | NA | NA |
| **2504** | NA | NA | NA | NA | 0.00E+00 | NA | NA | NA | NA |

| | | | | | | | | | |
|---|---|---|---|---|---|---|---|---|---|
| **2505** | NA | NA | 0.00E+00 | NA | Inf | NA | (14.9,Inf] | NA | NA |
| **2506** | NA | NA | NA | NA | 2.21E-01 | NA | NA | NA | NA |
| **2507** | NA | NA | 2.05E+00 | NA | 2.50E+00 | NA | (1.34,14.9] | NA | (0.192,21] |
| **2508** | NA | NA | NA | NA | 2.59E-01 | NA | NA | NA | NA |
| **2509** | NA | NA | 0.00E+00 | NA | Inf | NA | (14.9,Inf] | NA | NA |
| **2510** | NA | NA | NA | NA | 0.00E+00 | NA | NA | NA | NA |
| **2511** | NA | NA | 0.00E+00 | NA | 0.00E+00 | NA | (1.34,14.9] | NA | NA |
| **2512** | NA | NA | NA | NA | 0.00E+00 | NA | NA | NA | NA |
| **2513** | NA | NA | NA | NA | 0.00E+00 | NA | NA | NA | NA |
| **2514** | NA | NA | 0.00E+00 | NA | NA | NA | NA | NA | NA |
| **2515** | NA | NA | NA | NA | 0.00E+00 | NA | NA | NA | NA |
| **2516** | NA | NA | 0.00E+00 | NA | 5.78E-01 | NA | (14.9,Inf] | NA | NA |
| **2517** | NA | NA | Inf | NA | NA | NA | NA | NA | NA |
| **2518** | NA | NA | Inf | NA | NA | NA | NA | NA | (21,Inf] |
| **2519** | NA | NA | NA | NA | 0.00E+00 | NA | NA | NA | NA |
| **2520** | NA | NA | Inf | NA | Inf | NA | NA | NA | (0.192,21] |
| **2521** | NA | NA | NA | NA | 0.00E+00 | NA | NA | NA | NA |
| **2522** | NA | NA | NA | NA | 0.00E+00 | NA | NA | NA | NA |
| **2523** | NA | NA | 0.00E+00 | NA | Inf | NA | (14.9,Inf] | NA | NA |
| **2524** | NA | NA | NA | NA | 0.00E+00 | NA | NA | NA | NA |
| **2525** | NA | NA | NA | NA | 0.00E+00 | NA | NA | NA | NA |
| **2526** | NA | NA | NA | NA | 3.38E-01 | NA | NA | NA | NA |
| **2527** | NA | NA | 0.00E+00 | NA | 2.62E-01 | NA | (1.34,14.9] | NA | NA |
| **2528** | NA | NA | NA | NA | 0.00E+00 | NA | NA | NA | NA |
| **2529** | NA | NA | NA | NA | 0.00E+00 | NA | NA | NA | NA |
| **2530** | NA | NA | NA | NA | Inf | NA | NA | NA | NA |
| **2531** | NA | NA | NA | NA | 0.00E+00 | NA | NA | NA | NA |
| **2532** | NA | NA | NA | NA | 0.00E+00 | NA | NA | NA | NA |
| **2533** | NA | NA | NA | NA | 0.00E+00 | NA | NA | NA | NA |
| **2534** | NA | NA | 0.00E+00 | NA | NA | NA | NA | NA | NA |
| **2535** | NA | NA | NA | NA | 0.00E+00 | NA | NA | NA | NA |
| **2536** | NA | NA | Inf | NA | 1.04E+00 | NA | NA | NA | (0.192,21] |
| **2537** | NA | NA | Inf | NA | 0.00E+00 | NA | NA | NA | (21,Inf] |
| **2538** | NA | NA | NA | NA | 0.00E+00 | NA | NA | NA | NA |
| **2539** | NA | NA | NA | NA | 0.00E+00 | NA | NA | NA | NA |
| **2540** | Inf | NA | NA | NA | 0.00E+00 | NA | NA | NA | NA |
| **2541** | NA | NA | NA | NA | 0.00E+00 | NA | NA | NA | NA |
| **2542** | NA | NA | NA | NA | 0.00E+00 | NA | NA | NA | NA |

| | | | | | | | | | | |
|---|---|---|---|---|---|---|---|---|---|---|
| **2543** | NA | NA | NA | NA | 0.00E+00 | | NA | NA | NA | NA |
| **2544** | NA | NA | 0.00E+00 | NA | Inf | | NA | (14.9,Inf] | NA | NA |
| **2545** | NA | NA | NA | NA | 0.00E+00 | | NA | NA | NA | NA |
| **2546** | NA | NA | NA | NA | 0.00E+00 | | NA | NA | NA | NA |
| **2547** | NA | NA | NA | NA | 3.20E+00 | | NA | NA | NA | NA |
| **2548** | NA | NA | NA | NA | 0.00E+00 | | NA | NA | NA | NA |
| **2549** | NA | NA | NA | NA | 0.00E+00 | | NA | NA | NA | NA |
| **2550** | NA | NA | NA | NA | Inf | | NA | NA | NA | NA |
| **2551** | NA | NA | NA | NA | 1.39E+00 | | NA | NA | NA | NA |
| **2552** | NA | NA | NA | NA | Inf | | NA | NA | NA | NA |
| **2553** | NA | NA | Inf | NA | 0.00E+00 | | NA | NA | NA | (21,Inf] |
| **2554** | NA | NA | NA | NA | 0.00E+00 | | NA | NA | NA | NA |
| **2555** | NA | NA | 0.00E+00 | NA | 0.00E+00 | | NA | (14.9,Inf] | NA | NA |
| **2556** | NA | NA | NA | NA | Inf | | NA | NA | NA | NA |
| **2557** | NA | NA | 0.00E+00 | NA | 0.00E+00 | | NA | (14.9,Inf] | NA | NA |
| **2558** | NA | NA | NA | NA | Inf | | NA | NA | NA | NA |
| **2559** | NA | NA | NA | NA | Inf | | NA | NA | NA | NA |
| **2560** | NA | NA | NA | NA | Inf | | NA | NA | NA | NA |
| **2561** | NA | NA | 0.00E+00 | NA | Inf | | NA | (14.9,Inf] | NA | NA |
| **2562** | NA | NA | 0.00E+00 | NA | 0.00E+00 | | NA | (0.101,1.34] | NA | NA |
| **2563** | NA | NA | NA | NA | 0.00E+00 | | NA | NA | NA | NA |
| **2564** | NA | NA | NA | NA | Inf | | NA | NA | NA | NA |
| **2565** | NA | NA | NA | NA | Inf | | NA | NA | NA | NA |
| **2566** | NA | NA | NA | NA | Inf | | NA | NA | NA | NA |
| **2567** | NA | NA | NA | NA | 0.00E+00 | | NA | NA | NA | NA |
| **2568** | NA | NA | NA | NA | 0.00E+00 | | NA | NA | NA | NA |
| **2569** | NA | NA | 0.00E+00 | NA | 0.00E+00 | | NA | (1.34,14.9] | NA | NA |
| **2570** | NA | NA | NA | NA | Inf | | NA | NA | NA | NA |
| **2571** | NA | NA | 6.99E+00 | NA | Inf | | NA | (14.9,Inf] | NA | (21,Inf] |
| **2572** | NA | NA | NA | NA | 5.34E-01 | | NA | NA | NA | NA |
| **2573** | NA | NA | 0.00E+00 | NA | Inf | | NA | (14.9,Inf] | NA | NA |
| **2574** | NA | NA | 0.00E+00 | NA | Inf | | NA | (14.9,Inf] | NA | NA |
| **2575** | NA | NA | NA | NA | 0.00E+00 | | NA | NA | NA | NA |
| **2576** | NA | NA | NA | NA | 0.00E+00 | | NA | NA | NA | NA |
| **2577** | NA | NA | NA | NA | Inf | | NA | NA | NA | NA |
| **2578** | NA | NA | 0.00E+00 | NA | Inf | | NA | (14.9,Inf] | NA | NA |
| **2579** | NA | NA | NA | NA | Inf | | NA | NA | NA | NA |
| **2580** | NA | NA | NA | NA | 0.00E+00 | | NA | NA | NA | NA |

| | | | | | | | | | |
|---|---|---|---|---|---|---|---|---|---|
| **2581** | NA | NA | NA | NA | 8.06E-01 | NA | NA | NA | NA |
| **2582** | NA | NA | NA | NA | 0.00E+00 | NA | NA | NA | NA |
| **2583** | NA | NA | NA | NA | Inf | NA | NA | NA | NA |
| **2584** | NA | NA | NA | NA | Inf | NA | NA | NA | NA |
| **2585** | NA | NA | NA | NA | 0.00E+00 | NA | NA | NA | NA |
| **2586** | NA | NA | NA | NA | Inf | NA | NA | NA | NA |
| **2587** | NA | NA | NA | NA | 0.00E+00 | NA | NA | NA | NA |
| **2588** | NA | NA | NA | NA | Inf | NA | NA | NA | NA |
| **2589** | NA | NA | 0.00E+00 | NA | 2.99E-01 | NA | (1.34,14.9] | NA | NA |
| **2590** | NA | NA | NA | NA | 1.79E+00 | NA | NA | NA | NA |
| **2591** | NA | NA | NA | NA | Inf | NA | NA | NA | NA |
| **2592** | NA | NA | NA | NA | 0.00E+00 | NA | NA | NA | NA |
| **2593** | NA | NA | Inf | NA | NA | NA | NA | NA | NA |
| **2594** | NA | NA | NA | NA | 0.00E+00 | NA | NA | NA | NA |
| **2595** | NA | NA | Inf | NA | NA | NA | NA | NA | NA |
| **2596** | NA | NA | NA | NA | 0.00E+00 | NA | NA | NA | NA |
| **2597** | NA | NA | NA | NA | 0.00E+00 | NA | NA | NA | NA |
| **2598** | NA | NA | NA | NA | Inf | NA | NA | NA | NA |
| **2599** | NA | NA | NA | NA | 0.00E+00 | NA | NA | NA | NA |
| **2600** | NA | NA | NA | NA | 0.00E+00 | NA | NA | NA | NA |
| **2601** | NA | NA | 0.00E+00 | NA | Inf | NA | (14.9,Inf] | NA | NA |
| **2602** | NA | NA | NA | NA | 9.02E-01 | NA | NA | NA | NA |
| **2603** | NA | NA | NA | NA | 0.00E+00 | NA | NA | NA | NA |
| **2604** | NA | NA | NA | NA | Inf | NA | NA | NA | NA |
| **2605** | NA | NA | NA | NA | 0.00E+00 | NA | NA | NA | NA |
| **2606** | NA | NA | 0.00E+00 | NA | Inf | NA | (14.9,Inf] | NA | NA |
| **2607** | NA | NA | NA | NA | 0.00E+00 | NA | NA | NA | NA |
| **2608** | NA | NA | NA | NA | 0.00E+00 | NA | NA | NA | NA |
| **2609** | NA | NA | NA | NA | Inf | NA | NA | NA | NA |
| **2610** | NA | NA | NA | NA | 0.00E+00 | NA | NA | NA | NA |
| **2611** | NA | NA | 0.00E+00 | NA | 0.00E+00 | NA | (1.34,14.9] | NA | NA |
| **2612** | NA | NA | NA | NA | Inf | NA | NA | NA | NA |
| **2613** | NA | NA | NA | NA | 1.36E+00 | NA | NA | NA | NA |
| **2614** | NA | NA | 0.00E+00 | NA | Inf | NA | (14.9,Inf] | NA | NA |
| **2615** | NA | NA | NA | NA | Inf | NA | NA | NA | NA |
| **2616** | NA | NA | NA | NA | Inf | NA | NA | NA | NA |
| **2617** | NA | NA | NA | NA | Inf | NA | NA | NA | NA |
| **2618** | NA | NA | Inf | NA | 0.00E+00 | NA | NA | NA | (21,Inf] |

| | | | | | | | | | | |
|---|---|---|---|---|---|---|---|---|---|---|
| **2619** | NA | NA | NA | NA | 0.00E+00 | | NA | NA | NA | NA |
| **2620** | NA | NA | NA | NA | Inf | | NA | NA | NA | NA |
| **2621** | NA | NA | NA | NA | Inf | | NA | NA | NA | NA |
| **2622** | NA | NA | 0.00E+00 | NA | Inf | | NA | (14.9,Inf] | NA | NA |
| **2623** | NA | NA | NA | NA | Inf | | NA | NA | NA | NA |
| **2624** | NA | NA | NA | NA | 0.00E+00 | | NA | NA | NA | NA |
| **2625** | NA | NA | NA | NA | 0.00E+00 | | NA | NA | NA | NA |
| **2626** | NA | NA | NA | NA | Inf | | NA | NA | NA | NA |
| **2627** | NA | NA | Inf | NA | Inf | | NA | NA | NA | (21,Inf] |
| **2628** | NA | NA | NA | NA | 0.00E+00 | | NA | NA | NA | NA |
| **2629** | NA | NA | NA | NA | 0.00E+00 | | NA | NA | NA | NA |
| **2630** | NA | NA | Inf | NA | NA | | NA | NA | NA | NA |
| **2631** | NA | NA | NA | NA | Inf | | NA | NA | NA | NA |
| **2632** | NA | NA | NA | NA | 0.00E+00 | | NA | NA | NA | NA |
| **2633** | NA | NA | NA | NA | Inf | | NA | NA | NA | NA |
| **2634** | NA | NA | NA | NA | Inf | | NA | NA | NA | NA |
| **2635** | NA | NA | NA | NA | 0.00E+00 | | NA | NA | NA | NA |
| **2636** | NA | NA | Inf | NA | 0.00E+00 | | NA | NA | NA | (21,Inf] |
| **2637** | NA | NA | NA | NA | Inf | | NA | NA | NA | NA |
| **2638** | NA | NA | NA | NA | Inf | | NA | NA | NA | NA |
| **2639** | NA | NA | NA | NA | 0.00E+00 | | NA | NA | NA | NA |
| **2640** | NA | NA | NA | NA | Inf | | NA | NA | NA | NA |
| **2641** | NA | NA | NA | NA | 0.00E+00 | | NA | NA | NA | NA |
| **2642** | NA | NA | NA | NA | 0.00E+00 | | NA | NA | NA | NA |
| **2643** | NA | NA | Inf | NA | 0.00E+00 | | NA | NA | NA | (21,Inf] |
| **2644** | NA | NA | 0.00E+00 | NA | Inf | | NA | (14.9,Inf] | NA | NA |
| **2645** | NA | NA | NA | NA | Inf | | NA | NA | NA | NA |
| **2646** | NA | NA | NA | NA | Inf | | NA | NA | NA | NA |
| **2647** | NA | NA | NA | NA | Inf | | NA | NA | NA | NA |
| **2648** | NA | NA | NA | NA | Inf | | NA | NA | NA | NA |
| **2649** | NA | NA | NA | NA | 0.00E+00 | | NA | NA | NA | NA |
| **2650** | NA | NA | NA | NA | Inf | | NA | NA | NA | NA |
| **2651** | NA | NA | NA | NA | Inf | | NA | NA | NA | NA |
| **2652** | NA | NA | NA | NA | Inf | | NA | NA | NA | NA |
| **2653** | NA | NA | NA | NA | Inf | | NA | NA | NA | NA |
| **2654** | NA | NA | NA | NA | Inf | | NA | NA | NA | NA |
| **2655** | NA | NA | NA | NA | 0.00E+00 | | NA | NA | NA | NA |
| **2656** | NA | NA | NA | NA | 0.00E+00 | | NA | NA | NA | NA |

| | | | | | | | | | |
|---|---|---|---|---|---|---|---|---|---|
| **2657** | NA | NA | NA | NA | Inf | NA | NA | NA | NA |
| **2658** | NA | NA | Inf | NA | 0.00E+00 | NA | NA | NA | (21,Inf] |
| **2659** | NA | NA | NA | NA | 0.00E+00 | NA | NA | NA | NA |
| **2660** | NA | NA | NA | NA | Inf | NA | NA | NA | NA |
| **2661** | NA | NA | 0.00E+00 | NA | Inf | NA | (14.9,Inf] | NA | NA |
| **2662** | NA | NA | 5.69E+00 | NA | Inf | NA | (14.9,Inf] | NA | (21,Inf] |
| **2663** | NA | NA | NA | NA | Inf | NA | NA | NA | NA |
| **2664** | NA | NA | NA | NA | 0.00E+00 | NA | NA | NA | NA |
| **2665** | NA | NA | NA | NA | Inf | NA | NA | NA | NA |
| **2666** | NA | NA | NA | NA | Inf | NA | NA | NA | NA |
| **2667** | NA | NA | NA | NA | Inf | NA | NA | NA | NA |
| **2668** | NA | NA | NA | NA | Inf | NA | NA | NA | NA |
| **2669** | NA | NA | NA | NA | Inf | NA | NA | NA | NA |
| **2670** | NA | NA | NA | NA | 0.00E+00 | NA | NA | NA | NA |
| **2671** | NA | NA | NA | NA | 0.00E+00 | NA | NA | NA | NA |
| **2672** | NA | NA | NA | NA | Inf | NA | NA | NA | NA |
| **2673** | NA | NA | NA | NA | Inf | NA | NA | NA | NA |
| **2674** | NA | NA | NA | NA | 0.00E+00 | NA | NA | NA | NA |
| **2675** | NA | NA | Inf | NA | 0.00E+00 | NA | NA | NA | (21,Inf] |
| **2676** | NA | NA | NA | NA | Inf | NA | NA | NA | NA |
| **2677** | NA | NA | NA | NA | Inf | NA | NA | NA | NA |
| **2678** | NA | NA | NA | NA | 0.00E+00 | NA | NA | NA | NA |
| **2679** | NA | NA | 7.23E+00 | NA | Inf | NA | (14.9,Inf] | NA | (21,Inf] |
| **2680** | NA | NA | NA | NA | Inf | NA | NA | NA | NA |
| **2681** | NA | NA | NA | NA | Inf | NA | NA | NA | NA |
| **2682** | NA | NA | Inf | NA | Inf | NA | NA | NA | (21,Inf] |
| **2683** | NA | NA | NA | NA | Inf | NA | NA | NA | NA |
| **2684** | NA | NA | 0.00E+00 | NA | Inf | NA | (14.9,Inf] | NA | NA |
| **2685** | NA | NA | 1.74E+02 | NA | 0.00E+00 | NA | (1.34,14.9] | NA | (21,Inf] |
| **2686** | NA | NA | 0.00E+00 | NA | Inf | NA | (14.9,Inf] | NA | NA |
| **2687** | NA | NA | NA | NA | Inf | NA | NA | NA | NA |
| **2688** | NA | NA | NA | NA | Inf | NA | NA | NA | NA |
| **2689** | NA | NA | NA | NA | 0.00E+00 | NA | NA | NA | NA |
| **2690** | NA | NA | NA | NA | Inf | NA | NA | NA | NA |
| **2691** | NA | NA | NA | NA | 0.00E+00 | NA | NA | NA | NA |
| **2692** | NA | NA | 0.00E+00 | NA | NA | NA | NA | NA | NA |
| **2693** | NA | NA | 0.00E+00 | NA | Inf | NA | (14.9,Inf] | NA | NA |
| **2694** | NA | NA | 0.00E+00 | NA | Inf | NA | (14.9,Inf] | NA | NA |

| | | | | | | | | | |
|---|---|---|---|---|---|---|---|---|---|
| **2695** | NA | NA | 2.63E+01 | NA | Inf | NA | (14.9,Inf] | NA | (21,Inf] |
| **2696** | NA | NA | NA | NA | 0.00E+00 | NA | NA | NA | NA |
| **2697** | NA | NA | NA | NA | Inf | NA | NA | NA | NA |
| **2698** | NA | NA | 0.00E+00 | NA | Inf | NA | (14.9,Inf] | NA | NA |
| **2699** | NA | NA | NA | NA | 0.00E+00 | NA | NA | NA | NA |
| **2700** | NA | NA | Inf | NA | Inf | NA | NA | NA | (0.192,21] |
| **2701** | NA | NA | NA | NA | 0.00E+00 | NA | NA | NA | NA |
| **2702** | NA | NA | 0.00E+00 | NA | Inf | NA | (14.9,Inf] | NA | NA |
| **2703** | NA | NA | NA | NA | Inf | NA | NA | NA | NA |
| **2704** | NA | NA | NA | NA | Inf | NA | NA | NA | NA |
| **2705** | NA | NA | NA | NA | Inf | NA | NA | NA | NA |
| **2706** | NA | NA | NA | NA | Inf | NA | NA | NA | NA |
| **2707** | NA | NA | NA | NA | Inf | NA | NA | NA | NA |
| **2708** | NA | NA | 0.00E+00 | NA | 0.00E+00 | NA | (14.9,Inf] | NA | NA |
| **2709** | NA | NA | 1.94E+02 | NA | NA | NA | (14.9,Inf] | NA | (21,Inf] |
| **2710** | NA | NA | 1.04E+02 | NA | NA | NA | NA | NA | NA |
| **2711** | NA | NA | 1.02E+02 | NA | NA | NA | (14.9,Inf] | NA | (21,Inf] |
| **2712** | NA | NA | 4.20E+01 | NA | NA | NA | (14.9,Inf] | NA | (21,Inf] |
| **2713** | NA | NA | 3.75E+00 | NA | NA | NA | (14.9,Inf] | NA | (21,Inf] |
| **2714** | NA | NA | 3.11E+00 | NA | NA | NA | NA | NA | NA |
| **2715** | NA | NA | 2.83E+00 | NA | NA | NA | (14.9,Inf] | NA | (21,Inf] |
| **2716** | NA | NA | 2.60E+00 | NA | NA | NA | (14.9,Inf] | NA | (21,Inf] |
| **2717** | NA | NA | 1.93E+00 | NA | NA | NA | NA | NA | NA |
| **2718** | NA | NA | 1.87E+00 | NA | NA | NA | NA | NA | NA |
| **2719** | NA | NA | 1.11E+00 | NA | NA | NA | NA | NA | NA |
| **2720** | NA | NA | 1.08E+00 | NA | NA | NA | (14.9,Inf] | NA | (21,Inf] |
| **2721** | NA | NA | 5.12E-01 | NA | NA | NA | (14.9,Inf] | NA | (21,Inf] |
| **2722** | NA | NA | 5.11E-01 | NA | NA | NA | NA | NA | NA |
| **2723** | NA | NA | 3.04E-02 | NA | NA | NA | NA | NA | NA |

| Row Number | Bins<br>Wash02GRvsWash01GRbins | Wash02PCFTvsWash01PCFTbins | ElutePCFTvsEluteGRbins | Wash01PCFTvsWash01GRbins | Wash02PCFTvsWash02GRbins |
|---|---|---|---|---|---|
| **1** | (0.00386,0.00875] | (0.00292,0.00665] | (0.46,Inf] | (0.936,1.11] | (0.798,1.72] |
| **2** | (0.00386,0.00875] | (0.00292,0.00665] | NA | (0.936,1.11] | (0.798,1.72] |
| **3** | (0.00875,Inf] | (0.00665,Inf] | (0.46,Inf] | (1.11,Inf] | (0.798,1.72] |
| **4** | (0.00179,0.00386] | (0.00125,0.00292] | (0,0.46] | (0.936,1.11] | (0.213,0.798] |
| **5** | (0.00179,0.00386] | (0.00292,0.00665] | (0,0.46] | (0.936,1.11] | (0.798,1.72] |
| **6** | (0,0.00179] | (0.00125,0.00292] | (0,0.46] | (1.11,Inf] | (0.798,1.72] |
| **7** | (0.00386,0.00875] | (0.00292,0.00665] | (0.46,Inf] | (0.936,1.11] | (0.213,0.798] |
| **8** | (0.00875,Inf] | (0.00665,Inf] | (0.46,Inf] | (1.11,Inf] | (0.798,1.72] |
| **9** | (0.00875,Inf] | (0.00665,Inf] | (0,0.46] | (1.11,Inf] | (1.72,Inf] |
| **10** | (0,0.00179] | (0.00125,0.00292] | (0.46,Inf] | (0.731,0.936] | (0.798,1.72] |
| **11** | (0.00179,0.00386] | (0.00125,0.00292] | (0.46,Inf] | (0.731,0.936] | (0.798,1.72] |
| **12** | (0.00386,0.00875] | (0.00665,Inf] | (0.46,Inf] | (0.936,1.11] | (0.798,1.72] |
| **13** | (0.00179,0.00386] | (0.00125,0.00292] | (0,0.46] | (0.936,1.11] | (0.798,1.72] |
| **14** | (0.00179,0.00386] | (0.00292,0.00665] | (0.46,Inf] | (0.936,1.11] | (0.798,1.72] |
| **15** | (0.00179,0.00386] | (0.00125,0.00292] | (0,0.46] | (0.936,1.11] | (0.213,0.798] |
| **16** | (0.00179,0.00386] | (0.00125,0.00292] | NA | (0.936,1.11] | (0.213,0.798] |
| **17** | (0.00179,0.00386] | (0.00292,0.00665] | (0.46,Inf] | (0.936,1.11] | (0.798,1.72] |
| **18** | (0.00386,0.00875] | (0.00292,0.00665] | (0,0.46] | (0.936,1.11] | (0.213,0.798] |
| **19** | (0.00179,0.00386] | (0.00125,0.00292] | (0,0.46] | (0.936,1.11] | (0.798,1.72] |
| **20** | (0,0.00179] | (0.00125,0.00292] | (0,0.46] | (0.936,1.11] | (0.798,1.72] |
| **21** | (0,0.00179] | (0,0.00125] | (0.46,Inf] | (1.11,Inf] | (0.798,1.72] |
| **22** | (0,0.00179] | (0.00125,0.00292] | (0,0.46] | (0.936,1.11] | (0.798,1.72] |
| **23** | (0,0.00179] | (0,0.00125] | NA | (0.936,1.11] | (1.72,Inf] |
| **24** | (0.00179,0.00386] | (0.00292,0.00665] | (0.46,Inf] | (1.11,Inf] | (0.798,1.72] |
| **25** | (0.00179,0.00386] | (0.00125,0.00292] | (0,0.46] | (1.11,Inf] | (0.798,1.72] |
| **26** | (0.00875,Inf] | (0.00665,Inf] | NA | (1.11,Inf] | (0.213,0.798] |
| **27** | (0,0.00179] | (0.00125,0.00292] | (0,0.46] | (0.936,1.11] | (0.798,1.72] |
| **28** | (0.00179,0.00386] | (0.00292,0.00665] | NA | (0.936,1.11] | (0.798,1.72] |
| **29** | (0.00386,0.00875] | (0.00292,0.00665] | NA | (0.936,1.11] | (0.798,1.72] |
| **30** | (0.00179,0.00386] | (0.00125,0.00292] | (0.46,Inf] | (0.936,1.11] | (0.798,1.72] |
| **31** | (0,0.00179] | (0.00125,0.00292] | (0.46,Inf] | (0.936,1.11] | (0.798,1.72] |
| **32** | (0.00386,0.00875] | (0.00125,0.00292] | (0,0.46] | (1.11,Inf] | (0.213,0.798] |
| **33** | (0.00386,0.00875] | (0.00125,0.00292] | NA | (0.936,1.11] | (0.213,0.798] |
| **34** | (0.00875,Inf] | (0.00665,Inf] | (0,0.46] | (1.11,Inf] | (0.798,1.72] |

| | | | | | |
|---|---|---|---|---|---|
| **35** | (0.00179,0.00386] | (0.00292,0.00665] | (0.46,Inf] | (0.936,1.11] | (0.798,1.72] |
| **36** | (0.00179,0.00386] | (0.00292,0.00665] | (0,0.46] | (0.936,1.11] | (0.798,1.72] |
| **37** | (0.00179,0.00386] | (0.00125,0.00292] | (0,0.46] | (0.936,1.11] | (0.213,0.798] |
| **38** | (0.00179,0.00386] | (0.00292,0.00665] | (0.46,Inf] | (0.936,1.11] | (0.798,1.72] |
| **39** | (0,0.00179] | (0.00125,0.00292] | NA | (1.11,Inf] | (0.798,1.72] |
| **40** | (0.00179,0.00386] | (0.00125,0.00292] | (0.46,Inf] | (0.936,1.11] | (0.213,0.798] |
| **41** | (0,0.00179] | (0.00125,0.00292] | NA | (0.936,1.11] | (0.798,1.72] |
| **42** | (0,0.00179] | (0.00125,0.00292] | NA | (0.936,1.11] | (0.798,1.72] |
| **43** | (0.00179,0.00386] | (0.00292,0.00665] | NA | (0.936,1.11] | (0.798,1.72] |
| **44** | (0.00179,0.00386] | (0.00125,0.00292] | NA | (0.936,1.11] | (0.798,1.72] |
| **45** | (0.00875,Inf] | (0.00665,Inf] | (0.46,Inf] | (1.11,Inf] | (0.798,1.72] |
| **46** | (0.00179,0.00386] | (0.00292,0.00665] | (0.46,Inf] | (0.936,1.11] | (0.798,1.72] |
| **47** | (0,0.00179] | (0,0.00125] | NA | (0.936,1.11] | (0.798,1.72] |
| **48** | (0,0.00179] | (0.00125,0.00292] | (0,0.46] | (0.936,1.11] | (0.798,1.72] |
| **49** | (0,0.00179] | (0.00125,0.00292] | (0,0.46] | (0.936,1.11] | (0.798,1.72] |
| **50** | (0.00875,Inf] | (0.00665,Inf] | (0.46,Inf] | (1.11,Inf] | (0.798,1.72] |
| **51** | (0.00386,0.00875] | (0.00292,0.00665] | NA | (0.936,1.11] | (0.798,1.72] |
| **52** | (0.00179,0.00386] | (0.00125,0.00292] | (0,0.46] | (1.11,Inf] | (0.213,0.798] |
| **53** | (0.00179,0.00386] | (0.00125,0.00292] | NA | (0.936,1.11] | (0.798,1.72] |
| **54** | (0,0.00179] | (0.00125,0.00292] | NA | (0.936,1.11] | (0.798,1.72] |
| **55** | (0,0.00179] | (0.00125,0.00292] | NA | (0.731,0.936] | (1.72,Inf] |
| **56** | (0.00386,0.00875] | (0.00292,0.00665] | NA | (1.11,Inf] | (0.213,0.798] |
| **57** | (0.00386,0.00875] | (0.00292,0.00665] | (0,0.46] | (1.11,Inf] | (0.798,1.72] |
| **58** | (0.00386,0.00875] | (0.00292,0.00665] | (0,0.46] | (1.11,Inf] | (0.798,1.72] |
| **59** | (0.00386,0.00875] | (0.00292,0.00665] | NA | (1.11,Inf] | (0.798,1.72] |
| **60** | (0,0.00179] | (0.00125,0.00292] | (0,0.46] | (0.936,1.11] | (1.72,Inf] |
| **61** | (0.00179,0.00386] | (0.00292,0.00665] | NA | (0.936,1.11] | (0.798,1.72] |
| **62** | (0.00179,0.00386] | (0.00292,0.00665] | NA | (0.936,1.11] | (1.72,Inf] |
| **63** | (0.00179,0.00386] | (0.00292,0.00665] | NA | (0.936,1.11] | (0.798,1.72] |
| **64** | (0.00386,0.00875] | (0.00292,0.00665] | NA | (0.731,0.936] | (0.798,1.72] |
| **65** | (0.00179,0.00386] | (0.00125,0.00292] | (0.46,Inf] | (0.936,1.11] | (0.213,0.798] |
| **66** | (0,0.00179] | (0.00665,Inf] | (0.46,Inf] | (0.731,0.936] | (1.72,Inf] |
| **67** | (0,0.00179] | (0.00125,0.00292] | (0.46,Inf] | (0.936,1.11] | (0.798,1.72] |
| **68** | (0.00179,0.00386] | (0.00125,0.00292] | (0,0.46] | (0.936,1.11] | (0.798,1.72] |
| **69** | NA | NA | NA | (1.11,Inf] | NA |
| **70** | (0.00386,0.00875] | (0.00665,Inf] | (0,0.46] | (0.731,0.936] | (0.798,1.72] |
| **71** | (0,0.00179] | (0.00125,0.00292] | (0.46,Inf] | (0.936,1.11] | (0.798,1.72] |
| **72** | (0.00179,0.00386] | (0.00292,0.00665] | (0.46,Inf] | (1.11,Inf] | (0.798,1.72] |

| | | | | | |
|---|---|---|---|---|---|
| **73** | (0.00179,0.00386] | (0.00292,0.00665] | NA | (0.731,0.936] | (0.798,1.72] |
| **74** | (0,0.00179] | (0.00292,0.00665] | (0.46,Inf] | (0.731,0.936] | (1.72,Inf] |
| **75** | (0.00179,0.00386] | (0.00292,0.00665] | NA | (0.936,1.11] | (1.72,Inf] |
| **76** | (0.00179,0.00386] | (0.00292,0.00665] | NA | (0.936,1.11] | (0.798,1.72] |
| **77** | (0.00179,0.00386] | (0.00125,0.00292] | NA | (1.11,Inf] | (0.798,1.72] |
| **78** | (0,0.00179] | (0.00125,0.00292] | (0.46,Inf] | (0.936,1.11] | (0.798,1.72] |
| **79** | (0.00179,0.00386] | (0.00292,0.00665] | (0.46,Inf] | (0.936,1.11] | (0.798,1.72] |
| **80** | (0.00179,0.00386] | (0.00292,0.00665] | (0.46,Inf] | (0.936,1.11] | (0.798,1.72] |
| **81** | (0.00386,0.00875] | (0.00665,Inf] | (0,0.46] | (0.936,1.11] | (1.72,Inf] |
| **82** | (0.00179,0.00386] | (0.00125,0.00292] | (0.46,Inf] | (1.11,Inf] | (0.798,1.72] |
| **83** | (0.00386,0.00875] | (0.00292,0.00665] | (0.46,Inf] | (0.936,1.11] | (0.798,1.72] |
| **84** | (0.00179,0.00386] | (0.00125,0.00292] | (0.46,Inf] | (0.936,1.11] | (0.798,1.72] |
| **85** | (0.00386,0.00875] | (0.00292,0.00665] | NA | (0.936,1.11] | (0.798,1.72] |
| **86** | (0.00179,0.00386] | (0.00292,0.00665] | NA | (1.11,Inf] | (0.798,1.72] |
| **87** | (0.00179,0.00386] | (0.00125,0.00292] | NA | (1.11,Inf] | (0.798,1.72] |
| **88** | (0.00179,0.00386] | (0.00125,0.00292] | (0.46,Inf] | (1.11,Inf] | (0.798,1.72] |
| **89** | (0,0.00179] | (0,0.00125] | NA | (0.936,1.11] | (0.798,1.72] |
| **90** | (0.00179,0.00386] | (0.00292,0.00665] | (0.46,Inf] | (0.731,0.936] | (0.798,1.72] |
| **91** | (0,0.00179] | (0,0.00125] | NA | (0.936,1.11] | (0.213,0.798] |
| **92** | (0,0.00179] | (0,0.00125] | NA | (0.731,0.936] | (0.213,0.798] |
| **93** | (0.00386,0.00875] | (0.00125,0.00292] | NA | (0.731,0.936] | (0.213,0.798] |
| **94** | (0.00386,0.00875] | (0.00292,0.00665] | (0,0.46] | (0.936,1.11] | (0.798,1.72] |
| **95** | (0,0.00179] | (0,0.00125] | NA | (0.936,1.11] | (0.798,1.72] |
| **96** | (0,0.00179] | (0.00125,0.00292] | NA | (0.731,0.936] | (0.798,1.72] |
| **97** | (0.00179,0.00386] | (0.00292,0.00665] | NA | (0.936,1.11] | (1.72,Inf] |
| **98** | (0.00179,0.00386] | (0.00125,0.00292] | NA | (0.936,1.11] | (0.213,0.798] |
| **99** | (0.00179,0.00386] | (0.00125,0.00292] | (0.46,Inf] | (0.936,1.11] | (0.798,1.72] |
| **100** | (0,0.00179] | (0,0.00125] | NA | (0.731,0.936] | (0.798,1.72] |
| **101** | (0.00179,0.00386] | (0.00125,0.00292] | NA | (0.731,0.936] | (0.213,0.798] |
| **102** | (0.00179,0.00386] | (0,0.00125] | (0,0.46] | (0.936,1.11] | (0.213,0.798] |
| **103** | (0.00386,0.00875] | (0.00125,0.00292] | NA | (0.731,0.936] | (0.213,0.798] |
| **104** | (0,0.00179] | (0.00125,0.00292] | NA | (0.936,1.11] | (1.72,Inf] |
| **105** | (0.00179,0.00386] | (0.00125,0.00292] | NA | (0.936,1.11] | (0.798,1.72] |
| **106** | (0.00386,0.00875] | (0.00125,0.00292] | NA | (1.11,Inf] | (0.213,0.798] |
| **107** | (0,0.00179] | (0,0.00125] | NA | (0,0.731] | (0.213,0.798] |
| **108** | (0.00179,0.00386] | (0.00292,0.00665] | (0.46,Inf] | (0.731,0.936] | (0.798,1.72] |
| **109** | (0.00386,0.00875] | (0.00665,Inf] | NA | (0.731,0.936] | (1.72,Inf] |
| **110** | (0,0.00179] | (0.00125,0.00292] | NA | (0.936,1.11] | (0.798,1.72] |

| | | | | | |
|---|---|---|---|---|---|
| **111** | (0.00386,0.00875] | (0,0.00125] | NA | (1.11,Inf] | (0.213,0.798] |
| **112** | (0.00179,0.00386] | (0,0.00125] | (0.46,Inf] | (0.936,1.11] | (0.213,0.798] |
| **113** | (0.00179,0.00386] | (0.00292,0.00665] | NA | (1.11,Inf] | (0.798,1.72] |
| **114** | (0.00179,0.00386] | (0.00125,0.00292] | NA | (0.936,1.11] | (0.798,1.72] |
| **115** | (0,0.00179] | (0.00125,0.00292] | NA | (0.936,1.11] | (0.798,1.72] |
| **116** | (0.00179,0.00386] | (0.00125,0.00292] | NA | (0.936,1.11] | (0.798,1.72] |
| **117** | (0,0.00179] | (0.00125,0.00292] | NA | (0.936,1.11] | (1.72,Inf] |
| **118** | (0.00386,0.00875] | (0.00292,0.00665] | (0.46,Inf] | (0.936,1.11] | (0.798,1.72] |
| **119** | (0,0.00179] | (0,0.00125] | NA | (0.936,1.11] | (0.213,0.798] |
| **120** | (0.00179,0.00386] | (0.00125,0.00292] | NA | (0.936,1.11] | (0.798,1.72] |
| **121** | (0.00386,0.00875] | (0.00125,0.00292] | NA | (0.936,1.11] | (0.213,0.798] |
| **122** | (0.00386,0.00875] | (0.00292,0.00665] | (0.46,Inf] | (1.11,Inf] | (0.798,1.72] |
| **123** | (0.00386,0.00875] | (0.00292,0.00665] | NA | (0.936,1.11] | (0.798,1.72] |
| **124** | (0.00179,0.00386] | (0.00125,0.00292] | NA | (0.731,0.936] | (0.798,1.72] |
| **125** | (0.00179,0.00386] | (0.00125,0.00292] | (0.46,Inf] | (0.731,0.936] | (0.213,0.798] |
| **126** | (0.00179,0.00386] | (0.00125,0.00292] | NA | (0.731,0.936] | (0.798,1.72] |
| **127** | (0.00179,0.00386] | (0.00125,0.00292] | NA | (0.936,1.11] | (0.798,1.72] |
| **128** | (0.00179,0.00386] | (0.00125,0.00292] | (0,0.46] | (1.11,Inf] | (0.213,0.798] |
| **129** | (0.00179,0.00386] | (0.00125,0.00292] | (0,0.46] | (1.11,Inf] | (0.213,0.798] |
| **130** | (0,0.00179] | (0.00125,0.00292] | NA | (0.936,1.11] | (0.798,1.72] |
| **131** | (0.00386,0.00875] | (0.00292,0.00665] | NA | (1.11,Inf] | (0.798,1.72] |
| **132** | (0.00179,0.00386] | (0.00292,0.00665] | NA | (0.936,1.11] | (0.798,1.72] |
| **133** | (0.00179,0.00386] | (0.00292,0.00665] | NA | (0.731,0.936] | (0.798,1.72] |
| **134** | (0.00179,0.00386] | (0.00125,0.00292] | NA | (0.936,1.11] | (0.798,1.72] |
| **135** | (0,0.00179] | (0,0.00125] | NA | (1.11,Inf] | (0.798,1.72] |
| **136** | (0.00179,0.00386] | (0.00125,0.00292] | NA | (0.731,0.936] | (0.213,0.798] |
| **137** | (0.00179,0.00386] | (0.00292,0.00665] | NA | (0.936,1.11] | (0.798,1.72] |
| **138** | (0.00386,0.00875] | (0.00292,0.00665] | NA | (0.936,1.11] | (0.213,0.798] |
| **139** | (0.00386,0.00875] | (0.00292,0.00665] | NA | (0.936,1.11] | (0.798,1.72] |
| **140** | (0.00179,0.00386] | (0.00292,0.00665] | (0.46,Inf] | (0.936,1.11] | (0.798,1.72] |
| **141** | (0,0.00179] | (0.00125,0.00292] | NA | (0.731,0.936] | (0.798,1.72] |
| **142** | (0.00386,0.00875] | (0.00292,0.00665] | (0.46,Inf] | (1.11,Inf] | (0.798,1.72] |
| **143** | (0.00386,0.00875] | (0.00665,Inf] | NA | (0.936,1.11] | (1.72,Inf] |
| **144** | (0.00179,0.00386] | (0.00125,0.00292] | NA | (0.936,1.11] | (0.213,0.798] |
| **145** | (0.00386,0.00875] | (0.00292,0.00665] | NA | (0.731,0.936] | (0.213,0.798] |
| **146** | (0,0.00179] | (0.00125,0.00292] | NA | (0.731,0.936] | (0.798,1.72] |
| **147** | (0.00179,0.00386] | (0.00125,0.00292] | (0.46,Inf] | (1.11,Inf] | (0.798,1.72] |
| **148** | (0,0.00179] | (0.00125,0.00292] | NA | (0.936,1.11] | (0.798,1.72] |

| | | | | | |
|---|---|---|---|---|---|
| **149** | (0.00179,0.00386] | (0.00125,0.00292] | (0,0.46] | (0.936,1.11] | (0.213,0.798] |
| **150** | (0.00179,0.00386] | (0.00125,0.00292] | NA | (0.936,1.11] | (0.798,1.72] |
| **151** | (0.00179,0.00386] | (0.00125,0.00292] | (0,0.46] | (0.936,1.11] | (0.798,1.72] |
| **152** | (0.00386,0.00875] | (0.00125,0.00292] | NA | (0.936,1.11] | (0.213,0.798] |
| **153** | (0.00386,0.00875] | (0.00125,0.00292] | (0,0.46] | (0.936,1.11] | (0.213,0.798] |
| **154** | (0,0.00179] | (0.00125,0.00292] | (0,0.46] | (0.936,1.11] | (0.798,1.72] |
| **155** | (0.00179,0.00386] | (0,0.00125] | NA | (0.731,0.936] | (0.213,0.798] |
| **156** | (0.00386,0.00875] | (0.00292,0.00665] | NA | (0.936,1.11] | (0.798,1.72] |
| **157** | (0.00179,0.00386] | (0.00125,0.00292] | (0.46,Inf] | (1.11,Inf] | (0.798,1.72] |
| **158** | (0.00386,0.00875] | (0.00665,Inf] | NA | (0.936,1.11] | (0.798,1.72] |
| **159** | (0,0.00179] | (0.00292,0.00665] | (0,0.46] | (0.936,1.11] | (1.72,Inf] |
| **160** | (0.00386,0.00875] | (0.00292,0.00665] | (0.46,Inf] | (0.731,0.936] | (0.213,0.798] |
| **161** | (0,0.00179] | (0.00125,0.00292] | NA | (0.936,1.11] | (0.798,1.72] |
| **162** | (0.00386,0.00875] | (0.00292,0.00665] | NA | (1.11,Inf] | (0.798,1.72] |
| **163** | (0.00179,0.00386] | (0.00292,0.00665] | NA | (0.731,0.936] | (0.798,1.72] |
| **164** | (0,0.00179] | (0.00125,0.00292] | NA | (0.731,0.936] | (0.798,1.72] |
| **165** | (0,0.00179] | (0.00125,0.00292] | NA | (0.936,1.11] | (0.798,1.72] |
| **166** | (0.00386,0.00875] | (0.00125,0.00292] | NA | (1.11,Inf] | (0.213,0.798] |
| **167** | (0,0.00179] | (0.00125,0.00292] | NA | (0.936,1.11] | (0.798,1.72] |
| **168** | (0,0.00179] | (0.00665,Inf] | NA | (0,0.731] | (1.72,Inf] |
| **169** | (0.00386,0.00875] | (0.00665,Inf] | (0.46,Inf] | (0.936,1.11] | (0.798,1.72] |
| **170** | (0.00386,0.00875] | (0.00292,0.00665] | NA | (0.936,1.11] | (0.798,1.72] |
| **171** | (0,0.00179] | (0,0.00125] | (0.46,Inf] | (1.11,Inf] | (0.798,1.72] |
| **172** | (0.00179,0.00386] | (0.00125,0.00292] | NA | (1.11,Inf] | (0.798,1.72] |
| **173** | (0.00179,0.00386] | (0.00125,0.00292] | NA | (0.936,1.11] | (0.798,1.72] |
| **174** | (0.00179,0.00386] | (0.00292,0.00665] | NA | (0.731,0.936] | (0.798,1.72] |
| **175** | (0.00179,0.00386] | (0.00125,0.00292] | NA | (0.731,0.936] | (0.213,0.798] |
| **176** | (0.00875,Inf] | (0.00292,0.00665] | NA | (0.936,1.11] | (0.213,0.798] |
| **177** | (0.00386,0.00875] | (0.00292,0.00665] | NA | (0.731,0.936] | (0.213,0.798] |
| **178** | (0.00875,Inf] | (0.00665,Inf] | NA | (0.731,0.936] | (0.798,1.72] |
| **179** | (0.00386,0.00875] | (0.00125,0.00292] | NA | (0.936,1.11] | (0.213,0.798] |
| **180** | (0.00179,0.00386] | (0.00125,0.00292] | NA | (1.11,Inf] | (0.798,1.72] |
| **181** | (0.00179,0.00386] | (0.00125,0.00292] | (0,0.46] | (0.936,1.11] | (0.798,1.72] |
| **182** | (0.00386,0.00875] | (0.00125,0.00292] | (0.46,Inf] | (0.731,0.936] | (0.213,0.798] |
| **183** | (0,0.00179] | (0.00125,0.00292] | NA | (0.731,0.936] | (0.798,1.72] |
| **184** | (0.00386,0.00875] | (0.00292,0.00665] | NA | (1.11,Inf] | (0.798,1.72] |
| **185** | (0.00179,0.00386] | (0.00125,0.00292] | NA | (0.731,0.936] | (0.798,1.72] |
| **186** | (0,0.00179] | (0.00665,Inf] | NA | (0.731,0.936] | (1.72,Inf] |

| | | | | | |
|---|---|---|---|---|---|
| **187** | (0,0.00179] | (0,0.00125] | NA | (0.936,1.11] | (0.798,1.72] |
| **188** | (0.00179,0.00386] | (0.00292,0.00665] | NA | (0.731,0.936] | (0.798,1.72] |
| **189** | (0.00179,0.00386] | (0.00292,0.00665] | NA | (1.11,Inf] | (0.798,1.72] |
| **190** | (0.00179,0.00386] | (0.00125,0.00292] | NA | (0.936,1.11] | (0.798,1.72] |
| **191** | (0.00386,0.00875] | (0.00292,0.00665] | NA | (1.11,Inf] | (0.798,1.72] |
| **192** | (0.00179,0.00386] | (0.00292,0.00665] | NA | (1.11,Inf] | (0.798,1.72] |
| **193** | (0.00179,0.00386] | (0.00125,0.00292] | NA | (0.731,0.936] | (0.213,0.798] |
| **194** | (0.00875,Inf] | (0.00292,0.00665] | NA | (0.936,1.11] | (0.213,0.798] |
| **195** | (0.00386,0.00875] | (0.00292,0.00665] | NA | (0.731,0.936] | (0.798,1.72] |
| **196** | (0,0.00179] | (0,0.00125] | NA | (0.936,1.11] | (0.213,0.798] |
| **197** | (0.00179,0.00386] | (0.00292,0.00665] | NA | (0.936,1.11] | (0.798,1.72] |
| **198** | (0.00179,0.00386] | (0.00125,0.00292] | NA | (0.936,1.11] | (0.798,1.72] |
| **199** | (0.00386,0.00875] | (0.00125,0.00292] | (0.46,Inf] | (0.936,1.11] | (0.213,0.798] |
| **200** | (0.00875,Inf] | (0.00665,Inf] | NA | (0.936,1.11] | (0.213,0.798] |
| **201** | (0.00179,0.00386] | (0.00665,Inf] | NA | (0.936,1.11] | (1.72,Inf] |
| **202** | (0.00386,0.00875] | (0.00125,0.00292] | NA | (0.731,0.936] | (0.213,0.798] |
| **203** | (0.00179,0.00386] | (0.00292,0.00665] | NA | (0.731,0.936] | (0.798,1.72] |
| **204** | (0.00179,0.00386] | (0.00292,0.00665] | NA | (0.731,0.936] | (0.798,1.72] |
| **205** | (0.00386,0.00875] | (0.00125,0.00292] | NA | (1.11,Inf] | (0.213,0.798] |
| **206** | (0.00179,0.00386] | (0.00292,0.00665] | NA | (0.936,1.11] | (0.798,1.72] |
| **207** | (0.00179,0.00386] | (0.00292,0.00665] | NA | (1.11,Inf] | (0.798,1.72] |
| **208** | (0,0.00179] | (0.00125,0.00292] | (0.46,Inf] | (0.731,0.936] | (0.798,1.72] |
| **209** | (0.00179,0.00386] | (0.00125,0.00292] | NA | (0.731,0.936] | (0.213,0.798] |
| **210** | (0.00386,0.00875] | (0.00292,0.00665] | NA | (0.731,0.936] | (0.213,0.798] |
| **211** | (0.00875,Inf] | (0.00665,Inf] | NA | (1.11,Inf] | (0.798,1.72] |
| **212** | (0,0.00179] | (0.00125,0.00292] | NA | (0.936,1.11] | (0.798,1.72] |
| **213** | NA | NA | NA | (0.731,0.936] | NA |
| **214** | (0.00386,0.00875] | (0.00292,0.00665] | NA | (1.11,Inf] | (0.798,1.72] |
| **215** | (0.00179,0.00386] | (0.00125,0.00292] | NA | (0.731,0.936] | (0.213,0.798] |
| **216** | (0.00386,0.00875] | (0.00292,0.00665] | (0.46,Inf] | (0.731,0.936] | (0.798,1.72] |
| **217** | (0,0.00179] | (0,0.00125] | NA | (0.936,1.11] | (0.213,0.798] |
| **218** | (0.00386,0.00875] | (0.00292,0.00665] | (0,0.46] | (0.936,1.11] | (0.798,1.72] |
| **219** | (0.00386,0.00875] | (0.00292,0.00665] | NA | (0.936,1.11] | (0.213,0.798] |
| **220** | (0.00179,0.00386] | (0.00125,0.00292] | NA | (0.936,1.11] | (0.798,1.72] |
| **221** | (0.00386,0.00875] | (0.00292,0.00665] | NA | (1.11,Inf] | (0.798,1.72] |
| **222** | (0.00179,0.00386] | (0.00125,0.00292] | (0.46,Inf] | (0.936,1.11] | (0.213,0.798] |
| **223** | (0,0.00179] | (0.00125,0.00292] | NA | (0.936,1.11] | (0.798,1.72] |
| **224** | (0,0.00179] | (0.00125,0.00292] | NA | (0.936,1.11] | (0.798,1.72] |

| | | | | | |
|---|---|---|---|---|---|
| **225** | (0.00179,0.00386] | (0,0.00125] | NA | (0.936,1.11] | (0.213,0.798] |
| **226** | (0,0.00179] | (0.00125,0.00292] | NA | (0.731,0.936] | (0.798,1.72] |
| **227** | (0.00179,0.00386] | (0.00665,Inf] | (0.46,Inf] | (0.936,1.11] | (1.72,Inf] |
| **228** | (0.00386,0.00875] | (0.00125,0.00292] | NA | (0.936,1.11] | (0.213,0.798] |
| **229** | (0,0.00179] | (0.00125,0.00292] | NA | (0.731,0.936] | (0.798,1.72] |
| **230** | NA | NA | NA | NA | (0.798,1.72] |
| **231** | (0.00179,0.00386] | (0,0.00125] | (0.46,Inf] | (0.731,0.936] | (0.213,0.798] |
| **232** | (0,0.00179] | (0.00125,0.00292] | NA | (0.936,1.11] | (0.798,1.72] |
| **233** | (0.00179,0.00386] | (0.00125,0.00292] | NA | (0.731,0.936] | (0.213,0.798] |
| **234** | (0,0.00179] | (0.00125,0.00292] | NA | (0.936,1.11] | (1.72,Inf] |
| **235** | (0.00179,0.00386] | (0.00292,0.00665] | NA | (0.731,0.936] | (0.798,1.72] |
| **236** | (0.00875,Inf] | (0.00665,Inf] | NA | (0.936,1.11] | (0.213,0.798] |
| **237** | (0.00179,0.00386] | (0.00292,0.00665] | NA | (0.731,0.936] | (0.798,1.72] |
| **238** | (0.00386,0.00875] | (0.00125,0.00292] | NA | (0.936,1.11] | (0.213,0.798] |
| **239** | (0.00179,0.00386] | (0.00292,0.00665] | NA | (0.936,1.11] | (0.798,1.72] |
| **240** | (0.00386,0.00875] | (0.00125,0.00292] | NA | (1.11,Inf] | (0.213,0.798] |
| **241** | (0.00875,Inf] | (0.00665,Inf] | NA | (0.936,1.11] | (0.213,0.798] |
| **242** | (0,0.00179] | (0.00125,0.00292] | NA | (0.731,0.936] | (0.798,1.72] |
| **243** | (0.00179,0.00386] | (0.00292,0.00665] | NA | (0.731,0.936] | (0.798,1.72] |
| **244** | (0.00386,0.00875] | (0.00292,0.00665] | NA | (0.936,1.11] | (0.213,0.798] |
| **245** | (0.00179,0.00386] | (0.00125,0.00292] | NA | (0.731,0.936] | (0.213,0.798] |
| **246** | (0.00179,0.00386] | (0.00125,0.00292] | (0,0.46] | (0.731,0.936] | (0.213,0.798] |
| **247** | (0.00386,0.00875] | (0.00292,0.00665] | NA | (0.936,1.11] | (0.798,1.72] |
| **248** | (0,0.00179] | (0,0.00125] | NA | (0.936,1.11] | (0.798,1.72] |
| **249** | (0.00386,0.00875] | (0.00292,0.00665] | NA | (0.936,1.11] | (0.213,0.798] |
| **250** | (0.00179,0.00386] | (0.00292,0.00665] | NA | (0.936,1.11] | (0.798,1.72] |
| **251** | (0.00179,0.00386] | (0.00125,0.00292] | NA | (0.936,1.11] | (0.798,1.72] |
| **252** | (0,0.00179] | (0,0.00125] | NA | (1.11,Inf] | (0.213,0.798] |
| **253** | (0,0.00179] | (0,0.00125] | NA | (1.11,Inf] | (0.798,1.72] |
| **254** | (0.00179,0.00386] | (0.00292,0.00665] | NA | (0,0.731] | (1.72,Inf] |
| **255** | (0.00386,0.00875] | (0,0.00125] | NA | (0.936,1.11] | (0,0.213] |
| **256** | (0.00386,0.00875] | (0,0.00125] | NA | (0.936,1.11] | (0,0.213] |
| **257** | (0.00386,0.00875] | (0.00292,0.00665] | (0.46,Inf] | (0.936,1.11] | (0.798,1.72] |
| **258** | (0.00386,0.00875] | (0.00292,0.00665] | NA | (0.936,1.11] | (0.213,0.798] |
| **259** | (0,0.00179] | (0.00125,0.00292] | (0,0.46] | (0.936,1.11] | (0.798,1.72] |
| **260** | (0.00179,0.00386] | (0.00292,0.00665] | (0.46,Inf] | (0.731,0.936] | (0.798,1.72] |
| **261** | (0,0.00179] | (0,0.00125] | (0.46,Inf] | (0.731,0.936] | (0.213,0.798] |
| **262** | (0,0.00179] | (0.00125,0.00292] | NA | (0.936,1.11] | (0.798,1.72] |

| | | | | | |
|---|---|---|---|---|---|
| **263** | (0,0.00179] | (0.00125,0.00292] | (0.46,Inf] | (0.731,0.936] | (0.798,1.72] |
| **264** | (0.00179,0.00386] | (0.00125,0.00292] | NA | (0.936,1.11] | (0.213,0.798] |
| **265** | (0,0.00179] | (0.00125,0.00292] | NA | (0.731,0.936] | (0.213,0.798] |
| **266** | (0,0.00179] | (0.00125,0.00292] | NA | (0.936,1.11] | (0.798,1.72] |
| **267** | (0.00179,0.00386] | (0.00125,0.00292] | NA | (0,0.731] | (0.213,0.798] |
| **268** | (0.00386,0.00875] | (0.00292,0.00665] | NA | (0.936,1.11] | (0.798,1.72] |
| **269** | (0.00179,0.00386] | (0.00125,0.00292] | NA | (0.936,1.11] | (0.213,0.798] |
| **270** | (0,0.00179] | (0.00125,0.00292] | NA | (0.936,1.11] | (0.798,1.72] |
| **271** | (0.00875,Inf] | (0.00665,Inf] | NA | (1.11,Inf] | (0.798,1.72] |
| **272** | (0.00386,0.00875] | (0.00125,0.00292] | NA | (1.11,Inf] | (0.213,0.798] |
| **273** | (0.00386,0.00875] | (0.00125,0.00292] | NA | (0.936,1.11] | (0,0.213] |
| **274** | (0.00386,0.00875] | (0.00665,Inf] | (0,0.46] | (0.731,0.936] | (0.798,1.72] |
| **275** | (0.00179,0.00386] | (0.00125,0.00292] | NA | (0.731,0.936] | (0.213,0.798] |
| **276** | (0.00386,0.00875] | (0.00292,0.00665] | NA | (0.936,1.11] | (0.798,1.72] |
| **277** | (0,0.00179] | (0.00125,0.00292] | NA | (0.731,0.936] | (0.798,1.72] |
| **278** | (0.00179,0.00386] | (0,0.00125] | NA | (0.936,1.11] | (0.213,0.798] |
| **279** | (0,0.00179] | (0.00125,0.00292] | NA | (0.731,0.936] | (0.798,1.72] |
| **280** | (0,0.00179] | (0.00125,0.00292] | NA | (0.731,0.936] | (0.798,1.72] |
| **281** | (0.00386,0.00875] | (0.00292,0.00665] | (0,0.46] | (0.936,1.11] | (0.213,0.798] |
| **282** | (0.00179,0.00386] | (0.00292,0.00665] | NA | (0.936,1.11] | (0.798,1.72] |
| **283** | (0.00179,0.00386] | (0.00292,0.00665] | NA | (0.731,0.936] | (0.798,1.72] |
| **284** | (0.00179,0.00386] | (0.00125,0.00292] | NA | (0.731,0.936] | (0.213,0.798] |
| **285** | (0.00386,0.00875] | (0.00292,0.00665] | NA | (0.936,1.11] | (0.213,0.798] |
| **286** | (0,0.00179] | NA | (0.46,Inf] | (0,0.731] | NA |
| **287** | (0,0.00179] | (0.00125,0.00292] | NA | (0.731,0.936] | (0.798,1.72] |
| **288** | (0.00179,0.00386] | (0.00125,0.00292] | NA | (0.731,0.936] | (0.213,0.798] |
| **289** | (0.00386,0.00875] | (0.00292,0.00665] | NA | (1.11,Inf] | (0.798,1.72] |
| **290** | (0.00386,0.00875] | (0.00125,0.00292] | NA | (0.936,1.11] | (0.213,0.798] |
| **291** | (0.00875,Inf] | (0.00292,0.00665] | (0,0.46] | (1.11,Inf] | (0.213,0.798] |
| **292** | (0.00179,0.00386] | (0.00125,0.00292] | NA | (0.936,1.11] | (0.798,1.72] |
| **293** | (0.00179,0.00386] | (0.00125,0.00292] | NA | (0.731,0.936] | (0.213,0.798] |
| **294** | (0.00179,0.00386] | (0.00125,0.00292] | NA | (1.11,Inf] | (0.798,1.72] |
| **295** | (0.00875,Inf] | (0.00665,Inf] | (0.46,Inf] | (1.11,Inf] | (0.798,1.72] |
| **296** | (0.00386,0.00875] | (0.00125,0.00292] | NA | (0.731,0.936] | (0.213,0.798] |
| **297** | (0,0.00179] | (0.00125,0.00292] | NA | (0.936,1.11] | (0.798,1.72] |
| **298** | (0.00179,0.00386] | (0.00125,0.00292] | NA | (0.936,1.11] | (0.213,0.798] |
| **299** | (0.00875,Inf] | (0.00292,0.00665] | NA | (0.936,1.11] | (0.213,0.798] |
| **300** | (0.00386,0.00875] | (0.00125,0.00292] | NA | (0.731,0.936] | (0.213,0.798] |

| | | | | | |
|---|---|---|---|---|---|
| **301** | (0,0.00179] | (0.00125,0.00292] | NA | (0.731,0.936] | (0.798,1.72] |
| **302** | (0,0.00179] | (0.00125,0.00292] | NA | (0.731,0.936] | (0.798,1.72] |
| **303** | (0.00179,0.00386] | (0.00292,0.00665] | NA | (0.731,0.936] | (0.798,1.72] |
| **304** | (0.00179,0.00386] | (0.00125,0.00292] | NA | (0.731,0.936] | (0.213,0.798] |
| **305** | (0.00875,Inf] | (0.00125,0.00292] | (0.46,Inf] | (0.936,1.11] | (0,0.213] |
| **306** | (0.00179,0.00386] | (0.00125,0.00292] | NA | (0.731,0.936] | (0.798,1.72] |
| **307** | (0.00179,0.00386] | (0,0.00125] | NA | (0.936,1.11] | (0,0.213] |
| **308** | (0.00179,0.00386] | (0.00125,0.00292] | NA | (1.11,Inf] | (0.798,1.72] |
| **309** | (0.00179,0.00386] | (0.00125,0.00292] | NA | (0.936,1.11] | (0.213,0.798] |
| **310** | (0.00179,0.00386] | (0.00125,0.00292] | NA | (1.11,Inf] | (0.798,1.72] |
| **311** | (0.00179,0.00386] | (0,0.00125] | NA | (0.936,1.11] | (0.213,0.798] |
| **312** | (0.00179,0.00386] | (0.00125,0.00292] | NA | (0.731,0.936] | (0.213,0.798] |
| **313** | (0,0.00179] | (0,0.00125] | NA | (0.936,1.11] | (0.213,0.798] |
| **314** | (0.00179,0.00386] | (0.00292,0.00665] | NA | (0.731,0.936] | (0.798,1.72] |
| **315** | (0.00179,0.00386] | (0,0.00125] | NA | (1.11,Inf] | (0.213,0.798] |
| **316** | (0,0.00179] | (0,0.00125] | NA | (0.936,1.11] | (0.798,1.72] |
| **317** | (0.00179,0.00386] | (0.00125,0.00292] | NA | (0.936,1.11] | (0.213,0.798] |
| **318** | (0,0.00179] | (0.00125,0.00292] | NA | (0.731,0.936] | (1.72,Inf] |
| **319** | (0.00179,0.00386] | (0.00292,0.00665] | NA | (0.936,1.11] | (0.798,1.72] |
| **320** | (0,0.00179] | (0,0.00125] | NA | (0.936,1.11] | (0.798,1.72] |
| **321** | (0.00179,0.00386] | (0.00125,0.00292] | NA | (0.936,1.11] | (0.213,0.798] |
| **322** | (0,0.00179] | (0.00125,0.00292] | NA | (0.731,0.936] | (0.798,1.72] |
| **323** | (0.00179,0.00386] | (0.00292,0.00665] | NA | (0.731,0.936] | (1.72,Inf] |
| **324** | (0.00386,0.00875] | (0.00292,0.00665] | (0.46,Inf] | (0.936,1.11] | (0.798,1.72] |
| **325** | (0.00179,0.00386] | (0.00292,0.00665] | NA | (0.936,1.11] | (0.798,1.72] |
| **326** | (0.00386,0.00875] | (0.00292,0.00665] | NA | (0.936,1.11] | (0.213,0.798] |
| **327** | (0.00179,0.00386] | (0,0.00125] | (0.46,Inf] | (1.11,Inf] | (0.213,0.798] |
| **328** | (0.00386,0.00875] | (0.00125,0.00292] | NA | (0.936,1.11] | (0.213,0.798] |
| **329** | (0.00386,0.00875] | (0.00292,0.00665] | NA | (1.11,Inf] | (0.798,1.72] |
| **330** | (0.00386,0.00875] | (0.00292,0.00665] | NA | (0.936,1.11] | (0.213,0.798] |
| **331** | (0.00179,0.00386] | (0.00125,0.00292] | NA | (0.731,0.936] | (0.798,1.72] |
| **332** | (0.00179,0.00386] | (0.00125,0.00292] | NA | (0.731,0.936] | (0.798,1.72] |
| **333** | (0.00386,0.00875] | (0.00125,0.00292] | (0.46,Inf] | (0.731,0.936] | (0.213,0.798] |
| **334** | (0.00179,0.00386] | (0.00125,0.00292] | NA | (0.936,1.11] | (0.798,1.72] |
| **335** | (0.00179,0.00386] | (0.00125,0.00292] | (0,0.46] | (0.731,0.936] | (0.798,1.72] |
| **336** | (0.00386,0.00875] | (0.00665,Inf] | NA | (0.936,1.11] | (0.798,1.72] |
| **337** | (0.00179,0.00386] | (0.00125,0.00292] | NA | (0.731,0.936] | (0.213,0.798] |
| **338** | (0.00386,0.00875] | (0.00292,0.00665] | (0.46,Inf] | (0.936,1.11] | (0.798,1.72] |

| | | | | | |
|---|---|---|---|---|---|
| **339** | (0.00386,0.00875] | (0.00292,0.00665] | NA | (0.731,0.936] | (0.213,0.798] |
| **340** | (0,0.00179] | (0.00125,0.00292] | NA | (0.731,0.936] | (0.798,1.72] |
| **341** | (0,0.00179] | (0.00125,0.00292] | NA | (0.731,0.936] | (0.798,1.72] |
| **342** | (0.00179,0.00386] | (0.00125,0.00292] | (0,0.46] | (0.731,0.936] | (0.798,1.72] |
| **343** | (0,0.00179] | (0.00125,0.00292] | NA | (0.731,0.936] | (0.798,1.72] |
| **344** | (0.00179,0.00386] | (0.00125,0.00292] | NA | (0.936,1.11] | (0.798,1.72] |
| **345** | (0.00179,0.00386] | (0.00665,Inf] | NA | (0.731,0.936] | (1.72,Inf] |
| **346** | (0.00386,0.00875] | (0.00665,Inf] | NA | (0.731,0.936] | (0.798,1.72] |
| **347** | (0.00179,0.00386] | (0.00125,0.00292] | NA | (0.936,1.11] | (0.213,0.798] |
| **348** | (0.00179,0.00386] | (0.00125,0.00292] | NA | (0.731,0.936] | (0.798,1.72] |
| **349** | (0.00179,0.00386] | (0.00125,0.00292] | NA | (0.731,0.936] | (0.798,1.72] |
| **350** | (0,0.00179] | (0,0.00125] | NA | (0,0.731] | (0.213,0.798] |
| **351** | (0.00179,0.00386] | (0.00125,0.00292] | NA | (1.11,Inf] | (0.798,1.72] |
| **352** | (0,0.00179] | (0.00125,0.00292] | NA | (0.936,1.11] | (0.798,1.72] |
| **353** | (0,0.00179] | (0.00125,0.00292] | NA | (0.731,0.936] | (0.798,1.72] |
| **354** | (0.00179,0.00386] | (0.00125,0.00292] | (0,0.46] | (1.11,Inf] | (0.798,1.72] |
| **355** | (0.00179,0.00386] | (0.00292,0.00665] | NA | (0.936,1.11] | (0.798,1.72] |
| **356** | (0.00386,0.00875] | (0.00125,0.00292] | NA | (1.11,Inf] | (0.213,0.798] |
| **357** | (0,0.00179] | (0.00125,0.00292] | NA | (0.731,0.936] | (0.798,1.72] |
| **358** | (0.00875,Inf] | (0.00292,0.00665] | NA | (0.936,1.11] | (0.213,0.798] |
| **359** | (0,0.00179] | (0.00125,0.00292] | NA | (0.936,1.11] | (0.798,1.72] |
| **360** | (0.00386,0.00875] | (0.00125,0.00292] | NA | (0.936,1.11] | (0.213,0.798] |
| **361** | (0.00386,0.00875] | (0.00292,0.00665] | NA | (1.11,Inf] | (0.798,1.72] |
| **362** | (0.00386,0.00875] | (0.00292,0.00665] | NA | (0.936,1.11] | (0.798,1.72] |
| **363** | (0,0.00179] | (0.00125,0.00292] | (0.46,Inf] | (0,0.731] | (0.798,1.72] |
| **364** | (0,0.00179] | (0.00125,0.00292] | (0.46,Inf] | (0,0.731] | (0.798,1.72] |
| **365** | (0.00386,0.00875] | (0.00292,0.00665] | (0.46,Inf] | (0.731,0.936] | (0.798,1.72] |
| **366** | (0.00386,0.00875] | (0.00125,0.00292] | NA | (1.11,Inf] | (0.213,0.798] |
| **367** | (0.00386,0.00875] | (0.00125,0.00292] | NA | (0.936,1.11] | (0.213,0.798] |
| **368** | (0,0.00179] | (0.00125,0.00292] | NA | (0.936,1.11] | (0.798,1.72] |
| **369** | (0.00179,0.00386] | (0.00125,0.00292] | NA | (1.11,Inf] | (0.798,1.72] |
| **370** | (0.00179,0.00386] | (0.00125,0.00292] | NA | (0.731,0.936] | (0.798,1.72] |
| **371** | (0.00179,0.00386] | (0,0.00125] | NA | (0.731,0.936] | (0.213,0.798] |
| **372** | (0,0.00179] | (0.00125,0.00292] | NA | (1.11,Inf] | (1.72,Inf] |
| **373** | (0.00386,0.00875] | (0.00292,0.00665] | NA | (1.11,Inf] | (0.798,1.72] |
| **374** | (0,0.00179] | (0.00125,0.00292] | NA | (0.936,1.11] | (1.72,Inf] |
| **375** | (0.00179,0.00386] | (0.00125,0.00292] | (0.46,Inf] | (0.936,1.11] | (0.798,1.72] |
| **376** | (0.00179,0.00386] | (0.00292,0.00665] | NA | (0.936,1.11] | (0.798,1.72] |

| | | | | | |
|---|---|---|---|---|---|
| **377** | (0.00179,0.00386] | (0.00125,0.00292] | NA | (0.731,0.936] | (0.798,1.72] |
| **378** | (0.00386,0.00875] | (0.00292,0.00665] | NA | (1.11,Inf] | (0.798,1.72] |
| **379** | (0.00179,0.00386] | (0.00292,0.00665] | NA | (1.11,Inf] | (0.798,1.72] |
| **380** | (0.00179,0.00386] | (0.00125,0.00292] | NA | (1.11,Inf] | (0.798,1.72] |
| **381** | (0.00179,0.00386] | (0.00125,0.00292] | NA | (0.936,1.11] | (0.213,0.798] |
| **382** | (0.00179,0.00386] | (0.00125,0.00292] | NA | (0.936,1.11] | (0.798,1.72] |
| **383** | (0.00386,0.00875] | (0.00665,Inf] | NA | (1.11,Inf] | (0.798,1.72] |
| **384** | (0.00179,0.00386] | (0.00125,0.00292] | NA | (0.936,1.11] | (0.798,1.72] |
| **385** | (0.00386,0.00875] | (0.00292,0.00665] | (0,0.46] | (1.11,Inf] | (0.798,1.72] |
| **386** | (0.00179,0.00386] | (0.00125,0.00292] | (0.46,Inf] | (0.731,0.936] | (0.798,1.72] |
| **387** | (0.00386,0.00875] | (0.00665,Inf] | NA | (0.936,1.11] | (0.798,1.72] |
| **388** | NA | NA | NA | (0.731,0.936] | NA |
| **389** | (0.00386,0.00875] | (0.00125,0.00292] | NA | (0.936,1.11] | (0.213,0.798] |
| **390** | (0,0.00179] | (0.00292,0.00665] | NA | (0.731,0.936] | (1.72,Inf] |
| **391** | NA | NA | NA | (0.731,0.936] | NA |
| **392** | (0.00179,0.00386] | (0.00125,0.00292] | NA | (0.731,0.936] | (0.798,1.72] |
| **393** | (0.00179,0.00386] | (0.00292,0.00665] | NA | (0.731,0.936] | (0.798,1.72] |
| **394** | (0.00875,Inf] | (0.00665,Inf] | NA | (1.11,Inf] | (0.213,0.798] |
| **395** | (0.00179,0.00386] | (0.00125,0.00292] | NA | (0.731,0.936] | (0.213,0.798] |
| **396** | (0,0.00179] | (0.00125,0.00292] | NA | (0.936,1.11] | (0.798,1.72] |
| **397** | (0.00179,0.00386] | (0.00125,0.00292] | NA | (0.936,1.11] | (0.213,0.798] |
| **398** | (0,0.00179] | (0,0.00125] | NA | (1.11,Inf] | (0.798,1.72] |
| **399** | (0.00179,0.00386] | (0.00125,0.00292] | (0.46,Inf] | (0.731,0.936] | (0.213,0.798] |
| **400** | (0.00875,Inf] | (0.00665,Inf] | NA | (0,0.731] | (0.798,1.72] |
| **401** | (0,0.00179] | (0.00125,0.00292] | NA | (0.936,1.11] | (0.213,0.798] |
| **402** | (0.00179,0.00386] | (0.00292,0.00665] | NA | (0.731,0.936] | (0.213,0.798] |
| **403** | (0,0.00179] | (0,0.00125] | NA | (0.731,0.936] | (0.213,0.798] |
| **404** | (0.00179,0.00386] | (0.00292,0.00665] | (0.46,Inf] | (0.936,1.11] | (0.798,1.72] |
| **405** | (0.00875,Inf] | (0.00665,Inf] | NA | (0.936,1.11] | (0.798,1.72] |
| **406** | NA | NA | NA | (1.11,Inf] | NA |
| **407** | (0.00179,0.00386] | (0.00125,0.00292] | NA | (1.11,Inf] | (0.213,0.798] |
| **408** | (0.00179,0.00386] | (0.00292,0.00665] | NA | (0.936,1.11] | (0.798,1.72] |
| **409** | (0.00386,0.00875] | (0.00125,0.00292] | (0.46,Inf] | (0.731,0.936] | (0.213,0.798] |
| **410** | (0,0.00179] | (0.00125,0.00292] | NA | (0.936,1.11] | (0.798,1.72] |
| **411** | (0.00875,Inf] | (0.00665,Inf] | NA | (0.731,0.936] | (0.798,1.72] |
| **412** | (0.00875,Inf] | (0.00292,0.00665] | NA | (1.11,Inf] | (0.213,0.798] |
| **413** | (0,0.00179] | (0,0.00125] | NA | (0.731,0.936] | (0.213,0.798] |
| **414** | (0.00386,0.00875] | (0.00665,Inf] | NA | (0.731,0.936] | (0.798,1.72] |

| | | | | | |
|---|---|---|---|---|---|
| **415** | (0,0.00179] | (0.00125,0.00292] | NA | (0.936,1.11] | (0.798,1.72] |
| **416** | (0.00179,0.00386] | (0.00292,0.00665] | NA | (0.936,1.11] | (0.798,1.72] |
| **417** | (0.00386,0.00875] | (0.00292,0.00665] | NA | (0,0.731] | (0.213,0.798] |
| **418** | (0.00179,0.00386] | (0.00292,0.00665] | (0.46,Inf] | (0.936,1.11] | (0.798,1.72] |
| **419** | (0.00179,0.00386] | (0.00292,0.00665] | NA | (0.731,0.936] | (0.798,1.72] |
| **420** | (0.00179,0.00386] | (0.00125,0.00292] | NA | (1.11,Inf] | (0.798,1.72] |
| **421** | (0.00179,0.00386] | (0.00125,0.00292] | NA | (0.731,0.936] | (0.213,0.798] |
| **422** | (0.00179,0.00386] | (0,0.00125] | NA | (1.11,Inf] | (0,0.213] |
| **423** | (0.00386,0.00875] | (0.00292,0.00665] | NA | (0.936,1.11] | (0.798,1.72] |
| **424** | (0,0.00179] | (0,0.00125] | NA | (0.731,0.936] | (0.213,0.798] |
| **425** | (0.00179,0.00386] | (0.00125,0.00292] | NA | (1.11,Inf] | (0.213,0.798] |
| **426** | (0.00386,0.00875] | (0.00292,0.00665] | NA | (0.936,1.11] | (0.798,1.72] |
| **427** | (0.00386,0.00875] | (0.00125,0.00292] | NA | (0.936,1.11] | (0.213,0.798] |
| **428** | (0.00386,0.00875] | (0.00292,0.00665] | NA | (1.11,Inf] | (0.798,1.72] |
| **429** | (0.00386,0.00875] | (0.00292,0.00665] | NA | (1.11,Inf] | (0.798,1.72] |
| **430** | (0.00875,Inf] | (0.00292,0.00665] | (0,0.46] | (0.731,0.936] | (0.213,0.798] |
| **431** | (0.00386,0.00875] | (0.00125,0.00292] | NA | (0.731,0.936] | (0.213,0.798] |
| **432** | (0.00386,0.00875] | (0.00292,0.00665] | NA | (0.731,0.936] | (0.213,0.798] |
| **433** | (0,0.00179] | (0,0.00125] | NA | (0.731,0.936] | (0.798,1.72] |
| **434** | (0.00179,0.00386] | (0.00125,0.00292] | (0.46,Inf] | (0.731,0.936] | (0.213,0.798] |
| **435** | (0.00875,Inf] | (0.00665,Inf] | (0.46,Inf] | (1.11,Inf] | (0.213,0.798] |
| **436** | (0.00386,0.00875] | (0.00292,0.00665] | (0.46,Inf] | (0.731,0.936] | (0.798,1.72] |
| **437** | (0.00386,0.00875] | (0.00292,0.00665] | NA | (0.936,1.11] | (0.213,0.798] |
| **438** | (0.00179,0.00386] | (0.00125,0.00292] | NA | (0.936,1.11] | (0.213,0.798] |
| **439** | (0.00386,0.00875] | (0.00292,0.00665] | NA | (0.731,0.936] | (0.213,0.798] |
| **440** | (0.00386,0.00875] | (0,0.00125] | NA | (1.11,Inf] | (0.798,1.72] |
| **441** | (0.00179,0.00386] | (0,0.00125] | NA | (0,0.731] | (0,0.213] |
| **442** | (0.00386,0.00875] | (0.00125,0.00292] | NA | (1.11,Inf] | (0.213,0.798] |
| **443** | (0.00386,0.00875] | (0.00125,0.00292] | NA | (1.11,Inf] | (0.213,0.798] |
| **444** | (0.00386,0.00875] | (0.00292,0.00665] | NA | (0.731,0.936] | (0.798,1.72] |
| **445** | (0.00386,0.00875] | (0.00125,0.00292] | NA | (0.731,0.936] | (0.213,0.798] |
| **446** | (0.00179,0.00386] | (0.00292,0.00665] | (0,0.46] | (0.936,1.11] | (0.798,1.72] |
| **447** | (0.00179,0.00386] | (0.00125,0.00292] | NA | (0.731,0.936] | (0.213,0.798] |
| **448** | (0.00386,0.00875] | (0.00292,0.00665] | NA | (1.11,Inf] | (0.213,0.798] |
| **449** | (0.00386,0.00875] | (0.00125,0.00292] | NA | (0.936,1.11] | (0.213,0.798] |
| **450** | (0.00386,0.00875] | (0.00125,0.00292] | NA | (0.936,1.11] | (0.213,0.798] |
| **451** | (0,0.00179] | (0,0.00125] | (0.46,Inf] | (0.731,0.936] | (0.213,0.798] |
| **452** | (0,0.00179] | (0,0.00125] | (0.46,Inf] | (0.731,0.936] | (0.213,0.798] |

| | | | | | |
|---|---|---|---|---|---|
| **453** | (0.00179,0.00386] | (0.00292,0.00665] | NA | (0.731,0.936] | (0.798,1.72] |
| **454** | (0,0.00179] | (0.00125,0.00292] | NA | (0.936,1.11] | (0.798,1.72] |
| **455** | (0.00179,0.00386] | (0.00125,0.00292] | NA | (0.731,0.936] | (0.213,0.798] |
| **456** | (0.00179,0.00386] | (0.00125,0.00292] | NA | (0.731,0.936] | (0.213,0.798] |
| **457** | (0.00179,0.00386] | (0.00125,0.00292] | NA | (0.731,0.936] | (0.213,0.798] |
| **458** | (0,0.00179] | (0.00125,0.00292] | NA | (0.936,1.11] | (1.72,Inf] |
| **459** | (0.00179,0.00386] | (0.00125,0.00292] | NA | (0.731,0.936] | (0.213,0.798] |
| **460** | (0,0.00179] | NA | (0.46,Inf] | (0.731,0.936] | NA |
| **461** | (0,0.00179] | (0,0.00125] | NA | (0.936,1.11] | (0.213,0.798] |
| **462** | (0.00179,0.00386] | (0,0.00125] | NA | (0.731,0.936] | (0.213,0.798] |
| **463** | (0.00386,0.00875] | (0.00125,0.00292] | NA | (1.11,Inf] | (0.213,0.798] |
| **464** | (0,0.00179] | (0.00125,0.00292] | (0.46,Inf] | (0,0.731] | (0.213,0.798] |
| **465** | (0,0.00179] | (0.00125,0.00292] | (0.46,Inf] | (0,0.731] | (0.213,0.798] |
| **466** | (0.00386,0.00875] | (0.00125,0.00292] | NA | (0.936,1.11] | (0.213,0.798] |
| **467** | (0.00179,0.00386] | (0.00292,0.00665] | NA | (0,0.731] | (0.798,1.72] |
| **468** | (0,0.00179] | (0.00125,0.00292] | (0.46,Inf] | (0,0.731] | (0.798,1.72] |
| **469** | (0.00179,0.00386] | (0.00292,0.00665] | NA | (0.936,1.11] | (1.72,Inf] |
| **470** | (0.00386,0.00875] | (0.00665,Inf] | NA | (0,0.731] | (0.798,1.72] |
| **471** | (0,0.00179] | (0.00125,0.00292] | NA | (0,0.731] | (0.798,1.72] |
| **472** | (0,0.00179] | (0,0.00125] | NA | (0.731,0.936] | (0.213,0.798] |
| **473** | (0.00179,0.00386] | (0.00125,0.00292] | NA | (0.731,0.936] | (0.798,1.72] |
| **474** | (0.00179,0.00386] | (0.00292,0.00665] | NA | (0.731,0.936] | (0.798,1.72] |
| **475** | (0.00386,0.00875] | (0.00292,0.00665] | NA | (0.936,1.11] | (0.798,1.72] |
| **476** | (0.00386,0.00875] | (0.00292,0.00665] | NA | (1.11,Inf] | (0.213,0.798] |
| **477** | (0.00179,0.00386] | (0.00125,0.00292] | NA | (0.936,1.11] | (0.213,0.798] |
| **478** | (0.00179,0.00386] | (0.00125,0.00292] | NA | (0.936,1.11] | (0.213,0.798] |
| **479** | (0.00179,0.00386] | (0,0.00125] | NA | (0.731,0.936] | (0,0.213] |
| **480** | (0.00386,0.00875] | (0.00125,0.00292] | NA | (0.731,0.936] | (0.213,0.798] |
| **481** | (0.00386,0.00875] | (0.00125,0.00292] | NA | (0.936,1.11] | (0.213,0.798] |
| **482** | (0.00386,0.00875] | (0.00292,0.00665] | NA | (0.936,1.11] | (0.798,1.72] |
| **483** | (0.00386,0.00875] | (0.00125,0.00292] | NA | (1.11,Inf] | (0.213,0.798] |
| **484** | (0.00386,0.00875] | (0.00665,Inf] | (0,0.46] | (1.11,Inf] | (1.72,Inf] |
| **485** | (0,0.00179] | (0,0.00125] | NA | (0.731,0.936] | (0.213,0.798] |
| **486** | (0.00386,0.00875] | (0.00665,Inf] | NA | (0.936,1.11] | (0.798,1.72] |
| **487** | (0.00179,0.00386] | (0,0.00125] | NA | (0.936,1.11] | (0.213,0.798] |
| **488** | (0.00386,0.00875] | (0.00292,0.00665] | NA | (0.731,0.936] | (0.798,1.72] |
| **489** | (0.00386,0.00875] | (0.00292,0.00665] | (0.46,Inf] | (0.936,1.11] | (0.213,0.798] |
| **490** | (0.00179,0.00386] | (0.00125,0.00292] | NA | (0.731,0.936] | (0.213,0.798] |

| | | | | | |
|---|---|---|---|---|---|
| **491** | (0.00179,0.00386] | (0.00292,0.00665] | NA | (0.936,1.11] | (0.798,1.72] |
| **492** | (0.00386,0.00875] | (0.00292,0.00665] | NA | (0.936,1.11] | (0.213,0.798] |
| **493** | (0,0.00179] | (0,0.00125] | NA | (0.936,1.11] | (0.798,1.72] |
| **494** | (0.00386,0.00875] | (0.00292,0.00665] | (0.46,Inf] | (0.936,1.11] | (0.798,1.72] |
| **495** | (0.00179,0.00386] | (0.00125,0.00292] | (0.46,Inf] | (0.936,1.11] | (0.213,0.798] |
| **496** | (0,0.00179] | (0.00125,0.00292] | NA | (0.731,0.936] | (0.798,1.72] |
| **497** | (0.00386,0.00875] | (0.00292,0.00665] | NA | (0.731,0.936] | (0.213,0.798] |
| **498** | (0.00179,0.00386] | (0.00292,0.00665] | NA | (0.731,0.936] | (0.798,1.72] |
| **499** | (0.00875,Inf] | (0.00292,0.00665] | NA | (1.11,Inf] | (0.213,0.798] |
| **500** | (0.00179,0.00386] | (0.00125,0.00292] | NA | (1.11,Inf] | (0.213,0.798] |
| **501** | (0.00179,0.00386] | (0.00125,0.00292] | NA | (0.936,1.11] | (0.798,1.72] |
| **502** | (0.00179,0.00386] | (0.00125,0.00292] | NA | (0.936,1.11] | (0.213,0.798] |
| **503** | (0.00179,0.00386] | (0.00292,0.00665] | NA | (0.731,0.936] | (0.213,0.798] |
| **504** | (0.00179,0.00386] | (0.00292,0.00665] | NA | (0,0.731] | (0.213,0.798] |
| **505** | (0.00386,0.00875] | (0.00292,0.00665] | NA | (0.936,1.11] | (0.798,1.72] |
| **506** | (0.00179,0.00386] | (0.00125,0.00292] | NA | (0.731,0.936] | (0.213,0.798] |
| **507** | (0.00875,Inf] | (0.00665,Inf] | NA | (0,0.731] | (0.213,0.798] |
| **508** | (0.00179,0.00386] | (0.00292,0.00665] | NA | (0.731,0.936] | (0.798,1.72] |
| **509** | (0.00386,0.00875] | (0.00665,Inf] | NA | (1.11,Inf] | (1.72,Inf] |
| **510** | (0.00179,0.00386] | (0.00125,0.00292] | NA | (0.731,0.936] | (0.213,0.798] |
| **511** | (0,0.00179] | (0.00125,0.00292] | NA | (0,0.731] | (0.798,1.72] |
| **512** | (0.00179,0.00386] | (0.00292,0.00665] | NA | (1.11,Inf] | (1.72,Inf] |
| **513** | (0.00179,0.00386] | (0.00292,0.00665] | NA | (0.731,0.936] | (0.798,1.72] |
| **514** | (0.00875,Inf] | (0.00665,Inf] | NA | (1.11,Inf] | (0.798,1.72] |
| **515** | (0,0.00179] | (0.00125,0.00292] | NA | (0.936,1.11] | (0.798,1.72] |
| **516** | (0.00386,0.00875] | (0.00125,0.00292] | NA | (0.936,1.11] | (0.213,0.798] |
| **517** | (0.00875,Inf] | (0.00665,Inf] | NA | (0.936,1.11] | (0.798,1.72] |
| **518** | (0.00179,0.00386] | (0.00292,0.00665] | NA | (0.936,1.11] | (0.798,1.72] |
| **519** | (0,0.00179] | NA | NA | (1.11,Inf] | NA |
| **520** | (0.00179,0.00386] | (0,0.00125] | NA | (1.11,Inf] | (0.213,0.798] |
| **521** | (0.00179,0.00386] | (0.00292,0.00665] | NA | (0.731,0.936] | (0.798,1.72] |
| **522** | (0.00386,0.00875] | (0.00125,0.00292] | NA | (0.936,1.11] | (0.213,0.798] |
| **523** | (0.00179,0.00386] | (0.00125,0.00292] | (0,0.46] | (0.731,0.936] | (0.213,0.798] |
| **524** | (0,0.00179] | (0.00125,0.00292] | NA | (0.936,1.11] | (1.72,Inf] |
| **525** | (0.00179,0.00386] | (0.00125,0.00292] | NA | (0.731,0.936] | (0.798,1.72] |
| **526** | (0.00386,0.00875] | (0.00665,Inf] | (0,0.46] | (0.936,1.11] | (0.798,1.72] |
| **527** | (0.00179,0.00386] | (0.00665,Inf] | NA | (0.731,0.936] | (1.72,Inf] |
| **528** | (0.00179,0.00386] | (0.00125,0.00292] | NA | (0.731,0.936] | (0.213,0.798] |

| | | | | | |
|---|---|---|---|---|---|
| **529** | (0.00875,Inf] | (0.00665,Inf] | NA | (0.731,0.936] | (0.798,1.72] |
| **530** | (0.00386,0.00875] | (0.00292,0.00665] | NA | (0.731,0.936] | (0.213,0.798] |
| **531** | (0.00386,0.00875] | (0.00292,0.00665] | NA | (1.11,Inf] | (0.798,1.72] |
| **532** | (0,0.00179] | (0.00125,0.00292] | NA | (0.731,0.936] | (0.213,0.798] |
| **533** | (0.00386,0.00875] | (0.00292,0.00665] | NA | (0.936,1.11] | (0.213,0.798] |
| **534** | (0.00179,0.00386] | (0.00125,0.00292] | NA | (0.936,1.11] | (0.798,1.72] |
| **535** | (0.00179,0.00386] | (0.00125,0.00292] | (0.46,Inf] | (1.11,Inf] | (0.798,1.72] |
| **536** | (0.00386,0.00875] | (0.00125,0.00292] | NA | (0.936,1.11] | (0.213,0.798] |
| **537** | (0,0.00179] | (0,0.00125] | NA | (0.936,1.11] | (0.213,0.798] |
| **538** | (0.00179,0.00386] | (0,0.00125] | NA | (0.936,1.11] | (0,0.213] |
| **539** | (0.00179,0.00386] | (0.00125,0.00292] | NA | (0.731,0.936] | (0.213,0.798] |
| **540** | (0.00179,0.00386] | (0.00125,0.00292] | NA | (0.936,1.11] | (0.798,1.72] |
| **541** | NA | (0,0.00125] | NA | (0,0.731] | (1.72,Inf] |
| **542** | (0.00179,0.00386] | (0.00125,0.00292] | NA | (0.731,0.936] | (0.798,1.72] |
| **543** | (0,0.00179] | (0.00292,0.00665] | NA | (0.936,1.11] | (1.72,Inf] |
| **544** | (0.00386,0.00875] | (0.00125,0.00292] | NA | (0.936,1.11] | (0.213,0.798] |
| **545** | NA | NA | NA | (1.11,Inf] | NA |
| **546** | (0.00179,0.00386] | (0,0.00125] | NA | (1.11,Inf] | (0.213,0.798] |
| **547** | (0.00386,0.00875] | (0.00292,0.00665] | NA | (0.731,0.936] | (0.213,0.798] |
| **548** | (0.00386,0.00875] | (0.00292,0.00665] | NA | (0.936,1.11] | (0.213,0.798] |
| **549** | (0.00386,0.00875] | (0.00665,Inf] | NA | (0,0.731] | (0.798,1.72] |
| **550** | (0.00179,0.00386] | (0.00125,0.00292] | NA | (1.11,Inf] | (0.798,1.72] |
| **551** | (0.00179,0.00386] | (0.00292,0.00665] | NA | (0.731,0.936] | (0.798,1.72] |
| **552** | (0.00179,0.00386] | (0,0.00125] | NA | (1.11,Inf] | (0.213,0.798] |
| **553** | (0,0.00179] | (0.00125,0.00292] | NA | (0.936,1.11] | (1.72,Inf] |
| **554** | (0.00179,0.00386] | (0,0.00125] | NA | (0.731,0.936] | (0.213,0.798] |
| **555** | (0,0.00179] | (0.00125,0.00292] | NA | (0,0.731] | (0.798,1.72] |
| **556** | (0.00179,0.00386] | NA | NA | (0,0.731] | NA |
| **557** | (0.00179,0.00386] | (0,0.00125] | NA | (1.11,Inf] | (0.213,0.798] |
| **558** | (0.00386,0.00875] | (0.00125,0.00292] | NA | (0.936,1.11] | (0.213,0.798] |
| **559** | (0,0.00179] | (0.00292,0.00665] | NA | (0.731,0.936] | (1.72,Inf] |
| **560** | (0.00386,0.00875] | (0.00125,0.00292] | NA | (0.731,0.936] | (0.213,0.798] |
| **561** | (0.00386,0.00875] | (0.00125,0.00292] | NA | (0.936,1.11] | (0.213,0.798] |
| **562** | (0.00386,0.00875] | (0,0.00125] | NA | (1.11,Inf] | (0,0.213] |
| **563** | (0,0.00179] | (0,0.00125] | NA | (1.11,Inf] | (0.798,1.72] |
| **564** | (0.00179,0.00386] | (0,0.00125] | NA | (0.731,0.936] | (0.213,0.798] |
| **565** | (0.00179,0.00386] | (0.00292,0.00665] | NA | (0.936,1.11] | (1.72,Inf] |
| **566** | (0.00179,0.00386] | (0,0.00125] | NA | (1.11,Inf] | (0.213,0.798] |

| | | | | | |
|---|---|---|---|---|---|
| **567** | (0.00875,Inf] | (0.00665,Inf] | NA | (1.11,Inf] | (0.798,1.72] |
| **568** | (0.00875,Inf] | (0.00292,0.00665] | NA | (0.936,1.11] | (0.213,0.798] |
| **569** | (0.00179,0.00386] | (0.00125,0.00292] | NA | (0.731,0.936] | (0.798,1.72] |
| **570** | (0.00386,0.00875] | (0.00292,0.00665] | NA | (0.731,0.936] | (0.213,0.798] |
| **571** | (0,0.00179] | (0.00125,0.00292] | NA | (0.731,0.936] | (0.798,1.72] |
| **572** | (0.00875,Inf] | (0.00665,Inf] | NA | (0.936,1.11] | (0.213,0.798] |
| **573** | (0.00386,0.00875] | (0.00292,0.00665] | NA | (1.11,Inf] | (0.798,1.72] |
| **574** | (0.00386,0.00875] | (0.00125,0.00292] | NA | (1.11,Inf] | (0.213,0.798] |
| **575** | (0.00386,0.00875] | (0.00125,0.00292] | NA | (1.11,Inf] | (0.213,0.798] |
| **576** | (0.00179,0.00386] | (0.00292,0.00665] | NA | (1.11,Inf] | (1.72,Inf] |
| **577** | (0.00179,0.00386] | (0.00125,0.00292] | NA | (0.936,1.11] | (0.798,1.72] |
| **578** | (0.00386,0.00875] | (0.00292,0.00665] | NA | (0.936,1.11] | (0.798,1.72] |
| **579** | (0.00179,0.00386] | (0.00125,0.00292] | NA | (0.936,1.11] | (0.213,0.798] |
| **580** | (0.00386,0.00875] | (0.00292,0.00665] | NA | (0.936,1.11] | (0.798,1.72] |
| **581** | (0.00179,0.00386] | (0.00292,0.00665] | NA | (0.936,1.11] | (0.798,1.72] |
| **582** | (0.00179,0.00386] | (0.00125,0.00292] | NA | (1.11,Inf] | (0.798,1.72] |
| **583** | (0.00875,Inf] | (0.00665,Inf] | (0,0.46] | (1.11,Inf] | (0.798,1.72] |
| **584** | (0.00179,0.00386] | (0.00292,0.00665] | NA | (0,0.731] | (0.213,0.798] |
| **585** | (0.00179,0.00386] | (0.00292,0.00665] | NA | (0,0.731] | (0.213,0.798] |
| **586** | (0.00179,0.00386] | (0.00125,0.00292] | NA | (0.936,1.11] | (0.213,0.798] |
| **587** | (0,0.00179] | (0.00125,0.00292] | NA | (0.731,0.936] | (0.798,1.72] |
| **588** | (0,0.00179] | (0,0.00125] | NA | (0.731,0.936] | (0.798,1.72] |
| **589** | (0.00179,0.00386] | (0.00125,0.00292] | NA | (0.936,1.11] | (0.798,1.72] |
| **590** | (0,0.00179] | (0,0.00125] | NA | (0.731,0.936] | (0.213,0.798] |
| **591** | (0,0.00179] | (0.00125,0.00292] | NA | (0.731,0.936] | (1.72,Inf] |
| **592** | (0.00179,0.00386] | (0.00292,0.00665] | NA | (0.936,1.11] | (0.798,1.72] |
| **593** | (0,0.00179] | (0.00292,0.00665] | NA | (0.731,0.936] | (1.72,Inf] |
| **594** | (0.00179,0.00386] | (0.00125,0.00292] | NA | (0.731,0.936] | (0.213,0.798] |
| **595** | (0.00386,0.00875] | (0.00125,0.00292] | NA | (1.11,Inf] | (0.213,0.798] |
| **596** | (0.00386,0.00875] | NA | NA | (1.11,Inf] | NA |
| **597** | (0.00179,0.00386] | (0,0.00125] | NA | (0.936,1.11] | (0.213,0.798] |
| **598** | (0.00386,0.00875] | (0.00292,0.00665] | (0.46,Inf] | (1.11,Inf] | (0.213,0.798] |
| **599** | (0.00179,0.00386] | (0.00292,0.00665] | NA | (0.731,0.936] | (1.72,Inf] |
| **600** | (0.00179,0.00386] | (0,0.00125] | NA | (0.731,0.936] | (0,0.213] |
| **601** | (0.00386,0.00875] | (0.00292,0.00665] | NA | (0,0.731] | (0.213,0.798] |
| **602** | (0.00875,Inf] | (0.00125,0.00292] | NA | (1.11,Inf] | (0.213,0.798] |
| **603** | (0.00179,0.00386] | (0.00665,Inf] | NA | (0.731,0.936] | (0.798,1.72] |
| **604** | (0.00179,0.00386] | (0.00292,0.00665] | NA | (1.11,Inf] | (1.72,Inf] |

| | | | | | |
|---|---|---|---|---|---|
| **605** | (0.00179,0.00386] | (0,0.00125] | (0.46,Inf] | (0.731,0.936] | (0,0.213] |
| **606** | (0.00179,0.00386] | (0.00292,0.00665] | NA | (0,0.731] | (1.72,Inf] |
| **607** | (0.00875,Inf] | (0.00665,Inf] | NA | (0.731,0.936] | (0.213,0.798] |
| **608** | (0.00386,0.00875] | (0.00125,0.00292] | NA | (0.731,0.936] | (0.213,0.798] |
| **609** | (0.00386,0.00875] | (0.00125,0.00292] | NA | (0.936,1.11] | (0.213,0.798] |
| **610** | (0,0.00179] | (0.00125,0.00292] | NA | (0.936,1.11] | (0.798,1.72] |
| **611** | (0.00179,0.00386] | (0.00125,0.00292] | NA | (1.11,Inf] | (0.798,1.72] |
| **612** | NA | NA | NA | (0.936,1.11] | NA |
| **613** | (0.00179,0.00386] | (0.00292,0.00665] | NA | (0.936,1.11] | (0.798,1.72] |
| **614** | (0.00179,0.00386] | (0.00125,0.00292] | NA | (0,0.731] | (0.213,0.798] |
| **615** | (0.00179,0.00386] | (0.00125,0.00292] | NA | (0,0.731] | (0.213,0.798] |
| **616** | (0.00179,0.00386] | (0.00292,0.00665] | NA | (1.11,Inf] | (0.798,1.72] |
| **617** | (0.00179,0.00386] | (0.00125,0.00292] | NA | (0.731,0.936] | (0.213,0.798] |
| **618** | (0.00179,0.00386] | (0.00665,Inf] | (0,0.46] | (1.11,Inf] | (1.72,Inf] |
| **619** | (0.00386,0.00875] | (0.00292,0.00665] | NA | (0.936,1.11] | (0.213,0.798] |
| **620** | (0.00179,0.00386] | (0.00292,0.00665] | NA | (0.731,0.936] | (0.213,0.798] |
| **621** | (0.00179,0.00386] | (0,0.00125] | NA | (0.731,0.936] | (0.213,0.798] |
| **622** | (0,0.00179] | (0.00125,0.00292] | NA | (0,0.731] | (0.798,1.72] |
| **623** | (0.00386,0.00875] | (0.00292,0.00665] | NA | (0.936,1.11] | (0.213,0.798] |
| **624** | (0,0.00179] | (0.00125,0.00292] | NA | (1.11,Inf] | (1.72,Inf] |
| **625** | (0,0.00179] | (0,0.00125] | NA | (0.731,0.936] | (0.213,0.798] |
| **626** | (0.00179,0.00386] | (0.00292,0.00665] | NA | (0.936,1.11] | (1.72,Inf] |
| **627** | (0.00875,Inf] | (0.00292,0.00665] | NA | (1.11,Inf] | (0.798,1.72] |
| **628** | (0.00875,Inf] | (0,0.00125] | NA | (1.11,Inf] | (0,0.213] |
| **629** | (0.00386,0.00875] | (0.00665,Inf] | (0.46,Inf] | (0.731,0.936] | (0.798,1.72] |
| **630** | (0.00179,0.00386] | (0.00665,Inf] | NA | (0.731,0.936] | (1.72,Inf] |
| **631** | (0.00179,0.00386] | (0.00125,0.00292] | NA | (0.731,0.936] | (0.213,0.798] |
| **632** | (0.00179,0.00386] | (0.00292,0.00665] | NA | (0.936,1.11] | (0.798,1.72] |
| **633** | (0,0.00179] | (0,0.00125] | NA | (0.731,0.936] | (0.213,0.798] |
| **634** | (0.00179,0.00386] | (0.00125,0.00292] | NA | (0,0.731] | (0.798,1.72] |
| **635** | (0.00179,0.00386] | (0.00125,0.00292] | NA | (0.936,1.11] | (0.798,1.72] |
| **636** | (0.00179,0.00386] | (0.00125,0.00292] | NA | (0,0.731] | (0.798,1.72] |
| **637** | (0,0.00179] | (0.00125,0.00292] | NA | (1.11,Inf] | (1.72,Inf] |
| **638** | (0,0.00179] | (0.00125,0.00292] | NA | (0.731,0.936] | (1.72,Inf] |
| **639** | (0.00386,0.00875] | (0.00292,0.00665] | NA | (0.731,0.936] | (0.798,1.72] |
| **640** | (0,0.00179] | (0.00125,0.00292] | NA | (0.731,0.936] | (0.798,1.72] |
| **641** | (0.00386,0.00875] | (0.00125,0.00292] | NA | (1.11,Inf] | (0.213,0.798] |
| **642** | (0.00386,0.00875] | (0.00125,0.00292] | NA | (0.731,0.936] | (0.213,0.798] |

| | | | | | |
|---|---|---|---|---|---|
| **643** | (0.00386,0.00875] | (0.00292,0.00665] | NA | (0.731,0.936] | (0.798,1.72] |
| **644** | (0.00386,0.00875] | (0.00292,0.00665] | NA | (0,0.731] | (0.213,0.798] |
| **645** | (0.00179,0.00386] | (0.00125,0.00292] | NA | (0,0.731] | (0.213,0.798] |
| **646** | (0.00386,0.00875] | (0.00665,Inf] | NA | (0.731,0.936] | (0.798,1.72] |
| **647** | (0.00179,0.00386] | (0.00125,0.00292] | NA | (0.731,0.936] | (0.213,0.798] |
| **648** | (0,0.00179] | (0.00292,0.00665] | NA | (0.731,0.936] | (0.798,1.72] |
| **649** | (0.00179,0.00386] | (0.00292,0.00665] | NA | (0.936,1.11] | (0.798,1.72] |
| **650** | NA | NA | NA | (1.11,Inf] | NA |
| **651** | (0.00386,0.00875] | (0.00292,0.00665] | NA | (0.936,1.11] | (0.798,1.72] |
| **652** | (0.00179,0.00386] | (0.00125,0.00292] | NA | (0.936,1.11] | (0.213,0.798] |
| **653** | (0.00386,0.00875] | (0.00665,Inf] | NA | (0.936,1.11] | (0.798,1.72] |
| **654** | (0.00179,0.00386] | (0.00125,0.00292] | NA | (0.936,1.11] | (0.798,1.72] |
| **655** | (0.00875,Inf] | (0.00665,Inf] | NA | (0.936,1.11] | (1.72,Inf] |
| **656** | (0.00386,0.00875] | (0,0.00125] | NA | (0.731,0.936] | (0,0.213] |
| **657** | (0.00386,0.00875] | (0.00125,0.00292] | NA | (0.936,1.11] | (0.213,0.798] |
| **658** | (0.00386,0.00875] | (0.00292,0.00665] | (0.46,Inf] | (0.936,1.11] | (0.213,0.798] |
| **659** | (0.00386,0.00875] | (0.00125,0.00292] | NA | (0.731,0.936] | (0.213,0.798] |
| **660** | (0.00179,0.00386] | (0.00292,0.00665] | NA | (0.936,1.11] | (0.798,1.72] |
| **661** | NA | NA | NA | (0,0.731] | NA |
| **662** | (0.00386,0.00875] | (0.00125,0.00292] | NA | (0.936,1.11] | (0.213,0.798] |
| **663** | (0.00179,0.00386] | (0.00292,0.00665] | NA | (0.936,1.11] | (0.798,1.72] |
| **664** | (0,0.00179] | (0,0.00125] | NA | (0,0.731] | (0.213,0.798] |
| **665** | NA | (0.00292,0.00665] | NA | (1.11,Inf] | (1.72,Inf] |
| **666** | (0.00386,0.00875] | (0.00292,0.00665] | NA | (0.936,1.11] | (0.213,0.798] |
| **667** | (0.00386,0.00875] | (0.00292,0.00665] | NA | (0.936,1.11] | (0.798,1.72] |
| **668** | (0.00386,0.00875] | (0.00292,0.00665] | NA | (0.731,0.936] | (0.798,1.72] |
| **669** | (0.00179,0.00386] | (0,0.00125] | NA | (1.11,Inf] | (0.213,0.798] |
| **670** | (0,0.00179] | NA | NA | (0.731,0.936] | NA |
| **671** | (0,0.00179] | (0.00125,0.00292] | NA | (0.731,0.936] | (0.798,1.72] |
| **672** | NA | NA | NA | (0,0.731] | NA |
| **673** | (0.00875,Inf] | (0.00665,Inf] | (0.46,Inf] | (0.731,0.936] | (0.213,0.798] |
| **674** | (0.00386,0.00875] | (0.00292,0.00665] | NA | (0.731,0.936] | (0.213,0.798] |
| **675** | (0.00179,0.00386] | (0.00125,0.00292] | NA | (0,0.731] | (0.213,0.798] |
| **676** | (0.00179,0.00386] | (0.00125,0.00292] | NA | (0.936,1.11] | (0.798,1.72] |
| **677** | (0.00179,0.00386] | (0,0.00125] | NA | (0.731,0.936] | (0.213,0.798] |
| **678** | NA | NA | NA | (0.936,1.11] | NA |
| **679** | (0,0.00179] | (0.00292,0.00665] | NA | (0,0.731] | (1.72,Inf] |
| **680** | (0.00386,0.00875] | (0.00125,0.00292] | NA | (0,0.731] | (0.213,0.798] |

| | | | | | |
|---|---|---|---|---|---|
| **681** | (0.00179,0.00386] | NA | NA | (0.936,1.11] | NA |
| **682** | (0,0.00179] | (0,0.00125] | NA | (0.936,1.11] | (0.213,0.798] |
| **683** | (0.00179,0.00386] | (0.00292,0.00665] | NA | (0.731,0.936] | (1.72,Inf] |
| **684** | (0.00386,0.00875] | (0.00292,0.00665] | NA | (0.731,0.936] | (0.213,0.798] |
| **685** | (0.00179,0.00386] | (0.00125,0.00292] | NA | (0.731,0.936] | (0.798,1.72] |
| **686** | (0.00386,0.00875] | (0.00125,0.00292] | NA | (0.936,1.11] | (0.213,0.798] |
| **687** | (0.00875,Inf] | (0.00665,Inf] | NA | (0.936,1.11] | (0.213,0.798] |
| **688** | (0.00386,0.00875] | (0.00665,Inf] | NA | (0,0.731] | (1.72,Inf] |
| **689** | (0.00179,0.00386] | (0,0.00125] | NA | (1.11,Inf] | (0,0.213] |
| **690** | (0.00386,0.00875] | (0.00125,0.00292] | NA | (0.731,0.936] | (0.213,0.798] |
| **691** | (0.00179,0.00386] | (0.00292,0.00665] | NA | (1.11,Inf] | (1.72,Inf] |
| **692** | (0,0.00179] | (0.00292,0.00665] | (0.46,Inf] | (0.731,0.936] | (1.72,Inf] |
| **693** | (0.00875,Inf] | (0.00292,0.00665] | NA | (1.11,Inf] | (0.213,0.798] |
| **694** | (0.00386,0.00875] | (0.00292,0.00665] | NA | (1.11,Inf] | (0.798,1.72] |
| **695** | (0.00386,0.00875] | (0.00292,0.00665] | NA | (1.11,Inf] | (0.798,1.72] |
| **696** | (0,0.00179] | (0.00292,0.00665] | NA | (0,0.731] | (0.798,1.72] |
| **697** | (0.00386,0.00875] | NA | NA | (0.731,0.936] | NA |
| **698** | (0,0.00179] | (0.00665,Inf] | NA | (1.11,Inf] | (1.72,Inf] |
| **699** | (0.00386,0.00875] | (0.00292,0.00665] | NA | (0.936,1.11] | (0.798,1.72] |
| **700** | (0,0.00179] | (0.00125,0.00292] | NA | (1.11,Inf] | (0.798,1.72] |
| **701** | (0,0.00179] | (0.00125,0.00292] | NA | (0.936,1.11] | (1.72,Inf] |
| **702** | (0.00386,0.00875] | (0,0.00125] | NA | (1.11,Inf] | (0,0.213] |
| **703** | (0,0.00179] | (0,0.00125] | NA | (0.731,0.936] | (0.213,0.798] |
| **704** | (0.00875,Inf] | (0.00292,0.00665] | NA | (1.11,Inf] | (0.213,0.798] |
| **705** | (0.00179,0.00386] | (0,0.00125] | NA | (0.731,0.936] | (0.213,0.798] |
| **706** | (0.00386,0.00875] | (0.00292,0.00665] | NA | (0.731,0.936] | (0.213,0.798] |
| **707** | (0.00386,0.00875] | (0.00292,0.00665] | NA | (0.936,1.11] | (0.798,1.72] |
| **708** | (0.00179,0.00386] | (0.00125,0.00292] | NA | (0,0.731] | (0.213,0.798] |
| **709** | (0.00386,0.00875] | (0.00292,0.00665] | (0,0.46] | (1.11,Inf] | (0.213,0.798] |
| **710** | (0,0.00179] | (0.00125,0.00292] | NA | (0.731,0.936] | (1.72,Inf] |
| **711** | (0.00386,0.00875] | (0.00292,0.00665] | NA | (0.936,1.11] | (0.213,0.798] |
| **712** | (0,0.00179] | NA | NA | (0,0.731] | NA |
| **713** | (0.00386,0.00875] | (0.00292,0.00665] | NA | (0.731,0.936] | (0.798,1.72] |
| **714** | (0.00875,Inf] | (0.00665,Inf] | NA | (0.936,1.11] | (0.213,0.798] |
| **715** | (0.00386,0.00875] | (0.00125,0.00292] | NA | (0.936,1.11] | (0.213,0.798] |
| **716** | (0.00386,0.00875] | (0.00292,0.00665] | NA | (0.731,0.936] | (0.798,1.72] |
| **717** | (0.00386,0.00875] | (0.00665,Inf] | (0,0.46] | (0.936,1.11] | (1.72,Inf] |
| **718** | (0.00386,0.00875] | (0.00665,Inf] | (0,0.46] | (0.936,1.11] | (1.72,Inf] |

| | | | | | |
|---|---|---|---|---|---|
| **719** | (0.00179,0.00386] | (0.00125,0.00292] | (0.46,Inf] | (0.731,0.936] | (0.213,0.798] |
| **720** | (0.00179,0.00386] | (0.00125,0.00292] | (0.46,Inf] | (0.731,0.936] | (0.213,0.798] |
| **721** | (0.00386,0.00875] | (0.00292,0.00665] | NA | (0.731,0.936] | (0.213,0.798] |
| **722** | (0.00386,0.00875] | (0.00125,0.00292] | NA | (0.936,1.11] | (0.213,0.798] |
| **723** | (0.00386,0.00875] | (0.00125,0.00292] | NA | (1.11,Inf] | (0.213,0.798] |
| **724** | (0,0.00179] | (0,0.00125] | NA | (1.11,Inf] | (0.798,1.72] |
| **725** | (0.00386,0.00875] | (0.00665,Inf] | NA | (1.11,Inf] | (1.72,Inf] |
| **726** | (0.00386,0.00875] | (0.00125,0.00292] | NA | (0.936,1.11] | (0.213,0.798] |
| **727** | (0.00386,0.00875] | (0.00292,0.00665] | NA | (0.936,1.11] | (0.798,1.72] |
| **728** | (0.00179,0.00386] | (0.00125,0.00292] | NA | (0.936,1.11] | (0.213,0.798] |
| **729** | (0.00179,0.00386] | (0.00125,0.00292] | NA | (0.731,0.936] | (0.213,0.798] |
| **730** | (0.00386,0.00875] | (0.00125,0.00292] | NA | (1.11,Inf] | (0.798,1.72] |
| **731** | (0.00875,Inf] | (0.00292,0.00665] | NA | (1.11,Inf] | (0.213,0.798] |
| **732** | (0.00179,0.00386] | (0.00125,0.00292] | NA | (0,0.731] | (0.213,0.798] |
| **733** | NA | NA | NA | (0,0.731] | NA |
| **734** | (0,0.00179] | (0.00292,0.00665] | NA | (0,0.731] | (1.72,Inf] |
| **735** | (0.00179,0.00386] | (0.00292,0.00665] | (0.46,Inf] | (0.731,0.936] | (0.798,1.72] |
| **736** | (0.00386,0.00875] | (0.00665,Inf] | NA | (1.11,Inf] | (0.798,1.72] |
| **737** | (0.00179,0.00386] | (0,0.00125] | NA | (1.11,Inf] | (0.213,0.798] |
| **738** | (0.00179,0.00386] | (0.00292,0.00665] | NA | (1.11,Inf] | (0.798,1.72] |
| **739** | (0.00386,0.00875] | (0.00125,0.00292] | NA | (0.936,1.11] | (0.213,0.798] |
| **740** | (0.00875,Inf] | (0.00125,0.00292] | NA | (1.11,Inf] | (0.213,0.798] |
| **741** | (0.00386,0.00875] | (0,0.00125] | (0.46,Inf] | (1.11,Inf] | (0,0.213] |
| **742** | (0.00179,0.00386] | (0.00292,0.00665] | NA | (1.11,Inf] | (1.72,Inf] |
| **743** | NA | NA | (0.46,Inf] | NA | (0.798,1.72] |
| **744** | (0.00875,Inf] | (0.00665,Inf] | (0.46,Inf] | (1.11,Inf] | (0.213,0.798] |
| **745** | (0.00179,0.00386] | NA | NA | (1.11,Inf] | NA |
| **746** | (0,0.00179] | (0.00292,0.00665] | NA | (0.731,0.936] | (1.72,Inf] |
| **747** | (0.00386,0.00875] | (0.00665,Inf] | NA | (1.11,Inf] | (1.72,Inf] |
| **748** | (0.00179,0.00386] | (0,0.00125] | NA | (1.11,Inf] | (0.213,0.798] |
| **749** | NA | (0,0.00125] | NA | (0,0.731] | (1.72,Inf] |
| **750** | (0.00875,Inf] | (0.00665,Inf] | NA | (0.731,0.936] | (0.798,1.72] |
| **751** | (0.00179,0.00386] | (0.00292,0.00665] | NA | (0,0.731] | (0.798,1.72] |
| **752** | (0.00875,Inf] | (0.00292,0.00665] | NA | (0.936,1.11] | (0.213,0.798] |
| **753** | (0.00386,0.00875] | (0.00125,0.00292] | NA | (0.936,1.11] | (0.213,0.798] |
| **754** | (0.00386,0.00875] | (0.00292,0.00665] | NA | (0.731,0.936] | (0.213,0.798] |
| **755** | (0.00179,0.00386] | (0.00292,0.00665] | NA | (0.731,0.936] | (0.798,1.72] |
| **756** | (0.00386,0.00875] | (0.00125,0.00292] | (0.46,Inf] | (1.11,Inf] | (0.213,0.798] |

| | | | | | |
|---|---|---|---|---|---|
| **757** | (0.00179,0.00386] | (0.00125,0.00292] | NA | (1.11,Inf] | (0.798,1.72] |
| **758** | (0.00179,0.00386] | (0.00292,0.00665] | NA | (0.936,1.11] | (1.72,Inf] |
| **759** | (0.00179,0.00386] | (0,0.00125] | NA | (0.936,1.11] | (0.213,0.798] |
| **760** | NA | NA | NA | NA | (0.213,0.798] |
| **761** | (0.00179,0.00386] | (0.00665,Inf] | NA | (0,0.731] | (0.798,1.72] |
| **762** | (0.00179,0.00386] | (0.00665,Inf] | NA | (1.11,Inf] | (1.72,Inf] |
| **763** | (0.00179,0.00386] | (0.00292,0.00665] | NA | (0,0.731] | (0.798,1.72] |
| **764** | NA | (0,0.00125] | NA | (0.936,1.11] | (1.72,Inf] |
| **765** | NA | NA | NA | (0.936,1.11] | NA |
| **766** | (0.00179,0.00386] | NA | NA | (0.936,1.11] | NA |
| **767** | (0.00386,0.00875] | (0.00292,0.00665] | (0.46,Inf] | (1.11,Inf] | (0.213,0.798] |
| **768** | (0.00179,0.00386] | (0,0.00125] | NA | (0.936,1.11] | (0.213,0.798] |
| **769** | (0.00875,Inf] | (0,0.00125] | NA | (0.731,0.936] | (0,0.213] |
| **770** | (0.00386,0.00875] | NA | NA | (0.731,0.936] | NA |
| **771** | (0.00386,0.00875] | (0.00292,0.00665] | (0.46,Inf] | (0.731,0.936] | (0.213,0.798] |
| **772** | NA | NA | NA | (0.731,0.936] | NA |
| **773** | (0.00179,0.00386] | (0.00292,0.00665] | NA | (0.731,0.936] | (0.798,1.72] |
| **774** | NA | NA | NA | (1.11,Inf] | NA |
| **775** | (0.00875,Inf] | (0.00292,0.00665] | NA | (0.936,1.11] | (0.213,0.798] |
| **776** | (0,0.00179] | (0.00125,0.00292] | NA | (0.731,0.936] | (1.72,Inf] |
| **777** | (0.00179,0.00386] | (0.00665,Inf] | NA | (0.731,0.936] | (1.72,Inf] |
| **778** | (0.00386,0.00875] | (0.00665,Inf] | NA | (0.936,1.11] | (0.798,1.72] |
| **779** | (0.00179,0.00386] | (0.00292,0.00665] | NA | (0,0.731] | (0.213,0.798] |
| **780** | (0.00875,Inf] | (0.00292,0.00665] | NA | (0.731,0.936] | (0,0.213] |
| **781** | (0,0.00179] | (0,0.00125] | NA | (1.11,Inf] | (0.213,0.798] |
| **782** | (0.00875,Inf] | (0.00665,Inf] | NA | (1.11,Inf] | (0.798,1.72] |
| **783** | (0,0.00179] | (0.00125,0.00292] | (0.46,Inf] | (0.936,1.11] | (1.72,Inf] |
| **784** | (0.00179,0.00386] | (0.00125,0.00292] | NA | (0.936,1.11] | (0.213,0.798] |
| **785** | (0.00386,0.00875] | (0.00665,Inf] | NA | (0.731,0.936] | (0.798,1.72] |
| **786** | (0,0.00179] | (0.00125,0.00292] | NA | (0.936,1.11] | (1.72,Inf] |
| **787** | (0.00386,0.00875] | (0.00665,Inf] | NA | (0.731,0.936] | (0.213,0.798] |
| **788** | (0.00386,0.00875] | (0.00292,0.00665] | (0,0.46] | (0.936,1.11] | (0.798,1.72] |
| **789** | NA | NA | NA | (0,0.731] | NA |
| **790** | (0.00179,0.00386] | (0.00125,0.00292] | NA | (0.731,0.936] | (0.213,0.798] |
| **791** | (0.00179,0.00386] | (0.00292,0.00665] | NA | (0.936,1.11] | (0.798,1.72] |
| **792** | (0.00386,0.00875] | (0.00292,0.00665] | NA | (0.731,0.936] | (0.213,0.798] |
| **793** | (0.00386,0.00875] | (0.00292,0.00665] | NA | (0.731,0.936] | (0.213,0.798] |
| **794** | (0.00179,0.00386] | (0,0.00125] | NA | (0.731,0.936] | (0,0.213] |

| | | | | | |
|---|---|---|---|---|---|
| **795** | (0.00875,Inf] | (0.00292,0.00665] | NA | (1.11,Inf] | (0.213,0.798] |
| **796** | (0,0.00179] | (0.00292,0.00665] | NA | (1.11,Inf] | (1.72,Inf] |
| **797** | (0.00179,0.00386] | NA | NA | (0.731,0.936] | NA |
| **798** | NA | NA | NA | (0.936,1.11] | NA |
| **799** | (0.00875,Inf] | (0.00665,Inf] | NA | (1.11,Inf] | (0.798,1.72] |
| **800** | (0.00386,0.00875] | (0.00292,0.00665] | NA | (0.731,0.936] | (0.798,1.72] |
| **801** | NA | (0.00125,0.00292] | NA | (0,0.731] | (1.72,Inf] |
| **802** | NA | NA | NA | (1.11,Inf] | NA |
| **803** | (0.00179,0.00386] | (0.00292,0.00665] | NA | (0.936,1.11] | (0.798,1.72] |
| **804** | (0,0.00179] | NA | NA | (0.731,0.936] | NA |
| **805** | (0,0.00179] | (0.00665,Inf] | (0.46,Inf] | (0.731,0.936] | (1.72,Inf] |
| **806** | (0,0.00179] | (0.00125,0.00292] | NA | (0.731,0.936] | (0.213,0.798] |
| **807** | (0,0.00179] | (0.00125,0.00292] | NA | (0.936,1.11] | (1.72,Inf] |
| **808** | (0.00179,0.00386] | (0.00292,0.00665] | NA | (0.731,0.936] | (0.798,1.72] |
| **809** | (0.00179,0.00386] | NA | NA | (0.731,0.936] | NA |
| **810** | (0.00179,0.00386] | (0,0.00125] | (0.46,Inf] | (0,0.731] | (0,0.213] |
| **811** | (0.00386,0.00875] | (0.00665,Inf] | NA | (0.731,0.936] | (0.798,1.72] |
| **812** | (0.00386,0.00875] | (0.00125,0.00292] | NA | (0.731,0.936] | (0.213,0.798] |
| **813** | (0.00179,0.00386] | (0,0.00125] | NA | (1.11,Inf] | (0.213,0.798] |
| **814** | (0,0.00179] | (0.00125,0.00292] | NA | (0.731,0.936] | (0.798,1.72] |
| **815** | (0.00386,0.00875] | (0.00125,0.00292] | NA | (0,0.731] | (0.213,0.798] |
| **816** | (0.00179,0.00386] | (0.00292,0.00665] | NA | (0,0.731] | (0.213,0.798] |
| **817** | (0.00179,0.00386] | (0,0.00125] | NA | (1.11,Inf] | (0,0.213] |
| **818** | (0.00179,0.00386] | NA | NA | (0,0.731] | NA |
| **819** | NA | (0,0.00125] | NA | (0,0.731] | (1.72,Inf] |
| **820** | (0.00179,0.00386] | (0.00292,0.00665] | NA | (0,0.731] | (0.798,1.72] |
| **821** | (0.00875,Inf] | (0.00665,Inf] | NA | (0.731,0.936] | (0.798,1.72] |
| **822** | (0.00179,0.00386] | (0.00125,0.00292] | NA | (0.936,1.11] | (0.213,0.798] |
| **823** | (0.00179,0.00386] | (0.00292,0.00665] | NA | (0,0.731] | (0.798,1.72] |
| **824** | (0,0.00179] | NA | NA | (0,0.731] | NA |
| **825** | NA | (0.00125,0.00292] | NA | (0.731,0.936] | (1.72,Inf] |
| **826** | (0,0.00179] | (0.00292,0.00665] | NA | (1.11,Inf] | (1.72,Inf] |
| **827** | (0.00179,0.00386] | (0.00292,0.00665] | NA | (0.731,0.936] | (0.798,1.72] |
| **828** | (0.00386,0.00875] | (0.00665,Inf] | NA | (0,0.731] | (1.72,Inf] |
| **829** | (0,0.00179] | (0.00125,0.00292] | NA | (0.936,1.11] | (1.72,Inf] |
| **830** | (0,0.00179] | (0.00292,0.00665] | NA | (1.11,Inf] | (1.72,Inf] |
| **831** | (0.00875,Inf] | (0.00665,Inf] | NA | (0.731,0.936] | (0.213,0.798] |
| **832** | (0.00386,0.00875] | (0.00292,0.00665] | NA | (0.731,0.936] | (0.798,1.72] |

| | | | | | |
|---|---|---|---|---|---|
| **833** | (0.00875,Inf] | (0.00665,Inf] | NA | (0.936,1.11] | (0.798,1.72] |
| **834** | (0.00875,Inf] | (0.00665,Inf] | NA | (0.936,1.11] | (0.798,1.72] |
| **835** | (0.00386,0.00875] | (0.00665,Inf] | NA | (0,0.731] | (0.798,1.72] |
| **836** | (0.00179,0.00386] | (0.00125,0.00292] | NA | (0.731,0.936] | (0.213,0.798] |
| **837** | (0.00179,0.00386] | (0.00292,0.00665] | NA | (0,0.731] | (1.72,Inf] |
| **838** | (0.00179,0.00386] | (0,0.00125] | NA | (1.11,Inf] | (0,0.213] |
| **839** | NA | NA | NA | (0,0.731] | NA |
| **840** | (0,0.00179] | (0.00125,0.00292] | NA | (0.731,0.936] | (0.798,1.72] |
| **841** | (0.00179,0.00386] | NA | NA | (0.731,0.936] | NA |
| **842** | (0.00179,0.00386] | (0.00665,Inf] | NA | (1.11,Inf] | (1.72,Inf] |
| **843** | NA | NA | NA | (0.936,1.11] | NA |
| **844** | NA | NA | NA | (0.936,1.11] | NA |
| **845** | (0,0.00179] | (0.00125,0.00292] | NA | (1.11,Inf] | (1.72,Inf] |
| **846** | (0.00179,0.00386] | (0.00125,0.00292] | NA | (0.731,0.936] | (0.213,0.798] |
| **847** | (0.00386,0.00875] | (0.00292,0.00665] | NA | (0.731,0.936] | (0.213,0.798] |
| **848** | (0.00179,0.00386] | (0.00125,0.00292] | NA | (0,0.731] | (0.213,0.798] |
| **849** | (0,0.00179] | (0.00125,0.00292] | NA | (0,0.731] | (0.798,1.72] |
| **850** | (0,0.00179] | (0.00125,0.00292] | NA | (0,0.731] | (0.798,1.72] |
| **851** | (0.00179,0.00386] | (0,0.00125] | NA | (0.731,0.936] | (0,0.213] |
| **852** | (0.00179,0.00386] | NA | (0.46,Inf] | (0.731,0.936] | NA |
| **853** | (0.00179,0.00386] | (0.00292,0.00665] | NA | (0.731,0.936] | (0.798,1.72] |
| **854** | (0.00386,0.00875] | (0.00292,0.00665] | NA | (0,0.731] | (0.213,0.798] |
| **855** | (0,0.00179] | (0,0.00125] | NA | (0.936,1.11] | (0.213,0.798] |
| **856** | (0.00179,0.00386] | (0.00292,0.00665] | NA | (0,0.731] | (0.213,0.798] |
| **857** | (0.00386,0.00875] | (0.00292,0.00665] | (0.46,Inf] | (1.11,Inf] | (0.798,1.72] |
| **858** | (0,0.00179] | NA | NA | (0.936,1.11] | NA |
| **859** | (0.00386,0.00875] | NA | NA | (0.936,1.11] | NA |
| **860** | (0.00386,0.00875] | NA | NA | (0.731,0.936] | NA |
| **861** | (0.00875,Inf] | (0.00292,0.00665] | NA | (0.936,1.11] | (0.213,0.798] |
| **862** | (0.00386,0.00875] | (0.00292,0.00665] | NA | (0.731,0.936] | (0.798,1.72] |
| **863** | (0.00179,0.00386] | (0.00292,0.00665] | NA | (0,0.731] | (0.798,1.72] |
| **864** | (0.00179,0.00386] | (0.00125,0.00292] | NA | (0.936,1.11] | (0.213,0.798] |
| **865** | (0.00386,0.00875] | (0.00125,0.00292] | (0.46,Inf] | (0.731,0.936] | (0.213,0.798] |
| **866** | (0.00179,0.00386] | NA | NA | (0,0.731] | NA |
| **867** | (0.00179,0.00386] | NA | NA | (0.936,1.11] | NA |
| **868** | (0.00386,0.00875] | NA | NA | (0.936,1.11] | NA |
| **869** | (0.00179,0.00386] | (0.00292,0.00665] | NA | (0.936,1.11] | (0.798,1.72] |
| **870** | (0.00386,0.00875] | (0.00292,0.00665] | (0.46,Inf] | (0,0.731] | (0.213,0.798] |

| | | | | | |
|---|---|---|---|---|---|
| **871** | (0.00179,0.00386] | (0.00292,0.00665] | NA | (0.731,0.936] | (1.72,Inf] |
| **872** | (0.00875,Inf] | (0.00292,0.00665] | NA | (0.731,0.936] | (0.213,0.798] |
| **873** | (0.00875,Inf] | (0.00292,0.00665] | (0.46,Inf] | (1.11,Inf] | (0.213,0.798] |
| **874** | (0.00875,Inf] | (0.00665,Inf] | NA | (0.936,1.11] | (0.798,1.72] |
| **875** | (0,0.00179] | NA | NA | (0.731,0.936] | NA |
| **876** | (0.00179,0.00386] | (0.00665,Inf] | NA | (0.731,0.936] | (1.72,Inf] |
| **877** | (0.00179,0.00386] | (0.00125,0.00292] | NA | (0,0.731] | (0.213,0.798] |
| **878** | (0,0.00179] | NA | NA | (0.731,0.936] | NA |
| **879** | (0.00386,0.00875] | (0.00292,0.00665] | (0,0.46] | (1.11,Inf] | (0.798,1.72] |
| **880** | (0.00179,0.00386] | (0.00292,0.00665] | NA | (0,0.731] | (0.798,1.72] |
| **881** | (0.00179,0.00386] | (0.00125,0.00292] | NA | (0.936,1.11] | (0.213,0.798] |
| **882** | (0.00386,0.00875] | (0.00125,0.00292] | (0.46,Inf] | (0,0.731] | (0,0.213] |
| **883** | NA | (0,0.00125] | NA | (0,0.731] | (1.72,Inf] |
| **884** | (0.00386,0.00875] | (0.00125,0.00292] | NA | (0.731,0.936] | (0.213,0.798] |
| **885** | (0.00386,0.00875] | (0.00292,0.00665] | NA | (0.731,0.936] | (0.798,1.72] |
| **886** | (0.00875,Inf] | (0.00292,0.00665] | NA | (1.11,Inf] | (0.213,0.798] |
| **887** | (0,0.00179] | (0,0.00125] | NA | (0.936,1.11] | (0.798,1.72] |
| **888** | (0.00179,0.00386] | (0.00292,0.00665] | NA | (0,0.731] | (0.798,1.72] |
| **889** | (0.00386,0.00875] | (0.00292,0.00665] | (0.46,Inf] | (0.731,0.936] | (0.798,1.72] |
| **890** | NA | NA | NA | (0.731,0.936] | NA |
| **891** | (0.00386,0.00875] | (0.00292,0.00665] | NA | (0.731,0.936] | (0.213,0.798] |
| **892** | NA | NA | NA | (1.11,Inf] | NA |
| **893** | NA | NA | NA | (1.11,Inf] | NA |
| **894** | (0.00875,Inf] | (0.00665,Inf] | NA | (1.11,Inf] | (0.798,1.72] |
| **895** | (0.00386,0.00875] | (0.00125,0.00292] | NA | (0.936,1.11] | (0.213,0.798] |
| **896** | (0.00386,0.00875] | (0.00292,0.00665] | NA | (0.731,0.936] | (0.213,0.798] |
| **897** | (0.00386,0.00875] | NA | NA | (1.11,Inf] | NA |
| **898** | (0.00179,0.00386] | (0.00125,0.00292] | NA | (0.731,0.936] | (0.213,0.798] |
| **899** | (0,0.00179] | (0,0.00125] | NA | (0,0.731] | (0.213,0.798] |
| **900** | (0,0.00179] | (0.00125,0.00292] | NA | (0.936,1.11] | (1.72,Inf] |
| **901** | (0.00179,0.00386] | (0,0.00125] | NA | (0,0.731] | (0,0.213] |
| **902** | (0.00179,0.00386] | NA | NA | (0.936,1.11] | NA |
| **903** | (0.00386,0.00875] | (0.00665,Inf] | NA | (0.936,1.11] | (0.798,1.72] |
| **904** | (0,0.00179] | (0,0.00125] | NA | (0.731,0.936] | (0.798,1.72] |
| **905** | (0.00386,0.00875] | (0.00665,Inf] | (0,0.46] | (1.11,Inf] | (1.72,Inf] |
| **906** | (0.00386,0.00875] | (0.00125,0.00292] | NA | (1.11,Inf] | (0.213,0.798] |
| **907** | (0.00179,0.00386] | (0.00292,0.00665] | NA | (1.11,Inf] | (1.72,Inf] |
| **908** | (0,0.00179] | (0,0.00125] | NA | (0.936,1.11] | (0.798,1.72] |

| | | | | | |
|---|---|---|---|---|---|
| **909** | (0.00179,0.00386] | (0.00292,0.00665] | NA | (0.731,0.936] | (0.213,0.798] |
| **910** | (0,0.00179] | (0.00292,0.00665] | NA | (0,0.731] | (1.72,Inf] |
| **911** | (0.00386,0.00875] | (0.00665,Inf] | NA | (0.731,0.936] | (0.798,1.72] |
| **912** | (0,0.00179] | NA | NA | (0.936,1.11] | NA |
| **913** | NA | (0,0.00125] | NA | (1.11,Inf] | (1.72,Inf] |
| **914** | (0.00179,0.00386] | (0.00125,0.00292] | NA | (0.936,1.11] | (0.798,1.72] |
| **915** | NA | NA | (0,0.46] | NA | (0.798,1.72] |
| **916** | (0,0.00179] | NA | NA | (1.11,Inf] | NA |
| **917** | (0.00386,0.00875] | (0.00292,0.00665] | NA | (0.731,0.936] | (0.213,0.798] |
| **918** | (0.00386,0.00875] | (0.00292,0.00665] | NA | (0.731,0.936] | (0.213,0.798] |
| **919** | (0,0.00179] | NA | NA | (0,0.731] | NA |
| **920** | (0.00179,0.00386] | NA | (0.46,Inf] | (0.731,0.936] | NA |
| **921** | (0.00875,Inf] | (0.00665,Inf] | NA | (0.731,0.936] | (0.213,0.798] |
| **922** | (0.00875,Inf] | (0.00665,Inf] | NA | (0,0.731] | (0.798,1.72] |
| **923** | (0.00179,0.00386] | (0.00292,0.00665] | NA | (0.936,1.11] | (0.798,1.72] |
| **924** | (0.00179,0.00386] | (0.00125,0.00292] | NA | (0.936,1.11] | (0.798,1.72] |
| **925** | (0.00875,Inf] | (0.00665,Inf] | NA | (0.936,1.11] | (0.213,0.798] |
| **926** | (0,0.00179] | (0.00125,0.00292] | NA | (0.731,0.936] | (1.72,Inf] |
| **927** | (0.00875,Inf] | (0.00292,0.00665] | NA | (1.11,Inf] | (0.213,0.798] |
| **928** | NA | NA | NA | (0.731,0.936] | NA |
| **929** | (0,0.00179] | (0.00665,Inf] | NA | (0.731,0.936] | (1.72,Inf] |
| **930** | (0.00875,Inf] | (0.00292,0.00665] | NA | (0.731,0.936] | (0.213,0.798] |
| **931** | (0.00386,0.00875] | NA | NA | (0.936,1.11] | NA |
| **932** | (0.00875,Inf] | (0.00292,0.00665] | (0.46,Inf] | (0.731,0.936] | (0.213,0.798] |
| **933** | (0.00875,Inf] | (0.00292,0.00665] | (0.46,Inf] | (1.11,Inf] | (0.213,0.798] |
| **934** | (0.00386,0.00875] | NA | NA | (0.936,1.11] | NA |
| **935** | (0.00179,0.00386] | (0,0.00125] | NA | (0,0.731] | (0,0.213] |
| **936** | (0.00386,0.00875] | (0,0.00125] | NA | (0.731,0.936] | (0,0.213] |
| **937** | (0.00386,0.00875] | (0,0.00125] | NA | (0.731,0.936] | (0,0.213] |
| **938** | (0.00875,Inf] | (0.00125,0.00292] | NA | (1.11,Inf] | (0.213,0.798] |
| **939** | (0.00386,0.00875] | NA | NA | (0.731,0.936] | NA |
| **940** | (0.00386,0.00875] | (0.00665,Inf] | NA | (0.731,0.936] | (0.798,1.72] |
| **941** | (0.00875,Inf] | (0.00665,Inf] | NA | (0,0.731] | (0.798,1.72] |
| **942** | (0,0.00179] | (0.00292,0.00665] | (0.46,Inf] | (0,0.731] | (1.72,Inf] |
| **943** | (0.00875,Inf] | (0.00292,0.00665] | (0.46,Inf] | (1.11,Inf] | (0.213,0.798] |
| **944** | (0.00386,0.00875] | (0.00665,Inf] | (0.46,Inf] | (0.936,1.11] | (0.798,1.72] |
| **945** | (0.00179,0.00386] | NA | NA | (1.11,Inf] | NA |
| **946** | (0.00386,0.00875] | (0,0.00125] | NA | (0.731,0.936] | (0,0.213] |

| | | | | | |
|---|---|---|---|---|---|
| **947** | (0.00386,0.00875] | (0.00292,0.00665] | (0,0.46] | (0,0.731] | (0.213,0.798] |
| **948** | (0.00386,0.00875] | (0.00292,0.00665] | NA | (0,0.731] | (0.213,0.798] |
| **949** | (0,0.00179] | (0,0.00125] | NA | (1.11,Inf] | (0.798,1.72] |
| **950** | (0.00386,0.00875] | (0.00125,0.00292] | NA | (1.11,Inf] | (0.798,1.72] |
| **951** | (0.00179,0.00386] | NA | (0.46,Inf] | (0.731,0.936] | NA |
| **952** | (0.00386,0.00875] | (0.00292,0.00665] | (0,0.46] | (0.731,0.936] | (0.213,0.798] |
| **953** | (0.00386,0.00875] | (0,0.00125] | NA | (0.936,1.11] | (0,0.213] |
| **954** | NA | NA | NA | (0.936,1.11] | NA |
| **955** | (0.00179,0.00386] | (0.00665,Inf] | NA | (0.936,1.11] | (1.72,Inf] |
| **956** | (0.00875,Inf] | (0.00665,Inf] | NA | (0.936,1.11] | (0.798,1.72] |
| **957** | (0.00179,0.00386] | (0.00125,0.00292] | NA | (1.11,Inf] | (0.213,0.798] |
| **958** | (0.00875,Inf] | (0.00665,Inf] | NA | (0.936,1.11] | (0.213,0.798] |
| **959** | NA | NA | NA | (0.731,0.936] | NA |
| **960** | (0.00875,Inf] | (0.00665,Inf] | (0,0.46] | (0.731,0.936] | (0.213,0.798] |
| **961** | (0,0.00179] | (0.00125,0.00292] | NA | (0.731,0.936] | (0.798,1.72] |
| **962** | (0,0.00179] | (0.00665,Inf] | NA | (0.731,0.936] | (1.72,Inf] |
| **963** | (0.00386,0.00875] | (0.00665,Inf] | NA | (0.731,0.936] | (1.72,Inf] |
| **964** | (0.00875,Inf] | NA | NA | (0.936,1.11] | NA |
| **965** | (0.00875,Inf] | (0.00665,Inf] | NA | (0,0.731] | (0.798,1.72] |
| **966** | (0.00179,0.00386] | (0.00125,0.00292] | NA | (0.936,1.11] | (0.798,1.72] |
| **967** | (0.00875,Inf] | (0.00665,Inf] | NA | (0,0.731] | (0.213,0.798] |
| **968** | (0,0.00179] | (0.00292,0.00665] | NA | (0.731,0.936] | (1.72,Inf] |
| **969** | (0.00386,0.00875] | (0,0.00125] | NA | (0.936,1.11] | (0,0.213] |
| **970** | (0.00386,0.00875] | (0.00292,0.00665] | (0.46,Inf] | (0.731,0.936] | (0.798,1.72] |
| **971** | NA | NA | NA | (0.731,0.936] | NA |
| **972** | (0.00386,0.00875] | NA | NA | (0.731,0.936] | NA |
| **973** | (0.00875,Inf] | (0.00665,Inf] | NA | (0.731,0.936] | (0.798,1.72] |
| **974** | (0.00875,Inf] | (0.00665,Inf] | (0.46,Inf] | (0.936,1.11] | (0.213,0.798] |
| **975** | NA | (0.00665,Inf] | NA | (0.731,0.936] | (1.72,Inf] |
| **976** | (0.00179,0.00386] | (0.00292,0.00665] | NA | (0.731,0.936] | (0.798,1.72] |
| **977** | (0.00875,Inf] | (0.00292,0.00665] | (0.46,Inf] | (1.11,Inf] | (0.213,0.798] |
| **978** | (0.00386,0.00875] | (0.00292,0.00665] | NA | (0.731,0.936] | (0.213,0.798] |
| **979** | (0.00875,Inf] | (0.00665,Inf] | NA | (0.731,0.936] | (0.213,0.798] |
| **980** | (0.00386,0.00875] | (0.00125,0.00292] | NA | (1.11,Inf] | (0.798,1.72] |
| **981** | NA | NA | NA | (0.936,1.11] | NA |
| **982** | NA | NA | NA | (0.731,0.936] | NA |
| **983** | (0.00386,0.00875] | (0.00292,0.00665] | NA | (1.11,Inf] | (0.798,1.72] |
| **984** | NA | NA | NA | (0.731,0.936] | NA |

| | | | | | |
|---|---|---|---|---|---|
| **985** | (0.00179,0.00386] | NA | NA | (0.731,0.936] | NA |
| **986** | (0.00386,0.00875] | (0,0.00125] | (0.46,Inf] | (0.936,1.11] | (0,0.213] |
| **987** | (0.00386,0.00875] | (0,0.00125] | NA | (0.936,1.11] | (0,0.213] |
| **988** | (0,0.00179] | (0.00125,0.00292] | NA | (0,0.731] | (0.798,1.72] |
| **989** | (0.00179,0.00386] | (0.00665,Inf] | NA | (0,0.731] | (1.72,Inf] |
| **990** | (0.00875,Inf] | NA | NA | (1.11,Inf] | NA |
| **991** | (0,0.00179] | (0.00665,Inf] | (0,0.46] | (0.936,1.11] | (1.72,Inf] |
| **992** | (0.00386,0.00875] | (0,0.00125] | NA | (0.731,0.936] | (0,0.213] |
| **993** | (0.00875,Inf] | (0.00292,0.00665] | NA | (1.11,Inf] | (0.213,0.798] |
| **994** | (0,0.00179] | (0,0.00125] | NA | (0.731,0.936] | (0.213,0.798] |
| **995** | NA | (0.00665,Inf] | NA | (0,0.731] | (1.72,Inf] |
| **996** | NA | NA | NA | (0.731,0.936] | NA |
| **997** | NA | (0.00292,0.00665] | NA | (0.936,1.11] | (1.72,Inf] |
| **998** | (0.00386,0.00875] | (0.00292,0.00665] | NA | (0.731,0.936] | (0.213,0.798] |
| **999** | (0.00386,0.00875] | (0.00292,0.00665] | NA | (0.731,0.936] | (0.798,1.72] |
| **1000** | (0.00179,0.00386] | (0.00292,0.00665] | NA | (0,0.731] | (0.798,1.72] |
| **1001** | NA | (0,0.00125] | NA | (0.731,0.936] | (1.72,Inf] |
| **1002** | (0.00386,0.00875] | (0.00292,0.00665] | NA | (0.731,0.936] | (0.213,0.798] |
| **1003** | NA | (0.00292,0.00665] | NA | (0.731,0.936] | (1.72,Inf] |
| **1004** | (0.00386,0.00875] | (0.00125,0.00292] | NA | (1.11,Inf] | (0.213,0.798] |
| **1005** | NA | NA | NA | (0,0.731] | NA |
| **1006** | (0.00179,0.00386] | NA | NA | (0.731,0.936] | NA |
| **1007** | (0.00875,Inf] | (0.00292,0.00665] | (0.46,Inf] | (0.936,1.11] | (0.213,0.798] |
| **1008** | (0.00386,0.00875] | (0,0.00125] | NA | (1.11,Inf] | (0.213,0.798] |
| **1009** | (0.00179,0.00386] | NA | NA | (1.11,Inf] | NA |
| **1010** | (0.00386,0.00875] | (0.00665,Inf] | NA | (0.731,0.936] | (0.798,1.72] |
| **1011** | (0.00386,0.00875] | NA | NA | (0.936,1.11] | NA |
| **1012** | NA | NA | NA | (0,0.731] | NA |
| **1013** | (0,0.00179] | (0.00125,0.00292] | NA | (1.11,Inf] | (1.72,Inf] |
| **1014** | NA | NA | NA | (0.936,1.11] | NA |
| **1015** | (0,0.00179] | (0.00125,0.00292] | NA | (0.936,1.11] | (0.798,1.72] |
| **1016** | NA | (0,0.00125] | NA | (0.731,0.936] | (1.72,Inf] |
| **1017** | (0.00179,0.00386] | NA | NA | (0,0.731] | NA |
| **1018** | (0.00386,0.00875] | (0.00125,0.00292] | NA | (0.936,1.11] | (0.213,0.798] |
| **1019** | (0.00179,0.00386] | (0.00665,Inf] | NA | (0,0.731] | (1.72,Inf] |
| **1020** | NA | NA | NA | (0,0.731] | NA |
| **1021** | NA | NA | NA | (0,0.731] | NA |
| **1022** | (0.00179,0.00386] | (0.00125,0.00292] | NA | (0.731,0.936] | (0.798,1.72] |

| | | | | | |
|---|---|---|---|---|---|
| **1023** | (0.00875,Inf] | (0.00125,0.00292] | NA | (0.936,1.11] | (0,0.213] |
| **1024** | NA | NA | NA | (0.731,0.936] | NA |
| **1025** | (0.00386,0.00875] | (0.00665,Inf] | NA | (0.936,1.11] | (0.798,1.72] |
| **1026** | (0.00875,Inf] | (0.00292,0.00665] | NA | (0.731,0.936] | (0.213,0.798] |
| **1027** | NA | (0,0.00125] | NA | (0.936,1.11] | (1.72,Inf] |
| **1028** | NA | (0.00125,0.00292] | NA | (0.731,0.936] | (1.72,Inf] |
| **1029** | NA | NA | NA | (1.11,Inf] | NA |
| **1030** | (0.00386,0.00875] | (0.00292,0.00665] | (0,0.46] | (0.936,1.11] | (0.213,0.798] |
| **1031** | (0.00386,0.00875] | NA | NA | (0.936,1.11] | NA |
| **1032** | (0.00875,Inf] | (0.00665,Inf] | NA | (0.936,1.11] | (0.213,0.798] |
| **1033** | (0.00386,0.00875] | (0,0.00125] | NA | (0.936,1.11] | (0,0.213] |
| **1034** | (0.00386,0.00875] | (0.00125,0.00292] | NA | (0.731,0.936] | (0.213,0.798] |
| **1035** | NA | NA | (0.46,Inf] | (0,0.731] | NA |
| **1036** | (0.00386,0.00875] | (0.00292,0.00665] | (0.46,Inf] | (0.936,1.11] | (0.213,0.798] |
| **1037** | (0.00179,0.00386] | NA | NA | (0.731,0.936] | NA |
| **1038** | (0.00386,0.00875] | (0.00665,Inf] | NA | (1.11,Inf] | (1.72,Inf] |
| **1039** | (0.00386,0.00875] | (0.00665,Inf] | NA | (0,0.731] | (1.72,Inf] |
| **1040** | NA | (0.00292,0.00665] | NA | (0.731,0.936] | (1.72,Inf] |
| **1041** | (0.00875,Inf] | (0.00665,Inf] | (0.46,Inf] | (0.936,1.11] | (0.798,1.72] |
| **1042** | (0.00386,0.00875] | (0.00292,0.00665] | NA | (1.11,Inf] | (0.213,0.798] |
| **1043** | NA | (0,0.00125] | NA | (0.731,0.936] | (1.72,Inf] |
| **1044** | (0.00875,Inf] | (0.00665,Inf] | NA | (1.11,Inf] | (0.798,1.72] |
| **1045** | (0.00386,0.00875] | (0.00292,0.00665] | NA | (0.936,1.11] | (0.798,1.72] |
| **1046** | (0.00386,0.00875] | (0.00125,0.00292] | (0.46,Inf] | (1.11,Inf] | (0.213,0.798] |
| **1047** | (0.00386,0.00875] | NA | NA | (0,0.731] | NA |
| **1048** | NA | NA | NA | (0.936,1.11] | NA |
| **1049** | (0.00875,Inf] | (0.00292,0.00665] | NA | (1.11,Inf] | (0.213,0.798] |
| **1050** | (0.00386,0.00875] | (0,0.00125] | NA | (0.936,1.11] | (0,0.213] |
| **1051** | (0.00179,0.00386] | (0.00125,0.00292] | NA | (1.11,Inf] | (0.798,1.72] |
| **1052** | (0.00875,Inf] | (0.00665,Inf] | NA | (1.11,Inf] | (1.72,Inf] |
| **1053** | (0.00875,Inf] | (0.00665,Inf] | NA | (0.936,1.11] | (0.213,0.798] |
| **1054** | (0.00875,Inf] | (0,0.00125] | NA | (1.11,Inf] | (0,0.213] |
| **1055** | (0.00875,Inf] | (0.00665,Inf] | NA | (0,0.731] | (0.798,1.72] |
| **1056** | NA | NA | NA | (0,0.731] | NA |
| **1057** | (0.00875,Inf] | (0.00125,0.00292] | NA | (1.11,Inf] | (0,0.213] |
| **1058** | NA | (0.00125,0.00292] | NA | (1.11,Inf] | (1.72,Inf] |
| **1059** | (0,0.00179] | (0.00292,0.00665] | NA | (1.11,Inf] | (1.72,Inf] |
| **1060** | (0.00386,0.00875] | (0.00125,0.00292] | NA | (0.731,0.936] | (0.213,0.798] |

| | | | | | |
|---|---|---|---|---|---|
| **1061** | NA | NA | NA | (0.731,0.936] | NA |
| **1062** | (0.00386,0.00875] | (0.00292,0.00665] | NA | (0,0.731] | (0.213,0.798] |
| **1063** | (0.00179,0.00386] | (0.00125,0.00292] | NA | (0.731,0.936] | (0.213,0.798] |
| **1064** | (0.00875,Inf] | (0.00125,0.00292] | (0.46,Inf] | (1.11,Inf] | (0,0.213] |
| **1065** | NA | NA | NA | (0,0.731] | NA |
| **1066** | (0.00386,0.00875] | (0.00292,0.00665] | NA | (0.731,0.936] | (0.213,0.798] |
| **1067** | (0.00875,Inf] | (0.00665,Inf] | NA | (1.11,Inf] | (1.72,Inf] |
| **1068** | (0.00875,Inf] | (0.00292,0.00665] | NA | (0.731,0.936] | (0.213,0.798] |
| **1069** | (0.00386,0.00875] | NA | NA | (0.731,0.936] | NA |
| **1070** | (0.00386,0.00875] | (0.00665,Inf] | NA | (0.936,1.11] | (0.798,1.72] |
| **1071** | NA | NA | NA | (0.731,0.936] | NA |
| **1072** | NA | NA | NA | (0.936,1.11] | NA |
| **1073** | NA | NA | NA | (0,0.731] | NA |
| **1074** | (0.00179,0.00386] | (0,0.00125] | NA | (0.731,0.936] | (0.213,0.798] |
| **1075** | (0.00875,Inf] | (0.00665,Inf] | NA | (0,0.731] | (0.798,1.72] |
| **1076** | (0.00386,0.00875] | (0.00125,0.00292] | NA | (0.731,0.936] | (0,0.213] |
| **1077** | NA | NA | NA | (1.11,Inf] | NA |
| **1078** | NA | (0.00292,0.00665] | NA | (0.936,1.11] | (1.72,Inf] |
| **1079** | NA | NA | NA | (0.731,0.936] | NA |
| **1080** | (0.00386,0.00875] | NA | NA | (1.11,Inf] | NA |
| **1081** | (0.00179,0.00386] | NA | NA | (0,0.731] | NA |
| **1082** | (0.00179,0.00386] | (0.00665,Inf] | NA | (0,0.731] | (0.798,1.72] |
| **1083** | (0.00179,0.00386] | NA | NA | (0,0.731] | NA |
| **1084** | NA | NA | NA | (1.11,Inf] | NA |
| **1085** | (0.00179,0.00386] | (0.00665,Inf] | NA | (0.936,1.11] | (1.72,Inf] |
| **1086** | (0.00875,Inf] | (0.00665,Inf] | (0,0.46] | (0.936,1.11] | (0.798,1.72] |
| **1087** | (0,0.00179] | (0.00125,0.00292] | NA | (1.11,Inf] | (1.72,Inf] |
| **1088** | (0.00875,Inf] | (0.00292,0.00665] | NA | (0.936,1.11] | (0.213,0.798] |
| **1089** | (0.00875,Inf] | (0.00665,Inf] | (0,0.46] | (1.11,Inf] | (0.798,1.72] |
| **1090** | (0.00179,0.00386] | (0.00665,Inf] | NA | (0.936,1.11] | (1.72,Inf] |
| **1091** | (0.00875,Inf] | (0.00665,Inf] | NA | (0.731,0.936] | (0.213,0.798] |
| **1092** | (0,0.00179] | NA | NA | (0,0.731] | NA |
| **1093** | (0.00875,Inf] | (0.00665,Inf] | NA | (0.731,0.936] | (0.213,0.798] |
| **1094** | (0.00386,0.00875] | NA | NA | (0.936,1.11] | NA |
| **1095** | NA | (0.00292,0.00665] | NA | (1.11,Inf] | (1.72,Inf] |
| **1096** | (0,0.00179] | NA | NA | (0,0.731] | NA |
| **1097** | (0.00875,Inf] | (0.00125,0.00292] | (0.46,Inf] | (0,0.731] | (0,0.213] |
| **1098** | (0.00386,0.00875] | (0,0.00125] | NA | (0.731,0.936] | (0,0.213] |

| | | | | | |
|---|---|---|---|---|---|
| **1099** | (0.00875,Inf] | (0.00665,Inf] | (0.46,Inf] | (1.11,Inf] | (0.798,1.72] |
| **1100** | NA | (0.00665,Inf] | NA | (0,0.731] | (1.72,Inf] |
| **1101** | (0.00875,Inf] | (0.00665,Inf] | NA | (0.936,1.11] | (0.798,1.72] |
| **1102** | (0.00875,Inf] | (0.00292,0.00665] | NA | (0,0.731] | (0.213,0.798] |
| **1103** | (0.00875,Inf] | NA | NA | (1.11,Inf] | NA |
| **1104** | (0.00875,Inf] | (0.00125,0.00292] | NA | (0.731,0.936] | (0,0.213] |
| **1105** | (0.00386,0.00875] | (0.00125,0.00292] | NA | (0.936,1.11] | (0.213,0.798] |
| **1106** | (0.00386,0.00875] | (0.00292,0.00665] | NA | (0.936,1.11] | (0.798,1.72] |
| **1107** | (0.00386,0.00875] | (0.00665,Inf] | NA | (0.731,0.936] | (1.72,Inf] |
| **1108** | (0.00875,Inf] | (0.00665,Inf] | NA | (1.11,Inf] | (0.213,0.798] |
| **1109** | (0.00386,0.00875] | NA | NA | (0.936,1.11] | NA |
| **1110** | (0.00875,Inf] | (0.00665,Inf] | NA | (1.11,Inf] | (0.213,0.798] |
| **1111** | (0.00875,Inf] | NA | NA | (0.936,1.11] | NA |
| **1112** | (0,0.00179] | (0,0.00125] | NA | (1.11,Inf] | (1.72,Inf] |
| **1113** | NA | (0,0.00125] | NA | (0.936,1.11] | (1.72,Inf] |
| **1114** | (0.00179,0.00386] | (0.00665,Inf] | NA | (0.731,0.936] | (1.72,Inf] |
| **1115** | NA | (0.00292,0.00665] | NA | (0,0.731] | (1.72,Inf] |
| **1116** | (0,0.00179] | (0,0.00125] | NA | (0,0.731] | (0.213,0.798] |
| **1117** | (0.00875,Inf] | NA | NA | (0.731,0.936] | NA |
| **1118** | NA | (0,0.00125] | NA | (0.731,0.936] | (1.72,Inf] |
| **1119** | (0.00875,Inf] | (0,0.00125] | NA | (0.731,0.936] | (0,0.213] |
| **1120** | NA | (0.00665,Inf] | NA | (1.11,Inf] | (1.72,Inf] |
| **1121** | (0.00875,Inf] | (0.00665,Inf] | (0.46,Inf] | (0.731,0.936] | (0.213,0.798] |
| **1122** | (0,0.00179] | (0.00292,0.00665] | NA | (0,0.731] | (0.798,1.72] |
| **1123** | (0.00875,Inf] | (0.00292,0.00665] | NA | (0,0.731] | (0,0.213] |
| **1124** | NA | NA | NA | (0.731,0.936] | NA |
| **1125** | (0.00875,Inf] | (0.00665,Inf] | NA | (1.11,Inf] | (0.213,0.798] |
| **1126** | (0.00875,Inf] | (0.00665,Inf] | NA | (1.11,Inf] | (0.798,1.72] |
| **1127** | NA | NA | (0,0.46] | NA | (0.798,1.72] |
| **1128** | (0.00179,0.00386] | NA | NA | (0,0.731] | NA |
| **1129** | (0.00875,Inf] | (0.00125,0.00292] | (0.46,Inf] | (1.11,Inf] | (0,0.213] |
| **1130** | NA | NA | NA | (0.731,0.936] | NA |
| **1131** | (0.00386,0.00875] | (0.00665,Inf] | NA | (1.11,Inf] | (1.72,Inf] |
| **1132** | (0.00875,Inf] | (0.00292,0.00665] | NA | (1.11,Inf] | (0.213,0.798] |
| **1133** | (0.00179,0.00386] | NA | NA | (0.936,1.11] | NA |
| **1134** | NA | NA | NA | (0.731,0.936] | NA |
| **1135** | (0.00875,Inf] | (0.00665,Inf] | NA | (0.731,0.936] | (0.213,0.798] |
| **1136** | (0,0.00179] | (0.00292,0.00665] | NA | (1.11,Inf] | (1.72,Inf] |

| | | | | | |
|---|---|---|---|---|---|
| **1137** | NA | (0.00125,0.00292] | NA | (0.936,1.11] | (1.72,Inf] |
| **1138** | (0.00386,0.00875] | (0.00125,0.00292] | NA | (0,0.731] | (0,0.213] |
| **1139** | (0.00179,0.00386] | (0.00292,0.00665] | NA | (1.11,Inf] | (0.798,1.72] |
| **1140** | NA | (0.00292,0.00665] | NA | (0,0.731] | (1.72,Inf] |
| **1141** | (0.00875,Inf] | (0.00665,Inf] | NA | (0,0.731] | (0.213,0.798] |
| **1142** | (0.00179,0.00386] | NA | NA | (0,0.731] | NA |
| **1143** | (0.00875,Inf] | (0.00665,Inf] | NA | (1.11,Inf] | (1.72,Inf] |
| **1144** | NA | NA | NA | (1.11,Inf] | NA |
| **1145** | (0.00875,Inf] | (0.00665,Inf] | (0,0.46] | (0.731,0.936] | (0.798,1.72] |
| **1146** | (0.00386,0.00875] | (0.00125,0.00292] | NA | (0.936,1.11] | (0.213,0.798] |
| **1147** | (0.00386,0.00875] | (0.00665,Inf] | NA | (0.731,0.936] | (0.798,1.72] |
| **1148** | (0.00875,Inf] | (0.00665,Inf] | (0.46,Inf] | (0.936,1.11] | (0.213,0.798] |
| **1149** | NA | (0.00125,0.00292] | NA | (0.731,0.936] | (1.72,Inf] |
| **1150** | (0.00386,0.00875] | (0.00125,0.00292] | NA | (0,0.731] | (0,0.213] |
| **1151** | (0,0.00179] | (0,0.00125] | NA | (0,0.731] | (1.72,Inf] |
| **1152** | (0.00386,0.00875] | (0.00292,0.00665] | NA | (0,0.731] | (0.213,0.798] |
| **1153** | (0.00386,0.00875] | NA | NA | (0.731,0.936] | NA |
| **1154** | NA | NA | NA | (0.936,1.11] | NA |
| **1155** | (0.00179,0.00386] | (0.00665,Inf] | NA | (0.731,0.936] | (0.798,1.72] |
| **1156** | NA | (0.00665,Inf] | NA | (0,0.731] | (1.72,Inf] |
| **1157** | (0.00386,0.00875] | NA | NA | (0.731,0.936] | NA |
| **1158** | NA | NA | NA | (0.936,1.11] | NA |
| **1159** | NA | (0.00292,0.00665] | NA | (0.936,1.11] | (1.72,Inf] |
| **1160** | (0.00875,Inf] | NA | NA | (1.11,Inf] | NA |
| **1161** | NA | (0.00665,Inf] | NA | (0,0.731] | (1.72,Inf] |
| **1162** | NA | NA | NA | (0.936,1.11] | NA |
| **1163** | (0.00386,0.00875] | (0.00292,0.00665] | NA | (0,0.731] | (0.213,0.798] |
| **1164** | (0.00179,0.00386] | NA | NA | (0,0.731] | NA |
| **1165** | (0.00386,0.00875] | (0.00665,Inf] | (0.46,Inf] | (0.731,0.936] | (0.798,1.72] |
| **1166** | (0.00179,0.00386] | NA | NA | (0.731,0.936] | NA |
| **1167** | NA | NA | NA | (0,0.731] | NA |
| **1168** | (0.00875,Inf] | NA | NA | (1.11,Inf] | NA |
| **1169** | (0,0.00179] | (0,0.00125] | NA | (0.731,0.936] | (0.798,1.72] |
| **1170** | (0.00386,0.00875] | (0.00665,Inf] | (0.46,Inf] | (0,0.731] | (0.798,1.72] |
| **1171** | NA | NA | (0.46,Inf] | (0.936,1.11] | NA |
| **1172** | (0.00875,Inf] | (0.00665,Inf] | NA | (0.731,0.936] | (0.798,1.72] |
| **1173** | (0.00179,0.00386] | (0.00125,0.00292] | NA | (0,0.731] | (0.213,0.798] |
| **1174** | (0.00386,0.00875] | (0.00292,0.00665] | NA | (1.11,Inf] | (0.213,0.798] |

| | | | | | |
|---|---|---|---|---|---|
| **1175** | NA | NA | NA | (0,0.731] | NA |
| **1176** | (0.00179,0.00386] | NA | NA | (0,0.731] | NA |
| **1177** | (0.00875,Inf] | NA | NA | (1.11,Inf] | NA |
| **1178** | (0.00179,0.00386] | (0.00125,0.00292] | NA | (0.936,1.11] | (0.213,0.798] |
| **1179** | NA | NA | NA | (0.936,1.11] | NA |
| **1180** | (0.00179,0.00386] | (0.00665,Inf] | NA | (0,0.731] | (1.72,Inf] |
| **1181** | (0,0.00179] | NA | NA | (0.731,0.936] | NA |
| **1182** | (0.00386,0.00875] | (0.00665,Inf] | NA | (1.11,Inf] | (0.798,1.72] |
| **1183** | NA | NA | NA | (0.936,1.11] | NA |
| **1184** | (0.00386,0.00875] | (0.00125,0.00292] | (0.46,Inf] | (0,0.731] | (0,0.213] |
| **1185** | NA | NA | NA | (0,0.731] | NA |
| **1186** | (0.00386,0.00875] | (0.00292,0.00665] | NA | (0.936,1.11] | (0.798,1.72] |
| **1187** | (0.00875,Inf] | (0.00665,Inf] | NA | (0,0.731] | (0.213,0.798] |
| **1188** | (0.00179,0.00386] | (0.00292,0.00665] | NA | (0,0.731] | (0.798,1.72] |
| **1189** | NA | NA | NA | (0,0.731] | NA |
| **1190** | (0.00875,Inf] | NA | NA | (0,0.731] | NA |
| **1191** | NA | (0.00125,0.00292] | NA | (0.731,0.936] | (1.72,Inf] |
| **1192** | NA | NA | NA | (0,0.731] | NA |
| **1193** | (0.00875,Inf] | (0.00125,0.00292] | NA | (0,0.731] | (0,0.213] |
| **1194** | (0.00386,0.00875] | (0.00665,Inf] | NA | (0,0.731] | (1.72,Inf] |
| **1195** | NA | (0.00125,0.00292] | NA | (0.936,1.11] | (1.72,Inf] |
| **1196** | NA | NA | NA | (0.936,1.11] | NA |
| **1197** | (0.00875,Inf] | (0,0.00125] | NA | (0.936,1.11] | (0,0.213] |
| **1198** | NA | NA | NA | NA | (0.213,0.798] |
| **1199** | (0.00179,0.00386] | (0.00665,Inf] | NA | (0.731,0.936] | (1.72,Inf] |
| **1200** | (0.00875,Inf] | (0.00665,Inf] | NA | (1.11,Inf] | (0.213,0.798] |
| **1201** | NA | (0.00665,Inf] | NA | (0.731,0.936] | (1.72,Inf] |
| **1202** | (0.00875,Inf] | (0.00292,0.00665] | NA | (1.11,Inf] | (0.213,0.798] |
| **1203** | NA | NA | NA | (1.11,Inf] | NA |
| **1204** | (0.00875,Inf] | (0.00292,0.00665] | (0.46,Inf] | (0,0.731] | (0.213,0.798] |
| **1205** | NA | NA | NA | (0.731,0.936] | NA |
| **1206** | (0.00875,Inf] | (0.00665,Inf] | (0,0.46] | (1.11,Inf] | (1.72,Inf] |
| **1207** | (0.00875,Inf] | (0.00665,Inf] | NA | (0,0.731] | (0.213,0.798] |
| **1208** | (0.00875,Inf] | (0.00665,Inf] | NA | (1.11,Inf] | (0.798,1.72] |
| **1209** | (0.00875,Inf] | (0.00665,Inf] | NA | (1.11,Inf] | (0.213,0.798] |
| **1210** | NA | NA | NA | (0.731,0.936] | NA |
| **1211** | NA | NA | NA | (0.731,0.936] | NA |
| **1212** | (0.00179,0.00386] | (0.00125,0.00292] | NA | (0,0.731] | (0.213,0.798] |

| | | | | | |
|---|---|---|---|---|---|
| **1213** | (0.00875,Inf] | (0.00665,Inf] | NA | (0.936,1.11] | (1.72,Inf] |
| **1214** | (0,0.00179] | NA | NA | (0,0.731] | NA |
| **1215** | (0.00875,Inf] | (0.00665,Inf] | NA | (0,0.731] | (0.213,0.798] |
| **1216** | (0.00875,Inf] | NA | NA | (0,0.731] | NA |
| **1217** | NA | NA | NA | (0,0.731] | NA |
| **1218** | (0.00875,Inf] | (0.00292,0.00665] | NA | (1.11,Inf] | (0,0.213] |
| **1219** | (0.00179,0.00386] | (0.00292,0.00665] | NA | (0.936,1.11] | (0.798,1.72] |
| **1220** | (0.00875,Inf] | NA | NA | (0.731,0.936] | NA |
| **1221** | (0.00386,0.00875] | (0.00665,Inf] | NA | (0,0.731] | (0.213,0.798] |
| **1222** | (0.00386,0.00875] | (0.00292,0.00665] | NA | (1.11,Inf] | (0.213,0.798] |
| **1223** | (0.00386,0.00875] | (0.00665,Inf] | NA | (0,0.731] | (1.72,Inf] |
| **1224** | (0.00875,Inf] | (0.00665,Inf] | NA | (0.936,1.11] | (0.213,0.798] |
| **1225** | (0.00386,0.00875] | NA | NA | (0.936,1.11] | NA |
| **1226** | (0.00386,0.00875] | NA | NA | (1.11,Inf] | NA |
| **1227** | NA | NA | NA | NA | (0.798,1.72] |
| **1228** | NA | NA | NA | (0,0.731] | NA |
| **1229** | (0.00179,0.00386] | NA | NA | (0.936,1.11] | NA |
| **1230** | NA | (0.00125,0.00292] | NA | (1.11,Inf] | (1.72,Inf] |
| **1231** | (0.00386,0.00875] | (0.00125,0.00292] | NA | (0.936,1.11] | (0.213,0.798] |
| **1232** | NA | NA | NA | (0.731,0.936] | NA |
| **1233** | NA | NA | NA | (0.731,0.936] | NA |
| **1234** | NA | NA | NA | (1.11,Inf] | NA |
| **1235** | (0.00386,0.00875] | NA | NA | (0.731,0.936] | NA |
| **1236** | NA | (0.00125,0.00292] | NA | (0,0.731] | (1.72,Inf] |
| **1237** | (0.00875,Inf] | NA | NA | (1.11,Inf] | NA |
| **1238** | (0.00386,0.00875] | (0.00292,0.00665] | NA | (1.11,Inf] | (0.213,0.798] |
| **1239** | (0.00386,0.00875] | NA | NA | (0.731,0.936] | NA |
| **1240** | (0.00179,0.00386] | (0.00125,0.00292] | NA | (0,0.731] | (0.213,0.798] |
| **1241** | (0.00875,Inf] | (0.00665,Inf] | NA | (0.936,1.11] | (0.798,1.72] |
| **1242** | (0.00875,Inf] | (0.00665,Inf] | NA | (0.936,1.11] | (1.72,Inf] |
| **1243** | (0.00386,0.00875] | (0.00292,0.00665] | NA | (0.936,1.11] | (0.213,0.798] |
| **1244** | (0.00386,0.00875] | NA | NA | (0.731,0.936] | NA |
| **1245** | (0.00386,0.00875] | (0.00292,0.00665] | NA | (0,0.731] | (0.213,0.798] |
| **1246** | (0.00386,0.00875] | (0.00292,0.00665] | NA | (1.11,Inf] | (0.798,1.72] |
| **1247** | (0,0.00179] | NA | NA | (0.936,1.11] | NA |
| **1248** | (0,0.00179] | (0.00292,0.00665] | NA | (0,0.731] | (1.72,Inf] |
| **1249** | NA | NA | NA | (0,0.731] | NA |
| **1250** | NA | NA | NA | (0,0.731] | NA |

| | | | | | |
|---|---|---|---|---|---|
| **1251** | (0.00179,0.00386] | (0.00125,0.00292] | NA | (0,0.731] | (0.213,0.798] |
| **1252** | NA | NA | NA | (0.731,0.936] | NA |
| **1253** | (0.00386,0.00875] | (0.00125,0.00292] | NA | (0.731,0.936] | (0.213,0.798] |
| **1254** | (0.00179,0.00386] | (0,0.00125] | (0.46,Inf] | (0.936,1.11] | (0.213,0.798] |
| **1255** | (0.00179,0.00386] | (0.00125,0.00292] | (0.46,Inf] | (1.11,Inf] | (0.798,1.72] |
| **1256** | (0.00179,0.00386] | (0.00665,Inf] | (0.46,Inf] | (0,0.731] | (1.72,Inf] |
| **1257** | (0.00386,0.00875] | (0.00665,Inf] | NA | (1.11,Inf] | (1.72,Inf] |
| **1258** | NA | NA | NA | (0.731,0.936] | NA |
| **1259** | (0.00386,0.00875] | NA | NA | (0,0.731] | NA |
| **1260** | (0.00386,0.00875] | (0.00292,0.00665] | NA | (0,0.731] | (0.213,0.798] |
| **1261** | (0.00386,0.00875] | (0.00665,Inf] | NA | (0,0.731] | (1.72,Inf] |
| **1262** | (0.00875,Inf] | (0.00125,0.00292] | NA | (1.11,Inf] | (0,0.213] |
| **1263** | (0.00386,0.00875] | NA | NA | (0.731,0.936] | NA |
| **1264** | NA | NA | NA | (0.731,0.936] | NA |
| **1265** | (0.00179,0.00386] | (0.00665,Inf] | NA | (0,0.731] | (0.798,1.72] |
| **1266** | (0.00179,0.00386] | (0.00125,0.00292] | NA | (0.731,0.936] | (0.213,0.798] |
| **1267** | (0,0.00179] | (0,0.00125] | NA | (0,0.731] | (0.798,1.72] |
| **1268** | (0.00386,0.00875] | (0.00665,Inf] | NA | (0,0.731] | (0,0.213] |
| **1269** | (0.00386,0.00875] | (0.00292,0.00665] | NA | (0,0.731] | (0.213,0.798] |
| **1270** | (0.00875,Inf] | NA | NA | (1.11,Inf] | NA |
| **1271** | (0.00179,0.00386] | (0.00292,0.00665] | NA | (0,0.731] | (0.213,0.798] |
| **1272** | (0.00875,Inf] | (0.00665,Inf] | NA | (0.936,1.11] | (0.213,0.798] |
| **1273** | (0.00386,0.00875] | (0.00665,Inf] | NA | (0,0.731] | (0.798,1.72] |
| **1274** | (0.00386,0.00875] | (0.00665,Inf] | (0.46,Inf] | (0,0.731] | (0.213,0.798] |
| **1275** | NA | NA | NA | (0.731,0.936] | NA |
| **1276** | (0.00875,Inf] | (0.00665,Inf] | NA | (0.731,0.936] | (0.213,0.798] |
| **1277** | (0.00875,Inf] | NA | NA | (0,0.731] | NA |
| **1278** | NA | NA | NA | (1.11,Inf] | NA |
| **1279** | (0.00386,0.00875] | (0.00292,0.00665] | NA | (0,0.731] | (0.213,0.798] |
| **1280** | (0,0.00179] | (0.00665,Inf] | NA | (1.11,Inf] | (1.72,Inf] |
| **1281** | (0.00386,0.00875] | NA | NA | (0.731,0.936] | NA |
| **1282** | NA | (0,0.00125] | NA | (0.936,1.11] | (1.72,Inf] |
| **1283** | (0.00875,Inf] | (0,0.00125] | NA | (1.11,Inf] | (0,0.213] |
| **1284** | NA | NA | NA | (1.11,Inf] | NA |
| **1285** | (0.00386,0.00875] | NA | NA | (1.11,Inf] | NA |
| **1286** | NA | (0.00125,0.00292] | NA | (1.11,Inf] | (1.72,Inf] |
| **1287** | (0.00875,Inf] | (0.00665,Inf] | NA | (0.731,0.936] | (0.213,0.798] |
| **1288** | (0.00386,0.00875] | (0,0.00125] | NA | (0,0.731] | (0,0.213] |

| | | | | | |
|---|---|---|---|---|---|
| **1289** | (0.00386,0.00875] | (0.00665,Inf] | NA | (0,0.731] | (0.798,1.72] |
| **1290** | (0.00875,Inf] | (0.00292,0.00665] | NA | (0.731,0.936] | (0,0.213] |
| **1291** | (0.00386,0.00875] | NA | (0.46,Inf] | (0,0.731] | NA |
| **1292** | (0.00179,0.00386] | (0.00665,Inf] | NA | (0.731,0.936] | (1.72,Inf] |
| **1293** | (0.00386,0.00875] | NA | NA | (0,0.731] | NA |
| **1294** | (0.00875,Inf] | (0.00125,0.00292] | NA | (1.11,Inf] | (0,0.213] |
| **1295** | (0.00875,Inf] | (0.00292,0.00665] | NA | (0.936,1.11] | (0.213,0.798] |
| **1296** | NA | NA | NA | (0,0.731] | NA |
| **1297** | NA | (0.00125,0.00292] | NA | (0.936,1.11] | (1.72,Inf] |
| **1298** | (0.00875,Inf] | NA | NA | (1.11,Inf] | NA |
| **1299** | (0.00386,0.00875] | (0.00665,Inf] | NA | (0,0.731] | (0.213,0.798] |
| **1300** | (0.00875,Inf] | NA | NA | (0,0.731] | NA |
| **1301** | NA | NA | NA | (0.731,0.936] | NA |
| **1302** | (0.00386,0.00875] | (0.00292,0.00665] | NA | (0.731,0.936] | (0.213,0.798] |
| **1303** | (0.00875,Inf] | (0.00665,Inf] | (0.46,Inf] | (1.11,Inf] | (0.798,1.72] |
| **1304** | NA | NA | NA | (0,0.731] | NA |
| **1305** | NA | (0.00125,0.00292] | NA | (0,0.731] | (1.72,Inf] |
| **1306** | (0,0.00179] | NA | NA | (0.936,1.11] | NA |
| **1307** | NA | NA | NA | (0.936,1.11] | NA |
| **1308** | (0.00386,0.00875] | (0.00292,0.00665] | NA | (1.11,Inf] | (0.798,1.72] |
| **1309** | NA | NA | NA | (0,0.731] | NA |
| **1310** | (0.00386,0.00875] | (0.00292,0.00665] | NA | (1.11,Inf] | (0.798,1.72] |
| **1311** | (0.00875,Inf] | (0.00665,Inf] | NA | (0.731,0.936] | (1.72,Inf] |
| **1312** | (0.00875,Inf] | (0.00665,Inf] | NA | (0.731,0.936] | (0.798,1.72] |
| **1313** | (0.00875,Inf] | (0.00665,Inf] | NA | (0.731,0.936] | (0.213,0.798] |
| **1314** | (0.00386,0.00875] | (0.00292,0.00665] | NA | (1.11,Inf] | (0.798,1.72] |
| **1315** | NA | NA | NA | (1.11,Inf] | NA |
| **1316** | (0.00875,Inf] | (0.00665,Inf] | NA | (0,0.731] | (0.213,0.798] |
| **1317** | (0.00386,0.00875] | (0.00125,0.00292] | NA | (0.731,0.936] | (0,0.213] |
| **1318** | NA | NA | NA | (0,0.731] | NA |
| **1319** | (0.00386,0.00875] | NA | NA | (0,0.731] | NA |
| **1320** | NA | NA | NA | (0.936,1.11] | NA |
| **1321** | NA | NA | NA | (0,0.731] | NA |
| **1322** | NA | NA | NA | (0,0.731] | NA |
| **1323** | NA | NA | NA | (0,0.731] | NA |
| **1324** | NA | NA | NA | (0.936,1.11] | NA |
| **1325** | (0.00875,Inf] | NA | NA | (1.11,Inf] | NA |
| **1326** | NA | (0.00125,0.00292] | NA | (0,0.731] | (1.72,Inf] |

| | | | | | |
|---|---|---|---|---|---|
| **1327** | (0.00875,Inf] | NA | NA | (1.11,Inf] | NA |
| **1328** | (0.00386,0.00875] | NA | NA | (0.731,0.936] | NA |
| **1329** | NA | NA | (0.46,Inf] | (1.11,Inf] | NA |
| **1330** | NA | NA | (0.46,Inf] | NA | NA |
| **1331** | NA | NA | NA | (0.731,0.936] | NA |
| **1332** | NA | NA | NA | (0.731,0.936] | NA |
| **1333** | (0.00386,0.00875] | (0.00665,Inf] | NA | (0,0.731] | (0.798,1.72] |
| **1334** | NA | NA | NA | (0,0.731] | NA |
| **1335** | NA | (0.00665,Inf] | NA | (1.11,Inf] | (1.72,Inf] |
| **1336** | (0.00875,Inf] | (0.00665,Inf] | NA | (0,0.731] | (0.213,0.798] |
| **1337** | (0.00875,Inf] | NA | NA | (0.731,0.936] | NA |
| **1338** | (0.00875,Inf] | (0.00665,Inf] | NA | (1.11,Inf] | (0.798,1.72] |
| **1339** | (0,0.00179] | (0,0.00125] | NA | (0,0.731] | (0.798,1.72] |
| **1340** | (0.00179,0.00386] | (0.00665,Inf] | (0.46,Inf] | (0,0.731] | (0.798,1.72] |
| **1341** | NA | NA | NA | (1.11,Inf] | NA |
| **1342** | (0.00179,0.00386] | NA | NA | (1.11,Inf] | NA |
| **1343** | NA | NA | NA | (0.731,0.936] | NA |
| **1344** | (0.00386,0.00875] | NA | NA | (0,0.731] | NA |
| **1345** | (0,0.00179] | (0.00125,0.00292] | NA | (0.731,0.936] | (0.213,0.798] |
| **1346** | NA | NA | NA | (0,0.731] | NA |
| **1347** | NA | NA | NA | (1.11,Inf] | NA |
| **1348** | (0,0.00179] | (0.00665,Inf] | NA | (0,0.731] | (1.72,Inf] |
| **1349** | (0.00875,Inf] | (0.00665,Inf] | NA | (1.11,Inf] | (0.798,1.72] |
| **1350** | NA | (0.00665,Inf] | NA | (1.11,Inf] | (1.72,Inf] |
| **1351** | (0.00386,0.00875] | (0.00292,0.00665] | (0.46,Inf] | (0,0.731] | (0.213,0.798] |
| **1352** | (0.00875,Inf] | (0.00665,Inf] | NA | (1.11,Inf] | (0.798,1.72] |
| **1353** | (0.00386,0.00875] | (0.00292,0.00665] | NA | (0,0.731] | (0.213,0.798] |
| **1354** | (0.00875,Inf] | (0.00665,Inf] | NA | (0.936,1.11] | (0.798,1.72] |
| **1355** | NA | NA | (0.46,Inf] | (0.936,1.11] | NA |
| **1356** | (0.00875,Inf] | NA | NA | (1.11,Inf] | NA |
| **1357** | (0.00875,Inf] | (0.00125,0.00292] | NA | (0.731,0.936] | (0,0.213] |
| **1358** | NA | NA | NA | (0,0.731] | NA |
| **1359** | (0.00386,0.00875] | NA | NA | (1.11,Inf] | NA |
| **1360** | (0.00386,0.00875] | (0,0.00125] | NA | (0.731,0.936] | (0.213,0.798] |
| **1361** | (0.00179,0.00386] | NA | NA | (1.11,Inf] | NA |
| **1362** | (0.00875,Inf] | (0.00125,0.00292] | (0.46,Inf] | (0.731,0.936] | (0,0.213] |
| **1363** | (0.00386,0.00875] | (0.00292,0.00665] | NA | (0.936,1.11] | (0.213,0.798] |
| **1364** | (0.00875,Inf] | (0.00292,0.00665] | NA | (0.731,0.936] | (0.213,0.798] |

| | | | | | |
|---|---|---|---|---|---|
| **1365** | NA | NA | NA | (0,0.731] | NA |
| **1366** | NA | NA | NA | (0,0.731] | NA |
| **1367** | (0.00875,Inf] | (0.00292,0.00665] | NA | (1.11,Inf] | (0.213,0.798] |
| **1368** | (0.00875,Inf] | (0.00665,Inf] | NA | (0.936,1.11] | (0.798,1.72] |
| **1369** | (0.00875,Inf] | (0.00665,Inf] | NA | (1.11,Inf] | (1.72,Inf] |
| **1370** | (0.00875,Inf] | (0.00125,0.00292] | NA | (1.11,Inf] | (0.213,0.798] |
| **1371** | (0.00875,Inf] | (0.00665,Inf] | NA | (0,0.731] | (0.213,0.798] |
| **1372** | (0.00875,Inf] | (0.00665,Inf] | NA | (0,0.731] | (0.213,0.798] |
| **1373** | NA | NA | NA | (0.731,0.936] | NA |
| **1374** | NA | NA | NA | (1.11,Inf] | NA |
| **1375** | (0.00875,Inf] | NA | NA | (0,0.731] | NA |
| **1376** | (0.00386,0.00875] | (0.00665,Inf] | (0,0.46] | (1.11,Inf] | (0.798,1.72] |
| **1377** | NA | NA | NA | (0,0.731] | NA |
| **1378** | NA | NA | NA | (0,0.731] | NA |
| **1379** | (0.00875,Inf] | NA | (0.46,Inf] | (0,0.731] | NA |
| **1380** | (0.00386,0.00875] | (0.00125,0.00292] | (0.46,Inf] | (0,0.731] | (0,0.213] |
| **1381** | (0.00386,0.00875] | NA | NA | (1.11,Inf] | NA |
| **1382** | (0.00386,0.00875] | (0.00665,Inf] | NA | (0.936,1.11] | (1.72,Inf] |
| **1383** | NA | NA | (0.46,Inf] | (0,0.731] | NA |
| **1384** | (0.00386,0.00875] | NA | (0.46,Inf] | (0,0.731] | NA |
| **1385** | (0.00386,0.00875] | (0,0.00125] | NA | (0.936,1.11] | (0,0.213] |
| **1386** | (0.00875,Inf] | NA | NA | (0,0.731] | NA |
| **1387** | (0.00875,Inf] | (0.00292,0.00665] | NA | (0.731,0.936] | (0,0.213] |
| **1388** | NA | (0.00292,0.00665] | NA | (0,0.731] | (1.72,Inf] |
| **1389** | NA | NA | NA | (0.731,0.936] | NA |
| **1390** | (0.00386,0.00875] | (0.00292,0.00665] | NA | (0.936,1.11] | (0.213,0.798] |
| **1391** | (0.00179,0.00386] | (0.00292,0.00665] | NA | (0,0.731] | (0.798,1.72] |
| **1392** | (0.00875,Inf] | NA | (0.46,Inf] | (1.11,Inf] | NA |
| **1393** | (0.00386,0.00875] | (0.00665,Inf] | NA | (1.11,Inf] | (0.798,1.72] |
| **1394** | (0.00386,0.00875] | (0.00665,Inf] | (0.46,Inf] | (0,0.731] | (0.798,1.72] |
| **1395** | (0,0.00179] | NA | (0.46,Inf] | (0.936,1.11] | NA |
| **1396** | (0.00875,Inf] | (0.00125,0.00292] | NA | (1.11,Inf] | (0.213,0.798] |
| **1397** | (0.00875,Inf] | (0.00665,Inf] | NA | (0,0.731] | (0.213,0.798] |
| **1398** | NA | NA | NA | (0,0.731] | NA |
| **1399** | (0.00386,0.00875] | (0.00292,0.00665] | NA | (0.936,1.11] | (0.798,1.72] |
| **1400** | (0.00875,Inf] | (0.00665,Inf] | (0.46,Inf] | (0,0.731] | (0.213,0.798] |
| **1401** | (0,0.00179] | (0.00125,0.00292] | (0.46,Inf] | (0,0.731] | (1.72,Inf] |
| **1402** | NA | (0.00665,Inf] | NA | (0.731,0.936] | (1.72,Inf] |

| | | | | | |
|---|---|---|---|---|---|
| **1403** | (0.00386,0.00875] | (0.00665,Inf] | NA | (0,0.731] | (0.213,0.798] |
| **1404** | (0.00875,Inf] | (0.00292,0.00665] | NA | (0.936,1.11] | (0.213,0.798] |
| **1405** | (0.00386,0.00875] | (0.00292,0.00665] | NA | (0.731,0.936] | (0.213,0.798] |
| **1406** | (0.00179,0.00386] | (0.00292,0.00665] | NA | (0.731,0.936] | (0.798,1.72] |
| **1407** | (0.00875,Inf] | (0.00125,0.00292] | NA | (1.11,Inf] | (0,0.213] |
| **1408** | (0.00875,Inf] | NA | NA | (1.11,Inf] | NA |
| **1409** | (0.00179,0.00386] | (0.00665,Inf] | NA | (0,0.731] | (1.72,Inf] |
| **1410** | (0.00386,0.00875] | (0.00665,Inf] | NA | (0,0.731] | (0.798,1.72] |
| **1411** | NA | NA | NA | (0,0.731] | NA |
| **1412** | NA | NA | NA | (0.731,0.936] | NA |
| **1413** | (0.00875,Inf] | (0.00292,0.00665] | (0,0.46] | (1.11,Inf] | (0.213,0.798] |
| **1414** | NA | NA | NA | (0,0.731] | NA |
| **1415** | (0.00875,Inf] | (0.00665,Inf] | NA | (1.11,Inf] | (0.213,0.798] |
| **1416** | (0.00875,Inf] | NA | NA | (1.11,Inf] | NA |
| **1417** | (0.00875,Inf] | (0.00665,Inf] | NA | (1.11,Inf] | (0.213,0.798] |
| **1418** | (0.00875,Inf] | (0.00125,0.00292] | NA | (0,0.731] | (0.213,0.798] |
| **1419** | NA | NA | NA | (0,0.731] | NA |
| **1420** | (0.00179,0.00386] | (0.00665,Inf] | NA | (0.731,0.936] | (1.72,Inf] |
| **1421** | (0.00875,Inf] | NA | NA | (1.11,Inf] | NA |
| **1422** | NA | (0,0.00125] | NA | (0.936,1.11] | (1.72,Inf] |
| **1423** | (0.00875,Inf] | NA | NA | (0.936,1.11] | NA |
| **1424** | NA | NA | NA | NA | (0.213,0.798] |
| **1425** | (0.00179,0.00386] | (0.00125,0.00292] | NA | (0.936,1.11] | (0.213,0.798] |
| **1426** | NA | NA | NA | (0.731,0.936] | NA |
| **1427** | NA | NA | NA | (0,0.731] | NA |
| **1428** | NA | NA | (0.46,Inf] | (0.936,1.11] | NA |
| **1429** | NA | NA | NA | (1.11,Inf] | NA |
| **1430** | NA | NA | NA | (0.731,0.936] | NA |
| **1431** | (0.00875,Inf] | NA | NA | (0.936,1.11] | NA |
| **1432** | (0.00875,Inf] | NA | NA | (0.731,0.936] | NA |
| **1433** | NA | NA | NA | (1.11,Inf] | NA |
| **1434** | NA | (0.00125,0.00292] | NA | (1.11,Inf] | (1.72,Inf] |
| **1435** | NA | NA | NA | (0,0.731] | NA |
| **1436** | (0.00386,0.00875] | (0.00292,0.00665] | NA | (0,0.731] | (0.213,0.798] |
| **1437** | (0,0.00179] | (0.00292,0.00665] | NA | (0,0.731] | (1.72,Inf] |
| **1438** | (0.00179,0.00386] | (0.00665,Inf] | NA | (0,0.731] | (1.72,Inf] |
| **1439** | NA | NA | NA | (0,0.731] | NA |
| **1440** | NA | (0.00665,Inf] | NA | (0.731,0.936] | (1.72,Inf] |

| | | | | | |
|---|---|---|---|---|---|
| **1441** | (0.00875,Inf] | NA | NA | (0.731,0.936] | NA |
| **1442** | (0.00875,Inf] | (0.00292,0.00665] | NA | (1.11,Inf] | (0,0.213] |
| **1443** | NA | (0.00292,0.00665] | NA | (0,0.731] | (1.72,Inf] |
| **1444** | (0.00875,Inf] | (0.00125,0.00292] | NA | (0.731,0.936] | (0,0.213] |
| **1445** | NA | NA | NA | (0,0.731] | NA |
| **1446** | (0.00386,0.00875] | NA | NA | (0,0.731] | NA |
| **1447** | (0.00875,Inf] | NA | (0.46,Inf] | (0,0.731] | NA |
| **1448** | (0.00875,Inf] | (0.00665,Inf] | (0,0.46] | (0.731,0.936] | (0.213,0.798] |
| **1449** | (0.00179,0.00386] | NA | NA | (0.731,0.936] | NA |
| **1450** | NA | NA | NA | (0.731,0.936] | NA |
| **1451** | NA | NA | NA | (0.731,0.936] | NA |
| **1452** | (0.00386,0.00875] | (0,0.00125] | NA | (0.936,1.11] | (0,0.213] |
| **1453** | (0.00875,Inf] | NA | NA | (0,0.731] | NA |
| **1454** | (0.00875,Inf] | (0.00292,0.00665] | NA | (0.936,1.11] | (0,0.213] |
| **1455** | NA | (0.00292,0.00665] | NA | (0.936,1.11] | (1.72,Inf] |
| **1456** | NA | (0.00292,0.00665] | NA | (0,0.731] | (1.72,Inf] |
| **1457** | NA | NA | NA | (0,0.731] | NA |
| **1458** | NA | NA | NA | (1.11,Inf] | NA |
| **1459** | (0.00386,0.00875] | (0,0.00125] | NA | (1.11,Inf] | (0,0.213] |
| **1460** | (0.00875,Inf] | (0.00665,Inf] | NA | (0.731,0.936] | (0.213,0.798] |
| **1461** | (0.00875,Inf] | (0.00665,Inf] | NA | (0,0.731] | (0,0.213] |
| **1462** | NA | NA | NA | (0,0.731] | NA |
| **1463** | (0.00875,Inf] | (0.00292,0.00665] | NA | (1.11,Inf] | (0.213,0.798] |
| **1464** | (0.00875,Inf] | (0.00665,Inf] | NA | (0.731,0.936] | (0.213,0.798] |
| **1465** | (0.00875,Inf] | NA | NA | (1.11,Inf] | NA |
| **1466** | (0.00179,0.00386] | (0.00665,Inf] | NA | (0,0.731] | (0.798,1.72] |
| **1467** | (0.00875,Inf] | (0.00292,0.00665] | NA | (0.936,1.11] | (0.213,0.798] |
| **1468** | NA | NA | NA | (0.731,0.936] | NA |
| **1469** | (0.00875,Inf] | (0.00665,Inf] | (0.46,Inf] | (1.11,Inf] | (0.798,1.72] |
| **1470** | (0.00386,0.00875] | (0.00292,0.00665] | NA | (0.731,0.936] | (0.213,0.798] |
| **1471** | (0.00386,0.00875] | (0.00665,Inf] | NA | (0,0.731] | (0.798,1.72] |
| **1472** | NA | NA | NA | (1.11,Inf] | NA |
| **1473** | NA | NA | (0.46,Inf] | NA | NA |
| **1474** | NA | NA | NA | (0.731,0.936] | NA |
| **1475** | (0,0.00179] | (0.00665,Inf] | NA | (0,0.731] | (1.72,Inf] |
| **1476** | NA | (0.00292,0.00665] | NA | (0.936,1.11] | (1.72,Inf] |
| **1477** | NA | NA | NA | (0,0.731] | NA |
| **1478** | (0.00875,Inf] | NA | NA | (1.11,Inf] | NA |

| | | | | | |
|---|---|---|---|---|---|
| **1479** | (0.00386,0.00875] | (0.00665,Inf] | NA | (0,0.731] | (1.72,Inf] |
| **1480** | (0.00875,Inf] | (0.00665,Inf] | NA | (0.731,0.936] | (0.213,0.798] |
| **1481** | (0.00875,Inf] | (0.00665,Inf] | NA | (0,0.731] | (0.798,1.72] |
| **1482** | NA | NA | NA | (0.936,1.11] | NA |
| **1483** | NA | (0.00292,0.00665] | NA | (0,0.731] | (1.72,Inf] |
| **1484** | (0.00386,0.00875] | (0.00665,Inf] | NA | (1.11,Inf] | (0.798,1.72] |
| **1485** | (0.00875,Inf] | NA | NA | (0.936,1.11] | NA |
| **1486** | (0.00875,Inf] | (0.00665,Inf] | NA | (1.11,Inf] | (0.798,1.72] |
| **1487** | NA | NA | NA | (0.731,0.936] | NA |
| **1488** | (0.00875,Inf] | (0.00665,Inf] | NA | (0.936,1.11] | (0.798,1.72] |
| **1489** | (0.00875,Inf] | (0.00665,Inf] | NA | (1.11,Inf] | (0.798,1.72] |
| **1490** | (0.00875,Inf] | (0.00665,Inf] | (0,0.46] | (0,0.731] | (0.798,1.72] |
| **1491** | (0.00875,Inf] | (0.00665,Inf] | NA | (1.11,Inf] | (0.798,1.72] |
| **1492** | NA | (0.00292,0.00665] | NA | (1.11,Inf] | (1.72,Inf] |
| **1493** | (0.00179,0.00386] | (0.00292,0.00665] | NA | (0.936,1.11] | (0.798,1.72] |
| **1494** | NA | NA | NA | (1.11,Inf] | NA |
| **1495** | (0,0.00179] | (0.00292,0.00665] | NA | (1.11,Inf] | (1.72,Inf] |
| **1496** | (0.00179,0.00386] | (0.00665,Inf] | NA | (0.731,0.936] | (1.72,Inf] |
| **1497** | NA | (0.00665,Inf] | NA | (0,0.731] | (1.72,Inf] |
| **1498** | (0.00875,Inf] | (0.00292,0.00665] | NA | (1.11,Inf] | (0.213,0.798] |
| **1499** | (0.00875,Inf] | (0.00665,Inf] | NA | (1.11,Inf] | (0.213,0.798] |
| **1500** | (0.00875,Inf] | (0.00665,Inf] | NA | (0,0.731] | (0.213,0.798] |
| **1501** | (0.00875,Inf] | (0.00665,Inf] | (0.46,Inf] | (0,0.731] | (1.72,Inf] |
| **1502** | (0.00875,Inf] | (0.00125,0.00292] | NA | (1.11,Inf] | (0.213,0.798] |
| **1503** | (0.00875,Inf] | (0.00665,Inf] | (0.46,Inf] | (1.11,Inf] | (0.213,0.798] |
| **1504** | (0.00386,0.00875] | (0.00125,0.00292] | NA | (0.936,1.11] | (0.213,0.798] |
| **1505** | (0.00875,Inf] | NA | NA | (0.936,1.11] | NA |
| **1506** | NA | NA | NA | (1.11,Inf] | NA |
| **1507** | (0.00875,Inf] | (0.00125,0.00292] | NA | (1.11,Inf] | (0.213,0.798] |
| **1508** | (0.00386,0.00875] | (0.00665,Inf] | NA | (0.731,0.936] | (1.72,Inf] |
| **1509** | NA | NA | NA | (0,0.731] | NA |
| **1510** | (0.00179,0.00386] | (0.00125,0.00292] | NA | (1.11,Inf] | (0.798,1.72] |
| **1511** | (0.00875,Inf] | NA | NA | (0.936,1.11] | NA |
| **1512** | (0.00875,Inf] | (0.00665,Inf] | NA | (1.11,Inf] | (0.798,1.72] |
| **1513** | (0.00179,0.00386] | (0.00292,0.00665] | NA | (1.11,Inf] | (0.798,1.72] |
| **1514** | NA | (0.00292,0.00665] | NA | (0.731,0.936] | (1.72,Inf] |
| **1515** | NA | NA | NA | (0.731,0.936] | NA |
| **1516** | (0.00875,Inf] | (0,0.00125] | NA | (1.11,Inf] | (0,0.213] |

| | | | | | |
|---|---|---|---|---|---|
| **1517** | (0.00386,0.00875] | (0.00665,Inf] | NA | (0,0.731] | (0.213,0.798] |
| **1518** | NA | (0.00665,Inf] | NA | (0,0.731] | (1.72,Inf] |
| **1519** | NA | NA | NA | (0.731,0.936] | NA |
| **1520** | (0.00386,0.00875] | NA | NA | (1.11,Inf] | NA |
| **1521** | (0.00179,0.00386] | (0.00292,0.00665] | (0.46,Inf] | (0.731,0.936] | (0.798,1.72] |
| **1522** | (0.00875,Inf] | NA | NA | (0,0.731] | NA |
| **1523** | (0.00875,Inf] | NA | NA | (1.11,Inf] | NA |
| **1524** | NA | NA | NA | (0.936,1.11] | NA |
| **1525** | NA | (0.00665,Inf] | NA | (1.11,Inf] | (1.72,Inf] |
| **1526** | (0.00875,Inf] | (0.00292,0.00665] | NA | (1.11,Inf] | (0,0.213] |
| **1527** | NA | NA | NA | (0.731,0.936] | NA |
| **1528** | (0.00875,Inf] | (0.00665,Inf] | NA | (0.936,1.11] | (0.213,0.798] |
| **1529** | (0.00875,Inf] | (0.00292,0.00665] | NA | (0,0.731] | (0,0.213] |
| **1530** | NA | NA | NA | NA | NA |
| **1531** | NA | NA | NA | (0.731,0.936] | NA |
| **1532** | NA | NA | NA | (0.731,0.936] | NA |
| **1533** | NA | NA | NA | (0,0.731] | NA |
| **1534** | (0.00386,0.00875] | (0.00292,0.00665] | NA | (0,0.731] | (0.213,0.798] |
| **1535** | NA | NA | NA | (0.936,1.11] | NA |
| **1536** | (0.00875,Inf] | (0.00665,Inf] | NA | (0,0.731] | (0.798,1.72] |
| **1537** | (0.00875,Inf] | (0.00665,Inf] | NA | (0.936,1.11] | (0.213,0.798] |
| **1538** | NA | NA | NA | (1.11,Inf] | NA |
| **1539** | NA | NA | NA | (1.11,Inf] | NA |
| **1540** | (0.00179,0.00386] | (0.00665,Inf] | NA | (0.731,0.936] | (1.72,Inf] |
| **1541** | NA | (0.00292,0.00665] | NA | (0,0.731] | (1.72,Inf] |
| **1542** | NA | NA | NA | (0,0.731] | NA |
| **1543** | (0.00875,Inf] | NA | NA | (0.731,0.936] | NA |
| **1544** | (0.00875,Inf] | (0.00665,Inf] | NA | (0.731,0.936] | (0.213,0.798] |
| **1545** | (0.00179,0.00386] | (0.00665,Inf] | NA | (0,0.731] | (1.72,Inf] |
| **1546** | NA | NA | (0.46,Inf] | (0,0.731] | NA |
| **1547** | (0.00386,0.00875] | (0.00665,Inf] | (0.46,Inf] | (0,0.731] | (1.72,Inf] |
| **1548** | (0.00386,0.00875] | (0.00292,0.00665] | NA | (0,0.731] | (0.213,0.798] |
| **1549** | NA | (0.00665,Inf] | NA | (1.11,Inf] | (1.72,Inf] |
| **1550** | NA | NA | NA | (0.731,0.936] | NA |
| **1551** | NA | NA | NA | (1.11,Inf] | NA |
| **1552** | (0.00875,Inf] | (0.00665,Inf] | NA | (1.11,Inf] | (1.72,Inf] |
| **1553** | NA | NA | NA | (0.731,0.936] | NA |
| **1554** | (0.00875,Inf] | (0.00665,Inf] | NA | (0,0.731] | (0.213,0.798] |

| | | | | | |
|---|---|---|---|---|---|
| **1555** | (0.00179,0.00386] | NA | NA | (0,0.731] | NA |
| **1556** | (0.00875,Inf] | (0.00665,Inf] | NA | (0,0.731] | (0.213,0.798] |
| **1557** | NA | NA | NA | NA | (1.72,Inf] |
| **1558** | (0.00386,0.00875] | (0.00665,Inf] | NA | (0,0.731] | (0.798,1.72] |
| **1559** | NA | NA | NA | (1.11,Inf] | NA |
| **1560** | NA | NA | NA | (0.731,0.936] | NA |
| **1561** | NA | NA | NA | (0.731,0.936] | NA |
| **1562** | NA | NA | NA | (0,0.731] | NA |
| **1563** | NA | NA | NA | (0.936,1.11] | NA |
| **1564** | NA | NA | NA | (0,0.731] | NA |
| **1565** | NA | NA | NA | (0.731,0.936] | NA |
| **1566** | NA | NA | (0.46,Inf] | NA | (0.213,0.798] |
| **1567** | (0,0.00179] | NA | NA | (0,0.731] | NA |
| **1568** | NA | (0.00665,Inf] | NA | (0,0.731] | (1.72,Inf] |
| **1569** | (0.00875,Inf] | (0.00665,Inf] | NA | (0.731,0.936] | (0.213,0.798] |
| **1570** | NA | NA | NA | (0,0.731] | NA |
| **1571** | NA | NA | NA | (0.731,0.936] | NA |
| **1572** | (0,0.00179] | NA | NA | (0,0.731] | NA |
| **1573** | NA | NA | NA | (0,0.731] | NA |
| **1574** | NA | NA | NA | (0,0.731] | NA |
| **1575** | NA | NA | NA | (0.936,1.11] | NA |
| **1576** | NA | NA | NA | (1.11,Inf] | NA |
| **1577** | (0.00875,Inf] | NA | NA | (0.731,0.936] | NA |
| **1578** | NA | NA | (0.46,Inf] | (1.11,Inf] | NA |
| **1579** | (0.00875,Inf] | (0.00125,0.00292] | (0.46,Inf] | (0.936,1.11] | (0,0.213] |
| **1580** | NA | NA | NA | (0,0.731] | NA |
| **1581** | NA | NA | NA | (0,0.731] | NA |
| **1582** | (0.00875,Inf] | (0.00665,Inf] | (0.46,Inf] | (0,0.731] | (0.213,0.798] |
| **1583** | (0.00875,Inf] | (0.00665,Inf] | NA | (0.936,1.11] | (0.213,0.798] |
| **1584** | (0.00875,Inf] | (0.00292,0.00665] | NA | (0,0.731] | (0.213,0.798] |
| **1585** | (0.00179,0.00386] | (0.00292,0.00665] | NA | (0,0.731] | (0.798,1.72] |
| **1586** | (0.00179,0.00386] | (0.00665,Inf] | NA | (0.936,1.11] | (1.72,Inf] |
| **1587** | NA | NA | NA | (0,0.731] | NA |
| **1588** | NA | NA | NA | (0,0.731] | NA |
| **1589** | (0.00875,Inf] | NA | NA | (1.11,Inf] | NA |
| **1590** | NA | NA | NA | (0.731,0.936] | NA |
| **1591** | NA | NA | NA | (0.731,0.936] | NA |
| **1592** | NA | (0.00125,0.00292] | NA | (1.11,Inf] | (1.72,Inf] |

| | | | | | |
|---|---|---|---|---|---|
| **1593** | NA | NA | NA | (1.11,Inf] | NA |
| **1594** | (0.00875,Inf] | NA | (0.46,Inf] | (0.731,0.936] | NA |
| **1595** | (0.00875,Inf] | (0.00665,Inf] | NA | (0,0.731] | (0.798,1.72] |
| **1596** | NA | (0.00125,0.00292] | NA | (1.11,Inf] | (1.72,Inf] |
| **1597** | (0.00875,Inf] | (0.00665,Inf] | NA | (0,0.731] | (0.213,0.798] |
| **1598** | (0.00875,Inf] | (0.00665,Inf] | NA | (0,0.731] | (0.798,1.72] |
| **1599** | (0.00875,Inf] | (0.00665,Inf] | NA | (1.11,Inf] | (1.72,Inf] |
| **1600** | NA | NA | NA | (0,0.731] | NA |
| **1601** | NA | NA | NA | (0,0.731] | NA |
| **1602** | (0.00875,Inf] | (0.00665,Inf] | (0.46,Inf] | (0.936,1.11] | (0.213,0.798] |
| **1603** | (0.00875,Inf] | NA | NA | (0,0.731] | NA |
| **1604** | NA | (0.00125,0.00292] | NA | (1.11,Inf] | (1.72,Inf] |
| **1605** | (0.00386,0.00875] | NA | NA | (1.11,Inf] | NA |
| **1606** | NA | NA | NA | (0,0.731] | NA |
| **1607** | NA | (0.00292,0.00665] | NA | (0,0.731] | (1.72,Inf] |
| **1608** | NA | NA | NA | (0,0.731] | NA |
| **1609** | NA | NA | NA | (1.11,Inf] | NA |
| **1610** | (0.00875,Inf] | (0.00665,Inf] | NA | (0,0.731] | (0.798,1.72] |
| **1611** | NA | NA | NA | (0.936,1.11] | NA |
| **1612** | (0.00386,0.00875] | (0.00665,Inf] | NA | (0,0.731] | (1.72,Inf] |
| **1613** | NA | (0.00665,Inf] | NA | (0,0.731] | (1.72,Inf] |
| **1614** | NA | NA | NA | (0,0.731] | NA |
| **1615** | (0.00875,Inf] | NA | NA | (0,0.731] | NA |
| **1616** | NA | (0.00665,Inf] | NA | (0.731,0.936] | (1.72,Inf] |
| **1617** | (0.00875,Inf] | (0.00665,Inf] | NA | (0,0.731] | (0.798,1.72] |
| **1618** | (0.00386,0.00875] | (0.00125,0.00292] | NA | (1.11,Inf] | (0.213,0.798] |
| **1619** | NA | NA | NA | (0.936,1.11] | NA |
| **1620** | (0.00386,0.00875] | (0.00665,Inf] | NA | (1.11,Inf] | (1.72,Inf] |
| **1621** | (0.00875,Inf] | (0.00125,0.00292] | NA | (1.11,Inf] | (0.213,0.798] |
| **1622** | (0.00875,Inf] | (0.00665,Inf] | (0.46,Inf] | (0,0.731] | (0.213,0.798] |
| **1623** | NA | (0.00665,Inf] | NA | (0.936,1.11] | (1.72,Inf] |
| **1624** | NA | NA | (0.46,Inf] | (1.11,Inf] | NA |
| **1625** | NA | NA | NA | (1.11,Inf] | NA |
| **1626** | NA | NA | NA | (1.11,Inf] | NA |
| **1627** | NA | NA | NA | (0,0.731] | NA |
| **1628** | NA | NA | NA | (0.936,1.11] | NA |
| **1629** | (0.00875,Inf] | (0.00665,Inf] | NA | (0,0.731] | (0.798,1.72] |
| **1630** | NA | NA | NA | (0.936,1.11] | NA |

| | | | | | |
|---|---|---|---|---|---|
| **1631** | (0.00875,Inf] | (0.00665,Inf] | NA | (0,0.731] | (0.213,0.798] |
| **1632** | (0.00875,Inf] | NA | NA | (0,0.731] | NA |
| **1633** | (0.00875,Inf] | NA | NA | (0,0.731] | NA |
| **1634** | NA | NA | NA | (0,0.731] | NA |
| **1635** | (0.00875,Inf] | NA | (0.46,Inf] | (1.11,Inf] | NA |
| **1636** | NA | NA | NA | (1.11,Inf] | NA |
| **1637** | NA | (0.00665,Inf] | NA | (0,0.731] | (1.72,Inf] |
| **1638** | NA | NA | NA | (1.11,Inf] | NA |
| **1639** | (0.00179,0.00386] | (0.00665,Inf] | NA | (0.731,0.936] | (1.72,Inf] |
| **1640** | NA | NA | NA | (1.11,Inf] | NA |
| **1641** | (0.00386,0.00875] | (0.00665,Inf] | NA | (0,0.731] | (0.213,0.798] |
| **1642** | NA | (0.00665,Inf] | NA | (0,0.731] | (1.72,Inf] |
| **1643** | (0.00875,Inf] | NA | NA | (0,0.731] | NA |
| **1644** | (0.00875,Inf] | NA | (0.46,Inf] | (1.11,Inf] | NA |
| **1645** | (0.00875,Inf] | (0.00125,0.00292] | NA | (0,0.731] | (0,0.213] |
| **1646** | (0.00875,Inf] | (0.00665,Inf] | (0.46,Inf] | (1.11,Inf] | (1.72,Inf] |
| **1647** | NA | NA | NA | (0,0.731] | NA |
| **1648** | NA | NA | NA | (0,0.731] | NA |
| **1649** | (0.00875,Inf] | (0.00665,Inf] | NA | (1.11,Inf] | (0.213,0.798] |
| **1650** | NA | NA | NA | (0.731,0.936] | NA |
| **1651** | (0.00386,0.00875] | (0.00292,0.00665] | NA | (0,0.731] | (0.213,0.798] |
| **1652** | (0.00875,Inf] | (0.00665,Inf] | NA | (0,0.731] | (0.213,0.798] |
| **1653** | NA | NA | NA | (0.731,0.936] | NA |
| **1654** | (0.00386,0.00875] | NA | NA | (0.936,1.11] | NA |
| **1655** | NA | NA | NA | (1.11,Inf] | NA |
| **1656** | (0.00875,Inf] | (0.00665,Inf] | NA | (1.11,Inf] | (0.798,1.72] |
| **1657** | NA | NA | NA | (0,0.731] | NA |
| **1658** | (0.00386,0.00875] | NA | NA | (1.11,Inf] | NA |
| **1659** | (0.00875,Inf] | (0.00665,Inf] | NA | (0.731,0.936] | (0.213,0.798] |
| **1660** | (0.00875,Inf] | (0.00292,0.00665] | (0.46,Inf] | (0.936,1.11] | (0.213,0.798] |
| **1661** | NA | (0.00665,Inf] | NA | (1.11,Inf] | (1.72,Inf] |
| **1662** | (0.00875,Inf] | NA | NA | (0,0.731] | NA |
| **1663** | NA | (0.00125,0.00292] | NA | (0.731,0.936] | (1.72,Inf] |
| **1664** | (0.00875,Inf] | (0.00665,Inf] | NA | (0,0.731] | (0.798,1.72] |
| **1665** | (0.00875,Inf] | NA | NA | (1.11,Inf] | NA |
| **1666** | NA | NA | NA | (0,0.731] | NA |
| **1667** | (0.00386,0.00875] | (0.00665,Inf] | NA | (1.11,Inf] | (1.72,Inf] |
| **1668** | (0.00179,0.00386] | (0.00665,Inf] | NA | (0.936,1.11] | (1.72,Inf] |

| | | | | | |
|---|---|---|---|---|---|
| **1669** | NA | NA | NA | (1.11,Inf] | NA |
| **1670** | NA | NA | NA | (0,0.731] | NA |
| **1671** | (0.00386,0.00875] | NA | NA | (0,0.731] | NA |
| **1672** | (0.00875,Inf] | (0.00665,Inf] | NA | (0.731,0.936] | (0,0.213] |
| **1673** | (0.00875,Inf] | (0.00665,Inf] | NA | (1.11,Inf] | (0.798,1.72] |
| **1674** | NA | NA | NA | (0,0.731] | NA |
| **1675** | NA | (0.00665,Inf] | NA | (0.731,0.936] | (1.72,Inf] |
| **1676** | NA | NA | NA | (1.11,Inf] | NA |
| **1677** | (0.00386,0.00875] | (0.00292,0.00665] | NA | (0.936,1.11] | (0.213,0.798] |
| **1678** | NA | NA | NA | (0,0.731] | NA |
| **1679** | (0.00875,Inf] | NA | NA | (0.731,0.936] | NA |
| **1680** | NA | NA | NA | (0,0.731] | NA |
| **1681** | NA | NA | NA | (0,0.731] | NA |
| **1682** | NA | (0.00125,0.00292] | NA | (1.11,Inf] | (1.72,Inf] |
| **1683** | (0.00875,Inf] | NA | NA | (0.936,1.11] | NA |
| **1684** | (0,0.00179] | (0.00665,Inf] | NA | (0.731,0.936] | (1.72,Inf] |
| **1685** | (0.00875,Inf] | (0.00665,Inf] | NA | (0,0.731] | (0.213,0.798] |
| **1686** | NA | (0.00665,Inf] | NA | (0,0.731] | (1.72,Inf] |
| **1687** | NA | NA | NA | (0,0.731] | NA |
| **1688** | NA | NA | (0.46,Inf] | (0,0.731] | NA |
| **1689** | NA | NA | NA | (0,0.731] | NA |
| **1690** | (0.00875,Inf] | NA | NA | (1.11,Inf] | NA |
| **1691** | (0.00875,Inf] | (0.00665,Inf] | NA | (0,0.731] | (0.798,1.72] |
| **1692** | (0.00386,0.00875] | (0.00292,0.00665] | NA | (0,0.731] | (0.213,0.798] |
| **1693** | NA | (0.00665,Inf] | NA | (0.731,0.936] | (1.72,Inf] |
| **1694** | (0.00875,Inf] | (0.00665,Inf] | (0.46,Inf] | (0,0.731] | (1.72,Inf] |
| **1695** | (0.00875,Inf] | (0.00665,Inf] | (0.46,Inf] | (1.11,Inf] | (1.72,Inf] |
| **1696** | NA | (0.00292,0.00665] | (0.46,Inf] | (0.731,0.936] | (1.72,Inf] |
| **1697** | NA | NA | NA | (1.11,Inf] | NA |
| **1698** | NA | (0.00665,Inf] | NA | (0,0.731] | (1.72,Inf] |
| **1699** | (0.00875,Inf] | (0.00665,Inf] | NA | (0,0.731] | (0.213,0.798] |
| **1700** | (0.00179,0.00386] | (0.00292,0.00665] | NA | (1.11,Inf] | (1.72,Inf] |
| **1701** | (0.00875,Inf] | NA | NA | (0,0.731] | NA |
| **1702** | NA | NA | NA | (0,0.731] | NA |
| **1703** | NA | NA | NA | (0.731,0.936] | NA |
| **1704** | (0.00386,0.00875] | (0.00665,Inf] | NA | (0,0.731] | (1.72,Inf] |
| **1705** | (0.00875,Inf] | (0.00665,Inf] | NA | (1.11,Inf] | (0.213,0.798] |
| **1706** | NA | (0.00125,0.00292] | NA | (1.11,Inf] | (1.72,Inf] |

| | | | | | |
|---|---|---|---|---|---|
| **1707** | (0.00875,Inf] | (0.00292,0.00665] | NA | (1.11,Inf] | (0.213,0.798] |
| **1708** | (0.00875,Inf] | (0.00292,0.00665] | NA | (1.11,Inf] | (0,0.213] |
| **1709** | (0.00386,0.00875] | NA | NA | (0,0.731] | NA |
| **1710** | (0.00875,Inf] | (0.00665,Inf] | (0.46,Inf] | (1.11,Inf] | (0.213,0.798] |
| **1711** | NA | NA | (0.46,Inf] | (0,0.731] | NA |
| **1712** | (0.00875,Inf] | (0.00665,Inf] | NA | (0,0.731] | (0.213,0.798] |
| **1713** | (0.00875,Inf] | (0.00665,Inf] | NA | (1.11,Inf] | (0.798,1.72] |
| **1714** | NA | NA | NA | (0.936,1.11] | NA |
| **1715** | NA | NA | NA | (1.11,Inf] | NA |
| **1716** | (0.00875,Inf] | NA | NA | (0,0.731] | NA |
| **1717** | NA | (0.00665,Inf] | NA | (0,0.731] | (1.72,Inf] |
| **1718** | (0.00875,Inf] | NA | NA | (1.11,Inf] | NA |
| **1719** | NA | NA | NA | (0,0.731] | NA |
| **1720** | NA | NA | NA | (1.11,Inf] | NA |
| **1721** | NA | NA | (0,0.46] | NA | (0.798,1.72] |
| **1722** | (0.00875,Inf] | (0.00665,Inf] | NA | (0,0.731] | (0.213,0.798] |
| **1723** | (0.00875,Inf] | (0.00665,Inf] | NA | (1.11,Inf] | (0.213,0.798] |
| **1724** | (0.00386,0.00875] | NA | NA | (0,0.731] | NA |
| **1725** | (0.00875,Inf] | (0.00125,0.00292] | NA | (1.11,Inf] | (0,0.213] |
| **1726** | NA | NA | NA | (1.11,Inf] | NA |
| **1727** | (0.00179,0.00386] | (0.00665,Inf] | (0.46,Inf] | (0.936,1.11] | (1.72,Inf] |
| **1728** | NA | NA | NA | NA | (0.213,0.798] |
| **1729** | NA | NA | NA | (0,0.731] | NA |
| **1730** | (0.00386,0.00875] | NA | NA | (0,0.731] | NA |
| **1731** | NA | NA | (0.46,Inf] | NA | NA |
| **1732** | NA | NA | (0.46,Inf] | NA | NA |
| **1733** | NA | NA | NA | (1.11,Inf] | NA |
| **1734** | (0.00386,0.00875] | (0.00292,0.00665] | NA | (0.731,0.936] | (0.213,0.798] |
| **1735** | (0.00875,Inf] | (0.00665,Inf] | NA | (0.731,0.936] | (0.798,1.72] |
| **1736** | NA | NA | (0.46,Inf] | (1.11,Inf] | NA |
| **1737** | NA | NA | NA | (0.936,1.11] | NA |
| **1738** | (0.00875,Inf] | (0.00665,Inf] | NA | (1.11,Inf] | (0.213,0.798] |
| **1739** | (0.00875,Inf] | NA | NA | (0,0.731] | NA |
| **1740** | (0.00875,Inf] | (0.00665,Inf] | NA | (1.11,Inf] | (0.213,0.798] |
| **1741** | (0.00875,Inf] | NA | NA | (1.11,Inf] | NA |
| **1742** | (0.00875,Inf] | (0.00665,Inf] | NA | (0,0.731] | (0,0.213] |
| **1743** | NA | NA | NA | (0,0.731] | NA |
| **1744** | (0.00875,Inf] | NA | NA | (1.11,Inf] | NA |

| | | | | | |
|---|---|---|---|---|---|
| **1745** | NA | NA | NA | (0,0.731] | NA |
| **1746** | NA | NA | NA | (0.731,0.936] | NA |
| **1747** | NA | NA | NA | (0,0.731] | NA |
| **1748** | NA | NA | NA | (0,0.731] | NA |
| **1749** | (0.00875,Inf] | (0.00125,0.00292] | NA | (1.11,Inf] | (0.213,0.798] |
| **1750** | (0.00875,Inf] | NA | NA | (1.11,Inf] | NA |
| **1751** | NA | (0.00665,Inf] | NA | (0.731,0.936] | (1.72,Inf] |
| **1752** | NA | (0.00665,Inf] | NA | (0,0.731] | (1.72,Inf] |
| **1753** | NA | (0.00665,Inf] | NA | (0,0.731] | (1.72,Inf] |
| **1754** | (0.00875,Inf] | NA | NA | (1.11,Inf] | NA |
| **1755** | NA | NA | NA | (0,0.731] | NA |
| **1756** | (0.00875,Inf] | (0.00125,0.00292] | NA | (1.11,Inf] | (0,0.213] |
| **1757** | NA | NA | NA | (0.936,1.11] | NA |
| **1758** | NA | NA | NA | (0,0.731] | NA |
| **1759** | NA | NA | NA | (0,0.731] | NA |
| **1760** | (0.00179,0.00386] | (0.00665,Inf] | (0,0.46] | (0.731,0.936] | (1.72,Inf] |
| **1761** | NA | NA | NA | (0,0.731] | NA |
| **1762** | NA | NA | NA | (0.936,1.11] | NA |
| **1763** | (0.00875,Inf] | NA | NA | (0,0.731] | NA |
| **1764** | NA | NA | NA | (0.936,1.11] | NA |
| **1765** | (0.00875,Inf] | NA | (0.46,Inf] | (0,0.731] | NA |
| **1766** | (0.00875,Inf] | (0.00125,0.00292] | NA | (1.11,Inf] | (0,0.213] |
| **1767** | NA | NA | NA | (1.11,Inf] | NA |
| **1768** | NA | NA | NA | (0.936,1.11] | NA |
| **1769** | NA | NA | NA | (0,0.731] | NA |
| **1770** | (0.00875,Inf] | NA | NA | (1.11,Inf] | NA |
| **1771** | NA | NA | NA | (0,0.731] | NA |
| **1772** | (0.00875,Inf] | (0.00665,Inf] | (0.46,Inf] | (1.11,Inf] | (0.213,0.798] |
| **1773** | (0.00875,Inf] | (0.00665,Inf] | (0,0.46] | (1.11,Inf] | (0.213,0.798] |
| **1774** | NA | NA | NA | (0,0.731] | NA |
| **1775** | NA | NA | NA | (0.731,0.936] | NA |
| **1776** | NA | NA | NA | (1.11,Inf] | NA |
| **1777** | (0.00386,0.00875] | NA | NA | (0,0.731] | NA |
| **1778** | NA | NA | NA | (0,0.731] | NA |
| **1779** | (0.00875,Inf] | NA | NA | (0,0.731] | NA |
| **1780** | (0.00875,Inf] | (0.00665,Inf] | NA | (0.936,1.11] | (0.213,0.798] |
| **1781** | NA | NA | NA | NA | (0.798,1.72] |
| **1782** | NA | NA | NA | (0,0.731] | NA |

| | | | | | |
|---|---|---|---|---|---|
| **1783** | NA | NA | NA | (0,0.731] | NA |
| **1784** | (0.00875,Inf] | (0.00125,0.00292] | NA | (1.11,Inf] | (0,0.213] |
| **1785** | NA | (0.00665,Inf] | NA | (0.936,1.11] | (1.72,Inf] |
| **1786** | (0.00875,Inf] | (0.00665,Inf] | (0.46,Inf] | (0,0.731] | (0.213,0.798] |
| **1787** | NA | NA | (0.46,Inf] | (0,0.731] | NA |
| **1788** | (0.00386,0.00875] | (0.00665,Inf] | (0,0.46] | (1.11,Inf] | (1.72,Inf] |
| **1789** | (0.00875,Inf] | (0.00665,Inf] | NA | (0,0.731] | (1.72,Inf] |
| **1790** | NA | NA | NA | (0,0.731] | NA |
| **1791** | (0.00875,Inf] | (0.00665,Inf] | NA | (1.11,Inf] | (1.72,Inf] |
| **1792** | NA | NA | NA | (1.11,Inf] | NA |
| **1793** | NA | NA | (0.46,Inf] | NA | NA |
| **1794** | NA | NA | (0.46,Inf] | NA | NA |
| **1795** | NA | (0.00665,Inf] | NA | (1.11,Inf] | (1.72,Inf] |
| **1796** | NA | NA | NA | (1.11,Inf] | NA |
| **1797** | NA | NA | NA | (0.731,0.936] | NA |
| **1798** | (0.00875,Inf] | (0.00665,Inf] | NA | (1.11,Inf] | (0.213,0.798] |
| **1799** | (0.00875,Inf] | NA | NA | (1.11,Inf] | NA |
| **1800** | NA | NA | NA | (0,0.731] | NA |
| **1801** | NA | NA | NA | (1.11,Inf] | NA |
| **1802** | NA | (0.00665,Inf] | NA | (0.731,0.936] | (1.72,Inf] |
| **1803** | NA | NA | NA | (1.11,Inf] | NA |
| **1804** | NA | (0.00125,0.00292] | NA | (1.11,Inf] | (1.72,Inf] |
| **1805** | (0.00875,Inf] | (0.00665,Inf] | (0,0.46] | (0,0.731] | (0.798,1.72] |
| **1806** | (0,0.00179] | NA | NA | (1.11,Inf] | NA |
| **1807** | NA | NA | NA | (0.731,0.936] | NA |
| **1808** | (0,0.00179] | NA | (0.46,Inf] | (0,0.731] | NA |
| **1809** | NA | NA | NA | (1.11,Inf] | NA |
| **1810** | (0.00875,Inf] | NA | NA | (0.936,1.11] | NA |
| **1811** | (0.00875,Inf] | (0.00665,Inf] | NA | (0,0.731] | (0,0.213] |
| **1812** | NA | NA | NA | (0.936,1.11] | NA |
| **1813** | NA | NA | NA | NA | NA |
| **1814** | (0.00386,0.00875] | NA | NA | (0,0.731] | NA |
| **1815** | NA | NA | (0.46,Inf] | NA | NA |
| **1816** | NA | NA | NA | (0.731,0.936] | NA |
| **1817** | NA | NA | NA | (0,0.731] | NA |
| **1818** | NA | NA | NA | (0,0.731] | NA |
| **1819** | NA | NA | NA | (0,0.731] | NA |
| **1820** | NA | (0.00665,Inf] | NA | (0,0.731] | (1.72,Inf] |

| | | | | | |
|---|---|---|---|---|---|
| **1821** | NA | NA | NA | (0,0.731] | NA |
| **1822** | NA | NA | NA | (1.11,Inf] | NA |
| **1823** | (0.00875,Inf] | NA | NA | (1.11,Inf] | NA |
| **1824** | (0.00875,Inf] | (0.00292,0.00665] | NA | (1.11,Inf] | (0.798,1.72] |
| **1825** | NA | NA | NA | (1.11,Inf] | NA |
| **1826** | (0.00875,Inf] | (0.00665,Inf] | NA | (0.936,1.11] | (0.798,1.72] |
| **1827** | NA | (0.00665,Inf] | NA | (0,0.731] | (1.72,Inf] |
| **1828** | (0.00875,Inf] | NA | NA | (0,0.731] | NA |
| **1829** | NA | NA | NA | (1.11,Inf] | NA |
| **1830** | (0.00875,Inf] | (0.00665,Inf] | NA | (0,0.731] | (0.798,1.72] |
| **1831** | NA | NA | NA | (0,0.731] | NA |
| **1832** | NA | NA | NA | (0.936,1.11] | NA |
| **1833** | NA | NA | NA | (0,0.731] | NA |
| **1834** | (0.00875,Inf] | (0.00665,Inf] | NA | (0,0.731] | (0.213,0.798] |
| **1835** | (0.00875,Inf] | (0.00665,Inf] | NA | (1.11,Inf] | (0.213,0.798] |
| **1836** | NA | NA | NA | (0.731,0.936] | NA |
| **1837** | NA | (0.00665,Inf] | NA | (0.731,0.936] | (1.72,Inf] |
| **1838** | NA | NA | NA | (0,0.731] | NA |
| **1839** | NA | NA | NA | (1.11,Inf] | NA |
| **1840** | (0.00875,Inf] | (0.00665,Inf] | NA | (0,0.731] | (0.798,1.72] |
| **1841** | NA | (0.00665,Inf] | (0.46,Inf] | (0,0.731] | (1.72,Inf] |
| **1842** | (0.00875,Inf] | NA | NA | (0,0.731] | NA |
| **1843** | NA | NA | (0.46,Inf] | NA | NA |
| **1844** | NA | NA | NA | (0,0.731] | NA |
| **1845** | NA | NA | NA | (0,0.731] | NA |
| **1846** | (0.00386,0.00875] | (0.00665,Inf] | NA | NA | (0.213,0.798] |
| **1847** | NA | NA | NA | (1.11,Inf] | NA |
| **1848** | NA | NA | NA | NA | NA |
| **1849** | NA | (0.00665,Inf] | NA | (0,0.731] | (1.72,Inf] |
| **1850** | NA | NA | NA | (0.731,0.936] | NA |
| **1851** | NA | NA | NA | (1.11,Inf] | NA |
| **1852** | NA | NA | NA | (1.11,Inf] | NA |
| **1853** | (0.00386,0.00875] | (0.00665,Inf] | NA | (0,0.731] | (1.72,Inf] |
| **1854** | (0.00875,Inf] | NA | NA | (1.11,Inf] | NA |
| **1855** | NA | NA | (0.46,Inf] | (0,0.731] | NA |
| **1856** | NA | NA | NA | (0,0.731] | NA |
| **1857** | NA | (0.00665,Inf] | NA | (0,0.731] | (1.72,Inf] |
| **1858** | (0.00875,Inf] | (0.00665,Inf] | NA | (1.11,Inf] | (1.72,Inf] |

| | | | | | |
|---|---|---|---|---|---|
| **1859** | (0.00386,0.00875] | (0.00665,Inf] | NA | (0,0.731] | (0.798,1.72] |
| **1860** | NA | NA | NA | (0,0.731] | NA |
| **1861** | NA | NA | NA | (1.11,Inf] | NA |
| **1862** | NA | NA | NA | NA | NA |
| **1863** | NA | NA | NA | (1.11,Inf] | NA |
| **1864** | (0.00875,Inf] | (0.00665,Inf] | NA | NA | (1.72,Inf] |
| **1865** | (0.00875,Inf] | NA | (0.46,Inf] | (0,0.731] | NA |
| **1866** | (0.00875,Inf] | NA | NA | (0,0.731] | NA |
| **1867** | NA | NA | NA | NA | (0.798,1.72] |
| **1868** | (0.00386,0.00875] | (0.00665,Inf] | NA | (1.11,Inf] | (1.72,Inf] |
| **1869** | NA | (0.00665,Inf] | NA | (0,0.731] | (1.72,Inf] |
| **1870** | (0.00875,Inf] | NA | NA | (0,0.731] | NA |
| **1871** | NA | NA | NA | (0,0.731] | NA |
| **1872** | (0.00875,Inf] | (0.00665,Inf] | NA | (0.936,1.11] | (0.213,0.798] |
| **1873** | (0.00875,Inf] | NA | NA | (0,0.731] | NA |
| **1874** | NA | (0.00665,Inf] | NA | (1.11,Inf] | (1.72,Inf] |
| **1875** | (0.00875,Inf] | (0.00665,Inf] | NA | (1.11,Inf] | (1.72,Inf] |
| **1876** | NA | NA | NA | (0.731,0.936] | NA |
| **1877** | NA | NA | NA | (1.11,Inf] | NA |
| **1878** | NA | NA | NA | (0.731,0.936] | NA |
| **1879** | (0.00386,0.00875] | NA | NA | (1.11,Inf] | NA |
| **1880** | NA | NA | NA | (0,0.731] | NA |
| **1881** | NA | NA | NA | NA | NA |
| **1882** | NA | (0.00665,Inf] | NA | (0,0.731] | (1.72,Inf] |
| **1883** | NA | NA | (0.46,Inf] | (0,0.731] | NA |
| **1884** | (0.00875,Inf] | (0.00665,Inf] | NA | (1.11,Inf] | (0.213,0.798] |
| **1885** | (0.00875,Inf] | NA | NA | (1.11,Inf] | NA |
| **1886** | NA | NA | NA | (1.11,Inf] | NA |
| **1887** | NA | NA | NA | (0,0.731] | NA |
| **1888** | NA | NA | NA | (1.11,Inf] | NA |
| **1889** | (0.00875,Inf] | (0.00665,Inf] | NA | (0.936,1.11] | (1.72,Inf] |
| **1890** | (0.00875,Inf] | (0.00665,Inf] | NA | (1.11,Inf] | (0.798,1.72] |
| **1891** | NA | NA | NA | (0,0.731] | NA |
| **1892** | NA | NA | NA | (1.11,Inf] | NA |
| **1893** | (0.00875,Inf] | NA | NA | (0.731,0.936] | NA |
| **1894** | NA | NA | NA | (0,0.731] | NA |
| **1895** | (0.00875,Inf] | NA | NA | (0.731,0.936] | NA |
| **1896** | NA | NA | NA | (0,0.731] | NA |

| | | | | | |
|---|---|---|---|---|---|
| **1897** | NA | NA | NA | (0,0.731] | NA |
| **1898** | NA | NA | (0.46,Inf] | NA | (0.798,1.72] |
| **1899** | (0.00875,Inf] | (0.00665,Inf] | NA | (0,0.731] | (0,0.213] |
| **1900** | NA | NA | NA | NA | NA |
| **1901** | NA | NA | NA | (0,0.731] | NA |
| **1902** | NA | NA | NA | (0,0.731] | NA |
| **1903** | (0.00875,Inf] | (0.00665,Inf] | NA | (0,0.731] | (1.72,Inf] |
| **1904** | NA | (0.00665,Inf] | NA | (0,0.731] | (1.72,Inf] |
| **1905** | (0.00875,Inf] | NA | NA | (0,0.731] | NA |
| **1906** | NA | NA | NA | (0,0.731] | NA |
| **1907** | NA | NA | NA | (0.731,0.936] | NA |
| **1908** | NA | NA | (0.46,Inf] | NA | NA |
| **1909** | NA | NA | NA | (0,0.731] | NA |
| **1910** | NA | NA | NA | (0.731,0.936] | NA |
| **1911** | NA | (0.00665,Inf] | NA | (0,0.731] | (1.72,Inf] |
| **1912** | (0.00875,Inf] | (0.00665,Inf] | NA | (1.11,Inf] | (0.798,1.72] |
| **1913** | (0.00875,Inf] | NA | NA | (1.11,Inf] | NA |
| **1914** | (0.00875,Inf] | (0.00665,Inf] | (0.46,Inf] | (0,0.731] | (0.213,0.798] |
| **1915** | NA | (0.00665,Inf] | (0.46,Inf] | NA | (1.72,Inf] |
| **1916** | (0.00875,Inf] | (0.00665,Inf] | NA | (1.11,Inf] | (0.213,0.798] |
| **1917** | NA | NA | (0.46,Inf] | (0.936,1.11] | NA |
| **1918** | NA | (0.00665,Inf] | NA | NA | (1.72,Inf] |
| **1919** | NA | NA | NA | (0,0.731] | NA |
| **1920** | (0.00875,Inf] | (0.00665,Inf] | NA | NA | (0.798,1.72] |
| **1921** | (0.00875,Inf] | (0.00665,Inf] | (0.46,Inf] | NA | (0.213,0.798] |
| **1922** | NA | NA | (0.46,Inf] | (1.11,Inf] | NA |
| **1923** | NA | NA | NA | NA | NA |
| **1924** | (0.00875,Inf] | NA | (0.46,Inf] | (1.11,Inf] | NA |
| **1925** | NA | (0.00665,Inf] | NA | (0.731,0.936] | (1.72,Inf] |
| **1926** | (0.00875,Inf] | (0.00665,Inf] | NA | (1.11,Inf] | (0,0.213] |
| **1927** | NA | NA | (0.46,Inf] | NA | NA |
| **1928** | NA | NA | NA | (0,0.731] | NA |
| **1929** | (0.00875,Inf] | NA | NA | (1.11,Inf] | NA |
| **1930** | NA | NA | NA | (0,0.731] | NA |
| **1931** | (0.00386,0.00875] | NA | NA | (0,0.731] | NA |
| **1932** | (0.00875,Inf] | NA | NA | (1.11,Inf] | NA |
| **1933** | NA | NA | (0.46,Inf] | NA | NA |
| **1934** | (0.00875,Inf] | (0.00665,Inf] | NA | (1.11,Inf] | (0.798,1.72] |

| | | | | | |
|---|---|---|---|---|---|
| **1935** | (0.00875,Inf] | NA | NA | (1.11,Inf] | NA |
| **1936** | (0.00875,Inf] | NA | NA | (0.731,0.936] | NA |
| **1937** | (0.00179,0.00386] | (0.00665,Inf] | (0.46,Inf] | (0,0.731] | (1.72,Inf] |
| **1938** | (0.00875,Inf] | (0.00665,Inf] | NA | (0.936,1.11] | (0.798,1.72] |
| **1939** | NA | NA | NA | (0,0.731] | NA |
| **1940** | NA | NA | NA | (0,0.731] | NA |
| **1941** | (0.00875,Inf] | NA | NA | (1.11,Inf] | NA |
| **1942** | NA | NA | NA | (1.11,Inf] | NA |
| **1943** | NA | NA | NA | (0,0.731] | NA |
| **1944** | NA | NA | NA | NA | NA |
| **1945** | NA | NA | NA | (0,0.731] | NA |
| **1946** | NA | NA | (0.46,Inf] | NA | NA |
| **1947** | NA | NA | NA | NA | NA |
| **1948** | NA | NA | NA | (0,0.731] | NA |
| **1949** | NA | (0.00665,Inf] | NA | (0.936,1.11] | (1.72,Inf] |
| **1950** | NA | NA | (0.46,Inf] | NA | NA |
| **1951** | (0.00875,Inf] | (0.00665,Inf] | NA | NA | (1.72,Inf] |
| **1952** | NA | NA | NA | NA | NA |
| **1953** | NA | NA | NA | (1.11,Inf] | NA |
| **1954** | (0.00386,0.00875] | (0.00665,Inf] | NA | (1.11,Inf] | (1.72,Inf] |
| **1955** | NA | (0.00292,0.00665] | NA | (1.11,Inf] | (1.72,Inf] |
| **1956** | NA | NA | NA | NA | (1.72,Inf] |
| **1957** | NA | (0.00665,Inf] | NA | (1.11,Inf] | (1.72,Inf] |
| **1958** | NA | NA | NA | NA | NA |
| **1959** | NA | (0.00665,Inf] | (0,0.46] | (0,0.731] | (1.72,Inf] |
| **1960** | NA | (0.00665,Inf] | NA | NA | (1.72,Inf] |
| **1961** | NA | NA | NA | NA | NA |
| **1962** | NA | NA | NA | (0,0.731] | NA |
| **1963** | NA | NA | NA | (0.731,0.936] | NA |
| **1964** | NA | NA | NA | (0.731,0.936] | NA |
| **1965** | NA | NA | NA | (0.731,0.936] | NA |
| **1966** | NA | NA | NA | (0,0.731] | NA |
| **1967** | (0.00875,Inf] | (0.00665,Inf] | (0.46,Inf] | (1.11,Inf] | (1.72,Inf] |
| **1968** | NA | NA | NA | (0,0.731] | NA |
| **1969** | NA | NA | NA | (0,0.731] | NA |
| **1970** | (0.00875,Inf] | (0.00665,Inf] | NA | (1.11,Inf] | (1.72,Inf] |
| **1971** | NA | (0.00665,Inf] | NA | (1.11,Inf] | (1.72,Inf] |
| **1972** | NA | NA | (0.46,Inf] | NA | NA |

| | | | | | |
|---|---|---|---|---|---|
| **1973** | (0.00875,Inf] | (0.00665,Inf] | (0,0.46] | (0,0.731] | (1.72,Inf] |
| **1974** | NA | NA | NA | NA | (1.72,Inf] |
| **1975** | NA | NA | NA | NA | (1.72,Inf] |
| **1976** | NA | NA | NA | (0,0.731] | NA |
| **1977** | NA | NA | NA | NA | (0.798,1.72] |
| **1978** | NA | NA | (0.46,Inf] | NA | NA |
| **1979** | (0.00875,Inf] | NA | NA | (1.11,Inf] | NA |
| **1980** | (0.00875,Inf] | NA | NA | (1.11,Inf] | NA |
| **1981** | NA | (0.00665,Inf] | NA | (0,0.731] | (1.72,Inf] |
| **1982** | NA | NA | (0.46,Inf] | NA | NA |
| **1983** | NA | NA | NA | (1.11,Inf] | NA |
| **1984** | NA | NA | (0.46,Inf] | NA | NA |
| **1985** | NA | NA | NA | (1.11,Inf] | NA |
| **1986** | (0.00875,Inf] | (0.00665,Inf] | NA | (0.731,0.936] | (0.213,0.798] |
| **1987** | (0.00875,Inf] | (0.00665,Inf] | (0.46,Inf] | (1.11,Inf] | (0.213,0.798] |
| **1988** | NA | NA | NA | (1.11,Inf] | NA |
| **1989** | NA | NA | NA | NA | NA |
| **1990** | NA | NA | (0.46,Inf] | (1.11,Inf] | NA |
| **1991** | NA | NA | NA | NA | (0.213,0.798] |
| **1992** | NA | NA | NA | NA | NA |
| **1993** | (0.00875,Inf] | (0.00665,Inf] | (0.46,Inf] | (1.11,Inf] | (0.798,1.72] |
| **1994** | NA | NA | (0.46,Inf] | NA | (1.72,Inf] |
| **1995** | NA | NA | (0.46,Inf] | NA | NA |
| **1996** | NA | NA | (0.46,Inf] | NA | NA |
| **1997** | NA | NA | NA | NA | NA |
| **1998** | NA | NA | NA | NA | NA |
| **1999** | (0.00875,Inf] | NA | NA | NA | NA |
| **2000** | NA | NA | NA | (0,0.731] | NA |
| **2001** | NA | NA | NA | (1.11,Inf] | NA |
| **2002** | NA | NA | NA | NA | NA |
| **2003** | NA | NA | (0.46,Inf] | NA | NA |
| **2004** | (0.00875,Inf] | (0.00665,Inf] | NA | (0,0.731] | (0.213,0.798] |
| **2005** | NA | NA | (0.46,Inf] | NA | NA |
| **2006** | NA | NA | NA | NA | NA |
| **2007** | (0.00875,Inf] | NA | NA | NA | NA |
| **2008** | NA | NA | NA | NA | NA |
| **2009** | (0.00875,Inf] | NA | (0.46,Inf] | (0.936,1.11] | NA |
| **2010** | NA | NA | NA | (0,0.731] | NA |

| | | | | | |
|---|---|---|---|---|---|
| **2011** | NA | NA | NA | NA | NA |
| **2012** | NA | (0.00665,Inf] | NA | (1.11,Inf] | (1.72,Inf] |
| **2013** | NA | NA | (0.46,Inf] | NA | NA |
| **2014** | NA | NA | NA | NA | (0.213,0.798] |
| **2015** | NA | NA | NA | NA | (1.72,Inf] |
| **2016** | NA | NA | (0.46,Inf] | NA | NA |
| **2017** | (0.00875,Inf] | (0.00665,Inf] | (0.46,Inf] | (1.11,Inf] | (0.798,1.72] |
| **2018** | NA | NA | (0.46,Inf] | (1.11,Inf] | NA |
| **2019** | NA | NA | (0.46,Inf] | NA | NA |
| **2020** | NA | NA | (0.46,Inf] | NA | NA |
| **2021** | NA | NA | (0.46,Inf] | NA | NA |
| **2022** | NA | NA | NA | NA | NA |
| **2023** | NA | NA | (0.46,Inf] | (0,0.731] | NA |
| **2024** | NA | NA | NA | NA | (0.798,1.72] |
| **2025** | NA | NA | (0.46,Inf] | NA | NA |
| **2026** | NA | NA | NA | NA | NA |
| **2027** | (0.00875,Inf] | (0.00665,Inf] | NA | (1.11,Inf] | (0.798,1.72] |
| **2028** | NA | NA | (0.46,Inf] | NA | (0.798,1.72] |
| **2029** | NA | NA | NA | NA | NA |
| **2030** | NA | NA | (0.46,Inf] | NA | NA |
| **2031** | NA | NA | NA | NA | (0.213,0.798] |
| **2032** | NA | NA | NA | NA | NA |
| **2033** | NA | NA | NA | NA | (1.72,Inf] |
| **2034** | NA | NA | NA | NA | (0.213,0.798] |
| **2035** | NA | NA | (0.46,Inf] | NA | NA |
| **2036** | NA | NA | (0.46,Inf] | NA | NA |
| **2037** | NA | NA | (0.46,Inf] | NA | NA |
| **2038** | NA | NA | NA | NA | (0,0.213] |
| **2039** | NA | NA | NA | NA | (0,0.213] |
| **2040** | NA | NA | NA | NA | (0.213,0.798] |
| **2041** | NA | NA | NA | (1.11,Inf] | NA |
| **2042** | NA | NA | NA | NA | (0.213,0.798] |
| **2043** | NA | NA | (0.46,Inf] | NA | NA |
| **2044** | NA | NA | NA | (1.11,Inf] | NA |
| **2045** | NA | NA | NA | NA | (1.72,Inf] |
| **2046** | NA | NA | NA | NA | (1.72,Inf] |
| **2047** | NA | NA | (0.46,Inf] | NA | NA |
| **2048** | NA | NA | (0.46,Inf] | NA | NA |

| | | | | | |
|---|---|---|---|---|---|
| **2049** | NA | NA | (0.46,Inf] | NA | NA |
| **2050** | NA | NA | (0.46,Inf] | NA | NA |
| **2051** | NA | NA | NA | NA | NA |
| **2052** | (0.00875,Inf] | (0.00665,Inf] | NA | (1.11,Inf] | (0.213,0.798] |
| **2053** | NA | NA | (0.46,Inf] | NA | NA |
| **2054** | NA | NA | NA | (1.11,Inf] | NA |
| **2055** | NA | NA | (0.46,Inf] | NA | NA |
| **2056** | NA | NA | (0.46,Inf] | NA | NA |
| **2057** | NA | NA | NA | NA | (1.72,Inf] |
| **2058** | NA | NA | (0.46,Inf] | NA | NA |
| **2059** | NA | NA | (0.46,Inf] | NA | NA |
| **2060** | NA | NA | (0.46,Inf] | NA | NA |
| **2061** | NA | NA | (0.46,Inf] | NA | NA |
| **2062** | NA | NA | (0.46,Inf] | NA | NA |
| **2063** | NA | NA | NA | NA | NA |
| **2064** | NA | NA | NA | NA | NA |
| **2065** | NA | NA | NA | NA | (0.213,0.798] |
| **2066** | NA | NA | NA | NA | NA |
| **2067** | NA | NA | (0.46,Inf] | NA | NA |
| **2068** | NA | NA | (0.46,Inf] | NA | (0.213,0.798] |
| **2069** | NA | NA | NA | NA | (0,0.213] |
| **2070** | NA | NA | NA | NA | NA |
| **2071** | NA | NA | NA | NA | NA |
| **2072** | NA | NA | NA | NA | (0.798,1.72] |
| **2073** | NA | NA | NA | (1.11,Inf] | NA |
| **2074** | NA | NA | (0.46,Inf] | NA | NA |
| **2075** | NA | NA | (0.46,Inf] | NA | NA |
| **2076** | NA | NA | NA | NA | (1.72,Inf] |
| **2077** | NA | NA | (0.46,Inf] | NA | NA |
| **2078** | NA | NA | (0.46,Inf] | NA | NA |
| **2079** | NA | NA | (0.46,Inf] | NA | NA |
| **2080** | NA | NA | NA | NA | NA |
| **2081** | NA | NA | NA | NA | (0.798,1.72] |
| **2082** | NA | NA | NA | NA | (0.798,1.72] |
| **2083** | NA | NA | NA | NA | (0.213,0.798] |
| **2084** | NA | NA | (0.46,Inf] | NA | NA |
| **2085** | NA | NA | (0,0.46] | NA | (0.798,1.72] |
| **2086** | NA | NA | NA | NA | (0.798,1.72] |

| | | | | | |
|---|---|---|---|---|---|
| **2087** | NA | NA | NA | NA | NA |
| **2088** | NA | NA | NA | NA | (0.798,1.72] |
| **2089** | NA | NA | NA | NA | NA |
| **2090** | NA | NA | NA | NA | (0.798,1.72] |
| **2091** | NA | NA | NA | NA | (1.72,Inf] |
| **2092** | NA | NA | (0,0.46] | NA | (1.72,Inf] |
| **2093** | NA | NA | NA | NA | (0,0.213] |
| **2094** | NA | NA | NA | NA | (0.798,1.72] |
| **2095** | NA | NA | NA | NA | (1.72,Inf] |
| **2096** | NA | NA | (0.46,Inf] | NA | NA |
| **2097** | NA | NA | NA | NA | (0.213,0.798] |
| **2098** | NA | NA | (0.46,Inf] | NA | NA |
| **2099** | NA | NA | NA | NA | NA |
| **2100** | NA | NA | (0.46,Inf] | NA | (0.213,0.798] |
| **2101** | NA | NA | (0.46,Inf] | NA | (0.213,0.798] |
| **2102** | NA | NA | NA | NA | (1.72,Inf] |
| **2103** | NA | NA | (0.46,Inf] | NA | NA |
| **2104** | NA | NA | NA | NA | NA |
| **2105** | NA | NA | NA | NA | NA |
| **2106** | NA | NA | (0.46,Inf] | NA | NA |
| **2107** | NA | NA | NA | NA | (0,0.213] |
| **2108** | NA | NA | NA | NA | NA |
| **2109** | NA | NA | (0.46,Inf] | NA | NA |
| **2110** | NA | NA | (0.46,Inf] | NA | NA |
| **2111** | NA | NA | (0.46,Inf] | NA | NA |
| **2112** | NA | NA | (0.46,Inf] | NA | NA |
| **2113** | NA | NA | NA | NA | NA |
| **2114** | NA | NA | (0.46,Inf] | NA | (1.72,Inf] |
| **2115** | NA | NA | NA | NA | (1.72,Inf] |
| **2116** | NA | NA | NA | (1.11,Inf] | NA |
| **2117** | NA | NA | NA | NA | (0.213,0.798] |
| **2118** | NA | NA | (0.46,Inf] | NA | NA |
| **2119** | NA | NA | NA | NA | (0,0.213] |
| **2120** | NA | NA | (0.46,Inf] | NA | NA |
| **2121** | NA | NA | (0.46,Inf] | NA | (1.72,Inf] |
| **2122** | NA | NA | NA | NA | (0.798,1.72] |
| **2123** | NA | NA | (0.46,Inf] | NA | NA |
| **2124** | NA | NA | (0.46,Inf] | NA | NA |

| | | | | | |
|---|---|---|---|---|---|
| **2125** | NA | NA | (0.46,Inf] | NA | NA |
| **2126** | NA | NA | NA | NA | NA |
| **2127** | NA | NA | (0.46,Inf] | NA | NA |
| **2128** | NA | NA | NA | NA | NA |
| **2129** | NA | NA | NA | NA | (0.213,0.798] |
| **2130** | NA | NA | NA | NA | (0,0.213] |
| **2131** | NA | NA | NA | NA | NA |
| **2132** | NA | NA | (0.46,Inf] | NA | NA |
| **2133** | NA | NA | NA | NA | NA |
| **2134** | NA | NA | NA | NA | NA |
| **2135** | NA | NA | (0.46,Inf] | NA | NA |
| **2136** | NA | NA | NA | NA | NA |
| **2137** | NA | NA | (0.46,Inf] | NA | NA |
| **2138** | NA | NA | NA | NA | (1.72,Inf] |
| **2139** | NA | NA | NA | NA | (0.213,0.798] |
| **2140** | NA | NA | NA | NA | NA |
| **2141** | NA | NA | NA | NA | (0,0.213] |
| **2142** | NA | NA | (0.46,Inf] | NA | NA |
| **2143** | NA | NA | (0.46,Inf] | NA | NA |
| **2144** | NA | NA | NA | NA | NA |
| **2145** | NA | NA | NA | NA | (0.798,1.72] |
| **2146** | NA | NA | (0.46,Inf] | NA | NA |
| **2147** | NA | NA | NA | NA | (1.72,Inf] |
| **2148** | NA | NA | NA | NA | (0,0.213] |
| **2149** | NA | NA | NA | NA | NA |
| **2150** | NA | NA | (0.46,Inf] | NA | NA |
| **2151** | NA | NA | NA | NA | NA |
| **2152** | NA | NA | NA | NA | (1.72,Inf] |
| **2153** | NA | NA | NA | NA | NA |
| **2154** | NA | NA | NA | NA | NA |
| **2155** | NA | NA | NA | NA | NA |
| **2156** | NA | NA | NA | NA | (0.798,1.72] |
| **2157** | NA | NA | (0.46,Inf] | NA | NA |
| **2158** | NA | NA | NA | NA | (0.213,0.798] |
| **2159** | NA | NA | (0.46,Inf] | NA | NA |
| **2160** | NA | NA | NA | NA | (0.798,1.72] |
| **2161** | NA | NA | (0.46,Inf] | NA | NA |
| **2162** | NA | NA | NA | NA | (0.798,1.72] |

| | | | | | |
|---|---|---|---|---|---|
| **2163** | NA | NA | (0,0.46] | NA | (1.72,Inf] |
| **2164** | NA | NA | NA | NA | NA |
| **2165** | NA | NA | NA | NA | NA |
| **2166** | NA | NA | NA | NA | (0,0.213] |
| **2167** | NA | NA | NA | NA | (0.213,0.798] |
| **2168** | NA | NA | NA | NA | (0.213,0.798] |
| **2169** | NA | NA | NA | NA | (1.72,Inf] |
| **2170** | NA | NA | NA | NA | (0.213,0.798] |
| **2171** | NA | NA | NA | NA | NA |
| **2172** | NA | NA | (0.46,Inf] | NA | NA |
| **2173** | NA | NA | (0.46,Inf] | NA | NA |
| **2174** | NA | NA | NA | NA | (0,0.213] |
| **2175** | NA | NA | NA | NA | (0.798,1.72] |
| **2176** | NA | NA | NA | NA | NA |
| **2177** | NA | NA | (0.46,Inf] | NA | NA |
| **2178** | NA | NA | NA | NA | (0,0.213] |
| **2179** | NA | NA | NA | NA | (1.72,Inf] |
| **2180** | NA | NA | NA | NA | NA |
| **2181** | NA | NA | (0.46,Inf] | NA | NA |
| **2182** | NA | NA | NA | NA | (0,0.213] |
| **2183** | NA | NA | (0.46,Inf] | NA | NA |
| **2184** | NA | NA | (0.46,Inf] | NA | NA |
| **2185** | NA | NA | NA | NA | (0.798,1.72] |
| **2186** | NA | NA | NA | NA | NA |
| **2187** | NA | NA | NA | NA | (1.72,Inf] |
| **2188** | NA | NA | NA | NA | (0.213,0.798] |
| **2189** | NA | NA | NA | NA | NA |
| **2190** | NA | NA | (0.46,Inf] | NA | NA |
| **2191** | NA | NA | NA | NA | (1.72,Inf] |
| **2192** | NA | NA | NA | NA | (1.72,Inf] |
| **2193** | NA | NA | NA | NA | (0.213,0.798] |
| **2194** | NA | NA | NA | NA | NA |
| **2195** | NA | NA | NA | NA | (0.213,0.798] |
| **2196** | NA | NA | NA | NA | (1.72,Inf] |
| **2197** | NA | NA | NA | NA | NA |
| **2198** | NA | NA | NA | NA | NA |
| **2199** | NA | NA | NA | NA | (0.798,1.72] |
| **2200** | NA | NA | NA | NA | (0.213,0.798] |

| | | | | | |
|---|---|---|---|---|---|
| **2201** | NA | NA | NA | NA | (1.72,Inf] |
| **2202** | NA | NA | NA | NA | NA |
| **2203** | NA | NA | NA | NA | NA |
| **2204** | NA | NA | (0.46,Inf] | NA | NA |
| **2205** | NA | NA | (0,0.46] | NA | (0.798,1.72] |
| **2206** | NA | NA | NA | NA | NA |
| **2207** | NA | NA | NA | NA | (0.213,0.798] |
| **2208** | NA | NA | (0.46,Inf] | NA | NA |
| **2209** | NA | NA | (0.46,Inf] | NA | NA |
| **2210** | NA | NA | NA | NA | (1.72,Inf] |
| **2211** | NA | NA | NA | NA | (0.798,1.72] |
| **2212** | NA | NA | NA | NA | (1.72,Inf] |
| **2213** | NA | NA | NA | NA | NA |
| **2214** | NA | NA | NA | NA | NA |
| **2215** | NA | NA | (0.46,Inf] | NA | NA |
| **2216** | NA | NA | (0.46,Inf] | NA | NA |
| **2217** | NA | NA | NA | NA | (0.798,1.72] |
| **2218** | NA | NA | (0.46,Inf] | NA | (0.213,0.798] |
| **2219** | NA | NA | (0.46,Inf] | NA | NA |
| **2220** | NA | NA | NA | NA | NA |
| **2221** | NA | NA | NA | NA | (0.213,0.798] |
| **2222** | NA | NA | (0.46,Inf] | NA | NA |
| **2223** | NA | NA | NA | NA | NA |
| **2224** | NA | NA | (0.46,Inf] | NA | NA |
| **2225** | NA | NA | NA | NA | NA |
| **2226** | NA | NA | NA | NA | NA |
| **2227** | NA | NA | NA | NA | (0.798,1.72] |
| **2228** | NA | NA | NA | NA | (0,0.213] |
| **2229** | NA | NA | NA | NA | (0,0.213] |
| **2230** | NA | NA | NA | NA | NA |
| **2231** | NA | NA | (0.46,Inf] | NA | (0,0.213] |
| **2232** | NA | NA | NA | NA | NA |
| **2233** | NA | NA | NA | NA | (0.213,0.798] |
| **2234** | NA | NA | NA | NA | (0.213,0.798] |
| **2235** | NA | NA | (0,0.46] | NA | (1.72,Inf] |
| **2236** | NA | NA | NA | NA | (0.798,1.72] |
| **2237** | NA | NA | NA | NA | (1.72,Inf] |
| **2238** | NA | NA | NA | NA | (0.213,0.798] |

| | | | | | |
|---|---|---|---|---|---|
| **2239** | NA | NA | NA | NA | NA |
| **2240** | NA | NA | NA | NA | NA |
| **2241** | NA | NA | NA | NA | (1.72,Inf] |
| **2242** | NA | NA | NA | NA | (1.72,Inf] |
| **2243** | NA | NA | NA | NA | (1.72,Inf] |
| **2244** | NA | NA | NA | NA | (1.72,Inf] |
| **2245** | NA | NA | NA | NA | (0,0.213] |
| **2246** | NA | NA | NA | NA | (0.213,0.798] |
| **2247** | NA | NA | (0.46,Inf] | NA | (0.213,0.798] |
| **2248** | NA | NA | NA | NA | (0.213,0.798] |
| **2249** | NA | NA | NA | NA | NA |
| **2250** | NA | NA | NA | NA | (0,0.213] |
| **2251** | NA | NA | NA | NA | NA |
| **2252** | NA | NA | NA | NA | (0.798,1.72] |
| **2253** | NA | NA | NA | NA | (1.72,Inf] |
| **2254** | NA | NA | NA | NA | (1.72,Inf] |
| **2255** | NA | NA | NA | NA | NA |
| **2256** | NA | NA | (0.46,Inf] | NA | (0.213,0.798] |
| **2257** | NA | NA | NA | NA | (0,0.213] |
| **2258** | NA | NA | NA | NA | NA |
| **2259** | NA | NA | NA | NA | NA |
| **2260** | NA | NA | NA | NA | NA |
| **2261** | NA | NA | NA | NA | (1.72,Inf] |
| **2262** | NA | NA | (0.46,Inf] | NA | NA |
| **2263** | NA | NA | NA | NA | (0.213,0.798] |
| **2264** | NA | NA | NA | NA | NA |
| **2265** | NA | NA | NA | NA | (0.213,0.798] |
| **2266** | NA | NA | (0.46,Inf] | NA | NA |
| **2267** | NA | NA | (0.46,Inf] | NA | NA |
| **2268** | NA | NA | NA | NA | NA |
| **2269** | NA | NA | NA | NA | NA |
| **2270** | NA | NA | NA | NA | NA |
| **2271** | NA | NA | (0.46,Inf] | NA | NA |
| **2272** | NA | NA | (0.46,Inf] | NA | NA |
| **2273** | NA | NA | NA | NA | NA |
| **2274** | NA | NA | NA | NA | (1.72,Inf] |
| **2275** | NA | NA | NA | NA | (0.213,0.798] |
| **2276** | NA | NA | (0.46,Inf] | NA | NA |

| | | | | | |
|---|---|---|---|---|---|
| **2277** | NA | NA | NA | NA | (0,0.213] |
| **2278** | NA | NA | NA | NA | (0.213,0.798] |
| **2279** | NA | NA | NA | NA | NA |
| **2280** | NA | NA | (0.46,Inf] | NA | (1.72,Inf] |
| **2281** | NA | NA | NA | NA | (1.72,Inf] |
| **2282** | NA | NA | NA | NA | NA |
| **2283** | NA | NA | (0.46,Inf] | NA | NA |
| **2284** | NA | NA | NA | NA | (1.72,Inf] |
| **2285** | NA | NA | (0.46,Inf] | NA | NA |
| **2286** | NA | NA | NA | NA | (0.213,0.798] |
| **2287** | NA | NA | NA | NA | NA |
| **2288** | NA | NA | (0.46,Inf] | NA | NA |
| **2289** | NA | NA | NA | NA | (1.72,Inf] |
| **2290** | NA | NA | NA | NA | (1.72,Inf] |
| **2291** | NA | NA | NA | NA | (0.213,0.798] |
| **2292** | NA | NA | (0.46,Inf] | NA | NA |
| **2293** | NA | NA | NA | NA | NA |
| **2294** | NA | NA | NA | NA | NA |
| **2295** | NA | NA | NA | NA | (0.213,0.798] |
| **2296** | NA | NA | (0.46,Inf] | NA | NA |
| **2297** | NA | NA | NA | NA | (1.72,Inf] |
| **2298** | NA | NA | NA | NA | NA |
| **2299** | NA | NA | NA | NA | (1.72,Inf] |
| **2300** | NA | NA | (0.46,Inf] | NA | NA |
| **2301** | NA | NA | (0.46,Inf] | NA | NA |
| **2302** | NA | NA | NA | NA | (1.72,Inf] |
| **2303** | NA | NA | (0,0.46] | NA | NA |
| **2304** | NA | NA | (0.46,Inf] | NA | NA |
| **2305** | NA | NA | (0.46,Inf] | NA | (0.798,1.72] |
| **2306** | NA | NA | NA | NA | (1.72,Inf] |
| **2307** | NA | NA | NA | NA | (1.72,Inf] |
| **2308** | NA | NA | NA | NA | NA |
| **2309** | NA | NA | NA | NA | NA |
| **2310** | NA | NA | NA | NA | NA |
| **2311** | NA | NA | NA | NA | (0.213,0.798] |
| **2312** | NA | NA | (0.46,Inf] | NA | NA |
| **2313** | NA | NA | NA | NA | NA |
| **2314** | NA | NA | NA | NA | NA |

| | | | | | |
|---|---|---|---|---|---|
| **2315** | NA | NA | NA | NA | NA |
| **2316** | NA | NA | NA | NA | (0,0.213] |
| **2317** | NA | NA | (0.46,Inf] | NA | NA |
| **2318** | NA | NA | NA | NA | (0.798,1.72] |
| **2319** | NA | NA | NA | NA | (0.213,0.798] |
| **2320** | NA | NA | NA | NA | (0,0.213] |
| **2321** | NA | NA | NA | NA | NA |
| **2322** | NA | NA | (0.46,Inf] | NA | NA |
| **2323** | NA | NA | NA | NA | (0.213,0.798] |
| **2324** | NA | NA | (0.46,Inf] | NA | NA |
| **2325** | NA | NA | (0,0.46] | NA | NA |
| **2326** | NA | NA | NA | NA | (0.798,1.72] |
| **2327** | NA | NA | NA | NA | (0.798,1.72] |
| **2328** | NA | NA | NA | NA | (0.798,1.72] |
| **2329** | NA | NA | NA | NA | NA |
| **2330** | NA | NA | NA | NA | (1.72,Inf] |
| **2331** | NA | NA | NA | NA | (1.72,Inf] |
| **2332** | NA | NA | NA | NA | (1.72,Inf] |
| **2333** | NA | NA | NA | NA | NA |
| **2334** | NA | NA | NA | NA | (1.72,Inf] |
| **2335** | NA | NA | NA | NA | NA |
| **2336** | NA | NA | NA | NA | (1.72,Inf] |
| **2337** | NA | NA | NA | NA | NA |
| **2338** | NA | NA | NA | NA | (1.72,Inf] |
| **2339** | NA | NA | (0.46,Inf] | NA | NA |
| **2340** | NA | NA | (0.46,Inf] | NA | (0.213,0.798] |
| **2341** | NA | NA | (0.46,Inf] | NA | (0.213,0.798] |
| **2342** | NA | NA | NA | NA | NA |
| **2343** | NA | NA | NA | NA | NA |
| **2344** | NA | NA | NA | NA | NA |
| **2345** | NA | NA | (0.46,Inf] | NA | NA |
| **2346** | NA | NA | NA | NA | (0.798,1.72] |
| **2347** | NA | NA | NA | NA | (0.213,0.798] |
| **2348** | NA | NA | NA | NA | NA |
| **2349** | NA | NA | (0.46,Inf] | NA | (0,0.213] |
| **2350** | NA | NA | NA | NA | (0,0.213] |
| **2351** | NA | NA | NA | NA | (0.798,1.72] |
| **2352** | NA | NA | (0.46,Inf] | NA | NA |

| | | | | | |
|---|---|---|---|---|---|
| **2353** | NA | NA | NA | NA | NA |
| **2354** | NA | NA | (0.46,Inf] | NA | NA |
| **2355** | NA | NA | NA | NA | (1.72,Inf] |
| **2356** | NA | NA | NA | NA | (0.213,0.798] |
| **2357** | NA | NA | NA | NA | NA |
| **2358** | NA | NA | NA | NA | (0,0.213] |
| **2359** | NA | NA | NA | NA | NA |
| **2360** | NA | NA | NA | NA | (1.72,Inf] |
| **2361** | NA | NA | NA | NA | (0.213,0.798] |
| **2362** | NA | NA | (0.46,Inf] | NA | (0.213,0.798] |
| **2363** | NA | NA | NA | NA | (0.213,0.798] |
| **2364** | NA | NA | (0,0.46] | NA | (0.798,1.72] |
| **2365** | NA | NA | NA | NA | NA |
| **2366** | NA | NA | NA | NA | (1.72,Inf] |
| **2367** | NA | NA | NA | NA | NA |
| **2368** | NA | NA | NA | NA | NA |
| **2369** | NA | NA | NA | NA | (0.213,0.798] |
| **2370** | NA | NA | NA | NA | (1.72,Inf] |
| **2371** | NA | NA | NA | NA | (0,0.213] |
| **2372** | NA | NA | NA | NA | NA |
| **2373** | NA | NA | (0.46,Inf] | NA | NA |
| **2374** | NA | NA | NA | NA | NA |
| **2375** | NA | NA | NA | NA | (0.213,0.798] |
| **2376** | NA | NA | (0.46,Inf] | NA | NA |
| **2377** | NA | NA | NA | NA | NA |
| **2378** | NA | NA | NA | NA | (0.213,0.798] |
| **2379** | NA | NA | (0.46,Inf] | NA | NA |
| **2380** | NA | NA | NA | NA | (0.213,0.798] |
| **2381** | NA | NA | NA | NA | NA |
| **2382** | NA | NA | NA | NA | NA |
| **2383** | NA | NA | (0,0.46] | NA | (1.72,Inf] |
| **2384** | NA | NA | (0.46,Inf] | NA | NA |
| **2385** | NA | NA | NA | NA | NA |
| **2386** | NA | NA | NA | NA | NA |
| **2387** | NA | NA | (0.46,Inf] | NA | NA |
| **2388** | NA | NA | (0.46,Inf] | NA | NA |
| **2389** | NA | NA | NA | NA | NA |
| **2390** | NA | NA | NA | NA | NA |

| | | | | | |
|---|---|---|---|---|---|
| **2391** | NA | NA | NA | NA | (1.72,Inf] |
| **2392** | NA | NA | NA | NA | (0.213,0.798] |
| **2393** | NA | NA | NA | NA | NA |
| **2394** | NA | NA | NA | NA | NA |
| **2395** | NA | NA | NA | NA | (0,0.213] |
| **2396** | NA | NA | NA | NA | NA |
| **2397** | NA | NA | NA | NA | NA |
| **2398** | NA | NA | NA | NA | (1.72,Inf] |
| **2399** | NA | NA | (0.46,Inf] | NA | NA |
| **2400** | NA | NA | NA | NA | NA |
| **2401** | NA | NA | NA | NA | NA |
| **2402** | NA | NA | NA | NA | NA |
| **2403** | NA | NA | NA | NA | NA |
| **2404** | NA | NA | NA | NA | NA |
| **2405** | NA | NA | (0.46,Inf] | NA | (1.72,Inf] |
| **2406** | NA | NA | (0,0.46] | NA | (1.72,Inf] |
| **2407** | NA | NA | NA | NA | (1.72,Inf] |
| **2408** | NA | NA | (0.46,Inf] | NA | NA |
| **2409** | NA | NA | NA | NA | NA |
| **2410** | NA | NA | (0.46,Inf] | NA | NA |
| **2411** | NA | NA | NA | NA | NA |
| **2412** | NA | NA | NA | NA | NA |
| **2413** | NA | NA | NA | NA | (1.72,Inf] |
| **2414** | NA | NA | (0,0.46] | NA | (0,0.213] |
| **2415** | NA | NA | (0.46,Inf] | NA | NA |
| **2416** | NA | NA | NA | NA | NA |
| **2417** | NA | NA | NA | NA | (1.72,Inf] |
| **2418** | NA | NA | (0.46,Inf] | NA | (0.798,1.72] |
| **2419** | NA | NA | NA | NA | (1.72,Inf] |
| **2420** | NA | NA | NA | NA | NA |
| **2421** | NA | NA | (0.46,Inf] | NA | NA |
| **2422** | NA | NA | (0.46,Inf] | NA | (0.213,0.798] |
| **2423** | NA | NA | NA | NA | NA |
| **2424** | NA | NA | NA | NA | (1.72,Inf] |
| **2425** | NA | NA | NA | NA | (1.72,Inf] |
| **2426** | NA | NA | NA | NA | (0.213,0.798] |
| **2427** | NA | NA | NA | NA | NA |
| **2428** | NA | NA | NA | NA | NA |

| | | | | | |
|---|---|---|---|---|---|
| **2429** | NA | NA | NA | NA | NA |
| **2430** | NA | NA | NA | NA | NA |
| **2431** | NA | NA | NA | NA | NA |
| **2432** | NA | NA | (0.46,Inf] | NA | NA |
| **2433** | NA | NA | NA | NA | NA |
| **2434** | NA | NA | NA | NA | NA |
| **2435** | NA | NA | NA | NA | (1.72,Inf] |
| **2436** | NA | NA | NA | NA | NA |
| **2437** | NA | NA | NA | NA | NA |
| **2438** | NA | NA | NA | NA | NA |
| **2439** | NA | NA | NA | NA | NA |
| **2440** | NA | NA | (0.46,Inf] | NA | NA |
| **2441** | NA | NA | NA | NA | (0,0.213] |
| **2442** | NA | NA | NA | NA | (1.72,Inf] |
| **2443** | NA | NA | NA | NA | NA |
| **2444** | NA | NA | NA | NA | (1.72,Inf] |
| **2445** | NA | NA | NA | NA | (0.798,1.72] |
| **2446** | NA | NA | NA | NA | (1.72,Inf] |
| **2447** | NA | NA | NA | NA | NA |
| **2448** | NA | NA | (0,0.46] | NA | (1.72,Inf] |
| **2449** | NA | NA | NA | NA | NA |
| **2450** | NA | NA | NA | NA | (1.72,Inf] |
| **2451** | NA | NA | (0.46,Inf] | NA | NA |
| **2452** | NA | NA | NA | NA | (1.72,Inf] |
| **2453** | NA | NA | NA | NA | (1.72,Inf] |
| **2454** | NA | NA | NA | NA | NA |
| **2455** | NA | NA | NA | NA | NA |
| **2456** | NA | NA | NA | NA | (1.72,Inf] |
| **2457** | NA | NA | NA | NA | (0.213,0.798] |
| **2458** | NA | NA | NA | NA | NA |
| **2459** | NA | NA | NA | NA | NA |
| **2460** | NA | NA | NA | NA | NA |
| **2461** | NA | NA | NA | NA | NA |
| **2462** | NA | NA | (0.46,Inf] | NA | NA |
| **2463** | NA | NA | NA | NA | NA |
| **2464** | NA | NA | (0.46,Inf] | NA | NA |
| **2465** | NA | NA | (0.46,Inf] | NA | (1.72,Inf] |
| **2466** | NA | NA | NA | NA | NA |

| | | | | | |
|---|---|---|---|---|---|
| **2467** | NA | NA | (0.46,Inf] | NA | NA |
| **2468** | NA | NA | (0.46,Inf] | NA | NA |
| **2469** | NA | NA | NA | NA | NA |
| **2470** | NA | NA | (0.46,Inf] | NA | (1.72,Inf] |
| **2471** | NA | NA | NA | NA | NA |
| **2472** | NA | NA | NA | NA | NA |
| **2473** | NA | NA | NA | NA | (1.72,Inf] |
| **2474** | NA | NA | (0.46,Inf] | NA | NA |
| **2475** | NA | NA | NA | NA | NA |
| **2476** | NA | NA | NA | NA | NA |
| **2477** | NA | NA | NA | NA | NA |
| **2478** | NA | NA | NA | NA | NA |
| **2479** | NA | NA | (0.46,Inf] | NA | NA |
| **2480** | NA | NA | NA | NA | (1.72,Inf] |
| **2481** | NA | NA | NA | NA | NA |
| **2482** | NA | NA | NA | NA | (0.213,0.798] |
| **2483** | NA | NA | NA | NA | NA |
| **2484** | NA | NA | NA | NA | (1.72,Inf] |
| **2485** | NA | NA | NA | NA | (1.72,Inf] |
| **2486** | NA | NA | NA | NA | (0.798,1.72] |
| **2487** | NA | NA | NA | NA | (1.72,Inf] |
| **2488** | NA | NA | NA | NA | NA |
| **2489** | NA | NA | NA | NA | NA |
| **2490** | NA | NA | NA | NA | NA |
| **2491** | NA | NA | NA | NA | (0.213,0.798] |
| **2492** | NA | NA | NA | NA | NA |
| **2493** | NA | NA | (0.46,Inf] | NA | NA |
| **2494** | NA | NA | NA | NA | (1.72,Inf] |
| **2495** | NA | NA | NA | NA | NA |
| **2496** | NA | NA | (0.46,Inf] | NA | NA |
| **2497** | NA | NA | NA | NA | (1.72,Inf] |
| **2498** | NA | NA | (0.46,Inf] | NA | (1.72,Inf] |
| **2499** | NA | NA | NA | NA | NA |
| **2500** | NA | NA | (0.46,Inf] | NA | NA |
| **2501** | NA | NA | NA | NA | NA |
| **2502** | NA | NA | NA | NA | (1.72,Inf] |
| **2503** | NA | NA | NA | NA | (0.213,0.798] |
| **2504** | NA | NA | NA | NA | NA |

| | | | | | |
|---|---|---|---|---|---|
| **2505** | NA | NA | NA | NA | (1.72,Inf] |
| **2506** | NA | NA | NA | NA | (0.213,0.798] |
| **2507** | NA | NA | (0.46,Inf] | NA | (1.72,Inf] |
| **2508** | NA | NA | NA | NA | (0.213,0.798] |
| **2509** | NA | NA | NA | NA | (1.72,Inf] |
| **2510** | NA | NA | NA | NA | NA |
| **2511** | NA | NA | NA | NA | NA |
| **2512** | NA | NA | NA | NA | NA |
| **2513** | NA | NA | NA | NA | NA |
| **2514** | NA | NA | NA | NA | NA |
| **2515** | NA | NA | NA | NA | NA |
| **2516** | NA | NA | NA | NA | (0.213,0.798] |
| **2517** | NA | NA | (0.46,Inf] | NA | NA |
| **2518** | NA | NA | (0.46,Inf] | NA | NA |
| **2519** | NA | NA | NA | NA | NA |
| **2520** | NA | NA | (0.46,Inf] | NA | (1.72,Inf] |
| **2521** | NA | NA | NA | NA | NA |
| **2522** | NA | NA | NA | NA | NA |
| **2523** | NA | NA | NA | NA | (1.72,Inf] |
| **2524** | NA | NA | NA | NA | NA |
| **2525** | NA | NA | NA | NA | NA |
| **2526** | NA | NA | NA | NA | (0.213,0.798] |
| **2527** | NA | NA | NA | NA | (0.213,0.798] |
| **2528** | NA | NA | NA | NA | NA |
| **2529** | NA | NA | NA | NA | NA |
| **2530** | NA | NA | NA | NA | (1.72,Inf] |
| **2531** | NA | NA | NA | NA | NA |
| **2532** | NA | NA | NA | NA | NA |
| **2533** | NA | NA | NA | NA | NA |
| **2534** | NA | NA | NA | NA | NA |
| **2535** | NA | NA | NA | NA | NA |
| **2536** | NA | NA | (0.46,Inf] | NA | (0.798,1.72] |
| **2537** | NA | NA | (0.46,Inf] | NA | NA |
| **2538** | NA | NA | NA | NA | NA |
| **2539** | NA | NA | NA | NA | NA |
| **2540** | (0.00875,Inf] | NA | NA | NA | NA |
| **2541** | NA | NA | NA | NA | NA |
| **2542** | NA | NA | NA | NA | NA |

| | | | | | |
|---|---|---|---|---|---|
| **2543** | NA | NA | NA | NA | NA |
| **2544** | NA | NA | NA | NA | (1.72,Inf] |
| **2545** | NA | NA | NA | NA | NA |
| **2546** | NA | NA | NA | NA | NA |
| **2547** | NA | NA | NA | NA | (1.72,Inf] |
| **2548** | NA | NA | NA | NA | NA |
| **2549** | NA | NA | NA | NA | NA |
| **2550** | NA | NA | NA | NA | (1.72,Inf] |
| **2551** | NA | NA | NA | NA | (0.798,1.72] |
| **2552** | NA | NA | NA | NA | (1.72,Inf] |
| **2553** | NA | NA | (0.46,Inf] | NA | NA |
| **2554** | NA | NA | NA | NA | NA |
| **2555** | NA | NA | NA | NA | NA |
| **2556** | NA | NA | NA | NA | (1.72,Inf] |
| **2557** | NA | NA | NA | NA | NA |
| **2558** | NA | NA | NA | NA | (1.72,Inf] |
| **2559** | NA | NA | NA | NA | (1.72,Inf] |
| **2560** | NA | NA | NA | NA | (1.72,Inf] |
| **2561** | NA | NA | NA | NA | (1.72,Inf] |
| **2562** | NA | NA | NA | NA | NA |
| **2563** | NA | NA | NA | NA | NA |
| **2564** | NA | NA | NA | NA | (1.72,Inf] |
| **2565** | NA | NA | NA | NA | (1.72,Inf] |
| **2566** | NA | NA | NA | NA | (1.72,Inf] |
| **2567** | NA | NA | NA | NA | NA |
| **2568** | NA | NA | NA | NA | NA |
| **2569** | NA | NA | NA | NA | NA |
| **2570** | NA | NA | NA | NA | (1.72,Inf] |
| **2571** | NA | NA | (0.46,Inf] | NA | (1.72,Inf] |
| **2572** | NA | NA | NA | NA | (0.213,0.798] |
| **2573** | NA | NA | NA | NA | (1.72,Inf] |
| **2574** | NA | NA | NA | NA | (1.72,Inf] |
| **2575** | NA | NA | NA | NA | NA |
| **2576** | NA | NA | NA | NA | NA |
| **2577** | NA | NA | NA | NA | (1.72,Inf] |
| **2578** | NA | NA | NA | NA | (1.72,Inf] |
| **2579** | NA | NA | NA | NA | (1.72,Inf] |
| **2580** | NA | NA | NA | NA | NA |

| | | | | | |
|---|---|---|---|---|---|
| **2581** | NA | NA | NA | NA | (0.798,1.72] |
| **2582** | NA | NA | NA | NA | NA |
| **2583** | NA | NA | NA | NA | (1.72,Inf] |
| **2584** | NA | NA | NA | NA | (1.72,Inf] |
| **2585** | NA | NA | NA | NA | NA |
| **2586** | NA | NA | NA | NA | (1.72,Inf] |
| **2587** | NA | NA | NA | NA | NA |
| **2588** | NA | NA | NA | NA | (1.72,Inf] |
| **2589** | NA | NA | NA | NA | (0.213,0.798] |
| **2590** | NA | NA | NA | NA | (1.72,Inf] |
| **2591** | NA | NA | NA | NA | (1.72,Inf] |
| **2592** | NA | NA | NA | NA | NA |
| **2593** | NA | NA | (0.46,Inf] | NA | NA |
| **2594** | NA | NA | NA | NA | NA |
| **2595** | NA | NA | (0.46,Inf] | NA | NA |
| **2596** | NA | NA | NA | NA | NA |
| **2597** | NA | NA | NA | NA | NA |
| **2598** | NA | NA | NA | NA | (1.72,Inf] |
| **2599** | NA | NA | NA | NA | NA |
| **2600** | NA | NA | NA | NA | NA |
| **2601** | NA | NA | NA | NA | (1.72,Inf] |
| **2602** | NA | NA | NA | NA | (0.798,1.72] |
| **2603** | NA | NA | NA | NA | NA |
| **2604** | NA | NA | NA | NA | (1.72,Inf] |
| **2605** | NA | NA | NA | NA | NA |
| **2606** | NA | NA | NA | NA | (1.72,Inf] |
| **2607** | NA | NA | NA | NA | NA |
| **2608** | NA | NA | NA | NA | NA |
| **2609** | NA | NA | NA | NA | (1.72,Inf] |
| **2610** | NA | NA | NA | NA | NA |
| **2611** | NA | NA | NA | NA | NA |
| **2612** | NA | NA | NA | NA | (1.72,Inf] |
| **2613** | NA | NA | NA | NA | (0.798,1.72] |
| **2614** | NA | NA | NA | NA | (1.72,Inf] |
| **2615** | NA | NA | NA | NA | (1.72,Inf] |
| **2616** | NA | NA | NA | NA | (1.72,Inf] |
| **2617** | NA | NA | NA | NA | (1.72,Inf] |
| **2618** | NA | NA | (0.46,Inf] | NA | NA |

| | | | | | |
|---|---|---|---|---|---|
| **2619** | NA | NA | NA | NA | NA |
| **2620** | NA | NA | NA | NA | (1.72,Inf] |
| **2621** | NA | NA | NA | NA | (1.72,Inf] |
| **2622** | NA | NA | NA | NA | (1.72,Inf] |
| **2623** | NA | NA | NA | NA | (1.72,Inf] |
| **2624** | NA | NA | NA | NA | NA |
| **2625** | NA | NA | NA | NA | NA |
| **2626** | NA | NA | NA | NA | (1.72,Inf] |
| **2627** | NA | NA | (0.46,Inf] | NA | (1.72,Inf] |
| **2628** | NA | NA | NA | NA | NA |
| **2629** | NA | NA | NA | NA | NA |
| **2630** | NA | NA | (0.46,Inf] | NA | NA |
| **2631** | NA | NA | NA | NA | (1.72,Inf] |
| **2632** | NA | NA | NA | NA | NA |
| **2633** | NA | NA | NA | NA | (1.72,Inf] |
| **2634** | NA | NA | NA | NA | (1.72,Inf] |
| **2635** | NA | NA | NA | NA | NA |
| **2636** | NA | NA | (0.46,Inf] | NA | NA |
| **2637** | NA | NA | NA | NA | (1.72,Inf] |
| **2638** | NA | NA | NA | NA | (1.72,Inf] |
| **2639** | NA | NA | NA | NA | NA |
| **2640** | NA | NA | NA | NA | (1.72,Inf] |
| **2641** | NA | NA | NA | NA | NA |
| **2642** | NA | NA | NA | NA | NA |
| **2643** | NA | NA | (0.46,Inf] | NA | NA |
| **2644** | NA | NA | NA | NA | (1.72,Inf] |
| **2645** | NA | NA | NA | NA | (1.72,Inf] |
| **2646** | NA | NA | NA | NA | (1.72,Inf] |
| **2647** | NA | NA | NA | NA | (1.72,Inf] |
| **2648** | NA | NA | NA | NA | (1.72,Inf] |
| **2649** | NA | NA | NA | NA | NA |
| **2650** | NA | NA | NA | NA | (1.72,Inf] |
| **2651** | NA | NA | NA | NA | (1.72,Inf] |
| **2652** | NA | NA | NA | NA | (1.72,Inf] |
| **2653** | NA | NA | NA | NA | (1.72,Inf] |
| **2654** | NA | NA | NA | NA | (1.72,Inf] |
| **2655** | NA | NA | NA | NA | NA |
| **2656** | NA | NA | NA | NA | NA |

| | | | | | |
|---|---|---|---|---|---|
| **2657** | NA | NA | NA | NA | (1.72,Inf] |
| **2658** | NA | NA | (0.46,Inf] | NA | NA |
| **2659** | NA | NA | NA | NA | NA |
| **2660** | NA | NA | NA | NA | (1.72,Inf] |
| **2661** | NA | NA | NA | NA | (1.72,Inf] |
| **2662** | NA | NA | (0.46,Inf] | NA | (1.72,Inf] |
| **2663** | NA | NA | NA | NA | (1.72,Inf] |
| **2664** | NA | NA | NA | NA | NA |
| **2665** | NA | NA | NA | NA | (1.72,Inf] |
| **2666** | NA | NA | NA | NA | (1.72,Inf] |
| **2667** | NA | NA | NA | NA | (1.72,Inf] |
| **2668** | NA | NA | NA | NA | (1.72,Inf] |
| **2669** | NA | NA | NA | NA | (1.72,Inf] |
| **2670** | NA | NA | NA | NA | NA |
| **2671** | NA | NA | NA | NA | NA |
| **2672** | NA | NA | NA | NA | (1.72,Inf] |
| **2673** | NA | NA | NA | NA | (1.72,Inf] |
| **2674** | NA | NA | NA | NA | NA |
| **2675** | NA | NA | (0.46,Inf] | NA | NA |
| **2676** | NA | NA | NA | NA | (1.72,Inf] |
| **2677** | NA | NA | NA | NA | (1.72,Inf] |
| **2678** | NA | NA | NA | NA | NA |
| **2679** | NA | NA | (0.46,Inf] | NA | (1.72,Inf] |
| **2680** | NA | NA | NA | NA | (1.72,Inf] |
| **2681** | NA | NA | NA | NA | (1.72,Inf] |
| **2682** | NA | NA | (0.46,Inf] | NA | (1.72,Inf] |
| **2683** | NA | NA | NA | NA | (1.72,Inf] |
| **2684** | NA | NA | NA | NA | (1.72,Inf] |
| **2685** | NA | NA | (0.46,Inf] | NA | NA |
| **2686** | NA | NA | NA | NA | (1.72,Inf] |
| **2687** | NA | NA | NA | NA | (1.72,Inf] |
| **2688** | NA | NA | NA | NA | (1.72,Inf] |
| **2689** | NA | NA | NA | NA | NA |
| **2690** | NA | NA | NA | NA | (1.72,Inf] |
| **2691** | NA | NA | NA | NA | NA |
| **2692** | NA | NA | NA | NA | NA |
| **2693** | NA | NA | NA | NA | (1.72,Inf] |
| **2694** | NA | NA | NA | NA | (1.72,Inf] |

| | | | | | |
|---|---|---|---|---|---|
| **2695** | NA | NA | (0.46,Inf] | NA | (1.72,Inf] |
| **2696** | NA | NA | NA | NA | NA |
| **2697** | NA | NA | NA | NA | (1.72,Inf] |
| **2698** | NA | NA | NA | NA | (1.72,Inf] |
| **2699** | NA | NA | NA | NA | NA |
| **2700** | NA | NA | (0.46,Inf] | NA | (1.72,Inf] |
| **2701** | NA | NA | NA | NA | NA |
| **2702** | NA | NA | NA | NA | (1.72,Inf] |
| **2703** | NA | NA | NA | NA | (1.72,Inf] |
| **2704** | NA | NA | NA | NA | (1.72,Inf] |
| **2705** | NA | NA | NA | NA | (1.72,Inf] |
| **2706** | NA | NA | NA | NA | (1.72,Inf] |
| **2707** | NA | NA | NA | NA | (1.72,Inf] |
| **2708** | NA | NA | NA | NA | NA |
| **2709** | NA | NA | (0.46,Inf] | NA | NA |
| **2710** | NA | NA | (0.46,Inf] | NA | NA |
| **2711** | NA | NA | (0.46,Inf] | NA | NA |
| **2712** | NA | NA | (0.46,Inf] | NA | NA |
| **2713** | NA | NA | (0.46,Inf] | NA | NA |
| **2714** | NA | NA | (0.46,Inf] | NA | NA |
| **2715** | NA | NA | (0.46,Inf] | NA | NA |
| **2716** | NA | NA | (0.46,Inf] | NA | NA |
| **2717** | NA | NA | (0.46,Inf] | NA | NA |
| **2718** | NA | NA | (0.46,Inf] | NA | NA |
| **2719** | NA | NA | (0.46,Inf] | NA | NA |
| **2720** | NA | NA | (0.46,Inf] | NA | NA |
| **2721** | NA | NA | (0.46,Inf] | NA | NA |
| **2722** | NA | NA | (0.46,Inf] | NA | NA |
| **2723** | NA | NA | (0,0.46] | NA | NA |
